# Supplementary material for: GeneScanner: profiling genetic variation across bacterial populations
Source: Microb Genom. 2026 Jun 2;12(6):001714. doi: 10.1099/mgen.0.001714 (PMC13229410; doi:10.1099/mgen.0.001714)
Supplement: Supplementary Material 4. [file mgen-12-01714-s004.pdf]

>ref

ATGCAAATAGACCCCGTTGCATATCATTGTTCCGAGTTGAAGTTCGTGCTTGTACCCGAA

GAGTCGGGAAAGTTATCCCCGACGGAAATAGGATTCCATTTTCAGATTGGGTGCCGCGTA

CATGACAGAAGACCCCGCTCTGAGTCAGTGATGACGCACCAACGCAGATATCGATGTCTC

ACGTCTACCTTATTACTTGCCTTGATCGTCGGCACCCGATATTTGTCAACTGGGAATCCT

CCTGGACCTATTATCGGTCCTTTGAAACTAACAGCAATTACCCCAGAAAGTGTCTGCTAA

>1

ATGCAAATAGACCCCGTTGCATATCATTGTTCCGAGTTGAAGTTCGTGCTTGTACCCGAA

GAGTCGGGAAAGTTATCCCCGACGGAAATAGGATTCCATTTTCAGATTGGGTGCCGCGTA

CATGACAGAAGACCCCGCTCTGAGTCAGTGATGACGCACCAACGCAGATATCGATGTCTC

ACGTCTACCTTATTACTTGCCTTGATCGTCGGCACCCGATATTTGTCAACTGGGAATCCT

CCTGGACCTATTATCGGTCCTTTGAAACTAACAGCAATTACCCCAGAAAGTGTCTGCTAA

>2

ATGCAAATAGACCCCGTTGCATATCATTGTTCCGAGTTGAAGTTCGTGCTTGTACCCGAA

GAGTCGGGAAAGTTATCCCCGACGGAAATAGGATTCCATTTTCAGATTGGGTGCCGCGTA

CATGACAGAAGACCCCGCTCTGAGTCAGTGATGACGCACCAACGCAGATATCGATGTCTC

ACGTCTACCTTATTACTTGCCTTGATCGTCGGCACCCGATATTTGTCAACTGGGAATCCT

CCTGGACCTATTATCGGTCCTTTGAAACTAACAGCAATTACCCCAGAAAGTGTCTGCTAA

>3

ATGCAAATAGACCCCGTTGCATATCATTGTTCCGAGTTGAAGTTCGTGCTTGTACCCGAA

GAGTCGGGAAAGTTATCCCCGACGGAAATAGGATTCCATTTTCAGATTGGGTGCCGCGTA

CATGACAGAAGACCCCGCTCTGAGTCAGTGATGACGCACCAACGCAGATATCGATGTCTC

ACGTCTACCTTATTACTTGCCTTGATCGTCGGCACCCGATATTTGTCAACTGGGAATCCT

CCTGGACCTATTATCGGTCCTTTGAAACTAACAGCAATTACCCCAGAAAGTGTCTGCTAA

>4

ATGCAAATAGACCCCGTTGCATATCATTGTTCCGAGTTGAAGTTCGTGCTTGTACCCGAA

GAGTCGGGAAAGTTATCCCCGACGGAAATAGGATTCCATTTTCAGATTGGGTGCCGCGTA

CATGACAGAAGACCCCGCTCTGAGTCAGTGATGACGCACCAACGCAGATATCGATGTCTC

ACGTCTACCTTATTACTTGCCTTGATCGTCGGCACCCGATATTTGTCAACTGGGAATCCT

CCTGGACCTATTATCGGTCCTTTGAAACTAACAGCAATTACCCCAGAAAGTGTCTGCTAA

>5

ATGCAAATAGACCCCGTTGCATATCATTGTTCCGAGTTGAAGTTCGTGCTTGTACCCGAA

GAGTCGGGAAAGTTATCCCCGACGGAAATAGGATTCCATTTTCAGATTGGGTGCCGCGTA

CATGACAGAAGACCCCGCTCTGAGTCAGTGATGACGCACCAACGCAGATATCGATGTCTC

ACGTCTACCTTATTACTTGCCTTGATCGTCGGCACCCGATATTTGTCAACTGGGAATCCT

CCTGGACCTATTATCGGTCCTTTGAAACTAACAGCAATTACCCCAGAAAGTGTCTGCTAA

>6

ATGCAAATAGACCCCGTTGCATATCATTGTTCCGAGTTGAAGTTCGTGCTTGTACCCGAA

GAGTCGGGAAAGTTATCCCCGACGGAAATAGGATTCCATTTTCAGATTGGGTGCCGCGTA

CATGACAGAAGACCCCGCTCTGAGTCAGTGATGACGCACCAACGCAGATATCGATGTCTC

ACGTCTACCTTATTACTTGCCTTGATCGTCGGCACCCGATATTTGTCAACTGGGAATCCT

CCTGGACCTATTATCGGTCCTTTGAAACTAACAGCAATTACCCCAGAAAGTGTCTGCTAA

>7

ATGCAAATAGACCCCGTTGCATATCATTGTTCCGAGTTGAAGTTCGTGCTTGTACCCGAA

GAGTCGGGAAAGTTATCCCCGACGGAAATAGGATTCCATTTTCAGATTGGGTGCCGCGTA

CATGACAGAAGACCCCGCTCTGAGTCAGTGATGACGCACCAACGCAGATATCGATGTCTC

ACGTCTACCTTATTACTTGCCTTGATCGTCGGCACCCGATATTTGTCAACTGGGAATCCT

CCTGGACCTATTATCGGTCCTTTGAAACTAACAGCAATTACCCCAGAAAGTGTCTGCTAA

>8

ATGCAAATAGACCCCGTTGCATATCATTGTTCCGAGTTGAAGTTCGTGCTTGTACCCGAA

GAGTCGGGAAAGTTATCCCCGACGGAAATAGGATTCCATTTTCAGATTGGGTGCCGCGTA

CATGACAGAAGACCCCGCTCTGAGTCAGTGATGACGCACCAACGCAGATATCGATGTCTC

ACGTCTACCTTATTACTTGCCTTGATCGTCGGCACCCGATATTTGTCAACTGGGAATCCT

CCTGGACCTATTATCGGTCCTTTGAAACTAACAGCAATTACCCCAGAAAGTGTCTGCTAA

>9

ATGCAAATAGACCCCGTTGCATATCATTGTTCCGAGTTGAAGTTCGTGCTTGTACCCGAA

GAGTCGGGAAAGTTATCCCCGACGGAAATAGGATTCCATTTTCAGATTGGGTGCCGCGTA

CATGACAGAAGACCCCGCTCTGAGTCAGTGATGACGCACCAACGCAGATATCGATGTCTC

ACGTCTACCTTATTACTTGCCTTGATCGTCGGCACCCG-TATTTGTCAACTGGGAATCCT

CCTGGACCTATTATCGGTCCTTTGAAACTAACAGCAATTACCCCAGAAAGTGTCTGCTAA

>10

ATGCAAATAGACCCCGTTGCATATCATTGTTCCGAGTTGAAGTTCGTGCTTGTACCCGAA

GAGTCGGGAAAGTTATCCCCGACGGAAATAGGATTCCATTTTCAGATTGGGTGCCGCGTA

CATGACAGAAGACCCCGCTCTGAGTCAGTGATGACGCACCAACGCAGATATCGATGTCTC

ACGTCTACCTTATTACTTGCCTTGATCGTCGGCACCCGATATTTGTCAACTGGGAATCCT

CCTGGACCTATTATCGGTCCTTTGAAACTAACAGCAATTACCCCAGAAAGTGTCTGCTAA

>11

ATGCAAATAGACCCCGTTGCATATCATTGTTCCGAGTTGAAGTTCGTGCTTGTACCCGAA

GAGTCGGGAAAGTTATCCCCGACGGAAATAGGATTCCATTTTCAGATTGGGTGCCGCGTA

CATGACAGAAGACCCCGCTCTGAGTCAGTGATGACGCACCAACGCAGATATCGATGTCTC

ACGTCTACCTTATTACTTGCCTTGATCGTCGGCACCCGATATTTGTCAACTGGGAATCCT

CCTGGACCTATTATCGGTCCTTTGAAACTAACAGCAATTACCCCAGAAAGTGTCTGCTAA

>12

ATGCAAATAGACCCCGTTGCATATCATTGTTCCGAGTTGAAGTTCGTGCTTGTACCCGAA

GAGTCGGGAAAGTTATCCCCGACGGAAATAGGATTCCATTTTCAGATTGGGTGCCGCGTA

CATGACAGAAGACCCCGCTCTGAGTCAGTGATGACGCACCAACGCAGATATCGATGTCTC

ACGTCTACCTTATTACTTGCCTTGATCGTCGGCACCCGATATTTGTCAACTGGGAATCCT

CCTGGACCTATTATCGGTCCTTTGAAACTAACAGCAATTACCCCAGAAAGTGTCTGCTAA

>13

ATGCAAATAGACCCCGTTGCATATCATTGTTCCGAGTTGAAGTTCGTGCTTGTACCCGAA

GAGTCGGGAAAGTTATCCCCGACGGAAATAGGATTCCATTTTCAGATTGGGTGCCGCGTA

CATGACAGAAGACCCCGCTCTGAGTCAGTGATGACGCACCAACGCAGATATCGATGTCTC

ACGTCTACCTTATTACTTGCCTTGATCGTCGGCACCCGATATTTGTCAACTGGGAATCCT

CCTGGACCTATTATCGGTCCTTTGAAACTAACAGCAATTACCCCAGAAAGTGTCTGCTAA

>14

ATGCAAATAGACCCCGTTGCATATCATTGTTCCGAGTTGAAGTTCGTGCTTGTACCCGAA

GAGTCGGGAAAGTTATCCCCGACGGAAATAGGATTCCATTTTCAGATTGGGTGCCGCGTA

CATGACAGAAGACCCCGCTCTGAGTCAGTGATGACGCACCAACGCAGATATCGATGTCTC

ACGTCTACCTTATTACTTGCCTTGATCGTCGGCACCCGATATTTGTCAACTGGGAATCCT

CCTGGACCTATTATCGGTCCTTTGAAACTAACAGCAATTACCCCAGAAAGTGTCTGCTAA

>15

ATGCAAATAGACCCCGTTGCATATCATTGTTCCGAGTTGAAGTTCGTGCTTGTACCCGAA

GAGTCGGGAAAGTTATCCCCGACGGAAATAGGATTCCATTTTCAGATTGGGTGCCGCGTA

CATGACAGAAGACCCCGCTCTGAGTCAGTGATGACGCACCAACGCAGATATCGATGTCTC

ACGTCTACCTTATTACTTGCCTTGATCGTCGGCACCCGATATTTGTCAACTGGGAATCCT

CCTGGACCTATTATCGGTCCTTTGAAACTAACAGCAATTACCCCAGAAAGTGTCTGCTAA

>16

ATGCAAATAGACCCCGTTGCATATCATTGTTCCGAGTTGAAGTTCGTGCTTGTACCCGAA

GAGTCGGGAAAGTTATCCCCGACGGAAATAGGATTCCATTTTCAGATTGGGTGCCGCGTA

CATGACAGAAGACCCCGCTCTGAGTCAGTGATGACGCACCAACGCAGATATCGATGTCTC

ACGTCTACCTTATTACTTGCCTTGATCGTCGGCACCCGATATTTGTCAACTGGGAATCCT

CCTGGACCTATTATCGGTCCTTTGAAACTAACAGCAATTACCCCAGAAAGTGTCTGCTAA

>17

ATGCAAATAGACCCCGTTGCATATCATTGTTCCGAGTTGAAGTTCGTGCTTGTACCCGAA

GAGTCGGGAAAGTTATCCCCGACGGAAATAGGATTCCATTTTCAGATTGGGTGCCGCGTA

CATGACAGAAGACCCCGCTCTGAGTCAGTGATGACGCACCAACGCAGATATCGATGTCTC

ACGTCTACCTTATTACTTGCCTTGATCGTCGGCACCCGATATTTGTCAACTGGGAATCCT

CCTGGACCTATTATCGGTCCTTTGAAACTAACAGCAATTACCCCAGAAAGTGTCTGCTAA

>18

ATGCAAATAGACCCCGTTGCATATCATTGTTCCGAGTTGAAGTTCGTGCTTGTACCCGAA

GAGTCGGGAAAGTTATCCCCGACGGAAATAGGATTCCATTTTCAGATTGGGTGCCGCGTA

CATGACAGAAGACCCCGCTCTGAGTCAGTGATGACGCACCAACGCAGATATCGATGTCTC

ACGTCTACCTTATTACTTGCCTTGATCGTCGGCACCCG-TATTTGTCAACTGGGAATCCT

CCTGGACCTATTATCGGTCCTTTGAAACTAACAGCAATTACCCCAGAAAGTGTCTGCTAA

>19

ATGCAAATAGACCCCGTTGCATATCATTGTTCCGAGTTGAAGTTCGTGCTTGTACCCGAA

GAGTCGGGAAAGTTATCCCCGACGGAAATAGGATTCCATTTTCAGATTGGGTGCCGCGTA

CATGACAGAAGACCCCGCTCTGAGTCAGTGATGACGCACCAACGCAGATATCGATGTCTC

ACGTCTACCTTATTACTTGCCTTGATCGTCGGCACCCGATATTTGTCAACTGGGAATCCT

CCTGGACCTATTATCGGTCCTTTGAAACTAACAGCAATTACCCCAGAAAGTGTCTGCTAA

>20

ATGCAAATAGACCCCGTTGCATATCATTGTTCCGAGTTGAAGTTCGTGCTTGTACCCGAA

GAGTCGGGAAAGTTATCCCCGACGGAAATAGGATTCCATTTTCAGATTGGGTGCCGCGTA

CATGACAGAAGACCCCGCTCTGAGTCAGTGATGACGCACCAACGCAGATATCGATGTCTC

ACGTCTACCTTATTACTTGCCTTGATCGTCGGCACCCGATATTTGTCAACTGGGAATCCT

CCTGGACCTATTATCGGTCCTTTGAAACTAACAGCAATTACCCCAGAAAGTGTCTGCTAA

>21

ATG-AAATAGACCCCGTTGCATATCATTGTTCCGAGTTGAAGTTCGTGCTTGTACCCGAA

GAGTCGGGAAAGTTATCCCCGACGGAAATAGGATTCCATTTTCAGATTGGGTGCCGCGTA

CATGACAGAAGACCCCGCTCTGAGTCAGTGATGACGCACCAACGCAGATATCGATGTCTC

ACGTCTACCTTATTACTTGCCTTGATCGTCGGCACCCGATATTTGTCAACTGGGAATCCT

CCTGGACCTATTATCGGTCCTTTGAAACTAACAGCAATTACCCCAGAAAGTGTCTGCTAA

>22

ATGCAAATAGACCCCGTTGCATATCATTGTTCCGAGTTGAAGTTCGTGCTTGTACCCGAA

GAGTCGGGAAAGTTATCCCCGACGGAAATAGGATTCCATTTTCAGATTGGGTGCCGCGTA

CATGACAGAAGACCCCGCTCTGAGTCAGTGATGACGCACCAACGCAGATATCGATGTCTC

ACGTCTACCTTATTACTTGCCTTGATCGTCGGCACCCGATATTTGTCAACTGGGAATCCT

CCTGGACCTATTATCGGTCCTTTGAAACTAACAGCAATTACCCCAGAAAGTGTCTGCTAA

>23

ATGCAAATAGACCCCGTTGCATATCATTGTTCCGAGTTGAAGTTCGTGCTTGTACCCGAA

GAGTCGGGAAAGTTATCCCCGACGGAAATAGGATTCCATTTTCAGATTGGGTGCCGCGTA

CATGACAGAAGACCCCGCTCTGAGTCAGTGATGACGCACCAACGCAGATATCGATGTCTC

ACGTCTACCTTATTACTTGCCTTGATCGTCGGCACCCGATATTTGTCAACTGGGAATCCT

CCTGGACCTATTATCGGTCCTTTGAAACTAACAGCAATTACCCCAGAAAGTGTCTGCTAA

>24

ATGCAAATAGACCCCGTTGCATATCATTGTTCCGAGTTGAAGTTCGTGCTTGTACCCGAA

GAGTCGGGAAAGTTATCCCCGACGGAAATAGGATTCCATTTTCAGATTGGGTGCCGCGTA

CATGACAGAAGACCCCGCTCTGAGTCAGTGATGACGCACCAACGCAGATATCGATGTCTC

ACGTCTACCTTATTACTTGCCTTGATCGTCGGCACCCGATATTTGTCAACTGGGAATCCT

CCTGGACCTATTATCGGTCCTTTGAAACTAACAGCAATTACCCCAGAAAGTGTCTGCTAA

>25

ATGCAAATAGACCCCGTTGCATATCATTGTTCCGAGTTGAAGTTCGTGCTTGTACCCGAA

GAGTCGGGAAAGTTATCCCCGACGGAAATAGGATTCCATTTTCAGATTGGGTGCCGCGTA

CATGACAGAAGACCCCGCTCTGAGTCAGTGATGACGCACCAACGCAGATATCGATGTCTC

ACGTCTACCTTATTACTTGCCTTGATCGTCGGCACCCGATATTTGTCAACTGGGAATCCT

CCTGGACCTATTATCGGTCCTTTGAAACTAACAGCAATTACCCCAGAAAGTGTCTGCTAA

>26

ATGCAAATAGACCCCGTTGCATATCATTGTTCCGAGTTGAAGTTCGTGCTTGTACCCGAA

GAGTCGGGAAAGTTATCCCCGACGGAAATAGGATTCCATTTTCAGATTGGGTGCCGCGTA

CATGACAGAAGACCCCGCTCTGAGTCAGTGATGACGCACCAACGCAGATATCGATGTCTC

ACGTCTACCTTATTACTTGCCTTGATCGTCGGCACCCGATATTTGTCAACTGGGAATCCT

CCTGGACCTATTATCGGTCCTTTGAAACTAACAGCAATTACCCCAGAAAGTGTCTGCTAA

>27

ATGCAAATAGACCCCGTTGCATATCATTGTTCCGAGTTGAAGTTCGTGCTTGTACCCGAA

GAGTCGGGAAAGTTATCCCCGACGGAAATAGGATTCCATTTTCAGATTGGGTGCCGCGTA

CATGACAGAAGACCCCGCTCTGAGTCAGTGATGACGCACCAACGCAGATATCGATGTCTC

ACGTCTACCTTATTACTTGCCTTGATCGTCGGCACCCGATATTTGTCAACTGGGAATCCT

CCTGGACCTATTATCGGTCCTTTGAAACTAACAGCAATTACCCCAGAAAGTGTCTGCTAA

>28

ATGCAAATAGACCCCGTTGCATATCATTGTTCCGAGTTGAAGTTCGTGCTTGTACCCGAA

GAGTCGGGAAAGTTATCCCCGACGGAAATAGGATTCCATTTTCAGATTGGGTGCCGCGTA

CATGACAGAAGACCCCGCTCTGAGTCAGTGATGACGCACCAACGCAGATATCGATGTCTC

ACGTCTACCTTATTACTTGCCTTGATCGTCGGCACCCGATATTTGTCAACTGGGAATCCT

CCTGGACCTATTATCGGTCCTTTGAAACTAACAGCAATTACCCCAGAAAGTGTCTGCTAA

>29

ATGCAAATAGACCCCGTTGCATATCATTGTTCCGAGTTGAAGTTCGTGCTTGTACCCGAA

GAGTCGGGAAAGTTATCCCCGACGGAAATAGGATTCCATTTTCAGATTGGGTGCCGCGTA

CATGACAGAAGACCCCGCTCTGAGTCAGTGATGACGCACCAACGCAGATATCGATGTCTC

ACGTCTACCTTATTACTTGCCTTGATCGTCGGCACCCG-TATTTGTCAACTGGGAATCCT

CCTGGACCTATTATCGGTCCTTTGAAACTAACAGCAATTACCCCAGAAAGTGTCTGCTAA

>30

ATGCAAATAGACCCCGTTGCATATCATTGTTCCGAGTTGAAGTTCGTGCTTGTACCCGAA

GAGTCGGGAAAGTTATCCCCGACGGAAATAGGATTCCATTTTCAGATTGGGTGCCGCGTA

CATGACAGAAGACCCCGCTCTGAGTCAGTGATGACGCACCAACGCAGATATCGATGTCTC

ACGTCTACCTTATTACTTGCCTTGATCGTCGGCACCCGATATTTGTCAACTGGGAATCCT

CCTGGACCTATTATCGGTCCTTTGAAACTAACAGCAATTACCCCAGAAAGTGTCTGCTAA

>31

ATG-AAATAGACCCCGTTGCATATCATTGTTCCGAGTTGAAGTTCGTGCTTGTACCCGAA

GAGTCGGGAAAGTTATCCCCGACGGAAATAGGATTCCATTTTCAGATTGGGTGCCGCGTA

CATGACAGAAGACCCCGCTCTGAGTCAGTGATGACGCACCAACGCAGATATCGATGTCTC

ACGTCTACCTTATTACTTGCCTTGATCGTCGGCACCCGATATTTGTCAACTGGGAATCCT

CCTGGACCTATTATCGGTCCTTTGAAACTAACAGCAATTACCCCAGAAAGTGTCTGCTAA

>32

ATGCAAATAGACCCCGTTGCATATCATTGTTCCGAGTTGAAGTTCGTGCTTGTACCCGAA

GAGTCGGGAAAGTTATCCCCGACGGAAATAGGATTCCATTTTCAGATTGGGTGCCGCGTA

CATGACAGAAGACCCCGCTCTGAGTCAGTGATGACGCACCAACGCAGATATCGATGTCTC

ACGTCTACCTTATTACTTGCCTTGATCGTCGGCACCCGATATTTGTCAACTGGGAATCCT

CCTGGACCTATTATCGGTCCTTTGAAACTAACAGCAATTACCCCAGAAAGTGTCTGCTAA

>33

ATGCAAATAGACCCCGTTGCATATCATTGTTCCGAGTTGAAGTTCGTGCTTGTACCCGAA

GAGTCGGGAAAGTTATCCCCGACGGAAATAGGATTCCATTTTCAGATTGGGTGCCGCGTA

CATGACAGAAGACCCCGCTCTGAGTCAGTGATGACGCACCAACGCAGATATCGATGTCTC

ACGTCTACCTTATTACTTGCCTTGATCGTCGGCACCCGATATTTGTCAACTGGGAATCCT

CCTGGACCTATTATCGGTCCTTTGAAACTAACAGCAATTACCCCAGAAAGTGTCTGCTAA

>34

ATGCAAATAGACCCCGTTGCATATCATTGTTCCGAGTTGAAGTTCGTGCTTGTACCCGAA

GAGTCGGGAAAGTTATCCCCGACGGAAATAGGATTCCATTTTCAGATTGGGTGCCGCGTA

CATGACAGAAGACCCCGCTCTGAGTCAGTGATGACGCACCAACGCAGATATCGATGTCTC

ACGTCTACCTTATTACTTGCCTTGATCGTCGGCACCCGATATTTGTCAACTGGGAATCCT

CCTGGACCTATTATCGGTCCTTTGAAACTAACAGCAATTACCCCAGAAAGTGTCTGCTAA

>35

ATGCAAATAGACCCCGTTGCATATCATTGTTCCGAGTTGAAGTTCGTGCTTGTACCCGAA

GAGTCGGGAAAGTTATCCCCGACGGAAATAGGATTCCATTTTCAGATTGGGTGCCGCGTA

CATGACAGAAGACCCCGCTCTGAGTCAGTGATGACGCACCAACGCAGATATCGATGTCTC

ACGTCTACCTTATTACTTGCCTTGATCGTCGGCACCCG-TATTTGTCAACTGGGAATCCT

CCTGGACCTATTATCGGTCCTTTGAAACTAACAGCAATTACCCCAGAAAGTGTCTGCTAA

>36

ATGCAAATAGACCCCGTTGCATATCATTGTTCCGAGTTGAAGTTCGTGCTTGTACCCGAA

GAGTCGGGAAAGTTATCCCCGACGGAAATAGGATTCCATTTTCAGATTGGGTGCCGCGTA

CATGACAGAAGACCCCGCTCTGAGTCAGTGATGACGCACCAACGCAGATATCGATGTCTC

ACGTCTACCTTATTACTTGCCTTGATCGTCGGCACCCGATATTTGTCAACTGGGAATCCT

CCTGGACCTATTATCGGTCCTTTGAAACTAACAGCAATTACCCCAGAAAGTGTCTGCTAA

>37

ATGCAAATAGACCCCGTTGCATATCATTGTTCCGAGTTGAAGTTCGTGCTTGTACCCGAA

GAGTCGGGAAAGTTATCCCCGACGGAAATAGGATTCCATTTTCAGATTGGGTGCCGCGTA

CATGACAGAAGACCCCGCTCTGAGTCAGTGATGACGCACCAACGCAGATATCGATGTCTC

ACGTCTACCTTATTACTTGCCTTGATCGTCGGCACCCGATATTTGTCAACTGGGAATCCT

CCTGGACCTATTATCGGTCCTTTGAAACTAACAGCAATTACCCCAGAAAGTGTCTGCTAA

>38

ATG-AAATAGACCCCGTTGCATATCATTGTTCCGAGTTGAAGTTCGTGCTTGTACCCGAA

GAGTCGGGAAAGTTATCCCCGACGGAAATAGGATTCCATTTTCAGATTGGGTGCCGCGTA

CATGACAGAAGACCCCGCTCTGAGTCAGTGATGACGCACCAACGCAGATATCGATGTCTC

ACGTCTACCTTATTACTTGCCTTGATCGTCGGCACCCGATATTTGTCAACTGGGAATCCT

CCTGGACCTATTATCGGTCCTTTGAAACTAACAGCAATTACCCCAGAAAGTGTCTGCTAA

>39

ATG-AAATAGACCCCGTTGCATATCATTGTTCCGAGTTGAAGTTCGTGCTTGTACCCGAA

GAGTCGGGAAAGTTATCCCCGACGGAAATAGGATTCCATTTTCAGATTGGGTGCCGCGTA

CATGACAGAAGACCCCGCTCTGAGTCAGTGATGACGCACCAACGCAGATATCGATGTCTC

ACGTCTACCTTATTACTTGCCTTGATCGTCGGCACCCGATATTTGTCAACTGGGAATCCT

CCTGGACCTATTATCGGTCCTTTGAAACTAACAGCAATTACCCCAGAAAGTGTCTGCTAA

>40

ATGCAAATAGACCCCGTTGCATATCATTGTTCCGAGTTGAAGTTCGTGCTTGTACCCGAA

GAGTCGGGAAAGTTATCCCCGACGGAAATAGGATTCCATTTTCAGATTGGGTGCCGCGTA

CATGACAGAAGACCCCGCTCTGAGTCAGTGATGACGCACCAACGCAGATATCGATGTCTC

ACGTCTACCTTATTACTTGCCTTGATCGTCGGCACCCGATATTTGTCAACTGGGAATCCT

CCTGGACCTATTATCGGTCCTTTGAAACTAACAGCAATTACCCCAGAAAGTGTCTGCTAA

>41

ATGCAAATAGACCCCGTTGCATATCATTGTTCCGAGTTGAAGTTCGTGCTTGTACCCGAA

GAGTCGGGAAAGTTATCCCCGACGGAAATAGGATTCCATTTTCAGATTGGGTGCCGCGTA

CATGACAGAAGACCCCGCTCTGAGTCAGTGATGACGCACCAACGCAGATATCGATGTCTC

ACGTCTACCTTATTACTTGCCTTGATCGTCGGCACCCGATATTTGTCAACTGGGAATCCT

CCTGGACCTATTATCGGTCCTTTGAAACTAACAGCAATTACCCCAGAAAGTGTCTGCTAA

>42

ATGCAAATAGACCCCGTTGCATATCATTGTTCCGAGTTGAAGTTCGTGCTTGTACCCGAA

GAGTCGGGAAAGTTATCCCCGACGGAAATAGGATTCCATTTTCAGATTGGGTGCCGCGTA

CATGACAGAAGACCCCGCTCTGAGTCAGTGATGACGCACCAACGCAGATATCGATGTCTC

ACGTCTACCTTATTACTTGCCTTGATCGTCGGCACCCGATATTTGTCAACTGGGAATCCT

CCTGGACCTATTATCGGTCCTTTGAAACTAACAGCAATTACCCCAGAAAGTGTCTGCTAA

>43

ATGCAAATAGACCCCGTTGCATATCATTGTTCCGAGTTGAAGTTCGTGCTTGTACCCGAA

GAGTCGGGAAAGTTATCCCCGACGGAAATAGGATTCCATTTTCAGATTGGGTGCCGCGTA

CATGACAGAAGACCCCGCTCTGAGTCAGTGATGACGCACCAACGCAGATATCGATGTCTC

ACGTCTACCTTATTACTTGCCTTGATCGTCGGCACCCGATATTTGTCAACTGGGAATCCT

CCTGGACCTATTATCGGTCCTTTGAAACTAACAGCAATTACCCCAGAAAGTGTCTGCTAA

>44

ATGCAAATAGACCCCGTTGCATATCATTGTTCCGAGTTGAAGTTCGTGCTTGTACCCGAA

GAGTCGGGAAAGTTATCCCCGACGGAAATAGGATTCCATTTTCAGATTGGGTGCCGCGTA

CATGACAGAAGACCCCGCTCTGAGTCAGTGATGACGCACCAACGCAGATATCGATGTCTC

ACGTCTACCTTATTACTTGCCTTGATCGTCGGCACCCGATATTTGTCAACTGGGAATCCT

CCTGGACCTATTATCGGTCCTTTGAAACTAACAGCAATTACCCCAGAAAGTGTCTGCTAA

>45

ATGCAAATAGACCCCGTTGCATATCATTGTTCCGAGTTGAAGTTCGTGCTTGTACCCGAA

GAGTCGGGAAAGTTATCCCCGACGGAAATAGGATTCCATTTTCAGATTGGGTGCCGCGTA

CATGACAGAAGACCCCGCTCTGAGTCAGTGATGACGCACCAACGCAGATATCGATGTCTC

ACGTCTACCTTATTACTTGCCTTGATCGTCGGCACCCGATATTTGTCAACTGGGAATCCT

CCTGGACCTATTATCGGTCCTTTGAAACTAACAGCAATTACCCCAGAAAGTGTCTGCTAA

>46

ATGCAAATAGACCCCGTTGCATATCATTGTTCCGAGTTGAAGTTCGTGCTTGTACCCGAA

GAGTCGGGAAAGTTATCCCCGACGGAAATAGGATTCCATTTTCAGATTGGGTGCCGCGTA

CATGACAGAAGACCCCGCTCTGAGTCAGTGATGACGCACCAACGCAGATATCGATGTCTC

ACGTCTACCTTATTACTTGCCTTGATCGTCGGCACCCGATATTTGTCAACTGGGAATCCT

CCTGGACCTATTATCGGTCCTTTGAAACTAACAGCAATTACCCCAGAAAGTGTCTGCTAA

>47

ATGCAAATAGACCCCGTTGCATATCATTGTTCCGAGTTGAAGTTCGTGCTTGTACCCGAA

GAGTCGGGAAAGTTATCCCCGACGGAAATAGGATTCCATTTTCAGATTGGGTGCCGCGTA

CATGACAGAAGACCCCGCTCTGAGTCAGTGATGACGCACCAACGCAGATATCGATGTCTC

ACGTCTACCTTATTACTTGCCTTGATCGTCGGCACCCGATATTTGTCAACTGGGAATCCT

CCTGGACCTATTATCGGTCCTTTGAAACTAACAGCAATTACCCCAGAAAGTGTCTGCTAA

>48

ATGCAAATAGACCCCGTTGCATATCATTGTTCCGAGTTGAAGTTCGTGCTTGTACCCGAA

GAGTCGGGAAAGTTATCCCCGACGGAAATAGGATTCCATTTTCAGATTGGGTGCCGCGTA

CATGACAGAAGACCCCGCTCTGAGTCAGTGATGACGCACCAACGCAGATATCGATGTCTC

ACGTCTACCTTATTACTTGCCTTGATCGTCGGCACCCGATATTTGTCAACTGGGAATCCT

CCTGGACCTATTATCGGTCCTTTGAAACTAACAGCAATTACCCCAGAAAGTGTCTGCTAA

>49

ATGCAAATAGACCCCGTTGCATATCATTGTTCCGAGTTGAAGTTCGTGCTTGTACCCGAA

GAGTCGGGAAAGTTATCCCCGACGGAAATAGGATTCCATTTTCAGATTGGGTGCCGCGTA

CATGACAGAAGACCCCGCTCTGAGTCAGTGATGACGCACCAACGCAGATATCGATGTCTC

ACGTCTACCTTATTACTTGCCTTGATCGTCGGCACCCGATATTTGTCAACTGGGAATCCT

CCTGGACCTATTATCGGTCCTTTGAAACTAACAGCAATTACCCCAGAAAGTGTCTGCTAA

>50

ATGCAAATAGACCCCGTTGCATATCATTGTTCCGAGTTGAAGTTCGTGCTTGTACCCGAA

GAGTCGGGAAAGTTATCCCCGACGGAAATAGGATTCCATTTTCAGATTGGGTGCCGCGTA

CATGACAGAAGACCCCGCTCTGAGTCAGTGATGACGCACCAACGCAGATATCGATGTCTC

ACGTCTACCTTATTACTTGCCTTGATCGTCGGCACCCGATATTTGTCAACTGGGAATCCT

CCTGGACCTATTATCGGTCCTTTGAAACTAACAGCAATTACCCCAGAAAGTGTCTGCTAA

>51

ATGCAAATAGACCCCGTTGCATATCATTGTTCCGAGTTGAAGTTCGTGCTTGTACCCGAA

GAGTCGGGAAAGTTATCCCCGACGGAAATAGGATTCCATTTTCAGATTGGGTGCCGCGTA

CATGACAGAAGACCCCGCTCTGAGTCAGTGATGACGCACCAACGCAGATATCGATGTCTC

ACGTCTACCTTATTACTTGCCTTGATCGTCGGCACCCGATATTTGTCAACTGGGAATCCT

CCTGGACCTATTATCGGTCCTTTGAAACTAACAGCAATTACCCCAGAAAGTGTCTGCTAA

>52

ATG-AAATAGACCCCGTTGCATATCATTGTTCCGAGTTGAAGTTCGTGCTTGTACCCGAA

GAGTCGGGAAAGTTATCCCCGACGGAAATAGGATTCCATTTTCAGATTGGGTGCCGCGTA

CATGACAGAAGACCCCGCTCTGAGTCAGTGATGACGCACCAACGCAGATATCGATGTCTC

ACGTCTACCTTATTACTTGCCTTGATCGTCGGCACCCGATATTTGTCAACTGGGAATCCT

CCTGGACCTATTATCGGTCCTTTGAAACTAACAGCAATTACCCCAGAAAGTGTCTGCTAA

>53

ATGCAAATAGACCCCGTTGCATATCATTGTTCCGAGTTGAAGTTCGTGCTTGTACCCGAA

GAGTCGGGAAAGTTATCCCCGACGGAAATAGGATTCCATTTTCAGATTGGGTGCCGCGTA

CATGACAGAAGACCCCGCTCTGAGTCAGTGATGACGCACCAACGCAGATATCGATGTCTC

ACGTCTACCTTATTACTTGCCTTGATCGTCGGCACCCGATATTTGTCAACTGGGAATCCT

CCTGGACCTATTATCGGTCCTTTGAAACTAACAGCAATTACCCCAGAAAGTGTCTGCTAA

>54

ATGCAAATAGACCCCGTTGCATATCATTGTTCCGAGTTGAAGTTCGTGCTTGTACCCGAA

GAGTCGGGAAAGTTATCCCCGACGGAAATAGGATTCCATTTTCAGATTGGGTGCCGCGTA

CATGACAGAAGACCCCGCTCTGAGTCAGTGATGACGCACCAACGCAGATATCGATGTCTC

ACGTCTACCTTATTACTTGCCTTGATCGTCGGCACCCGATATTTGTCAACTGGGAATCCT

CCTGGACCTATTATCGGTCCTTTGAAACTAACAGCAATTACCCCAGAAAGTGTCTGCTAA

>55

ATGCAAATAGACCCCGTTGCATATCATTGTTCCGAGTTGAAGTTCGTGCTTGTACCCGAA

GAGTCGGGAAAGTTATCCCCGACGGAAATAGGATTCCATTTTCAGATTGGGTGCCGCGTA

CATGACAGAAGACCCCGCTCTGAGTCAGTGATGACGCACCAACGCAGATATCGATGTCTC

ACGTCTACCTTATTACTTGCCTTGATCGTCGGCACCCGATATTTGTCAACTGGGAATCCT

CCTGGACCTATTATCGGTCCTTTGAAACTAACAGCAATTACCCCAGAAAGTGTCTGCTAA

>56

ATGCAAATAGACCCCGTTGCATATCATTGTTCCGAGTTGAAGTTCGTGCTTGTACCCGAA

GAGTCGGGAAAGTTATCCCCGACGGAAATAGGATTCCATTTTCAGATTGGGTGCCGCGTA

CATGACAGAAGACCCCGCTCTGAGTCAGTGATGACGCACCAACGCAGATATCGATGTCTC

ACGTCTACCTTATTACTTGCCTTGATCGTCGGCACCCGATATTTGTCAACTGGGAATCCT

CCTGGACCTATTATCGGTCCTTTGAAACTAACAGCAATTACCCCAGAAAGTGTCTGCTAA

>57

ATG-AAATAGACCCCGTTGCATATCATTGTTCCGAGTTGAAGTTCGTGCTTGTACCCGAA

GAGTCGGGAAAGTTATCCCCGACGGAAATAGGATTCCATTTTCAGATTGGGTGCCGCGTA

CATGACAGAAGACCCCGCTCTGAGTCAGTGATGACGCACCAACGCAGATATCGATGTCTC

ACGTCTACCTTATTACTTGCCTTGATCGTCGGCACCCGATATTTGTCAACTGGGAATCCT

CCTGGACCTATTATCGGTCCTTTGAAACTAACAGCAATTACCCCAGAAAGTGTCTGCTAA

>58

ATGCAAATAGACCCCGTTGCATATCATTGTTCCGAGTTGAAGTTCGTGCTTGTACCCGAA

GAGTCGGGAAAGTTATCCCCGACGGAAATAGGATTCCATTTTCAGATTGGGTGCCGCGTA

CATGACAGAAGACCCCGCTCTGAGTCAGTGATGACGCACCAACGCAGATATCGATGTCTC

ACGTCTACCTTATTACTTGCCTTGATCGTCGGCACCCGATATTTGTCAACTGGGAATCCT

CCTGGACCTATTATCGGTCCTTTGAAACTAACAGCAATTACCCCAGAAAGTGTCTGCTAA

>59

ATGCAAATAGACCCCGTTGCATATCATTGTTCCGAGTTGAAGTTCGTGCTTGTACCCGAA

GAGTCGGGAAAGTTATCCCCGACGGAAATAGGATTCCATTTTCAGATTGGGTGCCGCGTA

CATGACAGAAGACCCCGCTCTGAGTCAGTGATGACGCACCAACGCAGATATCGATGTCTC

ACGTCTACCTTATTACTTGCCTTGATCGTCGGCACCCGATATTTGTCAACTGGGAATCCT

CCTGGACCTATTATCGGTCCTTTGAAACTAACAGCAATTACCCCAGAAAGTGTCTGCTAA

>60

ATGCAAATAGACCCCGTTGCATATCATTGTTCCGAGTTGAAGTTCGTGCTTGTACCCGAA

GAGTCGGGAAAGTTATCCCCGACGGAAATAGGATTCCATTTTCAGATTGGGTGCCGCGTA

CATGACAGAAGACCCCGCTCTGAGTCAGTGATGACGCACCAACGCAGATATCGATGTCTC

ACGTCTACCTTATTACTTGCCTTGATCGTCGGCACCCGATATTTGTCAACTGGGAATCCT

CCTGGACCTATTATCGGTCCTTTGAAACTAACAGCAATTACCCCAGAAAGTGTCTGCTAA

>61

ATGCAAATAGACCCCGTTGCATATCATTGTTCCGAGTTGAAGTTCGTGCTTGTACCCGAA

GAGTCGGGAAAGTTATCCCCGACGGAAATAGGATTCCATTTTCAGATTGGGTGCCGCGTA

CATGACAGAAGACCCCGCTCTGAGTCAGTGATGACGCACCAACGCAGATATCGATGTCTC

ACGTCTACCTTATTACTTGCCTTGATCGTCGGCACCCGATATTTGTCAACTGGGAATCCT

CCTGGACCTATTATCGGTCCTTTGAAACTAACAGCAATTACCCCAGAAAGTGTCTGCTAA

>62

ATGCAAATAGACCCCGTTGCATATCATTGTTCCGAGTTGAAGTTCGTGCTTGTACCCGAA

GAGTCGGGAAAGTTATCCCCGACGGAAATAGGATTCCATTTTCAGATTGGGTGCCGCGTA

CATGACAGAAGACCCCGCTCTGAGTCAGTGATGACGCACCAACGCAGATATCGATGTCTC

ACGTCTACCTTATTACTTGCCTTGATCGTCGGCACCCGATATTTGTCAACTGGGAATCCT

CCTGGACCTATTATCGGTCCTTTGAAACTAACAGCAATTACCCCAGAAAGTGTCTGCTAA

>63

ATGCAAATAGACCCCGTTGCATATCATTGTTCCGAGTTGAAGTTCGTGCTTGTACCCGAA

GAGTCGGGAAAGTTATCCCCGACGGAAATAGGATTCCATTTTCAGATTGGGTGCCGCGTA

CATGACAGAAGACCCCGCTCTGAGTCAGTGATGACGCACCAACGCAGATATCGATGTCTC

ACGTCTACCTTATTACTTGCCTTGATCGTCGGCACCCGATATTTGTCAACTGGGAATCCT

CCTGGACCTATTATCGGTCCTTTGAAACTAACAGCAATTACCCCAGAAAGTGTCTGCTAA

>64

ATGCAAATAGACCCCGTTGCATATCATTGTTCCGAGTTGAAGTTCGTGCTTGTACCCGAA

GAGTCGGGAAAGTTATCCCCGACGGAAATAGGATTCCATTTTCAGATTGGGTGCCGCGTA

CATGACAGAAGACCCCGCTCTGAGTCAGTGATGACGCACCAACGCAGATATCGATGTCTC

ACGTCTACCTTATTACTTGCCTTGATCGTCGGCACCCGATATTTGTCAACTGGGAATCCT

CCTGGACCTATTATCGGTCCTTTGAAACTAACAGCAATTACCCCAGAAAGTGTCTGCTAA

>65

ATGCAAATAGACCCCGTTGCATATCATTGTTCCGAGTTGAAGTTCGTGCTTGTACCCGAA

GAGTCGGGAAAGTTATCCCCGACGGAAATAGGATTCCATTTTCAGATTGGGTGCCGCGTA

CATGACAGAAGACCCCGCTCTGAGTCAGTGATGACGCACCAACGCAGATATCGATGTCTC

ACGTCTACCTTATTACTTGCCTTGATCGTCGGCACCCGATATTTGTCAACTGGGAATCCT

CCTGGACCTATTATCGGTCCTTTGAAACTAACAGCAATTACCCCAGAAAGTGTCTGCTAA

>66

ATGCAAATAGACCCCGTTGCATATCATTGTTCCGAGTTGAAGTTCGTGCTTGTACCCGAA

GAGTCGGGAAAGTTATCCCCGACGGAAATAGGATTCCATTTTCAGATTGGGTGCCGCGTA

CATGACAGAAGACCCCGCTCTGAGTCAGTGATGACGCACCAACGCAGATATCGATGTCTC

ACGTCTACCTTATTACTTGCCTTGATCGTCGGCACCCGATATTTGTCAACTGGGAATCCT

CCTGGACCTATTATCGGTCCTTTGAAACTAACAGCAATTACCCCAGAAAGTGTCTGCTAA

>67

ATGCAAATAGACCCCGTTGCATATCATTGTTCCGAGTTGAAGTTCGTGCTTGTACCCGAA

GAGTCGGGAAAGTTATCCCCGACGGAAATAGGATTCCATTTTCAGATTGGGTGCCGCGTA

CATGACAGAAGACCCCGCTCTGAGTCAGTGATGACGCACCAACGCAGATATCGATGTCTC

ACGTCTACCTTATTACTTGCCTTGATCGTCGGCACCCGATATTTGTCAACTGGGAATCCT

CCTGGACCTATTATCGGTCCTTTGAAACTAACAGCAATTACCCCAGAAAGTGTCTGCTAA

>68

ATG-AAATAGACCCCGTTGCATATCATTGTTCCGAGTTGAAGTTCGTGCTTGTACCCGAA

GAGTCGGGAAAGTTATCCCCGACGGAAATAGGATTCCATTTTCAGATTGGGTGCCGCGTA

CATGACAGAAGACCCCGCTCTGAGTCAGTGATGACGCACCAACGCAGATATCGATGTCTC

ACGTCTACCTTATTACTTGCCTTGATCGTCGGCACCCGATATTTGTCAACTGGGAATCCT

CCTGGACCTATTATCGGTCCTTTGAAACTAACAGCAATTACCCCAGAAAGTGTCTGCTAA

>69

ATGCAAATAGACCCCGTTGCATATCATTGTTCCGAGTTGAAGTTCGTGCTTGTACCCGAA

GAGTCGGGAAAGTTATCCCCGACGGAAATAGGATTCCATTTTCAGATTGGGTGCCGCGTA

CATGACAGAAGACCCCGCTCTGAGTCAGTGATGACGCACCAACGCAGATATCGATGTCTC

ACGTCTACCTTATTACTTGCCTTGATCGTCGGCACCCGATATTTGTCAACTGGGAATCCT

CCTGGACCTATTATCGGTCCTTTGAAACTAACAGCAATTACCCCAGAAAGTGTCTGCTAA

>70

ATGCAAATAGACCCCGTTGCATATCATTGTTCCGAGTTGAAGTTCGTGCTTGTACCCGAA

GAGTCGGGAAAGTTATCCCCGACGGAAATAGGATTCCATTTTCAGATTGGGTGCCGCGTA

CATGACAGAAGACCCCGCTCTGAGTCAGTGATGACGCACCAACGCAGATATCGATGTCTC

ACGTCTACCTTATTACTTGCCTTGATCGTCGGCACCCGATATTTGTCAACTGGGAATCCT

CCTGGACCTATTATCGGTCCTTTGAAACTAACAGCAATTACCCCAGAAAGTGTCTGCTAA

>71

ATGCAAATAGACCCCGTTGCATATCATTGTTCCGAGTTGAAGTTCGTGCTTGTACCCGAA

GAGTCGGGAAAGTTATCCCCGACGGAAATAGGATTCCATTTTCAGATTGGGTGCCGCGTA

CATGACAGAAGACCCCGCTCTGAGTCAGTGATGACGCACCAACGCAGATATCGATGTCTC

ACGTCTACCTTATTACTTGCCTTGATCGTCGGCACCCGATATTTGTCAACTGGGAATCCT

CCTGGACCTATTATCGGTCCTTTGAAACTAACAGCAATTACCCCAGAAAGTGTCTGCTAA

>72

ATGCAAATAGACCCCGTTGCATATCATTGTTCCGAGTTGAAGTTCGTGCTTGTACCCGAA

GAGTCGGGAAAGTTATCCCCGACGGAAATAGGATTCCATTTTCAGATTGGGTGCCGCGTA

CATGACAGAAGACCCCGCTCTGAGTCAGTGATGACGCACCAACGCAGATATCGATGTCTC

ACGTCTACCTTATTACTTGCCTTGATCGTCGGCACCCGATATTTGTCAACTGGGAATCCT

CCTGGACCTATTATCGGTCCTTTGAAACTAACAGCAATTACCCCAGAAAGTGTCTGCTAA

>73

ATG-AAATAGACCCCGTTGCATATCATTGTTCCGAGTTGAAGTTCGTGCTTGTACCCGAA

GAGTCGGGAAAGTTATCCCCGACGGAAATAGGATTCCATTTTCAGATTGGGTGCCGCGTA

CATGACAGAAGACCCCGCTCTGAGTCAGTGATGACGCACCAACGCAGATATCGATGTCTC

ACGTCTACCTTATTACTTGCCTTGATCGTCGGCACCCGATATTTGTCAACTGGGAATCCT

CCTGGACCTATTATCGGTCCTTTGAAACTAACAGCAATTACCCCAGAAAGTGTCTGCTAA

>74

ATGCAAATAGACCCCGTTGCATATCATTGTTCCGAGTTGAAGTTCGTGCTTGTACCCGAA

GAGTCGGGAAAGTTATCCCCGACGGAAATAGGATTCCATTTTCAGATTGGGTGCCGCGTA

CATGACAGAAGACCCCGCTCTGAGTCAGTGATGACGCACCAACGCAGATATCGATGTCTC

ACGTCTACCTTATTACTTGCCTTGATCGTCGGCACCCGATATTTGTCAACTGGGAATCCT

CCTGGACCTATTATCGGTCCTTTGAAACTAACAGCAATTACCCCAGAAAGTGTCTGCTAA

>75

ATGCAAATAGACCCCGTTGCATATCATTGTTCCGAGTTGAAGTTCGTGCTTGTACCCGAA

GAGTCGGGAAAGTTATCCCCGACGGAAATAGGATTCCATTTTCAGATTGGGTGCCGCGTA

CATGACAGAAGACCCCGCTCTGAGTCAGTGATGACGCACCAACGCAGATATCGATGTCTC

ACGTCTACCTTATTACTTGCCTTGATCGTCGGCACCCGATATTTGTCAACTGGGAATCCT

CCTGGACCTATTATCGGTCCTTTGAAACTAACAGCAATTACCCCAGAAAGTGTCTGCTAA

>76

ATG-AAATAGACCCCGTTGCATATCATTGTTCCGAGTTGAAGTTCGTGCTTGTACCCGAA

GAGTCGGGAAAGTTATCCCCGACGGAAATAGGATTCCATTTTCAGATTGGGTGCCGCGTA

CATGACAGAAGACCCCGCTCTGAGTCAGTGATGACGCACCAACGCAGATATCGATGTCTC

ACGTCTACCTTATTACTTGCCTTGATCGTCGGCACCCGATATTTGTCAACTGGGAATCCT

CCTGGACCTATTATCGGTCCTTTGAAACTAACAGCAATTACCCCAGAAAGTGTCTGCTAA

>77

ATGCAAATAGACCCCGTTGCATATCATTGTTCCGAGTTGAAGTTCGTGCTTGTACCCGAA

GAGTCGGGAAAGTTATCCCCGACGGAAATAGGATTCCATTTTCAGATTGGGTGCCGCGTA

CATGACAGAAGACCCCGCTCTGAGTCAGTGATGACGCACCAACGCAGATATCGATGTCTC

ACGTCTACCTTATTACTTGCCTTGATCGTCGGCACCCGATATTTGTCAACTGGGAATCCT

CCTGGACCTATTATCGGTCCTTTGAAACTAACAGCAATTACCCCAGAAAGTGTCTGCTAA

>78

ATGCAAATAGACCCCGTTGCATATCATTGTTCCGAGTTGAAGTTCGTGCTTGTACCCGAA

GAGTCGGGAAAGTTATCCCCGACGGAAATAGGATTCCATTTTCAGATTGGGTGCCGCGTA

CATGACAGAAGACCCCGCTCTGAGTCAGTGATGACGCACCAACGCAGATATCGATGTCTC

ACGTCTACCTTATTACTTGCCTTGATCGTCGGCACCCGATATTTGTCAACTGGGAATCCT

CCTGGACCTATTATCGGTCCTTTGAAACTAACAGCAATTACCCCAGAAAGTGTCTGCTAA

>79

ATGCAAATAGACCCCGTTGCATATCATTGTTCCGAGTTGAAGTTCGTGCTTGTACCCGAA
GAGTCGGGAAAGTTATCCCCGACGGAAATAGGATTCCATTTTCAGATTGGGTGCCGCGTA
CATGACAGAAGACCCCGCTCTGAGTCAGTGATGACGCACCAACGCAGATATCGATGTCTC
ACGTCTACCTTATTACTTGCCTTGATCGTCGGCACCCGATATTTGTCAACTGGGAATCCT
CCTGGACCTATTATCGGTCCTTTGAAACTAACAGCAATTACCCCAGAAAGTGTCTGCTAA

>80

ATGCAAATAGACCCCGTTGCATATCATTGTTCCGAGTTGAAGTTCGTGCTTGTACCCGAA
GAGTCGGGAAAGTTATCCCCGACGGAAATAGGATTCCATTTTCAGATTGGGTGCCGCGTA
CATGACAGAAGACCCCGCTCTGAGTCAGTGATGACGCACCAACGCAGATATCGATGTCTC
ACGTCTACCTTATTACTTGCCTTGATCGTCGGCACCCGATATTTGTCAACTGGGAATCCT
CCTGGACCTATTATCGGTCCTTTGAAACTAACAGCAATTACCCCAGAAAGTGTCTGCTAA

>81

ATGCAAATAGACCCCGTTGCATATCATTGTTCCGAGTTGAAGTTCGTGCTTGTACCCGAA
GAGTCGGGAAAGTTATCCCCGACGGAAATAGGATTCCATTTTCAGATTGGGTGCCGCGTA
CATGACAGAAGACCCCGCTCTGAGTCAGTGATGACGCACCAACGCAGATATCGATGTCTC
ACGTCTACCTTATTACTTGCCTTGATCGTCGGCACCCGATATTTGTCAACTGGGAATCCT
CCTGGACCTATTATCGGTCCTTTGAAACTAACAGCAATTACCCCAGAAAGTGTCTGCTAA

>82

ATGCAAATAGACCCCGTTGCATATCATTGTTCCGAGTTGAAGTTCGTGCTTGTACCCGAA
GAGTCGGGAAAGTTATCCCCGACGGAAATAGGATTCCATTTTCAGATTGGGTGCCGCGTA
CATGACAGAAGACCCCGCTCTGAGTCAGTGATGACGCACCAACGCAGATATCGATGTCTC
ACGTCTACCTTATTACTTGCCTTGATCGTCGGCACCCGATATTTGTCAACTGGGAATCCT
CCTGGACCTATTATCGGTCCTTTGAAACTAACAGCAATTACCCCAGAAAGTGTCTGCTAA

>83

ATGCAAATAGACCCCGTTGCATATCATTGTTCCGAGTTGAAGTTCGTGCTTGTACCCGAA

GAGTCGGGAAAGTTATCCCCGACGGAAATAGGATTCCATTTTCAGATTGGGTGCCGCGTA

CATGACAGAAGACCCCGCTCTGAGTCAGTGATGACGCACCAACGCAGATATCGATGTCTC

ACGTCTACCTTATTACTTGCCTTGATCGTCGGCACCCGATATTTGTCAACTGGGAATCCT

CCTGGACCTATTATCGGTCCTTTGAAACTAACAGCAATTACCCCAGAAAGTGTCTGCTAA

>84

ATGCAAATAGACCCCGTTGCATATCATTGTTCCGAGTTGAAGTTCGTGCTTGTACCCGAA

GAGTCGGGAAAGTTATCCCCGACGGAAATAGGATTCCATTTTCAGATTGGGTGCCGCGTA

CATGACAGAAGACCCCGCTCTGAGTCAGTGATGACGCACCAACGCAGATATCGATGTCTC

ACGTCTACCTTATTACTTGCCTTGATCGTCGGCACCCGATATTTGTCAACTGGGAATCCT

CCTGGACCTATTATCGGTCCTTTGAAACTAACAGCAATTACCCCAGAAAGTGTCTGCTAA

>85

ATGCAAATAGACCCCGTTGCATATCATTGTTCCGAGTTGAAGTTCGTGCTTGTACCCGAA

GAGTCGGGAAAGTTATCCCCGACGGAAATAGGATTCCATTTTCAGATTGGGTGCCGCGTA

CATGACAGAAGACCCCGCTCTGAGTCAGTGATGACGCACCAACGCAGATATCGATGTCTC

ACGTCTACCTTATTACTTGCCTTGATCGTCGGCACCCGATATTTGTCAACTGGGAATCCT

CCTGGACCTATTATCGGTCCTTTGAAACTAACAGCAATTACCCCAGAAAGTGTCTGCTAA

>86

ATGCAAATAGACCCCGTTGCATATCATTGTTCCGAGTTGAAGTTCGTGCTTGTACCCGAA

GAGTCGGGAAAGTTATCCCCGACGGAAATAGGATTCCATTTTCAGATTGGGTGCCGCGTA

CATGACAGAAGACCCCGCTCTGAGTCAGTGATGACGCACCAACGCAGATATCGATGTCTC

ACGTCTACCTTATTACTTGCCTTGATCGTCGGCACCCGATATTTGTCAACTGGGAATCCT

CCTGGACCTATTATCGGTCCTTTGAAACTAACAGCAATTACCCCAGAAAGTGTCTGCTAA

>87

ATG-AAATAGACCCCGTTGCATATCATTGTTCCGAGTTGAAGTTCGTGCTTGTACCCGAA

GAGTCGGGAAAGTTATCCCCGACGGAAATAGGATTCCATTTTCAGATTGGGTGCCGCGTA

CATGACAGAAGACCCCGCTCTGAGTCAGTGATGACGCACCAACGCAGATATCGATGTCTC

ACGTCTACCTTATTACTTGCCTTGATCGTCGGCACCCGATATTTGTCAACTGGGAATCCT

CCTGGACCTATTATCGGTCCTTTGAAACTAACAGCAATTACCCCAGAAAGTGTCTGCTAA

>88

ATGCAAATAGACCCCGTTGCATATCATTGTTCCGAGTTGAAGTTCGTGCTTGTACCCGAA

GAGTCGGGAAAGTTATCCCCGACGGAAATAGGATTCCATTTTCAGATTGGGTGCCGCGTA

CATGACAGAAGACCCCGCTCTGAGTCAGTGATGACGCACCAACGCAGATATCGATGTCTC

ACGTCTACCTTATTACTTGCCTTGATCGTCGGCACCCGATATTTGTCAACTGGGAATCCT

CCTGGACCTATTATCGGTCCTTTGAAACTAACAGCAATTACCCCAGAAAGTGTCTGCTAA

>89

ATGCAAATAGACCCCGTTGCATATCATTGTTCCGAGTTGAAGTTCGTGCTTGTACCCGAA

GAGTCGGGAAAGTTATCCCCGACGGAAATAGGATTCCATTTTCAGATTGGGTGCCGCGTA

CATGACAGAAGACCCCGCTCTGAGTCAGTGATGACGCACCAACGCAGATATCGATGTCTC

ACGTCTACCTTATTACTTGCCTTGATCGTCGGCACCCGATATTTGTCAACTGGGAATCCT

CCTGGACCTATTATCGGTCCTTTGAAACTAACAGCAATTACCCCAGAAAGTGTCTGCTAA

>90

ATGCAAATAGACCCCGTTGCATATCATTGTTCCGAGTTGAAGTTCGTGCTTGTACCCGAA

GAGTCGGGAAAGTTATCCCCGACGGAAATAGGATTCCATTTTCAGATTGGGTGCCGCGTA

CATGACAGAAGACCCCGCTCTGAGTCAGTGATGACGCACCAACGCAGATATCGATGTCTC

ACGTCTACCTTATTACTTGCCTTGATCGTCGGCACCCGATATTTGTCAACTGGGAATCCT

CCTGGACCTATTATCGGTCCTTTGAAACTAACAGCAATTACCCCAGAAAGTGTCTGCTAA

>91

ATGCAAATAGACCCCGTTGCATATCATTGTTCCGAGTTGAAGTTCGTGCTTGTACCCGAA

GAGTCGGGAAAGTTATCCCCGACGGAAATAGGATTCCATTTTCAGATTGGGTGCCGCGTA

CATGACAGAAGACCCCGCTCTGAGTCAGTGATGACGCACCAACGCAGATATCGATGTCTC

ACGTCTACCTTATTACTTGCCTTGATCGTCGGCACCCGATATTTGTCAACTGGGAATCCT

CCTGGACCTATTATCGGTCCTTTGAAACTAACAGCAATTACCCCAGAAAGTGTCTGCTAA

>92

ATGCAAATAGACCCCGTTGCATATCATTGTTCCGAGTTGAAGTTCGTGCTTGTACCCGAA

GAGTCGGGAAAGTTATCCCCGACGGAAATAGGATTCCATTTTCAGATTGGGTGCCGCGTA

CATGACAGAAGACCCCGCTCTGAGTCAGTGATGACGCACCAACGCAGATATCGATGTCTC

ACGTCTACCTTATTACTTGCCTTGATCGTCGGCACCCGATATTTGTCAACTGGGAATCCT

CCTGGACCTATTATCGGTCCTTTGAAACTAACAGCAATTACCCCAGAAAGTGTCTGCTAA

>93

ATGCAAATAGACCCCGTTGCATATCATTGTTCCGAGTTGAAGTTCGTGCTTGTACCCGAA

GAGTCGGGAAAGTTATCCCCGACGGAAATAGGATTCCATTTTCAGATTGGGTGCCGCGTA

CATGACAGAAGACCCCGCTCTGAGTCAGTGATGACGCACCAACGCAGATATCGATGTCTC

ACGTCTACCTTATTACTTGCCTTGATCGTCGGCACCCGATATTTGTCAACTGGGAATCCT

CCTGGACCTATTATCGGTCCTTTGAAACTAACAGCAATTACCCCAGAAAGTGTCTGCTAA

>94

ATGCAAATAGACCCCGTTGCATATCATTGTTCCGAGTTGAAGTTCGTGCTTGTACCCGAA

GAGTCGGGAAAGTTATCCCCGACGGAAATAGGATTCCATTTTCAGATTGGGTGCCGCGTA

CATGACAGAAGACCCCGCTCTGAGTCAGTGATGACGCACCAACGCAGATATCGATGTCTC

ACGTCTACCTTATTACTTGCCTTGATCGTCGGCACCCGATATTTGTCAACTGGGAATCCT

CCTGGACCTATTATCGGTCCTTTGAAACTAACAGCAATTACCCCAGAAAGTGTCTGCTAA

>95

ATGCAAATAGACCCCGTTGCATATCATTGTTCCGAGTTGAAGTTCGTGCTTGTACCCGAA

GAGTCGGGAAAGTTATCCCCGACGGAAATAGGATTCCATTTTCAGATTGGGTGCCGCGTA

CATGACAGAAGACCCCGCTCTGAGTCAGTGATGACGCACCAACGCAGATATCGATGTCTC

ACGTCTACCTTATTACTTGCCTTGATCGTCGGCACCCGATATTTGTCAACTGGGAATCCT

CCTGGACCTATTATCGGTCCTTTGAAACTAACAGCAATTACCCCAGAAAGTGTCTGCTAA

>96

ATGCAAATAGACCCCGTTGCATATCATTGTTCCGAGTTGAAGTTCGTGCTTGTACCCGAA

GAGTCGGGAAAGTTATCCCCGACGGAAATAGGATTCCATTTTCAGATTGGGTGCCGCGTA

CATGACAGAAGACCCCGCTCTGAGTCAGTGATGACGCACCAACGCAGATATCGATGTCTC

ACGTCTACCTTATTACTTGCCTTGATCGTCGGCACCCGATATTTGTCAACTGGGAATCCT

CCTGGACCTATTATCGGTCCTTTGAAACTAACAGCAATTACCCCAGAAAGTGTCTGCTAA

>97

ATGCAAATAGACCCCGTTGCATATCATTGTTCCGAGTTGAAGTTCGTGCTTGTACCCGAA

GAGTCGGGAAAGTTATCCCCGACGGAAATAGGATTCCATTTTCAGATTGGGTGCCGCGTA

CATGACAGAAGACCCCGCTCTGAGTCAGTGATGACGCACCAACGCAGATATCGATGTCTC

ACGTCTACCTTATTACTTGCCTTGATCGTCGGCACCCG-TATTTGTCAACTGGGAATCCT

CCTGGACCTATTATCGGTCCTTTGAAACTAACAGCAATTACCCCAGAAAGTGTCTGCTAA

>98

ATGCAAATAGACCCCGTTGCATATCATTGTTCCGAGTTGAAGTTCGTGCTTGTACCCGAA

GAGTCGGGAAAGTTATCCCCGACGGAAATAGGATTCCATTTTCAGATTGGGTGCCGCGTA

CATGACAGAAGACCCCGCTCTGAGTCAGTGATGACGCACCAACGCAGATATCGATGTCTC

ACGTCTACCTTATTACTTGCCTTGATCGTCGGCACCCGATATTTGTCAACTGGGAATCCT

CCTGGACCTATTATCGGTCCTTTGAAACTAACAGCAATTACCCCAGAAAGTGTCTGCTAA

>99

ATGCAAATAGACCCCGTTGCATATCATTGTTCCGAGTTGAAGTTCGTGCTTGTACCCGAA

GAGTCGGGAAAGTTATCCCCGACGGAAATAGGATTCCATTTTCAGATTGGGTGCCGCGTA

CATGACAGAAGACCCCGCTCTGAGTCAGTGATGACGCACCAACGCAGATATCGATGTCTC

ACGTCTACCTTATTACTTGCCTTGATCGTCGGCACCCGATATTTGTCAACTGGGAATCCT

CCTGGACCTATTATCGGTCCTTTGAAACTAACAGCAATTACCCCAGAAAGTGTCTGCTAA
