# Supplementary material for: GeneScanner: profiling genetic variation across bacterial populations
Source: Microb Genom. 2026 Jun 2;12(6):001714. doi: 10.1099/mgen.0.001714 (PMC13229410; doi:10.1099/mgen.0.001714)
Supplement: Supplementary Material 6. [file mgen-12-01714-s006.pdf]

>ref

ATGCAAATAGACCCCGTTGCATATCATTGTTCCGAGTTGAAGTTCGTGCTTGTACCCGAAGAGTCGGGAAAGTTAT
CCCCGACGGAAATAGGATTCCATTTTCAGATTGGGTGCCGCGTACATGACAGAAGACCCCGCTCTGAGTCAGTGA
TGACGCACCAACGCAGATATCGATGTCTCACGTCTACCTTATTACTTGCCTTGATCGTCGGCACCCGATATTTGTCA
ACTGGGAATCCTCCTGGACCTATTATCGGTCCTTTGAAACTAACAGCAATTACCCCAGAAAGTGTCTGCTAA

>p1_ind1994

ATGCAAATAGACCCCGTTGCATATCATTGTTCCGAGTTGAAGTTCGTGCTTGTACCCGAAGAGTCGGGAAAGTTAT
CCCCGACGGAAATAGGATTCCATTTTCAGATTGGGTGCCGCGTACATGACAGAAGACCCCGCTCTGAGTCAGTGA
TGACGCACCAACGCAGATATCGATGTCTCACGTCTACCTTATTACTTGCCTTGATCGTCGGCACCCGATATTTGTCA
ACTGGGAATCCTCCTGGACCTATTATCGGTCCTTTGAAACTAACAGCAATTACCCCAGAAAGTGTCTGCTAA

>p1_ind2665

ATGCAAATAGACCCCGTTGCATATCATTGTTCCGAGTTGAAGTTCGTGCTTGTACCCGAAGAGTCGGGAAAGTTAT
CCCCGACGGAAATAGGATTCCATTTTCAGATTGGGTGCCGCGTACATGACAGAAGACCCCGCTCTGAGTCAGTGA
TGACGCACCAACGCAGATATCGATGTCTCACGTCTACCTTATTACTTGCCTTGATCGTCGGCACCCGATATTTGTCA
ACTGGGAATCCTCCTGGACCTATTATCGGTCCTTTGAAACTAACAGCAATTACCCCAGAAAGTGTCTGCTAA

>p1_ind3972

ATGCAAATAGACCCCGTTGCATATCATTGTTCCGAGTTGAAGTTCGTGCTTGTACCCGAAGAGTCGGGAAAGTTAT
CCCCGACGGAAATAGGATTCCATTTTCAGATTGGGTGCCGCGTACATGACAGAAGACCCCGCTCTGAGTCAGTGA
TGACGCACCAACGCAGATATCGATGTCTCACGTCTACCTTATTACTTGCCTTGATCGTCGGCACCCGATATTTGTCA
ACTGGGAATCCTCCTGGACCTATTATCGGTCCTTTGAAACTAACGGCAATTACCCCAGAAAGTGTCTGCTAA

>p1_ind931

ATGCAAATAGACCCCGTTGCATATCATTGTTCCGAGTAGAAGTTCGTGCTTGTACCCGAAGAGTCGGGAAAGTTAT
CCCCGACGGAAATAGGATTCCATTTTCAGACTGGGCGCCGCGTACATGACAGAAGACCCCGCTCTGAGTCAGTGA

TGACGCACCAACGCAGATATCGATGTCTCACGTCTACCTTATTACTTGCCTTGATCGTCGGCACCCGATATTTGTCA
ACTGGGAATCCTCCTGGACCTATTATCGGTCCTTTGAAACTAACAGCAATTACCCCAGAAAGTGTCTGCTAA

>p1_ind4211

ATGCAAATAGACCCCGTTGCATATCATTGTTCCGAGTTGAAGTTCGTGCTTGTACCCGAAGAGTCGGGAAAGTTAT
CCCCGACGGAAATAGGATTCCATTTTCAGATTGGGCGCCGCGTACATGACAGAAGACCCCGCTCTGAGTCAGTGA
TGACGCACCAACGCAGATATCGATGTCTCACGTCTACCTTATTACTTGCCTTGATCGTCGGCACCCGATATTTGTCA
ACTGGGAATCCTCCTGGACCTATTATCGGTCCTTTGAAACTAACAGCAATTACCCCAGAAAGTGTCTGCTAA

>p1_ind4169

ATGCAAATAGACCCCGTTGCATATCATTGTTCCGAGTTGAAGTTCGTGCTTGTACCCGAAGAGTCGGGAAAGTTAT
CCCCGACGGAAATAGGATTCCATTTTGAGATTGGGTGCCGCGTACATGACAGAAGACCCCGCTCTGAGTCAGTGA
TGGCGCACCAACGCAAATATCGATGTCTCACGTCTACCTTATTACTTGCCTTGATCGTCGGCACCCGATATTTGTCA
ACTGGGAATCCTCCTGGACCTATTATCGGTCCTTTGAAACTAACAGCAATTACGCCAGAAAGTGTCTGCTAA

>p1_ind1458

ATGCAAATAGACCCCGTTGCATATCATTGTTCCGAGTTGAAGTTCGTGCTTGTACCCGAAGAGTCGGGAAAGTTAT
CCCCGACGGAAATAGGATTCCATTTTCAGATTGGACGCCGCGTACATGACAGAAGACCCCGCTCTGAGTCAGTGA
TGACGCACCAACGCAGATATCGATGTCTCACGTCTACCTTATTACTTGCCTTGATCGTCGGCACCCGATATTTGTCA
ACTGGGAATCCTCCTGGACCTATTATCGGTCCTTTGAAACTAACAGCAATTACCCCAGAAAGTGTCTGCTAA

>p1_ind1648

ATGCAAATAGACCCCGTTGCATATCATTGTTCCGAGTTGAAGTTCGTGCTTGTACCCGAAGAGTCGGGAAAGTTAT
CCCCGACGGAAATAGGATTCCATTTTCAGACTGGGCGCCGCGTACATGACAGAAGACCCCGCTCTGAGTCAGTGA
TGACGCACCAACGCAGATATCGATGTCTCACGTCTACCTTATTACTTGCCTTGATCGTCGGCACCCGACATTTGTCA
ACTGGGAATCCTCCTGGACCTATTATCGGTCCTTTGAAACTAACAGCAATTACCCCAGAAAGTGTCTGCTAA

>p1_ind1555

ATGCAAATAGACCCCGTTGCATATCATTGTTCCGAGTTGAAGTTCGTGCTTGTACCCGAAGAGTCGGGAAAGTTAT
CCCCGACGGAAATAGGATTCCATTTTCAGACTGGGCGCCGCGTACATGACAGAAGACCCCGCTCTGAGTCAGTGA
TGGCGCACCAACGCAAATATCGATGTCTCACGTCTACCTTATTACTTGCCTTGATCGTCGGCACCCGATATTTGTCA
ACTGGGAATCCTCCTGGACCTATTATCGGTCCTTTGAAACTAACAGCAATTACACCAGAAAGTGTCTGCTAA

>p1_ind1751

ATGCAAATAGACCCCGTTGCATATCATTGTTCCGAGTTGAAGTTCGTGCTTGTACCCGAAGAGTCGGGAAAGTTAT
CCCCGACGGAAATAGGATTCCATTTTCAGATTGGGTGCCGCGTACATGACAGAAGACCCCGCTCTGAGTCAGTGA
TGGCGCACCAACGCAAATATCGATGTCTCACGTCTACCTTATTACTTGCCTTGATCGTCGGCACCCGATATTTGTCA
ACTGGGAATCCTCCTGGACCTATTATCGGTCCTTTGAAACTAACAGCAATTACCCCAGAAAGTGTCTGCTAA

>p1_ind5163

ATGCAAATAGACCCCGTTGCATATCATTGTTCCGAGTTGAAGTTCGTGCTTGTACCCGAAGAGTCGGGAAAGTTAT
CCCCGACGGAAATAGGATTCCATTTTCAGACTGGGCGCCGCGTACATGACAGAAGACCCCGCTCTGAGTCAGTGA
TGACGCACCAACGCAGATATCGATGTCTCACGTCTACCTTATTACTTGCCTTGATCGTCGGCACCCGATATTTGTCA
ACTGGGAATCCTCCTGGACCTATTATCGGTCCTTTGAAACTAACAGCAATTACCCCAGAAAGTGTCTGCTAA

>p1_ind5131

ATGCAAATAGACCCCGTTGCATATCATTGTTCCGAGTTGAAGTTCGTGCTTGTACCCGAAGAGTCGGGAAAGTTAT
CCCCGACGGAAATAGGATTCCATTTTCAGATTGGGTGCCGCGTACATGACAGAAGACCCCGCTCTGAGTCAGTGA
TGGCGCACCAACGCAAATATCGATGTCTCACGTCTACCTTATTACTTGCCTTGATCGTCGGCACCCGATATTTGTCA
ACTGGGAATCCTCCTGGACCTATTATCGGTCCTTTGAAACTAACAGCAATTACCCCAGAAAGTGTCTGCTAA

>p1_ind787

ATGCAAATAGACCCCGTTGCATATCATTGTTCCGAGTTGAAGTTCGTGCTTGTACCCGAAGAGTCGGGAAAGTTAT
CCCCGACGGAAATAGGATTCCATTTTCAGATTGGGTGCCGCGTACATGACAGAAGACCCCGCTCTGAGTCAGTGA
TGACGCACCAACGCAGATATCGATGTCTCACGTCTACCTTATTACTTGCCTTGATCGTCGGCACCCGATATTTGTCA
ACTGGGAATCCTCCTGGACCTATTATCGGTCCTTTGAAACTAACGGCAATTACCCCAGAAAGTGTCTGCTAA

>p1_ind2611

ATGCAAATAGACCCCGTTGCATATCATTGTTCCGAGTTGAAGTTCGTGCTTGTACCCGAAGAGTCGGGAAAGTTAT
CCCCGACGGAAATAGGATTCCATTTTCAGATTGGGCGCCGCGTACATGACAGAAGACCCCGCTCTGAGTCAGTGA
TGGCGCACCAACGTAGATATCGATGTCTCACGTCTACCTTATTACTTGCCTTGATCGTCGGCACCCGATATTTGTCA
ACTGGGAATCCTCGTGGACCTATTATCGGTCCTTTGAAACTAACAGCAATTACCCCAGAAAGTGTCTGCTAA

>p1_ind5087

ATGAAAATAGACCCTGTTGCATATCATTGTTCCGAGTTGAAGTTCGTGCTTGTACCCGAAGAGTCGGGAAAGTTAT
CCCCGACGGAAATAGGATTCCATTTTCAGATTGGGTGCCGCGTACATGACAGAAGACCCCGTTCTGAGTCAGTGAT
GTCGCACCAACGCAGATATCGATGTCTCACGTCTACCTTATTACTTGCCTTGATCGTCGGCACCCGATATTTGTCAA
CTGGGAATCCTCCTGGACCTATTATCGGTCCTTTGAAACTAACAGCAATTACCCCAGAAAGTGTCTGCTAA

>p1_ind2287

ATGCAAATAGACCCCGTTGCATATCATTGTTCCGAGTTGAAGTTCGTGCTTGTACCCGAAGAGTCGGGAAAGTTAT
CCCCGACGGAAATAGGATTCCATTTTCAGATTGGGTGCCGCGTACATGACAGAAGACCCCGCTCTGAGTCAGTTAT
GACGCACCAACGCAGATATCGATGTCTCACGTCTACCTTATTACTTGCCTTGATCGTCGGCACCCGATATTTGTCAA
CTGGGAATCCTCCTGGACCTATTATCGGTCCTTTGAAACTAACAGCAATTACCCCAGAAAGTGTCTGCTAA

>p1_ind2428

ATGCAAATAGACCCCGTTGCATATCATTGTTCCGAGTTGAAGTTCGTGCTTGTACCCGAAGAGTCGGGAAAGTTAT
CCCCGACGGAAATAGGATTCCATTTTCAGACTGGGCGCCGCGTACATGACAGAAGACCCCGCTCTGAGTCAGTGA

TGACGCACCAACGCAGATATCGATGTCTCACGTCTACCTAATTACTTGCCTTGATCGTCGGCACCCGATATTTGTCA
ACTGGGAATCCTCCTGGACCTATTATCGGTCCTTTGAAACTAACAGCAATTACCCCAGAAAGTGTCTGCTAA

>p1_ind3761

ATGCAAATAGACCCCGTTGCATATCATTGTTCCGAGTTGAAGTTCGTGCTTGTACCCGAAGAGTCGGGAAAGTTAT
CCCCGACGGAAATAGGATTCCATTTTCAGACTGGGCGCCGCGTACATGACAGAAGACCCCGCTCTGAGTCAGTGA
TGACGCACCAACGCAGATATCGATGTCTCACGTCTACCTTATTACTTGCCTTGATCGTCGGCACCCGATATTTGTCA
ACTGGGAATCCTCCTGGACCTATTATCGGTCCTTTGAAACTAACAGCAATTACCCCAGAAAGTGTCTGCTAA

>p1_ind4030

ATGCAAATAGACCCCGTTGCATATCATTGTTCCGAGTTGAAGTTCGTGCTTGTACCCGAAGAGTCGGGAAAGTTAT
CCCCGACGGAAATAGGATTCCATTTTCAGATTGGGTGCCGCGTACATGACAGAAGACCCCGCTCTGAGTCAGTGA
TGACGCACCAACGCAGATATCGATGTCTCACGTCTACCTTATTACTTGCCTTGATCGTCGGCACCCGATATTTGTCA
ACTGGGAATCCTCCTGGACCTATTATCGGTCCTTTGAAACTAACAGCAATTACCCCAGAAAGTGTCTGCTAA

>p1_ind4959

ATGCAAATAGACCCCGTTGCATATCATTGTTCCGAGTTGAAGTTCGTGCTTGTACCCGAAGAGTCGGGAAAGTTAT
CCCCGACGGAAATAGGATTCCATTTTCAGATTGGGTGCCGCGTACATGACAGAAGACCCCGCTCTGAGTCAGTGA
TGACGCACCAACGCAGATATCGATGTCTCACGTCTACCTTATTACTTGCCTTGATCGTCGGCACCCGATATTTGTCA
ACTGGGAATCCTCCTGGACCTATTATCGGTCCTTTGAAACTAACAGCAATTACCCCAGAAAGTGTCTGCTAA

>p1_ind4815

ATGCAAATAGACCCCGTTGCATATCATTGTTCCGAGTTGAAGTTCGTGCTTGTACCCGAAGAGTCGGGAAAGTTAT
CCCCGACGGAAATAGGATTCCATTTTCAGATTGGGTGCCGCGTACATGACAGAAGACCCCGCTCTGAGTCAGTGA
TGACGCACCAACGCAGATATCGATGTCTCACGTCTACCTTATTACTTGCCTTGATCGTCGGCACCCGATATTTGTCA
ACTGGGAATCCTCCTGGACCTATTATCGGTCCTTTGAAACTAACAGCAATTACCCCAGAAAGTGTCTGCTAA

>p1_ind125

ATGCAAATAGACCCCGTTGCATATCATTGTTCCGAGTTGAAGTTCGTGCTTGTACCCGAAGAGTCGGGAAAGTTAT
CCCCGACGGAAATAGGATTACATTTTCAGATTGGGCGCCGCGTACATGACAGAAGACCCCGCTCTGAGTCAGTGA
TGACGCACCAACGCAGATATCGATGTCTCACGTCTACCTTATTACTTGCCTTGATCGTCGGCACCCGATATTTGTCA
ACTGGGCATCCTCCTGGACCTATTATCGGTCCTTTGAAACTAACAGCAATTACCCCAGAAAGTGTCTGCTAA

>p1_ind2824

ATGCAAATAGACCCCGTTGCATATCATTGTTCCGAGTTGAAGTTCGTGCTTGTACCCGAAGAGTCGGGAAAGTTAT
CCCCGACGGAAATAGGATTCCATTTTCAGATTGGGTGCCGCGTACATGACAGCAGACCCCGCTCTGAGTCAGTGAT
GGCGCACCAACGCAAATATCGATGTCTCACGTCTACCTTATTACTTGCCTTGATCGTCGGCACCCGATTTTTGTCAA
CTGGGAATCCTCCTGGACCTATTATCGGTCCTTTGAAACTAACAGCAATTACCCCAGAAAGTGTCTGCTAA

>p1_ind4843

ATGCAAATAGACCCCGTTGCATATCATTGTTCCGAGTTGAAGTTCGTGCTTGTACCCGAAGAGTCGGGAAAGTTAT
CCCCGACGGAAATAGGATTCCATTTTCAGATTGGGTGCCGCGTACATGACAGAAGACCCCGCTCTGTGTCAGTGAT
GACGCACCTACGCAGATATCGATGTCTCACGTCTACCTTATTACTTGCCTTGATCGTCGGCACCCGATATTTGTCAA
CTGGGAATCCTCCTGGACCTATTATCGGTCCTTTGAAACTAACAGCAATTACCCCAGAAAGTGTCTGCTAA

>p1_ind337

ATGCAAATAGACCCCGTTGCATATCATTGATCCGAGTTGAAGTTCGTGCTTGTACCCGAAGAGTCGGGAAAGTTAT
CCCCGACGGAAATAGGATTCCATTTTCAGATTGGGTGCCGCGTACATGACAGAAGACCCCGCTCTGAGTCTGTGAT
GACGCACCAACGCAGATATCGATGTCTCACGTCTACCTTATTACTTGCCTTGATCGTCGGCACCCGATATTTGTCAA
CTGGGAATCCTCCTGGACCTATTATCGGTCCTTTGAAACTAACAGCAATTACCCCAGAAAGTGTCTGCTAA

>p1_ind5056

ATGCAAATAGACCCCGTTGCATATCATTGTTCCGAGTTGAAGTTCGTGCTTGTACCCGAAGAGTCGGGAAAGTTAT
CCCCGACGGAAATAGGATTTCATTTTCAGACTGGGCGCCGCGTACATGACAGAAGACCCCGCTCTGAGTCAGTGA
TGACGCACCAACGCAGATATCGATGTCTCACGTCTACCTAATTACTTGCCTTGATCGTCGGCACCCGATATTTGTCA
ACTGGGAATCCTCCTGGACCTATTATCGGTCCTTTGAAACTAACAGCAATTACCCCAGAAAGTGTCTGCTAA

>p1_ind766

ATGCAAATAGACCCCGTTGCATATCATTGTTCCGAGTTGAAGTTCGTGCTTGTACCCGAAGAGTCGGGAAAGTTAT
CCCCGACGGAAATAGGATTCCATTTTCAGATTGGGTGCCGCGTACATGACAGAAGACCCCGCTCTGAGTCAGTGA
TGACGCACCAACGCAGATATCGATGTCTCACGTCTACCTTATTACTTGCCTTGATCGTCGGCACCCGATATTTGTCA
ACTGGGAATCCTCCTGGACCTATTATCGGTCCTTTGAAACTAACAGCAATTACCCCAGAAAGTGTCTGCTAA

>p1_ind1362

ATGCAAATAGACCCCGTTGCATATCATTGTTCCGAGTTGAAGTTCGTGCTTGTACCCGAAGAGTCGGGAAAGTTAT
CCCCGACGGAAATAGGATTCCATTTTCAGATTGGGTGCCGCGTACATGACAGAAGACCCCGCTCTGAGTCAGTGA
TGGCGCACCAACGCAAATATCGATGTCTCACGTCTACCTTATTACTTGCCTTGATCGTCGGCACCCGATATTTGTCA
ACTGGGAATCCTCCTGGACCTATTATCGGTCCTTTGAAACTAACAGCAATTACCCCAGAAAGTGTCTGCTAA

>p1_ind3345

ATGCAAATAGACCCCGTTGCATATCATTGTTCCGAGTTGAAGTTCGTGCTTGTACCCGAAGAGTCGGGAAAGTTAT
CCCCGACGGAAATAGGATTCCATTTTCAGATTGGGTGCCGCGTACATGACAGAAGACCCCGCTCTGAGTCAGTGA
TGACGCACCAACGCAGATATCGATGTCTCACGTCTACCTTATTACTTGCCTTGATCGTCGGCACCCGATATTTGTCA
ACTGGGAATCCTCCTGGACCTATTATCGGTCCTTTGAAACTAACAGCAATTACCCCAGAAAGTGTCTGCTAA

>p1_ind2058

ATGCAAATAGACCCCGTTGCATATCATTGTTCCGAGTTGAAGTTCGTGCTTGTACCCGAAGAGTCGGGAAAGTTAT
CCCCGACGGAAATAGGATTCCATTTTCAGATTGGGTGCCGCGTACATGACAGAAGACCCCGTTCTGAGTCAGTGAT

GTCGCACCAACGCAGATATCGATGTCTCACGTCTACCTTATTACTTGCCTTGATCGTCGGCACCCGATATTTGTCAA
CTGGGAATCCTCCTGGACCTATTATCGGTCCTTTGAAACTAACAGCAATTACCCCAGAAAGTGTCTGCTAA

>p1_ind848

ATGCAAATAGACCCCGTTGCATATCATTGTTCCGAGTTGAAGTTCGTGCTTGTACCCGAAGAGTCGGGAAAGTTAT
CCCCGACGGAAATAGGATTCCATTTTCAGATTGGGTGCCGCGTACATGACAGAAGACCCCGCTCTGAGTCAGTGA
TGACGCACCAACGCAGATATCGATGTCTCACGTCTACCTTATTACTTGCCTTGATCGTCGGCACCCGATATTTGTCA
ACTGGGAATCCTCCTGGACCTATTATCGGTCCTTTGAAACTAACAGGAATTACCCCAGAAAGTGTCTGCTAA

>p1_ind4564

ATGCAAATAGACCCCGTTGCATATCATTGTTCCGAGTTGAAGTTCGTGCTTGTACCCGCAGAGTCGGGAAAGTTAT
CCCCGACGGAAATAGGATTCCATTTTCAGATTGGGTGCCGCGTACATGACAGAAGACCCCGCTCTGAGTCAGTGA
TGACGCACCAACGCAGATATCGATGTCTCACGTCTACCTTATTACTTGCCTTGATCGTCGGCACCCGATATTTGTCA
ACTGGGAATCCTCCTGGACCTATTATCGGTCCTTTGAAACTAACAGCAATTACCCCAGAAAGTGTCTGCTAA

>p1_ind1338

AAGCAAATAGACCCCGTTGCATATCATTGTTCCGAGTTGAAGTTCGTGCTTGTACCCGAAGAGTCGGGAAAGTTAT
CCCCGACGGAAATAGGATTCCATTTTCAGACTGGGCGCCGCGTACATGACAGCAGACCCCGCTCTGAGTCAGTGA
TGACGCACCAACGCAGATATCGATGTCTCACGTCTACCTTATTACTTGCCTTGATCGTCGGCACCCGATATTTGTCA
ACTGGGAATCCTCCTGGACCTATTATCGGTCCTTTGAAACTAACAGCAATTACCCCAGAAAGTGTCTGCTAA

>p1_ind502

ATGCAAATAGACCCCGTTGCATATCATTGTTCCGAGTTGAAGTTCGTGCTTGTACCCGAAGAGTCGGGAAAGTTAT
CCCCGACGGAAATAGGATTCCATTTTCAGACTGGGCGCCGCGTACATGACAGAAGACCCCGCTCTGAGTCAGTGA
TGACGCACCAACGCAGATATCGATGTCTCACGTCTACCTTATTACTTGCCTTGATCGTCGGCACCCGATATTTGTCA
ACTGGGAATCCTCCTGGACCTATTATCGGTCCTTTGAAACTAACAGCAATTACCCCAGAAAGTGTCTGCTAA

>p1_ind910

ATGCAAATAGACCCCGTTGCATATCATTGTTCCGAGTTGAAGTTCGTGCTTGTACCCGAAGAGTCGGGAAAGTTAT
CCCCGACGGAAATAGGATTCCATTTTCAGATTGGACGCCGCGTACATGACAGAAGACCCCGCTCTGAGTCAGTGA
TGACGCACCAACGCAGATATCGATGTCTCACGTCTACCTTATTACTTGCCTTGATCGTCGGCACCCGATATTTGTCA
ACTGGGAATCCTCCTGGACCTATTATCGGTCCTTTGAAACTAACAGCAATTACCCCAGAAAGTGTCTGCTAA

>p1_ind1847

ATGCAAATAGACCCCGTTGCATATCATTGTTCCGAGTTGAAGTTCGTGCTTGTACCCGAAGAGTCGGGAAAGTTAT
CCCCGACGGAAATAGGATTCCATTTTCAGATTGGGCGCCGCGTACATGACAGAAGACCCCGCTCTGAGTCAGTGA
TGACGCACCAACGCAGATATCGATGTCTCACGTCTACCTTATTACTTGCCTTGATCGTCGGCACCCGATATTTGTCA
ACTGGGAATCCTCCTGGACCTATTATCGGTCCTTTGAAACTAACAGCAATTACCCCAGAAAGTGTCTGCTAA

>p1_ind3020

ATGCAAATAGACCCCGTTGCATATCATTGTTCCGAGTTGAAGTTCGTGCTTGTACCCGAAGAGTCGGGAAAGTTAT
CCCCGACGGAAATAGGATTCCATTTTCAGATTGGGTGCCGCGTACATGACAGAAGACCCCGCTCTGAGTCAGTGA
TGACGCACCAACGCAGATATCGATGTCTCACGTCTACCTTATTACTTGCCTTGATCGTCGGCACCCGATATTTGTCA
ACTGGGAATCCTCCTGGACCTATTATCGGTCCTTTGAAACTAACAGCAATTACCCCAGAAAGTGTCTGCTAA

>p1_ind2195

ATGCAAATAGACCCCGTTGCATATCATTGTTCCGAGTTGAAGTTCGTGCTTGTACCCGAAGAGTCGGGAAAGTTAT
CCCCGACGGAAATAGGATTCCATTTTCAGATTGGGTGCCGCGTACATGACAGAAGACCCCGCTCTGAGTCAGTGA
TGACGCACCAACGCAGATATCGATGTCTCACGTCTACCTTATTACTTGCCTTGATCGTCGGCACCCGATATTTGTCA
ACTGGGAATCCTCCTGGACCTATTATCGGTCCTTTGAAACTAACAGGAATTACCCCAGAAAGTGTCTGCTAA

>p1_ind3201

ATGCAAATAGACCCCGTTGCATATCATTGTTCCGAGTTGAAGTTCGTGCTTGTACCCGAAGAGTCGGGAAAGTTAT
CCCCGACGGAAATAGGATTCCATTTTCAGATTGGGTGCCGCGCACATGACAGAAGACCCCGCTCTGAGTCAGTGA
TGACGCACCAACGCAGATATCGATGTCTCACGTCTACCTTATTACTTGCCTTGATCGTCGGCACCCGATATTTGTCA
ACTGGGAATCCCCCTGGACCTATTATCGGTCCTTTGAAACTAACAGCAATTACCCCAGAAAGTGTCTGCTAA

>p1_ind531

ATGCAAATAGACCCTGTTGCATATCATTGTTCCGAGTTGAAGTTCGTGCTTGTACCCGAAGAGTCGGGAAAGTTAT
CCCCGACGGAAATAGGATTCCATTTTCAGATTGGGTGCCGCGTACATGACAGAAGACCCCGCTCTGAGTCAGTGA
TGGCGCACCAACGCAAATATCGATGTCTCACGTCTACCTTATTACTTGCCTTGATCGTCGGCACCCGATATTTGTCA
ACTGGGAATCCTCCTGGACCTATTATCGGTCCTTTGAAACTAACAGCAATTACCCCAGAAAGTGTCTGCTAA

>p1_ind2763

ATGCAAATAGACCCCGTTGCATATCATTGTTCCGAGTTGAAGTTCGTGCTTGTACCCGAAGAGTCGGGAAAGTTAT
CCCCGACGGAAATAGGATTCCATTTTCAGATTGGGCGCCGCGTACATGACAGAAGACCCCGCTCTGAGTCAGTGA
TGGCGCACCAACGTAGATATCGATGTCTCACGTCTACCTTATTACTTGCCTTGATCGTCGGCACCCGATATTTGTCA
ACTGGGAATCCTCCTGGACCTATTATCGGTCCTTTGAAACTAACAGCAATTACCCCAGAAAGTGTCTGCTAA

>p1_ind4202

ATGCAAATAGACCCCGTTGCATATCATTGTTCCGAGTTGAAGTTCGTGCTTGTACCCGAAGAGTCGGGAAAGTTAT
CCCCGACGGAAATAGGATTCCATTTTCAGACTGGGCGCCGCGTACATGACAGAAGACCCCGCTCTGAGTCAGTGA
TGGCGCACCAACGCAGATATCGATGTCTCACGTCTACCTTATTACTTGCCTTGATCGTCGGCACCCGATATTTGTCA
ACTGGGAATCCTCCTGGACCTATTATCGGTCCTTTGAAACTAACAGCAATTACCCCAGAAAGTGTCTGCTAA

>p1_ind1649

ATGCAAATAGACCCCGTTGCATATCATTGTTCCGAGTTGAAGTTCGTGCTTGTACCCGAAGAGTCGGGAAAGTTAT
CCCCGACGGAAATAGGATTCCATTTTCAGACTGGACGCCGCGTACATGACAGAAGACCCCGCTCTGAGTCAGTGA

TGACGCACCAACGCAGATATCGATGTCTCACGTCTACCTTATTACTTGCCTTGATCGTCGGCACCCGATATTTGTCA
ACTGGGAATCCTCCTGGACCTATTATCGGTCCTTTGAAACTAACAGCAATTACCCCAGAAAGTGTCTGCTAA

>p1_ind373

ATGCAAATAGACCCCGTTGCATATCATTGTTCCGAGTTGAAGTTCGTGCTTGTACCCGAAGAGTCGGGAAAGTTAT
CCCCGACGGAAATAGGATTCCATTTTCAGATTGGGTGCCGCGTACATGACAGAAGACCCCGCTCTGAGTCAGTGA
TGACGCACCAACGCAGATATCGATGTCTCACGTCTACCTTATTACTTGCCTTGATCGTCGGCACCCGATATTTGTCA
ACTGGGAATCCTCCTGGACCTATTATCGGTCCTTTGAAACTAACAGCAATTACCCCAGAAAGTGTCTGCTAA

>p1_ind56

ATGCAAATAGACCCCGTTGCATATCATTGTTCCGAGTTGAAGTTCGTGCTTGTACCCGAAGAGTCGGGAAAGTTAT
CCCCGACGGAAATAGGATTCCATTTTCAGATTGGGTGCCGCGTACATGACAGAAGACCCCGCTCTGAGTCAGTGA
TGACGCACCAACGCAGATATCGATGTCTCACGTCTACCTTATTACTTGCCTTGATCGTCGGCACCCGATATTTGTCA
ACTGGGAATCCTCCTGGACCTATTATCGGTCCTTTGAAACTAACAGCAATTACCCCATAAAGTGTCTGCTAA

>p1_ind3562

ATGCAAATAGACCCCGTTGCATATCATTGTTCCGAGTTGAAGTTCGTGCTTGTACCCGAAGAGTCGGGAAAGTTAT
CCCCGACGGAAATAGGATTCCATTTTCAGACTGGGCGCCGCGTACATGACAGAAGACCCCGCTCTGAGTCAGTGA
TGACGCACCAACGCAGATATCGATGTCTCACGTCTACCTTATTACTTGCCTTGATCGTCGGCACCCGATATTTGTCA
ACTGGGAATCCTCCTGGACCTATTATCGGTCCTTTGAAACTAACAGCAATTACCCCAGAAAGTGTCTGCTAA

>p1_ind2213

ATGCAAATAGACCCCGTTGCATATCATTGTTCCGAGTTGAAGTTCGTGCTTGTACCCGAAGAGTCGGGAAAGTTAT
CCCCGACGGAAATAGGATTCCATTTTCAGACTGGGCGCCGCGTACATGACAGAAGACCCCGCTCTGAGTCAGTGA
TGACGCACCAACGCAGATATCGATGTCTCACGTCTACCTTATTACTTGCCTTGATCGTCGGCACCCGATATTTGTCA
ACTGGGAATCCTCCTGGACCTATTATCGGTCCTTTGAAACTAACAGCAATTACCCCAGAAAGTGTCTGCTAA

>p1_ind1459

ATGCAAATAGACCCCGTTGCATATCATTGTTCCGAGTTGAAGTTCGTGCTTGTACCCGAAGAGTCGGGAAAGTTAT
CCCCGACGGAAATAGGATTCCATTTTCAGACTGGGCGCCGCGTACATGACAGAAGACCCCGCTCTGAGTCAGTGA
TGACGCACCAACGCAGATATCGATGTCTCACGTCTACCTTATTACTTGCCTTGATCGTCGGCACCCGATATTTGTCA
ACTGGGAATCCTCCTGGACCTATTATCGGTCCTTTGAAACTAACAGCAATTACCCCAGAAAGTGTCTGCTAA

>p1_ind4700

AAGCAAATAGACCCCGTTGCATATCATTGTTCCGAGTTGAAGTTCGTGCTTGTACCCGAAGAGTCGGGAAAGTTAT
CCCCGACGGAAATAGGATTCCATTTTCAGACTGGGCGCCGCGTACATGACAGCAGACCCCGCTCTGAGTCAGTGA
TGACGCACCAACGCAGATATCGATGTCTCACGTCTACCTTATTACTTGCCTTGATCGTCGGCACCCGATATTTGTCA
ACTGGGAATCCTCCTGGACCTATTATCGGTCCTTTGAAACTAACAGCAATTACCCCAGAAAGTGTCTGCTAA

>p1_ind1952

ATGCAAATAGACCCTGTTGCATATCATTGTTCCGAGTTGAAGTTCGTGCTTGTACCCGAAGAGTCGGGAAAGTTAT
CCCCGACGGAAATAGGATTCCATTTTCAGATTGGGTGCCGCGTACATGACAGAAGACCCCGCTCTGAGTCAGTGA
TGGCGCACCAACGCAGATATCGATGTCTCACGTCTACCTTATTACTTGCCTTGATCGTCGGCACCCGATATTTGTCA
ACTGGGAATCCTCCTGGACCTATTATCGGTCCTTTGAAACTAACAGCAATTACCCCAGAAAGTGTCTGCTAA

>p1_ind3497

ATGCAAATAGACCCTGTTGCATATCATTGTTCCGAGTTGAAGTTCGTGCTTGTACCCGAAGAGTCGGGAAAGTTAT
CCCCGACGGAAATAGGATTACATTTTCAGATTGGGCGCCGCGTACATGACAGAAGACCCCGCTCTGAGTCAGTGA
TGACGCACCAACGCAGATATCGATGTCTCACGTCTACCTTATTACTTGCCTTGATCGTCGGCACCCGATATTTGTCA
ACTGGGAATCCTCCTGGACCTATTATCGGTCCTTTGAAACTAACAGCAATTACCCCAGAAAGTGTCTGCTAA

>p1_ind4942

ATGCAAATAGACCCCGTTGCATATCATTGTTCCGAGTTGAAGTTCGTGCTTGTACCCGAAGAGTCGGGAAAGTTAT
CCCCGACGGAAATAGGATTCCATTTTCAGATTGGGTGCCGCGTACATGACAGAAGACCCCGCTCTGAGTCAGTGA
TGGCGCACCAACGCAAATATCGATGTCTCACGTCTACCTTATTACTTGCCTTGATCGTCGGCACCCGATATTTGTCA
ACTGGGAATCCTCCTGGACCTATTATCGGTCCTTTGAAACTAACAGCAATTACCCCAGAAAGTGTCTGCTAA

>p1_ind1503

ATGCAAATAGACCCCGTTGCATATCATTGTTCCGAGTAGAAGTTCGTGCTTGTACCCGAAGAGTCGGGAAAGTTAT
CCCCGACGGAAATAGGATTCCATTTTCAGACTGGGCGCCGCGTACATGACAGAAGACCCCGCTCTGAGTCAGTGA
TGACGCACCAACGCAGATATCGATGTCTCACGTCTACCTTATTACTTGCCTTGATCGTCGGCACCCGATATTTGTCA
ACTGGGAATCCTCCTGGACCTATTATCGGTCCTTTGAAACTAACAGCAATTACCCCAGAAAGTGTCTGCTAA

>p1_ind2292

ATGCAAATAGACCCTGTTGCATATCATTGTTCCGAGTTGAAGTTCGTGCTTGTACCCGAAGAGTCGGGAAAGTTAT
CCCCGACGGAAATAGGATTCCATTTTCAGATTGGGTGCCGCGTACATGACAGAAGACCCCGCTCTGAGTCAGTGA
TGGCGCACCAACGCAAATACAGATGTCTCACGTCTACCTTATTACTTGCCTTGATCGTCGGCACCCGATATTTGTCA
ACTGGGAATCCTCCTGGACCTATTATCGGTCCTTTGAAACTAACAGCAATTACCCCAGAAAGTGTCTGCTAA

>p1_ind2000

ATGCAAATAGACCCCGTTGCATATCATTGTTCCGAGTTGAAGTTCGTGCTTGTACCCGAAGAGTCGGGAAAGTTAT
CCCCGACGGAAATAGGATTCCATTTTCAGATTGGGTGCCGCGCACATGACAGAAGACCCCGCTCTGAGTCAGTGA
TGACGCACCAACGCAGATATCGATGTCTCACGTCTACCTTATTACTTGCCTTGATCGTCGGCACCCGATATTTGTCA
ACTGGGAATCCCCCTGGACCTATTATCGGTCCTTTGAAACTAACAGCAATTACCCCAGAAAGTGTCTGCTAA

>p1_ind888

ATGCAAATAGACCCCGTTGCATATCATTGTTCCGAGTTGAAGTTCGTGCTTGTACCCGAAGAGTCGGGAAAGTTAT
CCCCGACGGAAATAGGATTCCATTTTCAGATTGGGTGCCGCGTACATGACAGAAGACCCCGCTCTGTGTCAGTGAT

GACGCACCTACGCAGATATCGATGTCTCACGTCTACCTTATTACTTGCCTTGATCGTCGGCACCCGATATTTGTCAA
CTGGGAATCCTCCTGGACCTATTATCGGTCCTTTGAAACTAACAGCAATTACCCCAGAAAGTGTCTGCTAA

>p1_ind3832

ATGCAAATAGACCCCGTTGCATATCATTGTTCCGAGTAGAAGTTCGTGCTTGTACCCGAAGAGTCGGGAAAGTTAT
CCCCGACGGAAATAGGATTCCATTTTCAGACTGGGCGCCGCGTACATGACAGAAGACCCCGCTCTGAGTCAGTGA
TGACGCACCAACGCAGATATCGATGTCTCACGTCTACCTTATTACTTGCCTTGATCGTCGGCACCCGATATTTGTCA
ACTGGGAATCCTCCTGGACCTATTATCGGTCCTTTGAAACTAACAGCAATTACCCCAGAAAGTGTCTGCTAA

>p1_ind322

ATGCAAATAGACCCCGTTGCATATCATTGTTCCGAGTTGAAGTTCGTGCTTGTACCCGAAGAGTCGGGAAAGTTAT
CCCCGACGGAAATAGGATTCCATTTTCAGACTGGGCGCCGCGTACATGACAGAAGACCCCGCTCTGAGTCAGTGA
TGACGCACCAACGCAGATATCGATGTCTCACGTCTACCTTATTACTTGCCTTGATCGTCGGCACCCGATATTTGTCA
ACTGGGAATCCTCCTGGACCTATTCTCGGTCCTTTGAAACTAACAGCAATTACCCCAGAAAGTGTCTGCTAA

>p1_ind302

AAGCAAATAGACCCCGTTGCATATCATTGTTCCGAGTTGAAGTTCGTGCTTGTACCCGAAGAGTCGGGAAAGTTAT
CCCCGACGGAAATAGGATTCCATTTTCAGACTGGGCGCCGCGTACATGACAGAAGACCCCGCTCTGAGTCAGTGA
TGACGCACCAACGCAGATATCGATGTCTCACGTCTACCTTATTACTTGCCTTGATCGTCGGCACCCGATATTTGTCA
ACTGGGAATCCTCCTGGACCTATTATCGGTCCTTTGAAACTAACAGCAATTACCCCAGAAAGTGTCTGCTAA

>p1_ind1816

ATGCAAATAGACCCCGTTGCATATCATTGTTCCGAGTTGAAGTTCGTGCTTGTACCCGAAGAGTCGGGAAAGTTAT
CCCCGACGGAAATAGGATTCCATTTTCAGATTGGGTGCCGCGCACATGACAGAAGACCCCGCTCTGAGTCAGTGA
TGACGCACCAACGCAGATATCGATGTCTCACGTCTACCTTATTACTTGCCTTGATCGTCGGCACCCGATATTTGTCA
ACTGGGAATCCCCCTGGACCTATTATCGGTCCTTTGAAACTAACAGCAATTACCCCAGAAAGTGTCTGCTAA

>p1_ind854

ATGCAAATAGACCCCGTTGCATATCATTGTTCCGAGTTGAAGTTCGTGCTTGTACCCGAAGAGTCGGGAAAGTTAT
CCCCGACGGAAATAGGATTCCATTTTCAGATTGGGTGCCGCGTACATGACAGAAGACCCCGCTCTGAGTCAGTGA
TGACGCACCAACGCAGATATCGATGTCTCACGTCTACCTTATTACTTGCCTTGATCGTCGGCACCCGATATTTGTCA
ACTGGGAATCCTCCTGGACCTATTCTCGGTCCTTTGAAACTAACAGCAATTACCCCAGAAAGTGTCTGCTAA

>p1_ind2063

ATGCAAATAGACCCCGTTGCATATCATTGTTCCGAGTTGAAGTTCGTGCTTGTACCCGAAGAGTCGGGAAAGTTAT
CCCCGACGGAAATAGGATTCCATTTTCAGACTGGGCGCCGCGTACATGACAGAAGACCCCGCTCTGAGTCAGTGA
TGACGCACCAATGCAGATATCGATGTCTCACGTCTACCTTATTACTTGCCTTGATCGTCGGCACCCGATATTTGTCA
ACTGGGAATCCTCCTGGACCTATTATCGGTCCTTTGAAACTAACAGCAATTACCCCAGAAAGTGTCTGCTAA

>p1_ind2666

ATGCAAATAGACCCCGTTGCATATCATTGTTCCGAGTAGAAGTTCGTGCTTGTACCCGAAGAGTCGGGAAAGTTAT
CCCCGACGGAAATAGGATTCCATTTTCAGACTGGGCGCCGCGTACATGACAGAAGACCCCGCTCTGAGTCAGTGA
TGACGCACCAACGCAGATATCGATGTCTCACGTCTACCTTATTACTTGCCTTGATCGTCGGCACCCGATATTTGTCA
ACTGGGAATCCTCCTGGACCTATTATCGGTCCTTTGAAACTAACAGCAATTACCCCAGAAAGTGTCTGCTAA

>p1_ind2976

ATGCAAATAGACCCCGTTGCATATCATTGTTCCGAGTTGAAGTTCGTGCTTGTACCCGAAGAGTCGGGAAAGTTAT
CCCCGACGGAAATAGGATTCCATTTTCAGATTGGGCGCCGCGTACATGACAGAAGACCCCGCTCTGAGTCAGTGA
TGACGCACCAACGCAGATATCGATGTCTCACGTCTACCTTATTACTTGCCTTGATCGTCGGCACCCGATATTTGTCA
ACTGGGAATCCTCCTGGACCTATTATCGGTCCTTTGAAACTAACAGCAATTACCCCAGAAAGTGTCTGCTAA

>p1_ind3280

ATGCAAATAGACCCCGTTGCATATCATTGATCCGAGTTGAAGTTCGTGCTTGTACCCGAAGAGTCGGGAAAGTTAT
CCCCGACGGAAATAGGATTCCATTTTCAGATTGGGTGCCGCGTACATGACAGAAGACCCCGTTCTGAGTCAGTGAT
GACGCACCAACGCAGATATCGATGTCTCACGTCTACCTTATTACTTGCCTTGATCGTCGGCACCCGATATTTGTCAA
CTGGGAATCCTCCTGGACCTATTATCGGTCCTTTGAAACTAACAGCAATTACCCCAGAAAGTGTCTGCTAA

>p1_ind1949

ATGCAAATAGACCCCGTTGCATATCATTGTTCCGAGTTGAAGTTCGTGCTTGTACCCGAAGAGTCGGGAAAGTTAT
CCCCGACGGAAATAGGATTCCATTTTCAGACTGGGCGCCGCGTACATGACAGAAGACCCCGCTCTGAGTCAGTGA
TGCCGCACCAACGCAGATATCGATGTCTCACGTCTACCTTATTACTTGCCTTGATCGTCGGCACCCGATATTTGTCA
ACTGGGAATCCTCCTGGACCTATTATCGGTCCTTTGAAACTAACAGCAATTACCCCAGAAAGTGTCTGCTAA

>p1_ind3384

ATGCAAATAGACCCCGTTGCATATCATTGTTCCGAGTTGAAGTTCGTGCTTGTACCCGAAGAGTCGGGAAAGTTAT
CCCCGACGGAAATAGGATTCCATTTTCAGATTGGGTGCCGCGCACATGACAGAAGACCCCGCTCTGAGTCAGTGA
TGACGCACCAACGCAGATATCGATGTCTCACGTCTACCTTATTACTTGCCTTGATCGTCGGCACCCGATATTTGTCA
ACTGGGAATCCCCCTGGACCTATTATCGGTCCTTTGAAACTAACAGCAATTACCCCAGAAAGTGTCTGCTAA

>p1_ind2496

ATGCAAATAGACCCCGTTGCATATCATTGTTCCGAGTTGAAGTTCGTGCTTGTACCCGAAGAGTCGGGAAAGTTAT
CCCCGACGGAAATAGGATTCCATTTTCAGACTGGGCGCCGCGTACATGACAGAAGACCCCGCTCTGAGTCAGTGA
TGACGCACCAACGCAGATATCGATGTCTCACGTCTACCTTATTACTTGCCTTGATCGTCGGCACCCGATATTTGTCA
ACTGGGAATCCTCCTGGACCTATTATCGGTCCTTTGAAACTAACAGCAATTACCCCAGAAAGTGTCTGCTAA

>p1_ind893

ATGCAAATAGACCCCGTTGCATATCATTGTTCCGAGTTGAAGTTCGTGCTTGTACCCGAAGAGTCGGGAAAGTTAT
CCCCGACGGAAATAGGATTCCATTTTCAGACTGGGCGCCGCGTACATGACAGAAGACCCCGCTCTGAGTCAGTGA

TGACGCACCAACGCAGATATCGATGTCTCACGTCTACCTTATTACTTGCCTTGATCGTCGGCACCCGATATTTGTCA
ACTGGGAATCCTCCTGGACCTATTATCGGTCCTTTGAAACTAACAGCAATTACCCCAGAAAGTGTCTGCTAA

>p1_ind982

ATGCAAATAGACCCCGTTGCATATCATCGTTCCGAGTTGAAGTTCGTGCTTGTACCCGAAGAGTCGGGAAAGTTAT
CCCCGACGGAAATAGGATTCCATTTTCAGATTGGGTGCCGCGTACATGACAGAAGACCCCGCTCTGAGTCAGTGA
TGACGCACCAACGCAGATATCGATGTCTCACGTCTACCTTATTACTTGCCTTGATCGTCGGCACCCGATATTTGTCA
ACTGGGAATCCTCCTGGACCTATTATCGGTCCTTTGAAACTAACAGCAATTACCCCAGAAAGTGTCTGCTAA

>p1_ind2960

ATGCAAATAGACCCCGTTGCATATCATTGTTCCGAGTTGAAGTTCGTGCTTGTACCCGAAGAGTCGGGAAAGTTAT
CCCCGACGGAAATAGGATTCCATTTTCAGACTGGGCGCCGCGTACATGACAGAAGACCCCGCTCTGAGTCAGTGA
TGGCGCACCAACGCAAATATCGATGTCTCACGTCTACCTTATTACTTGCCTTGATCGTCGGCACCCGATATTTGTCA
ACTGGGAATCCTCCTGGACCTATTATCGGTCCTTTGAAACTAACAGCAATTACACCAGAAAGTGTCTGCTAA

>p1_ind1509

ATGCAAATAGACCCCGTTGCATATCATTGTTCCGAGTTGAAGTTCGTGCTTGTACCCGAAGAGTCGGGAAAGTTAT
CCCCGACGGAAATAGGATTCCATTTTCAGACTGGGCGCCGCGTACATGACAGAAGACCCCGCTCTGAGTCAGTGA
TGGCGCACCAACGCAAATATCGATGTCTCACGTCTACCTTATTACTTGCCTTGATCGTCGGCACCCGATATTTGTCA
ACTGGGAATCCTCCTGGACCTATTATCGGTCCTTTGAAACTAACAGCAATTACACCAGAAAGTGTCTGCTAA

>p1_ind172

ATGCAAATAGACCCCGTTGCATATCATTGTTCCGAGTTGAAGTTCGTGCTTGTACCCGAAGAGTCGGGAAAGTTAT
CCCCGACGGAAATAGGATTCCATTTTCAGACTGGGCGCCGCGTACATGACAGAAGACCCCGCTCTGAGTCAGTGA
TGACGCACCAACGCAGATATCGATGTCTCACGTCTACCTTATTACTTGCCTTGATCGTCGGCACCCGATATTTGTCA
ACTGGGAATCCTCCTGGACCTATTATCGGTCCTTTGAAACTAACAGCAATTACCCCAGAAAGTGTCTGCTAA

>p1_ind1514

ATGCAAATAGACCCCGTTGCATATCATTGTTCCGAGTTGAAGTTCGTGCTTGTACCCGAAGAGTCGGGAAAGTTAT
CCCCGACGGAAATAGGATTCCATTTTCAGACTGGGCGCCGCGTACATGACAGAAGACCCCGCTCTGAGTCAGTGA
TGGCGCACCAACGCAGATATCGATGTCTCACGTCTACCTTATTACTTGCCTTGATCGTCGGCACCCGATATTTGTCA
ACTGGGAATCCTCCTGGACCTATTATCGGTCCTTTGAAACTAACAGCAATTACCCCAGAAAGTGTCTGCTAA

>p1_ind2459

ATGCAAATAGACCCCGTTGCATATCATTGTTCCGAGTTGAAGTTCGTGCTTGTACCCGAAGAGTCGGGAAAGTTAT
CCCCGACGGAAATAGGATTCCATTTTCAGACTGGGCGCCGCGTACATGACAGAAGACCCCGCTCTGAGTCAGTGA
TGACGCACCAACGCAGATATCGATGTCTCACGTCTACCTTATTACTTGCCTTGATCGTCGGCACCCGATATTTGTCA
ACTGGGAATCCTCCTGGACCTATTATCGGTCCTTTGAAACTAACAGCAATTACCCCAGAAAGTGTCTGCTAA

>p1_ind2202

ATGCAAATAGACCCCGTTGCATATCATTGTTCCGAGTTGAAGTTCGTGCTTGTACCCGAAGAGTCGGGAAAGTTAT
CCCCGACGGAAATAGGATTCCATTTTCAGACTGGGCGCCGCGTACATGACAGAAGACCCCGCTCTGAGTCAGTGA
TGACGCACCAACGCAGATATCGATGTCTCACGTCTACCTTATTACTTGCCTTGATCGTCGGCACCCGATATTTGTCA
ACTGGGAATCCTCCTGGACCTATTATCGGTCCTTTGAAACTAACAGCAATTACCCCAGAAAGTGTCTGCTAA

>p1_ind1017

ATGCAAATAGACCCCGTTGCATATCATTGTTCCGAGTTGAAGTTCGTGCTTGTACCCGAAGAGTCGGGAAAGTTAT
CCCCGACGGAAATAGGATTCCATTTTCAGACTGGGCGCCGCGTACATGACAGAAGACCCCGCTCTGAGTCAGTGA
TGACGCACCAACGCAGATATCGATGTCTCACGTCTACCTTATTACTTGGCTTGATCGTCGGCACCCGATATTTGTCA
ACTGGGAATCCTCCTGGACCTATTATCGGTCCTTTGAAACTAACAGCAATTACCCCAGAAAGTGTCTGCTAA

>p1_ind3155

ATGCAAATAGACCCCGTTGCATATCATTGTTCCGAGTTGAAGTTCGTGCTTGTACCCGAAGAGTCGGGAAAGTTAT
CCCCGACGGAAATAGGATTCCATTTTCAGATTGGGTGCCGCGCACATGACAGAAGACCCCGCTCTGAGTCAGTGA
TGACGCACCAACGCAGATATCGATGTCTCACGTCTACCTTATTACTTGCCTTGATCGTCGGCACCCGATATTTGTCA
ACTGGGAATCCCCCTGGACCTATTATCGGTCCTTTGAAACTAACAGCAATTACCCCAGAAAGTGTCTGCTAA

>p1_ind4464

ATGCAAATAGACCCCGTTGCATATCATTGTTCCGAGTTGAAGTTCGTGCTTGTACCCGAAGAGTCGGGAAAGTTAT
CCCCGACGGAAATAGGATTCCATTTTCAGATTGGGTGCCGCGTACATGACAGAAGACCCCGCTCTGAGTCAGTGA
TGACGCACCAACGCAGATATCGATGTCTCACGTCTACCTTATTACTTGCCTTGATCGTCGGCACCCGATATTTGTCA
ACTGGGAATCCTCCTGGACCTATTATCGGTCCTTTGAAACTAACAGCAATTACCCCAGAAAGTGTCTGCTAA

>p1_ind4761

ATGCAAATAGACCCCGTTGCATATCATTGTTCCGAGTTGAAGTTCGTGCTTGTACCCGAAGAGTCGGGAAAGTTAT
CCCCGACGGAAATAGGATTCCATTTTCAGACTGGGCGCCGCGTACATGACAGAAGACCCCGCTCTGAGTCAGTGA
TGACGCACCAACGCAGATATCGATGTCTCACGTCTACCTTATTACTTGCCTTGATCGTCGGCACCCGATATTTGTCA
ACTGGGAATCCTCCTGGACCTATTCTCGGTCCTTTGAAACTAACAGCAATTACCCCAGAAAGTGTCTGCTAA

>p1_ind154

ATGCAAATAGACCCCGTTGCATATCATTGTTCCGAGTTGAAGTTCGTGCTTGTACCCGAAGAGTCGGGAAAGTTAT
CCCCGACGGAAATAGGATTCCATTTTCAGATTGGGTGCCGCGTACATGACAGAAGACCCCGCTCTGAGTCAGTGA
TGGCGCACCAACGCAAATACAGATGTCTCACGTCTACCTTAATACTTGCCTTGATCGTCGGCACCCGATATTTGTCA
ACTGGGAATCCTCCTGGACCTATTATCGGTCCTTTGAAACTAACAGCAATTACCCCAGAAAGTGTCTGCTAA

>p1_ind2619

ATGCAAATAGACCCCGTTGCATATCATTGTTCCGAGTTGAAGTTCGTGCTTGTACCCGAAGAGTCGGGAAAGTTAT
CCCCGACGGAAATAGGATTCCATTTTCAGATTGGGTGCCGCGCACATGACAGAAGACCCCGCTCTGAGTCAGTGA

TGGCGCACCAACGTAGATATCGATGTCTCACGTCTACCTTATTACTTGCCTTGATCGTCGGCACCCGATATTTGTCA
ATTGGGAATCCTCCTGGACCTATTATCGGTCCTTTGAAACTAACAGCAATTACCCCAGAAAGTGTCTGGTAA

>p1_ind3250

ATGCAAATAGACCCCGTTGCATATCATTGTTCCGAGTTGAAGTTCGTGCTTGTACCCGAAGAGTCGGGAAAGTTAT
CCCCGACGGAAATAGGATTCCATTTTGAGATTGGGTGCCGCGTACATGACAGAAGACCCCGCTCTGAGTCAGTGA
TGGCGCACCAACGCAAATATCGATGTCTCACGTCTACCTTATTACTTGCCTTGATCGTCGGCACCCGATATTTGTCA
ACTGGGAATCCTCCTGGACCTATTATCGGTCCTTTGAAACTAACAGCAATTACCCCAGAAAGTGTCTGCTAA

>p1_ind5044

ATGCAAATAGACCCCGTTGCATATCATTGTTCCGAGTTGAAGTTCGTGCTTGTACCCGAAGAGTCGGGAAAGTTAT
CCCCGACGGAAATAGGATTCCATTTTCAGATTGGGTGCCGCGTACATGACAGAAGACCCCGCTCTGAGTCAGTGA
TGACGCACCAACGCAGATATCGATGTCTCACGTCTACCTTATTACTTGCCTTGATCGTCGGCACCCGATATTTGTCA
ACTGGGAATCCTCCTGGACCTATTATCGGTCCTTTGAAACTAACAGCAATTACCCCAGAAAGTGTCTGCTAA

>p1_ind1472

ATGCAAATAGACCCCGTTGCATATCATTGTTCCGAGTTGAAGTTCGTGCTTGTACCCGAAGAGTCGGGAAAGTTAT
CCCCGACGGAAATAGGATTCCATTTTCAGATTGGGTGCCGCGTACATGACAGAAGACCCCGCCCTGAGTCAGTGA
TGACGCACCAACGCAGATATTGATGTCTCACGTCTACCTTATTACTTGCCTTGATCGTCGGCACCCGATATTTGTCA
ACTGGGAATCCTCCTGGACCTATTATCGGTCCTTTGAAACTAACAGCAATTACCCCAGAAAGTGTCTGCTAA

>p1_ind2898

ATGCAAATAGACCCCGTTGCATATCATTGTTCCGAGTTGAAGTTCGTGCTTGTACCCGAAGAGTCGGGAAAGTTAT
CCCCGACGGAAATAGGATTCCATTTTCAGACTGGGCGCCGCGTACATGACAGAAGACCCCGCTCTGAGTCAGTGA
TGACGCACCAACGCAGATATCGATGTCTCACGTCTACCTTATTACTTGCCTTGATCGTCGGCACCCGATATTTGTCA
ACTGGGAATCCTCCTGGACCTATTATCGGTCCTTTGAAACTAACAGCAATTACCCCAGAAAGTGTCTGCTAA

>p1_ind2797

ATGCAAATAGACCCCGTTGCATATCATTGTTCCGAGTTGAAGTTCGTGCTTGTACCCGAAGAGTCGGGAAAGTTAT
CCCCGACGGAAATAGGATTCCATTTTCAGATTGGGTGCCGCGCACATGACAGAAGACCCCGCTCTGAGTCAGTGA
TGACGCACCAACGCAGATATCGATGTCTCACGTCTACCTTATTACTTGCCTTGATCGTCGGCACCCGATATTTGTCA
ACTGGGAATCCCCCTGGACCTATTATCGGTCCTTTGAAACTAACAGCAATTACCCCAGAAAGTGTCTGCTAA

>p1_ind129

ATGCAAATAGACCCCGTTGCATATCATTGTTCCGAGTTGAAGTTCGTGCTTGTACCCGAAGAGTCGGGAAAGTTAT
CCCCGACGGAAATAGGATTCCATTTTCAGATTGGGTGCCGCGTACATGACAGAAGACCCCGCTCTGAGTCAGTGA
TGACGCACCAACGCAGATATCGATGTCTCACGTCTACCTTATTACTTGCCTTGATCGTCGGCACCCGATATTTGTCA
ACTGGGAATCCTCCTGGACCTATTATCGGTCCTTTGAAACTAACAGCAATTACCCCAGAAAGTGTCTGCTAA

>p1_ind1155

ATGCAAATAGACCCCGTTGCATATCATTGTTCCGAGTTGAAGTTCGTGCTTGTACCCGAAGAGTCGGGAAAGTTAT
CCCCGACGGAAATAGGATTCCATTTTCAGATTGGGCGCCGCGTACATGACAGAAGACCCCGCTCTGAGTCAGTGA
TGACGCACCAACGCAGATATCGATGTCTCACGTCTACCTTATTACTTGCCTTGATCGTCGGCACCCGATATTTGTCA
ACTGGGAATCCTCCTGGACCTATTATCGGTCCTTTGAAACTAACAGCAATTACCCCAGAAAGTGTCTGCTAA

>p1_ind2917

ATGCAAATAGACCCCGTTGCATATCATTGTTCCGAGTAGAAGTTCGTGCTTGTACCCGAAGAGTCGGGAAAGTTAT
CCCCGACGGAAATAGGATTCCATTTTCAGACTGGGCGCCGCGTACATGACAGAAGACCCCGCTCTGAGTCAGTGA
TGACGCACCTACGCAGATATCGATGTCTCACGTCTACCTTATTACTTGCCTTGATCGTCGGCACCCGATATTTGTCA
ACTGGGAATCCTCCTGGACCTATTATCGGTCCTTTGAAACTAACAGCAATTACCCCAGAAAGTGTCTGCTAA

>p1_ind3809

ATGCAAATAGACCCCGTTGCATATCATTGTTCCGAGTTGAAGTTCGTGCTTGTACCCGAAGAGTCGGGAAAGTTAT
CCCCGACGGAAATAGGATTCCATTTTGAGATTGGGTGCCGCGTACATGACAGAAGACCCCGCTCTGAGTCAGTGA
TGGCGCACCAACGCAAATATCGATGTCTCACGTCTACCTTATTACTTGCCTTGATCGTCGGCACCCGATATTTGTCA
ACTGGGAATCCTCCTGGACCTATTATCGGTCCTTTGAAACTAACAGCAATTACCCCAGAAAGTGTCTGCTAA

>p1_ind1521

ATGCAAATAGACCCCGTTGCATATCATTGTTCCGAGTTGAAGTTCGTGCTTGTACCCGAAGAGTCGGGAAAGTTAT
CCCCGACGGAAATAGGATTCCATTTTCAGATTGGGCGCCGCGTACATGACAGAAGACCCCGCTCTGAGTCAGTGA
TGACGCACCAACGCAGATATCGATGTCTCACGTCTACCTTATTACTTGCCTTGATCGTCGGCACCCGATATTTGTCA
ACTGGGAATCCTCCTGGACCTATTATCGGTCCTTTGAAACTAACAGCAATTACCCCAGAAAGTGTCTGCTAA

>p1_ind5021

ATGCAAATAGACCCCGTTGCATATCATTGTTCCGAGTTGAAGTTCGTGCTTGTACCCGAAGAGTCGGGAAAGTTAT
CCCCGACGGAAATAGGATTCCATTTTCAGACTGGGCGCCGCGTACATGACAGAAGACCCCGCTCTGAGTCAGTGA
TGCCGCACCAACGCAGATATCGATGTCTCACGTCTACCTTATTACTTGCCTTGATCGTCGGCACCCGATATTTGTCA
ACTGGGAATCCTCCTGGACCTATTATCGGTCCTTTGAAACTAACAGCAATTACCCCAGAAAGTGTCTGCTAA

>p1_ind315

ATGCAAATAGACCCCGTTGCATATCATTGTTCCGAGTTGAAGTTCGTGCTTGTACCCGAAGAGTCGGGAAAGTTAT
CCCCGACGGAAATACGATTCCATTTTCAGATTGGGTGCCGCGTACATGACAGAAGACCCCGCTCTGAGTCAGTGAT
GACGCACCAACGCAGATATCGATGTCTCACGTCTACCTTATTACTTGCCTTGATCGTCGGCACCCGATATTTGTCAA
CTGGGAATCCTCCTGGACCTATTATCGGTCCTTTGAAACTAACAGCAATTACCCCAGAAAGTGTCTGCTAA

>p1_ind722

ATGCAAATAGACCCCGTTGCATATCATTGTTCCGAGTTGAAGTTCGTGCTTGTACCCGAAGAGTCGGGAAAGTTAT
CCCCGACGGAAATAGGATTCCATTTTCAGACTGGGCGCCGCGTACATGACAGAAGACCCCGCTCTGAGTCAGTGA

TGACGCACCAACGCAGATATCGATGTCTCACGTCTACCTTATTACTTGCCTTGATCGTCGGCACCCGATATTTGTCA
ACTGGGAATCCTCCTGGACCTATTATCGGTCCTTTGAAACTAACAGCAATTACCCCAGAAAGTGTCTGCTAA

>p1_ind2999

ATGCAAATAGACCCCGTTGCATATCATTGTTCCGAGTTGAAGTTCGTGCTTGTACCCGAAGAGTCGGGAAAGTTAT
CCCCGACGGAAATAGGATTCCATTTTCAGATTGGGCGCCGCGTACATGACAGAAGACCCCGCTCTGAGTCAGTGA
TGACGCACCAACGCAGATATCGATGTCTCACGTCTACCTTATTACTTGCCTTGATCGTCGGCACCCGATATTTGTCA
ACTGGGAATCCTCCTGGACCTATTATCGGTCCTTTGAAACTAACAGCAATTACCCCAGAAAGTGTCTGCTAA

>p1_ind2027

ATGCAAATAGACCCTGTTGCATATCATTGTTCCGAGTTGAAGTTCGTGCTTGTACCCGAAGAGTCGGGAAAGTTAT
CCCCGACGGAAATAGGATTCCATTTTCAGATTGGGTGCCGCGTACATGACAGAAGACCCCGCTCTGAGTCAGTGA
TGGCGCACCAACGCAGATATCGATGTCTCACGTCTACCTTATTACTTGCCTTGATCGTCGGCACCCGATATTTGTCA
ACTGGGAATCCTCCTGGACCTATTATCGGTCCTTTGAAACTAACAGCAATTACCCCAGAAAGTGTCTGCTAA

>p1_ind5127

ATGCAAATAGACCCCGTTGCATATCATTGTTCCGAGTTGAAGTTCGTGCTTGTACCCGAAGAGTCGGGAAAGTTAT
CCCCGACGGAAATAGGATTCCATTTTCAGATTGGGTGCCGCGTACATGACAGAAGACCCCGCTCTGAGTCAGTGA
TGACGCACCAACGCAGATATCGATGTCTCACGTCTACCTTATTACTTGCCTTGATCGTCGGCACCCGATATTTGTCA
ACTGGGAATCCTCCTGGACCTATTATCGGTCCTTTGAAACTAACAGCAATTACCCCAGAAAGTGTCTGCTAA

>p1_ind4844

ATGCAAATAGACCCCGTTGCATATCATTGTTCCGAGTTGAAGTTCGTGCTTGTACCCGAAGAGTCGGGAAAGTTAT
CCCCGACGGAAATAGGATTCCATTTTCAGATTGGGTGCCGCGCACATGACAGAAGACCCCGCTCTGAGTCAGTGA
TGACGCACCAACGCAGATATCGATGTCTCACGTCTACCTTATTACTTGCCTTGATCGTCGGCACCCGATATTTGTCA
ACTGGGAATCCCCCTGGACCTATTATCGGTCCTTTGAAACTAACAGCAATTACCCCAGAAAGTGTCTGCTAA

>p1_ind2788

ATGCAAATAGACCCCGTTGCATATCATTGTTCCGAGTTGAAGTTCGTGCTTGTACCCGAAGAGTCGGGAAAGTTAT
CCCCGACGGAAATAGGATTCCATTTTCAGATTGGGTGCCGCGTACATGACAGAAGACCCCGCTCTGAGTCAGTGA
TGACGCACCAACGCAGATATCGATGTCTCACGTCTACCTTATTACTTGCCTTGATCGTCGGCACCCGATATTTGTCA
ACTGGGAATCCTCCTGGACCTATTATCGGTCCTTTGAAACTAACAGCAATTACCCCAGAAAGTGTCTGCTAA

>p1_ind2799

ATGCAAATAGACCCCGTTGCATATCATTGTTCCGAGTTGAAGTTCGTGCTTGTACCCGAAGAGTCGGGAAAGTTAT
CCCCGACGGAAATAGGATTCCATTTTCAGATTGGGTGCCGCGTACATGACAGAAGACCCCGCTCTGAGTCAGTGA
TGACGCACCAACGCAGATATCGATGTCTCACGTCTACCTTATTACTTGCCTTGATCGTCGGCACCCGATATTTGTCA
ACTGGGAATCCTCCTGGACCTATTATCGGTCCTTTGAAACTAACAGCAATTACTCCAGAAAGTGTCTGCTAA

>p1_ind2860

ATGCAAATAGACCCCGTTGCATATCATTGTTCCGAGTTGAAGTTCGTGCTTGTACCCGAAGAGTCGGGAAAGTTAT
CCCCGACGGAAATAGGATTCCATTTTCAGATTGGGTGCCGCGTACATGACAGAAGACCCCGCTCTGAGTCAGTGA
TGACGCACCAACGCAGATATCGATGTCTCACGTCTACCTTATTACTTGCCTTGATCGTCGGCACCCGATATTTGTCA
ACTGGGAATCCTCCTGGACCTATTATCGGTCCTTTGAAACTAACAGCAATTACCCCAGAAAGTGTCTGCTAA

>p1_ind1268

ATGCAAATAGACCCCGTTGCATATCATTGTTCCGAGTTGAAGTTCGTGCTTGTACCCGAAGAGTCGGGAAAGTTAT
CCCCGACGGAAATAGGATTCCATTTTCAGACTGGGCGCCGCGTACATGACAGAAGACCCCGCTCTGAGTCAGTGA
TGACGCACCAACGCAGATATCGATGTCTCACGTCTACCTTATTACTTGCCTTGATCGTCGGCACCCGATATTTGTCA
ACTGGGAATCCTCCTGGACCTATTATCGGTCCTTTGAAACTAACAGCAATTACCCCAGAAAGTGTCTGCTAA

>p1_ind2838

ATGCAAATAGACCCCGTTGCATATCATTGTTCCGAGTTGAAGTTCGTGCTTGTACCCGAAGAGTCGGGAAAGTTAT
CCCCGACGGAAATAGGATTCCATTTTCAGATTGGGTGCCGCGTACATGACAGAAGACCCCGCTCTGAGTCAGTGA
TGACGCACCAACGCAGATATCGATGTCTCACGTCTACCTTATTACTTGCCTTGATCGTCGGCACCCGATATTTGTCA
ACTGGGAATCCTCCTGGACCTATTATCGGTCCTTTGAAACTAACAGCAATTACCCCAGAAAGTGTCTGCTAA

>p1_ind3696

ATGCAAATAGACCCCGTTGCATATCATTGTTCCGAGTAGAAGTTCGTGCTTGTACCCGAAGAGTCGGGAAAGTTAT
CCCCGACGGAAATAGGATTCCATTTTCAGACTGGGCGCCGCGTACATGACAGAAGACCCCGCTCTGAGTCAGTGA
TGACGCACCAACGCAGATATCGATGTCTCACGTCTACCTTATTACTTGCCTTGATCGTCGGCACCCGATATTTGTCA
ACTGGGAATCCTCCTGGACCTATTATCGGTCCTTTGAAACTAACAGCAATTACCCCAGAAAGTGTCTGCTAA

>p1_ind1040

ATGCAAATAGACCCCGTTGCATATCATTGTTCCGAGTAGAAGTTCGTGCTTGTACCCGAAGAGTCGGGAAAGTTAT
CCCCGACGGAAATAGGATTCCATTTTCAGACTGGGCGCCGCGTACATGACAGAAGACCCCGCTCTGAGTCAGTGA
TGACGCACCAACGCAGATATCGATGTCTCACGTCTACCTTATTACTTGCCTTGATCGTCGGCACCCGATATTTGTCA
ACTGGGAATCCTCCTGGACCTATTATCGGTCCTTTGAAACTAACAGCAATTACCCCAGAAAGTGTCTGCTAA

>p1_ind3471

ATGCAAATAGACCCCGTTGCATATCATTGTTCCGAGTTGAAGTTCGTGCTTGTACCCGAAGAGTCGGGAAAGTTAT
CCCCGACGGAAATAGGATTCCATTTTCAGATTGGGTGCCGCGTACATGACAGAAGACCCCGCTCTGAGTCAGTGA
TGACGCACCAACGCAGATATCGATGTCTCACGTCTACCTTATTACTTGCCTTGATCGTCGGCACCCGATATTTGTCA
ACTGGGAATCCTCCTGGACCTATTATCGGTCCTTTGAAACTAACAGCAATTACCCCAGAAAGTGTCTGCTAA

>p1_ind1043

ATGCAAATAGACCCCGTTGCATATCATTGTTCCGAGTTGAAGTTCGTGCTTGTACCCGAAGAGTCGGGAAAGTTAT
CCCCGACGGAAATAGGATTCCATTTTCAGACTGGGCGCCGCGTACATGACAGAAGACCCCGCTCTGAGTCAGTGA

TGGCGCACCAACGCAAATATCGATGTCTCACGTCTACCTTATTACTTGCCTTGATCGTCGGCACCCGATATTTGTCA
ACTGGGAATCCTCCTGGACCTATTATCGGTCCTTTGAAACTAACAGCAATTACACCAGAAAGTGTCTGCTAA

>p1_ind1453

ATGCAAATAGACCCCGTTGCATATCATTGTTCCGAGTAGAAGTTCGTGCTTGTACCCGAAGAGTCGGGAAAGTTAT
CCCCGACGGAAATAGGATTCCATTTTCAGACTGGGCGCCGCGTACATGACAGAAGACCCCGCTCTGAGTCAGTGA
TGACGCACCAACGCAGATATCGATGTCTCACGTCTACCTTATTACTTGCCTTGATCGTCGGCACCCGATATTTGTCA
ACTGGGAATCCTCCTGGACCTATTATCGGTCCTTTGAAACTAACAGCAATTACCCCAGAAAGTGTCTGCTAA

>p1_ind747

ATGCAAATAGACCCTGTTGCATATCATTGTTCCGAGTTGAAGTTCGTGCTTGTACCCGAAGAGTCGGGAAAGTTAT
CCCCGACGGAAATAGGATTCCATTTTCAGATTGGGTGCCGCGTACATGACAGCAGACCCCGCTCTGAGTCAGTGAT
GGCGCACCAACGCAAATATCGATGTCTCACGTCTACCTTATTACTTGCCTTGATCGTCGGCACCCGATTTTTGTCAA
CTGGGAATCCTCCTGGACCTATTATCGGTCCTTTGAAACTAACAGCAATTACCCCAGAAAGTGTCTGCTAA

>p1_ind2098

ATGCAAATAGACCCCGTTGCATATCATTGTTCCGAGTTGAAGTTCGTGCTTGTACCCGAAGAGTCGGGAAAGTTAT
CCCCGACGGAAATAGGATTCCATTTTCAGATTGGGTGCCGCGTACATGACAGAAGACCCCGCTCTGAGTCAGTGA
TGACGCCCCAACGCAGATATCGATGTCTCACGTCTACCTTATTAATTGCCTTGATCGTCGGCACCCGATATTTGTCA
ACTGGGAATCCTCCTGGACCTATTATCGGTCCTTTGAAACTAACAGGAATTACCCCAGAAAGTGTCTGCTAA

>p1_ind2727

ATGCAAATAGACCCCGTTGCATATCATTGTTCCGAGTTGAAGTTCGTGCTTGTACCCGAAGAGTCGGGAAAGTTAT
CCCCGACGGAAATAGGATTCCATTTTCAGACTGGGCGCCGCGTACATGACAGAAGACCCCGCTCTGAGTCAGTGA
TGCCGCACCAACGCAGATATCGATGTCTCACGTCTACCTTATTACTTGCCTTGATCGTCGGCACCCGATATTTGTCA
ACTGGGAATCCTCCTGGACCTATTATCGGTCCTTTGAAACTAACAGCAATTACCCCAGAAAGTGTCTGCTAA

>p1_ind561

ATGCAAATAGACCCCGTTGCATATCATTGTTCCGAGTTGAAGTTCGTGCTTGTACCCGAAGAGTCGGGAAAGTTAT
CCCCGACGGAAATAGGATTCCATTTTCAGATTGGGTGCCGCGTACATGACAGAAGACCCCGCTCTGAGTCAGTGA
TGGCGCACCAACGCAAATATCGATGTCTCACGTCTACCTTATTACTTGCCTTGATCGTCGGCACCCGATATTTGTCA
ACTGGGAATCCTCCTGGACCTATTATCGGTCCTTTGAAACTAACAGCAATTACCCCAGAAAGTGTCTGCTAA

>p1_ind4873

ATGCAAATAGACCCCGTTGCATATCATTGTTCCGAGTTGAAGTTCGTGCTTGTACCCGAAGAGTCGGGAAAGTTAT
CCCCGACGGAAATAGGATTCCATTTTCAGACTGGGCGCCGCGTACATGACAGAAGACCCCGCTCTGAGTCAGTGA
TGACGCACCAACGCAGATATCGATGTCTCACGTCTACCTTGTTACTTGCCTTGATCGTCGGCACCCGATATTTGTCA
ACTGGGAATCCTCCTGGACCTATTCTCGGTCCTTTGAAACTAACAGCAATTACCCCAGAAAGTGTCTGCTAA

>p1_ind4838

ATGCAAATAGACCCCGTTGCATATCATTGTTCCGAGTTGAAGTTCGTGCTTGTACCCGAAGAGTCGGGAAAGTTAT
CCCCGACGGAAATAGGATTCCATTTTCAGATTGGGTGCCGCGTACATGACAGAAGACCCCGCTCTGAGTCAGTGA
TGACGCACCAACGCAGATATCGATGTCTCACGTCTACCTTATTACTTGCCTTGATCGTCGGCACCCGATATTTGTCA
ACTGGGAATCCTCCTGGACCTATTATCGGTCCTTTGAAACTAACAGCAATTACCCCAGAAAGTGTCTGCTAA

>p1_ind4605

ATGCAAATAGACCCCGTTGCATATCATTGTTCCGAGTTGAAGTTCGTGCTTGTACCCGAAGAGTCGGGAAAGTTAT
CCCCGACGGAAATAGGATTCCATTTTCAGATTGGGTGCCGCGTACATGACAGAAGACCCCGCTCTGAGTCAGTGA
TGACGCCCCAACGCAGATATCGATGTCTCACGTCTACCTTATTACTTGCCTTGATCGTCGGCACCCGATATTTGTCA
ACTGGGAATCCTCCTGGACCTATTATCGGTCCTTTGAAACTAACAGGAATTACCCCAGAAAGTGTCTGCTAA

>p1_ind4362

ATGCAAATAGACCCTGTTGCATATCATTGTTCCGAGTTGAAGTTCGTGCTTGTACCCGAAGAGTCGGGAAAGTTAT
CCCCGACGGAAATAGGATTCCATTTTCAGATTGGGTGCCGCGTACATGACAGAAGACCCCGCTCTGAGTCAGTGA
TGGCGCACCAACGCAAATACAGATGTCTCACGTCTACCTTAATACTTGCCTTGATCGTCGGCACCCGATATTTGTCA
ACTGGGAATCCTCCTGGACCTATTATCGGTCCTTTGAAACTAACAGCAATTACCCCAGAAAGTGTCTGCTAA

>p1_ind3044

ATGCAAATAGACCCCGTTGCATATCATTGTTCCGAGTTGAAGTTCGTGCTTGTACCCGAAGAGTCGGGAAAGTTAT
CCCCGACGGAAATAGGATTCCATTTTCAGACTGGGCGCCGCGTACATGACAGAAGACCCCGCTCTGAGTCAGTGA
TGACGCACCAACGCAGATATCGATGTCTCACGTCTACCTTATTACTTGCCTTGATCGTCGGCACCCGATATTTGTCA
ACTGGGAATCCTCCTGGACCTATTATCGGTCCTTTGAAACTAACAGCAATTACCCCAGAAAGTGTCTGCTAA

>p1_ind2164

ATGCAAATAGACCCCGTTGCATATCATTGTTCCGAGTTGAAGTTCGTGCTTGTACCCGAAGAGTCGGGAAAGTTAT
CCCCGACGGAAATAGGATTCCATTTTCAGACTGGGCGCCGCGTACATGACAGAAGACCCCGCTCTGAGTCAGTGA
TGACGCACCAACGCAGATATCGATGTCTCACGTCTACCTTATTACTTGCCTTGATCGTCGGCACCCGATATTTGTCA
ACTGGGAATCCTCCTGGACCTATTCTCGGTACTTTGAAACTAACAGCAATTACCCCAGAAAGTGTCTGCTAA

>p1_ind2768

ATGCAAATAGACCCCGTTGCATATCATTGTTCCGAGTTGAAGTTCGTGCTTGTACCCGAAGAGTCGGGAAAGTTAT
CCCCGACGGAAATAGGATTCCATTTTCAGATTGGGTGCCGCGTACATGACAGAAGACCCCGCTCTGTGTCAGTGAT
GACGCACCTACGCAGATATCGATGTCTCACGTCTACCTTATTACTTGCCTTGATCGTCGGCACCCGATATTTGTCAA
CTGGGAATCCTCCTGGACCTATTATCGGTCCTTTGAAACTAACAGCAATTACCCCAGAAAGTGTCTGCTAA

>p1_ind2505

ATGCAAATAGACCCCGTTGCATATCATTGTTCCGAGTTGAAGTTCGTGCTTGTACCCGAAGAGTCGGGAAAGTTAT
CCCCGACGGAAATAGGATTCCATTTTGAGATTGGGTGCCGCGTACATGACAGAAGACCCCGCTCTGAGTCAGTGA

TGACGCACCAACGCAGATATCGATGTCTCACGTCTACCTTATTACTTGCCTTGATCGTCGGCACCCGATATTTGTCA
ACTGGGAATCCTCCTGGACCTATTATCGGTCCTTTGAAACTAACAGCAATTACCCCAGAAAGTGTCTGCTAA

>p1_ind2476

ATGCAAATAGACCCCGTTGCATATCATTGTTCCAAGTTGAAGTTCGTGCTTGTACCCGAAGAGTCGGGAAAGTTAT
CCCCGACGGAAATAGGATTCCATTTTCAGATTGGGCGCCGCGTACATGACAGAAGACCCCGCTCTGAGTCAGTGA
TGACGCACCAACGCAGATATCGATGTCTCACGTCTACCTTATTACTTGCCTTGATCGTCGGCACCCGATATTTGTCA
ACTGGGAATCCTCCTGGACCTATTATCGGTCCTTTGAAACTAACAGCAATTACCCCAGAAAGTGTCTGCTAA

>p1_ind4165

ATGCAAATAGACCCCGTTGCATATCATTGTTCCGAGTTGAAGTTCGTGCTTGTACCCGAAGAGTCGGGAAAGTTAT
CCCCGACGGAAATAGGATTCCATTTTGAGATTGGGTGCCGCGTACATGACAGAAGACCCCGCTCTGAGTCAGTGA
TGGCGCACCAACGCAAATATCGATGTCTCACGTCTACCTTATTACTTGCCTTGATCGTCGGCACCCGATATTTGTCA
ACTGGGAATCCTCCTGGACCTATTATCGGTCCTTTGAAACTAACAGCAATTACCCCAGAAAGTGTCTGCTAA

>p1_ind4334

ATGCAAATAGACCCCGTTGCATATCATTGTTCCGAGTTGAAGTTCGTGCTTGTACCCGAAGAGTCGGGAAAGTTAT
CCCCGACGGAAATAGGATTCCATTTTCAGACTGGGCGCCGCGTACATGACAGAAGACCCCGCTCTGAGTCAGTGA
TGACGCACCAACGCAGATATCGATGTCTCACGTCTACCTTATTACTTGCCTTGATCGTCGGCACCCGATATTTGTCA
ACTGGGAATCCTCCTGGACCTATTATCGGTCCTTTGAAACTAACAGCAATTAACCCAGAAAGTGTCTGCTAA

>p1_ind2841

ATGCAAATAGACCCCGTTGCATATCATTGTTCCGAGTTGAAGTTCGTGCTTGTACCCGAAGAGTCGGGAAAGTTAT
CCCCGACGGAAATAGGATTCCATTTTCAGACTGGGCGCCGCGTACATGACAGAAGACCCCGCTCTGAGTCAGTGA
TGACGCACCAACGCAGATATCGATGTCTCACGTCTACCTTATTACTTGCCTTGATCGTCGGCACCCGATATTTGTCA
ACTGGGAATCCTCCTGGACCTATTATCGGTCCTTTGAAACTAACAGCAATTACCCCAGAAAGTGTCTGCTAA

>p1_ind5149

ATGCAAATAGACCCCGTTGCATATCATTGTTCCGAGTTGAAGTTCGTGCTTGTACCCGAAGAGTCGGGAAAGTTAT
CCCCGACGGAAATAGGATTCCATTTTGAGATTGGGTGCCGCGTACATGACAGAAGACCCCGCTCTGAGTCAGTGA
TGGCGCACCAACGCAAATATCGATGTCTCACGTCTACCTTATTACTTGCCTTGATCGTCGGCACCCGATATTTGTCA
ACTGGGAATCCTCCTGGACCTATTATCGGTCCTTTGAAACTAACAGCAATTACCCCAGAAAGTGTCTGCTAA

>p1_ind4855

ATGCAAATAGACCCCGTTGCATATCATTGTTCCGAGTTGAAGTTCGTGCTTGTACCCGAAGAGTCGGGAAAGTTAT
CCCCGACGGAAATAGGACTCCATTTTCAGATTGGGCGCCGCGTACATGACAGAAGACCCCGCTCTGAGTCAGTGA
TGACGCACCAACGCAGATATCGATGTCTCACGTCTACCTTATTACTTGCCTTGATCGTCGGCACCCGATATTTGTCA
ACTGGGAATCCTCCTGGACCTATTATCGGTCCTTTGAAACTAACAGCAATTACCCCAGAAAGTGTCTGCTAA

>p1_ind2334

ATGCAAATAGACCCCGTTGCATATCATTGTTCCGAGTTGAAGTTCGTGCTTGTACCCGAAGAGTCGGGAAAGTTAT
CCCCGACGGAAATAGGATTCCATTTTCAGACTGGGCGCCGCGTACATGACAGAAGACCCCGCTCTGAGTCAGTGA
TGACGCACCAACGCAGATATCGATGTCTCACGTCTACCTTATTACTTGCCTTGATCGTCGGCACCCGATATTTGTCA
ACTGGGAATCCTCCTGGACCTATTCTCGGTCCTTTGAAACTAACAGCAATTACCCCAGAAAGTGTCTGCTAA

>p1_ind2610

ATGCAAATAGACCCCGTTGCATATCATTGTTCCGAGTTGAAGTTCGTGCTTGTACCCGAAGAGTCGGGAAAGTTAT
CCCCGACGGAAATAGGATTCCATTTTCAGATTGGGTGCCGCGTACATGACAGAAGACCCCGCTCTGAGTCAGTGA
TGACGCACCAACGCAGATATCGATGTCTCACGTCTACCTTATTACTTGCCTTGATCGTCGGCACCCGATATTTGTCA
ACTGGGAATCCTCCTGGACCTATTATCGGTCCTTTGAAACTAACAGCAATTACCCCAGAAAGTGTCTGCTAA

>p1_ind4816

ATGCAAATAGACCCCGTTGCATATCATTGTTCCGAGTTGAAGTTCGTGCTTGTACCCGAAGAGTCGGGAAAGTTAT
CCCCGACGGAAATAGGATTCCATTTTCAGATTGGGTGCCGCGTACATGACAGAAGACCCCGCTCTGAGTCAGTGA
TGACGCACCAACGCAGATATCGATGTCTCACGTCTACCTTATTACTTGCCTTGATCGTCGGCACCCGATATTTGTCA
ACTGGGAATCCTCCTGGACCTATTATCGGTCCTTTGAAACTAACAGCAATTACCCCAGAAAGTGTCTGCTAA

>p1_ind2617

ATGCAAATAGACCCCGTTGCATATCATTGTTCCGAGTTGAAGTTCGTGCTTGTACCCGAAGAGTCGGGAAAGTTAT
CCCCGACGGAAATAGGATTCCATTTTCAGACTGGGCGCCGCGTACATGACAGAAGACCCCGCTCTGAGTCAGTGA
TGACGCACCAACGCAGATATCGATGTCTCACGTCTACCTTATTACTTGCCTTGATCGTCGGCACCCGATATTTGTCA
ACTGGGAATCCTCCTGGACCTATTATCGGTCCTTTGAAACTAACAGCAATTACCCCAGAAAGTGTCTGCTAA

>p1_ind1139

ATGCAAATAGCCCCCGTTGCATATCATTGTTCCGAGTTGAAGTTCGTGCTTGTACCCGAAGAGTCGGGAAAGTTAT
CCCCGACGGAAATAGGATTCCATTTTCAGATTGGACGCCGCGTACATGACAGAAGACCCCGCTCTGAGTCAGTGA
TGACGCACCAACGCAGATATCGATGTCTCACGTCTACCTTATTACTTGCCTTGATCGTCGGCACCCGATATTTGTCA
ACTGGGAATCCTCCTGGACCTATTATCGGTCCTTTGAAACTAACAGCAATTACCCCAGAAAGTGTCTGCTAA

>p1_ind2500

ATGCAAATAGACCCCGTTGCATATCATTGTTCCGAGTTGAAGTTCGTGCTTGTACCCGAAGAGTCGGGAAAGTTAT
CCCCGACGGAAATAGGATTCCATTTTCAGACTGGGCGCCGCGTACATGACAGAAGACCCCGCTCTGAGTCAGTGA
TGACGCACCAACGCAGACATCGATGTCTCACGTCTACCTTATTACTTGCCTTGATCGTCGGCACCCGATATTTGTCA
ACTGGGAATCCTCCTGGACCTATTATCGGTCCTTTGAAACTAACAGCAATTACCCCAGAAAGTGTCTGCTAA

>p1_ind3049

ATGCAAATAGACCCCGTTGCATATCATTGTTCCGAGTTGAAGTTCGTGCTTGTACCCGAAGAGTCGGGAAAGTTAT
CCCCGACGGAAATAGGATTCCATTTTCAGATTGGGTGCCGCGCACATGACAGAAGACCCCGCTCTGTGTCAGTGAT

GACGCACCTACGCAGATATCGATGTCTCACGTCTACCTTATTACTTGCCTTGATCGTCGGCACCCGATATTTGTCAA
CTGGGAATCCTCCTGGACCTATTATCGGTCCTTTGAAACTAACAGCAATTACCCCAGAAAGTGTCTGCTAA

>p1_ind371

ATGCAAATAGACCCCGTTGCATATCATTGTTCCGAGTTGAAGTTCGTGCTTGTACCCGAAGAGTCGGGAAAGTTAT
CCCCGACGGAAATAGGATTCCATTTTCAGATTGGACGCCGCGTACATGACAGAAGACCCCGCTCTGAGTCAGTGA
TGACGCACCAACGCAGATATCGATGTCTCACGTCTACCTTATTACTTGCCTTGATCGTCGGCACCCGATATTTGTCA
ACTGGGAATCCTCCTGGACCTATTATCGGTCCTTTGAAACTAACAGCAATTACCCCAGAAAGTGTCTGCTAA

>p1_ind3178

ATGCAAATAGACCCCGTTGCATATCATTGTTCCGAGTTGAAGTTCGTGCTTGTACCCGAAGAGTCGGGAAAGTTAT
CCCCGACGGAAATAGGATTCCATTTTCAGATTGGGTGCCGCGTACATGACAGAAGACCCCGCTCTGAGTCAGTGA
TGGCGCACCAACGCAAATATCGATGTCTCACGTCTACCTTATTACTTGCCTTGATCGTCGGCACCCGATATTTGTCA
ACTGGGAATCCTCCTGGACCTATTATCGGTCCTTTGAAACTAACAGCAATTACCCCAGAAAGTGTCTGCTAA

>p1_ind2250

ATGCAAATAGACCCCGTTGCATATCATTGTTCCGAGTTGAAGTTCGTGCTTGTACCCGAAGAGTCGGGAAAGTTAT
CCCCGACGGAAATAGGATTCCATTTTCAGACTGGGCGCCCCGTACATGACAGAAGACCCCGCTCTGAGTCAGTGA
TGACGCACCAACGCAGATATCGATGTCTCACGTCTACCTTATTACTTGCCTTGATCGTCGGCACCCGATATTTGTCA
ACTGGGAATCCTCCTGGACCTATTATCGGTCCTTTGAAACTAACAGCAATTACCCCAGAAAGTGTCTGCTAA

>p1_ind3247

ATGCAAATAGACCCCGTTGCATATCATTGTTCCGAGTTGAAGTTCGTGCTTGTACCCGAAGAGTCGGGAAAGTTAT
CCCCGACGGAAATAGGATTCCATTTTCAGACTGGGCGCCGCGTACATGACAGAAGACCCCGCTCTGAGTCAGTGA
TGGCGCACCAACGCAGATATCGATGTCTCACGTCTACCTTATTACTTGCCTTGATCGTCGGCACCCGATATTTGTCA
ACTGGGAATCCTCCTGGACCTATTATCGGTCCTTTGAAACTAACAGCAATTACCCCAGAAAGTGTCTGCTAA

>p1_ind3786

ATGCAAATAGACCCCGTTGCATATCATTGTTCCGAGTTGAAGTTCGTGCTTGTACCCGAAGAGTCGGGAAAGTTAT
CCCCGACGGAAATAGGATTCCATTTTCAGATTGGGTGCCGCGTACATGACAGAAGACCCCGCTCTGAGTCAGTGA
TGACGCACCAACGCAGATATCGATGTCTCACGTCTACCTTATTACTTGCCTTGATCGTCGGCACCCGATATTTGTCA
ACTGGGAATCCTCCTGGACCTATTATCGGTCCTTTGAAACTAACAGCAATTACCCCAGAAAGTGTCTGCTAA

>p1_ind2077

ATGCAAATAGACCCCGTTGCATATCATTGTTCCGAGTTGAAGTTCGTGCTTGTACCCGAAGAGTCGGGAAAGTTAT
CCCCGACGGAAATAGGATTCCATTTTCAGACTGGGCGCCGCGTACATGACAGAAGACCCCGCTCTGAGTCAGTGA
TGACGCACCAACGCAGATATCGATGTCTCACGTCTACCTTATTACTTGCCTTGATCGTCGGCACCCGATATTTGTCA
ACTGGGAATCCTCCTGGACCTATTATCGGTCCTTTGAAACTAACAGCAATTACCCCAGAAAGTGTCTGCTAA

>p1_ind2362

ATGCAAATAGACCCCGTTGCATATCATTGTTCCGAGTTGAAGTTCGTGCTTGTACCCGAAGAGTCGGGAAAGTTAT
CCCCGACGGAAATAGGATTCCATTTTCAGATTGGGTGCCGCGTACATGACAGAAGACCCCGCTCTGAGTCAGTGA
TGACGCACCAACGCAGATATCGATGTCTCACGTCTACCTTATTACTTGCCTTGATCGTCGGCACCCGATATTTGTCA
ACTGGGAATCCTCCTGGACCTATTATCGGTCCTTTGAAACTAACAGCAATTACTCCAGAAAGTGTCTGCTAA

>p1_ind628

ATGCAAATAGACCCCGTTGCATATCATTGTTCCGAGTTGAAGTTCGTGCTTGTACCCGAAGAGTCGGGAAAGTTAT
CCCCGACGGAAATAGGATTCCATTTTCAGATTGGACGCCGCGTACATGACAGAAGACCCCGCTCTGAGTCAGTGA
TGACGCACCAACGCAGATATCGATGTCTCACGTCTACCTTATTACTTGCCTTGATCGTCGGCACCCGATATTTGTCA
ACTGGGAATCCTCCTGGACCTATTATCGGTCCTTTGAAACTAACAGCAATTACCCCAGAAAGTGTCTGCTAA

>p1_ind4065

ATGCAAATAGACCCTGTTGCATATCATTGTTCCGAGTTGAAGTTCGTGCTTGTACCCGAAGAGTCGGGAAAGTTAT
CCCCGACGGAAATAGGATTACATTTTCAGATTGGGCGCCGCGTACATGACAGAAGACCCCGCTCTGAGTCAGTGA
TGACGCACCAACGCAGATATCGATGTCTCACGTCTACCTTATTACTTGCCTTGATCGTCGGCACCCGATATTTGTCA
ACTGGGAATCCTCCTGGACCTATTATCGGTCCTTTGAAACTAACAGCAATTACCCCAGAAAGTGTCTGCTAA

>p1_ind3512

ATGCAAATAGACCCCGTTGCATATCATTGTTCCGAGTTGAAGTTCGTGCTTGTACCCGAAGAGTCGGGAAAGTTAT
CCCCGACGGAAATAGGATTCCATTTTCAGACTGGGCGCCGCGTACATGACAGAAGACCCCGCTCTGAGTCAGTGA
TGACGCACCAACGCAGATATCGATGTCTCACGTCTACCTTATTACTTGCCTTGATCGTCGGCACCCGATATTTGTCA
ACTGGGAATCCTCCTGGACCTATTCTCGGTCCTTTGAAACTAACAGCAATTACCCCAGAAAGTGTCTGCTAA

>p1_ind1174

ATGCAAATAGACCCCGTTGCATATCATTGTTCCGAGTTGAAGTTCGTGCTTGTACCCGAAGAGTCGGGAAAGTTAT
CCCCGACGGAAATAGGATTCCATTTTCAGATTGGGTGCCGCGCACATGACAGAAGACCCCGCTCTGAGTCAGTGA
TGACGCACCAACGCAGATATCGATGTCTCACGTCTACCTTATTACTTGCCTTGATCGTCGGCACCCGATATTTGTCA
ACTGGGAATCCCCCTGGACCTATTATCGGTCCTTTGAAACTAACAGCAATTACCCCAGAAAGTGTCTGCTAA

>p1_ind695

ATGCAAATAGCCCCCGTTGCATATCATTGTTCCGAGTTGAAGTTCGTGCTTGTACCCGAAGAGTCGGGAAAGTTAT
CCCCGACGGAAATAGGATTCCATTTTCAGATTGGACGCCGCGTACATGACAGAAGACCCCGCTCTGAGTCAGTGA
TGACGCACCAACGCAGATATCGATGTCTCACGTCTACCTTATTACTTGCCTTGATCGTCGGCACCCGATATTTGTCA
ACTGGGAATCCTCCTGGACCTATTATCGGTCCTTTGAAACTAACAGCAATTACCCCAGAAAGTGTCTGCTAA

>p1_ind736

ATGCAAATAGACCCCGTTGCATATCATTGTTCCGAGTTGAAGTTCGTGCTTGTACCCGAAGAGTCGGGAAAGTTAT
CCCCGACGGAAATAGGATTCCATTTTCAGACTGGGCGCCGCGTACATGACAGAAGACCCCGCTCTGAGTCAGTGA

TGACGCACCAACGCAGATATCGATGTCTCACGTCTACCTTATTACTTGCCTTGATCGTCGGCACCCGATATTTGTCA
ACTGGGAATCCTCCTGGAGCTATTATCGGTCCTTTGAAACTAACAGCAATTACCCCAGAAAGTGTCTGCTAA

>p1_ind1701

ATGCAAATAGACCCCGTTGCATATCATTGTTCCGAGTTGAAGTTCGTGCTTGTACCCGAAGAGTCGGGAAAGTTAT
CCCCGACGGAAATAGGATTCCATTTTCAGACTGGGCGCCGCGTACATGACAGAAGACCCCGCTCTGAGTCAGTGA
TGACGCACCAACGCAGATATCGATGTCTCACGTCTACCTTATTACTTGCCTTGATCGTCGGCACCCGATATTTGTCA
ACTGGGAATCCTCCTGGACCTATTATCGGTCCTTTGAAACTAACAGCAATTACCCCAGAAAGTGTCTGCTAA

>p1_ind1610

ATGCAAATAGACCCCGTTGCATATCATTGTTCCGAGTTGAAGTTCGTGCTTGTACCCGAAGAGTCGGGAAAGTTAT
CCCCGACGGAAATAGGATTCCATTTTCAGATTGGGTGCCGCGTACATGACAGAAGACCCCGCTCTGAGTCAGTGA
TGACGCACCAACGCAGATATCGATGTCTCACGTCTACCTTATTACTTGCCTTGATCGTCGGCACCCGATATTTGTCA
ACTGGGAATCCTCCTGGACCTATTATCGGTCCTTTGAAACTAACAGCAATTACCCCAGAAAGTGTCTGCTAA

>p1_ind1629

ATGCAAATAGACCCCGTTGCATATCATTGTTCCGAGTTGAAGTTCGTGCTTGTACCCGAAGAGTCGGGAAAGTTAT
CCCCGACGGAAATAGGATTCCATTTTCAGATTGGGTGCCGCGTACATGACAGCAGACCCCGCTCTGAGTCAGTGAT
GGCGCACCAACGCAAATATCGATGTCTCACGTCTACCTTATTACTTGCCTTGATCGTCGGCACCCGATTTTTGTCAA
CTGGGAATCCTCCTGGACCTATTATCGGTCCTTTGAAACTAACAGCAATTACCCCAGAAAGTGTCTGCTAA

>p1_ind2948

ATGCAAATAGACCCCGTTGCATATCATTGTTCCGAGTTGAAGTTCGTGCTTGTACCCGAAGAGTCGGGAAAGTTAT
CCCCGACGGAAATAGGATTCCATTTTGAGATTGGGTGCCGCGTACATGACAGAAGACCCCGCTCTGAGTCAGTGA
TGGCGCACCAACGCAAATATCGATGTCTCACGTCTACCTTATTACTTGCCTTGATCGTCGGCACCCGATATTTGTCA
ACTGGGAATCCTCCTGGACCTATTATCGGTCCTTTGAAACTAACAGCAATTACCCCAGAAAGTGTCTGCTAA

>p1_ind1404

ATGCAAATAGACCCCGTTGCATATCATTGTTCCGAGTTGAAGTTCGTGCTTGTACCCGAAGAGTCGGGAAAGTTAT
CCCCGACGGAAATAGGATTCCATTTTCAGACTGGGCGCCGCGTACATGACAGAAGACCCCGCTCTGAGTCAGTGA
TGACGCACCAACGCAGATATCGATGTCTCACGTCTACCTTATTACTTGCCTTGATCGTCGGCACCCGATATTTGTCA
ACTGGGAATCCTCCTGGACCTATTATCGGTCCTTTGAAACTAACAGCAATTACCCCAGAAAGTGTCTGCTAA

>p1_ind237

ATGCAAATAGACCCCGTTGCATATCATTGTTCCGAGTTGAAGTTCGTGCTTGTACCCGAAGAGTCGGGAAAGTTAT
CCCCGACGGAAATAGGATTCCATTTTCAGATTGGGCGCCGCGTACATGACAGAAGACCCCGCTCTGAGTCAGTGA
TGACGCACCAACGCAGATATCGATGTCTCACGTCTACCTTATTACTTGCCTTGATCGTCGGCACCCGATATTTGTCA
ACTGGGAATCCTCCTGGACCTATTATCGGTCCTTTGAAACTAACAGCAATTACCCCAGAAAGTGTCTGCTAA

>p1_ind1754

ATGCAAATAGACCCCGTTGCATATCATTGTTCCGAGTTGAAGTTCGTGCTTGTACCCGAAGAGTCGGGAAAGTTAT
CCCCGACGGAAATAGGATTCCATTTTCAGATTGGGTGCCGCGTACATGACAGAAGACCCCGCTCTGAGTCAGTGA
TGACGCACCAACGCAGATATCGATGTCTCACGTCTACCTTATTACTTGCCTTGATCGTCGGCACCCGATATTTGTCA
ACTGGGAATCCTCCTGGACCTATTATCGGTCCTTTGAAACTAACAGCAATTACCCCAGAAAGTGTCTGCTAA

>p1_ind1048

ATGCAAATAGACCCCGTTGCATATCATTGTTCCGAGTTGAAGTTCGTGCTTGTACCCGAAGAGTCGGGAAAGTTAT
CCCCGACGGAAATAGGATTCCATTTTCAGATTGGGTGCCGCGTACATGACAGAAGACCCCGCTCTGAGTCAGTGA
TGACGCACCAACGCAGATATCGATGTCTCACGTCTACCTTATTACTTGCCTTGATCGTCGGCACCCGATATTTGTCA
ACTGGGAATCCTCCTGGACCTATTATCGGTCCTTTGAAACTAACAGCAATTACCCCAGAAAGTGTCTGCTAA

>p1_ind3523

ATGCAAATAGACCCCGTTGCATATCATTGTTCCGAGTTGAAGTTCGTGCTTGTACCCGAAGAGTCGGGAAAGTTAT
CCCCGACGGAAATAGGATTCCATTTTCAGATTGGGTGCCGCGTACATGACAGAAGACCCCGCTCTGAGTCAGTGA
TGACGCACCAACGCAGATATCGATGTCTCACGTCTACCTTATTACTTGCCTTGATCGTCGGCACCCGATATTTGTCA
ACTGGGAATCCTCCTGGACCTATTATCGGTCCTTTGAAACTAACAGCAATTACCCCAGAAAGTGTCTGCTAA

>p1_ind483

ATGCAAATAGACCCCGTTGCATATCATTGTTCCGAGTTGAAGTTCGTGCTTGTACCCGAAGAGTCGGGAAAGTTAT
CCCCGACGGAAATAGGATTCCATTTTCAGACTGGGCGCCGCGTACATGACAGAAGACCCCGCTCTGAGTCAGTGA
TGACGCACCAACGCAGATATCGATGTCTCACGTCTACCTTATTACTTGCCTTGATCGTCGGCACCCGATATTTGTCA
ACTGGGAATCCTCCTGGACCTATTATCGGTCCTTTGAAACTAACAGCAATTACCCCAGAAAGTGTCTGCTAA

>p1_ind1635

ATGCAAATAGACCCCGTTGCATATCATTGTTCCGAGTTGAAGTTCGTGCTTGTACCCGAAGAGTCGGGAAAGTTAT
CCCCGACGGAAATAGGATTCCATTTTCAGATTGGGTGCCGCGTACATGACAGAAGACCCCGCTCTGTGTCAGTGAT
GACGCACCTACGCAGATATCGATGTCTCACGTCTACCTTATTACTTGCCTTGATCGTCGGCACCCGATATTTGTCAA
CTGGGAATCCTCCTGGACCTATTATCGGTCCTTTGAAACTAACAGCAATTACCCCAGAAAGTGTCTGCTAA

>p1_ind2423

ATGCAAATAGACCCCGTTGCATATCATTGTTCAGAGTTGAAGTTCGTGCTTGTACCCGAAGAGTCGGGAAAGTTAT
CCCCGACGGAAATAGGATTCCATTTTCAGATTGGGCGCCGCGTACATGACAGAAGACCCCGCTCTGAGTCAGTGA
TGACGCACCAACGCAGATATCGATGTCTCACGTCTACCTTATTACTTGCCTTGATCGTCGGCACCCGATATTTGTCA
ACTGGGAATCCTCCTGGACCTATTATCGGTCCTTTGAAACTAACAGCAATTACCCCAGAAAGTGTCTGCTGA

>p1_ind1084

ATGCAAATAGACCCCGTTGCATATCATTGATCCGAGTTGAAGTTCGTGCTTGTACCCGAAGAGTCGGGAAAGTTAT
CCCCGACGGAAATAGGATTCCATTTTCAGACTGGGCGCCGCGTACATGACAGAAGACCCCGCTCTGAGTCAGTGA

TGACGCACCAACGCAGATATCGATGTCTCACGTCTACCTTATTACTTGCCTTGATCGTCGGCACCCGATATTTGTCA
ACTGGGAATCCTCCTGGACCTATTATCGGTCCTTTGAAACTAACAGCAATTACCCCAGAAAGTGTCTGCTAA

>p1_ind4624

ATGCAAATAGACCCCGTTGCATATCATTGTTCCGAGTTGAAGTTCGTGCTTGTACCCGAAGAGTCGGGAAAGTTAT
CCCCGACGGAAATAGGATTCCATTTTCAGATTGGGTGCCGCGTACATGACAGAAGACCCCGCTCTGAGTCAGTGA
TGACGCACCAACGCAGATATCGATGTCTCACGTCTACCTTATTACTTGCCTTGATCGTCGGCACCCGATATTTGTCA
ACTGGGAATCCTCCTGGACCTATTATCGGTCCTTTGAAACTAACAGCAATTACCCCAGAAAGTGTCTGCTAA

>p1_ind3979

ATGCAAATAGACCCTGTTGCATATCATTGTTCCGAGTTGAAGTTCGTGCTTGTACCCGAAGAGTCGGGAAAGTTAT
CCCCGACGGAAATAGGATTCCATTTTCAGATTGGGTGCCGCGTACATGACAGAAGACCCCGCTCTGAGTCAGTGA
TGGCGCACCAACGCAGATATCGATGGCTCACGTCTACCTTATTACTTGCCTTGATCGTCGGCACCCGATATTTGTCA
ACTGGGAATCCTCCTGGACCTATTATCGGTCCTTTGAAACTAACAGCAATTACCCCAGAAAGTGTCTGCTAA

>p1_ind75

ATGCAAATAGACCCCGTTGCATATCATTGTTCCGAGTTGAAGTTCGTGCTTGTACCCGAAGAGTCGGGAAAGTTAT
CCCCGACGGAAATAGGATTCCATTTTCAGACTGGGCGCCGCGTACATGACAGAAGACCCCGCTCTGAGTCAGTGA
TGCCGCACCAACGCAGATATCGATGTCTCACGTCTACCTTATTACTTGCCTTGATCGTCGGCACCCGATATTTGTCA
ACTGGGAATCCTCCTGGACCTATTATCGGTCCTTTGAAACTAACAGCAATTACCCCAGAAAGTGTCTGCTAA

>p1_ind741

ATGCAAATAGACCCCGTTGCATATCATTGTTCCGAGTTGAAGTTCGTGCTTGTACCCGAAGAGTCGGGAAAGTTAT
CCCCGACGGAAATAGGATTCCATTTTCAGATTGGGCGCCGCGTACATGACAGAAGACCCCGCTCTGAGTCAGTGA
TGACGCACCAACGCAGATATCGATGTCTCACGTCTACCTTATTACTTGCCTTGATCGTCGGCACCCGATATTTGTCA
ACTGGGAATCCTCCTGGACCTATTATCGGTCCTTTGAAACTAACAGCAATTACCCCAGAAAGTGTCTGCTAA

>p1_ind4421

ATGCAAATAGACCCCGTTGCATATCATTGTTCCGAGTTGAAGTTCGTGCTTGTACCCGAAGAGTCGGGAAAGTTAT
CCCCGACGGAAATAGGATTCCATTTTCAGATTGGGTGCCGCGTACATGACAGAAGACCCCGCTCTGAGTCAGTGA
TGACGCACCAACGCAGATATCGATGTCTCACGTCTACCTTATTACTTGCCTTGATCGTCGGCACCCGATATTTGTCA
ACTGGGAATCCTCCTGGACCTATTATCGGTCCTTTGAAACTAACAGCAATTACCCCAGAAAGTGTCTGCTAA

>p1_ind1542

ATGCAAATAGACCCCGTTGCATATCATTGTTCCGAGTTGAAGTTCGTGCTTGTACCCGAAGAGTCGGGAAAGTTAT
CCCCGACGGAAATAGGATTCCATTTTCAGATTGGGCGCCGCGTACATGACAGAAGACCCCGCTCTGAGTCAGTGA
TGACGCACCAACGCAGATATCGATGTCTCACGTCTACCTTATTACTTGCCTTGATCGTCGGCACCCGATATTTGTCA
ACTGGGAATCCTCCTGGACCTATTATCGGTCCTTTGAAACTAACAGCAATTACCCCAGAAAGTGTCTGCTAA

>p1_ind3140

ATGCAAATAGACCCCGTTGCATATCATTGTTCCGAGTTGAAGTTCGTGCTTGTACCCGAAGAGTCGGGAAAGTTAT
CCCCGACGGAAATAGGATTCCATTTTCAGACTGGGCGCCGCGTACATGACAGAAGACCCCGCTCTGAGTCAGTGA
TGACGCACCAACGCAGATATCGATGTCTCACGTCTACCTTATTACTTGCCTTGATCGTCGGCACCCGATATTTGTCA
ACTGGGAATCCTCCTGGACCTATTATCGGTCCTTTGAAACTAACAGCAATTACCCCAGAAAGTGTCTGCTAA

>p1_ind4973

ATGCAAATAGACCCCGTTGCATATCATTGTTCCGAGTTGAAGTTCGTGCTTGTACCCGAAGAGTCGGGAAAGTTAT
CCCCGACGGAAATAGGATTCCATTTTCAGATTGGGTGCCGCGTACATGACAGAAGACCCCGCTCTGAGTCAGTGA
TGACGCACCAACGCAGATATCGATGTCTCACGTCTACCTTATTACTTGCCTTGATCGTCGGCACCCGATATTTGTCA
ACTGGGAATCCTCCTGGACCTATTATCGGTCCTTTGAAACTAACAGCAATTACCCCAGAAAGTGTCTGCTAA

>p1_ind917

ATGCAAATAGACCCCGTTGCATATCATTGTTCCGAGTTGAAGTTCGTGCTTGTACCCGAAGAGTCGGGAAAGTTAT
CCCCGACGGAAATAGGATTCCATTTTCAGATTGGGTGCCGCGTACATGACAGAAGACCCCGCTCTGAGTCAGTGA
TGACGCACCAACGCAGATATCGATGTCTCACGTCTACCTTATTACTTGCCTTGATCGTCGGCACCCGATATTTGTCA
ACTGGGAATCCTCCTGGACCTATTATCGGTCCTTTGAAACTAACAGCAATTACCCCAGAAAGTGTCTGCTAA

>p1_ind4923

ATGCAAATAGACCCCGTTGCATATCATTGTTCCGAGTTGAAGTTCGTGCTTGTACCCGAAGAGTCGGGAAAGTTAT
CCCCGACGGAAATAGGATTCCATTTTCAGATTGGACGCCGCGTACATGACAGAAGACCCCGCTCTGAGTCAGTGA
TGACGCACCAACGCAGATATCGATGTCTCACGTCTACCTTATTACTTGCCTTGATCGTCGGCACCCGATATTTGTCA
ACTGGGAATCCTCCTGGACCTATTATCGGTCCTTTGAAACTAACAGCAATTACCCCAGAAAGTGTCTGCTAA

>p1_ind4911

ATGCAAATAGACCCCGTTGCATATCATTGTTCCGAGTTGAAGTTCGTGCTTGTACCCGAAGAGTCGGGAAAGTTAT
CCCCGACGGAAATAGGATTCCATTTTCAGACTGGGCGCCGCGTACATGACAGAAGACCCCGCTCTGAGTCAGTGA
TGACGCACCAACGCAGATATCGATGTCTCACGTCTACCTTATTACTTGCCTTGATCGTCGGCACCCGATATTTGTCA
ACTGGGAATCCTCCTGGACCTATTATCGGTCCTTTGAAACTAACAGCAATTACCCCAGAAAGTGTCTGCTAA

>p1_ind4245

ATGCAAATAGACCCCGTTGCATATCATTGTTCCGAGTTGAAGTTCGTGCTTGTACCCGAAGAGTCGGGAAAGTTAT
CCCCGACGGAAATAGGATTCCATTTTCAGATTGGGTGCCGCGTACATGACAGAAGACCCCGCTCTGAGTCAGTGA
TGACGCCCCAACGCAGATATCGATGTCTCACGTCTACCTTATTAATTGCCTTGATCGTCGGCACCCGATATTTGTCA
ACTGGGAATCCTCCTGGACCTATTATCGGTCCTTTGAAACTAACAGGAATTACCCCAGAAAGTGTCTGCTAA

>p1_ind1261

ATGCAAATAGACCCCGTTGCATATCATTGTTCCGAGTTGAAGTTCGTGCTTGTACCCGAAGAGTCGGGAAAGTTAT
CCCCGACGGAAATAGGATTCCATTTTCAGACTGGGCGCCGCGTACATGACAGAAGACCCCGCTCTGAGTCAGTGA

TGACGCACCAACGCAGATATCGATGTCTCACGTCTACCTTATTACTTGCCTTGATCGTCGGCACCCGATATTTGTCA
ACTGGGAATCCTCCTGGACCTATTATCGGTCCTTTGAAACTAACAGCAATTACCCCAGAAAGTGTCTGCTAA

>p1_ind3481

AAGCAAATAGACCCCGTTGCATATCATTGTTCCGAGTTGAAGTTCGTGCTTGTACCCGAAGAGTCGGGAAAGTTAT
CCCCGACGGAAATAGGATTCCATTTTCAGACTGGGCGCCGCGTACATGACAGCAGACCCCGCTCTGAGTCAGTGA
TGACGCACCAACGCAGATATCGATGTCTCACGTCTACCTTATTACTTGCCTTGATCGTCGGCACCCGATATTTGTCA
ACTGGGAATCCTCCTGGACCTATTATCGGTCCTTTGAAACTAACAGCAATTACCCCAGAAAGTGTCTGCTAA

>p1_ind2426

ATGCAAATAGACCCCGTTGCATATCATTGTTCCGAGTTGAAGTTCGTGCTTGTACCCGAAGAGTCGGGAAAGTTAT
CCCCGACGGAAATAGGATTCCATTTTCAGACTGGGCGCCGCGTACATGACAGAAGACCCCGCTCTGAGTCAGTGA
TGACGCACCAACGCAGATATCGATGTCTCACGTCTACCTTATTACTTGCCTTGATCGTCGGCACCCGATATTTGTCA
ACTGGGAATCCTCCTGGACCTATTATCGGTCCTTTGAAACTAACAGCAATTACCCCAGAAAGTGTCTGCTAA

>p1_ind2710

ATGCAAATAGACCCCGTTGCATATCATTGTTCCGAGTTGAAGTTCGTGCTTGTACCCGAAGAGTCGGGAAAGTTAT
CCCCGACGGAAATAGGATTCCATTTTCAGATTGGGCGCCGCGTACATGACAGAAGACCCCGCTCTGAGTCAGTGA
TGGCGCACCAACGTAGATATCGATGTCTCACGTCTACCTTATTACTTGCCTTGATCGTCGGCACCCGATATTTGTCA
ACTGGGAATCCTCCTGGACCTATTATCGGTCCTTTGAAACTAACAGCAATTACCCCAGAAAGTGTCTGCTAA

>p1_ind923

ATGCAAATAGACCCCGTTGCATATCATTGTTCCGAGTTGAAGTTCGTGCTTGTACCCGAAGAGTCGGGAAAGTTAT
CCCCGACGGAAATAGGATTCCATTTTCAGACTGGGCGCCGCGTACATGACAGAAGACCCCGCTCTGAGTCAGTGA
TGACGCACCAACGCAGATATCGATGTCTCACGTCTACCTTATTACTTGCCTTGATCGTCGGCACCCGACATTTGTCA
ACTGGGAATCCTCCTGGACCTATTATCGGTCCTTTGAAACTAACAGCAATTACCCCAGAAAGTGTCTGCTAA

>p1_ind3720

ATGCAAATAGACCCCGTTGCATATCATTGTTCCGAGTTGAAGTTCGTGCTTGTACCCGAAGAGTCGGGAAAGTTAT
CCCCGACGGAAATAGGATTCCATTTTCAGACTGGGCGCCGCGTACATGACAGAAGACCCCGCTCTGAGTCAGTGA
TGACGCACCAACGCAGATATCGATGTCTCACGTCTACCTTATTACTTGCCTTGATCGTCGGCACCCGATATTTGTCA
ACTGGGAATCCTCCTGGACCTATTATCGGTCCTTTGAAACTAACAGCAATTACCCCAGAAAGTGTCTGCCAA

>p1_ind567

ATGCAAATAGACCCCGTTGCATATCATTGTTCCGAGTTGAAGTTCGTGCTTGTACCCGAAGAGTCGGGAAAGTTAT
CCCCGACGGAAATAGGATTCCATTTTCAGATTGGGTGCCGCGTACATGACAGAAGACCCCGCTCTGAGTCAGTGA
TGACGCACCAACGCAGATATCGATGTCTCACGTCTACCTTATTACTTGCCTTGATCGTCGGCACCCGATATTTGTCA
ACTGGGAATCCTCCTGGACCTATTATCGGTCCTTTGAAACTAACAGCAATTACCCCAGAAAGTGTCTGCTAA

>p1_ind3139

ATGCAAATAGACCCCGTTGCATATCATTGTTCCGAGTTGAAGTTCGTGCTTGTACCCGAAGAGTCGGGAAAGTTAT
CCCCGACGGAAATAGGATTCCATTTTCAGATTGGGTGCCGCGTACATGACAGAAGACCCCGCTCTGAGTCAGTGA
TGACGCACCAACGCAGATATCGATGTCTCACGTCTACCTTATTACTTGCCTTGATCGTCGGCACCCGATATTTGTCA
ACTGGGAATCCTCCTGGACCTATTATCGGTCCTTTGAAACTAACAGCAATTACCCCAGAAAGTGTCTGCTAA

>p1_ind1398

ATGCAAATAGACCCCGTTGCATATCATTGTTCCGAGTTGAAGTTCGTGCTTGTACCCGAAGAGTCGGGAAAGTTAT
CCCCGACGGAAATAGGATTCCATTTTGAGATTGGGTGCCGCGTACATGACAGAAGACCCCGCTCTGAGTCAGTGA
TGACGCACCAACGCAGATATCGATGTCTCACGTCTACCTTATTACTTGGCTTGATCGTCGGCACCCGATATTTGTCA
ACTGGGAATCCTCCTGGACCTATTATCGGTCCTTTGAAACTAACAGCAATTACCCCAGAAAGTGTCTGCTAA

>p1_ind2540

ATGCAAATAGACCCCGTTGCATATCATTGTTCCGAGTTGAAGTTCGTGCTTGTACCCGAATAGTCGGGAAAGTTAT
CCCCGACGGAAATAGGATTCCATTTTCAGATTGGGCGCCGCGTACATGACAGAAGACCCCGCTCTGAGTCAGTGA
TGACGCACCAACGCAGATATCGATGTCTCACGTATACCTTATTACTTGCCTTGATCGTCGGCACCCGATATTTGTCA
ACTGGGAATCCTCCTGGACCTATTATCGGTCCTTTGAAACTAACAGCAATTACCCCAGAAAGTGTCTGCTAA

>p1_ind394

ATGCAAATAGACCCCGTTGCATATCATTGTTCCGAGTTGAAGTTCGTGCTTGTACCCGAAGAGTCGGGAAAGTTAT
CCCCGACGGAAATAGGATTCCATTTTCAGATTGGGTGCCGCGTACATGACAGAAGACCCCGCTCTGAGTCAGTGA
TGACGCACCAACGCAGATATCGATGTCTCACGTCTACCTTATTACTTGCCTTGATCGTCGGCACCCGATATTTGTCA
ACTGGGAATCCTCCTGGACCTATTATCGGTCCTTTGAAACTAACAGCAATTACCCCAGAAAGTGTCTGCTAA

>p1_ind2852

ATGCAAATAGACCCCGTTGCATATCATTGTTCCGAGTTGAAGTTCGTGCTTGTACCCGAAGAGTCGGGAAAGTTAT
CCCCGACGGAAATAGGATTTCATTTTCAGACTGGGCGCCGCGTACATGACAGAAGACCCCGCTCTGAGTCAGTGA
TGACGCACCAACGCAGATATCGATGTCTCACGTCTACCTAATTACTTGCCTTGATCGTCGGCACCCGATATTTGTCA
ACTGGGAATCCTCCTGGACCTATTATCGGTCCTTTGAAACTAACAGCAATTACCCCAGAAAGTGTCTGCTAA

>p1_ind2400

ATGCAAATAGACCCCGTTGCATATCATTGTTCCGAGTTGAAGTTCGTGCTTGTACCCGAAGAGTCGGGAAAGTTAT
CCCCGACGGAAATAGGATTCCATTTTCAGATTGGGTGCCGCGTACATGACAGAAGACCCCGCTCTGAGTCAGTGA
TGACGCCCCAACGCAGATATCGATGTCTCACGTCTACCTTATTACTTGCCTTGATCGTCGGCACCCGATATTTGTCA
ACTGGGAATCCTCCTGGACCTATTATCGGTCCTTTGAAACTAACAGGAATTACCCCAGAAAGTGTCTGCTAA

>p1_ind52

AAGCAAATAGACCCCGTTGCATATCATTGTTCCGAGTTGAAGTTCGTGCTTGTACCCGAAGAGTCGGGAAAGTTAT
CCCCGACGGAAATAGGATTCCATTTTCAGACTGGGCGCCGCGTACATGACAGCAGACCCCGCTCTGAGTCAGTGA

TGACGCACCAACGCAGATATCGATGTCTCACGTCTACCTTATTACTTGCCTTGATCGTCGGCACCCGATATTTGTCA
ACTGGGAATCCTCCTGGACCTATTATCGGTCCTTTGAAACTAACAGCAATTACCCCAGAAAGTGTCTGCTAA

>p1_ind55

ATGCAAATAGACCCCGTTGCATATCATTGTTCCGAGTTGAAGTTCGTGCTTGTACCCGAAGAGTCGGGAAAGTTAT
CCCCGACGGAAATAGGATTCCATTTTCAGATTGGGCGCCGCGTACATGACAGAAGACCCCGCTCTGAGTCAGTGA
TGACGCACCAACGCAGATATCGATGTCTCACGTCTACCTTATTACTTGCCTTGATCGTCGGCACCCGATATTTGTCA
ACTGGGAATCCTCCTGGACCTATTATCGGTCCTTTGAAACTAACAGCAATTACCCCAGAAAGTGTCTGCTAA

>p1_ind1875

ATGCAAATAGACCCTGTTGCATATCATTGTTCCGAGTTGAAGTTCGTGCTTGTACCCGAAGAGTCGGGAAAGTTAT
CCCCGACGGAAATAGGATTCCATTTTCAGATTGGGTGCCGCGTACATGACAGAAGACCCCGCTCTGAGTCAGTGA
TGGCGCACCAACGCAGATATCGATGTCTCACGTCTACCTTATTACTTGCCTTGATCGTCGGCACCCGATATTTGTCA
ACTGGGAATCCTCCTGGACCTATTATCGGTCCTTTGAAACTAACAGCAATTACCCCAGAAAGTGTCTGCTAA

>p1_ind3327

ATGCAAATAGACCCCGTTGCATATCATTGTTCCGAGTTGAAGTTCGTGCTTGTACCCGAAGAGTCGGGAAAGTTAT
CCCCGACGGAAATAGGATTCCATTTTCAGACTGGGCGCCGCGTACATGACAGAAGACCCCGCTCTGAGTCAGTGA
TGACGCACCAACGCAGATATCGATGTCTCACGTCTACCTAATTACTTGCCTTGATCGTCGGCACCCGATATTTGTCA
ACTGGGAATCCTCCTGGACCTATTATCGGTCCTTTGAAACTAACAGCAATTACCCCAGAAAGTGTCTGCTAA

>p1_ind3660

AAGCAAATAGACCCCGTTGCATATCATTGTTCCGAGTTGAAGTTCGTGCTTGTACCCGAAGAGTCGGGAAAGTTAT
CCCCGACGGAAATAGGATTCCATTTTCAGACTGGGCGCCGCGTACATGACAGAAGACCCCGCTCTGAGTCAGTGA
TGACGCACCAACGCAGATATCGATGTCTCACGTCTACCTTATTACTTGCCTTGATCGTCGGCACCCGATATTTGTCA
ACTGGGAATCCTCCTGGACCTATTATCGGTCCTTTGAAACTAACAGCAATTACCCCAGAAAGTGTCTGCTAA

>p1_ind4547

ATGCAAATAGACCCCGTTGCATATCATTGTTCCGAGTTGAAGTTCGTGCTTGTACCCGAAGAGTCGGGAAAGTTAT
CCCCGACGGAAATAGGATTCCATTTTCAGATTGGGTGCCGCGTACATGACAGAAGACCCCGCTCTGAGTCAGTGA
TGACGCACCAACGCAGATATCGATGTCTCACGTCTACCTTATTACTTGCCTTGATCGTCGGCACCCGATATTTGTCA
ACTGGGAATCCTCCTGGACCTATTATCGGTCCTTTGAAACTAACAGCAATTACCCCAGAAAGTGTCTGCTAA

>p1_ind96

ATGCAAATAGACCCCGTTGCATATCATTGTTCCGAGTTGAAGTTCGTGCTTGTACCCGAAGAGTCGGGAAAGTTAT
CCCCGACGGAAATAGGATTCCATTTTCAGATTGGGTGCCGCGTACATGACAGAAGACCCCGCTCTGAGTCAGTGA
TGACGCACCAACGCAGATATCGATGTCTCACGTCTACCTTATTACTTGCCTTGATCGTCGGCACCCGATATTTGTCA
ACTGGGAATCCTCCTGGACCTATTATCGGTCCTTTGAAACTAACAGCAATTACCCCAGAAAGTGTCTGCTAA

>p1_ind952

ATGCAAATAGACCCTGTTGCATATCATTGTTCCGAGTTGAAGTTCGTGCTTGTACCCGAAGAGTCGGGAAAGTTAT
CCCCGACGGAAATAGGATTACATTTTCAGATTGGGCGCCGCGTACATGACAGAAGACCCCGCTCTGAGTCAGTGA
TGACGCACCAACGCAGATATCGATGTCTCACGTCTACCTTATTACTTGCCTTGATCGTCGGCACCCGATATTTGTCA
ACTGGGAATCCTCCTGGACCTATTATCGGTCCTTTGAAACTAACAGCAATTACCCCAGAAAGTGTCTGCTAA

>p1_ind885

ATGCAAATAGACCCCGTTGCATATCATTGTTCCGAGTTGAAGTTCGTGCTTGTACCCGAAGAGTCGGGAAAGTTAT
CCCCGACGGAAATAGGATTCCATTTTCAGATTGGGCGCCGCGTACATGACAGAAGACCCCGCTCTGAGTCAGTGA
TGACGCACCAACGCAGATATCGATGTCTCACGTCTACCTTATTACTTGCCTTGATCGTCGGCACCCGATATTTGTCA
ACTGGGAATCCTCCTGGACCTATTATCGGTCCTTTGAAACTAACAGCAATTACCCCAGAAAGTGTCTGCTAA

>p1_ind130

ATGCAAATAGACCCCGTTGCATATCATTGTTCCGAGTTGAAGTTCGTGCTTGTACCCGAAGAGTCGGGAAAGTTAT
CCCCGACGGAAATAGGATTCCATTTTCAGATTGGGTGCCGCGTACATGACAGAAGACCCCGCTCTGAGTCAGTGA
TGACGCACCAACGCAGATATCGATGTCTCACGTCTACCTTATTACTTGCCTTGATCGTCGGCACCCGATATTTGTCA
ACTGGGAATCCTCCTGGACCTATTATCGGTCCTTTGAAACTAACAGCAATTACCCCAGAAAGTGTCTGCTAA

>p1_ind4596

ATGCAAATAGACCCCGTTGCATATCATTGTTCCGAGTTGAAGTTCGTGCTTGTACCCGAAGAGTCGGGAAAGTTAT
CCCCGACGGAAATAGGATTCCATTTTCAGACTGGGCGCCGCGTACATGACAGAAGACCCCGCTCTGAGTCAGTGA
TGCCGCACCAACGCAGATATCGATGTCTCACGTCTACCTTATTACTTGCCTTGATCGTCGGCACCCGATATTTGTCA
ACTGGGAATCCTCCTGGACCTATTATCGGTCCTTTGAAACTAACAGCAATTACCCCAGAAAGTGTCTGCTAA

>p1_ind3917

ATGCAAATAGACCCCGTTGCATATCATTGTTCCGAGTTGAAGTTCGTGCTTGTACCCGAAGAGTCGGGAAAGTTAT
CCCCGACGGAAATAGGATTCCATTTTCAGACTGGGCGCCGCGTACATGACAGAAGACCCCGCTCTGAGTCAGTGA
TGACGCACCAACGCAGATATCGATGTCTCACGTCTACCTTATTACTTGCCTTGATCGTCGGCACCCGATATTTGTCA
ACTGGGAATCCTCCTGGACCTATTCTCGGTCCTTTGAAACTAACAGCAATTACCCCAGAAAGTGTCTGCTAA

>p1_ind4153

ATGCAAATAGACCCCGTTGCATATCATTGTTCCGAGTTGAAGTTCGTGCTTGTACCCGAAGAGTCGGGAAAGTTAT
CCCCGACGGAAATAGGATTCCATTTTCAGATTGGGTGCCGCGTACATGACAGAAGACCCCGCCCTGAGTCAGTGA
TGACGCACCAACGCAGATATCGATGTCTCACGTCTACCTTATTACTTGCCTTGATCGTCGGCACCCGATATTTGTCA
ACTGGGAATCCTCCTGGACCTATTATCGGTCCTTTGAAACTAACAGCAATTACCCCAGAAAGTGTCTGCTAA

>p1_ind2558

ATGCAAATAGACCCCGTTGCATATCATTGTTCCGAGTTGAAGTTCGTGCTTGTACCCGAAGAGTCGGGAAAGTTAT
CCCCGACGGAAATAGGATTCCATTTTCAGACTGGGCGCCGCGCACATGACAGAAGACCCCGCTCTGAGTCAGTGA

TGACGCACCAACGCAGATATCGATGTCTCACGTCTACCTTATTACTTGCCTTGATCGTCGGCACCCGATATTTGTCA
ACTGGGAATCCTCCTGGACCTATTATCGGTCCTTTGAAACTAACAGCAATTACCCCAGAAAGTGTCTGCTAA

>p1_ind1848

ATGCAAATAGACCCCGTTGCATATCATTGTTCCGAGTTGAAGTTCGTGCTTGTACCCGAAGAGTCGGGAAAGTTAT
CCCCGACGGAAATAGGATTCCATTTTCAGACTGGGCGCCGCGTACATGACAGAAGACCCCGCTCTGAGTCAGTGA
TGACGCACCAACGCAGATATCGATGTCTCACGTCTACCTTATTACTTGCCTTGATCGTCGGCACCCGATATTTGTCA
ACTGGGAATCCTCCTGGACCTATTATCGGTCCTTTGAAACTAACAGCAATTACCCCAGAAAGTGTCTGCTAA

>p1_ind2933

ATGCAAATAGACCCCGTTGCATATCATTGTTCCGAGTTGAAGTTCGTGCTTGTACCCGAAGAGTCGGGAAAGTTAT
CCCCGACGGAAATAGGATTCCATTTTCAGACTGGGCGCCGCGTACATGACAGAAGACCCCGCTCTGAGTCAGTGA
TGACGCTCCAACGCAGATATCGATGTCTCACGTCTGCCTTATTACTTGCCTTGATCGTCGGCACCCGATATTTGTCA
ACTGGGAATCCTCCTGGACCTATTATCGGTCCTTTGAAACTAACAGCAATTACCCCAGAAAGTGTCTGCTAA

>p1_ind3664

ATGCAAATAGACCCCGTTGCATATCATTGTTCCGAGTTGAAGTTCGTGCTTGTACCCGCAGAGTCGGGAAAGTTAT
CCCCGACGGAAATAGGATTCCATTTTCAGATTGGGTGCCGCGTACATGACAGAAGACCCCGCTCTGAGTCAGTGA
TGACGCACCAACGCAGATATCGATGTCTCACGTCTACCTTATTACTTGCCTTGATCGTCGGCACCCGATATTTGTCA
ACTGGGAATCCTCCTGGACCTATTATCGGTCCTTTGAAACTAACAGCAATTACCCCAGAAAGTGTCTGCTAA

>p1_ind2921

ATGCAAATAGACCCCGTTGCATATCATTGTTCCGAGTTGAAGTTCGTGCTTGTACCCGAAGAGTCGGGAAAGTTAT
CCCCGACGGAAATAGGATTCCATTTTCAGATTGGACGCCGCGTACATGACAGAAGACCCCGCTCTGAGTCAGTGA
TGACGCACCAACGCAGATATCGATGTCTCACGTCTACCTTATTACTTGCCTTGATCGTCGGCACCCGATATTTGTCA
ACTGGGAATCCTCCTGGACCTATTATCGGTCCTTTGAAACTAACAGCAATTACCCCAGAAAGTGTCTGCTAA

>p1_ind4981

ATGCAAATAGACCCCGTTGCATATCATTGTTCCGAGTTGAAGTTCGTGCTTGTACCCGAAGAGTCGGGAAAGTTAT
CCCCGACGGAAATAGGATTCCATTTTCAGATTGGACGCCGCGTACATGACAGAAGACCCCGCTCTGAGTCAGTGA
TGACGCACCAACGCAGATATCGATGTCTCACGTCTACCTTATTACTTGCCTTGATCGTCGGCACCCGATATTTGTCA
ACTGGGAATCCTCCTGGACCTATTATCGGTCCTTTGAAACTAACAGCAATTACCCCAGAAAGTGTCTGCTAA

>p1_ind2777

ATGCAAATAGACCCCGTTGCATATCATTGTTCCGAGTTGAAGTTCGTGCTTGTACCCGAAGAGTCGGGAAAGTTAT
CCCCGACGGAAATAGGATTCCATTTTCAGACTGGGCGCCGCGTACATGACAGAAGACCCCGCTCTGAGTCAGTGA
TGCCGCACCAACGCAGATATCGATGTCTCACGTCTACCTTATTACTTGCCTTGATCGTCGGCACCCGATATTTGTCA
ACTGGGAATCCTCCTGGACCTATTATCGGTCCTTTGAAACTAACAGCAATTACCCCAGAAAGTGTCTGCTAA

>p1_ind988

ATGCAAATAGACCCCGTTGCATATCATTGTTCCGAGTTGAAGTTCGTGCTTGTACCCGAAGAGTCGGGAAAGTTAT
CCCCGACGGAAATAGGATTCCATTTTCAGATTGGGCGCCGCGTACATGACAGAAGACCCCGCTCTGAGTCAGTGA
TGACGCACCAACGCAGATATCGATGTCTCACGTCTACCTTATTACTTGCCTTGATCGTCGGCACCCGATATTTGTCA
ACTGGGAATCCTCCTGGACCTATTATCGGTCCTTTGAAACTAACAGCAATTACCCCAGAAAGTGTCTGCTAA

>p1_ind1394

ATGCAAATAGACCCCGTTGCATATCATTGTTCCGAGTTGAAGTTCGTGCTTGTACCCGAAGAGTCGGGAAAGTTAT
CCCCGACGGAAATAGGATTCCATTTTCAGATTGGGTGCCGCGCACATGACAGAAGACCCCGCTCTGAGTCAGTGA
TGACGCACCAACGCAGATATCGATGTCTCACGTCTACCTTATTACTTGCCTTGATCGTCGGCACCCGATATTTGTCA
ACTGGGAATCCCCCTGGACCTATTATCGGTCCTTTGAAACTAACAGCAATTACCCCAGAAAGTGTCTGCTAA

>p1_ind709

ATGCAAATAGACCCCGTTGCATATCATTGTTCCAAGTTGAAGTTCGTGCTTGTACCCGAAGAGTCGGGAAAGTTAT
CCCCGACGGAAATAGGATTCCATTTTCAGATTGGGCGCCGCGTACATGACAGAAGACCCCGCTCTGAGTCAGTGA
TGACGCACCAACGCAGATATCGATGTCTCACGTCTACCTTATTACTTGCCTTGATCGTCGGCACCCGATATTTGTCA
ACTGGGAATCCTCCTGGACCTATTATCGGTCCTTTGAAACTAACAGCAATTACCCCAGAAAGTGTCTGCTAA

>p1_ind2819

ATGCAAATAGACCCCGTTGCATATCATTGTTCCGAGTTGAAGTTCGTGCTTGTACCCGAAGAGTCGGGAAAGTTAT
CCCCGACGGAAATAGGATTCCATTTTCAGATTGGGTGCCGCGTACATGACAGAAGACCCCGCTCTGAGTCAGTGA
TGACGCACCAACGCAGATATCGATGTCTCACGTCTACCTTATTACTTGCCTTGATCGTCGGCACCCGATATTTGTCA
ACTGGGAATCCTCCTGGACCTATTATCGGTCCTTTGAAACTAACAGCAATTACCCCAGAAAGTGTCTGCTAA

>p1_ind1416

ATGCAAATAGCCCCCGTTGCATATCATTGTTCCGAGTTGAAGTTCGTGCTTGTACCCGAAGAGTCGGGAAAGTTAT
CCCCGACGGAAATAGGATTCCATTTTCAGATTGGACGCCGCGTACATGACAGAAGACCCCGCTCTGAGTCAGTGA
TGACGCACCAACGCAGATATCGATGTCTCACGTCTACCTTATTACTTGCCTTGATCGTCGGCACCCGATATTTGTCA
ACTGGGAATCCTCCTGGACCTATTATCGGTCCTTTGAAACTAACAGCAATTACCCCAGAAAGTGTCTGCTAA

>p1_ind188

ATGCAAATAGACCCCGTTGCATATCATTGTTCCGAGTTGAAGTTCGTGCTTGTACCCGAAGAGTCGGGAAAGTTAT
CCCCGACGGAAATAGGATTCCATTTTCAGACTGGGCGCCGCGTACATGACAGAAGACCCCGCTCTGAGTCAGTGA
TGACGCACCAACGCAGATATCGATGTCTCACGTCTACCTTATTACTTGCCTTGATCGTCGGCACCCGATATTTGTCA
ACTGGGAATCCTCCTGGACCTATTATCGGTCCTTTGAAACTAACAGCAATTACCCCAGAAAGTGTCTGCTAA

>p1_ind294

ATGCAAATAGACCCCGTTGCATATCATTGTTCCGAGTTGAAGTTCGTGCTTGTACCCGAAGAGTCGGGAAAGTTAT
CCCCGACGGAAATAGGATTCCATTTTCAGATTGGGTGCCGCGTACATGACAGAAGACCCCGCTCTGAGTCAGTGA

TGACGCACCAACGCAGATATCGATGTCTCACGTCTACCTTATTACTTGCCTTGATCGTCGGCACCCGATTTTTGTCA
ACTGGGAATCCTCCTGGACCTATTATCGGTCCTTTGAAACTAACAGCAATTACCCCAGAAAGTGTCTGCTAA

>p1_ind4387

ATGCAAATAGACCCCGTTGCATATCATTGTTCCGAGTTGAAGTTCGTGCTTGTACCCGAAGAGTCGGGAAAGTTAT
CCCCGACGGAAATAGGATTCCATTTTCAGATTGGGCGCCGCGTACATGACAGAAGACCCCGCTCTGAGTCAGTGA
TGACGCACCAACGCAGATATCGATGTCTCACGTCTACCTTATTACTTGCCTTGATCGTCGGCACCCGATATTTGTCA
ACTGGGAATCCTCCTGGACCTATTATCGGTCCTTTGAAACTAACAGCAATTACCCCAGAAAGTGTCTGCTAA

>p1_ind967

ATGCAAATAGACCCCGTTGCATATCATTGTTCCGAGTTGAAGTTCGTGCTTGTACCCGAAGAGTCGGGAAAGTTAT
CCCCGACGGAAATAGGATTCCATTTTCAGATTGGGTGCCGCGTACATGACAGAAGACCCCGCTCTGAGTCAGTGA
TGACGCACCAACGCAGATATCGATGTCTCACGTCTACCTTATTACTTGCCTTGATCGTCGGCACCCGATATTTGTCC
ACTGGGAATCCTCCTGGACCTATTATCGGTCCTTTGAAACTAACAGCAATTACCCCAGAAAGTGTCTGCTAA

>p1_ind1572

ATGCAAATAGACCCTGTTGCATATCATTGTTCCGAGTTGAAGTTCGTGCTTGTACCCGAAGAGTCGGGAAAGTTAT
CCCCGACGGAAATAGGATTACATTTTCAGATTGGGCGCCGCGTACATGACAGAAGACCCCGCTCTGAGTCAGTGA
TGACGCACCAACGCAGATATCGATGTCTCACGTCTACCTTATTACTTGCCTTGATCGTCGGCACCCGATATTTGTCA
ACTGGGAATCCTCCTGGACCTATTATCGGTCCTTTGAAACTAACAGCAATTACCCCAGAAAGTGTCTGCTAA

>p1_ind1417

ATGCAAATAGACCCCGTTGCATATCATTGTTCCGAGTTGAAGTTCGTGCTTGTACCCGAAGAGTCGGGAAAGTTAT
CCCCGACGGAAATAGGATTCCATTTTCAGACTGGGCGCCGCGTACATGACAGAAGACCCCGCTCTGAGTCAGTGA
TGACGCACCAACGCAGATATCGATGTCTCACGTCTACCTTATTACTTGCCTTGATCGTCGGCACCCGATATTTGTCA
ACTGGGAATCCTCCTGGACCTATTATCGGTCCTTTGAAACTAACAGCAATTACCCCAGAAAGTGTCTGCTAA

>p1_ind3500

ATGCAAATAGACCCCGTTGCATATCATTGTTCCGAGTTGAAGTTCGTGCTTGTACCCGAAGAGTCGGGAAAGTTAT
CCCCGACGGAAATAGGATTCCATTTTCAGACTGGGCGCCGCGTACATGACAGAAGACCCCGCTCTGAGTCAGTGA
TGACGCACCAACGCAGATATCGATGTCTCACGTCTACCTTATTACTTGCCTTGATCGTCGGCACCCGATATTTGTCA
ACTGGGAATCCTCCTGGACCTATTCTCGGTCCTTTGAAACTAACAGCAATTACCCCAGAAAGTGTCTGCTAA

>p1_ind456

ATGCAAATAGACCCCGTTGCATATCATTGTTCCGAGTTGAAGTTCGTGCTTGTACCCGAAGAGTCGGGAAAGTTAT
CCCCGACGGAAATAGGATTCCATTTTCAGATTGGGTGCCGCGTACATGACAGAAGACCCCGCTCTGAGTCAGTGA
TGACGCACCAACGCAGATATCGATGTCTCACGTCTACCTTATTACTTGCCTTGATCGTCGGCACCCGATATTTGTCA
ACTGGGAATCCTCCTGGACCTATTATCGGTCCTTTGAAACTAACAGCAATTACCCCAGAAAGTGTCTGCTAA

>p1_ind6

ATGCAAATAGACCCCGTTGCATATCATTGTTCCGAGTTGAAGTTCGTGCTTGTACCCGAAGAGTCGGGAAAGTTAT
CCCCGACGGAAATAGGATTCCATTTTCAGATTGGGTGCCGCGTACATGACAGAAGACCCCGCTCTGAGTCAGTGA
TGACGCACCAACGCAGATATCGATGTCTCACGTCTACCTTATTACTTGCCTTGATCGTCGGCACCCGATATTTGTCA
ACTGGGAATCCTCCTGGACCTATTATCGGTCCTTTGAAACTAACAGCAATTACCCCAGAAAGTGTCTGCTAA

>p1_ind944

ATGCAAATAGACCCCGTTGCATATCATTGTTCCGAGTTGAAGTTCGTGCTTGTACCCGAAGAGTCGGGAAAGTTAT
CCCCGACGGAAATAGGATTCCATTTTCAGATTGGGCGCCGCGTACATGACAGAAGACCCCGCTCTGAGTCAGTGA
TGACGCACCAACGCAGATATCGATGTCTCACGTCTACCTTATTACTTGCCTTGATCGTCGGCACCCGATATTTGTCA
ACTGGGAATCCTCCTGGACCTATTATCGGTCCTTTGAAACTAACAGCAATTACCCCAGAAAGTGTCTGCTAA

>p1_ind1805

ATGCAAATAGACCCCGTTGCATATCATTGTTCCGAGTTGAAGTTCGTGCTTGTACCCGAAGAGTCGGGAAAGTTAT
CCCCGACGGAAATAGGATTCCATTTTCAGATTGGGTGCCGCGTACATGACAGAAGACCCCGCTCTGAGTCAGTGA
TGACGCACCAACGCAGATATCGATGTCTCACGTCTACCTTATTACTTGCCTTGATCGTCGGCACCCGATATTTGTCA
ACTGGGAATCCTCCTGGACCTATTATCGGTCCTTTGAAACTAACAGCAATTACTCCAGAAAGTGTCTGCTAA

>p1_ind2420

ATGCAAATAGACCCCGTTGCATATCATTGTTCCGAGTTGAAGTTCGTGCTTGTACCCGAAGAGTCGGGAAAGTTAT
CCCCGACGGAAATAGGATTCCATTTTCAGACTGGGCGCCGCGTACATGACAGAAGACCCCGCTCTGAGTCAGTGA
TGACGCACCAACGCAGATATCGATGTCTCACGTCTACCTTATTACTTGCCTTGATCGTCGGCACCCGATATTTGTCA
ACTGGGAATCCTCCTGGACCTATTATCCGTCCTTTGAAACTAACAGCAATTACCCCAGAAAGTGTCTGCTAA

>p1_ind4728

ATGCAAATAGACCCCGTTGCATATCATTGTTCCGAGTTGAAGTTCGTGCTTGTACCCGAAGAGTCGGGAAAGTTAT
CCCCGACGGAAATAGGATTCCATTTTCAGACTGGGCGCCGCGTACATGACAGAAGACCCCGCTCTGAGTCAGTGA
TGACGCACCAACGCAGATATCGATGTCTCACGTCTGCCTTATTACTTGCCTTGATCGTCGGCACCCGATATTTGTCA
ACTGGGAATCCTCCTGGACCTATTATCGGTCCTTTGAAACTAACAGCAATTACCCCAGAAAGTGTCTGCTAA

>p1_ind368

ATGCAAATAGACCCCGTTGCATATCATTGATCCGAGTTGAAGTTCGTGCTTGTACCCGAAGAGTCGGGAAAGTTAT
CCCCGACGGAAATAGGATTCCATTTTCAGACTGGGCGCCGCGTACATGACAGAAGACCCCGCTCTGAGTCAGTGA
TGACGCACCAACGCAGATATCGATGTCTCACGTCTACCTTATTACTTGCCTTGATCGTCGGCACCCGATATTTGTCA
ACTGGGAATCCTCCTGGACCTATTATCGGTCCTTTGAAACTAACAGCAATTACCCCAGAAAGTGTCTGCTAA

>p1_ind2595

ATGCAAATAGACCCCGTTGCATATCATTGTTCCGAGTTGAAGTTCGTGCTTGTACCCGAAGAGTCGGGAAAGTTAT
CCCCGACGGAAATAGGATTCCATTTTCAGATTGGGTGCCGCGTACATGACAGAAGACCCCGCTCTGTGTCAGTGAT

GACGCACCTACGCAGATATCGATGTCTCACGTCTACCTTATTACTTGCCTTGATCGTCGGCACCCGATATTTGTCAA
CTGGGAATCCTCCTGGACCTATTATCGGTCCTTTGAAACTAACAGCAATTACCCCAGAAAGTGTCTGCTAA

>p1_ind2887

ATGCAAATAGACCCCGTTGCATATCATTGTTCCGAGTTGAAGTTCGTGCTTGTACCCGAAGAGTCGGGAAAGTTAT
CCCCGACGGAAATAGGATTCCATTTTCAGATTGGGTGCCGCGTACATGACAGAAGACCCCGCTCTGAGTCAGTGA
TGACGCACCAACGCAGATATCGATGTCTCACGTCTACCTTATTACTTGCCTTGATCGTCGGCACCCGATATTTGTCA
ACTGGGAATCCTCCTGGACCTATTATCGGTCCTTTGAAACTAACAGCAATTACCCCAGAAAGTGTCTGCTAA

>p1_ind4257

ATGCAAATAGACCCCGTTGCATATCATTGTTCCGAGCAGAAGTTCGTGCTTGTACCCGAAGAGTCGGGAAAGTTAT
CCCCGACGGAAATAGGATTCCATTTTCAGATTGGGTGCCGCGTACATGACAGAAGACCCCGCTCTGAATCAGTGAT
GACGCACCAACGCAGATATCGATGTCTCACGTCTACCTTATTACTTGCCTTGATCGTCGGCACCCGATATTTGTCAA
CTGGGAATCCTCCTGGACCTATTATCGGTCCTTTTAAACTAACAGCAATTACCCCAGAAAGTGTCTGCTAA

>p1_ind1880

ATGCAAATAGACCCCGTTGCATATCATTGTTCCGAGTTGAAGTTCGTGCTTGTACCCGAAGAGTCGGGAAAGTTAT
CCCCGACGGAAATAGGATTCCATTTTCAGACTGGGCGCCGCGTACATGACAGAAGACCCCGCTCTGAGTCAGTGA
TGACGCACCAACGCAGATATCGATGTCTCACGTCTACCTTATTACTTGCCTTGATCGTCGGCACCCGATATTTGTCA
ACTGGGAATCCTCCTGGACCTATTATCGGTCCTTTGAAACTAACAGCAATTACCCCAGAAAGTGTCTGCTAA

>p1_ind2704

ATGCAAATAGACCCCGTTGCATATCATTGTTCCGAGTTGAAGTTCGTGCTTGTACCCGAAGAGTCGGGAAAGTTAT
CCCCGACGGAAATAGGATTCCATTTTCAGATTGGGCGCCGCGTACATGACAGAAGACCCCGCTCTGAGTCAGTGA
TGACGCACCAACGCAGATATCGATGTCTCACGTCTACCTTATTACTTGCCTTGATCGTCGGCACCCGATATTTGTCA
ACTGGGAATCCTCCTGGACCTATTATCGGTCCTTTGAAACTAACAGCAATTACCCCAGAAAGTGTCTGCTAA

>p1_ind2081

ATGCAAATAGACCCCGTTGCATATCATTGATCCGAGTTGAAGTTCGTGCTTGTACCCGAAGAGTCGGGAAAGTTAT
CCCCGACGGAAATAGGATTCCATTTTCAGATTGGGTGCCGCGTACATGACAGAAGACCCCGTTCTGAGTCAGTGAT
GACGCACCAACGCAGATATCGATGTCTCACGTCTACCTTATTACTTGCCTTGATCGTCGGCACCCGATATTTGTCAA
CTGGGAATCCTCCTGGACCTATTATCGGTCCTTTGAAACTAACAGCAATTACCCCAGAAAGTGTCTGCTAA

>p1_ind842

ATGCAAATAGACCCCGTTGCATATCATTGTTCCGAGTTGAAGTTCGTGCTTGTACCCGAAGAGTCGGGAAAGTTAT
CCCCGACGGAAATAGGATTCCATTTTCAGATTGGGTGCCGCGTACATGACAGAAGACCCCGCTCTGAGTCAGTGA
TGACGCACCAACGCAGATATCGATGTCTCACGTCTACCTTATTACTTGCCTTGATCGTCGGCACCCGATATTTGTCA
ACTGGGAATCCTCCTGGACCTATTATCGGTCCTTTGAAACTAACAGCAATTACCCCAGAAAGTGTCTGCTAA

>p1_ind1646

ATGCAAATAGACCCCGTTGCATATCATTGATCCGAGTTGAAGTTCGTGCTTGTACCCGAAGAGTCGGGAAAGTTAT
CCCCGACGGAAATAGGATTCCATTTTCAGACTGGGCGCCGCGTACATGACAGAAGACCCCGCTCTGAGTCAGTGA
TGACGCACCAACGCAGATATCGATGTCTCACGTCTACCTTATTACTTGCCTTGATCGTCGGCACCCGATATTTGTCA
ACTGGGAATCCTCCTGGACCTATTATCGGTCCTTTGAAACTAACAGCAATTACCCCAGAAAGTGTCTGCTAA

>p1_ind384

ATGCAAATAGACCCCGTTGCATATCATTGTTCCGAGTTGAAGTTCGTGCTTGTACCCGAAGAGTCGGGAAAGTTAT
CCCCGACGGAAATAGGATTCCATTTTCAGACTGGGCGCCGCGTACATGACAGAAGACCCCGCTCTGAGTCAGTGA
TGACGCACCAACGCAGATATCGATGTCTCACGTCTACCTTATTACTTGCCTTGATCGTCGGCACCCGATATTTGTCA
ACTGGGAATCCTCCTGGACCTATTATCGGTCCTTTGAAACTAACAGCAATTACCCCAGAAAGTGTCTGCTAA

>p1_ind1565

ATGCAAATAGACCCCGTTGCATATCATTGTTCCGAGTTGAAGTTCGTGCTTGTACCCGAAGAGTCGGGAAAGTTAT
CCCCGACGGAAATAGGATTCCATTTTCAGACTGGGCGCCGCGTACATGACAGAAGACCCCGCTCTGAGTCAGTGA
TGACGCACCAACGCAGATATCGATGTCTCACGTCTACCTTATTACTTGCCTTGATCGTCGGCACCCGATATTTGTCA
ACTGGGAATCCTCCTGGACCTATTATCGGTCCTTTGAAACTAACAGCAATTACCCCAGAAAGTGTCTGCTAA

>p1_ind4737

ATGCAAATAGACCCCGTTGCATATCATTGTTCCGAGTTGAAGTTCGTGCTTGTACCCGAAGAGTCGGGAAAGTTAT
CCCCGACGGAAATAGGATTCCATTTTCAGATTGGGTGCCGCGTACATGACAGAAGACCCCGCTCTGAGTCAGTGA
TGACGCACCAACGCAGATATCGATGTCTCACGTCTACCTTATTACTTGCCTTGATCGTCGGCACCCGATATTTGTCA
ACTGGGAATCCTCCTGGACCTATTATCGGTCCTTTGAAACTAACAGCAATTACCCCAGAAAGTGTCTGCTAA

>p1_ind3338

ATGCAAATAGACCCCGTTGCATATCATTGTTCCGAGTTGAAGTTCGTGCTTGTACCCGAAGAGTCGGGAAAGTTAT
CCCCGACGGAAATAGGATTCCATTTTCAGATTGGGTGCCGCGTACATGACAGAAGACCCCGCTCTGAGTCAGTGA
TGACGCACCAACGCAGATATCGATGTCTCACGTCTACCTTATTACTTGCCTTGATCGTCGGCACCCGATATTTGTCA
ACTGGGAATCCTCCTGGACCTATTCTCGGTCCTTTGAAACTAACAGCAATTACCCCAGAAAGTGTCTGCTAA

>p1_ind1435

ATGCAAATAGACCCCGTTGCATATCATTGTTCCGAGTTGAAGTTCGTGCTTGTACCCGAAGAGTCGGGAAAGTTAT
CCCCGACGGAAATAGGATTCCATTTTCAGACTGGGCGCCGCGTACATGACAGAAGACCCCGCTCTGAGTCAGTGA
TGACGCACCAACGCAGATATCGATGTCTCACGTCTACCTTATTACTTGCCTTGATCGTCGGCACCCGATATTTGTCA
ACTGGGAATCCTCCTGGACCTATTATCGGTCCTTTGAAACTAACAGCAATTACCCCAGAAAGTGTCTGCTAA

>p1_ind4429

ATGCAAATAGACCCCGTTGCATATCATTGTTCCGAGTTGAAGTTCGTGCTTGTACCCGAAGAGTCGGGAAAGTTAT
CCCCGACGGAAATAGGATTCCATTTTCAGACTGGGCGCCGCGTACATGACAGAAGACCCCGCTCTGAGTCAGTGA

TGACGCACCAACGCAGATATCGATGTCTCACGTCTACCTTATTACTTGCCTTGATCGTCGGCACCCGATATTTGTCA
ACTGGGAATCCTCCTGGACCTATTATCGGTCCTTTGAAACTAACAGCAATTACCCCAGAAAGTGTCTGCTAA

>p1_ind1010

ATGCAAATAGACCCCGTTGCATATCATTGTTCCGAGTTGAAGTTCGTGCTTGTACCCGAAGAGTCGGGAAAGTTAT
CCCCGACGGAAATAGGATTCCATTTTCAGACTGGGCGCCGCGTACATGACAGAAGACCCCGCTCTGAGTCAGTGA
TGACGCACCAACGCAGATATCGATGTCTCACGTCTACCTTATTACTTGCCTTGATCGTCGGCACCCGATATTTGTCA
ACTGGGAATCCTCCTGGACCTATTATCGGTCCTTTGAAACTAACAGCAATTACCCCAGAAAGTGTCTGCTAA

>p1_ind2514

AAGCAAATAGACCCCGTTGCATATCATTGTTCCGAGTTGAAGTTCGTGCTTGTACCCGAAGAGTCGGGAAAGTTAT
CCCCGACGGAAATAGGATTCCATTTTCAGACTGGGCGCCGCGTACATGACAGCAGACCCCGCTCTGAGTCAGTGA
TGACGCACCAACGCAGATATCGATGTCTCACGTCTACCTTATTACTTGCCTTGATCGTCGGCACCCGATATTTGTCA
ACTGGGAATCCTCCTGGACCTATTATCGGTCCTTTGAAACTAACAGCAATTACCCCAGAAAGTGTCTGCTAA

>p1_ind1449

ATGCAAATAGACCCTGTTGCATATCATTGTTCCGAGTTGAAGTTCGTGCTTGTACCCGAAGAGTCGGGAAAGTTAT
CCCCGACGGAAATAGGATTACATTTTCAGATTGGGCGCCGCGTACATGACAGAAGACCCCGCTCTGAGTCAGTGA
TGACGCACCAACGCAGATATCGATGTCTCACGTCTACCTTATTACTTGCCTTGATCGTCGGCACCCGATATTTGTCA
ACTGGGAATCCTCCTGGACCTATTATCGGTCCTTTGAAACTAACAGCAATTACCCCAGAAAGTGTCTGCTAA

>p1_ind1657

ATGCAAATAGACCCCGTTGCATATCATTGTTCAGAGTTGAAGTTCGTGCTTGTACCCGAAGAGTCGGGAAAGTTAT
CCCCGACGGAAATAGGATTCCATTTTCAGATTGGGCGCCGCGTACATGACAGAAGACCCCGCTCTGAGTCAGTGA
TGACGCACCAACGCAGATATCGATGTCTCACGTCTACCTTATTACTTGCCTTGATCGTCGGCACCCGATATTTGTCA
ACTGGGAATCCTCCTGGACCTATTATCGGTCCTTTGAAACTAACAGCAATTACCCCAGAAAGTGTCTGCTGA

>p1_ind3647

ATGCAAATAGACCCTGTTGCATATCATTGTTCCGAGTTGAAGTTCGTGCTTGTACCCGAAGAGTCGGGAAAGTTAT
CCCCGACGGAAATAGGATTCCATTTTCAGATTGGGTGCCGCGTACATGACAGAAGACCCCGCTCTGAGTCAGTGA
TGGCGCACCAACGCAAATATCGATGTCTCACGTCTACCTTATTACTTGCCTTGATCGTCGGCACCCGATATTTGTCA
ACTGGGAATCCTCCTGGACCTATTATCGGTCCTTTGAAACTAACAGCAATTACCCCAGAAAGTGTCTGCTAA

>p1_ind23

ATGCAAATAGACCCCGTTGCATATCATTGTTCCGAGTTGAAGTTCGTGCTTGTACCCGAAGAGTCGGGAAAGTTAT
CCCCGACGGAAATAGGATTCCATTTTCAGATTGGGTGCCGCGTACATGACAGAAGACCCCGCTCTGAGTCAGTGA
TGACGCACCAACGCAGATATCGATGTCTCACGTCTACCTTATTACTTGCCTTGATCGTCGGCACCCGATATTTGTCA
ACTGGGAATCCTCCTGGACCTATTATCGGTCCTTTGAAACTAACAGCAATTACCCCAGAAAGTGTCTGCTAA

>p1_ind1588

ATGCAAATAGACCCCGTTGCATATCATTGTTCCGAGTTGAAGTTCGTGCTTGTACCCGAAGAGTCGGGAAAGTTAT
CCCCGACGGAAATAGGATTCCATTTTCAGACTGGGCGCCGCGTACATGACAGAAGACCCCGCTCTGAGTCAGTGA
TGACGCACCAACGCAGATATCGATGTCTCACGTCTACCTTATTACTTGCCTTGATCGTCGGCACCCGATATTTGTCA
ACTGGGAATCCTCCTGGACCTATTATCGGTCCTTTGAAACTAACAGCAATTACCCCAGAAAGTGTCTGCTAA

>p1_ind2743

ATGCAAATAGACCCCGTTGCATATCATTGTTCCGAGTTGAAGTTCGTGCTTGTACCCGAAGAGTCGGGAAAGTTAT
CCCCGACGGAAATAGGATTCCATTTTCAGATTGGGTGCCGCGTACATGACAGAAGACCCCGCTCTGAGTCAGTGA
TGACGCACCAACGCAGATATCGATGTCTCACGTCTACCTTATTACTTGCCTTGATCGTCGGCACCCGATATTTGTCA
ACTGGGAATCCTCCTGGACCTATTATCGGTCCTTTGAAACTAACAGCAATTACCCCAGAAAGTGTCTGCTAA

>p1_ind3491

ATGCAAATAGACCCCGTTGCATATCATTGTTCCGAGTTGAAGTTCGTGCTTGTACCCGAAGAGTCGGGAAAGTTAT
CCCCGACGGAAATAGGATTCCATTTTCAGACTGGGCGCCGCGTACATGACAGAAGACCCCGCTCTGAGTCAGTGA
TGACGCACCAACGCAGATATCGATGTCTCACGTCTACCTTATTACTTGCCTTGATCGTCGGCACCCGATATTTGTCA
ACTGGGAATCCTCCTGGACCTATTATCGGTCCTTTGAAACTAACAGCAATTACCCCAGAAAGTGTCTGCTAA

>p1_ind3037

ATGCAAATAGACCCCGTTGCATATCATTGTTCCGAGTAGAAGTTCGTGCTTGTACCCGAAGAGTCGGGAAAGTTAT
CCCCGACGGAAATAGGATTCCATTTTCAGACTGGGCGCCGCGTACATGACAGAAGACCCCGCTCTGAGTCAGTGA
TGACGCACCAACGCAGATATCGATGTCTCACGTCTACCTTATTACTTGCCTTGATCGTCGGCACCCGATATTTGTCA
ACTGGGAATCCTCCTGGACCTATTATCGGTCCTTTGAAACTAACAGCAATTACCCCAGAAAGTGTCTGCTAA

>p1_ind213

ATGCAAATAGACCCCGTTGCATATCATTGTTCCGAGTTGAAGTTCGTGCTTGTACCCGAAGAGTCGGGAAAGTTAT
CCCCGACGGAAATAGGATTCCATTTTCAGATTGGGTGCCGCGTACATGACAGAAGACCCCGCTCTGAGTCTGTGAT
GACGCACCAACGCAGATATCGATGTCTCACGTCTACCTTATTACTTGCCTTGATCGTCGGCACCCGATATTTGTCAA
CTGGGAATCCTCCTGGACCTATTATCGGTCCTTTGAAACTAACAGCAATTACCCCAGAAAGTGTCTGCTAA

>p1_ind2389

ATGCAAATAGACCCCGTTGCATATCATTGTTCCGAGTTGAAGTTCGTGCTTGTACCCGAAGAGTCGGGAAAGTTAT
CCCCGACGGAAATAGGATTCCATTTTCAGATTGGGTGCCGCGTACATGACAGAAGACCCCGCTCTGAGTCAGTGA
TGACGCACCAACGCAGATATCGATGTCTCACGTCTACCTTATTACTTGCCTTGATCGTCGGCACCCGATATTTGTCA
ACTGGGAATCCTCCTGGACCTATTATCGGTCCTTTGAAACTAACAGCAATTACCCCAGAAAGTGTCTGCTAA

>p1_ind3656

ATGCAAATAGACCCCGTTGCATATCATTGTTCCGAGTTGAAGTTCGTGCTTGTACCCGAAGAGTCGGGAAAGTTAT
CCCCGACGGAAATAGGATTCCATTTTCAGATTGGGTGCCGCGTACATGACAGAAGACCCCGCTCTGAGTCAGTGA

TGACGCACCAACGCAGATATCGATGTCTCACGTCTACCTTATTACTTGCCTTGATCGTCGGCACCCGATATTTGTCA
ACTGGGAATCCTCCTGGACCTATTATCGGTCCTTTGAAACTAACAGCAATTACCCCAGAAAGTGTCTGCTAA

>p1_ind3529

ATGCAAATAGACCCCGTTGCATATCATTGTTCCGAGTTGAAGTTCGTGCTTGTACCCGAAGAGTCGGGAAAGTTAT
CCCCGACGGAAATAGGATTCCATTTTCAGATTGGGTGCCGCGTACATGACAGAAGACCCCGCTCTGAGTCAGTGA
TGACGCACCAACGCAGATATCGATGTCTCACGTCTACCTTATTACTTGCCTTGATCGTCGGCACCCGATATTTGTCA
ACTGGGAATCCTCCTGGACCTATTATCGGTCCTTTGAAACTAACAGCAATTACCCCAGAAAGTGTCTGCTAA

>p1_ind4721

ATGCAAATAGACCCCGTTGCATATCATTGTTCCGAGTTGAAGTTCGTGCTTGTACCCGAAGAGTCGGGAAAGTTAT
CCCCGACGGAAATAGGATTCCATTTTCAGATTGGGTGCCGCGTACATGACAGAAGACCCCGCTCTGAGTCAGTGA
TGACGCACCAACGCAGATATCGATGTCTCACGTCTACCTTATTACTTGCCTTGATCGTCGGCACCCGATATTTGTCA
ACTGGGAATCCTCCTGGACCTATTATCGGTCCTTTGAAACTAACAGCAATTACCCCAGAAAGTGTCTGCTAA

>p1_ind5048

ATGCAAATAGACCCCGTTGCATATCATTGTTCCGAGTTGAAGTTCGTGCTTGTACCCGAAGAGTCGGGAAAGTTAT
CCCCGACGGAAATAGGATTCCATTTTCAGACTGGGCGCCGCGTACATGACAGAAGACCCCGCTCTGAGTCAGTGA
TGACGCACCAACGCAGATATCGATGTCTCACGTCTACCTTATTACTTGCCTTGATCGTCGGCACCCGATATTTGTCA
ACTGGGAATCCTCCTGGACCTATTATCGGTCCTTTGAAACTAACAGCAATTACCCCAGAAAGTGTCTGCTAA

>p1_ind2484

ATGCAAATAGACCCCGTTGCATATCATTGTTCCGAGTTGAAGTTCGTGCTTGTACCCGAAGAGTCGGGAAAGTTAT
CCCCGACGGAAATAGGATTCCATTTTCAGATTGGGTGCCGCGTACATGACAGAAGACCCCGCTCTGAGTCAGTGA
TGACGCACCAACGCAGATATCGATGTCTCACGTCTACCTTATTACTTGCCTTGATCGTCGGCACCCGATATTTGTCA
ACTGGGAATCCTCCTGGACCTATTATCGGTCCTTTGAAACTAACAGCAATTACCCCAGAAAGTGTCTGCTAA

>p1_ind1764

ATGCAAATAGACCCCGTTGCATATCATTGTTCCGAGTTGAAGTTCGTGCTTGTACCCGAAGAGTCGAGAAAGTTAT
CCCCGACGGAAATAGGATTCCATTTTCAGATTGGGTGCCGCGTACATGACAGAAGACCCCGCTCTGAGTCAGTGA
TGACGCACCAACGCAGATATCGATGTCTCACGTCTACCTTATTACTTGCCTTGATCGTCGGCACCCGATATTTGTCA
ACTGGGAATCCTCCTGGACCTATTATCGGTCCTTTGAAACTAACAGCAATTACCCCAGAAAGTGTCTGCTAA

>p1_ind3636

ATGCAAATAGACCCCGTTGCATATCATTGTTCCGAGTTGAAGTTCGTGCTTGTACCCGAAGAGTCGGGAAAGTTAT
CCCCGACGGAAATAGGATTCCATTTTCAGATTGGGCGCCGCGTACATGACAGAAGACCCCGCTCTGAGTCAGTGA
TGGCGCACCAACGTAGATATCGATGTCTCACGTCTACCTTATTACTTGCCTTGATCGTCGGCACCCGATATTTGTCA
ACTGGGAATCCTCCTGGACCTATTATCGGTCCTTTGAAACTAACAGCAATTACCCCAGAAAGTGTCTGCTAA

>p1_ind3950

ATGCAAATAGACCCCGTTGCATATCATTGTTCCGAGTTGAAGTTCGTGCTTGTACCCGAAGAGTCGGGAAAGTTAT
CCCCGACGGAAATAGGATTCCATTTTCAGATTGGGTGCCGCGCACATGACAGAAGACCCCGCTCTGTGTCAGTGAT
GACGCACCTACGCAGATATCGATGTCTCACGTCTACCTTATTACTTGCCTTGATCGTCGGCACCCGATATTTGTCAA
CTGGGAATCCTCCTGGACCTATTATCGGTCCTTTGAAACTAACAGCAATTACCCCAGAAAGTGTCTGCTAA

>p1_ind2635

ATGCAAATAGACCCCGTTGCATATCATTGTTCCGAGTTGAAGTTCGTGCTTGTACCCGAAGAGTCGGGAAAGTTAT
CCCCGACGGAAATAGGATTCCATTTTCAGACTGGGCGCCGCGTACATGACAGAAGACCCCGCTCTGAGTCAGTGA
TGACGCACCAACGCAGATATCGATGTCTCACGTCTACCTTATTACTTGCCTTGATCGTCGGCACCCGATATTTGTCA
ACTGGGAATCCTCCTGGACCTATTATCGGTCCTTTGAAACTAACAGCAATTACCCCAGAAAGTGTCTGCTAA

>p1_ind493

ATGCAAATAGACCCCGTTGCATATCATTGTTCCGAGTTGAAGTTCGTGCTTGTACCCGAAGAGTCGGGAAAGTTAT
CCCCGACGGAAATAGGATTCCATTTTCAGACTGGGCGCCGCGTACATGACAGAAGACCCCGCTCTGAGTCAGTGA
TGACGCACCAACGCAGATATCGATGTCTCACGTCTACCTTATTACTTGCCTTGATCGTCGGCACCCGATATTTGTCA
ACTGGGAATCCTCCTGGACCTATTATCGGTCCTTTGAAACTAACAGCAATTACCCCAGAAAGTGTCTGCTAA

>p1_ind3387

ATGCAAATAGACCCCGTTGCATATCATTGTTCCGAGTTGAAGTTCGTGCTTGTACCCGAAGAGTCGGGAAAGTTAT
CCCCGACGGAAATAGGATTCCATTTTCAGATTGGGTGCCGCGTACATGACAGAAGACCCCGCTCTGAGTCAGTGA
TGACGCTCCAACGCAGATATCGATGTCTCACGTCTACCTTATTACTTGCCTTGATCGTCGGCACCCGATATTTGTCA
ACTGGGAATCCTCCTGGACCTATTATCGGTCCTTTGAAACTAACAGCAATTACCCCAGAAAGTGTCTGCTAA

>p1_ind305

ATGCAAATAGACCCCGTTGCATATCATTGTTCCGAGTTGAAGTTCGTGCTTGTACCCGAAGAGTCGGGAAAGTTAT
CCCCGACGGAAATAGGATTCCATTTTCAGACTGGGCGCCGCGTACATGACAGAAGACCCCGCTCTGAGTCAGTGA
TGACGCACCAACGCAGATATCGATGTCTCACGTCTACCTTATTACTTGCCTTGATCGTCGGCACCCGATATTTGTCA
ACTGGGAATCCTCCTGGACCTATTATCGGTCCTTTGAAACTAACAGCAATTACCCCAGAAAGTGTCTGCTAA

>p1_ind3147

ATGCAAATAGACCCCGTTGCATATCATTGTTCCGAGTTGAAGTTCGTGCTTGTACCCGAAGAGTCGGGAAAGTTAT
CCCCGACGGAAATAGGATTCCATTTTCAGATTGGGTGCCGCGCACATGACAGAAGACCCCGCTCTGAGTCAGTGA
TGGCGCACCAACGTAGATATCGATGTCTCACGTCTACCTTATTACTTGCCTTGATCGTCGGCACCCGATATTTGTCA
ATTGGGAATCCTCCTGGACCTATTATCGGTCCTTTGAAACTAACAGCAATTACCCCAGAAAGTGTCTGCTAA

>p1_ind2760

ATGCAAATAGACCCCGTTGCATATCATTGTTCCGAGTTGAAGTTCGTGCTTGTACCCGAAGAGTCGGGAAAGTTAT
CCCCGACGGAAATAGGATTCCATTTTCAGACTGGGCGCCGCGTACATGACAGAAGACCCCGCTCTGAGTCAGTGA

TGACGCACCAACGCAGATATCGATGTCTCACGTCTACCTTATTACTTGCCTTGATCGTCGGCACCCGATATTTGTCA
ACTGGGAATCCTCCTGGACCTATTATCGGTCCTTTGAAACTAACAGCAATTACCCCAGAAAGTGTCTGCTAA

>p1_ind3088

ATGCAAATAGACCCCGTTGCATATCATTGTTCCGAGTTGAAGTTCGTGCTTGTACCCGAAGAGTCGGGAAAGTTAT
CCCCGACGGAAATAGGATTCCATTTTCAGACTGGGCGCCGCGTACATGACAGAAGACCCCGCTCTGAGTCAGTGA
TGACGCACCAACGCAGATATCGATGTCTCACGTCTACCTTATTACTTGCCTTGATCGTCGGCACCCGATATTTGTCA
ACTGGGAATCCTCCTGGACCTATTATCGGTCCTTTGAAACTAACAGCAATTACCCCAGAAAGTGTCTGCTAA

>p1_ind2154

ATGCAAATAGACCCCGTTGCATATCATTGTTCCGAGTTGAAGTTCGTGCTTGTACCCGAAGAGTCGGGAAAGTTAT
CCCCGACGGAAATAGGATTCCATTTTCAGATTGGGCGCCGCGTACATGACAGAAGACCCCGCTCTGAGTCAGTGA
TGACGCACCAACGCAGATATCGATGTCTCACGTCTACCTTATTACTTGCCTTGATCGTCGGCACCCGATATTTGTCA
ACTGGGAATCCTCCTGGACCTATTATCGGTCCTTTGAAACTAACAGCAATTACCCCAGAAAGTGTCTGCTAA

>p1_ind1368

ATGCAAATAGACCCCGTTGCATATCATTGTTCCGAGTTGAAGTTCGTGCTTGTACCCGAAGAGTCGGGAAAGTTAT
CCCCGACGGAAATAGGATTCCATTTTCAGACTGGGCGCCGCGTACATGACAGAAGACCCCGCTCTGAGTCAGTGA
TGACGCACCAACGCAGATATCGATGTCTCACGTCTACCTTATTACTTGCCTTGATCGTCGGCACCCGATATTTGTCA
ACTGGGAATCCTCCTGGACCTATTATCGGTCCTTTGAAACTAACAGCAATTACCCCAGAAAGTGTCTGCTAA

>p1_ind666

ATGCAAATAGACCCCGTTGCATATCATTGTTCCGAGTTGAAGTTCGTGCTTGTACCCGAAGAGTCGGGAAAGTTAT
CCCCGACGGAAATAGGATTCCATTTTCAGACTGGGCGCCGCGTACATGACAGAAGACCCCGCTCTGAGTCAGTGA
TGCCGCACCAACGCAGATATCGATGTCTCACGTCTACCTTATTACTTGCCTTGATCGTCGGCACCCGATATTTGTCA
ACTGGGAATCCTCCTGGACCTATTATCGGTCCTTTGAAACTAACAGCAATTACCCCAGAAAGTGTCTGCTAA

>p1_ind2128

ATGCAAATAGACCCCGTTGCATATCATTGTTCCGAGTTGAAGTTCGTGCTTGTACCCGAAGAGTTGGGAAAGTTAT
CCCCGACGGAAATAGGATTCCATTTTCAGACTGGGCGCCGCGTACATGACAGAAGACCCCGCTCTGAGTCAGTGA
TGACGCACCAACGCAGATATCGATGTCTCACGTCTACCTTATTACTTGCCTTGATCGTCGGCACCCGATATTTGTCA
ACTGGGAATCCTCCTGGACCTATTATCGGTCCTTTGAAACTAACAGCAATTACCCCAGAAAGTGTCTGCTAA

>p1_ind2813

ATGCAAATAGACCCCGTTGCATATCATTGTTCCGAGTTGAAGTTCGTGCTTGTACCCGAAGAGTCGGGAAAGTTAT
CCCCGACGGAAATAGGATTCCATTTTCAGATTGGGTGCCGCGTACATGACAGAAGACCCCGCTCTGAGTCAGTGA
TGACGCCCCAACGCAGATATCGATGTCTCACGTCTACCTTATTAATTGCCTTGATCGTCGGCACCCGATATTTGTCA
ACTGGGAATCCTCCTGGACCTATTATCGGTCCTTTGAAACTAACCGGAATTACCCCAGAAAGTGTCTGCTAA

>p1_ind2511

ATGCAAATAGACCCCGTTGCATATCATTGTTCCGAGTTGAAGTTCGTGCTTGTACCCGAAGAGTCGGGAAAGTTAT
CCCCGACGGAAATAGGATTCCATTTTCAGACTGGGCGCCGCGTACATGACAGAAGACCCCGCTCTGAGTCAGTGA
TGACGCACCAACGCAGATATCGATGTCTCACGTCTACCTTATTACTTGCCTTGATCGTCGGCACCCGATATTTGTCA
ACTGGGAATCCTCCTGGACCTATTATCGGTCCTTTGAAACTAACAGCAATTACCCCAGAAAGTGTCTGCTAA

>p1_ind512

ATGCAAATAGACCCCGTTGCATATCATTGTTCCGAGTTGAAGTTCGTGCTTGTACCCGAAGAGTCGGGAAAGTTAT
CCCCGACGGAAATAGGATTCCATTTTCAGATTGGACGCCGCGTACATGACAGAAGACCCCGCTCTGAGTCAGTGA
TGACGCACCAACGCAGATATCGATGTCTCACGTCTACCTTATTACTTGCCTTGATCGTCGGCACCCGATATTTGTCA
ACTGGGAATCCTCCTGGACCTATTATCGGTCCTTTGAAACTAACAGCAATTACCCCAGAAAGTGTCTGCTAA

>p1_ind4471

ATGCAAATAGACCCCGTTGCATATCATTGTTCCGAGTTGAAGTTCGTGCTTGTACCCGAAGAGTCGGGAAAGTTAT
CCCCGACGGAAATAGGATTCCATTTTCAGACTGGGCGCCGCGTACATGACAGAAGACCCCGCTCTGAGTCAGTGA
TGACGCACCAACGCAGATATCGATGTCTCACGTCAACCTTATTACTTGCCTTGATCGTCGGCACCCGATATTTGTCA
ACTGGGAATCCTCCTGGACCTATTATCGGTCCTTTGAAACTAACAGCAATTACCCCAGAAAGTGTCTGCTAA

>p1_ind4138

ATGCAAATAGACCCCGTTGCATATCATTGTTCCGAGTTGAAGTTCGTGCTTGTACCCGAAGAGTCGGGAAAGTTAT
CCCCGACGGAAATAGGATTCCATTTTCAGATTGGGTGCCGCGTACATGACAGAAGACCCCGTTCTGAGTCAGTGAT
GTCGCACCAACGCAGATATCGATGTCTCACGTCTACCTTATTACTTGCCTTGATCGTCGGCACCCGATATTTGTCAA
CTGGGAATCCTCCTGGACCTATTATCGGTCCTTTGAAACTAACAGCAATTACCCCAGAAAGTGTCTGCTAA

>p1_ind4715

ATGCAAATAGACCCCGTTGCATATCATTGTTCCGAGTTGAAGTTCGTGCTTGTACCCGAAGAGTCGGGAAAGTTAT
CCCCGACGGAAATAGGATTCCATTTTCAGACTGGGCGCCGCGTACATGACAGAAGACCCCGCTCTGAGTCAGTGA
TGACGCACCAACGCAGATATCGATGTCTCACGTCTACCTTATTACTTGCCTTGATCGTCGGCACCCGATATTTGTCA
ACTGGGAATCCTCCTGGACCTATTCTCGGTCCTTTGAAACTAACAGCAATTACCCCAGAAAGTGTCTGCTAA

>p1_ind2385

ATGCAAATAGACCCCGTTGCATATCATTGTTCCGAGTTGAAGTTCGTGCTTGTACCCGAAGAGTCGGGAAAGTTAT
CCCCGACGGAAATAGGATTCCATTTTCAGACTGGGCGCCGCGTACATGACAGAAGACCCCGCTCTGAGTCAGTGA
TGACGCACCAACGCAGATATCGATGTCTCACGTCTACCTTATTACTTGCCTTGATCGTCGGCACCCGATATTTGTCA
ACTGGGAATCCTCCTGGACCTATTCTCGGTCCTTTGAAACTAACAGCAATTACCCCAGAAAGTGTCTGCTAA

>p1_ind2512

ATGCAAATAGACCCCGTTGCATATCATTGTTCCGAGTTGAAGTTCGTGCTTGTACCCGAAGAGTCGGGAAAGTTAT
CCCCGACGGAAATAGGATTCCATTTTCAGATTGGGTGCCGCGTACATGACAGAAGACCCCGCTCTGAGTCAGTGA

TGACGCACCAACGCAGATATCGATGTCTCACGTCTACCTTATTACTTGCCTTGATCGTCGGCACCCGATATTTGTCA
ACTGGGAATCCTCCTGGACCTATTATCGGTCCTTTGAAACTAACAGCAATTACCCCAGAAAGTGTCTGCTAA

>p1_ind3588

ATGCAAATAGACCCCGTTGCATATCATTGTTCCGAGTTGAAGTTCGTGCTTGTACCCGAATAGTCGGGAAAGTTAT
CCCCGACGGAAATAGGATTCCATTTTCAGATTGGGCGCCGCGTACATGACAGAAGACCCCGCTCTGAGTCAGTGA
TGACGCACCAACGCAGATATCGATGTCTCACGTATACCTTATTACTTGCCTTGATCGTCGGCACCCGATATTTGTCA
ACTGGGAATCCTCCTGGACCTATTATCGGTCCTTTGAAACTAACAGCAATTACCCCAGAAAGTGTCTGCTAA

>p1_ind4597

ATGCAAATAGACCCCGTTGCATATCATTGTTCCGAGTTGAAGTTCGTGCTTGTACCCGAAGAGTCGGGAAAGTTAT
CCCCGACGGAAATAGGATTCCATTTTCAGACTGGGCGCCGCGTACATGACAGAAGACCCCGCTCTGAGTCAGTGA
TGACGCACCAACGCAGATATCGATGTCTCACGTCTACCTTATTACTTGCCTTGATCGTCGGCACCCGATATTTGTCA
ACTGGGAATCCTCCTGGACCTATTATCGGTCCTTTGAAACTAACAGCAATTACCCCAGAAAGTGTCTGCTAA

>p1_ind4495

ATGCAAATAGACCCCGTTGCATATCATTGTTCCGAGTTGAAGTTCGTGCTTGTACCCGAAGAGTCGGGAAAGTTAT
CCCCGACGGAAATAGGATTCCATTTTCAGATTGGGTGCCGCGTACATGACAGAAGACCCCGCTCTGAGTCAGTGA
TGACGCACCAACGCAGATATCGATGTCTCACGTCTACCTTATTACTTGCCTTGATCGTCGGCACCCGATATTTGTCA
ACTGGGAATCCTCCTGGACCTATTATCGGTCCTTTGAAACTAACAGCAATTACCCCAGAAAGTGTCTGCTAA

>p1_ind3219

ATGCAAATAGACCCCGTTGCATATCATTGTTCCGAGTTGAAGTTCGTGCTTGTACCCGAAGAGTCGGGAAAGTTAT
CCCCGACGGAAATAGGATTCCATTTTCAGACTGGGCGCCGCGTACATGACAGAAGACCCCGCTCTGAGTCAGTGA
TGACGCACCAACGCAGATATCGATGTCTCACGTCTACCTTATTACTTGCCTTGATCGTCGGCACCCGATATTTGTCA
ACTGGGAATCCTCCTGGACCTATTATCGGTCCTTTGAAACTAACAGCAATTACCCCAGAAAGTGTCTGCTAA

>p1_ind2463

ATGCAAATAGACCCTGTTGCATATCATTGTTCCGAGTTGAAGTTCGTGCTTGTACCCGAAGAGTCGGGAAAGTTAT
CCCCGACGGAAATAGGATTCCATTTTCAGATTGGGTGCCGCGTACATGACAGAAGACCCCGCTCTGAGTCAGTGA
TGGCGCACCAACGCAGATATCGATGGCTCACGTCTACCTTATTACTTGCCTTGATCGTCGGCACCCGATATTTGTCA
ACTGGGAATCCTCCTGGACCTATTATCGGTCCTTTGAAACTAACAGCAATTACCCCAGAAAGTGTCTGCTAA

>p1_ind2647

ATGCAAATAGACCCCGTTGCATATCATTGTTCCGAGTTGAAGTTCGTGCTTGTACCCGAAGAGTCGGGAAAGTTAT
CCCCGACGGAAATAGGATTCCATTTTCAGATTGGGCGCCGCGTACATGACAGAAGACCCCGCTCTGAGTCAGTGA
TGACGCACCAACGCAGATATCGATGTCTCACGTCTACCTTATTACTTGCCTTGATCGTCGGCACCCGATATTTGTCA
ACTGGGAATCCTCCTGGACCTATTATCGGTCCTTTGAAACTAACAGCAATTACCCCAGAAAGTGTCTGCTAA

>p1_ind2298

ATGCAAATAGACCCCGTTGCATATCATTGTTCCGAGTAGAAGTTCGTGCTTGTACCCGAAGAGTCGGGAAAGTTAT
CCCCGACGGAAATAGGATTCCATTTTCAGACTGGGCGCCGCGTACATGACAGAAGACCCCGCTCTGAGTCAGTGA
TGACGCACCTACGCAGATATCGATGTCTCACGTCTACCTTATTACTTGCCTTGATCGTCGGCACCCGATATTTGTCA
ACTGGGAATCCTCCTGGACCTATTATCGGTCCTTTGAAACTAACAGCAATTACCCCAGAAAGTGTCTGCTAA

>p1_ind3024

ATGCAAATAGACCCCGTTGCATATCATTGTTCCGAGTAGAAGTTCGTGCTTGTACCCGAAGAGTCGGGAAAGTTAT
CCCCGACGGAAATAGGATTCCTTTTTCAGACTGGGCGCCGCGTACATGACAGAAGACCCCGCTCTGAGTCAGTGA
TGACGCACCAACGCAGATATCGATGTCTCACGTCTACCTTATTACTTGCCTTGATCGTCGGCACCCGATATTTGTCA
ACTGGGAATCCTCCTGGACCTATTATCGGTCCTTTGAAACTAACAGCAATTACCCCAGAAAGTGTCTGCTAA

>p1_ind2201

ATGCAAATAGACCCCGTTGCATATCATTGTTCCGAGTTGAAGTTCGTGCTTGTACCCGAAGAGTCGGGAAAGTTAT
CCCCGACGGAAATAGGATTCCATTTTCAGATTGGGTGCCGCGTACATGACAGAAGACCCCGCTCTGAGTCAGTGA
TGACGCACCAACGCAGATATCGATGTCTCACGTCTACCTTATTACTTGCCTTGATCGTCGGCACCCGATATTTGTCA
ACTGGGAATCCTCCTGGACCTATTATCGGTCCTTTGAAACTAACAGCAATTACCCCAGAAAGTGTCTGCTAA

>p1_ind4410

ATGCAAATAGACCCCGTTGCATATCATTGTTCCGAGTTGAAGTTCGTGCTTGTACCCGAAGAGTCGGGAAAGTTAT
CCCCGACGGAAATAGGATTCCATTTTCAGACTGGGCGCCGCGTACATGACAGAAGACCCCGCTCTGAGTCAGTGA
TGACGCACCAACGCAGATATCGATGTCTCACGTCTACCTTATTACTTGCCTTGATCGTCGGCACCCGATATTTGTCA
ACTGGGAATCCTCCTGGACCTATTATCGGTCCTTTGAAACTAACAGCAATTACCCCAGAAAGTGTCTGCTAA

>p1_ind2932

ATGCAAATAGACCCTGTTGCATATCATTGTTCCGAGTTGAAGTTCGTGCTTGTACCCGAAGAGTCGGGAAAGTTAT
CCCCGACGGAAATAGGATTACATTTTCAGATTGGGCGCCGCGTACATGACAGAAGACCCCGCTCTGAGTCAGTGA
TGACGCACCAACGCAGATATCGATGTCTCACGTCTACCTTATTACTTGCCTTGATCGTCGGCACCCGATATTTGTCA
ACTGGGAATCCTCCTGGACCTATTATCGGTCCTTTGAAACTAACAGCAATTACCCCAGAAAGTGTCTGCTAA

>p1_ind894

ATGCAAATAGACCCCGTTGCATATCATTGTTCCGAGTTGAAGTTCGTGCTTGTACCCGAAGAGTCGGGAAAGTTAT
CCCCGACGGAAATAGGATTCCATTTTCAGATTGGGCGCCGCGTACATGACAGAAGACCCCGCTCTGAGTCAGTGA
TGACGCACCAACGCAGATATCGATGTCTCACGTCTACCTTATTACTTGCCTTGATCGTCGGCACCCGATATTTGTCA
ACTGGGAATCCTCCTGGACCTATTATCGGTCCTTTGAAACTAACAGCAATTACCCCAGAAAGTGTCTGCTAA

>p1_ind1377

ATGCAAATAGACCCCGTTGCATATCATTGTTCCGAGTTGAAGTTCGTGCTTGTACCCGAAGAGTCGGGAAAGTTAT
CCCCGACGGAAATAGGATTCCATTTTCAGACTGGGCGCCCCGTACATGACAGAAGACCCCGCTCTGAGTCAGTGA

TGACGCACCAACGCAGATATCGATGTCTCACGTCTACCTTATTACTTGCCTTGATCGTCGGCACCCGATATTTGTCA
ACTGGGAATCCTCCTGGACCTATTATCGGTCCTTTGAAACTAACAGCAATTACCCCAGAAAGTGTCTGCTAA

>p1_ind2662

ATGCAAATAGACCCCGTTGCATATCATTGTTCCGAGTTGAAGTTCGTGCTTGTACCCGAAGAGTCGGGAAAGTTAT
CCCCGACGGAAATAGGATTCCATTTTCAGACTGGGCGCCGCGTACATGACAGAAGACCCCGCTCTGAGTCAGTGA
TGACGCACCAACGCAGATATCGATGTCTCACGTCTACCTTATTACTTGCCTTGATCGTCGGCACCCGATATTTGTCA
ACTGGGAATCCTCCTGGACCTATTATCGGTCCTTTGAAACTAACAGCAATTACCCCAGAAAGTGTCTGCTAA

>p1_ind1642

ATGCAAATAGACCCCGTTGCATATCATTGTTCCGAGTTGAAGTTCGTGCTTGTACCCGAAGAGTCGGGAAAGTTAT
CCCCGACGGAAATAGGATTCCATTTTCAGATTGGGTGCCGCGTACATGACAGAAGACCCCGCTCTGAGTCAGTGA
TGACGCACCAACGCAGATATCGATGTCTCACGTCTACCTTATTACTTGCCTTGATCGTCGGCACCCGATATTTGTCA
ACTGGGAATCCTCCTGGACCTATTATCGGTCCTTTGAAACTAACAGCAATTACTCCAGAAAGTGTCTGCTAA

>p1_ind3662

ATGCAAATAGACCCCGTTGCATATCATTGTTCCGAGTTGAAGTTCGTGCTTGTACCCGAAGAGTCGGGAAAGTTAT
CCCCGACGGAAATAGGATTCCATTTTCAGACTGGGCGCCGCGTACATGACAGAAGACCCCGCTCTGAGTCAGTGA
TGACGCACCAACGCAGATATCGATGTCTCACGTCTACCTTATTACTTGCCTTGATCGTCGGCACCCGATATTTGTCA
ACTGGGAATCCTCCTGGACCTATTATCGGTCCTTTGAAACTAACAGCAATTACCCCAGAAAGTGTCTGCTAA

>p1_ind204

ATGCAAATAGACCCCGTTGCATATCATTGTTCCGAGTTGAAGTTCGTGCTTGTACCCGAAGAGTCGGGAAAGTTAT
CCCCGACGGAAATAGGATTCCATTTTCAGATTGGGCGCCGCGTACATGACAGAAGACCCCGCTCTGAGTCAGTGA
TGGCGCACCAACGTAGATATCGATGTCTCACGTCTACCTTATTACTTGCCTTGATCGTCGGCACCCGATATTTGTCA
ACTGGGAATCCTCCTGGACCTATTATCGGTCCTTTGAAACTAACAGCAATTACCCCAGAAAGTGTCTGCTAA

>p1_ind530

ATGCAAATAGACCCCGTTGCATATCATTGTTCCGAGTTGAAGTTCGTGCTTGTACCCGAAGAGTCGGGAAAGTTAT
CCCCGACGGAAATAGGATTCCATTTTCAGACTGGGCGCCGCGTACATGACAGAAGACCCCGCTCTGAGTCAGTGA
TGACGCACCAACGCAGATATCGATGTCTCACGTCAACCTTATTACTTGCCTTGATCGTCGGCACCCGATATTTGTCA
ACTGGGAATCCTCCTGGACCTATTATCGGTCCTTTGAAACTAACAGCAATTACCCCAGAAAGTGTCTGCTAA

>p1_ind4642

ATGCAAATAGACCCCGTTGCATATCATTGTTCCGAGTTGAAGTTCGTGCTTGTACCCGAAGAGTCGGGAAAGTTAT
CCCCGACGGAAATAGGATTCCATTTTCAGACTGGGCGCCGCGTACATGACAGAAGACCCCGCTCTGAGTCAGTGA
TGACGCACCAACGCAGATATCGATGTCTCACGTCTACCTTATTACTTGCCTTGATCGTCGGCACCCGATATTTGTCA
ACTGGGAATCCTCCTGGACCTATTATCGGTCCTTTGAAACTAACAGCAATTACCCCAGAAAGTGTCTGCTAA

>p1_ind1810

ATGCAAATAGACCCCGTTGCATATCATTGTTCCGAGTTGAAGTTCGTGCTTGTACCCGAAGAGTCGGGAAAGTTAT
CCCCGACGGAAATAGGATTCCATTTTCAGACTGGGCGCCGCGTACATGACAGAAGACCCCGCTCTGAGTCAGTGA
TGACGCACCAACGCAGATATCGATGTCTCACGTCTACCTTATTACTTGCCTTGATCGTCGGCACCCGATATTTGTCA
ACTGGGAATCCTCCTGGACCTATTATCGGTCCTTTGAAACTAACAGCAATTACCCCAGAAAGTGTCTGCTAA

>p1_ind2902

ATGCAAATAGACCCCGTTGCATATCATTGTTCCGAGTTGAAGTTCGTGCTTGTACCCGAAGAGTCGGGAAAGTTAT
CCCCGACGGAAATAGGATTCCATTTTCAGACTGGGCGCCGCGTACATGACAGAAGACCCCGCTCTGAGTCAGTGA
TGCCGCACCAACGCAGATATCGATGTCTCACGTCTACCTTATTACTTGCCTTGATCGTCGGCACCCGATATTTGTCA
ACTGGGAATCCTCCTGGACCTATTATCGGTCCTTTGAAACTAACAGCAATTACCCCAGAAAGTGTCTGCTAA

>p1_ind2744

ATGCAAATAGACCCCGTTGCATATCATTGTTCCGAGTTGAAGTTCGTGCTTGTACCCGAAGAGTCGGGAAAGTTAT
CCCCGACGGAAATAGGATTCCATTTTGAGATTGGGTGCCGCGTACATGACAGAAGACCCCGCTCTGAGTCAGTGA
TGGCGCACCAACGCAAATATCGATGTCTCACGTCTACCTTATTACTTGCCTTGATCGTCGGCACCCGATATTTGTCA
ACTGGGAATCCTCCTGGACCTATTATCGGTCCTTTGAAACTAACAGCAATTACCCCAGAAAGTGTCTGCTAA

>p1_ind1102

ATGCAAATAGACCCCGTTGCATATCATTGTTCCGAGTTGAAGTTCGTGCTTGTACCCGAAGAGTCGGGAAAGTTAT
CCCCGACGGAAATAGGATTCCATTTTCAGATTGGACGCCGCGTACATGACAGAAGACCCCGCTCTGAGTCAGTGA
TGACGCACCAACGCAGATATCGATGTCTCACGTCTACCTTATTACTTGCCTTGATCGTCGGCACCCGATATTTGTCA
ACTGGGAATCCTCCTGGACCTATTATCGGTCCTTTGAAACTAACAGCAATTACCCCAGAAAGTGTCTGCTAA

>p1_ind3376

ATGCAAATAGACCCCGTTGCATATCATTGTTCCGAGTTGAAGTTCGTGCTTGTACCCGAAGAGTCGGGAAAGTTAT
CCCCGACGGAAATAGGATTCCATTTTCAGATTGGGTGCCGCGTACATGACAGAAGACCCCGCTCTGAGTCAGTGA
TGACGCACCAACGCAGATATCGATGTCTCACGTCTACCTTATTACTTGCCTTGATCGTCGGCACCCGATATTTGTCA
ACTGGGAATCCTCCTGGACCTATTATCGGTCCTTTGAAACTAACAGCAATTACCCCAGAAAGTGTCTGCTAA

>p1_ind2598

ATGCAAATAGACCCCGTTGCATATCATTGTTCCGAGTTGAAGTTCGTGCTTGTACCCGAAGAGTCGGGAAAGTTAT
CCCCGACGGAAATAGGATTCCATTTTGAGATTGGGTGCCGCGTACATGACAGAAGACCCCGCTCTGAGTCAGTGA
TGACGCACCAACGCAGATATCGATGTCTCACGTCTACCTTATTACTTGCCTTGATCGTCGGCACCCGATATTTGTCA
ACTGGGAATCCTCCTGGACCTATTATCGGTCCTTTGAAACTAACAGCAATTACCCCAGAAAGTGTCTGCTAA

>p1_ind3843

ATGCAAATAGACCCTGTTGCATATCATTGTTCCGAGTTGAAGTTCGTGCTTGTACCCGAAGAGTCGGGAAAGTTAT
CCCCGACGGAAATAGGATTACATTTTCAGATTGGGCGCCGCGTACATGACAGAAGACCCCGCTCTGAGTCAGTGA

TGACGCACCAACGCAGATATCGATGTCTCACGTCTACCTTATTACTTGCCTTGATCGTCGGCACCCGATATTTGTCA
ACTGGGAATCCTCCTGGACCTATTATCGGTCCTTTGAAACTAACAGCAATTACCCCAGAAAGTGTCTGCTAA

>p1_ind36

ATGCAAATAGACCCCGTTGCATATCATTGTTCCGAGTTGAAGTTCGTGCTTGTACCCGAAGAGTCGGGAAAGTTAT
CCCCGACGGAAATAGGATTCCATTTTCAGACTGGGCGCCGCGTACATGACAGAAGACCCCGCTCTGAGTCAGTGA
TGACGCACCAACGCAGATATCGATGTCTCACGTCTACCTTATTACTTGCCTTGATCGTCGGCACCCGATATTTGTCA
ACTGGGAATCCTCCTGGACCTATTATCGGTCCTTTGAAACTAACAGCAATTACCCCAGAAAGTGTCTGCTAA

>p1_ind1487

ATGCAAATAGACCCCGTTGCATATCATTGTTCCGAGTTGAAGTTCGTGCTTGTACCCGAAGAGTCGGGAAAGTTAT
CCCCGACGGAAATAGGATTCCATTTTCAGATTGGGCGCCGCGTACATGACAGAAGACCCCGCTCTGAGTCAGTGA
TGACGCACCAACGCAGATATCGATGTCTCACGTCTACCTTATTACTTGCCTTGACCGTCGGCACCCGATATTTGTCA
ACTGGGAATCCTCCTGGACCTATTATCGGTCCTTTGAAACTAACAGCAATTACCCCAGAAAGTGTCTGCTAA

>p1_ind3408

ATGCAAATAGACCCCGTTGCATATCATTGTTCCGAGTTGAAGTTCGTGCTTGTACCCGAAGAGTCGGGAAAGTTAT
CCCCGACGGAAATAGGATTCCATTTTCAGATTGGGTGCCGCGTACATGACAGAAGACCCCGCTCTGAGTCAGTGA
TGACGCACCAACGCAGATATCGATGTCTCACGTCTACCTTATTACTTGCCTTGATCGTCGGCACCCGATATTTGTCA
ACTGGGAATCCTCCTGGACCTATTATCGGTCCTTTGAAACTAACAGCAATTACCCCAGAAAGTGTCTGCTAA

>p1_ind2255

ATGCAAATAGACCCCGTTGCATATCATTGTTCCGAGTTGAAGTTCGTGCTTGTACCCGAAGAGTCGGGAAAGTTAT
CCCCGACGGAAATAGGATTCCATTTTCAGATTGGGCGCCGCGTACATGACAGAAGACCCCGCTCTGAGTCAGTGA
TGACGCACCAACGCAGATATCGATGTCTCACGTCTACCTTATTACTTGCCTTGATCGTCGGCACCCGATATTTGTCA
ACTGGGAATCCTCCTGGACCTATTATCGGTCCTTTGAAACTAACAGCAATTACCCCAGAAAGTGTCTGCTAA

>p1_ind379

ATGCAAATAGACCCCGTTGCATATCATTGTTCCGAGTTGAAGTTCGTGCTTGTACCCGAAGAGTCGGGAAAGTTAT
CCCCGACGGAAATAGGATTCCATTTTCAGATTGGGCGCCGCGTACATGACAGAAGACCCCGCTCTGAGTCAGTGA
TGACGCACCAACGCAGATATCGATGTCTCACGTCTACCTTATTACTTGCCTTGATCGTCGGCACCCGATATTTGTCA
ACTGGGAATCCTCCTGGACCTATTATCGGTCCTTTGAAACTAACAGCAATTACCCCAGAAAGTGTCTGCTAA

>p1_ind1411

ATGCAAATAGACCCCGTTGCATATCATTGTTCCGAGTTGAAGTTCGTGCTTGTACCCGAAGAGTCGGGAAAGTTAT
CCCCGACGGAAATAGGATTCCATTTTCAGACTGGGCGCCGCGTACATGACAGAAGACCCCGCTCTGAGTCAGTGA
TGACGCACCAACGCAGATATCGATGTCTCACGTCTACCTAATTACTTGCCTTGATCGTCGGCACCCGATATTTGTCA
ACTGGGAATCCTCCTGGACCTATTATCGGTCCTTTGAAACTAACAGCAATTACCCCAGAAAGTGTCTGCTAA

>p1_ind5007

ATGCAAATAGACCCTGTTGCATATCATTGTTCCGAGTTGAAGTTCGTGCTTGTACCCGAAGAGTCGGGAAAGTTAT
CCCCGACGGAAATAGGATTCCATTTTCAGATTGGGTGCCGCGTACATGACAGAAGACCCCGCTCTGAGTCAGTGA
TGGCGCACCAACGCAAATATCGATGTCTCACGTCTACCTTATTACTTGCCTTGATCGTCGGCACCCGATATTTGTCA
ACTGGGAATCCTCCTGGACCTATTATCGGTCCTTTGAAACTAACAGCAATTACCCCAGAAAGTGTCTGCTAA

>p1_ind2323

ATGCAAATAGACCCCGTTGCATATCATTGTTCCGAGTTGAAGTTCGTGCTTGTACCCGAAGAGTCGGGAAAGTTAT
CCCCGACGGAAATAGGATTCCATTTTCAGATTGGACGCCGCGTACATGACAGAAGACCCCGCTCTGAGTCAGTGA
TGACGCACCAACGCAGATATCGATGTCTCACGTCTACCTTATTACTTGCCTTGATCGTCGGCACCCGATATTTGTCA
ACTGGGAATCCTCCTGGACCTATTATCGGTCCTTTGAAACTAACAGCAATTACCCCAGAAAGTGTCTGCTAA

>p1_ind4189

ATGCAAATAGACCCCGTTGCATATCATTGTTCCGAGTTGAAGTTCGTGCTTGTACCCGAAGAGTCGGGAAAGTTAT
CCCCGACGGAAATAGGATTCCATTTTCAGACTGGGCGCCGCGTACATGACAGAAGACCCCGCTCTGAGTCAGTGA
TGCCGCACCAACGCAGATATCGATGTCTCACGTCTACCTTATTACTTGCCTTGATCGTCGGCACCCGATATTTGTCA
ACTGGGAATCCTCCTGGACCTATTATCGGTCCTTTGAAACTAACAGCAATTACCCCAGAAAGTGTCTGCTAA

>p1_ind3335

ATGCAAATAGACCCCGTTGCATATCATTGTTCCGAGTTGAAGTTCGTGCTTGTACCCGAAGAGTCGGGAAAGTTAT
CCCCGACGGAAATAGGATTCCATTTTCAGACTGGGCGCCGCGTACATGACAGAAGACCCCGCTCTGAGTCAGTGA
TGACGCACCAACGCAGATATCGATGTCTCACGTCTACCTTATTACTTGCCTTGATCGTCGGCACCCGATATTTGTCA
ACTGGGAATCCTCCTGGACCTATTATCGGTCCTTTGAAACTAACAGCAATTACCCCAGAAAGTGTCTGCTAA

>p1_ind664

ATGCAAATAGACCCCGTTGCATATCATTGTTCCGAGTTGAAGTTCGTGCTTGTACCCGAAGAGTCGGGAAAGTTAT
CCCCGACGGAAATAGGATTCCATTTTCAGATTGGGTGCCGCGTACATGACAGAAGACCCCGCTCTGAGTCAGTGA
TGACGCACCAACGCAGATATCGATGTCTCACGTCTACCTTATTACTTGCCTTGATCGTCGGCACCCGATATTTGTCA
ACTGGGAATCCTCCTGGACCTATTATCGGTCCTTTGAAACTAACAGCAATTACCCCAGAAAGTGTCTGCTAA

>p1_ind1992

ATGCAAATAGACCCCGTTGCATATCATTGTTCCGAGTTGAAGTTCGTGCTTGTACCCGAAGAGTCGGGAAAGTTAT
CCCCGACGGAAATAGGATTCCATTTTCAGACTGGGCGCCGCGTACATGACAGAAGACCCCGCTCTGAGTCAGTGA
TGCCGCACCAACGCAGATATCGATGTCTCACGTCTACCTTATTACTTGCCTTGATCGTCGGCACCCGATATTTGTCA
ACTGGGAATCCTCCTGGACCTATTATCGGTCCTTTGAAACTAACAGCAATTACCCCAGAAAGTGTCTGCTAA

>p1_ind1730

ATGCAAATAGACCCCGTTGCATATCATTGTTCCGAGTTGAAGTTCGTGCTTGTACCCGAAGAGTCGGGAAAGTTAT
CCCCGACGGAAATAGGATTCCATTTTCAGACTGGGTGCCGCGTACATGACAGAAGACCCCGCTCTGAGTCAGTGA

TGACGCACCAACGCAGATATCGATGTCTCACGTCTACCTTATTACTTGCCTTGATCGTCGGCACCCGATATTTGTCA
ACTGGGAATCCTCCTGGACCTATTATCGGTCCTTTGAAACTAACAGCAATTACTCCAGAAAGTGTCTGCTAA

>p1_ind1445

ATGCAAATAGACCCCGTTGCATATCATTGTTCCGAGTAGAAGTTCGTGCTTGTACCCGAAGAGTCGGGAAAGTTAT
CCCCGACGGAAATAGGATTCCATTTTCAGACTGGGCGCCGCGTACATGACAGAAGACCCCGCTCTGAGTCAGTGA
TGACGCACCAACGCAGATATCGATGTCTCACGTCTACCTTATTACTTGCCTTGATCGTCGGCACCCGATATTTGTCA
ACTGGGAATCCTCCTGGACCTATTATCGGTCCTTTGAAACTAACAGCAATTACCCCAGAAAGTGTCTGCTAA

>p1_ind1228

ATGCAAATAGACCCCGTTGCATATCATTGTTCCGAGTTGAAGTTCGTGCTTGTACCCGAAGAGTCGGGAAAGTTAT
CCCCGACGGAAATAGGATTCCATTTTCAGACTGGACGCCGCGTACATGACAGAAGACCCCGCTCTGAGTCAGTGA
TGACGCACCAACGCAGATATCGATGTCTCACGTCTACCTTATTACTTGCCTTGATCGTCGGCACCCGATATTTGTCA
ACTGGGAATCCTCCTGGACCTATTATCGGTCCTTTGAAACTAACAGCAATTACCCCAGAAAGTGTCTGCTAA

>p1_ind174

ATGCAAATAGACCCCGTTGCATATCATTGTTCCGAGTTGAAGTTCGTGCTTGTACCCGAAGAGTCGGGAAAGTTAT
CCCCGACGGAAATAGGATTCCATTTTCAGATTGGGTGCCGCGTACATGACAGAAGACCCCGCTCTGAGTCAGTGA
TGACGCACCAACGCAGATATCGATGTCTCACGTCTACCTTATTACTTGCCTTGATCGTCGGCACCCGATATTTGTCA
ACTGGGAATCCTCCTGGACCTATTATCGGTCCTTTGAAACTAACAGCAATTACTCCAGAAAGTGTCTGCTAA

>p1_ind4228

ATGCAAATAGACCCCGTTGCATATCATTGTTCCGAGTTGAAGTTCGTGCTTGTACCCGAAGAGTCGGGAAAGTTAT
CCCCGACGGAAATAGGATTCCATTTTCAGATTGGGCGCCGCGTACATGACAGAAGACCCCGCTCTGAGTCAGTGA
TGACGCACCAACGCAGATATCGATGTCTCACGTCTACCTTATTACTTGCCTTGATCGTCGGCACCCGATATTTGTCA
ACTGGGAATCCTCCTGGACCTATTATCGGTCCTTTGAAACTAACAGCAATTACCCCAGAAAGTGTCTGCTAA

>p1_ind3697

ATGCAAATAGACCCCGTTGCATATCATTGTTCCGAGTTGAAGTTCGTGCTTGTACCCGAAGAGTCGGGAAAGTTAT
CCCCGACGGAAATAGGATTCCATTTTCAGACTGGGCGCCGCGTACATGACAGAAGACCCCGCTCTGAGTCAGTGA
TGACGCACCAACGCAGATATCGATGTCTCACGTCTACCTTATTACTTGCCTTGATCGTCGGCACCCGATATTTGTCA
ACTGGGAATCCTCCTGGACCTATTATCGGTCCTTTGAAACTAACAGCAATTACCCCAGAAAGTGTCTGCTAA

>p1_ind3428

ATGCAAATAGACCCCGTTGCATATCATTGTTCCGAGTTGAAGTTCGTGCTTGTACCCGAAGAGTCGGGAAAGTTAT
CCCCGACGGAAATAGGATTCCATTTTCAGACTGGGCGCCGCGTACATGACAGAAGACCCCGCTCTGAGTCAGTGA
TGACGCACCAACGCAGATATCGATGTCTCACGTCTACCTTATTACTTGCCTTGATCGTCGGCACCCGATATTTGTCA
ACTGGGAATCCTCCTGGACCTAGTATCGGTCCTTTGAAACTAACAGCAATTACCCCAGAAAGTGTCTGCTAA

>p1_ind3298

ATGCAAATAGACCCCGTTGCATATCATTGTTCCGAGTTGAAGTTCGTGCTTGTACCCGAAGAGTCGGGAAAGTTAT
CCCCGACGGAAATAGGATTCCATTTTCAGACTGGGCGCCGCGTACATGACAGAAGACCCCGCTCTGAGTCAGTGA
TGACGCACCAACGCAGATATCGATGTCTCACGTCTACCTTATTACTTGCCTTGATCGTCGGCACCCGATATTTGTCA
ACTGGGAATCCTCCTGGACCTATTATCGGTCCTTTGAAACTAACAGCAATTACCCCAGAAAGTGTCTGCTAA

>p1_ind2214

ATGCAAATAGACCCCGTTGCATATCATTGTTCCGAGTTGAAGTTCGTGCTTGTACCCGAAGAGTCGGGAAAGTTAT
CCCCGACGGAAATACGATTCCATTTTCAGATTGGGTGCCGCGTACATGACAGAAGACCCCGCTCTGAGTCAGTGAT
GACGCACCAACGCAGATATCGATGTCTCACGTCTACCTTATTACTTGCCTTGATCGTCGGCACCCGATATTTGTCAA
CTGGGAATCCTCCTGGACCTATTCTCGGTCCTTTGAAACTAACAGCAATTACCCCAGAAAGTGTCTGCTAA

>p1_ind4921

ATGCAAATAGACCCCGTTGCATATCATTGTTCCGAGTAGAAGTTCGTGCTTGTACCCGAAGAGTCGGGAAAGTTAT
CCCCGACGGAAATAGGATTCCATTTTCAGACTGGGCGCCGCGTACATGACAGAAGACCCCGCTCTGAGTCAGTGA
TGACGCACCAACGCAGATATCGATGTCTCACGTCTACCTTATTACTTGCCTTGATCGTCGGCACCCGATATTTGTCA
ACTGGGAATCCTCCTGGACCTATTATCGGTCCTTTGAAACTAACAGCAATTACCCCAGAAAGTGTCTGCTAA

>p1_ind4218

ATGCAAATAGACCCTGTTGCATATCATTGTTCCGAGTTGAAGTTCGTGCTTGTACCCGAAGAGTCGGGAAAGTTAT
CCCCGACGGAAATAGGATTCCATTTTCAGATTGGGTGCCGCGTACATGACAGAAGACCCCGCTCTGAGTCAGTGA
TGGCGCACCAACGCAAATACAGATGTCTCACGTCTACCTTATTACTTGCCTTGATCGTCGGCACCCGATATTTGTCA
ACTGGGAATCCTCCTGGACCTATTATCGGTCCTTTGAAACTAACAGCAATTACCCCAGAAAGTGTCTGCTAA

>p1_ind3813

ATGCAAATAGACCCCGTTGCATATCATTGTTCCGAGTTGAAGTTCGTGCTTGTACCCGAAGAGTCGGGAAAGTTAT
CCCCGACGGAAATAGGATTCCATTTTCAGATTGGGTGCCGCGTACATGACAGAAGACCCCGCTCTGAGTCAGTGA
TGACGCACCAACGCAGATATCGATGTCTCACGTCTACCTTATTACTTGCCTTGATCGTCGGCACCCGATATTTGTCA
ACTGGGAATCCTCCTGGACCTATTATCGGTCCTTTGAAACTAACAGCAATTACTCCAGAAAGTGTCTGCTAA

>p1_ind947

ATGCAAATAGACCCCGTTGCATATCATTGTTCCGAGTTGAAGTTCGTGCTTGTACCCGAAGAGTCGGGAAAGTTAT
CCCCGACGGAAATAGGATTCCATTTTCAGACTGGGCGCCGCGTACATGACAGAAGACCCCGCTCTGAGTCAGTGA
TGACGCACCAACGCAGATATCGATGTCTCACGTCTACCTTATTACTTGCCTTGATCGTCGGCACCCGATATTTGTCA
ACTGGGAATCCTCCTGGACCTATTATCGGTCCTTTGAAACTAACAGCAATTACCCCAGAAAGTGTCTGCTAA

>p1_ind100

ATGCAAATAGACCCCGTTGCATATCATTGTTCCGAGTTGAAGTTCGTGCTTGTACCCGAAGAGTCGGGAAAGTTAT
CCCCGACGGAAATAGGATTCCATTTTCAGATTGGGCGCCGCGTACATGACAGAAGACCCCGCTCTGAGTCAGTGA

TGACGCACCAACGCAGATATCGATGTCTCACGTCTACCTTATTACTTGCCTTGATCGTCGGCACCCGATATTTGTCA
ACTGGGAATCCTCCTGGACCTATTATCGGTCCTTTGAAACTAACAGCAATTACCCCAGAAAGTGTCTGCTAA

>p1_ind1600

ATGCAAATAGACCCCGTTGCATATCATTGTTCCGAGTTGAAGTTCGTGCTTGTACCCGAAGAGTCGGGAAAGTTAT
CCCCGACGGAAATAGGATTCCATTTTCAGATTGGGTGCCGCGCACATGACAGAAGACCCCGCTCTGAGTCAGTGA
TGACGCTCCAACGCAGATATCGATGTCTCACGTCTACCTTATTACTTGCCTTGATCGTCGGCACCCGATATTTGTCA
ACTGGGAATCCTCCTGGACCTATTATCGGTCCTTTGAAACTAACAGCAATTACCCCAGAAAGTGTCTGCTAA

>p1_ind3316

ATGCAAATAGACCCCGTTGCATATCATTGTTCCGAGTTGAAGTTCGTGCTTGTACCCGAAGAGTCGGGAAAGTTAT
CCCCGACGGAAATAGGATTCCATTTTCAGACTGGGCGCCGCGTACATGACAGAAGACCCCGCTCTGAGTCAGTGA
TGACGCACCAACGCAGATATCGATGTCTCACGTCTACCTTATTACTTGCCTTGATCGTCGGCACCCGATATTTGTCA
ACTGGGAATCCTCCTGGACCTATTATCGGTCCTTTGAAACTAACAGCAATTACCCCAGAAAGTGTCTGCTAA

>p1_ind4068

ATGCAAATAGACCCCGTTGCATATCATTGTTCCGAGTTGAAGTTCGTGCTTGTACCCGAAGAGTCGGGAAAGTTAT
CCCCGACGGAAATAGGATTCCATTTTCAGACTGGGCGCCGCGTACATGACAGAAGACCCCGCTCTGAGTCAGTGA
TGACGCACCAACGCAGACATCGATGTCTCACGTCTACCTTATTACTTGCCTTGATCGTCGGCACCCGATATTTGTCA
ACTGGGAATCCTCCTGGACCTATTATCGGTCCTTTGAAACTAACAGCAATTACCCCAGAAAGTGTCTGCTAA

>p1_ind2810

AAGCAAATAGACCCCGTTGCATATCATTGTTCCGAGTTGAAGTTCGTGCTTGTACCCGAAGAGTCGGGAAAGTTAT
CCCCGACGGAAATAGGATTCCATTTTCAGACTGGGCGCCGCGTACATGACAGAAGACCCCGCTCTGAGTCAGTGA
TGACGCACCAACGCAGATATCGATGTCTCACGTCTACCTTATTACTTGCCTTGATCGTCGGCACCCGATATTTGTCA
ACTGGGAATCCTCCTGGACCTATTATCGGTCCTTTGAAACTAACAGCAATTACCCCAGAAAGTGTCTGCTAA

>p1_ind1223

ATGCAAATAGACCCCGTTGCATATCATTGTTCCGAGTTGAAGTTCGTGCTTGTACCCGAAGAGTCGGGAAAGTTAT
CCCCGACGGAAATAGGATTCCATTTTCAGATTGGGCGCCGCGTACATGACAGAAGACCCCGCTCTGAGTCAGTGA
TGACGCACCAACGCAGATATCGATGTCTCACGTCTACCTTATTACTTGCCTTGATCGTCGGCACCCGATATTTGTCA
ACTGGGAATCCTCCTGGACCTATTATCGGTCCTTTGAAACTAACAGCAATTACCCCAGAAAGTGTCTGCTAA

>p1_ind3151

ATGCAAATAGACCCCGTTGCATATCATTGTTCCGAGTTGAAGTTCGTGCTTGTACCCGAAGAGTCGGGAAAGTTAT
CCCCGACGGAAATAGGATTCCATTTTCAGACTGGGCGCCGCGTACATGACAGAAGACCCCGCTCTGAGTCAGTGA
TGACGCACCAACGCAGATATCGATGTCTCACGTCTACCTTATTACTTGGCTTGATCGTCGGCACCCGATATTTGTCA
ACTGGGAATCCTCCTGGACCTATTATCGGTCCTTTGAAACTAACAGCAATTACCCCAGAAAGTGTCTGCTAA

>p1_ind2321

ATGCAAATAGACCCCGTTGCATATCATTGTTCCGAGTTGAAGTTCGTGCTTGTACCCGAAGAGTCGGGAAAGTTAT
CCCCGACGGAAATAGGATTCCATTTTCAGACTGGGCGCCGCGTACATGACAGAAGACCCCGCTCTGAGTCAGTGA
TGACGCACCAACGCAGATATCGATGTCTCACGTCTACCTTATTACTTGCCTTGATCGTCGGCACCCGATATTTGTCA
ACTGGGAATCCTCCTGGACCTATTATCGGTCCTTTGAAACTAACAGCAATTACCCCAGAAAGTGTCTGCTAA

>p1_ind3835

ATGCAAATAGACCCCGTTGCATATCATTGTTCCGAGTTGAAGTTCGTGCTTGTACCCGAAGAGTCGGGAAAGTTAT
CCCCGACGGAAATAGGATTCCATTTTCAGACTGGGCGCCGCGTACATGACAGAAGACCCCGCTCTGAGTCAGTGA
TGACGCACCAACGCAGATATCGATGTCTCACGTCTACCTTATTACTTGCCTTGATCGTCGGCACCCGATATTTGTCA
ACTGGGAATCCTCCTGGACCTATTATCGGTCCTTTGAAACTAACAGCAATTACCCCAGAAAGTGTCTGCTAA

>p1_ind281

ATGCAAATAGACCCCGTTGCATATCATTGTTCCGAGTTGAAGTTCGTGCTTGTACCCGAAGAGTCGGGAAAGTTAT
CCCCGACGGAAATAGGATTCCATTTTCAGACTGGGCGCCGCGTACATGACAGAAGACCCCGCTCTGAGTCAGTGA
TGACGCACCAACGCAGATATCGATGTCTCACGTCTACCTTATTACTTGCCTTGATCGTCGGCACCCGATATTTGTCA
ACTGGGAATCCTCCTGGAGCTATTATCGGTCCTTTGAAACTAACAGCAATTACCCCAGAAAGTGTCTGCTAA

>p1_ind2147

ATGCAAATAGACCCCGTTGCATATCATTGTTCCGAGTTGAAGTTCGTGCTTGTACCCGAAGAGTCGGGAAAGTTAT
CCCCGACGGAAATAGGATTCCATTTTCAGATTGGGTGCCGCGTACATGACAGAAGACCCCGCTCTGAGTCAGTGA
TGACGCACCAACGCAGATATCGATGTCTCACGTCTACCTTATTACTTGCCTTGATCGTCGGCACCCGATATTTGTCA
ACTGGGAATCCTCCTGGACCTATTATCGGTCCTTTGAAACTAACAGCAATTACCCCAGAAAGTGTCTGCTAA

>p1_ind5034

ATGCAAATAGACCCCGTTGCATATCATTGTTCCGAGTTGAAGTTCGTGCTTGTACCCGAAGAGTCGGGAAAGTTAT
CCCCGACGGAAATAGGATTCCATTTTCAGATTGGGCGCCGCGTACATGACAGAAGACCCCGCTCTGAGTCAGTGA
TGACGCACCAACGCAGATATCGATGTCTCACGTCTACCTTATTACTTGCCTTGATCGTCGGCACCCGATATTTGTCA
ACTGGGAATCCTCCTGGACCTATTATCGGTCCTTTGAAACTAACAGCAATTACCCCAGAAAGTGTCTGCTAA

>p1_ind3445

ATGCAAATAGACCCCGTTGCATATCATTGTTCCGAGTTGAAGTTCGTGCTTGTACCCGAAGAGTCGGGAAAGTTAT
CCCCGACGGAAATAGGATTCCATTTTCAGATTGGACGCCGCGTACATGACAGAAGACCCCGCTCTGAGTCAGTGA
TGACGCACCAACGCAGATATCGATGTCTCACGTCTACCTTATTACTTGCCTTGATCGTCGGCACCCGATATTTGTCA
ACTGGGAATCCTCCTGGACCTATTATCGGTCCTTTGAAACTAACAGCAATTACCCCAGAAAGTGTCTGCTAA

>p1_ind292

ATGCAAATAGACCCCGTTGCATATCATTGTTCCGAGTAGAAGTTCGTGCTTGTACCCGAAGAGTCGGGAAAGTTAT
CCCCGACGGAAATAGGATTCCATTTTCAGACTGGGCGCCGCGTACATGACAGAAGACCCCGCTCTGAGTCAGTGA

TGACGCACCAACGCAGATATCGATGTCTCACGTCTACCTTATTACTTGCCTTGATCGTCGGCACCCGATATTTGTCA
ACTGGGAATCCTCCTGGACCTATTATCGGTCCTTTGAAACTAACAGCAATTACCCCAGAAAGTGTCTGCTAA

>p1_ind182

ATGCAAATAGACCCCGTTGCATATCATTGTTCAGAGTTGAAGTTCGTGCTTGTACCCGAAGAGTCGGGAAAGTTAT
CCCCGACGGAAATAGGATTCCATTTTCAGACTGGGCGCCGCGTACATGACAGAAGACCCCGCTCTGAGTCAGTGA
TGACGCACCAACGCAGATATCGATGTCTCACGTCTACCTTATTACTTGCCTTGATCGTCGGCACCCGATATTTGTCA
ACTGGGAATCCTCCTGGACCTATTATCGGTCCTTTGAAACTAACAGCAATTACCCCAGAAAGTGTCTGCTAA

>p1_ind459

ATGCAAATAGACCCCGTTGCATATCATTGTTCCGAGTTGAAGTTCGTGCTTGTACCCGAAGAGTCGGGAAAGTTAT
CCCCGACGGAAATAGGATTCCATTTTCAGACTGGGCGCCGCGTACATGACAGAAGACCCCGCTCTGAGTCAGTGA
TGACGCACCAACGCAGACATCGATGTCTCACGTCTACCTTATTACTTGCCTTGATCGTCGGCACCCGATATTTGTCA
ACTGGGAATCCTCCTGGACCTATTATCGGTCCTTTGAAACTAACAGCAATTACCCCAGAAAGTGTCTGCTAA

>p1_ind417

ATGCAAATAGACCCCGTTGCATATCATTGTTCCGAGTTGAAGTTCGTGCTTGTACCCGAAGAGTCGGGAAAGTTAT
CCCCGACGGAAATAGGATTCCATTTTCAGATTGGGTGCCGCGTACATGACAGAAGACCCCGCTCTGAGTCAGTGA
TGACGCACCAACGCAGATATCGATGTCTCACGTCTACCTTATTACTTGCCTTGATCGTCGGCACCCGATATTTGTCA
ACTGGGAATCCTCCTGGACCTATTATCGGTCCTTTGAAACTAACAGCAATTACCCCAGAAAGTGTCTGCTAA

>p1_ind2847

ATGCAAATAGACCCCGTTGCATATCATTGTTCCGAGTTGAAGTTCGTGCTTGTACCCGAAGAGTCGGGAAAGTTAT
CCCCGACGGAAATAGGATTCCATTTTCAGATTGGGTGCCGCGTACATGACAGAAGACCCCGCTCTGAGTCAGTGA
TGACGCACCAACGCAGATATCGATGTCTCACGTCTACCTTATTACTTGCCTTGATCGTCGGCACCCGATATTTGTCA
ACTGGGAATCCTCCTGGACCTATTATCGGTCCTTTGAAACTAACAGCAATTACCCCAGAAAGTGTCTGCTAA

>p1_ind1033

ATGCAAATAGACCCCGTTGCATATCATTGTTCCGAGTTGAAGTTCGTGCTTGTACCCGAAGAGTCGGGAAAGTTAT
CCCCGACGGAAATAGGATTCCATTTTCAGATTGGGTGCCGCGTACATGACAGAAGACCCCGCTCTGAGTCAGTGA
TGACGCACCAACGCAGATATCGATGTCTCACGTCTACCTTATTACTTGCCTTGATCGTCGGCACCCGATATTTGTCA
ACTGGGAATCCTCCTGGACCTATTATCGGTCCTTTGAAACTAACAGCAATTACCCCAGAAAGTGTCTGCTAA

>p1_ind3919

ATGCAAATAGACCCCGTTGCATATCATTGTTCCGAGTTGAAGTTCGTGCTTGTACCCGAAGAGTCGGGAAAGTTAT
CCCCGACGGAAATAGGATTCCATTTTCAGATTGGGTGCCGCGTACATGACAGAAGACCCCGCTCTGAGTCAGTGA
TGACGCACCAACGCAGATATCGATGTCTCACGTCTACCTTATTACTTGCCTTGATCGTCGGCACCCGATATTTGTCA
ACTGGGAATCCTCCTGGACCTATTATCGGTCCTTTGAAACTAACAGCAATTACCCCAGAAAGTGTCTGCTAA

>p1_ind1864

ATGCAAATAGACCCCGTTGCATATCATTGTTCCGAGTTGAAGTTCGTGCTTGTACCCGAAGAGTCGGGAAAGTTAT
CCCCGACGGAAATAGGATTCCATTTTCAGATTGGGTGCCGCGTACATGACAGAAGACCCCGCTCTGAGTCAGTGA
TGACGCACCAACGCAGATATCGATGTCTCACGTCTACCTTATTACTTGCCTTGATCGTCGGCACCCGATATTTGTCA
ACTGGGAATCCTCCTGGACCTATTATCGGTCCTTTGAAACTAACAGCAATTACCCCAGAAAGTGTCTGCTAA

>p1_ind1849

ATGCAAATAGACCCTGTTGCATATCATTGTTCCGAGTTGAAGTTCGTGCTTGTACCCGAAGAGTCGGGAAAGTTAT
CCCCGACGGAAATAGGATTCCATTTTCAGATTGGGTGCCGCGTACATGACAGAAGACCCCGCTCTGAGTCAGTGA
TGGCGCACCAACGCAAATATCGATGTCTCACGTCTACCTTATTACTTGCCTTGATCGTCGGCACCCGATATTTGTCA
ACTGGGAATCCTCCTGGACCTATTATCGGTCCTTTGAAACTAACAGCAATTACCCCAGAAAGTGTCTGCTAA

>p1_ind3718

ATGCAAATAGACCCCGTTGCATATCATTGTTCCGAGTTGAAGTTCGTGCTTGTACCCGCAGAGTCGGGAAAGTTAT
CCCCGACGGAAATAGGATTCCATTTTCAGATTGGGTGCCGCGTACATGACAGAAGACCCCGCTCTGAGTCAGTGA
TGACGCACCAACGCAGATATCGATGTCTCACGTCTACCTTATTACTTGCCTTGATCGTCGGCACCCGATATTTGTCA
ACTGGGAATCCTCCTGGACCTATTATCGGTCCTTTGAAACTAACAGCAATTACCCCAGAAAGTGTCTGCTAA

>p1_ind492

ATGCAAATAGACCCCGTTGCATATCATTGTTCCGAGTTGAAGTTCGTGCTTGTACCCGAAGAGTCGGGAAAGTTAT
CCCCGACGGAAATAGGATTCCATTTTCAGATTGGGTGCCGCGTACATGACAGAAGACCCCGCTCTGAGTCAGTGA
TGACGCACCAACGCAGATATCGATGTCTCACGTCTACCTTATTACTTGCCTTGATCGTCGGCACCCGATATTTGTCA
ACTGGGAATCCTCCTGGACCTATTATCGGTCCTTTGAAACTAACAGCAATTACCCCAGAAAGTGTCTGCTAA

>p1_ind2523

ATGCAAATAGACCCCGTTGCATATCATTGTTCCGAGTTGAAGTTCGTGCTTGTACCCGAAGAGTCGGGAAAGTTAT
CCCCGACGGAAATAGGATTCCATTTTCAGATTGGGTGCCGCGTACATGACAGAAGACCCCGCTCTGAGTCAGTGA
TGACGCACCAACGCAGATATCGATGTCTCACGTCTACCTTATTACTTGCCTTGATCGTCGGCACCCGATATTTGTCA
ACTGGGAATCCTCCTGGACCTATTATCGGTCCTTTGAAACTAACAGCAATTACTCCAGAAAGTGTCTGCTAA

>p1_ind3131

ATGCAAATAGACCCCGTTGCATATCATTGTTCCGAGTTGAAGTTCGTGCTTGTACCCGAAGAGTCGGGAAAGTTAT
CCCCGACGGAAATAGGATTCCATTTTCAGATTGGGCGCCGCGTACATGACAGAAGACCCCGCTCTGAGTCAGTGA
TGACGCACCAACGCAGATATCGATGTCTCACGTCTACCTTATTACTTGCCTTGATCGTCGGCACCCGATATTTGTCA
ACTGGGAATCCTCCTGGACCTATTATCGGTCCTTTGAAACTAACAGCAATTACCCCAGAAAGTGTCTGCTAA

>p1_ind4117

ATGCAAATAGACCCCGTTGCATATCATTGTTCCGAGTTGAAGTTCGTGCTTGTACCCGAAGAGTCGGGAAAGTTAT
CCCCGACGGAAATAGGATTCCATTTTGAGATTGGGTGCCGCGTACATGACAGAAGACCCCGCTCTGAGTCAGTGA

TGACGCACCAACGCAGATATCGATGTCTCACGTCTACCTTATTACTTGCCTTGATCGTCGGCACCCGATATTTGTCA
ACTGGGAATCCTCCTGGACCTATTATCGGTCCTTTGAAACTAACAGCAATTACCCCAGAAAGTGTCTGCTAA

>p1_ind5028

ATGCAAATAGACCCCGTTGCATATCATTGTTCCGAGTTGAAGTTCGTGCTTGTACCCGAAGAGTCGGGAAAGTTAT
CCCCGACGGAAATAGGATTCCATTTTCAGATTGGACGCCGCGTACATGACAGAAGACCCCGCTCTGAGTCAGTGA
TGACGCACCAACGCAGATATCGATGTCTCACGTCTACCTTATTACTTGCCTTGATCGTCGGCACCCGATATTTGTCA
ACTGGGAATCCTCCTGGACCTATTATCGGTCCTTTGAAACTAACAGCAATTACCCCAGAAAGTGTCTGCTAA

>p1_ind223

ATGCAAATAGACCCCGTTGCATATCATTGTTCCGAGTTGAAGTTCGTGCTTGTACCCGAAGAGTCGGGAAAGTTAT
CCCCGACGGAAATAGGATTCCATTTTCAGACTGGGCGCCGCGTACATGACAGAAGACCCCGCTCTGAGTCAGTGA
TGACGCACCAACGCAGATATCGATGTCTCACGTCTACCTTATTACTTGCCTTGATCGTCGGCACCCGATATTTGTCA
ACTGGGAATCCTCCTGGACCTATTATCGGTCCTTTGAAACTAACAGCAATTACCCCAGAAAGTGTCTGCTAA

>p1_ind644

ATGCAAATAGACCCCGTTGCATATCATTGTTCCGAGTTGAAGTTCGTGCTTGTACCCGAAGAGTCGGGAAAGTTAT
CCCCGACGGAAATAGGATTCCATTTTCAGACTGGGCGCCGCGTACATGACAGAAGACCCCGCTCTGAGTCAGTGA
TGGCGCACCAACGCAGATATCGATGTCTCACGTCTACCTTATTACTTGCCTTGATCGTCGGCACCCGATATTTGTCA
ACTGGGAATCCTCCTGGACCTATTATCGGTCCTTTGAAACTAACAGCAATTACCCCAGAAAGTGTCTGCTAA

>p1_ind97

ATGCAAATAGACCCCGTTGCATATCATTGTTCCGAGTTGAAGTTCGTGCTTGTACCCGAAGAGTCGGGAAAGTTAT
CCCCGACGGAAATAGGATTCCATTTTCAGATTGGGTGCCGCGCACATGACAGAAGACCCCGCTCTGAGTCAGTGA
TGACGCACCAACGCAGATATCGATGTCTCACGTCTACCTTATTACTTGCCTTGATCGTCGGCACCCGATATTTGTCA
ACTGGGAATCCCCCTGGACCTATTATCGGTCCTTTGAAACTAACAGCAATTACCCCAGAAAGTGTCTGCTAA

>p1_ind3743

ATGCAAATAGACCCTGTTGCATATCATTGTTCCGAGTTGAAGTTCGTGCTTGTACCCGAAGAGTCGGGAAAGTTAT
CCCCGACGGAAATAGGATTACATTTTCAGATTGGGCGCCGCGTACATGACAGAAGACCCCGCTCTGAGTCAGTGA
TGACGCACCAACGCAGATATCGATGTCTCACGTCTACCTTATTACTTGCCTTGATCGTCGGCACCCGATATTTGTCA
ACTGGGAATCCTCCTGGACCTATTATCGGTCCTTTGAAACTAACAGCAATTACCCCAGAAAGTGTCTGCTAA

>p1_ind3168

ATGCAAATAGACCCCGTTGCATATCATTGTTCCGAGTAGAAGTTCGTGCTTGTACCCGAAGAGTCGGGAAAGTTAT
CCCCGACGGAAATAGGATTCCATTTTCAGACTGGGCGCCGCGTACATGACAGAAGACCCCGCTCTGAGTCAGTGA
TGACGCACCAACGCAGATATCGATGTCTCACGTCTACCTTATTACTTGCCTTGATCGTCGGCACCCGATATTTGTCA
ACTGGGAATCCTCCTGGACCTATTATCGGTCCTTTGAAACTAACAGCAATTACCCCAGAAAGTGTCTGCTAA

>p1_ind1273

ATGCAAATAGACCCTGTTGCATATCATTGTTCCGAGTTGAAGTTCGTGCTTGTACCCGAAGAGTCGGGAAAGTTAT
CCCCGACGGAAATAGGATTCCATTTTCAGATTGGGTGCCGCGTACATGACAGAAGACCCCGCTCTGAGTCAGTGA
TGGCGCACCAACGCAGATATCGATGTCTCACGTCTACCTTATTACTTGCCTTGATCGTCGGCACCCGATATTTGTCA
ACTGGGAATCCTCCTGGACCTATTATCGGTCCTTTGAAACTAACAGCAATTACCCCAGAAAGTGTCTGCTAA

>p1_ind5136

ATGCAAATAGACCCCGTTGCATATCATTGTTCCGAGTTGAAGTTCGTGCTTGTACCCGAAGAGTCGGGAAAGTTAT
CCCCGACGGAAATAGGATTCCATTTTCAGATTGGACGCCGCGTACATGACAGAAGACCCCGCTCTGAGTCAGTGA
TGACGCACCAACGCAGATATCGATGTCTCACGTCTACCTTATTACTTGCCTTGATCGTCGGCACCCGATATTTGTCA
ACTGGGAATCCTCCTGGACCTATTATCGGTCCTTTGAAACTAACAGCAATTACCCCAGAAAGTGTCTGCTAA

>p1_ind3214

ATGCAAATAGACCCCGTTGCATATCATTGTTCCGAGCAGAAGTTCGTGCTTGTACCCGAAGAGTCGGGAAAGTTAT
CCCCGACGGAAATAGGATTCCATTTTCAGATTGGGTGCCGCGTACATGACAGAAGACCCCGCTCTGAATCAGTGAT
GACGCACCAACGCAGATATCGATGTCTCACGTCTACCTTATTACTTGCCTTGATCGTCGGCACCCGATATTTGTCAA
CTGGGAATCCTCCTGGACCTATTATCGGTCCTTTGAAACTAACAGCAATTACCCCAGAAAGTGTCTGCTAA

>p1_ind4837

ATGCAAATAGACCCCGTTGCATATCATTGTTCCGAGTTGAAGTTCGTGCTTGTACCCGAAGAGTCGGGAAAGTTAT
CCCCGACGGAAATAGGATTCCATTTTCAGACTGGGCGCCGCGTACATGACAGAAGACCCCGCTCTGAGTCAGTGA
TGACGCACCAACGCAGATATCGATGTCTCACGTCTACCTTATTACTTGCCTTGATCGTCGGCACCCGATATTTGTCA
ACTGGGAATCCTCCTGGACCTATTATCGGTCCTTTGAAACTAACAGCAATTACCCCAGAAAGTGTCTGCTAA

>p1_ind3302

ATGCAAATAGACCCCGTTGCATATCATTGTTCCGAGTTGAAGTTCGTGCTTGTACCCGAAGAGTCGGGAAAGTTAT
CCCCGACGGAAATAGGATTCCATTTTCAGATTGGGTGCCGCGCACATGACAGAAGACCCCGCTCTGAGTCAGTGA
TGACGCACCAACGCAGATATCGATGTCTCACGTCTACCTTATTACTTGCCTTGATCGTCGGCACCCGATATTTGTCA
ACTGGGAATCCTCCTGGACCTATTATCGGTCCTTTGAAACTAACAGCAATTACCCCAGAAAGTGTCTGCTAA

>p1_ind1167

ATGCAAATAGACCCCGTTGCATATCATTGTTCCGAGTTGAAGTTCGTGCTTGTACCCGAAGAGTCGGGAAAGTTAT
CCCCGACGGAAATAGGATTCCATTTTCAGACTGGGCGCCGCGTACATGACAGAAGACCCCGCTCTGAGTCAGTGA
TGACGCACCAACGCAGATATCGATGTCTCACGTCTACCTTATTACTTGCCTTGATCGTCGGCACCCGATATTTGTCA
ACTGGGAATCCTCCTGGACCTATTCTCGGTCCTTTGAAACTAACAGCAATTACCCCAGAAAGTGTCTGCTAA

>p1_ind3558

ATGCAAATAGACCCTGTTGCATATCATTGTTCCGAGTTGAAGTTCGTGCTTGTACCCGAAGAGTCGGGAAAGTTAT
CCCCGACGGAAATAGGATTCCATTTTCAGATTGGGTGCCGCGTACATGACAGAAGACCCCGCTCTGAGTCAGTGA

TGGCGCACCAACGCAAATATCGATGTCTCACGTCTACCTTATTACTTGCCTTGATCGTCGGCACCCGATATTTGTCA
ACTGGGAATCCTCCTGGACCTATTATCGGTCCTTTGAAACTAACAGCAATTACCCCAGAAAGTGTCTGCTAA

>p1_ind686

ATGCAAATAGACCCCGTTGCATATCATTGTTCCGAGTTGAAGTTCGTGCTTGTACCCGAAGAGTCGGGAAAGTTAT
CCCCGACGGAAATAGGATTCCATTTTCAGACTGGGCGCCGCGTACATGACAGAAGACCCCGCTCTGAGTCAGTGA
TGACGCACCAACGCAGATATCGATGTCTCACGTCTACCTTATTACTTGCCTTGATCGTCGGCACCCGATATTTGTCA
ACTGGGAATCCTCCTGGACCTATTATCGGTCCTTTGAAACTAACAGCAATTACCCCAGAAAGTGTCTGCTAA

>p1_ind3518

ATGCAAATAGACCCCGTTGCATATCATTGTTCCGAGTTGAAGTTCGTGCTTGTACCCGAAGAGTCGGGAAAGTTAT
CCCCGACGGAAATAGGATTCCATTTTCAGATTGGGCGCCGCGTACATGACAGAAGACCCCGCTCTGAGTCAGTGA
TGACGCACCAACGCAGATATCGATGTCTCACGTCTACCTTATTACTTGCCTTGATCGTCGGCACCCGATATTTGTCA
ACTGGGAATCCTCCTGGACCTATTATCGGTCCTTTGAAACTAACAGCAATTACCCCAGAAAGTGTCTGCTAA

>p1_ind401

ATGCAAATAGACCCCGTTGCATATCATTGTTCCGAGTTGAAGTTCGTGCTTGTACCCGAAGAGTCGGGAAAGTTAT
CCCCGACGGAAATAGGATTCCATTTTCAGACTGGGCGCCGCGTACATGACAGAAGACCCCGCTCTGAGTCAGTGA
TGACGCACCAACGCAGATATCGATGTCTCACGTCTACCTTATTACTTGCCTTGATCGTCGGCACCCGATATTTGTCA
ACTGGGAATCCTCCTGGACCTATTATCGGTCCTTTGAAACTAACAGCAATTACCCCAGAAAGTGTCTGCTAA

>p1_ind1278

ATGCAAATAGACCCCGTTGCATATCATTGTTCCGAGTTGAAGTTCGTGCTTGTACCCGAAGAGTCGGGAAAGTTAT
CCCCGACGGAAATAGGATTCCATTTTCAGATTGGGTGCCGCGTACATGACAGAAGACCCCGCTCTGAGTCAGTGA
TGACGCACCAACGCAGATATCGATGTCTCACGTCTACCTTATTACTTGCCTTGATCGTCGGCACCCGATATTTGTCC
ACTGGGAATCCTCCTGGACCTATTATCGGTCCTTTGAAACTAACAGCAATTACCCCAGAAAGTGTCTGCTAA

>p1_ind3506

ATGCAAATAGACCCCGTTGCATATCATTGTTCCGAGTTGAAGTTCGTGCTTGTACCCGAAGAGTCGGGAAAGTTAT
CCCCGACGGAAATAGGATTCCATTTTCAGACTGGGCGCCGCGTACATGACAGAAGACCCCGCTCTGAGTCAGTGA
TGACGCACCAACGCAGATATCGATGTCTCACGTCTACCTTATTACTTGCCTTGATCGTCGGCACCCGATATTTGTCA
ACTGGGAATCCTCCTGGACCTATTATCGGTCCTTTGAAACTAACAGCAATTACCCCAGAAAGTGTCTGCTAA

>p1_ind4069

ATGCAAATAGACCCCGTTGCATATCATTGTTCCGAGTTGAAGTTCGTGCTTGTACCCGAAGAGTCGGGAAAGTTAT
CCCCGACGGAAATAGGATTCCATTTTCAGACTGGGCGCCGCGTACATGACAGAAGACCCCGCTCTGAGTCAGTGA
TGACGCACCAACGCAGATATCGATGTCTCACGTCTACCTTATTACTTGCCTTGATCGTCGGCACCCGATATTTGTCA
ACTGGGAATCCTCCTGGACCTATTATCGGTCCTTTGAAACTAACAGCAATTACCCCAGAAAGTGTCTGCTAA

>p1_ind4909

ATGCAAATAGACCCCGTTGCATATCATTGTTCCGAGTTGAAGTTCGTGCTTGTACCCGAAGAGTCGGGAAAGTTAT
CCCCGACGGAAATAGGATTCCATTTTCAGACTGGGCGCCGCGTACATGACAGAAGACCCCGCTCTGAGTCAGTGA
TGACGCACCAACGCAGACATCGATGTCTCACGTCTACCTTATTACTTGCCTTGATCGTCGGCACCCGATATTTGTCA
ACTGGGAATCCTCCTGGACCTATTATCGGTCCTTTGAAACTAACAGCAATTACCCCAGAAAGTGTCTGCTAA

>p1_ind3007

ATGCAAATAGACCCCGTTGCATATCATTGTTCCGAGTTGAAGTTCGTGCTTGTACCCGAAGAGTCGGGAAAGTTAT
CCCCGACGGAAATAGGATTCCATTTTCAGACTGGGCGCCGCGTACATGACAGAAGACCCCGCTCTGAGTCAGTGA
TGACGCACCAACGCAGATATCGATGTCTCACGTCTACCTTATTACTTGCCTTGATCGTCGGCACCCGATATTTGTCA
ACTGGGAATCCTCCTGGACCTATTATCGGTCCTTTGAAACTAACAGCAATTACCCCAGAAAGTGTCTGCTAA

>p1_ind1722

ATGCAAATAGACCCCGTTGCATATCATTGTTCCGAGTTGAAGTTCGTGCTTGTACCCGAAGAGTCGGGAAAGTTAT
CCCCGACGGAAATAGGATTCCATTTTCAGATTGGGTGCCGCGTACATGACAGAAGACCCCGCTCTGAGTCAGTGA
TGACGCACCAACGCAGATATCGATGTCTCACGTCTACCTTATTACTTGCCTTGATCGTCGGCACCCGATATTTGTCA
ACTGGGAATCCTCCTGGACCTATTATCGGTCCTTTGAAACTAACAGCAATTACCCCAGAAAGTGTCTGCTAA

>p1_ind4059

ATGCAAATAGACCCCGTTGCATATCATTGTTCCGAGTTGAAGTTCGTGCTTGTACCCGAAGAGTCGGGAAAGTTAT
CCCCGACGGAAATAGGATTCCATTTTCAGACTGGGCGCCGCGTACATGACAGAAGACCCCGCTCTGAGTCAGTGA
TGACGCACCAACGCAGATATCGATGTCTCACGTCTACCTTATTACTTGCCTTGATCGTCGGCACCCGATATTTGTCA
ACTGGGAATCCTCCTGGACCTATTATCGGTCCTTTGAAACTAACAGCAATTACCCCAGAAAGTGTCTGCTAA

>p1_ind4120

ATGCAAATAGACCCCGTTGCATATCATTGTTCCGAGTTGAAGTTCGTGCTTGTACCCGAAGAGTCGGGAAAGTTAT
CCCCGACGGAAATAGGATTCCATTTTCAGATTGGGTGCCGCGTACATGACAGAAGACCCCGCTCTGAGTCAGTGA
TGGCGCACCAACGCAAATATCGATGTCTCACGTCTACCTTATTACTTGCCTTGATCGTCGGCACCCGATATTTGTCA
ACTGGGAATCCTCCTGGACCTATTATCGGTCCTTTGAAACTAACAGCAATTACCCCAGAAAGTGTCTGCTAA

>p1_ind3565

ATGCAAATAGACCCCGTTGCATATCATTGTTCCGAGTTGAAGTTCGTGCTTGTACCCGAAGAGTCGGGAAAGTTAT
CCCCGACGGAAATAGGATTCCATTTTCAGACTGGGCGCCGCGTACATGACAGAAGACCCCGCTCTGAGTCAGTGA
TGACGCACCAACGCAGATATCGATGTCTCACGTCTACCTTATTACTTGCCTTGATCGTCGGCACCCGATATTTGTCA
ACTGGGAATCCTCCTGGACCTATTATCGGTCCTTTGAAACTAACAGCAATTACCCCAGAAAGTGTCTGCTAA

>p1_ind752

ATGCAAATAGACCCCGTTGCATATCATTGTTCCGAGTTGAAGTTCGTGCTTGTACCCGAAGAGTCGGGAAAGTTAT
CCCCGACGGAAATAGGATTCCATTTTCAGATTGGGTGCCGCGTACATGACAGAAGACCCCGCTCTGAGTCAGTGA

TGACGCCCCAACGCAGATATCGATGTCTCACGTCTACCTTATTACTTGCCTTGATCGTCGGCACCCGATATTTGTCA
ACTGGGAATCCTCCTGGACCTATTATCGGTCCTTTGAAACTAACAGGAATTACCCCAGAAAGTGTCTGCTAA

>p1_ind3834

ATGCAAATAGACCCCGTTGCATATCATTGTTCCGAGTTGAAGTTCGTGCTTGTACCCGAAGAGTCGGGAAAGTTAT
CCCCGACGGAAATAGGATTCCATTTTCAGACTGGGCGCCGCGTACATGACAGAAGACCCCGCTCTGAGTCAGTGA
TGGCGCACCAACGCAGATATCGATGTCTCACGTCTACCTTATTACTTGCCTTGATCGTCGGCACCCGATATTTGTCA
ACTGGGAATCCTCCTGGACCTATTATCGGTCCTTTGAAACTAACAGCAATTACCCCAGAAAGTGTCTGCTAA

>p1_ind4076

ATGCAAATAGACCCCGTTGCATATCATTGTTCCGAGTTGAAGTTCGTGCTTGTACCCGAAGAGTCGGGAAAGTTAT
CCCCGACGGAAATAGGATTCCATTTTCAGACTGGGCGCCGCGTACATGACAGAAGACCCCGCTCTGAGTCAGTGA
TGACGCACCAACGCAGATATCGATGTCTCACGTCTACCTTATTACTTGCCTTGATCGTCGGCACCCGATATTTGTCA
ACTGGGAATCCTCCTGGACCTATTATCGGTCCTTTGAAACTAACAGCAATTACCCCAGAAAGTGTCTGCTAA

>p1_ind3156

ATGCAAATAGACCCCGTTGCATATCATTGTTCCGAGTTGAAGTTCGTGCTTGTACCCGAAGAGTCGGGAAAGTTAT
CCCCGACGGAAATAGGATTCCATTTTCAGATTGGGCGCCGCGTACATGACAGAAGACCCCGCTCTGAGTCAGTGA
TGACGCACCAACGCAGATATCGATGTCTCACGTCTACCTTATTACTTGCCTTGATCGTCGGCACCCGATATTTGTCA
ACTGGGAATCCTCCTGGACCTATTATCGGTCCTTTGAAACTAACAGCAATTACCCCAGAAAGTGTCTGCTAA

>p1_ind332

ATGCAAATAGACCCCGTTGCATATCATTGTTCCGAGTTGAAGTTCGTGCTTGTACCCGAAGAGTCGGGAAAGTTAT
CCCCGACGGAAATAGGATTCCATTTTCAGATTGGGTGCCGCGCACATGACAGAAGACCCCGCTCTGAGTCAGTGA
TGACGCACCAACGCAGATATCGATGTCTCACGTCTACCTTATTACTTGCCTTGATCGTCGGCACCCGATATTTGTCA
ACTGGGAATCCCCCTGGACCTATTATCGGTCCTTTGAAACTAACAGCAATTACCCCAGAAAGTGTCTGCTAA

>p1_ind4305

ATGCAAATAGACCCCGTTGCATATCATTGTTCCGAGTAGAAGTTCGTGCTTGTACCCGAAGAGTCGGGAAAGTTAT
CCCCGACGGAAATAGGATTCCATTTTCAGACTGGGCGCCGCGTACATGACAGAAGACCCCGCTCTGAGTCAGTGA
TGACGCACCAACGCAGATATCGATGTCTCACGTCTACCTTATTACTTGCCTTGATCGTCGGCACCCGATATTTGTCA
ACTGGGAATCCTCCTGGACCTATTATCGGTCCTTTGAAACTAACAGCAATTACCCCAGAAAGTGTCTGCTAA

>p1_ind3925

ATGCAAATAGACCCCGTTGCATATCATTGTTCCGAGTTGAAGTTCGTGCTTGTACCCGAAGAGTCGGGAAAGTTAT
CCCCGACGGAAATAGGATTCCATTTTCAGATTGGGTGCCGCGTACATGACAGAAGACCCCGCTCTGAGTCAGTGA
TGACGCACCAACGCAGATATCGATGTCTCACGTCTACCTTATTACTTGCCTTGATCGTCGGCACCCGATATTTGTCA
ACTGGGAATCCTCCTGGACCTATTATCGGTCCTTTGAAACTAACAGCAATTACCCCAGAAAGTGTCTGCTAA

>p1_ind2571

ATGCAAATAGACCCCGTTGCATATCATTGTTCCGAGTTGAAGTTCGTGCTTGTACCCGAAGAGTCGGGAAAGTTAT
CCCCGACGGAAATAGGATTCCATTTTCAGATTGGGTGCCGCGTACATGACAGAAGACCCCGCTCTGAGTCAGTGA
TGACGCACCAACGCAGATATCGATGTCTCACGTCTACCTTATTACTTGCCTTGATCGTCGGCACCCGATATTTGTCA
ACTGGGAATCCTCCTGGACCTATTATCGGTCCTTTGAAACTAACAGTAATTACCCCAGAAAGTGTCTGCTAA

>p1_ind1513

ATGCAAATAGACCCCGTTGCATATCATTGTTCCGAGTTGAAGTTCGTGCTTGTACCCGAAGAGTCGGGAAAGTTAT
CCCCGACGGAAATAGGATTCCATTTTCAGATTGGGTGCCGCGTACATGACAGAAGACCCCGCTCTGAGTCAGTGA
TGACGCACCAACGCAGATATCGATGTCTCACGTCTACCTTATTACTTGCCTTGATCGTCGGCACCCGATATTTGTCA
ACTGGGAATCCTCCTGGACCTATTATCGGTCCTTTGAAACTAACAGCAATTACCCCAGAAAGTGTCTGCTAA

>p1_ind769

ATGCAAATAGACCCCGTTGCATATCATTGTTCCGAGTTGAAGTTCGTGCTTGTACCCGAAGAGTCGGGAAAGTTAT
CCCCGACGGAAATAGGATTCCATTTTCAGACTGGGCGCCGCGTACATGACAGAAGACCCCGCTCTGAGTCAGTGA
TGACGCACCAACGCAGATATCGATGTCTCACGTCTACCTTATTACTTGCCTTGATCGTCGGCACCCGATATTTGTCA
ACTGGGAATCCTCCTGGACCTATTATCGGTCCTTTGAAACTAACAGCAATTACCCCAGAAAGTGTCTGCTAA

>p1_ind1909

ATGCAAATAGACCCCGTTGCATATCATTGTTCCGAGTTGAAGTTCGTGCTTGTACCCGAAGAGTCGGGAAAGTTAT
CCCCGACGGAAATAGGATTCCATTTTCAGACTGGGCGCCGCGTACATGACAGAAGACCCCGCTCTGAGTCAGTGA
TGACGCACCAACGCAGATATCGATGTCTCACGTCTACCTTATTACTTGCCTTGATCGTCGGCACCCGATATTTGTCA
ACTGGGAATCCTCCTGGACCTATTATCGGTCCTTTGAAACTAACAGCAATTACCCCAGAAAGTGTCTGCTAA

>p1_ind1257

ATGCAAATAGACCCCGTTGCATATCATTGTTCCGAGTTGAAGTTCGTGCTTGTACCCGAAGAGTCGGGAAAGTTAT
CCCCGACGGAAATAGGATTCCATTTTCAGACTGGGCGCCGCGTACATGACAGAAGACCCCGCTCTGAGTCAGTGA
TGACGCACCAACGCAGATATCGATGTCTCACGTCTACCTAATTACTTGCCTTGATCGTCGGCACCCGATATTTGTCA
ACTGGGAATCCTCCTGGACCTATTATCGGTCCTTTGAAACTAACAGCAATTACCCCAGAAAGTGTCTGCTAA

>p1_ind1972

ATGCAAATAGACCCCGTTGCATATCATTGTTCCGAGTTGAAGTTCGTGCTTGTACCCGAAGAGTCGGGAAAGTTAT
CCCCGACGGAAATAGGATTCCATTTTCAGATTGGGTGCCGCGTACATGACAGAAGACCCCGCTCTGAGTCAGTGA
TGACGCACCAACGCAGATATCGATGTCTCACGTCTACCTTATTACTTGCCTTGATCGTCGGCACCCGATATTTGTCA
ACTGGGAATCCTCCTGGACCTATTATCGGTCCTTTGAAACTAACAGTAATTACCCCAGAAAGTGTCTGCTAA

>p1_ind1298

ATGCAAATAGACCCCGTTGCATATCATTGTTCCGAGTTGAAGTTCGTGCTTGTACCCGAAGAGTCGGGAAAGTTAT
CCCCGACGGAAATAGGATTCCATTTTCAGACTGGGCGCCGCGTACATGACAGAAGACCCCGCTCTGAGTCAGTGA

TGACGCACCAACGCAGATATCGATGTCTCACGTCTACCTTATTACTTGCCTTGATCGTCGGCACCCGATATTTGTCA
ACTGGGAATCCTCCTGGACCTATTATCGGTCCTTTGAAACTAACAGCAATTACCCCAGAAAGTGTCTGCTAA

>p1_ind44

ATGCAAATAGACCCCGTTGCATATCATTGTTCCGAGTTGAAGTTCGTGCTTGTACCCGAAGAGTCGGGAAAGTTAT
CCCCGACGGAAATAGGATTCCATTTTCAGATTGGGTGCCGCGTACATGACAGAAGACCCCGCTCTGAGTCAGTGA
TGGCGCACCAACGTAGATATCGATGTCTCACGTCTACCTTATTACTTGCCTTGATCGTCGGCACCCGATATTTGTCA
ACTGGGAATCCTCCTGGACCTATTATCGGTCCTTTGAAACTAACAGCAATTACCCCAGAAAGTGTCTGCTAA

>p1_ind1373

ATGCAAATAGACCCCGTTGCATATCATTGTTCCGAGTAGAAGTTCGTGCTTGTACCCGAAGAGTCGGGAAAGTTAT
CCCCGACGGAAATAGGATTCCATTTTCAGACTGGGCGCCGCGTACATGACAGAAGACCCCGCTCTGAGTCAGTGA
TGACGCACCAACGCAGATATCGATGTCTCACGTCTACCTTATTACTTGCCTTGATCGTCGGCACCCGATATTTGTCA
ACTGGGAATCCTCCTGGACCTATTATCGGTCCTTTGAAACTAACAGCAATTACCCCAGAAAGTGTCTGCTAA

>p1_ind2363

ATGCAAATAGACCCCGTTGCATATCATTGTTCCGAGTAGAAGTTCGTGCTTGTACCCGAAGAGTCGGGAAAGTTAT
CCCCGACGGAAATAGGATTCCATTTTCAGACTGGGCGCCGCGTACATGACAGAAGACCCCGCTCTGAGTCAGTGA
TGACGCACCAACGCAGATATCGATGTCTCACGTCTACCTTATTACTTGCCTTGATCGTCGGCACCCGATATTTGTCA
ACTGGGAATCCTCCTGGACCTATTATCGGTCCTTTGAAACTAACAGCAATTACCCCAGAAAGTGTCTGCTAA

>p1_ind134

ATGCAAATAGACCCCGTTGCATATCATTGTTCCGAGTTGAAGTTCGTGCTTGTACCCGAAGAGTCGGGAAAGTTAT
CCCCGACGGAAATAGGATTCCATTTTCAGACTGGGCGCCGCGTACATGACAGAAGACCCCGCTCTGAGTCAGTGA
TGACGCACCAACGCAGATATCGATGTCTCACGTCTACCTAATTACTTGCCTTGATCGTCGGCACCCGATATTTGTCA
ACTGGGAATCCTCCTGGACCTATTATCGGTCCTTTGAAACTAACAGCAATTACCCCAGAAAGTGTCTGCTAA

>p1_ind2890

ATGCAAATAGACCCCGTTGCATATCATTGTTCCGAGTTGAAGTTCGTGCTTGTACCCGAAGAGTCGGGAAAGTTAT
CCCCGACGGAAATAGGATTCCATTTTCAGATTGGGTGCCGCGTACATGACAGAAGACCCCGCTCTGAGTCAGTGA
TGACGCACCAACGCAGATATCGATGTCTCACGTCTACCTTATTACTTGCCTTGATCGTCGGCACCCGATATTTGTCA
ACTGGGAATCCTCCTGGACCTATTATCGGTCCTTTGAAACTAACAGCAATTACCCCAGAAAGTGTCTGCTAA

>p1_ind635

ATGCAAATAGACCCCGTTGCATATCATTGTTCCGAGTTGAAGTTCGTGCTTGTACCCGAAGAGTCGGGAAAGTTAT
CCCCGACGGAAATAGGATTCCATTTTCAGATTGGGTGCCGCGCACATGACAGAAGACCCCGCTCTGAGTCAGTGA
TGACGCACCAACGCAGATATCGATGTCTCACGTCTACCTTATTACTTGCCTTGATCGTCGGCACCCGATATTTGTCA
ACTGGGAATCCCCCTGGACCTATTATCGGTCCTTTGAAACTAACAGCAATTACCCCAGAAAGTGTCTGCTAA

>p1_ind2751

ATGCAAATAGACCCTGTTGCATATCATTGTTCCGAGTTGAAGTTCGTGCTTGTACCCGAAGAGTCGGGAAAGTTAT
CCCCGACGGAAATAGGATTCCATTTTCAGATTGGGTGCCGCGTACATGACAGAAGACCCCGCTCTGAGTCAGTGA
TGGCGCACCAACGCAAATACAGATGTCTCACGTCTACCTTAATACTTGCCTTGATCGTCGGCACCCGATATTTGTCA
ACTGGGAATCCTCCTGGACCTATTATCGGTCCTTTGAAACTAACAGCAATTACCCCAGAAAGTGTCTGCTAA

>p1_ind403

ATGAAAATAGACCCTGTTGCATATCATTGTTCCGAGTTGAAGTTCGTGCTTGTACCCGAAGAGTCGGGAAAGTTAT
CCCCGACGGAAATAGGATTCCATTTTCAGATTGGGTGCCGCGTACATGACAGAAGACCCCGTTCTGAGTCAGTGAT
GTCGCACCAACGCAGATATCGATGTCTCACGTCTACCTTATTACTTGCCTTGATCGTCGGCACCCGATATTTGTCAA
CTGGGAATCCTCCTGGACCTATTATCGGTCCTTTGAAACTAACAGCAATTACCCCAGAAAGTGTCTGCTAA

>p1_ind3604

ATGCAAATAGACCCCGTTGCATATCATTGTTCCGAGTTGAAGTTCGTGCTTGTACCCGAAGAGTCGGGAAAGTTAT
CCCCGACGGAAATAGGATTCCATTTTCAGATTGGGTGCCGCGTACATGACAGAAGACCCCGCTCTGAGTCAGTGA
TGACGCACCAACGCAGATATCGATGTCTCACGTCTACCTTATTACTTGCCTTGATCGTCGGCACCCGATATTTGTCA
ACTGGGAATCCTCCTGGACCTATTATCGGTCCTTTGAAACTAACAGCAATTACCCCAGAAAGTGTCTGCTAA

>p1_ind4740

ATGCAAATAGACCCCGTTGCATATCATTGTTCCGAGTTGAAGTTCGTGCTTGTACCCGAAGAGTCGGGAAAGTTAT
CCCCGACGGAAATAGGATTCCATTTTCAGATTGGGTGCCGCGTACATGACAGAAGACCCCGCTCTGAGTCAGTGA
TGGCGCACCAACGCAAATATCGATGTCTCACGTCTACCTTATTACTTGCCTTGATCGTCGGCACCCGATATTTGTCA
ACTGGGAATCCTCCTGGACCTATTATCGGTCCTTTGAAACTAACAGCAATTACCCCAGAAAGTGTCTGCTAA

>p1_ind3401

ATGCAAATAGACCCCGTTGCATATCATTGTTCCGAGTTGAAGTTCGTGCTTGTACCCGAAGAGTCGGGAAAGTTAT
CCCCGACGGAAATAGGATTCCATTTTCAGACTGGGCGCCGCGTACATGACAGAAGACCCCGCTCTGAGTCAGTGA
TGACGCACCAACGCAGATATCGATGTCTCACGTCTACCTTATTACTTGCCTTGATCGTCGGCACCCGATATTTGTCA
ACTGGGAATCCTCCTGGACCTATTATCGGTCCTTTGAAACTAACAGCAATTACCCCAGAAAGTGTCTGCTAA

>p1_ind4082

ATGCAAATAGACCCCGTTGCATATCATTGTTCCGAGTTGAAGTTCGTGCTTGTACCCGAAGAGTCGGGAAAGTTAT
CCCCGACGGAAATAGGATTCCATTTTCAGACTGGGCGCCGCGTACATGACAGAAGACCCCGCTCTGAGTCAGTGA
TGACGCACCAACGCAGATATCGATGTCTCACGTCTACCTTATTACTTGCCTTGATCGTCGGCACCCGATATTTGTCA
ACTGGGAATCCTCCTGGACCTATTATCGGTCCTTTGAAACTAACAGCAATTACCCCAGAAAGTGTCTGCTAA

>p1_ind2304

ATGCAAATAGACCCCGTTGCATATCATTGTTCCGAGTTGAAGTTCGTGCTTGTACCCGAAGAGACGGGAAAGTTAT
CCCCGACGGAAATAGGATTCCATTTTCAGATTGGGTGCCGCGTACATGACAGAAGACCCCGCTCTGAGTCAGTGA

TGACGCACCAACGCAGATATCGATGTCTCACGTCTACCTTATTACTTGCCTTGATCGTCGGCACCCGATATTTGTCA
ACTGGGAATCCTCCTGGACCTATTATCGGTCCTTTGAAACTAACAGCAATTACCCCAGAAAGTGTCTGCTAA

>p1_ind2904

ATGCAAATAGACCCCGTTGCATATCATTGTTCCGAGTTGAAGTTCGTGCTTGTACCCGAAGAGTCGGGAAAGTTAT
CCCCGACGGAAATAGGATTCCATTTTCAGATTGGGTGCCGCGTACATGACAGAAGACCCCGCTCTGAGTCAGTGA
TGACGCACCAACGCAGATATCGATGTCTCACGTCTACCTTATTACTTGCCTTGATCGTCGGCACCCGATATTTGTCA
ACTGGGAATCCTCCTGGACCTATTATCGGTCCTTTGAAACTAACAGCAATTACCCCAGAAAGTGTCTGCTAA

>p1_ind2877

ATGCAAATAGACCCCGTTGCATATCATTGTTCCGAGTTGAAGTTCGTGCTTGTACCCGAAGAGTCGGGAAAGTTAT
CCCCGACGGAAATAGGATTCCATTTTCAGATTGGGCGCCGCGTACATGACAGAAGACCCCGCTCTGAGTCAGTGA
TGACGCACCAACGCAGATATCGATGTCTCACGTCTACCTTATTACTTGCCTTGATCGTCGGCACCCGATATTTGTCA
ACTGGGAATCCTCCTGGACCTATTATCGGTCCTTTGAAACTAACAGCAATTACCCCAGAAAGTGTCTGCTAA

>p1_ind504

ATGCAAATAGACCCTGTTGCATATCATTGTTCCGAGTTGAAGTTCGTGCTTGTACCCGAAGAGTCGGGAAAGTTAT
CCCCGACGGAAATAGGATTACATTTTCAGATTGGGCGCCGCGTACATGACAGAAGACCCCGCTCTGAGTCAGTGA
TGACGCACCAACGCAGATATCGATGTCTCACGTCTACCTTATTACTTGCCTTGATCGTCGGCACCCGATATTTGTCA
ACTGGGAATCCTCCTGGACCTATTATCGGTCCTTTGAAACTAACAGCAATTACCCCAGAAAGTGTCTGCTAA

>p1_ind1193

ATGCAAATAGACCCCGTTGCATATCATTGTTCAGAGTTGAAGTTCGTGCTTGTACCCGAAGAGTCGGGAAAGTTAT
CCCCGACGGAAATAGGATTCCATTTTCAGATTGGGCGCCGCGTACATGACAGAAGACCCCGCTCTGAGTCAGTGA
TGACGCACCAACGCAGATATCGATGTCTCACGTCTACCTTATTACTTGCCTTGATCGTCGGCACCCGATATTTGTCA
ACTGGGAATCCTCCTGGACCTATTATCGGTCCTTTGAAACTAACAGCAATTACCCCAGAAAGTGTCTGCTGA

>p1_ind3164

ATGCAAATAGACCCCGTTGCATATCATTGTTCCGAGTAGAAGTTCGTGCTTGTACCCGAAGAGTCGGGAAAGTTAT
CCCCGACGGAAATAGGATTCCATTTTCAGACTGGGCGCCGCGTACATGACAGAAGACCCCGCTCTGAGTCAGTGA
TGACGCACCAACGCAGATATCGATGTCTCACGTCTACCTTATTACTTGCCTTGATCGTCGGCACCCGATATTTGTCA
ACTGGGAATCCTCCTGGACCTATTATCGGTCCTTTGAAACTAACAGCAATTACCCCAGAAAGTGTCTGCTAA

>p1_ind4344

ATGCAAATAGACCCCGTTGCATATCATTGTTCCGAGTTGAAGTTCGTGCTTGTACCCGAAGAGTCGGGAAAGTTAT
CCCCGACGGAAATAGGATTCCATTTTCAGACTGGGCGCCGCGTACATGACAGAAGACCCCGCTCTGAGTCAGTGA
TGCCGCACCAACGCAGATATCGATGTCTCACGTCTACCTTATTACTTGCCTTGATCGTCGGCACCCGATATTTGTCA
ACTGGGAATCCTCCTGGACCTATTATCGGTCCTTTGAAACTAACAGCAATTACCCCAGAAAGTGTCTGCTAA

>p1_ind314

ATGCAAATAGACCCTGTTGCATATCATTGTTCCGAGTTGAAGTTCGTGCTTGTACCCGAAGAGTCGGGAAAGTTAT
CCCCGACGGAAATAGGATTACATTTTCAGATTGGGCGCCGCGTACATGACAGAAGACCCCGCTCTGAGTCAGTGA
TGACGCACCAACGCAGATATCGATGTCTCACGTCTACCTTATTACTTGCCTTGATCGTCGGCACCCGATATTTGTCA
ACTGGGAATCCTCCTGGACCTATTATCGGTCCTTTGAAACTAACAGCAATTACCCCAGAAAGTGTCTGCTAA

>p1_ind1615

ATGCAAATAGACCCCGTTGCATATCATTGTTCCGAGTTGAAGTTCGTGCTTGTACCCGAAGAGTCGGGAAAGTTAT
CCCCGACGGAAATAGGATTCCATTTTCAGATTGGGCGCCGCGTACATGACAGAAGACCCCGCTCTGAGTCAGTGA
TGACGCACCAACGCAGATATCGATGTCTCACGTCTACCTTATTACTTGCCTTGATCGTCGGCACCCGATATTTGTCA
ACTGGGAATCCTCCTGGACCTATTATCGGTCCTTTGAAACTAACAGCAATTACCCCAGAAAGTGTCTGCTAA

>p1_ind2402

AAGCAAATAGACCCCGTTGCATATCATTGTTCCGAGTTGAAGTTCGTGCTTGTACCCGAAGAGTCGGGAAAGTTAT
CCCCGACGGAAATAGGATTCCATTTTCAGACTGGGCGCCGCGTACATGACAGCAGACCCCGCTCTGAGTCAGTGA
TGACGCACCAACGCAGATATCGATGTCTCACGTCTACCTTATTACTTGCCTTGATCGTCGGCACCCGATATTTGTCA
ACTGGGAATCCTCCTGGACCTATTATCGGTCCTTTGAAACTAACAGCAATTACCCCAGAAAGTGTCTGCTAA

>p1_ind1606

ATGCAAATAGACCCCGTTGCATATCATTGTTCCGAGTTGAAGTTCGTGCTTGTACCCGAAGAGTCGGGAAAGTTAT
CCCCGACGGAAATAGGATTCCATTTTCAGACTGGGCGCCGCGTACATGACAGAAGACCCCGCTCTGAGTCAGTGA
TGACGCACCAACGCAGATATCGATGTCTCACGTCTACCTTATTACTTGCCTTGATCGTCGGCACCCGATATTTGTCA
ACTGGGAATCCTCCTGGACCTATTCTCGGTCCTTTGAAACTAACAGCAATTACCCCAGAAAGTGTCTGCTAA

>p1_ind2661

ATGCAAATAGACCCCGTTGCATATCATTGTTCCGAGTAGAAGTTCGTGCTTGTACCCGAAGAGTCGGGAAAGTTAT
CCCCGACGGAAATAGGATTCCATTTTCAGACTGGGCGCCGCGTACATGACAGAAGACCCCGCTCTGAGTCAGTGA
TGACGCACCAACGCAGATATCGATGTCTCACGTCTACCTTATTACTTGCCTTGATCGTCGGCACCCGATATTTGTCA
ACTGGGAATCCTCCTGGACCTATTATCGGTCCTTTGAAACTAACAGCAATTACCCCAGAAAGTGTCTGCTAA

>p1_ind391

ATGCAAATAGACCCCGTTGCATATCATTGTTCCGAGTTGAAGTTCGTGCTTGTACCCGAAGAGTCGGGAAAGTTAT
CCCCGACGGAAATAGGATTCCATTTTGAGATTGGGTGCCGCGTACATGACAGAAGACCCCGCTCTGAGTCAGTGA
TGGCGCACCAACGCAAATATCGATGTCTCACGTCTACCTTATTACTTGCCTTGATCGTCGGCACCCGATATTTGTCA
ACTGGGAATCCTCCTGGACCTATTATCGGTCCTTTGAAACTAACAGCAATTACCCCAGAAAGTGTCTGCTAA

>p1_ind2279

ATGCAAATAGACCCCGTTGCATATCATTGTTCCGAGTTGAAGTTCGTGCTTGTACCCGAAGAGTCGGGAAAGTTAT
CCCCGACGGAAATAGGATTCCATTTTCAGACTGGGCGCCGCGTACATGACAGAAGACCCCGCTCTGAGTCAGTGA

TGACGCACCAACGCAGATATCGATGTCTCACGTCTACCTTATTACTTGCCTTGATCGTCGGCACCCGACATTTGTCA
ACTGGGAATCCTCCTGGACCTATTATCGGTCCTTTGAAACTAACAGCAATTACCCCAGAAAGTGTCTGCTAA

>p1_ind2102

ATGCAAATAGACCCCGTTGCATATCATTGTTCCGAGTTGAAGTTCGTGCTTGTACCCGAAGAGTCGGGAAAGTTAT
CCCCGACGGAAATAGGATTCCATTTTCAGATTGGGCGCCGCGTACATGACAGAAGACCCCGCTCTGAGTCAGTGA
TGACGCACCAACGCAGATATCGATGTCTCACGTCTACCTTATTACTTGCCTTGATCGTCGGCACCCGATATTTGTCA
ACTGGGAATCCTCCTGGACCTATTATCGGTCCTTTGAAACTAACAGCAATTACCCCAGAAAGTGTCTGCTAA

>p1_ind5080

AAGCAAATAGACCCCGTTGCATATCATTGTTCCGAGTTGAAGTTCGTGCTTGTACCCGAAGAGTCGGGAAAGTTAT
CCCCGACGGAAATAGGATTCCATTTTCAGACTGGGCGCCGCGTACATGACAGCAGACCCCGCTCTGAGTCAGTGA
TGACGCACCAACGCAGATATCGATGTCTCACGTCTACCTTATTACTTGCCTTGATCGTCGGCACCCGATATTTGTCA
ACTGGGAATCCTCCTGGACCTATTATCGGTCCTTTGAAACTAACAGCAATTACCCCAGAAAGTGTCTGCTAA

>p1_ind244

ATGCAAATAGACCCCGTTGCATATCATTGTTCCGAGTTGAAGTTCGTGCTTGTACCCGAAGAGTCGGGAAAGTTAT
CCCCGACGGAAATAGGATTCCATTTTCAGACTGGGCGCCGCGTACATGACAGAAGACCCCGCTCTGAGTCAGTGA
TGACGCTCCAACGCAGATATCGATGTCTCACGTCTACCTTATTACTTGCCTTGATCGTCGGCACCCGATATTTGTCA
ACTGGGAATCCTCCTGGACCTATTATCGGTCCTTTGAAACTAACAGCAATTACCCCAGAAAGTGTCTGCTAA

>p1_ind3646

ATGCAAATAGACCCCGTTGCATATCATTGTTCCGAGTTGAAGTTCGTGCTTGTACCCGAAGAGTCGGGAAAGTTAT
CCCCGACGGAAATAGGATTCCATTTTCAGATTGGGTGCCGCGTACATGACACAAGACCCCGCTCTGAGTCAGTGAT
GACGCACCAACGCAGATATCGATGTCTCACGTGTACCTTATTACTTGCCTTGATCGTCGGCACCCGATATTTGTCAA
CTGGGAATCCTCCTGGACCTATTATCGGTCCTTTGAAACTAACAGCAATTACCCCAGAAAGTGTCTGCTAA

>p1_ind4019

ATGCAAATAGACCCCGTTGCATATCATTGTTCCGAGTAGAAGTTCGTGCTTGTACCCGAAGAGTCGGGAAAGTTAT
CCCCGACGGAAATAGGATTCCATTTTCAGACTGGGCGCCGCGTACATGACAGAAGACCCCGCTCTGAGTCAGTGA
TGACGCACCAACGCAGATATCGATGTCTCACGTCTACCTTATTACTTGCCTTGATCGTCGGCACCCGATATTTGTCA
ACTGGGAATCCTCCTGGACCTATTATCGGTCCTTTGAAACTAACAGCAATTACCCCAGAAAGTGTCTGCTAA

>p1_ind665

ATGCAAATAGACCCCGTTGCATATCATTGTTCCGAGTTGAAGTTCGTGCTTGTACCCGAAGAGTCGGGAAAGTTAT
CCCCGACGGAAATAGGATTCCATTTTCAGACTGGGCGCCGCGTACATGACAGAAGACCCCGCTCTGAGTCAGTGA
TGACGCACCAACGCAGATATCGATGTCTCACGTCTACCTTATTACTTGCCTTGATCGTCGGCACCCGATATTTGTCA
ACTGGGAATCCTCCTGGACCTATTATCGGTCCTTTGAAACTAACAGCAATTACCCCAGAAAGTGTCTGCTAA

>p1_ind41

ATGCAAATAGACCCCGTTGCATATCATTGATCCGAGTTGAAGTTCGTGCTTGTACCCGAAGAGTCGGGAAAGTTAT
CCCCGACGGAAATAGGATTCCATTTTCAGACTGGGCGCCGCGTACATGACAGAAGACCCCGCTCTGAGTCAGTGA
TGACGCACCAACGCAGATATCGATGTCTCACGTCTACCTTATTACTTGCCTTGATCGTCGGCACCCGATATTTGTCA
ACTGGGAATCCTCCTGGACCTATTATCGGTCCTTTGAAACTAACAGCAATTACCCCAGAAAGTGTCTGCTAA

>p1_ind3098

ATGCAAATAGACCCCGTTGCATATCATTGTTCCGAGTTGAAGTTCGTGCTTGTACCCGAAGAGTCGGGAAAGTTAT
CCCCGACGGAAATAGGATTCCATTTTCAGACTGGGCGCCGCGTACATGACAGAAGACCCCGCTCTGAGTCAGTGA
TGACGCACCAACGCAGATATCGATGTCTCACGTCTACCTTATTACTTGCCTTGATCGTCGGCACCCGATATTTGTCA
ACTGGGAATCCTCCTGGACCTATTATCGGTCCTTTGAAACTAACAGCAATTACCCCAGAAAGTGTCTGCTAA

>p1_ind3788

ATGCAAATAGACCCCGTTGCATATCATTGTTCCGAGTTGAAGTTCGTGCTTGTACCCGAAGAGTCGGGAAAGTTAT
CCCCGACGGAAATAGGATTCCATTTTCAGACTGGGCGCCGCGTACATGACAGAAGACCCCGCTCTGAGTCAGTGA
TGACGCACCAACGCAGATATCGATGTCTCACGTCTACCTTATTACTTGCCTTGATCGTCGGCACCCGATATTTGTCA
ACTGGGAATCCTCCTGGACCTATTATCGGTCCTTTGAAACTAACAGCAATTACCCCAGAAAGTGTCTGCTAA

>p1_ind4098

AAGCAAATAGACCCCGTTGCATATCATTGTTCCGAGTTGAAGTTCGTGCTTGTACCCGAAGAGTCGGGAAAGTTAT
CCCCGACGGAAATAGGATTCCATTTTCAGACTGGGCGCCGCGTACATGACAGAAGACCCCGCTCTGAGTCAGTGA
TGACGCACCAACGCAGATATCGATGTCTCACGTCTACCTTATTACTTGCCTTGATCGTCGGCACCCGATATTTGTCA
ACTGGGAATCCTCCTGGACCTATTATCGGTCCTTTGAAACTAACAGCAATTACCCCAGAAAGTGTCTGCTAA

>p1_ind4103

ATGCAAATAGACCCCGTTGCATATCATTGTTCCGAGTTGAAGTTCGTGCTTGTACCCGAAGAGTCGGGAAAGTTAT
CCCCGACGGAAATAGGATTCCATTTTCAGATTGGGTGCCGCGTACATGACAGAAGACCCCGCTCTGAGTCAGTGA
TGACGCACCAACGCAGATATCGATGTCTCACGTCTACCTTATTACTTGCCTTGATCGTCGGCACCCGATATTTGTCA
ACTGGGAATCCTCCTGGACCTATTATCGGTCCTTTGAAACTAACAGCAATTACCCCAGAAAGTGTCTGCTAA

>p1_ind3089

ATGCAAATAGACCCCGTTGCATATCATTGTTCCGAGTTGAAGTTCGTGCTTGTACCCGAAGAGTCGGGAAAGTTAT
CCCCGACGGAAATAGGATTCCATTTTCAGATTGGGTGCCGCGTACATGACAGAAGACCCCGTTCTGAGTCAGTGAT
GACGCACCAACGCAGATATCGATGTCTCACGTCTACCTTATTACTTGCCTTGATCGTCGGCACCCGATATTTGTCAA
CTGGGAATCCTCCTGGACCTATTATCGGTCCTTTGAAACTAACAGGAATTACCCCAGAAAGTGTCTGCTAA

>p1_ind3260

ATGCAAATAGACCCCGTTGCATATCATTGTTCCGAGTTGAAGTTCGTGCTTGTACCCGAAGAGTCGGGAAAGTTAT
CCCCGACGGAAATAGGATTCCATTTTCAGACTGGGCGCCGCGTACATGACAGAAGACCCCGCTCTGAGTCAGTGA

TGACGCACCAACGCAGATATCGATGTCTCACGTCTACCTTATTACTTGCCTTGATCGTCGGCACCCGATATTTGTCA
ACTGGGAATCCTCCTGGACCTATTATCGGTCCTTTGAAACTAACAGCAATTACCCCAGAAAGTGTCTGCTAA

>p1_ind69

ATGCAAATAGACCCCGTTGCATATCATTGTTCCGAGTTGAAGTTCGTGCTTGTACCCGAAGAGTCGGGAAAGTTAT
CCCCGACGGAAATAGGATTCCATTTTCAGATTGGGTGCCGCGTACATGACAGAAGACCCCGCTCTGAGTCAGTGA
TGGCGCACCAACGCAAATATCGATGTCTCACGTCTACCTTATTACTTGCCTTGATCGTCGGCACCCGATATTTGTCA
ACTGGGAATCCTCCTGGACCTATTATCGGTCCTTTGAAACTAACAGCAATTACCCCAGAAAGTGTCTGCTAA

>p1_ind4485

ATGCAAATAGACCCCGTTGCATATCATTGTTCCGAGTTGAAGTTCGTGCTTGTACCCGAAGAGTCGGGAAAGTTAT
CCCCGACGGAAATAGGATTCCATTTTCAGATTGGACGCCGCGTACATGACAGAAGACCCCGCTCTGAGTCAGTGA
TGACGCACCAACGCAGATATCGATGTCTCACGTCTACCTTATTACTTGCCTTGATCGTCGGCACCCGATATTTGTCA
ACTGGGAATCCTCCTGGACCTATTATCGGTCCTTTGAAACTAACAGCAATTACCCCAGAAAGTGTCTGCTAA

>p1_ind79

ATGCAAATAGACCCCGTTGCATATCATTGTTCCGAGTTGAAGTTCGTGCTTGTACCCGAAGAGTCGGGAAAGTTAT
CCCCGACGGAAATAGGATTCCATTTTCAGACTGGGCGCCGCGTACATGACAGAAGACCCCGCTCTGAGTCAGTGA
TGACGCACCAACGCAGATATCGATGTCTCACGTCTACCTTATTACTTGCCTTGATCGTCGGCACCCGATATTTGTCA
ACTGGGAATCCTCCTGGACCTATTCTCGGTCCTTTGAAACTAACAGCAATTACCCCAGAAAGTGTCTGCTAA

>p1_ind4116

ATGCAAATAGACCCCGTTGCATATCATTGTTCCGAGTTGAAGTTCGTGCTTGTACCCGAAGAGTCGGGAAAGTTAT
CCCCGACGGAAATAGGATTCCATTTTCAGATTGGGTGCCGCGCACATGACAGAAGACCCCGCTCTGAGTCAGTGA
TGACGCTCCAACGCAGATATCGATGTCTCACGTCTACCTTATTACTTGCCTTGATCGTCGGCACCCGATATTTGTCA
ACTGGGAATCCTCCTGGACCTATTATCGGTCCTTTGAAACTAACAGCAATTACCCCAGAAAGTGTCTGCTAA

>p1_ind934

ATGCAAATAGACCCCGTTGCATATCATTGTTCCGAGTTGAAGTTCGTGCTTGTACCCGAAGAGTCGGGAAAGTTAT
CCCCGACGGAAATAGGATTCCATTTTCAGACTGGGCGCCGCGTACATGACAGAAGACCCCGCTCTGAGTCAGTGA
TGACGCACCAACGCAGATATCGATGTCTCACGTCTACCTTATTACTTGCCTTGATCGTCGGCACCCGATATTTGTCA
ACTGGGAATCCTCCTGGACCTATTATCGGTCCTTTGAAACTAACAGCAATTACCCCAGAAAGTGTCTGCTAA

>p1_ind2235

ATGCAAATAGACCCCGTTGCATATCATTGTTCCGAGTAGAAGTTCGTGCTTGTACCCGAAGAGTCGGGAAAGTTAT
CCCCGACGGAAATAGGATTCCATTTTCAGACTGGGCGCCGCGTACATGACAGAAGACCCCGCTCTGAGTCAGTGA
TGACGCACCAACGCAGATATCGATGTCTCACGTCTACCTTATTACTTGCCTTGATCGTCGGCACCCGATATTTGTCA
ACTGGGAATCCTCCTGGACCTATTATCGGTCCTTTGAAACTAACAGCAATTACCCCAGAAAGTGTCTGCTAA

>p1_ind2073

ATGCAAATAGACCCCGTTGCATATCATTGTTCCGAGTTGAAGTTCGTGCTTGTACCCGAAGAGTCGGGAAAGTTAT
CCCCGACGGAAATAGGATTCCATTTTCAGATTGGGTGCCGCGTACATGACAGAAGACCCCGCTCTGAGTCAGTGA
TGACGCACCAACGCAGATATCGATGTCTCACGTCTACCTTATTACTTGCCTTGATCGTCGGCACCCGATATTTGTCA
ACTGGGAATCCTCCTGGACCTATTATCGGTCCTTTGAAACTAACAGCAATTACCCCAGAAAGTGTCTGCTAA

>p1_ind994

ATGCAAATAGACCCCGTTGCATATCATTGTTCCGAGTTGAAGTTCGTGCTTGTACCCGAAGAGTCGGGAAAGTTAT
CCCCGACGGAAATAGGATTCCATTTTCAGATTGGGTGCCGCGTACATGACAGAAGACCCCGCTCTGAGTCAGTGA
TGACGCACCAACGCAGATATCGATGTCTCACGTCTACCTTATTACTTGCCTTGATCGTCGGCACCCGATATTTGTCA
ACTGGGAATCCTCCTGGACCTATTATCGGTCCTTTGAAACTAACAGCAATTACCCCAGAAAGTGTCTGCTAA

>p1_ind4346

ATGCAAATAGCCCCCGTTGCATATCATTGTTCCGAGTTGAAGTTCGTGCTTGTACCCGAAGAGTCGGGAAAGTTAT
CCCCGACGGAAATAGGATTCCATTTTCAGATTGGACGCCGCGTACATGACAGAAGACCCCGCTCTGAGTCAGTGA
TGACGCACCAACGCAGATATCGATGTCTCACGTCTACCTTATTACTTGCCTTGATCGTCGGCACCCGATATTTGTCA
ACTGGGAATCCTCCTGGACCTATTATCGGTCCTTTGAAACTAACAGCAATTACCCCAGAAAGTGTCTGCTAA

>p1_ind404

ATGCAAATAGACCCCGTTGCATATCATTGTTCCGAGTTGAAGTTCGTGCTTGTACCCGAAGAGTCGGGAAAGTTAT
CCCCGACGGAAATAGGATTCCATTTTCAGACTGGGCGCCGCGTACATGACAGAAGACCCCGCTCTGAGTCAGTGA
TGACGCACCAACGCAGATATCGATGTCTCACGTCTACCTTATTACTTGCCTTGATCGTCGGCACCCGATATTTGTCA
ACTGGGAATCCTCCTGGACCTATTATCGGTCCTTTGAAACTAACAGCAATTACCCCAGAAAGTGTCTGCTAA

>p1_ind2560

ATGCAAATAGACCCCGTTGCATATCATTGTTCCGAGTTGAAGTTCGTGCTTGTACCCGAAGAGTCGGGAAAGTTAT
CCCCGACGGAAATAGGATTCCATTTTCAGATTGGACGCCGCGTACATGACAGAAGACCCCGCTCTGAGTCAGTGA
TGACGCACCAACGCAGATATCGATGTCTCACGTCTACCTTATTACTTGCCTTGATCGTCGGCACCCGATATTTGTCA
ACTGGGAATCCTCCTGGACCTATTATCGGTCCTTTGAAACTAACAGCAATTACCCCAGAAAGTGTCTGCTAA

>p1_ind4881

ATGCAAATAGACCCTGTTGCATATCATTGTTCCGAGTTGAAGTTCGTGCTTGTACCCGAAGAGTCGGGAAAGTTAT
CCCCGACGGAAATAGGATTCCATTTTCAGATTGGGTGCCGCGTACATGACAGAAGACCCCGCTCTGAGTCAGTGA
TGGCGCACCAACGCAAATACAGATGTCTCACGTCTACCTTATTACTTGCCTTGATCGTCGGCACCCGATATTTGTCA
ACTGGGAATCCTCCTGGACCTATTATCGGTCCTTTGAAACTAACAGCAATTACCCCAGAAAGTGTCTGCTAA

>p1_ind939

ATGCAAATAGACCCCGTTGCATATCATTGTTCCGAGTTGAAGTTCGTGCTTGTACCCGAAGAGTCGGGAAAGTTAT
CCCCGACGGAAATAGGATTCCATTTTCAGATTGGGTGCCGCGTACATGACACAAGACCCCGCTCTGAGTCAGTGAT

GACGCACCAACGCAGATATCGATGTCTCACGTGTACCTTATTACTTGCCTTGATCGTCGGCACCCGATATTTGTCAA
CTGGGAATCCTCCTGGACCTATTATCGGTCCTTTGAAACTAACAGCAATTACCCCAGAAAGTGTCTGCTAA

>p1_ind4509

ATGCAAATAGACCCCGTTGCATATCATTGTTCCGAGTAGAAGTTCGTGCTTGTACCCGAAGAGTCGGGAAAGTTAT
CCCCGACGGAAATAGGATTCCATTTTCAGACTGGGCGCCGCGTACATGACAGAAGACCCCGCTCTGAGTCAGTGA
TGACGCACCTACGCAGATATCGATGTCTCACGTCTACCTTATTACTTGCCTTGATCGTCGGCACCCGATATTTGTCA
ACTGGGAATCCTCCTGGACCTATTATCGGTCCTTTGAAACTAACAGCAATTACCCCAGAAAGTGTCTGCTAA

>p1_ind4604

ATGCAAATAGACCCCGTTGCATATCATTGTTCCGAGTTGAAGTTCGTGCTTGTACCCGAAGAGTCGGGAAAGTTAT
CCCCGACGGAAATAGGATTCCATTTTCAGATTGGGTGCCGCGTACATGACAGCAGACCCCGCTCTGAGTCAGTGAT
GGCGCACCAACGCAAATATCGATGTCTCACGTCTACCTTATTACTTGCCTTGATCGTCGGCACCCGATTTTTGTCAA
CTGGGAATCCTCCTGGACCTATTATCGGTCCTTTGAAACTAACAGCAATTACCCCAGAAAGTGTCTGCTAA

>p1_ind4031

ATGCAAATAGACCCCGTTGCATATCATTGTTCCGAGTTGAAGTTCGTGCTTGTACCCGAAGAGTCGGGAAAGTTAT
CCCCGACGGAAATAGGATTCCATTTTCAGACTGGGCGCCGCGTACATGACAGAAGACCCCGCTCTGAGTCAGTGA
TGACGCACCAACGCAGATATCGATGTCTCACGTCTACCTTATTACTTGCCTTGATCGTCGGCACCCGATATTTGTCA
ACTGGGAATCCTCCTGGACCTATTATCGGTCCTTTGAAACTAACAGCAATTACCCCAGAAAGTGTCTGCTAA

>p1_ind4992

ATGCAAATAGCCCCCGTTGCATATCATTGTTCCGAGTTGAAGTTCGTGCTTGTACCCGAAGAGTCGGGAAAGTTAT
CCCCGACGGAAATAGGATTCCATTTTCAGATTGGACGCCGCGTACATGACAGAAGACCCCGCTCTGAGTCAGTGA
TGACGCACCAACGCAGATATCGATGTCTCACGTCTACCTTATTACTTGCCTTGATCGTCGGCACCCGATATTTGTCA
ACTGGGAATCCTCCTGGACCTATTATCGGTCCTTTGAAACTAACAGCAATTACCCCAGAAAGTGTCTGCTAA

>p1_ind1632

ATGCAAATAGACCCCGTTGCATATCATTGTTCCGAGTTGAAGTTCGTGCTTGTACCCGAAGAGTCGGGAAAGTTAT
CCCCGACGGAAATAGGATTCCATTTTCAGATTGGGTGCCGCGTACATGACAGAAGACCCCGCTCTGAGTCAGTGA
TGACGCACCAACGCAGATATCGATGTCTCACGTCTACCTTATTACTTGCCTTGATCGTCGGCACCCGATATTTGTCA
ACTGGGAATCCTCCTGGACCTATTATCGGTCCTTTGAAACTAACAGCAATTACCCCAGAAAGTGTCTGCTAA

>p1_ind3021

ATGCAAATAGACCCCGTTGCATATCATTGTTCCGAGTTGAAGTTCGTGCTTGTACCCGAAGAGTCGGGAAAGTTAT
CCCCGACGGAAATAGGATTCCATTTTCAGACTGGGCGCCGCGTACATGACAGAAGACCCCGCTCTGAGTCAGTGA
TGACGCACCAACGCAGATATCGATGTCTCACGTCTACCTTATTACTTGCCTTGATCGTCGGCACCCGATATTTGTCA
ACTGGGAATCCTCCTGGACCTATTATCGGTCCTTTGAAACTAACAGCAATTACCCCAGAAAGTGTCTGCTAA

>p1_ind3237

ATGCAAATAGACCCCGTTGCATATCATTGTTCCGAGTTGAAGTTCGTGCTTGTACCCGAAGAGTCGGGAAAGTTAT
CCCCGACGGAAATAGGATTACATTTTCAGATTGGGCGCCGCGTACATGACAGAAGACCCCGCTCTGAGTCAGTGA
TGACGCACCAACGCAGATATCGATGTCTCACGTCTACCTTATTACTTGCCTTGATCGTCGGCACCCGATATTTGTCA
ACTGGGAATCCTCCTGGACCTATTATCGGTCCTTTGAAACTAACAGCAATTACCCCAGAAAGTGTCTGCTAA

>p1_ind3852

ATGCAAATAGACCCCGTTGCATATCATTGTTCCGAGTTGAAGTTCGTGCTTGTACCCGAAGAGTCGGGAAAGTTAT
CCCCGACGGAAATAGGATTCCATTTTCAGATTGGGTGCCGCGTACATGACAGAAGACCCCGCTCTGAGTCAGTGA
TGACGCACCAACGCAGATATCGATGTCTCACGTCTACCTTATTACTTGCCTTGATCGTCGGCACCCGATATTTGTCA
ACTGGGAATCCTCCTGGACCTATTATCGGTCCTTTGAAACTAACAGCAATTACCCCAGAAAGTGTCTGCTAA

>p1_ind4957

ATGCAAATAGACCCCGTTGCATATCATTGTTCCGAGTTGAAGTTCGTGCTTGTACCCGAAGAGTCGGGAAAGTTAT
CCCCGACGGAAATAGGATTCCATTTTCAGATTGGGTGCCGCGTACATGACAGAAGACCCCGCTCTGAGTCAGTGA
TGACGCACCAACGCAGATATCGATGTCTCACGTCTACCTTATTACTTGCCTTGATCGTCGGCACCCGGTATTTGTCA
ACTGGGAATCCTCCTGGACCTATTATCGGTCCTTTGAAACTAACAGCAATTACTCCAGAAAGTGTCTGCTAA

>p1_ind3616

ATGCAAATAGACCCCGTTGCATATCATTGTTCCGAGTTGAAGTTCGTGCTTGTACCCGAAGAGTCGGGAAAGTTAT
CCCCGACGGAAATAGGATTCCATTTTCAGATTGGGCGCCGCGTACATGACAGAAGACCCCGCTCTGAGTCAGTGA
TGACGCACCAACGCAGATATCGATGTCTCACGTCTACCTTATTACTTGCCTTGATCGTCGGCACCCGATATTTGTCA
ACTGGGAATCCTCCTGGACCTATTATCGGTCCTTTGAAACTAACAGCAATTACCCCAGAAAGTGTCTGCTAA

>p1_ind2520

ATGCAAATAGACCCCGTTGCATATCATTGTTCCGAGTTGAAGTTCGTGCTTGTACCCGAAGAGTCGGGAAAGTTAT
CCCCGACGGAAATAGGATTCCATTTTCAGATTGGGTGCCGCGTACATGACAGAAGACCCCGCTCTGAGTCAGTGA
TGGCGCACCAACGCAAATATCGATGTCTCACGTCTACCTTATTACTTGCCTTGATCGTCGGCACCCGATATTTGTCA
ACTGGGATTCCTCCTGGACCTATTATCGGTCCTTTGAAACTAACAGCAATTACCCCAGAAAGTGTCTGCTAA

>p1_ind5074

ATGCAAATAGACCCCGTTGCATATCATTGTTCCGAGTTGAAGTTCGTGCTTGTACCCGAAGAGTCGGGAAAGTTAT
CCCCGACGGAAATAGGATTCCATTTTCAGACTGGGCGCCGCGTACATGACAGAAGACCCCGCTCTGAGTCAGTGA
TGACGCACCAACGCAGATATCGATGTCTCACGTCTACCTTATTACTTGCCTTGATCGTCGGCACCCGATATTTGTCA
ACTGGGAATCCTCCTGGACCTATTATCGGTCCTTTGAAACTAACAGCAATTACCCCAGAAAGTGTCTGCTAA

>p1_ind660

ATGCAAATAGACCCCGTTGCATATCATTGTTCCGAGTAGAAGTTCGTGCTTGTACCCGAAGAGTCGGGAAAGTTAT
CCCCGACGGAAATAGGATTCCATTTTCAGACTGGGCGCCGCGTACATGACAGAAGACCCCGCTCTGAGTCAGTGA

TGACGCACCAACGCAGATATCGATGTCTCACGTCTACCTTATTACTTGCCTTGATCGTCGGCACCCGATATTTGTCA
ACTGGGAATCCTCCTGGACCTATTATCGGTCCTTTGAAACTAACAGCAATTACCCCAGAAAGTGGCTGCTAA

>p1_ind3769

ATGCAAATAGACCCCGTTGCATATCATTGTTCCGAGTTGAAGTTCGTGCTTGTACCCGAAGAGTCGGGAAAGTTAT
CCCCGACGGAAATAGGATTCCATTTTCAGATTGGGTGCCGCGCACATGACAGAAGACCCCGCTCTGAGTCAGTGA
TGACGCACCAACGCAGATATCGATGTCTCACGTCTACCTTATTACTTGCCTTGATCGTCGGCACCCGATATTTGTCA
ACTGGGAATCCTCCTGGACCTATTATCGGTCCTTTGAAACTAACAGGAATTACCCCAGAAAGTGTCTGCTAA

>p1_ind3571

ATGCAAATAGACCCCGTTGCATATCATTGTTCCGAGTTGAAGTTCGTGCTTGTACCCGAAGAGTCGGGAAAGTTAT
CCCCGACGGAAATAGGATTCCATTTTCAGATTGGGTGCCGCGCACATGACAGAAGACCCCGCTCTGAGTCAGTGA
TGACGCACCAACGCAGATATCGATGTCTCACGTCTACCTTATTACTTGCCTTGATCGTCGGCACCCGATATTTGTCA
ACTGGGAATCCCCCTGGACCTATTATCGGTCCTTTGAAACTAACAGCAATTACCCCAGAAAGTGTCTGCTAA

>p1_ind2837

ATGCAAATAGACCCCGTTGCATATCATTGTTCCGAGTTGAAGTTCGTGCTTGTACCCGAAGAGTCGGGAAAGTTAT
CCCCGACGGAAATAGGATTACATTTTCAGATTGGGCGCCGCGTACATGACAGAAGACCCCGCTCTGAGTCAGTGA
TGACGCACCAACGCAGATATCGATGTCTCACGTCTACCTTATTACTTGCCTTGATCGTCGGCACCCGATATTTGTCA
ACTGGGAATCCTCCTGGACCTATTATCGGTCCTTTGAAACTAACAGCAATTACCCCAGAAAGTGTCTGCTAA

>p1_ind1653

ATGCAAATAGACCCCGTTGCATATCATTGTTCCGAGTTGAAGTTCGTGCTTGTACCCGAAGAGTCGGGAAAGTTAT
CCCCGACGGAAATAGGATTCCATTTTCAGACTGGGCGCCGCGTACATGACAGAAGACCCCGCTCTGAGTCAGTGA
TGACGCTCCAACGCAGATATCGATGTCTCACGTCTGCCTTATTACTTGCCTTGATCGTCGGCACCCGATATTTGTCA
ACTGGGAATCCTCCTGGACCTATTATCGGTCCTTTGAAACTAACAGCAATTACCCCAGAAAGTGTCTGCTAA

>p1_ind3674

ATGCAAATAGACCCCGTTGCATATCATTGTTCCGAGTTGAAGTTCGTGCTTGTACCCGAAGAGTCGGGAAAGTTAT
CCCCGACGGAAATAGGATTCCATTTTCAGATTGGGCGCCGCGTACATGACAGAAGACCCCGCTCTGAGTCAGTGA
TGACGCACCAACGCAGATATCGATGTCTCACGTCTACCTTATTACTTGCCTTGATCGTCGGCACCCGATATTTGTCA
ACTGGGAATCCTCCTGGACCTATTATCGGTCCTTTGAAACTAACAGCAATTACCCCAGAAAGTGTCTGCTAA

>p1_ind1846

ATGCAAATAGACCCCGTTGCATATCATTGTTCCGAGTTGAAGTTCGTGCTTGTACCCGAAGAGTCGGGAAAGTTAT
CCCCGACGGAAATAGGATTCCATTTTCAGACTGGGCGCCGCGTACATGACAGAAGACCCCGCTCTGAGTCAGTGA
TGACGCACCAACGCAGATATCGATGTCTCACGTCTACCTTATTACTTGCCTTGATCGTCGGCACCCGATATTTGTCA
ACTGGGAATCCTCCTGGACCTATTATCGGTCCTTTGAAACTAACAGCAATTACCCCAGAAAGTGTCTGCTAA

>p1_ind786

ATGCAAATAGACCCCGTTGCATATCATTGTTCCGAGTTGAAGTTCGTGCTTGTACCCGAAGAGTCGGGAAAGTTAT
CCCCGACGGAAATAGGATTCCATTTTCAGATTGGGTGCCGCGTACATGACAGAAGACCCCGCTCTGAGTCAGTGA
TGACGCACCAACGCAGATATCGATGTCTCACGTCTACCTTATTACTTGCCTTGATCGTCGGCACCCGATATTTGTCA
ACTGGGAATCCTCCTGGACCTATTCTCGGTCCTTTGAAACTAACAGCAATTACCCCAGAAAGTGTCTGCTAA

>p1_ind2524

ATGCAAATAGACCCCGTTGCATATCATTGTTCCGAGTAGAAGTTCGTGCTTGTACCCGAAGAGTCGGGAAAGTTAT
CCCCGACGGAAATAGGATTCCATTTTCAGACTGGGCGCCGCGTACATGACAGAAGACCCCGCTCTGAGTCAGTGA
TGACGCACCAACGCAGATATCGATGTCTCACGTCTACCTTATTACTTGCCTTGATCGTCGGCACCCGATATTTGTCA
ACTGGGAATCCTCCTGGACCTATTATCGGTCCTTTGAAACTAACAGCAATTACCCCAGAAAGTGTCTGCTAA

>p1_ind3759

ATGCAAATAGACCCCGTTGCATATCATTGTTCCGAGTTGAAGTTCGTGCTTGTACCCGAAGAGTCGGGAAAGTTAT
CCCCGACGGAAATAGGATTCCATTTTGAGATTGGGTGCCGCGTACATGACAGAAGACCCCGCTCTGAGTCAGTGA
TGGCGCACCAACGCAAATATCGATGTCTCACGTCTACCTTATTACTTGCCTTGATCGTCGGCACCCGATATTTGTCA
ACTGGGAATCCTCCTGGACCTATTATCGGTCCTTTGAAACTAACAGCAATTACCCCAGAAAGTGTCTGCTAA

>p1_ind4575

ATGCAAATAGACCCCGTTGCATATCATTGTTCCGAGTTGAAGTTCGTGCTTGTACCCGAAGAGTCGGGAAAGTTAT
CCCCGACGGAAATAGGATTCCATTTTCAGATTGGGTGCCGCGTACATGACAGAAGACCCCGCTCTGAGTCAGTGA
TGACGCACCAACGCAGATATCGATGTCTCACGTCTACCTTATTACTTGCCTTGATCGTCGGCACCCGATATTTGTCA
ACTGGGAATCCTCCTGGACCTATTATCGGTCCTTTGAAACTAACAGTAATTACCCCAGAAAGTGTCTGCTAA

>p1_ind4683

ATGCAAATAGACCCCGTTGCATATCATTGTTCCGAGTTGAAGTTCGTGCTTGTACCCGAAGAGTCGGGAAAGTTAT
CCCCGACGGAAATAGGATTCCATTTTCAGACTGGGCGCCGCGTACATGACAGAAGACCCCGCTCTGAGTCAGTGA
TGACGCACCAACGCAGATATCGATGTCTCACGTCTACCTTATTACTTGCCTTGATCGTCGGCACCCGATATTTGTCA
ACTGGGAATCCTCCTGGACCTATTCTCGGTCCTTTGAAACTAACAGCAATTACCCCAGAAAGTGTCTGCTAA

>p1_ind4386

ATGCAAATAGACCCTGTTGCATATCATTGTTCCGAGTTGAAGTTCGTGCTTGTACCCGAAGAGTCGGGAAAGTTAT
CCCCGACGGAAATAGGATTCCATTTTCAGATTGGGTGCCGCGTACATGACAGAAGACCCCGCTCTGAGTCAGTGA
TGGCGCACCAACGCAAATATCGATGTCTCACGTCTACCTTATTACTTGCCTTGATCGTCGGCACCCGATATTTGTCA
ACTGGGAATCCTCCTGGACCTATTATCGGTCCTTTGAAACTAACAGCAATTACCCCAGAAAGTGTCTGCTAA

>p1_ind2690

ATGCAAATAGACCCCGTTGCATATCATTGTTCCGAGTTGAAGTTCGTGCTTGTACCCGAAGAGTCGGGAAAGTTAT
CCCCGACGGAAATAGGATTCCATTTTCAGACTGGGCGCCGCGTACATGACAGAAGACCCCGCTCTGAGTCAGTGA

TGACGCACCAACGCAGATATCGATGTCTCACGTCTACCTTATTACTTGCCTTGATCGTCGGCACCCGATATTTGTCA
ACTGGGAATCCTCCTGGACCTATTATCGGTCCTTTGAAACTAACAGCAATTACCCCAGAAAGTGTCTGCTAA

>p1_ind4712

AAGCAAATAGACCCCGTTGCATATCATTGTTCCGAGTTGAAGTTCGTGCTTGTACCCGAAGAGTCGGGAAAGTTAT
CCCCGACGGAAATAGGATTCCATTTTCAGACTGGGCGCCGCGTACATGACAGCAGACCCCGCTCTGAGTCAGTGA
TGACGCACCAACGCAGATATCGATGTCTCACGTCTACCTTATTACTTGCCTTGATCGTCGGCACCCGATATTTGTCA
ACTGGGAATCCTCCTGGACCTATTATCGGTCCTTTGAAACTAACAGCAATTACCCCAGAAAGTGTCTGCTAA

>p1_ind4186

ATGCAAATAGACCCCGTTGCATATCATTGTTCCGAGTTGAAGTTCGTGCTTGTACCCGAAGAGTCGGGAAAGTTAT
CCCCGACGGAAATAGGATTCCATTTTCAGATTGGGTGCCGCGTACATGACAGAAGACCCCGCTCTGAGTCAGTGA
TGACGCACCAACGCAGATATCGATGTCTCACGTCTACCTTATTACTTGCCTTGATCGTCGGCACCCGATATTTGTCA
ACTGGGAATCCTCCTGGACCTATTATCGGTCCTTTGAAACTAACAGCAATTACCCCAGAAAGTGTCTGCTAA

>p1_ind4811

ATGCAAATAGACCCCGTTGCATATCATTGTTCCGAGTTGAAGTTCGTGCTTGTACCCGAAGAGTCGGGAAAGTTAT
CCCCGACGGAAATACGATTCCATTTTCAGATTGGGTGCCGCGTACATGACAGAAGACCCCGCTCTGAGTCAGTGAT
GACGCACCAACGCAGATATCGATGTCTCACGTCTACCTTATTACTTGCCTTGATCGTCGGCACCCGATATTTGTCAA
CTGGGAATCCTCCTGGACCTATTCTCGGTCCTTTGAAACTAACAGCAATTACCCCAGAAAGTGTCTGCTAA

>p1_ind3263

AAGCAAATAGACCCCGTTGCATATCATTGTTCCGAGTTGAAGTTCGTGCTTGTACCCGAAGAGTCGGGAAAGTTAT
CCCCGACGGAAATAGGATTCCATTTTCAGACTGGGCGCCGCGTACATGACAGCAGACCCCGCTCTGAGTCAGTGA
TGACGCACCAACGCAGATATCGATGTCTCACGTCTACCTTATTACTTGCCTTGATCGTCGGCACCCGATATTTGTCA
ACTGGGAATCCTCCTGGACCTATTATCGGTCCTTTGAAACTAACAGCAATTACCCCAGAAAGTGTCTGCTAA

>p1_ind3728

ATGCAAATAGACCCCGTTGCATATCATTGTTCCGAGTTGAAGTTCGTGCTTGTACCCGAAGAGTCGGGAAAGTTAT
CCCCGACGGAAATAGGATTCCATTTTCAGATTGGGTGCCGCGTACATGACAGAAGACCCCGCTCTGAGTCAGTGA
TGACGCACCAACGCAGATATCGATGTCTCACGACTACCTTATTACTTGCCTTGATCGTCGGCACCCGATATTTGTCA
ACTGGGAATCCTCCTGGACCTATTATCGGTCCTTTGAAACTAACAGCAATTACCCCAGAAAGTGTCTGCTAA

>p1_ind229

ATGCAAATAGACCCCGTTGCATATCATTGTTCCGAGTTGAAGTTCGTGCTTGTACCCGAAGAGTCGGGAAAGTTAT
CCCCGACGGAAATAGGATTCCATTTTCAGATTGGGTGCCGCGTACATGACAGAAGACCCCGCTCTGAGTCAGTGA
TGACGCACCAACGCAGATATCGATGTCTCACGTCTACCTTATTACTTGCCTTGATCGTCGGCACCCGATATTTGTCA
ACTGGGAATCCTCCTGGACCTATTATCGGTCCTTTGAAACTAACAGCAATTACCCCAGAAAGTGTCTGCTAA

>p1_ind4926

ATGCAAATAGACCCCGTTGCATATCATTGTTCCGAGTTGAAGTTCGTGCTTGTACCCGAAGAGTCGGGAAAGTTAT
CCCCGACGGAAATAGGATTCCATTTTCAGACTGGGCGCCGCGTACATGACAGAAGACCCCGCTCTGAGTCAGTGA
TGCCGCACCAACGCAGATATCGATGTCTCACGTCTACCTTATTACTTGCCTTGATCGTCGGCACCCGATATTTGTCA
ACTGGGAATCCTCCTGGACCTATTATCGGTCCTTTGAAACTAACAGCAATTACCCCAGAAAGTGTCTGCTAA

>p1_ind4537

ATGCAAATAGACCCCGTTGCATATCATTGTTCCGAGTTGAAGTTCGTGCTTGTACCCGAAGAGTCGGGAAAGTTAT
CCCCGACGGAAATAGGATTCCATTTTCAGACTGGGCGCCGCGTACATGACAGAAGACCCCGCTCTGAGTCAGTGA
TGACGCACCAACGCAGATATCGATGTCTCACGTCTACCTTATTACTTGCCTTGATCGTCGGCACCCGATATTTGTCA
ACTGGGAATCCTCCTGGACCTATTATCGGTCCTTTGAAACTAACAGCAATTACCCCAGAAAGTGTCTGCTAA

>p1_ind2586

ATGCAAATAGACCCCGTTGCATATCATTGTTCCGAGTTGAAGTTCGTGCTTGTACCCGAAGAGTCGGGAAAGTTAT
CCCCGACGGAAATAGGATTCCATTTTCAGACTGGGCGCCGCGTACATGACAGAAGACCCCGCTCTGAGTCAGTGA
TGACGCACCAACGCAGATATCGATGTCTCACGTCTACCTTATTACTTGCCTTGATCGTCGGCACCCGATATTTGTCA
ACTGGGAATCCTCCTGGACCTATTATCGGTCCTTTGAAACTAACAGCAATTACCCCAGAAAGTGTCTGCTAA

>p1_ind3624

ATGCAAATAGACCCCGTTGCATATCATTGTTCCGAGTTGAAGTTCGTGCTTGTACCCGAAGAGTCGGGAAAGTTAT
CCCCGACGGAAATAGGATTCCATTTTCAGACTGGGCGCCGCGTACATGACAGAAGACCCCGCTCTGAGTCAGTGA
TGACGCACCAACGCAGATATCGATGTCTCACGTCTACCTTATTACTTGCCTTGATCGTCGGCACCCGATATTTGTCA
ACTGGGAATCCTCCTGGACCTATTATCGGTCCTTTGAAACTAACAGCAATTACCCCAGAAAGTGTCTGCTAA

>p1_ind5066

ATGCAAATAGACCCCGTTGCATATCATTGTTCCGAGTTGAAGTTCGTGCTTGTACCCGAAGAGTCGGGAAAGTTAT
CCCCGACGGAAATAGGATTCCATTTTCAGATTGGGTGCCGCGTACATGACAGAAGACCCCGCTCTGAGTCAGTGA
TGACGCACCAACGCAGATATCGATGTCTCACGTCTACCTTATTACTTGCCTTGATCGTCGGCACCCGATATTTGTCA
ACTGGGAATCCTCCTGGACCTATTATCGGTCCTTTGAAACTAACAGCAATTACCCCAGAAAGTGTCTGCTAA

>p1_ind2548

ATGCAAATAGACCCCGTTGCATATCATTGTTCCGAGTTGAAGTTCGTGCTTGTACCCGAAGAGTCGGGAAAGTTAT
CCCCGACGGAAATAGGATTCCATTTTCAGATTGGGCGCCGCGTACATGACAGAAGACCCCGCTCTGAGTCAGTGA
TGACGCACCAACGCAGATATCGATGTCTCACGTCTACCTTATTACTTGCCTTGATCGTCGGCACCCGATATTTGTCA
ACTGGGAATCCTCCTGGACCTATTATCGGTCCTTTGAAACTAACAGCAATTACCCCAGAAAGTGTCTGCTAA

>p1_ind850

ATGCAAATAGACCCCGTTGCATATCATTGTTCCGAGTTGAAGTTCGTGCTTGTACCCGAAGAGTCGGGAAAGTTAT
CCCCGACGGAAATAGGATTCCATTTTCAGACTGGGCGCCGCGTACATGACAGAAGACCCCGCTCTGAGTCAGTGA

TGACGCACCAACGCAGATATCGATGTCTCACGTCTACCTTATTACTTGCCTTGATCGTCGGCACCCGATATTTGTCA
ACTGGGAATCCTCCTGGACCTATTCTCGGTCCTTTGAAACTAACAGCAATTACCCCAGAAAGTGTCTGCTAA

>p1_ind1075

ATGCAAATAGACCCCGTTGCATATCATTGTTCCGAGTTGAAGTTCGTGCTTGTACCCGAAGAGTCGGGAAAGTTAT
CCCCGACGGAAATAGGATTCCATTTTCAGACTGGGCGCCGCGTACATGACAGAAGACCCCGCTCTGAGTCAGTGA
TGGCGCACCAACGCAAATATCGATGTCTCACGTCTACCTTATTACTTGCCTTGATCGTCGGCACCCGATATTTGTCA
ACTGGGAATCCTCCTGGACCTATTATCGGTCCTTTGAAACTAACAGCAATTACACCAGAAAGTGTCTGCTAA

>p1_ind4251

ATGCAAATAGACCCCGTTGCATATCATTGTTCCGAGTTGAAGTTCGTGCTTGTACCCGAAGAGTCGGGAAAGTTAT
CCCCGACGGAAATAGGATTCCATTTTCAGATTGGGTGCCGCGTACATGACAGAAGACCCCGCTCTGAGTCAGTGA
TGACGCACCAACGCAGATATCGATGTCTCACGTCTACCTTATTACTTGCCTTGATCGTCGGCACCCGATATTTGTCA
ACTGGGAATCCTCCTGGACCTATTATCGGTCCTTTGAAACTAACAGCAATTACCCCAGAAAGTGTCTGCTAA

>p1_ind2008

ATGCAAATAGACCCCGTTGCATATCATTGTTCCGAGTTGAAGTTCGTGCTTGTACCCGAAGAGTCGGGAAAGTTAT
CCCCGACGGAAATAGGATTCCATTTTCAGATTGGGCGCCGCGTACATGACAGAAGACCCCGCTCTGAGTCAGTGA
TGACGCACCAACGCAGATATCGATGTCTCACGTCTACCTTATTACTTGCCTTGATCGTCGGCACCCGATATTTGTCA
ACTGGGAATCCTCCTGGACCTATTATCGGTCCTTTGAAACTAACAGCAATTACCCCAGAAAGTGTCTGCTAA

>p1_ind498

ATGCAAATAGACCCCGTTGCATATCATTGTTCCGAGTTGAAGTTCGTGCTTGTACCCGAAGAGTCGGGAAAGTTAT
CCCCGACGGAAATAGGATTCCATTTTCAGACTGGGCGCCGCGTACATGACAGAAGACCCCGCTCTGAGTCAGTGA
TGACGCACCAACGCAGATATCGATGTCTCACGTCTACCTTATTACTTGCCTTGATCGTCGGCACCCGATATTTGTCA
ACTGGGAATCCTCCTGGACCTATTATCGGTCCTTTGAAACTAACAGCAATTACCCCAGAAAGTGTCTGCTAA

>p1_ind4086

ATGCAAATAGACCCCGTTGCATATCATTGTTCCGAGTTGAAGTTCGTGCTTGTACCCGAAGAGTCGGGAAAGTTAT
CCCCGACGGAAATAGGATTCCATTTTCAGATTGGGTGCCGCGTACATGACAGAAGACCCCGCTCTGAGTCAGTGA
TGACGCACCAACGCAGATATCGATGTCTCACGTCTACCTTATTACTTGCCTTGATCGTCGGCACCCGATATTTGTCA
ACTGGGAATCCTCCTGGACCTATTATCGGTCCTTTGAAACTAACAGCAATTACCCCAGAAAGTGTCTGCTAA

>p1_ind426

ATGCAAATAGACCCCGTTGCATATCATTGTTCCGAGTTGAAGTTCGTGCTTGTACCCGAAGAGTCGGGAAAGTTAT
CCCCGACGGAAATAGGATTCCATTTTCAGATTGGGTGCCGCGTACATGACAGAAGACCCCGCTCTGAGTCAGTGA
TGACGCACCAACGCAGATATCGATGTCTCACGTCTACCTTATTACTTGCCTTGATCGTCGGCACCCGATATTTGTCA
ACTGGGAATCCTCCTGGACCTATTATCGGTCCTTTGAAACTAACAGCAATTACCCCAGAAAGTGTCTGCTAA

>p1_ind1559

ATGCAAATAGACCCCGTTGCATATCATTGTTCCGAGTTGAAGTTCGTGCTTGTACCCGAAGAGTCGGGAAAGTTAT
CCCCGACGGAAATAGGATTCCATTTTCAGATTGGGTGCCGCGTACATGACAGAAGACCCCGCTCTGAGTCAGTGA
TGACGCACCAACGCAGATATCGATGTCTCACGTCTACCTTATTACTTGCCTTGATCGTCGGCACCCGATATTTGTCC
ACTGGGAATCCTCCTGGACCTATTATCGGTCCTTTGAAACTAACAGCAATTACCCCAGAAAGTGTCTGCTAA

>p1_ind746

ATGCAAATAGACCCCGTTGCATATCATTGTTCCGAGTTGAAGTTCGTGCTTGTACCCGAAGAGTCGGGAAAGTTAT
CCCCGACGGAAATAGGATTCCATTTTCAGATTGGGTGCCGCGTACATGACAGAAGACCCCGCTCTGAGTCAGTGA
TGACGCACCAACGCAGATATCGATGTCTCACGTCTACCTTATTACTTGCCTTGATCGTCGGCACCCGATATTTGTCA
ACTGGGAATCCTCCTGGACCTATTATCGGTCCTTTGAAACTAACAGCAATTACCCCAGAAAGTGTCTGCTAA

>p1_ind3815

ATGCAAATAGACCCCGTTGCATATCATTGTTCCGAGTTGAAGTTCGTGCTTGTACCCGAAGAGTCGGGAAAGTTAT
CCCCGACGGAAATAGGATTCCATTTTCAGACTGGGCGCCGCGTACATGACAGAAGACCCCGCTCTGAGTCAGTGA
TGACGCACCAACGCAGATATCGATGTCTCACGTCTACCTTATTACTTGCCTTGATCGTCGGCACCCGATATTTGTCA
ACTGGGAATCCTCCTGGACCTATTATCGGTCCTTTGAAACTAACAGCAATTACCCCAGAAAGTGTCTGCTAA

>p1_ind354

ATGCAAATAGACCCCGTTGCATATCATTGTTCCGAGTTGAAGTTCGTGCTTGTACCCGAAGAGTCGGGAAAGTTAT
CCCCGACGGAAATAGGATTCCATTTTCAGATTGGGTGCCGCGTACATGACAGAAGACCCCGCTCTGAGTCAGTGA
TGACGCCCCAACGCAGATATCGATGTCTCACGTCTACCTTATTACTTGCCTTGATCGTCGGCACCCGATATTTGTCA
ACTGGGAATCCTCCTGGACCTATTATCGGTCCTTTGAAACTAACAGGAATTACCCCAGAAAGTGTCTGCTAA

>p1_ind2547

ATGCAAATAGACCCCGTTGCATATCATTGTTCCGAGTTGAAGTTCGTGCTTGTACCCGAAGAGTCGGGAAAGTTAT
CCCCGACGGAAATAGGATTCCATTTTCAGACTGGGCGCCGCGTACATGACAGAAGACCCCGCTCTGAGTCAGTGA
TGACGCACCAACGCAGACATCGATGTCTCACGTCTACCTTATTACTTGCCTTGATCGTCGGCACCCGATATTTGTCA
ACTGGGAATCCTCCTGGACCTATTATCGGTCCTTTGAAACTAACAGCAATTACCCCAGAAAGTGTCTGCTAA

>p1_ind2569

ATGCAAATAGACCCCGTTGCATATCATTGTTCCGAGTTGAAGTTCGTGCTTGTACCCGAAGAGTCGGGAAAGTTAT
CCCCGACGGAAATAGGATTCCATTTTCAGACTGGGCGCCGCGTACATGACAGAAGACCCCGCTCTGAGTCAGTGA
TGACGCACCAACGCAGATATCGATGTCTCACGTCTACCTTATTACTTGCCTTGATCGTCGGCACCCGATATTTGTCA
ACTGGGAATCCTCCTGGACCTATTATCGGTCCTTTGAAACTAACAGCAATTACCCCAGAAAGTGTCTGCTAA

>p1_ind601

ATGCAAATAGACCCCGTTGCATATCATTGTTCCGAGTTGAAGTTCGTGCTTGTACCCGAAGAGTCGGGAAAGTTAT
CCCCGACGGAAATAGGATTCCATTTTCAGACTGGGCGCCGCGTACATGACAGAAGACCCCGCTCTGAGTCAGTGA

TGACGCACCAACGCAGATATCGATGTCTCACGTCTACCTTATTACTTGCCTTGATCGTCGGCACCCGATATTTGTCA
ACTGGGAATCCTCCTGGAGCTATTATCGGTCCTTTGAAACTAACAGCAATTACCCCAGAAAGTGTCTGCTAA

>p1_ind1200

AAGCAAATAGACCCCGTTGCATATCATTGTTCCGAGTTGAAGTTCGTGCTTGTACCCGAAGAGTCGGGAAAGTTAT
CCCCGACGGAAATAGGATTCCATTTTCAGACTGGGCGCCGCGTACATGACAGAAGACCCCGCTCTGAGTCAGTGA
TGACGCACCAACGCAGATATCGATGTCTCACGTCTACCTTATTACTTGCCTTGATCGTCGGCACCCGATATTTGTCA
ACTGGGAATCCTCCTGGACCTATTATCGGTCCTTTGAAACTAACAGCAATTACCCCAGAAAGTGTCTGCTAA

>p1_ind3770

ATGCAAATAGACCCCGTTGCATATCATTGTTCCGAGTAGAAGTTCGTGCTTGTACCCGAAGAGTCGGGAAAGTTAT
CCCCGACGGAAATAGGATTCCATTTTCAGACTGGGCGCCGCGTACATGACAGAAGACCCCGCTCTGAGTCAGTGA
TGACGCACCAACGCAGATATCGATGTCTCACGTCTACCTTATTACTTGCCTTGATCGTCGGCACCCGATATTTGTCA
ACTGGGAATCCTCCTGGACCTATTATCGGTCCTTTGAAACTAACAGCAATTACCCCAGAAAGTGTCTGCTAA

>p2_ind242

ATGCAAATAGACCCCGTTGCATATCATTGTTCCGAGTTGAAGTTCGTGCTTGTACCCGAAGAGTCGGGAAAGTTAT
CCCCGACGGAAATAGGATTCCATTTTCAGATTGGGTGCCGCGTACATGACAGAAGACCCCGCTCTGAGTCAGTGA
TGACGCACCAACGCAGATATCGATGTCTCACGTCTACCTTATTACTTGCCTTGATCGTCGGCACCCGATATTTGTCA
ACTGGGAATCCTCCTGGACCTATTATCGGTCCTTTGAAACTAACAGCAATTACCCCAGAAAGTGTCTGCTAA

>p2_ind2560

ATGCAAATAGACCCCGTTGCATATCATTGATCCGAGTTGAAGTTCGTGCTTGTACCCGAAGAGTCGGGAAAGTTAT
CCCCGACGGAAATAGGATTCCATTTTCAGATTGGGTGCCGCGTACATGACAGAAGACCCCGCTCTGAGTCAGTGA
TGACGCACCAACGCAGATATCGATGTCTCACGTCTACCTTATTACTTGCCTTGATCGTCGGCACCCGATATTTGTCA
ACTGGGAATCCTCCTGGACCTATTATCGGTCCTTTGAAACTAACAGCAATTACCCCAGAAAGTGTCTGCTAA

>p2_ind4457

ATGCAAATAGACCCCGTTGCATATCATTGTTCCGAGTTGAAGTTCGTGCTTGTACCCGAAGAGTCGGGAAAGTTAT
CCCCGACGGAAATAGGATTCCATTTTCAGATTGGGTGCCGCGTACATGACAGAAGACCCCGCTCTGTGTCAGTGAT
GACGCACCTACGCAGATATCGATGTCTCACGTCTACCTTATTACTTGCCTTGATCGTCGGCACCCGATATTTGTCAA
CTGGGAATCCTCCTGGACCTATTATCGGTCCTTTGAAACTAACAGCAATTACCCCAGAAAGTGTCTGCTAA

>p2_ind821

ATGCAAATAGACCCCGTTGCATATCATTGTTCCGAGTTGAAGTTCGTGCTTGTACCCGAAGAGTCGGGAAAGTTAT
CCCCGACGGAAATAGGATTCCATTTTCAGATTGGGTGCCGCGTACATGACAGAAGACCCCGCTCTGAGTCAGTGA
TGACGCACCAACGCAGATATCGATGTCTCACGTCTACCTTATTACTTGCCTTGATCGTCGGCACCCGATATTTGTCA
ACTGGGAATCCTCCTGGACCTATTATCGGTCCTTTGAAACTAACAGCAATTACCCCAGAAAGTGTCTGCTAA

>p2_ind1682

ATGCAAATAGACCCCGTTGCATATCATTGTTCCGAGTTGAAGTTCGTGCTTGTACCCGAAGAGTCGGGAAAGTTAT
CCCCGACGGAAATAGGATTCCATTTTCAGATTGGGTGCCGCGTACATGACAGAAGACCCCGCTCTGAGTCAGTGA
TGACGCACCAACGCAGATATCGATGTCTCACGTCTACCTTATTACTTGCCTTGATCGTCGGCACCCGATATTTGTCA
ACTGGGAATCCTCCTGGACCTATTATCGGTCCTTTGAAACTAACAGCAATTACCCCAGAAAGTGTCTGCTAA

>p2_ind2601

ATGCAAATAGACCCCGTTGCATATCATTGTTCCGAGTTGAAGTTCGTGCTTGTACCCGAAGAGTCGGGAAAGTTAT
CCCCGACGGAAATAGGATTACATTTTCAGATTGGGCGCCGCGTACATGACAGAAGACCCCGCTCTGAGTCAGTGA
TGACGCACCAACGCAGATATCGATGTCTCACGTCTACCTTATTACTTGCCTTGATCGTCGGCACCCGATATTTGTCA
ACTGGGAATCCTCCTGGACCTATTATCGGTCCTTTGAAACTAACAGCAATTACCCCAGAAAGTGTCTGCTAA

>p2_ind2922

ATGCAAATAGACCCCGTTGCATATCATTGTTCCGAGTTGAAGTTCGTGCTTGTACCCGAAGAGTCGGGAAAGTTAT
CCCCGACGGAAATAGGATTCCATTTTCAGACTGGGCGCCGCGTACATGACAGAAGACCCCGCTCTGAGTCAGTGA
TGACGCACCAACGCAGATATCGATGTCTCACGTCTACCTTATTACTTGCCTTGATCGTCGGCACCCGATATTTGTCA
ACTGGGAATCCTCCTGGACCTATTATCGGTCCTTTGAAACTAACAGCAATTACTCCAGAAAGTGTCTGCTAA

>p2_ind1893

ATGCAAATAGACCCCGTTGCATATCATTGTTCCGAGTTGAAGTTCGTGCTTGTACCCGAAGAGTCGGGAAAGTTAT
CCCCGACGGAAATAGGATTCCATTTTCAGATTGGGTGCCGCGTACATGACAGAAGACCCCGCTCTGAGTCAGTGA
TGGCGCACCAACGCAAATATCGATGTCTCACGTCTACCTTATTACTTGCCTTGATCGTCGGCACCCGATATTTGTCA
ACTGGGAATCCTCCTGGACCTATTATCGGTCCTTTGAAACTAACAGCAATTACCCCAGAAAGTGTCTGCTAA

>p2_ind1178

ATGCAAATAGACCCCGTTGCATATCATTGTTCCGAGTTGAAGTTCGTGCTTGTACCCGAAGAGTCGGGAAAGTTAT
CCCCGACGGAAATAGGATTCCATTTTCAGACTGGGCGCCGCGTACATGACAGAAGACCCCGCTCTGAGTCAGTGA
TGACGCACCAACGCAGATATCGATGTCTCACGTCTACCTTATTACTTGCCTTGATCGTCGGCACCCGATATTTGTCA
ACTGGGAATCCTCCTGGACCTATTATCGGTCCTTTGAAACTAACAGCAATTACACCAGAAAGTGTCTGCTAA

>p2_ind4109

ATGCAAATAGACCCCGTTGCATATCATTGTTCCGAGTTGAAGTTCGTGCTTGTACCCGAAGAGTCGGGAAAGTTAT
CCCCGACGGAAATAGGATTCCATTTTCAGATTGGGTGCCGCGTACATGACAGAAGACCCCGCTCTGAGTCAGTGA
TGACGCACCAACGCAGATATCGATGTCTCACGTCTACCTTATTACTTGCCTTGATCGTCGGCACCCGATATTTGTCA
ACTGGGAATCCTCCTGGACCTATTATCGGTCCTTTGAAACTAACAGCAATTACCCCAGAAAGTGTCTGCTAA

>p2_ind1138

ATGCAAATAGACCCCGTTGCATATCATTGTTCCGAGTTGAAGTTCGTGCTTGTACCCGAAGAGTCGGGAAAGTTAT
CCCCGACGGAAATAGGATTCCATTTTCAGATTGGGTGCCGCGTACATGACAGAAGACCCCGCTCTGAGTCAGTGA

TGACGCACCAACGCAGACATCGATGTCTCACGTCTACCTTATTACTTGCCTTGATCGTCGGCACCCGATATTTGTCA
ACTGGGAATCCTCCTGGACCTATTATCGGTCCTTTGAAACTAACAGCAATTACCCCAGAAAGTGTCTGCTAA

>p2_ind3776

ATGCAAATAGACCCCGTTGCATATCATTGTTCCGAGTTGAAGTTCGTGCTTGTACCCGAAGAGTCGGGAAAGTTAT
CCCCGACGGAAATAGGATTCCATTTTCAGATTGGGTGCCGCGTACATGACAGAAGACCCCGCTCTGAGTCAGTGA
TGACGCACCAACGCAGATATCGATGTCTCACGTCTACCTTATTACTTGCCTTGATCGTCGGCACCCGATATTTGTCA
ACTGGGAATCCTCCTGGACCTATTATCGGTCCTTTGAAACTAACAGCAATTACCCCAGAAAGTGTCTGCTAA

>p2_ind1546

ATGCAAATAGACCCCGTTGCATATCATTGTTCCGAGTTGAAGTTCGTGCTTGTACCCGAAGAGTCGGGAAAGTTAT
CCCCGACGGAAATAGGATTCCATTTTCAGATTGGGTGCCGCGTACATGACAGAAGACCCCGCTCTGAGTCAGTGA
TGACGCACCAACGCAGATATCGATGTCTCACGTCTACCTTATTACTTGCCTTGATCGTCGGCACCCGATATTTGTCA
ACTGGGAATCCTCCTGGACCTATTATCGGTCCTTTGAAACTAACAGCAATTACCCCAGAAAGTGTCTGCTAA

>p2_ind721

ATGCAAATAGACCCCGTTGCATATCATTGTTCCGAGTTGAAGTTCGTGCTTGTACCCGAAGAGTCGGGAAAGTTAT
CCCCGACGGAAATAGGATTCCATTTTCAGATTGGGTGCCGCGTACATGACAGAAGACCCCGCTCTGAGTCAGTGA
TGACGCACCAACGCAGATATCGATGTCTCACGTCTACCTTATTACTTGCCTTGATCGTCGGCACCCGATATTTGTCA
ACTGGGAATCCTCCTGGACCTATTATCGGTCCTTTGAAACTAACAGCAATTACCCCAGAAAGTGTCTGCTAA

>p2_ind4499

ATGCAAATAGACCCCGTTGCATATCATTGTTCCGAGTTGAAGTTCGTGCTTGTACCCGAAGAGTCGGGAAAGTTAT
CCCCGACGGAAATAGGATTCCATTTTCAGATTGGGTGCCGCGTACATGACAGAAGACCCCGCTCTGAGTCAGTGA
TGACGCACCAACGCAGATATCGATGTCTCACGTCTACCTTATTACTTGCCTTGATCGTCGGCACCCGATATTTGTCA
ACTGGGAATCCTCCTGGACCTATTATCGGTCCTTTGAAACTAACAGCAATTACCCCAGAAAGTGTCTGCTAA

>p2_ind343

ATGCAAATAGACCCCGTTGCATATCATTGTTCCGAGTTGAAGTTCGTGCTTGTACCCGAAGAGTCGGGAAAGTTAT
CCCCGACGGAAATAGGATTCCATTTTCAGATTGGGTGCCGCGTACATGACAGAAGACCCCGCTCTGAGTCAGTGA
TGACGCTCCAACGCAGATATCGATGTCTCACGTCTACCTTATTACTTGCCTTGATCGTCGGCACCCGATATTTGTCA
ACTGGGAATCCTCCTGGACCTATTATCGGTCCTTTGAAACTAACAGCAATTACCCCAGAAAGTGTCTGCTAA

>p2_ind2597

ATGCAAATAGACCCCGTTGCATATCATTGTTCCGAGTTGAAGTTCGTGCTTGTACCCGAAGAGTCGGGAAAGTTAT
CCCCGACGGAAATAGGATTCCATTTTCAGATTGGGTGCCGCGTACATGACAGAAGACCCCGCTCTGAGTCAGTGA
TGACGCTCCAACGCAGATATCGATGTCTCACGTCTACCTTATTACTTGCCTTGATCGTCGGCACCCGATATTTGTCA
ACTGGGAATCCTCCTGGACCTATTATCGGTCCTTTGAAACTAACAGCAATTACCCCAGAAAGTGTCTGCTAA

>p2_ind123

ATGCAAATAGACCCCGTTGCATATCATTGTTCCGAGTTGAAGTTCGTGCTTGTACCCGAAGAGTCGGGAAAGTTAT
CCCCGACGGAAATAGGATTCCATTTTCAGATTGGGTGCCGCGTACATGACAGAAGACCCCGCTCTGAGTCAGTGA
TGGCGCACCAACGCAGATATCGATGTCTCACGTCTACCTTATTACTTGCCTTGATCGTCGGCACCCGATATTTGTCA
ACTGGGAATCCTCCTGGACCTATTATCGGTCCTTTGAAACTAACAGCAATTACCCCAGAAAGTGTCTGCTAA

>p2_ind79

ATGCAAATAGACCCCGTTGCATATCATTGTTCCGAGTTGAAGTTCGTGCTTGTACCCGAAGAGTCGGGAAAGTTAT
CCCCGACGGAAATAGGATTCCATTTTCAGATTGGGTGCCGCGTACATGACAGAAGACCCCGTTCTGAGTCAGTGAT
GACGCACCAACGCAGATATCGATGTCTCACGTCTACCTTATTACTTGCCTTGATCGTCGGCACCCGATATTTGTCAA
CTGGGAATCCTCCTGGACCTATTATCGGTCCTTTGAAACTAACAGCAATTACCCCAGAAAGTGTCTGCTAA

>p2_ind183

ATGCAAATAGACCCTGTTGCATATCATTGTTCCGAGTTGAAGTTCGTGCTTGTACCCGAAGAGTCGGGAAAGTTAT
CCCCGACGGAAATAGGATTCCATTTTCAGATTGGGTGCCGCGTACATGACAGAAGACCCCGCTCTGAGTCAGTGA
TGGCGCACCAACGCAAATACAGATGTCTCACGTCTACCTTAATACTTGCCTTGATCGTCGGCACCCGATATTTGTCA
ACTGGGAATCCTCCTGGACCTATTATCGGTCCTTTGAAACTAACAGCAATTACCCCAGAAAGTGTCTGCTAA

>p2_ind834

ATGCAAATAGACCCCGTTGCATATCATTGTTCCGAGTTGAAGTTCGTGCTTGTACCCGAAGAGTCGGGAAAGTTAT
CCCCGACGGAAATAGGATTCCATTTTCAGATTGGGTGCCGCGTACATGACAGAAGACCCCGCTCTGAGTCAGTGA
TGACGCACCAACGCAGATATCGATGTCTCACGTCTACCTTATTACTTGCCTTGATCGTCGGCACCCGATATTTGTCA
ACTGGGAATCCTCCTGGACGTATTATCGGTCCTTTGAAACTAACAGCAATTACCCCAGAAAGTGTCTGCTAA

>p2_ind4244

ATGCAAATAGACCCCGTTGCATATCATTGTTCCGAGTTGAAGTTCGTGCTTGTACCCGAAGAGTCGGGAAAGTTAT
CCCCGACGGAAATAGGATTCCATTTTCAGATTGGGTGCCGCGTACATGACAGAAGACCCCGCTCTGAGTCAGTGA
TGACGCACCAACGCAGATATCGATGTCTCACGTCTACCTTATTACTTGCCTTGATCGTCGGCACCCGATATTTGTCA
ACTGGGAATCCTCCTGGACCTATTATCGGTCCTTTGAAACTAACAGGAATTACCCCAGAAAGTGTCTGCTAA

>p2_ind4582

ATGCAAATAGACCCCGTTGCATATCATTGTTCCGAGTTGAAGTTCGTGCTTGTACCCGAAGAGTCGGGAAAGTTAT
CCCCGACGGAAATAGGATTCCATTTTCAGATTGGGCGCCGCGTACATGACAGAAGACCCCGCTCTGAGTCAGTGA
TGACGCACCAACGCAGATATCGATGTCTCACGTCTACCTTATTACTTGCCTTGATCGTCGGCACCCGATATTTGTCA
ACTGGGAATCCTCCTGGACCTATTATCGGTCCTTTGAAACTAACAGCAATTACCCCAGAAAGTGTCTGCTAA

>p2_ind2689

ATGCAAATAGACCCCGTTGCATATCATTGATCCGAGTTGAAGTTCGTGCTTGTACCCGAAGAGTCGGGAAAGTTAT
CCCCGACGGAAATAGGATTCCATTTTCAGATTGGGTGCCGCGTACATGACAGAAGACCCCGTTCTGAGTCAGTGAT

GACGCACCAACGCAGATATCGATGTCTCACGTCTACCTTATTACTTGCCTTGATCGTCGGCACCCGATATTTGTCAA
CTGGGAATCCTCCTGGACCTATTATCGGTCCTTTGAAACTAACAGCAATTACCCCAGAAAGTGTCTGCTAA

>p2_ind239

ATGCAAATAGACCCCGTTGCATATCATTGTTCCGAGTTGAAGTTCGTGCTTGTACCCGAAGAGTCGGGAAAGTTAT
CCCCGACGGAAATAGGATTCCATTTTCAGACTGGGCGCCGCGTACATGACAGAAGACCCCGCTCTGAGTCAGTGA
TGACGCACCAACGCAGATATCGATGTCTCACGTCTACCTTATTACTTGCCTTGATCGTCGGCACCCGATATTTGTCA
ACTGGGAATCCTCCTGGACCTATTCTCGGTCCTTTGAAACTAACAGCAATTACCCCAGAAAGTGTCTGCTAA

>p2_ind822

ATGCAAATAGACCCCGTTGCATATCATTGTTCCGAGTTGAAGTTCGTGCTTGTACCCGAAGAGTCGGGAAAGTTAT
CCCCGACGGAAATAGGATTCCATTTTCAGATTGGGTGCCGCGTACATGACAGAAGACCCCGCTCTGAGTCAGTGA
TGACGCACCAACGCAGATATCGATGTCTCACGTCTACCTTATTACTTGCCTTGATCGTCGGCACCCGATATTTGTCA
ACTGGGAATCCTCCTGGACCTATTATCGGTCCTTTGAAACTAACAGCAATTACCCCAGAAAGTGTCTGCTAA

>p2_ind259

ATGCAAATAGACCCTGTTGCATATCATTGTTCCGAGTTGAAGTTCGTGCTTGTACCCGAAGAGTCGGGAAAGTTAT
CCCCGACGGAAATAGGATTCCATTTTCAGATTGGGTGCCGCGTACATGACAGAAGACCCCGCTCTGAGTCAGTGA
TGGCGCACCAACGCAAATATCGATGTCTCACGTCTACCTTATTACTTGCCTTGATCGTCGGCACCCGATATTTGTCA
ACTGGGAATCCTCCTGGACCTATTATCGGTCCTTTGAAACTAACAGCAATTACCCCAGAAAGTGTCTGCTAA

>p2_ind3355

ATGCAAATAGACCCCGTTGCATATCATTGTTCCGAGTTGAAGTTCGTGCTTGTACCCGAAGAGTCGGGAAAGTTAT
CCCCGACGGAAATAGGATTCCATTTTCAGATTGGGTGCCGCGTACATGACAGAAGACCCCGCTCTGAGTCAGTGA
TGACGCACCAACGCAGATATCGATGTCTCACGTCTACCTTATTACTTGCCTTGATCGTCGGCACCCGATATTTGTCA
ACTGGGAATCCTCCTGGACCTATTATCGGTCCTTTGAAACTAACACCAATTACCCCAGAAAGTGTCTGCTAA

>p2_ind4621

ATGCAAATAGACCCCGTTGCATATCATTGTTCAGAGTTGAAGTTCGTGCTTGTACCCGAAGAGTCGGGAAAGTTAT
CCCCGACGGAAATAGGATTCCATTTTCAGATTGGGCGCCGCGTACATGACAGAAGACCCCGCTCTGAGTCAGTGA
TGACGCACCAACGCAGATATCGATGTCTCACGTCTACCTTATTACTTGCCTTGATCGTCGGCACCCGATATTTGTCA
ACTGGGAATCCTCCTGGACCTATTATCGGTCCTTTGAAACTAACAGCAATTACCCCAGAAAGTGTCTGCTGA

>p2_ind3898

ATGCAAATAGACCCCGTTGCATATCATTGTTCCGAGTTGAAGTTCGTGCTTGTACCCGAAGAGTCGGGAAAGTTAT
CCCCGACGGAAATAGGATTCCATTTTCAGATTGGGTGCCGCGTACATGACAGAAGACCCCGCTCTGAGTCAGTGA
TGTCGCACCAACGCAGATATCGATGTCTCACGTCTACCTTATTACTTGCCTTGATCGTCGGCACCCGATATTTGTCA
ACTGGGAATCCTCCTGGACCTATTATCGGTCCTTTGAAACTAACAGCAATTACCCCAGAAAGTGTCTGCTAA

>p2_ind3124

ATGCAAATAGACCCCGTTGCATATCATTGTTCCGAGTTGAAGTTCGTGCTTGTACCCGAAGAGTCGGGAAAGTTAT
CCCCGACGGAAATAGGATTCCATTTTCAGACTGGGCGCCGCGTACATGACAGAAGACCCCGCTCTGAGTCAGTGA
TGTCGCACCAACGCAGATATCGATGTCTCACGTCTACCTTATTACTTGCCTTGATCGTCGGCACCCGATATTTGTCA
ACTGGGAATCCTCCTGGACCTATTATCGGTCCTTTGAAACTAACAGCAATTACCCCAGAAAGTGTCTGCTAA

>p2_ind2897

ATGCAAATAGACCCCGTTGCATATCATTGTTCCGAGTTGAAGTTCGTGCTTGTACCCGAAGAGTCGGGAAAGTTAT
CCCCGACGGAAATAGGATTCCATTTTCAGACTGGGCGCCGCGTACATGACAGAAGACCCCGCTCTGAGTCAGTGA
TGACGCACCAACGCAGATATCGATGTCTCACGTCTACCTTATTACTTGCCTTGATCGTCGGCACCCGATATTTGTCA
ACTGGGAATCCTCCTGGACCTATTATCGGTCCTTTGAAACTAACAGCAATTACCCCAGAAAGTGTCTGCTAA

>p2_ind428

ATGCAAATAGACCCCGTTGCATATCATTGTTCCGAGTTGAAGTTCGTGCTTGTACCCGAAGAGTCGGGAAAGTTAT
CCCCGACGGAAATAGGATTCCATTTTCAGATTGGGTGCCGCGTACATGACAGAAGACCCCGCTCTGAGTCAGTGA
TGACGCACCAACGCAGATATCGATGTCTCACGTCTACCTTATTACTTGCCTTGATCGTCGGCACCCGATATTTGTCA
ACTGGGAATCCTCCTGGACCTATTATCGGTCCTTTGAAACTAACAGCAATTACCCCAGAAAGTGTCTGCTAA

>p2_ind502

ATGCAAATAGACCCCGTTGCATATCATTGTTCCGAGTTGAAGTTCGTGCTTGTACCCGAAGAGTCGGGAAAGTTAT
CCCCGACGGAAATAGGATTCCATTGTCAGATTGGGTGCCGCGTACATGACAGAAGACCCCGCTCTGAGTCAGTGA
TGACGCACCAACGCAGATATCGATGTCTCACGTCTACCTTATTACTTGCCTTGATCGTCGGCACCCGATATTTGTCA
ACTGGGAATCCTCCTGGACCTATTATCGGTCCTTTGAAACTAACAGCAATTACCCCAGAAAGTGTCTGCTAA

>p2_ind2620

ATGCAAATAGACCCCGTTGCATATCATTGTTCCGAGTTGAAGTTCGTGCTTGTACCCGAAGAGTCGGGAAAGTTAT
CCCCGACGGAAATAGGATTCCATTTTCAGATTGGGTGCCGCGTACATGACAGAAGACCCCGCTCTGAGTCAGTGA
TGACGCACCAACGCAGATATCGATGTCTCACGTCTACCTTATTACTTGCCTTGATCGTCGGCACCCGATATTTGTCA
ACTGGGAATCCTCCTGGACCTATTATCGGTCCTTTGAAACTAACAGCAATTACCCCAGAAAGTGTCTGCTAA

>p2_ind3209

ATGCAAATAGACCCCGTTGCATATCATTGTTCCGAGTTGAAGTTCGTGCTTGTACCCGAAGAGTCGGGAAAGTTAT
CCCCGACGGAAATAGGATTACATTTTCAGATTGGGCGCCGCGTACATGACAGAAGACCCCGCTCTGAGTCAGTGA
TGGCGCACCAACGCAGATATCGATGTCTCACGTCTACCTTATTACTTGCCTTGATCGTCGGCACCCGATATTTGTCA
ACTGGGAATCCTCCTGGACCTATTATCGGTCCTTTGAAACTAACAGCAATTACCCCAGAAAGTGTCTGCTAA

>p2_ind3563

ATGCAAATAGACCCCGTTGCATATCATTGTTCCGAGTTGAAGTTCGTGCTTGTACCCGAAGAGTCGGGAAAGTTAT
CCCCGACGGAAATAGGATTCCATTTTCAGACTGGGCGCCGCGTACATGACAGAAGACCCCGCTCTGAGTCAGTGA

TGACGCACCAACGCAGATATCGATGTCTCACGTCTACCTTATTACTTGCCTTGATCGTCGGCACCCGATATTTGTCA
ACTGGGAATCCTCCTGGACCTATTATCGGTCCTTTGAAACTAACAGCAATTACACCAGAAAGTGTCTGCTAA

>p2_ind2492

ATGCAAATAGACCCCGTTGCATATCATTGTTCCGAGTTGAAGTTCGTGCTTGTACCCGAAGTGTCGGGAAAGTTAT
CCCCGACGGAAATAGGATTCCATTTTCAGATTGGGTGCCGCGTACATGACAGAAGACCCCGCTCTGAGTCAGTGA
TGACGCACCAACGCAGATATCGATGTCTCACGTCTACCTTATTACTTGCCTTGATCGTCGGCACCCGATATTTGTCA
ACTGGGAATCCTCCTGGACCTATTATCGGTCCTTTGAAACTAACGGCAATTACCCCAGAAAGTGTCTGCTAA

>p2_ind1667

ATGCAAATAGACCCCGTTGCATATCATTGTTCCGAGTTGAAGTTCGTGCTTGTACCCGAAGAGTCGGGAAAGTTAT
CCCCGACGGAAATAGGATTCCATTTTCAGATTGGGTGCCGCGTACATGACAGAAGACCCCGCTCTGAGTCAGTGA
TGACGCACCAACGCAGATATCGATGTCTCACGTCTACCTTATTACTTGCCTTGATCGTCGGCACCCGATATTTGTCA
ACTGGGAATCCTCCTGGACCTATTATCGGTCCTTTGAAACTAACAGCAATTACCCCAGAAAGTGTCTGCTAA

>p2_ind4307

ATGCAAATAGACCCCGTTGCATATCATTGTTCCGAGTTGAAGTTCGTGCTTGTACCCGAAGAGTCGGGAAAGTTAT
CCCCGACGGAAATAGGATTCCATTTTCAGATTGGGTGCCGCGTACATGACAGAAGACCCCGCTCTGAGTCAGTGA
TGACGCACCAACGCAGATATCGATGTCTCACGTCTACCTTATTACTTGCCTTGATCGTCGGCACCCGATATTTGTCA
ACTGGGAATCCTCCTGGACCTATTATCGGTCCTTTGAAACTAACAGCAATTACTCCAGAAAGTGTCTGCTAA

>p2_ind2107

ATGCAAATAGACCCCGTTGCATATCATTGATCCGAGTTGAAGTTCGTGCTTGTACCCGAAGAGTCGGGAAAGTTAT
CCCCGACGGAAATAGGATTCCATTTTCAGATTGGGTGCCGCGTACATGACAGAAGACCCCGCTCTGAGTCAGTGA
TGACGCACCAACGCAGATATCGATGTCTCACGTCTACCTTATTACTTGCCTTGATCGTCGGCACCCGATATTTGTCA
ACTGGGAATCCTCCTGGACCTATTATCGGTCCTTTGAAACTAACAGCAATTACCCCAGAAAGTGTCTGCTAA

>p2_ind1043

ATGCAAATAGACCCCGTTGCATATCATTGTTCCGAGTTGAAGTTCGTGCTTGTACCCGAAGAGTCGGGAAAGTTAT
CCCCGACGGAAATAGGATTCCATTTTCAGATTGGGTGCCGCGTACATGACAGAAGACCCCGCTCTGAGTCAGTGA
TGACGCACCAACGCAGATATCGATGTCTCACGTCTACCTTATTACTTGCCTTGATCGTCGGCACCCGATATTTGTCA
ACTGGGAATCCTCCTGGACCTATTATCGGTCCTTTGAAACTAACAGCAATTACCCCAGAAAGTGTCTGCTAA

>p2_ind4895

ATGCAAATAGACCCCGTTGCATATCATTGTTCCGAGTTGAAGTTCGTGCTTGTACCCGAAGAGTCGGGAAAGTTAT
CCCCGACGGAAATAGGATTCCATTTTCAGATTGGGTGCCGCGTACATGACAGAAGACCCCGCTCTGAGTCAGTGA
TGACGCTCCAACGCAGATATCGATGTCTCACGTCTACCTTATTACTTGCCTTGATCGTCGGCACCCGATATTTGTCA
ACTGGGAATCCTCCTGGACCTATTATCGGTCCTTTGAAACTAACAGCAATTACCCCAGAAAGTGTCTGCTAA

>p2_ind2705

ATGCAAATAGACCCCGTTGCATATCATTGTTCCGAGTTGAAGTTCGTGCTTGTACCCGAAGAGTCGGGAAAGTTAT
CCCCGACGGAAATAGGATTCCATTTTCAGATTGGGTGCCGCGTACATGACAGAAGACCCCGCTCTGAGTCAGTGA
TGACGCTCCAACGCAGATATCGATGTCTCACGTCTACCTTATTACTTGCCTTGATCGTCGGCACCCGATATTTGTCA
ACTGGGAATCCTCCTGGACCTATTATCGGTCCTTTGAAACTAACAGCAATTACCCCAGAAAGTGTCTGCTAA

>p2_ind2132

ATGCAAATAGACCCCGTTGCATATCATTGTTCCGAGTTGAAGTTCGTGCTTGTACCCGAAGAGTCGGGAAAGTTAT
CCCCGACGGAAATAGGATTCCATTTTCAGATTGGGTGCCGCGTACATGACAGAAGACCCCGCTCTGAGTCAGTGA
TGACGCCCCAACGCAGATATCGATGTCTCACGTCTACCTTATTACTTGCCTTGATCGTCGGCACCCGATATTTGTCA
ACTGGGAATCCTCCTGGACCTATTATCGGTCCTTTGAAACTAACAGGAATTACCCCAGAAAGTGTCTGCTAA

>p2_ind1723

ATGCAAATAGACCCCGTTGCATATCATTGTTCCGAGTTGAAGTTCGTGCTTGTACCCGAAGAGTCGGGAAAGTTAT
CCCCGACGGAAATAGGATTCCATTTTCAGACTGGGCGCCGCGTACATGACAGAAGACCCCGCTCTGAGTCAGTGA
TGACGCACCAACGCAGATATCGATGTCTCACGTCTACCTTATTACTTGCCTTGATCGTCGGCACCCGATATTTGTCA
ACTGGGAATCCTCCTGGACCTATTATCGGTCCTTTGAAACTAACAGCAATTACCCCAGAAAGTGTCTGCTAA

>p2_ind3108

ATGCAAATAGACCCCGTTGCATATCATTGTTCCGAGTTGAAGTTCGTGCTTGTACCCGAAGAGTCGGGAAAGTTAT
CCCCGACGGAAATAGGATTCCATTTTCAGATTGGGCGCCGCGTACATGACAGAAGACCCCGCTCTGAGTCAGTGA
TGACGCACCAACGTAGATATCGATGTCTCACGTCTACCTTATTACTTGCCTTGATCGTCGGCACCCGATATTTGTCA
ACTGGGAATCCTCCTGGACCTATTATCGGTCCTTTGAAACTAACAGCAATTACCCCAGAAAGTGTCTGCTAA

>p2_ind335

ATGCAAATAGACCCCGTTGCATATCATTGTTCCGAGTTGAAGTTCGTGCTTGTACCCGAAGAGTCGGGAAAGTTAT
CCCCGACGGAAATAGGATTCCATTTTCAGATTGGGTGCCGCGTACATGACAGAAGACCCCGCTCTGAGTCAGTGA
TGACGCACCAACGCAGATATCGATGTCTCACGTCTACCTTATTACTTGCCTTGATCGTCGGCACCCGATATTTGTCA
ACTGGGAATCCTCCTGGACCTATTATCGGTCCTTTGAAACTAACAGCAATTACTCCAGAAAGTGTCTGCTAA

>p2_ind848

ATGCAAATAGACCCCGTTGCATATCATTGTTCCGAGTTGAAGTTCGTGCTTGTACCCGAAGAGTCGGGAAAGTTAT
CCCCGACGGAAATAGGATTCCATTTTCAGATTGGGTGCCGCGTACATGACAGAAGACCCCGCTCTGAGTCAGTGA
TGACGCACCAACGCAGATATCGATGTCTCACGTCTACCTTATTACTTGCCTTGATCGTCGGCACCCGATATTTGTCA
ACTGGGAATCCTCCTGGACCTATTATCGGTCCTTTGAAACTAACAGCAATTACCCCAGAAAGTGTCTGCTAA

>p2_ind2380

ATGCAAATAGACCCTGTTGCATATCATTGTTCCGAGTTGAAGTTCGTGCTTGTACCCGAAGAGTCGGGAAAGTTAT
CCCCGACGGAAATAGGATTCCATTTTCAGATTGGGTGCCGCGTACATGACAGCAGACCCCGCTCTGAGTCAGTGAT

GGCGCACCAACGCAAATATCGATGTCTCACGTCTACCTTATTACTTGCCTTGATCGTCGGCACCCGATATTTGTCAA
CTGGGAATCCTCCTGGACCTATTATCGGTCCTTTGAAACTAACAGCAATTACCCCAGAAAGTGTCTGCTAA

>p2_ind4367

ATGCAAATAGACCCCGTTGCATATCATTGTTCCGAGTTGAAGTTCGTGCTTGTACCCGAAGAGTCGGGAAAGTTAT
CCCCGACGGAAATAGGATTCCATTTTCAGATTGGGTGCCGCGTACATGACAGAAGACCCCGTTCTGAGTCAGTGAT
GACGCACCAACGCAGATATCGATGTCTCACGTCTACCTTATTACTTGCCTTGATCGTCGGCACCCGATATTTGTCAA
CTGGGAATCCTCCTGGACCTATTATCGGTCCTTTGAAACTAACAGGAATTACCCCAGAAAGTGTCTGCTAA

>p2_ind4798

ATGCAAATAGACCCCGTTGCATATCATTGTTCCGAGTTGAAGTTCGTGCTTGTACCCGAAGAGTCGGGAAAGTTAT
CCCCGACGGAAATAGGATTCCATTTTCAGATTGGGTGCCGCGTACATGACAGAAGACCCCGCTCTGAGTCAGTGA
TGACGCACCAACGCAGATATCGATGTCTCACGTCTACCTTATTACTTGCCTTGATCGTCGGCACCCGATATTTGTCA
ACTGGGAATCCTCCTGGACCTATTATCGGTCCTTTGAAACTAACAGCAATTACCCCAGAAAGTGTCTGCTAA

>p2_ind1066

ATGCAAATAGACCCCGTTGCATATCATTGTTCCGAGTTGAAGTTCGTGCTTGTACCCGAAGAGTCGGGAAAGTTAT
CCCCGACGGAAATAGGATTCCATTTTCAGATTGGGTGCCGCGTACATGACAGAAGACCCCGTTCTGAGTCAGTGAT
GACGCACCAACGCAGATATCGATGTCTCACGTCTACCTTATTACTTGCCTTGATCGTCGGCACCCGATATTTGTCAA
CTGGGAATCCTCCTGGACCTATTATCGGTCCTTTGAAACTAACAGGAATTACCCCAGAAAGTGTCTGCTAA

>p2_ind2899

ATGCAAATAGACCCCGTTGCATATCATTGATCCGAGTTGAAGTTCGTGCTTGTACCCGAAGAGTCGGGAAAGTTAT
CCCCGACGGAAATAGGATTCCATTTTCAGATTGGGTGCCGCGTACATGACAGAAGACCCCGTTCTGAGTCAGTGAT
GACGCACCAACGCAGATATCGATGTCTCACGTCTACCTTATTACTTGCCTTGATCGTCGGCACCCGATATTTGTCAA
CTGGGAATCCTCCTGGACCTATTATCGGTCCTTTGAAACTAACAGCAATTACCCCAGAAAGTGTCTGCTAA

>p2_ind3417

ATGCAAATAGACCCCGTTGCATATCATTGTTCCGAGTTGAAGTTCGTGCTTGTACCCGAAGAGTCGGGAAAGTTAT
CCCCGACGGAAATAGGATTCCATTTTCAGACTGGGCGCCGCGTACATGACAGAAGACCCCGCTCTGAGTCAGTGA
TGACGCACCAACGCAGATATCGATGTCTCACGTCTACCTTATTACTTGCCTTGATCGTCGGCACCCGATATTTGTCA
ACTGGGAATCCTCCTGGACCTATTATCGGTCCTTTGAAACTAACAGCAATTACCCCAGAAAGTGTCTGCTAA

>p2_ind4025

ATGCAAATAGACCCCGTTGCATATCATTGTTCCGAGTTGAAGTTCGTGCTTGTACCCGAAGAGTCGGGAAAGTTAT
CCCCGACGGAAATAGGATTCCATTTTCAGATTGGGTGCCGCGTACATGACAGAAGACCCCGCTCTGAGTCAGTGA
TGACGCACCAACGCAGATATCGATGTCTCACGTCTACCTTATTACTTGCCTTGATCGTCGGCACCCGATATTTGTCA
ACTGGGAATCCTCCTGGACCTATTATCGGTCCTTTGAAACTAACAGCAATTACCCCAGAAAGTGTCTGCTAA

>p2_ind4222

ATGCAAATAGACCCCGTTGCATATCATTGTTCCGAGTTGAAGTTCGTGCTTGTACCCGAAGAGTCGGGAAAGTTAT
CCCCGACGGAAATAGGATTCCATTTTCAGATTGGGTGCCGCGTACATGACAGAAGACCCCGCTCTGAGTCAGTGA
TGACGCACCAACGCAGATATCGATGTCTCACGTCTACCTTATTACTTGCCTTGATCGTCGGCACCCGATATTTGTCA
ACTGGGAATCCTCCTGGACCTATTATCGGTCCTTTGAAACTAACAGCAATTACCCCAGAAAGTGTCTGCTAA

>p2_ind2825

ATGCAAATAGACCCCGTTGCATATCATTGTTCCGAGTTGAAGTTCGTGCTTGTACCCGAAGAGTCGGGAAAGTTAT
CCCCGACGGAAATAGGATTCCATTTTCAGATTGGGTGCCGCGTACATGACAGAAGACCCCGCTCTGAGTCAGTGA
TGACGCACCAACGCAGATATCGATGTCTCACGTCTACCTTATTACTTGCCTTGATCGTCGGCACCCGATATTTGTCA
ACTGGGAATCCTCCTGGACCTATTATCGGTCCTTTGAAACTAACAGCAATTACCCCAGAAAGTGTCTGCTAA

>p2_ind1786

ATGCAAATAGACCCCGTTGCATATCATTGTTCCGAGTTGAAGTTCGTGCTTGTACCCGAAGAGTCGGGAAAGTTAT
CCCCGACGGAAATAGGATTCCATTTTCAGATTGGGTGCCGCGTACATGACAGAAGACCCCGCTCTGAGTCAGTGA
TGACGCACCAACGCAGATATCGATGTCTCACGTCTACCTTATTACTTGCCTTGATCGTCGGCACCCGATATTTGTCA
ACTGGGAATCCTCCTGGACCTATTATCGGTCCTTTGAAACTAACAGCAATTACCCCAGAAAGTGTCTGCTAA

>p2_ind1829

ATGCAAATAGACCCTGTTGCATATCATTGTTCCGAGTTGAAGTTCGTGCTTGTACCCGAAGAGTCGGGAAAGTTAT
CCCCGACGGAAATAGGATTCCATTTTCAGATTGGGTGCCGCGTACATGACAGAAGACCCCGCTCTGAGTCAGTGA
TGACGCACCAACGCAGATATCGATGTCTCACGTCTACCTTATTACTTGCCTTGATCGTCGGCACCCGATATTTGTCA
ACTGGGAATCCTCCTGGACCTATTATCGGTCCTTTGAAACTAACAGCAATTACCCCAGAAAGTGTCTGCTAA

>p2_ind2753

ATGCAAATAGACCCCGTTGCATATCATTGTTCCGAGTTGAAGTTCGTGCTTGTACCCGAAGAGTCGGGAAAGTTAT
CCCCGACGGAAATAGGATTCCATTTTCAGATTGGGTGCCGCGTACATGACAGAAGACCCCGCTCTGAGTCAGTGA
TGACGCACCAACGCAGATATCGATGTCTCACGTCTACCTTATTACTTGCCTTGATCGTCGGCACCCGATATTTGTCA
ACTGGGAATCCTCCTGGACCTATTATCGGTCCTTTGAAACTAACAGCAATTACTCCAGAAAGTGTCTGCTAA

>p2_ind3171

ATGCAAATAGACCCCGTTGCATATCATTGTTCCGAGTTGAAGTTCGTGCTTGTACCCGAAGAGTCGGGAAAGTTAT
CCCCGACGGAAATAGGATTCCATTTTCAGATTGGGTGCCGCGTACATGACAGAAGACCCCGCTCTGAGTCAGTGA
TGACGCACCAACGCAGATATCGATGTCTCACGTCAACCTTATTACTTGCCTTGATCGTCGGCACCCGATATTTGTCA
ACTGGGAATCCTCCTGGACCTATTATCGGTCCTTTGAAACTAACAGCAATTACCCCAGAAAGTGTCTGCTAA

>p2_ind4408

ATGCAAATAGACCCCGTTGCATATCATTGTTCCGAGTTGAAGTTCGTGCTTGTACCCGAAGAGTCGGGAAAGTTAT
CCCCGACGGAAATAGGATTCCATTTTCAGATTGGGCGCCGCGTACATGACAGAAGACCCCGCTCTGAGTCAGTGA

TGACGCACCAACGCAGATATCGATGTCTCACGTCTACCTTATTACTTGCCTTGATCGTCGGCACCCGATATTTGTCA
ACTGGGAATCCTCCTGGACCTATTATCGGTCCTTTGAAACTAACAGCAATTACCCCAGAAAGTGTCTGCTAA

>p2_ind1383

ATGCAAATAGACCCCGTTGCATATCATTGTTCCGAGTTGAAGTTCGTGCTTGTACCCGAAGAGTCGGGAAAGTTAT
CCCCGACGGAAATAGGATTCCATTTTCAGATTGGGTGCCGCGTACATGACAGCAGACCCCGCTCTGAGTCAGTGAT
GACGCACCAACGCAGATATCGATGTCTCACGTCTACCTTATTACTTGCCTTGATCGTCGGCACCCGATATTTGTCAA
CTGGGAATCCTCCTGGACCTATTATCGGTCCTTTGAAACTAACAGCAATTACTCCAGAAAGTGTCTGCTAA

>p2_ind1512

ATGCAAATAGACCCCGTTGCATATCATTGTTCCGAGTTGAAGTTCGTGCTTGTACCCGAAGAGTCGGGAAAGTTAT
CCCCGACGGAAATAGGATTCCATTTTCAGATTGGGTGCCGCGTACATGACAGAAGACCCCGCTCTGAGTCAGTGA
TGGCGCACCAACGCAGATATCGATGTCTCACGTCTACCTTATTACTTGCCTTGATCGTCGGCACCCGATATTTGTCA
ACTGGGAATCCTCCTGGACCTATTATCGGTCCTTTGAAACTAACAGCAATTACCCCATAAAGTGTCTGCTAA

>p2_ind4942

ATGCAAATAGACCCCGTTGCATATCATTGTTCCGAGTTGAAGTTCGTGCTTGTACCCGAAGAGTCGGGAAAGTTAT
CCCCGACGGAAATAGGATTCCATTTTCAGACTGGGCGCCGCGTACATGACAGAAGACCCCGCTCTGAGTCAGTGA
TGACGCACCAACGCAGATATCGATGTCTCACGTCTACCTTATTACTTGCCTTGATCGTCGGCACCCGATATTTGTCA
ACTGGGAATCCTCCTGGACCTATTATCGGTCCTTTGAAACTAACAGCAATTACCCCAGAAAGTGTCTGCTAA

>p2_ind1243

ATGCAAATAGACCCCGTTGCATATCATTGTTCCGAGTTGAAGTTCGTGCTTGTACCCGAAGAGTCGGGAAAGTTAT
CCCCGACGGAAATAGGATTCCATTTTCAGATTGGGTGCCGCGTACATGACAGAAGACCCCGCTCTGAGTCAGTGA
TGACGCACCAACGCAGATATCGATGTCTCACGTCTACCTTATTACTTGCCTTGATCGTCGGCACCCGATATTTGTCA
ACTGGGAATCCTCCTGGACCTATTATCGGTCCTTTGAAACTAACAGCAATTACCCCAGAAAGTGTCTGCTAA

>p2_ind680

ATGCAAATAGACCCCGTTGCATATCATTGTTCCGAGTTGAAGTTCGTGCTTGTACCCGAAGAGTCGGGAAAGTTAT
CCCCGACGGAAATAGGATTCCATTTTCAGATTGGGTGCCGCGTACATGACAGAAGACCCCGCTCTGAGTCAGTGA
TGACGCACCAACGCAGATATCGATGTCTCACGTCTACCTTATTACTTGCCTTGATCGTCGGCACCCGATATTTGTCA
ACTGGGAATCCTCCTGGACCTATTATCGGTCCTTTGAAACTAACGGCAATTACCCCAGAAAGTGTCTGCTAA

>p2_ind677

ATGCAAATAGACCCCGTTGCATATCATTGTTCCGAGTTGAAGTTCGTGCTTGTACCCGAAGAGTCGGGAAAGTTAT
CCCCGACGGAAATAGGATTCCATTTTCAGATTGGGTGCCGCGTACATGACAGAAGACCCCGTTCTGAGTCAGTGAT
GACGCACCAACGCAGATATCGATGTCTCACGTCTACCTTATTACTTGCCTTGATCGTCGGCACCCGATATTTGTCAA
CTGGGAATCCTCCTGGACCTATTATCGGTCCTTTGAAACTAACAGCAATTACCCCAGAAAGTGTCTGCTAA

>p2_ind1967

ATGCAAATAGACCCCGTTGCATATCATTGTTCCGAGTTGAAGTTCGTGCTTGTACCCGAAGAGTCGGGAAAGTTAT
CCCCGACGGAAATAGGATTCCATTTTCAGATTGGGTGCCGCGTACATGACAGAAGACCCCGCTCTGAGTCAGTGA
TGACGCTCCAACGCAGATATCGATGTCTCACGTCTACCTTATTACTTGCCTTGATCGTCGGCACCCGATATTTGTCA
ACTGGGAATCCTCCTGGACCTATTATCGGTCCTTTGAAACTAACAGCAATTACCCCAGAAAGTGTCTGCTAA

>p2_ind4673

ATGCAAATAGACCCCGTTGCATATCATTGTTCCGAGTTGAAGTTCGTGCTTGTACCCGAAGAGTCGGGAAAGTTAT
CCCCGACGGAAATAGGATTCCATTTTCAGATTGGGTGCCGCGTACATGACAGAAGACCCCGCTCTGAGTCAGTGA
TGGCGCACCAACGCAGATATCGATGTCTCACGTCTACCTTATTACTTGCCTTGATCGTCGGCACCCGATATTTGTCA
ACTGGGAATCCTCCTGGACCTATTATCGGTCCTTTGAAACTAACAGCAATTACCCCATAAAGTGTCTGCTAA

>p2_ind770

ATGCAAATAGACCCCGTTGCATATCATTGTTCCGAGTTGAAGTTCGTGCTTGTACCCGAAGAGTCGGGAAAGTTAT
CCCCGACGGAAATAGGATTCCATTTTCAGATTGGGTGCCGCGTACATGACAGAAGACCCCGCTCTGAGTCAGTGA
TGACGCACCAACGCAGATATCGATGTCTCACGTCTACCTTATTACTTGCCTTGATCGTCGGCACCCGATATTTGTCA
ACTGGGAATCCTCCTGGACCTATTATCGGTCCTTTGAAACTAACAGCAATTACCCCAGAAAGTGTCTGCTAA

>p2_ind3509

ATGCAAATAGACCCCGTTGCATATCATTGTTCCGAGTTGAAGTTCGTGCTTGTACCCGAAGAGTCGGGAAAGTTAT
CCCCGACGGAAATAGGATTCCATTTTCAGATTGGGTGCCGCGTACATGACAGAAGACCCCGCTCTGTGTCAGTGAT
GACGCACCTACGCAGATATCGATGTCTCACGTCTACCTTATTACTTGCCTTGATCGTCGGCACCCGATATTTGTCAA
CTGGGAATCCTCCTGGACCTATTATCGGTCCTTTGAAACTAACAGCAATTACCCCAGAAAGTGTCTGCTAA

>p2_ind2965

ATGCAAATAGACCCCGTTGCATATCATTGTTCCGAGTTGAAGTTCGTGCTTGTACCCGAAGAGTCGGGAAAGTTAT
CCCCGACGGAAATAGGATTCCATTTTCAGATTGGGTGCCGCGTACATGACAGAAGACCCCGCTCTGAGTCAGTGA
TGGCGCACCAACGCAAATATCGATGTCTCACGTCTACCTTATTACTTGCCTTGATCGTCGGCACCCGATATTTGTCA
ACTGGGAATCCTCCTGGACCTATTATCGGTCCTTTGAAACTAACAGCAATTACCCCAGAAAGTGTCTGCTAA

>p2_ind4446

ATGCAAATAGACCCCGTTGCATATCATTGTTCCGAGTTGAAGTTCGTGCTTGTACCCGAAGAGTCGGGAAAGTTAT
CCCCGACGGAAATAGGATTCCATTTTCAGATTGGGCGCCGCGTACATGACAGAAGACCCCGCTCTGAGTCAGTGA
TGACGCACCAACGCAGATATCGATGTCTCACGTCTACCTTATTACTTGCCTTGATCGTCGGCACCCGATATTTGTCA
ACTGGGAATCCTCCTGGACCTATTATCGGTCCTTTGAAACTAACAGCAATTACCCCAGAAAGTGTCTGCTAA

>p2_ind4583

ATGCAAATAGACCCCGTTGCATATCATTGTTCCGAGTTGAAGTTCGTGCTTGTACCCGAAGAGTCGGGAAAGTTAT
CCCCGACGGAAATAGGATTCCATTTTCAGATTGGGTGCCGCGTACATGACAGAAGACCCCGCTCTGAGTCAGTGA

TGACGCACCAACGCAGATATCGATGTCTCACGTCTACCTTATTACTTGCCTTGATCGTCGGCACCCGATATTTGTCA
ACTGGGAATCCTCCTGGACCTATTATCGGTCCTTTGAAACTAACAGCAATTACTCCAGAAAGTGTCTGCTAA

>p2_ind3872

ATGCAAATAGACCCCGTTGCATATCATTGTTCCGAGTTGAAGTTCGTGCTTGTACCCGAAGAGTCGGGAAAGTTAT
CCCCGACGGAAATAGGATTCCATTTTCAGACTGGGCGCCGCGTACATGACAGAAGACCCCGCTCTGAGTCAGTGA
TGACGCACCAACGCAGATATCGATGTCTCACGTCTACCTTATTACTTGCCTTGATCGTCGGCACCCGATATTTGTCA
ACTGGGAATCCTCCTGGACCTATTATCGGTCCTTTGAAACTAACAGCAATTACCCCAGAAAGTGTCTGCTAA

>p2_ind2347

ATGCAAATAGACCCTGTTGCATATCATTGTTCCGAGTTGAAGTTCGTGCTTGTACCCGAAGAGTCGGGAAAGTTAT
CCCCGACGGAAATAGGATTCCATTTTCAGATTGGGTGCCGCGTACATGACAGAAGACCCCGCTCTGAATCAGTGAT
GACGCACCAACGCAGATATCGATGTCTCACGTCTACCTTATTACTTGCCTTGATCGTCGGCACCCGATATTTGTCAA
CTGGGAATCCTCCTGGACCTATTATCGGTCCTTTGAAACTAACAGCAATTACCCCAGAAAGTGTCTGCTAA

>p2_ind2094

ATGCAAATAGACCCCGTTGCATATCATTGTTCCGAGTTGAAGTTCGTGCTTGTACCCGAAGAGTCGGGAAAGTTAT
CCCCGACGGAAATAGGATTCCATTTTCAGACTGGGCGCCGCGTACATGACAGAAGACCCCGCTCTGAGTCAGTGA
TGACGCACCAACGCAGATATCGATGTCTCACGTCTACCTTATTACTTGCCTTGATCGTCGGCACCCGATATTTGTCA
ACTGGGAATCCTCCTGGAGCTATTATCGGTCCTTTGAAACTAACAGCAATTACCCCAGAAAGTGTCTGCTAA

>p2_ind180

ATGCAAATAGACCCCGTTGCATATCATTGTTCCGAGTTGAAGTTCGTGCTTGTACCCGAAGAGTCGGGAAAGTTAT
CCCCGACGGAAATAGGATTCCATTTTCAGATTGGGTGCCGCGTACATGACAGAAGACCCCGCTCTGAGTCAGTGA
TGACGCACCAACGCAGACATCGATGTCTCACGTCTACCTTATTACTTGCCTTGATCGTCGGCACCCGATATTTGTCA
ACTGGGAATCCTCCTGGACCTATTATCGGTCCTTTGAAACTAACAGCAATTACCCCAGAAAGTGTCTGCTAA

>p2_ind1771

ATGCAAATAGACCCCGTTGCATATCATTGTTCCGAGTTGAAGTTCGTGCTTGTACCCGAAGAGTCGGGAAAGTTAT
CCCCGACGGAAATAGGATTCCATTTTCAGATTGGGCGCCGCGTACATGACAGAAGACCCCGCTCTGAGTCAGTGA
TGACGCACCAACGCAGATATCGATGTCTCACGTCTACCTTATTACTTGCCTTGATCGTCGGCACCCGATATTTGTCA
ACTGGGAATCCTCCTGGACCTATTATCGGTCCTTTGAAACTAACAGCAATTACCCCAGAAAGTGTCTGCTAA

>p2_ind3374

ATGCAAATAGACCCCGTTGCATATCATTGATCCGAGTTGAAGTTCGTGCTTGTACCCGAAGAGTCGGGAAAGTTAT
CCCCGACGGAAATAGGATTCCATTTTCAGATTGGGTGCCGCGTACATGACAGAAGACCCCGTTCTGAGTCAGTGAT
GACGCACCAACGCAGATATCGATGTCTCACGTCTACCTTATTACTTGCCTTGATCGTCGGCACCCGATATTTGTCAA
CTGGGAATCCTCCTGGACCTATTATCGGTCCTTTGAAACTAACAGCAATTACCCCAGAAAGTGTCTGCTAA

>p2_ind1428

ATGCAAATAGACCCTGTTGCATATCATTGTTCCGAGTTGAAGTTCGTGCTTGTACCCGAAGAGTCGGGAAAGTTAT
CCCCGACGGAAATAGGATTCCATTTTCAGATTGGGTGCCGCGTACATGACAGAAGACCCCGCTCTGAGTCAGTGA
TGGCGCACCAACGCAAATACAGATGTCTCACGTCTACCTTAATACTTGCCTTGATCGTCGGCACCCGATATTTGTCA
ACTGGGAATCCTCCTGGACCTATTATCGGTCCTTTGAAACTAACAGCAATTACCCCAGAAAGTGTCTGCTAA

>p2_ind1867

ATGCAAATAGACCCCGTTGCATATCATTGTTCCGAGTTGAAGTTCGTGCTTGTACCCGAAGAGTCGGGAAAGTTAT
CCCCGACGGAAATAGGATTCCATTTTCAGATTGGGTGCCGCGTACATGACAGAAGACCCCGCTCTGAGTCAGTGA
TGACGCACCAACGCAGATATCGATGTCTCACGTCTACCTTATTACTTGCCTTGATCGTCGGCACCCGATATTTGTCA
ACTGGGAATCCTCCTGGACCTATTATCGGTCCTTTGAAACTAACAGCAATTACCCCAGAAAGTGTCTGCTAA

>p2_ind2629

ATGCAAATAGACCCCGTTGCATATCATTGTTCCGAGTTGAAGTTCGTGCTTGTACCCGAAGAGTCGGGAAAGTTAT
CCCCGACGGAAATAGGATTCCATTTTCAGATTGGGTGCCGCGTACATGACAGAAGACCCCGCTCTGAATCAGTGAT
GACGCACCAACGCAGATATCGATGTCTCACGTCTACCTTATTACTTGCCTTGATCGTCGGCACCCGATATTTGTCAA
CTGGGAATCCTCCTGGACCTATTATCGGTCCTTTGAAACTAACAGCAATTACCCCAGAAAGTGTCTGCTAA

>p2_ind3597

ATGCAAATAGACCCCGTTGCATATCATTGTTCCGAGTTGAAGTTCGTGCTTGTACCCGAAGAGTCGGGAAAGTTAT
CCCCGACGGAAATAGGATTCCATTTTCAGATTGGGTGCCGCGTACATGACAGAAGACCCCGCTCTGAGTCAGTGA
TGACGCACCAACGCAGATATCGATGTCTCACGTCTACCTTATTACTTGCCTTGATCGTCGGCACCCGATATTTGTCA
ACTGGGAATCCTCCTGGACCTATTATCGGTCCTTTGAAACTAACAGCAATTACCCCATAAAGTGTCTGCTAA

>p2_ind4760

ATGCAAATAGACCCCGTTGCATATCATTGTTCCGAGTTGAAGTTCGTGCTTGTACCCGAAGAGTCGGGAAAGTTAT
CCCCGACGGAAATAGGATTCCATTTTCAGATTGGGTGCCGCGTACATGACAGAAGACCCCGCTCTGAGTCAGTGA
TGGCGCACCAACGCAGATATCGATGTCTCACGTCTACCTTATTACTTGCCTTGATCGTCGGCACCCGATATTTGTCA
ACTGGGAATCCTCCTGGACCTATTATCGGTCCTTTGAAACTAACAGCAATTACCCCATAAAGTGTCTGCTAA

>p2_ind1161

ATGCAAATAGACCCCGTTGCATATCATTGTTCCGAGTTGAAGTTCGTGCTTGTACCCGAAGAGTCGGGAAAGTTAT
CCCCGACGGAAATAGGATTCCATTTTGAGATTGGGTGCCGCGTACATGACAGAAGACCCCGCTCTGAGTCAGTGA
TGGCGCACCAACGCAAATATCGATGTCTCACGTCTACCTTATTACTTGCCTTGATCGTCGGCACCCGATATTTGTCA
ACTGGGAATCCTCCTGGACCTATTATCGGTCCTTTGAAACTAACAGCAATTACCCCAGAAAGTGTCTGCTAA

>p2_ind3817

ATGCAAATAGACCCCGTTGCATATCATTGTTCCGAGTTGAAGTTCGTGCTTGTACCCGAAGAGTCGGGAAAGTTAT
CCCCGACGGAAATAGGATTCCATTTTCAGATTGGGCGCCGCGTACATGACAGAAGACCCCGCTCTGAGTCAGTGA

TGACGCACCAACGCAGATATCGATGTCTCACGTCTACCTTATTACTTGCCTTGATCGTCGGCACCCGATATTTGTCA
ACTGGGAATCCTCCTGGACCTATTATCGGTCCTTTGAAACTAACAGCAATTACCCCAGAAAGTGTCTGCTAA

>p2_ind3263

ATGCAAATAGACCCCGTTGCATATCATTGTTCCGAGTTGAAGTTCGTGCTTGTACCCGAAGAGTCGGGAAAGTTAT
CCCCGACGGAAATAGGATTCCATTTTCAGATTGGGTGCCGCGTACATGACAGAAGACCCCGCTCTGAGTCAGTGA
TGACGCCCCAACGCAGATATCGATGTCTCACGTCTACCTTATTACTTGCCTTGATCGTCGGCACCCGATATTTGTCA
ACTGGGAATCCTCCTGGACCTATTATCGGTCCTTTGAAACTAACAGCAATTACCCCAGAAAGTGTCTGCTAA

>p2_ind1690

ATGCAAATAGACCCCGTTGCATATCATTGTTCCGAGTTGAAGTTCGTGCTTGTACCCGAAGAGTCGGGAAAGTTAT
CCCCGACGGAAATAGGATTCCATTTTCAGATTGGGTGCCGCGTACATGACAGAAGACCCCGCTCTGAGTCAGTGA
TGACGCACCAACGCAGATATCGATGTCTCACGTCTACCTTATTACTTGCCTTGATCGTCGGCACCCGATATTTGTCA
ACTGGGAATCCTCCTGGACCTATTATCGGTCCTTTGAAACTAACAGCAATTACCCCAGAAAGTGTCTGCTAA

>p2_ind1703

ATGCAAATAGACCCCGTTGCATATCATTGTTCCGAGTTGAAGTTCGTGCTTGTACCCGAAGAGTCGGGAAAGTTAT
CCCCGACGGAAATAGGATTCCATTTTCAGATTGGGTGCCGCGTACATGACAGAAGACCCCGCTCTGAGTCAGTGA
TGACGCACCAACGCAGACATCGATGTCTCACGTCTACCTTATTACTTGCCTTGATCGTCGGCACCCGATATTTGTCA
ACTGGGAATCCTCCTGGACCTATTATCGGTCCTTTGAAACTAACAGCAATTACCCCAGAAAGTGTCTGCTAA

>p2_ind552

ATGCAAATAGACCCCGTTGCATATCATTGTTCCGAGTTGAAGTTCGTGCTTGTACCCGAAGAGTCGGGAAAGTTAT
CCCCGACGGAAATAGGATTCCATTTTCAGATTGGGTGCCGCGTACATGACAGAAGACCCCGCTCTGAGTCAGTGA
TGACGCACCAACGCAGATATCGATGTCTCACGTCTACCTTATTACTTGCCTTGATCGTCGGCACCCGATATTTGTCA
ACTGGGAATCCTCCTGGACCTATTATCGGTCCTTTGAAACTAACGGCAATTACCCCAGAAAGTGTCTGCTAA

>p2_ind2771

ATGCAAATAGACCCCGTTGCATATCATTGTTCCGAGTTGAAGTTCGTGCTTGTACCCGAAGAGTCGGGAAAGTTAT
CCCCGACGGAAATAGGATTCCATTTTCAGATTGGGTGCCGCGTACATGACAGAAGACCCCGCTCTGAGTCAGTGA
TGACGCACCAACGCAGATATCGATGTCTCACGTCTACCTTATTACTTGCCTTGATCGTCGGCACCCGATATTTGTCA
ACTGGGAATCCTCCTGGACCTATTATCGGTCCTTTGAAACTAACAGCAATTACCCCAGAAAGTGTCTGCTAA

>p2_ind296

ATGCAAATAGACCCCGTTGCATATCATTGTTCCGAGTTGAAGTTCGTGCTTGTACCCGAAGAGTCGGGAAAGTTAT
CCCCGACGGAAATAGGATTCCATTTTCAGATTGGGTGCCGCGTACATGACAGAAGACCCCGCTCTGAGTCAGTGA
TGACGCACCAACGCAGATATCGATGTCTCACGTCTACCTTATTACTTGCCTTGATCGTCGGCACCCGATATTTGTCA
ACTGGGAATCCTCCTGGACCTATTATCGGTCCTTTGAAACTAACAGGAATTACCCCAGAAAGTGTCTGCTAA

>p2_ind1895

ATGCAAATAGACCCCGTTGCATATCATTGTTCCGAGTTGAAGTTCGTGCTTGTACCCGAAGAGTCGGGAAAGTTAT
CCCCGACGGAAATAGGATTCCATTTTCAGATTGGGTGCCGCGTACATGACAGAAGACCCCGCTCTGAGTCAGTGA
TGACGCACCAACGCAGATATCGATGTCTCACGTCTACCTTATTACTTGCCTTGATCGTCGGCACCCGATATTTGTCA
ACTGGGAATCCTCCTGGACCTATTATCGGTCCTTTGAAACTAACGGCAATTACCCCAGAAAGTGTCTGCTAA

>p2_ind3721

ATGCAAATAGACCCCGTTGCATATCATTGTTCCGAGTTGAAGTTCGTGCTTGTACCCGAAGAGTCGGGAAAGTTAT
CCCCGACGGAAATAGGATTCCATTTTCAGATTGGGTGCCGCGTACATGACAGAAGACCCCGCTCTGAGTCAGTGA
TGACGCACCAACGCAGATATCGATGTCTCACGTCTACCTTATTACTTGCCTTGATCGTCGGCACCCGATATTTGTCA
ACTGGGAATCCTCCTGGACCTATTATCGGTCCTTTGAAACTAACAGCAATTACCCCAGAAAGTGTCTGCTAA

>p2_ind3153

ATGCAAATAGACCCCGTTGCATATCATTGATCCGAGTTGAAGTTCGTGCTTGTACCCGAAGAGTCGGGAAAGTTAT
CCCCGACGGAAATAGGATTCCATTTTCAGATTGGGTGCCGCGTACATGACAGAAGACCCCGTTCTGAGTCAGTGAT
GACGCACCAACGCAGATATCGATGTCTCACGTCTACCTTATTACTTGCCTTGATCGTCGGCACCCGATATTTGTCAA
CTGGGAATCCTCCTGGACCTATTATCGGTCCTTTGAAACTAACAGCAATTACCCCAGAAAGTGTCTGCTAA

>p2_ind2464

ATGCAAATAGACCCCGTTGCATATCATTGTTCCGAGTTGAAGTTCGTGCTTGTACCCGAAGAGTCGGGAAAGTTAT
CCCCGACGGAAATAGGATTCCATTTTCAGATTGGGTGCCGCGTACATGACAGAAGACCCCGCTCTGAGTCAGTGA
TGACGCACCAACGCAGACATCGATGTCTCACGTCTACCTTATTACTTGCCTTGATCGTCGGCACCCGATATTTGTCA
ACTGGGAATCCTCCTGGACCTATTATCGGTCCTTTGAAACTAACAGCAATTACCCCAGAAAGTGTCTGCTAA

>p2_ind163

ATGCAAATAGACCCCGTTGCATATCATTGTTCCGAGTTGAAGTTCGTGCTTGTACCCGAAGAGTCGGGAAAGTTAT
CCCCGACGGAAATAGGATTCCATTTTCAGACTGGGCGCCGCGTACATGACAGAAGACCCCGCTCTGAGTCAGTGA
TGACGCACCAACGCAGATATCGATGTCTCACGTCTACCTTATTACTTGCCTTGATCGTCGGCACCCGATATTTGTCA
ACTGGGAATCCTCCTGGACCTATTATCGGTCCTTTGAAACTAACAGCAATTACCCCAGAAAGTGTCTGCTAA

>p2_ind1980

ATGCAAATAGACCCCGTTGCATATCATTGTTCCGAGTTGAAGTTCGTGCTTGTACCCGAAGAGTCGGGAAAGTTAT
CCCCGACGGAAATAGGATTCCATTTTCAGATTGGGTGCCGCGTACATGACAGAAGACCCCGCTCTGAGTCAGTGA
TGACGCACCAACGCAGATATCGATGTCTCACGTCTACCTTATTACTTGCCTTGATCGTCGGCACCCGATATTTGTCA
ACTGGGAATCCTCCTGGACCTATTATCGGTCCTTTGAAACTAACAGCAATTACCCCAGAAAGTGTCTGCTAA

>p2_ind3649

ATGCAAATAGACCCCGTTGCATATCATTGTTCCGAGTTGAAGTTCGTGCTTGTACCCGAAGAGTCGGGAAAGTTAT
CCCCGACGGAAATAGGATTACATTTTCAGATTGGGCGCCGCGTACATGACAGAAGACCCCGCTCTGAGTCAGTGA

TGACGCACCAACGCAGATATCGATGTCTCACGTCTACCTTATTACTTGCCTTGATCGTCGGCACCCGATATTTGTCA
ACTGGGAATCCTCCTGGACCTATTATCGGTCCTTTGAAACTAACAGCAATTACCCCAGAAAGTGTCTGCTAA

>p2_ind3065

ATGCAAATAGACCCCGTTGCATATCATTGTTCCGAGTTGAAGTTCGTGCTTGTACCCGAAGAGTCGGGAAAGTTAT
CCCCGACGGAAATAGGATTCCATTTTCAGATTGGGTGCCGCGTACATGACAGAAGACCCCGCTCTGAGTCAGTGA
TGACGCACCAACGCAGATATCGATGTCTCACGTCTACCTTATTACTTGCCTTGATCGTCGGCACCCGATATTTGTCA
ACTGGGAATCCTCCTGGACCTATTATCGGTCCTTTGAAACTAACAGCAATTACCCCAGAAAGTGTCTGCTAA

>p2_ind4197

ATGCAAATAGACCCCGTTGCATATCATTGATCCGAGTTGAAGTTCGTGCTTGTACCCGAAGAGTCGGGAAAGTTAT
CCCCGACGGAAATAGGATTCCATTTTCAGATTGGGTGCCGCGTACATGACAGAAGACCCCGCTCTGAGTCAGTGA
TGACGCACCAACGCAGATATCGATGTCTCACGTCTACCTTATTACTTGCCTTGATCGTCGGCACCCGATATTTGTCA
ACTGGGAATCCTCCTGGACCTATTATCGGTCCTTTGAAACTAACAGCAATTACCCCAGAAAGTGTCTGCTAA

>p2_ind3051

ATGCAAATAGACCCCGTTGCATATCATTGTTCCGAGTTGAAGTTCGTGCTTGTACCCGAAGAGTCGGGAAAGTTAT
CCCCGACGGAAATAGGATTCCATTTTCAGATTGGGTGCCGCGTACATGACAGAAGACCCCGCTCTGAGTCAGTGA
TGACGCACCTACGCAGATATCGATGTCTCACGTCTACCTTATTACTTGCCTTGATCGTCGGCACCCGATATTTGTCA
ACTGGGAATCCTCCTGGACCTATTATCGGTCCTTTGAAACTAACAGCAATTACCCCAGAAAGTGTCTGCTAA

>p2_ind2459

ATGCAAATAGACCCCGTTGCATATCATTGTTCCGAGTTGAAGTTCGTGCTTGTACCCGAAGAGTCGGGAAAGTTAT
CCCCGACGGAAATAGGATTCCATTTTCAGATTGGGTGCCGCGTACATGACAGAAGACCCCGCTCTGAGTCAGTGA
TGACGCACCAACGCAGATATCGATGTCTCACGTCTACCTTATTACTTGCCTTGATCGTCGGCACCCGATATTTGTCA
ACTGGGAATCCTCCTGGACCTATTATCGGTCCTTTGAAACTAACAGCAATTACCCCAGAAAGTGTCTGCTAA

>p2_ind4810

ATGCAAATAGACCCCGTTGCATATCATTGATCCGAGTTGAAGTTCGTGCTTGTACCCGAAGAGTCGGGAAAGTTAT
CCCCGACGGAAATAGGATTCCATTTTCAGATTGGGTGCCGCGTACATGACAGAAGACCCCGTTCTGAGTCAGTGAT
GACGCACCAACGCAGATATCGATGTCTCACGTCTACCTTATTACTTGCCTTGATCGTCGGCACCCGATATTTGTCAA
CTGGGAATCCTCCTGGACCTATTATCGGTCCTTTGAAACTAACAGCAATTACCCCAGAAAGTGTCTGCTAA

>p2_ind2262

ATGCAAATAGACCCCGTTGCATATCATTGTTCCGAGTTGAAGTTCGTGCTTGTACCCGAAGAGTCGGGAAAGTTAT
CCCCGACGGAAATAGGATTCCATTTTCAGACTGGGTGCCGCGTACATGACAGAAGACCCCGCTCTGAGTCAGTGA
TGACGCACCAACGTAGATATCGATGTCTCACGTCTACCTTATTACTTGCCTTGATCGTCGGCACCCGATATTTGTCA
ACTGGGAATCCTCCTGGACCTATTATCGGTCCTTTGAAACTAACAGCAATTACCCCAGAAAGTGTCTGCTAA

>p2_ind3996

ATGCAAATAGACCCCGTTGCATATCATTGTTCCGAGTTGAAGTTCGTGCTTGTACCCGAAGAGTCGGGAAAGTTAT
CCCCGACGGAAATAGGATTCCATTTTCAGATTGGGTGCCGCGTACATGACAGAAGACCCCGCTCTGAGTCAGTGA
TGGCGCACCAACGCAGATATCGATGTCTCACGTCTACCTTATTACTTGCCTTGATCGTCGGCACCCGATATTTGTCA
ACTGGGAATCCTCCTGGACCTATTATCGGTCCTTTGAAACTAACAGCAATTACCCCATAAAGTGTCTGCTAA

>p2_ind4949

ATGCAAATAGACCCCGTTGCATATCATTGTTCCGAGTTGAAGTTCGTGCTTGTACCCGAAGAGTCGGGAAAGTTAT
CCCCGACGGAAATAGGATTCCATTTTCAGATTGGGTGCCGCGTACATGACAGAAGACCCCGCTCTGAGTCAGTGA
TGACGCACCTACGCAGATATCGATGTCTCAAGTCTACCTTATTACTTGCCTTGATCGTCGGCACCCGATATTTGTCA
ACTGGGAATCCTCCTGGACCTATTATCGGTCCTTTGAAACTAACAGCAATTACCCCAGAAAGTGTCTGCTAA

>p2_ind2339

ATGCAAATAGACCCCGTTGCATATCATTGTTCCGAGTTGAAGTTCGTGCTTGTACCCGAAGAGTCGGGAAAGTTAT
CCCCGACGGAAATAGGATTCCATTTTCAGATTGGGTGCCGCGTACATGACAGAAGACCCCGCTCTGAGTCAGTGA
TGGCGCACCAACGCAAATATCGATGTCTCACGTCTACCTTATTACTTGCCTTGATCGTCGGCACCCGATATTTGTCA
ACTGGGAATCCTCCTGGACCTATTATCGGTCCTTTGAAACTAACAGCAATTACCCCAGAAAGTGTCTGCTAA

>p2_ind3335

ATGCAAATAGACCCCGTTGCATATCATTGTTCCGAGTTGAAGTTCGTGCTTGTACCCGAAGAGTCGGGAAAGTTAT
CCCCGACGGAAATAGGATTCCATTTTCAGATTGGGTGCCGCGTACATGACAGAAGACCCCGCTCTGAGTCAGTGA
TGACGCACCAACGCAGATATCGATGTCTCACGTCTACCTTATTACTTGGCTTGATCGTCGGCACCCGATATTTGTCA
ACTGGGAATCCTCCTGGACCTATTATCGGTCCTTTGAAACTAACAGCAATTACCCCAGAAAGTGTCTGCTAA

>p2_ind3300

ATGCAAATAGACCCCGTTGCATATCATTGTTCCGAGTAGAAGTTCGTGCTTGTACCCGAAGAGTCGGGAAAGTTAT
CCCCGACGGAAATAGGATTCCATTTTCAGACTGGGCGCCGCGTACATGACAGAAGACCCCGCTCTGAGTCAGTGA
TGACGCACCAACGCAGATATCGATGTCTCACGTCTACCTTATTACTTGCCTTGATCGTCGGCACCCGATATTTGTCA
ACTGGGAATCCTCCTGGACCTATTATCGGTCCTTTGAAACTAACAGCAATTACCCCAGAAAGTGTCTGCTAA

>p2_ind2468

ATGCAAATAGACCCCGTTGCATATCATTGTTCCGAGTTGAAGTTCGTGCTTGTACCCGAAGAGTCGGGAAAGTTAT
CCCCGACGGAAATAGGATTCCATTTTCAGATTGGGTGCCGCGTACATGACAGAAGACCCCGCTCTGAGTCAGTGA
TGACGCACCAACGCAGATATCGATGTCTCACGTCTACCTTATTACTTGCCTTGATCGTCGGCACCCGATATTTGTTA
ACTGGGAATCCTCCTGGACCTATTATCGGTCCTTTGAAACTAACAGCAATTACCCCATAAAGTGTCTGCTAA

>p2_ind4474

ATGCAAATAGACCCCGTTGCATATCATTGTTCCGAGTTGAAGTTCGTGCTTGTACCCGAAGAGTCGGGAAAGTTAT
CCCCGACGGAAATAGGATTACATTTTCAGATTGGGCGCCGCGTACATGACAGAAGACCCCGCTCTGAGTCAGTGA

TGACGCACCTACGCAGATATCGATGTCTCACGTCTACCTTATTACTTGCCTTGATCGTCGGCACCCGATATTTGTCA
ACTGGGAATCCTCCTGGACCTATTATCGGTCCTTTGAAACTAACAGCAATTACCCCAGAAAGTGTCTGCTAA

>p2_ind2801

ATGCAAATAGACCCCGTTGCATATCATTGTTCCGAGTTGAAGTTCGTGCTTGTACCCGAAGAGTCGGGAAAGTTAT
CCCCGACGGAAATAGGATTCCATTTTCAGATTGGGTGCCGCGTACATGACAGAAGACCCCGTTCTGAGTCAGTGAT
GACGCACCAACGCAGATATCGATGTCTCACGTCTACCTTATTACTTGCCTTGATCGTCGGCACCCGATATTTGTCAA
CTGGGAATCCTCCTGGACCTATTATCGGTCCTTTGAAACTAACAGCAATTACCCCAGAAAGTGTCTGCTAA

>p2_ind3346

ATGCAAATAGACCCCGTTGCATATCATTGTTCCGAGTTGAAGTTCGTGCTTGTACCCGAAGAGTCGGGAAAGTTAT
CCCCGACGGAAATAGGATTCCATTTTCAGATTGGGCGCCGCGTACATGACAGAAGACCCCGCTCTGAGTCAGTGA
TGACGCACCAACGCAGATATCGATGTCTCACGTCTACCTTATTACTTGCCTTGATCGTCGGCACCCGATATTTGTCA
ACTGGGAATCCTCCTGGACCTATTATCGGTCCTTTGAAACTAACAGCAATTACCCCAGAAAGTGTCTGCTAA

>p2_ind1938

ATGCAAATAGACCCCGTTGCATATCATTGTTCCGAGTTGAAGTTCGTGCTTGTACCCGAAGAGTCGGGAAAGTTAT
CCCCGACGGAAATAGGATTCCATTTTCAGATTGGGTGCCGCGTACATGACAGAAGACCCCGCTCTGAGTCAGTGA
TGACGCACCAACGCAGATATCGATGTCTCACGTCTACCTTATTACTTGCCTTGATCGTCGGCACCCGATATTTGTCA
ACTGGGAATCCTCCTGGACCTATTATCGGTCCTTTGAAACTAACGGCAATTACCCCAGAAAGTGTCTGCTAA

>p2_ind2603

ATGCAAATAGACCCCGTTGCATATCATTGTTCCGAGTTGAAGTTCGTGCTTGTACCCGAAGAGTCGGGAAAGTTAT
CCCCGACGGAAATAGGATTCCATTTTCAGATTGGGTGCCGCGTACATGACAGAAGACCCCGCTCTGAGTCAGTGA
TGACGCACCAACGCAGATATCGATGTCTCACGTCTACCTTATTACTTGCCTTGATCGTCGGCACCCGATATTTGTCA
ACTGGGAATCCTCCTGGACCTATTATCGGTCCTTTGAAACTAACAGCAATTACCCCAGAAAGTGTCTGCTAA

>p2_ind2686

ATGCAAATAGACCCTGTTGCATATCATTGTTCCGAGTTGAAGTTCGTGCTTGTACCCGAAGAGTCGGGAAAGTTAT
CCCCGACGGAAATAGGATTCCATTTTCAGATTGGGTGCCGCGTACATGACAGAAGACCCCGCTCTGAGTCAGTGA
TGGCGCACCAACGCAAATACAGATGTCTCACGTCTACCTTAATACTTGCCTTGATCGTCGGCACCCGATATTTGTCA
ACTGGGAATCCTCCTGGACCTATTATCGGTCCTTTGAAACTAACAGCAATTACCCCAGAAAGTGTCTGCTAA

>p2_ind548

ATGCAAATAGACCCCGTTGCATATCATTGTTCCGAGTTGAAGTTCGTGCTTGTACCCGAAGAGTCGGGAAAGTTAT
CCCCGACGGAAATAGGATTCCATTTTCAGATTGGGTGCCGCGTACATGACAGAAGACCCCGCTCTGAGTCAGTGA
TGACGCACCAACGCAGATATCGATGTCTCACGTCTACCTTATTACTTGCCTTGATCGTCGGCACCCGATATTTGTCA
ACTGGGAATCCTCCTGGACCTATTATCGGTCCTTTGAAACTAAAAGCAATTACCCCAGAAAGTGTCTGCTAA

>p2_ind1919

ATGCAAATAGACCCCGTTGCATATCATTGTTCCGAGTTGAAGTTCGTGCTTGTACCCGAAGAGTCGGGAAAGTTAT
CCCCGACGGAAATAGGATTCCATTTTCAGATTGGGTGCCGCGTACATGACAGAAGACCCCGCTCTGAGTCAGTGA
TGACGCACCAACGCAGATATCGATGTCTCACGTCTACCTTATTACTTGCCTTGATCGTCGGCACCCGATATTTGTCA
ACTGGGAATCCTCCTGGACCTATTATCGGTCCTTTGAAACTAACAGCAATTACCCCAGAAAGTGTCTGCTAA

>p2_ind3947

ATGCAAATAGACCCCGTTGCATATCATTGTTCCGAGTTGAAGTTCGTGCTTGTACCCGAAGAGTCGGGAAAGTTAT
CCCCGACGGAAATAGGATTCCATTTTCAGATTGGGTGCCGCGTACATGACAGAAGACCCCGCTCTGAGTCAGTGA
TGACGCACCAACGCAGATATCGATGTCTCACGTCTACCTTATTACTTGCCTTGATCGTCGGCACCCGATATTTGTCA
ACTGGGAATCCTCCTGGACCTATTATCGGTCCTTTGAAACTAACGGCAATTACCCCAGAAAGTGTCTGCTAA

>p2_ind3316

ATGCAAATAGACCCCGTTGCATATCATTGTTCCGAGTTGAAGTTCGTGCTTGTACCCGAAGAGTCGGGAAAGTTAT
CCCCGACGGAAATAGGATTCCATTTTCAGATTGGGTGCCGCGTACATGACAGAAGACCCCGCTCTGAGTCAGTGA
TGACGCACCAACGCAGACATCGATGTCTCACGTCTACCTTATTACTTGCCTTGATCGTCGGCACCCGATATTTGTCA
ACTGGGAATCCTCCTGGACCTATTATCGGTCCTTTGAAACTAACAGCAATTACCCCAGAAAGTGTCTGCTAA

>p2_ind4552

ATGCAAATAGACCCCGTTGCATATCATTGTTCCGAGTTGAAGTTCGTGCTTGTACCCGAAGAGTCGGGAAAGTTAT
CCCCGACGGAAATAGGATTCCATTTTCAGATTGGGTGCCGCGTACATGACAGAAGACCCCGCTCTGAGTCAGTGA
TGACGCACCAACGCAGATATCGATGTCTCACGTCTACCTTATTACTTGCCTTGATCGTCGGCACCCGATATTTGTCA
ACTGGGAATCCTCCTGGACCTATTATCGGTCCTTTGAAACTAACAGCAATTACCCCAGAAAGTGTCTGCTAA

>p2_ind3982

ATGCAAATAGACCCCGTTGCATATCATTGTTCCGAGTTGAAGTTCGTGCTTGTACCCGAAGAGTCGGGAAAGTTAT
CCCCGACGGAAATAGGATTCCATTTTCAGATTGGGTGCCGCGTACATGACAGAAGACCCCGCTCTGTGTCAGTGAT
GACGCACCTACGCAGATATCGATGTCTCAAGTCTACCTTATTACTTGCCTTGATCGTCGGCACCCGATATTTGTCAA
CTGGGAATCCTCCTGGACCTATTATCGGTCCTTTGAAACTAACAGCAATTACCCCAGAAAGTGTCTGCTAA

>p2_ind474

ATGCAAATAGACCCCGTTGCATATCATTGTTCCGAGTTGAAGTTCGTGCTTGTACCCGAAGAGTCGGGAAAGTTAT
CCCCGACGGAAATAGGATTCCATTTTCAGATTGGGTGCCGCGTACATGACAGAAGACCCCGCTCTGAGTCAGTGA
TGACGCACCAACGCAGATATCGATGTCTCACGTCTACCTTATTACTTGCCTTGATCGTCGGCACCCGATATTTGTCA
ACTGGGAATCCTCCTGGACCTATTATCGGTCCTTTGAAACTAACAGGAATTACCCCAGAAAGTGTCTGCTAA

>p2_ind187

ATGCAAATAGACCCCGTTGCATATCATTGTTCCGAGTTGAAGTTCGTGCTTGTACCCGAAGAGTCGGGAAAGTTAT
CCCCGACGGAAATAGGATTCCATTTTCAGATTGGGTGCCGCGTACATGACAGAAGACCCCGCTCTGAGTCAGTGA

TGACGCACCAACGCAGATATCGATGTCTCACGTCTACCTTATTACTTGCCTTGATCGTCGGCACCCGATATTTGTCA
ACTGGGAATCCTCCTGGACCTATTATCGGTCCTTTGAAACTAACAGCAATTACCCCAGAAAGTGTCTGCTAA

>p2_ind3102

ATGCAAATAGACCCCGTTGCATATCATTGTTCCGAGTTGAAGTTCGTGCTTGTACCCGAAGAGTCGGGAAAGTTAT
CCCCGACGGAAATAGGATTCCATTTTCAGATTGGGTGCCGCGTACATGACAGAAGACCCCGCTCTGAGTCAGTGA
TGACGCACCAACGCAGATATCGATGTCTCACGTCTACCTTATTACTTGCCTTGATCGTCGGCACCCGATATTTGTCA
ACTGGGAATCCTCCTGGACCTATTATCGGTCCTTTGAAACTAACAGCAATTACCCCAGAAAGTGTCTGCTAA

>p2_ind3261

ATGCAAATAGACCCCGTTGCATATCATTGTTCCGAGTTGAAGTTCGTGCTTGTACCCGAAGAGTCGGGAAAGTTAT
CCCCGACGGAAATAGGATTCCATTTTCAGATTGGGTGCCGCGTACATGACAGAAGACCCCGCTCTGAGTCAGTGA
TGACGCACCAACGCAGATATCGATGTCTCACGTCTACCTTATTACTTGCCTTGATCGTCGGCACCCGATATTTGTCA
ACTGGGAATCCTCCTGGACCTATTATCGGTCCTTTGAAACTAACAGCAATTACCCCAGAAAGTGTCTGCTAA

>p2_ind3402

ATGCAAATAGACCCCGTTGCATATCATTGTTCCGAGTTGAAGTTCGTGCTTGTACCCGAAGAGTCGGGAAAGTTAT
CCCCGACGGAAATAGGATTCCATTTTCAGATTGGGTGCCGCGTACATGACAGAAGACCCCGCTCTGAGTCAGTGA
TGACGCTCCAACGCAGATATCGATGTCTCACGTCTACCTTATTACTTGCCTTGATCGTCGGCACCCGATATTTGTCA
ACTGGGAATCCTCCTGGACCTATTATCGGTCCTTTGAAACTAACAGCAATTACCCCAGAAAGTGTCTGCTAA

>p2_ind733

ATGCAAATAGACCCCGTTGCATATCATTGTTCCGAGTTGAAGTTCGTGCTTGTACCCGAAGAGTCGGGAAAGTTAT
CCCCGACGGAAATAGGATTCCATTTTCAGATTGGGTGCCGCGTACATGACAGAAGACCCCGCTCTGAGTCAGTGA
TGACGCACCAACGCAGATATCGATGTCTCACGACTACCTTATTACTTGCCTTGATCGTCGGCACCCGATATTTGTCA
ACTGGGAATCCTCCTGGACCTATTATCGGTCCTTTGAAACTAACAGCAATTACCCCAGAAAGTGTCTGCTAA

>p2_ind373

ATGCAAATAGACCCCGTTGCATATCATTGTTCCGAGTTGAAGTTCGTGCTTGTACCCGAAGAGTCGGGAAAGTTAT
CCCCGACGGAAATAGGATTCCATTTTCAGATTGGGTGCCGCGTACATGACAGAAGACCCCGCTCTGAGTCAGTGA
TGACGCACCAACGCAGATATCGATGTCTCACGTCTACCTTATTACTTGCCTTGATCGTCGGCACCCGATATTTGTCA
ACTGGGAATCCTCCTGGACCTATTATCGGTCCTTTGAAACTAACAGCAATTACCCCAGAAAGTGTCTGCTAA

>p2_ind2585

ATGCAAATAGACCCTGTTGCATATCATTGTTCCGAGTTGAAGTTCGTGCTTGTACCCGAAGAGTCGGGAAAGTTAT
CCCCGACGGAAATAGGATTCCATTTTCAGATTGGGTGCCGCGTACATGACAGAAGACCCCGCTCTGAGTCAGTGA
TGGCGCACCAACGCAGATATCGATGTCTCACGTCTACCTTATTACTTGCCTTGATCGTCGGCACCCGATATTTGTCA
ACTGGGAATCCTCCTGGACCTATTATCGGTCCTTTGAAACTAACAGCAATTACCCCAGAAAGTGTCTGCTAA

>p2_ind1015

ATGCAAATAGACCCCGTTGCATATCATTGTTCCGAGTTGAAGTTCGTGCTTGTACCCGAAGAGTCGGGAAAGTTAT
CCCCGACGGAAATAGGATTCCATTTTCAGATTGGGTGCCGCGTACATGACAGAAGACCCCGCCCTGAGTCAGTGA
TGACGCACCAACGCAGATATTGATGTCTCACGTCTACCTTATTACTTGCCTTGATCGTCGGCACCCGATATTTGTCA
ACTGGGAATCCTCCTGGACCTATTATCGGTCCTTTGAAACTAACAGCAATTACCCCAGAAAGTGTCTGCTAA

>p2_ind4325

ATGCAAATAGACCCCGTTGCATATCATTGTTCCGAGTTGAAGTTCGTGCTTGTACCCGAAGAGTCGGGAAAGTTAT
CCCCGACGGAAATAGGATTCCATTTTCAGATTGGGTGCCGCGTACATGACAGAAGACCCCGCCCTGAGTCAGTGA
TGACGCACCAACGCAGATATTGATGTCTCACGTCTACCTTATTACTTGCCTTGATCGTCGGCACCCGATATTTGTCA
ACTGGGAATCCTCCTGGACCTATTATCGGTCCTTTGAAACTAACAGCAATTACCCCAGAAAGTGTCTGCTAA

>p2_ind40

ATGCAAATAGACCCCGTTGCATATCATTGATCCGAGTTGAAGTTCGTGCTTGTACCCGAAGAGTCGGGAAAGTTAT
CCCCGACGGAAATAGGATTCCATTTTCAGATTGGGTGCCGCGTACATGACAGAAGACCCCGTTCTGAGTCAGTGAT
GACGCACCAACGCAGATATCGATGTCTCACGTCTACCTTATTACTTGCCTTGATCGTCGGCACCCGATATTTGTCAA
CTGGGAATCCTCCTGGACCTATTATCGGTCCTTTGAAACTAACAGCAATTACCCCAGAAAGTGTCTGCTAA

>p2_ind4853

ATGCAAATAGACCCCGTTGCATATCATTGTTCCGAGTTGAAGTTCGTGCTTGTACCCGAAGAGTCGGGAAAGTTAT
CCCCGACGGAAATAGGATTCCATTTTCAGATTGGGTGCCGCGTACATGACAGAAGACCCCGCCCTGAGTCAGTGA
TGACGCACCAACGCAGATATTGATGTCTCACGTCTACCTTATTACTTGCCTTGATCGTCGGCACCCGATATTTGTCA
ACTGGGAATCCTCCTGGACCTATTATCGGTCCTTTGAAACTAACAGCAATTACCCCAGAAAGTGTCTGCTAA

>p2_ind1445

ATGCAAATAGACCCCGTTGCATATCATTGTTCCGAGTTGAAGTTCGTGCTTGTACCCGAAGAGTCGGGAAAGTTAT
CCCCGACGGAAATAGGATTCCATTTTCAGATTGGGTGCCGCGTACATGACAGAAGACCCCGCTCTGAGTCAGTGA
TGACGCACCAACGCAGATATCGATGTCTCACGTCTACCTTATTACTTGCCTTGATCGTCGGCACCCGATATTTGTCA
ACTGGGAATCCTCCTGGACCTATTATCGGTCCTTTGAAACTAACAGCAATTACCCCAGAAAGTGTCTGCTAA

>p2_ind3630

ATGCAAATAGACCCCGTTGCATATCATTGTTCCGAGTTGAAGTTCGTGCTTGTACCCGAAGAGTCGGGAAAGTTAT
CCCCGACGGAAATAGGATTCCATTTTCAGATTGGGTGCCGCGTACATGACAGAAGACCCCGCTCTGAGTCAGTGA
TGACGCACCAACGCAGATATCGATGTCTCACGTCTACCTTATTACTTGCCTTGATCGTCGGCACCCGATATTTGTCA
ACTGGGAATCCTCCTGGACCTATTATCGGTCCTTTGAAACTAACAGCAATTACTCCAGAAAGTGTCTGCTAA

>p2_ind15

ATGCAAATAGACCCCGTTGCATATCATTGTTCCGAGTTGAAGTTCGTGCTTGTACCCGAAGAGTCGGGAAAGTTAT
CCCCGACGGAAATAGGATTCCATTTTCAGATTGGGTGCCGCGTACATGACAGAAGACCCCGCTCTGAGTCAGTGA

TGGCGCACCAACGCAAATATCGATGTCTCACGTCTACCTTATTACTTGCCTTGATCGTCGGCACCCGATATTTGTCA
ACTGGGAATCCTCCTGGACCTATTATCGGTCCTTTGAAACTAACAGCAATTACCCCAGAAAGTGTCTGCTAA

>p2_ind191

ATGCAAATAGACCCCGTTGCATATCATTGTTCCGAGTTGAAGTTCGTGCTTGTACCCGAAGAGTCGGGAAAGTTAT
CCCCGACGGAAATAGGATTCCATTTTCAGATTGGGTGCCGCGTACATGACAGAAGACCCCGCTCTGAGTCAGTGA
TGACGCACCAACGCAGATATCGATGTCTCACGTCAACCTTATTACTTGCCTTGATCGTCGGCACCCGATATTTGTCA
ACTGGGAATCCTCCTGGACCTATTATCGGTCCTTTGAAACTAACAGCAATTACCCCAGAAAGTGTCTGCTAA

>p2_ind1353

ATGCAAATAGACCCCGTTGCATATCATTGTTCCGAGTTGAAGTTCGTGCTTGTACCCGAAGAGTCGGGAAAGTTAT
CCCCGACGGAAATAGGATTCCATTTTCAGATTGGGTGCCGCGTACATGACAGAAGACCCCGCTCTGAGTCAGTGA
TGTCGCACCAACGCAGATATCGATGTCTCACGTCTACCTTATTACTTGCCTTGATCGTCGGCACCCGATATTTGTCA
ACTGGGAATCCTCCTGGACCTATTATCGGTCCTTTGAAACTAACAGCAATTACCCCAGAAAGTGTCTGCTAA

>p2_ind1504

ATGCAAATAGACCCCGTTGCATATCATTGTTCCGAGTTGAAGTTCGTGCTTGTACCCGAAGAGTCGGGAAAGTTAT
CCCCGACGGAAATAGGATTCCATTTTCAGATTGGGTGCCGCGTACATGACAGAAGACCCCGCTCTGAGTCAGTGA
TGACGCACCAACGCAGATATCGATGTCTCACGTCTACCTTATTACTTGCCTTGATCGTCGGCACCCGATATTTGTCA
ACTGGGAATCCTCCTGGACCTATTATCGGTCCTTTGAAACTAACAGCAATTACCCCAGAAAGTGTCTGCTAA

>p2_ind2446

ATGCAAATAGACCCCGTTGCATATCATTGTTCCGAGTTGAAGTTCGTGCTTGTACCCGAAGAGTCGGGAAAGTTAT
CCCCGACGGAAATAGGATTCCATTTTCAGATTGGGTGCCGCGTACATGACAGAAGACCCCGCTCTGAGTCAGTGA
TGACGCACCAACGCAGATATCGATGTCTCACGTCTACCTTATTACTTGCCTTGATCGTCGGCACCCGATATTTGTCA
ACTGGGAATCCTCCTGGACCTATTATCGGTCCTTTGAAACTAACAGCAATTACCCCAGAAAGTGTCTGCTAA

>p2_ind1483

ATGCAAATAGACCCCGTTGCATATCATTGTTCCGAGTTGAAGTTCGTGCTTGTACCCGAAGAGTCGGGAAAGTTAT
CCCCGACGGAAATAGGATTCCATTTTCAGATTGGGTGCCGCGTACATGACAGAAGACCCCGCTCTGAGTCAGTGA
TGGCGCACCAACGCAAATATCGATGTCTCACGTCTACCTTATTACTTGCCTTGATCGTCGGCACCCGATATTTGTCA
ACTGGGAATCCTCCTGGACCTATTATCGGTCCTTTGAAACTAACAGCAATTACCCCAGAAAGTGTCTGCTAA

>p2_ind2326

ATGCAAATAGACCCCGTTGCATATCATTGTTCCGAGTTGAAGTTCGTGCTTGTACCCGAAGAGTCGGGAAAGTTAT
CCCCGACGGAAATAGGATTCCATTTTCAGATTGGGTGCCGCGTACATGACAGAAGACCCCGCTCTGAGTCAGTGA
TGACGCACCAACGCAGATATCGATGTCTCACGTCTACCTTATTACTTGCCTTGATCGTCGGCACCCGATATTTGTCA
ACTGGGAATCCTCCTGGACCTATTATCGGTCCTTTGAAACTAACAGCAATTACCCCAGAAAGTGTCTGCTAA

>p2_ind3911

ATGCAAATAGACCCTGTTGCATATCATTGTTCCGAGTTGAAGTTCGTGCTTGTACCCGAAGAGTCGGGAAAGTTAT
CCCCGACGGAAATAGGATTCCATTTTCAGATTGGGTGCCGCGTACATGACAGAAGACCCCGCTCTGAGTCAGTGA
TGGCGCACCAACGCAAATACAGATGTCTCACGTCTACCTTAATACTTGCCTTGATCGTCGGCACCCGATATTTGTCA
ACTGGGAATCCTCCTGGACCTATTATCGGTCCTTTGAAACTAACAGCAATTACCCCAGAAAGTGTCTGCTAA

>p2_ind783

ATGCAAATAGACCCCGTTGCATATCATTGTTCCGAGTTGAAGTTCGTGCTTGTACCCGAAGAGTCGGGAAAGTTAT
CCCCGACGGAAATAGGATTCCATTTTCAGATTGGGTGCCGCGTACATGACAGAAGACCCCGCTCTGAGTCAGTGA
TGACGCACCAACGCAGATATCGATGTCTCACGTCTACCTTATTACTTGCCTTGATCGTCGGCACCCGATATTTGTCA
ACTGGGAATCCTCCTGGACCTATTATCGGTCCTTTGAAACTAACAGCAATTACCCCAGAAAGTGTCTGCTAA

>p2_ind1364

ATGCAAATAGACCCCGTTGCATATCATTGTTCCGAGTTGAAGTTCGTGCTTGTACCCGAAGAGTCGGGAAAGTTAT
CCCCGACGGAAATAGGATTCCATTTTCAGATTGGGTGCCGCGTACATGACAGAAGACCCCGCTCTGAGTCAGTGA
TGACGCACCAACGCAGATATCGATGTCTCACGTCTACCTTATTACTTGCCTTGATCGTCGGCACCCGATATTTGTCA
ACTGGGAATCCTCCTGGACCTATTATCGGTCCTTTGAAACTAACAGCAATTACCCCAGAAAGTGTCTGCTAA

>p2_ind1086

ATGCAAATAGACCCCGTTGCATATCATTGTTCCGAGTTGAAGTTCGTGCTTGTACCCGAAGAGTCGGGAAAGTTAT
CCCCGACGGAAATAGGATTCCATTTTCAGATTGGGTGCCGCGTACATGACAGAAGACCCCGCTCTGAGTCAGTGA
TGACGCACCAACGCAGACATCGATGTCTCACGTCTACCTTATTACTTGCCTTGATCGTCGGCACCCGATATTTGTCA
ACTGGGAATCCTCCTGGACCTATTATCGGTCCTTTGAAACTAACAGCAATTACCCCAGAAAGTGTCTGCTAA

>p2_ind1028

ATGCAAATAGACCCCGTTGCATATCATTGTTCCGAGTTGAAGTTCGTGCTTGTACCCGAAGAGTCGGGAAAGTTAT
CCCCGACGGAAATAGGATTACATTTTCAGATTGGGCGCCGCGTACATGACAGAAGACCCCGCTCTGAGTCAGTGA
TGACGCACCAACGCAGATATCGATGTCTCACGTCTACCTTATTACTTGCCTTGATCGTCGGCACCCGATATTTGTCA
ACTGGGAATCCTCCTGGACCTATTATCGGTCCTTTGAAACTAAAAGCAATTACCCCAGAAAGTGTCTGCTAA

>p2_ind3609

ATGCAAATAGACCCTGTTGCATATCATTGTTCCGAGTTGAAGTTCGTGCTTGTACCCGAAGAGTCGGGAAAGTTAT
CCCCGACGGAAATAGGATTCCATTTTCAGATTGGGTGCCGCGTACATGACAGCAGACCCCGCTCTGAGTCAGTGAT
GGCGCACCAACGCAAATATCGATGTCTCACGTCTACCTTATTACTTGCCTTGATCGTCGGCACCCGATATTTGTCAA
CTGGGAATCCTCCTGGACCTATTATCGGTCCTTTGAAACTAACAGCAATTACCCCAGAAAGTGTCTGCTAA

>p2_ind2717

ATGCAAATAGACCCCGTTGCATATCATTGTTCCGAGTTGAAGTTCGTGCTTGTACCCGAAGAGTCGGGAAAGTTAT
CCCCGACGGAAATAGGATTCCATTTTCAGATTGGGTGCCGCGTACATGACAGAAGACCCCGCTCTGAGTCAGTGA

TGACGCACCAACGCAGATATCGATGTCTCACGTCTACCTTATTACTTGCCTTGATCGTCGGCACCCGATATTTGTCA
ACTGGGAATCCTCCTGGACCTATTATCGGTCCTTTGAAACTAACAGCAATTACCCCAGAAAGTGTCTGCTAA

>p2_ind3751

ATGCAAATAGACCCCGTTGCATATCATTGTTCCGAGTTGAAGTTCGTGCTTGTACCCGAAGAGTCGGGAAAGTTAT
CCCCGACGGAAATAGGATTCCATTTTCAGATTGGGTGCCGCGTACATGACAGAAGACCCCGCTCTGAGTCAGTGA
TGACGCACCAACGCAGATATCGATGTCTCACGTCTACCTTATTACTTGCCTTGATCGTCGGCACCCGATATTTGTCA
ACTGGGAATCCTCCTGGACCTATTATCGGTCCTTTGAAACTAACGGCAATTACCCCAGAAAGTGTCTGCTAA

>p2_ind4072

ATGCAAATAGACCCCGTTGCATATCATTGTTCCGAGTTGAAGTTCGTGCTTGTACCCGAAGAGTCGGGAAAGTTAT
CCCCGACGGAAATAGGATTCCATTTTCAGATTGGGTGCCGCGTACATGACAGAAGACCCCGCTCTGAGTCAGTGA
TGACGCACCAACGCAGATATCGATGTCTCTCGTCTACCTTATTACTTGCCTTGATCGTCGGCACCCGATATTTGTCA
ACTGGGAATCCTCCTGGACCTATTATCGGTCCTTTGAAACTAACAGCAATTACCCCAGAAAGTGTCTGCTAA

>p2_ind2392

ATGCAAATAGACCCCGTTGCATATCATTGTTCCGAGTTGAAGTTCGTGCTTGTACCCGAAGAGTCGGGAAAGTTAT
CCCCGACGGAAATAGGATTCCATTTTCAGATTGGGTGCCGCGTACATGACAGAAGACCCCGCTCTGAGTCAGTGA
TGACGCACCTACGCAGATATCGATGTCTCACGTCTACCTTATTACTTGCCTTGATCGTCGGCACCCGATATTTGTCA
ACTGGGAATCCTCCTGGACCTATTATCGGTCCTTTGAAACTAACAGCAATTACCCCAGAAAGTGTCTGCTAA

>p2_ind3950

ATGCAAATAGACCCCGTTGCATATCATTGTTCCGAGTTGAAGTTCGTGCTTGTACCCGAAGAGTCGGGAAAGTTAT
CCCCGACGGAAATAGGATTCCATTTTCAGATTGGGTGCCGCGTACATGACAGAAGACCCCGCTCTGAGTCAGTGA
TGGCGCACCAACGCAGATATCGATGTCTCACGTCTACCTTATTACTTGCCTTGATCGTCGGCACCCGATATTTGTCA
ACTGGGAATCCTCCTGGACCTATTATCGGTCCTTTGAAACTAACAGCAATTACCCCAGAAAGTGTCTGCTAA

>p2_ind2593

ATGCAAATAGACCCCGTTGCATATCATTGTTCCGAGTTGAAGTTCGTGCTTGTACCCGAAGAGTCGGGAAAGTTAT
CCCCGACGGAAATAGGATTCCATTTTCAGATTGGGTGCCGCGTACATGACAGAAGACCCCGCTCTGAGTCAGTGA
TGACGCACCAACGCAGATATCGATGTCTCACGTCTACCTTATTACTTGCCTTGATCGTCGGCACCCGATATTTGTCA
ACTGGGAATCCTCCTGGACCTATTATCGGTCCTTTGAAACTAACAGCAATTACCCCAGAAAGTGTCTGCTAA

>p2_ind339

ATGCAAATAGACCCCGTTGCATATCATTGTTCCGAGTTGAAGTTCGTGCTTGTACCCGAAGAGTCGGGAAAGTTAT
CCCCGACGGAAATAGGATTCCATTTTCAGATTGGGTGCCGCGTACATGACAGAAGACCCCGCTCTGAGTCAGTGA
TGACGCACCAACGCAGATATCGATGTCTCACGTCTACCTTATTACTTGCCTTGATCGTCGGCACCCGATATTTGTCA
ACTGGGAATCCTCCTGGACCTATTATCGGTCCTTTGAAACTAACAGCAATTACCCCAGAAAGTGTCTGCTAA

>p2_ind286

ATGCAAATAGACCCCGTTGCATATCATTGTTCCGAGTTGAAGTTCGTGCTTGTACCCGAAGAGTCGGGAAAGTTAT
CCCCGACGGAAATAGGATTCCATTTTCAGATTGGGTGCCGCGTACATGACAGAAGACCCCGCTCTGAGTCAGTGA
TGACGCACCAACGCAGATATCGATGTCTCACGTCTACCTTATTACTTGCCTTGATCGTCGGCACCCGATATTTGTCA
ACTGGGAATCCTCCTGGACCTATTATCGGTCCTTTGAAACTAACAGCAATTACCCCAGAAAGTGTCTGCTAA

>p2_ind2936

ATGCAAATAGACCCCGTTGCATATCATTGATCCGAGTTGAAGTTCGTGCTTGTACCCGAAGAGTCGGGAAAGTTAT
CCCCGACGGAAATAGGATTCCATTTTCAGATTGGGTGCCGCGTACATGACAGAAGACCCCGTTCTGAGTCAGTGAT
GACGCACCAACGCAGATATCGATGTCTCACGTCTACCTTATTACTTGCCTTGATCGTCGGCACCCGATATTTGTCAA
CTGGGAATCCTCCTGGACCTATTATCGGTCCTTTGAAACTAACAGCAATTACCCCAGAAAGTGTCTGCTAA

>p2_ind3377

ATGCAAATAGACCCCGTTGCATATCATTGTTCCGAGTTGAAGTTCGTGCTTGTACCCGAAGAGTCGGGAAAGTTAT
CCCCGACGGAAATAGGATTCCATTTTCAGATTGGGTGCCGCGTACATGACAGAAGACCCCGCTCTGAGTCAGTGA
TGACGCACCAACGCAGATATCGATGTCTCACGTCTACCTTATTACTTGCCTTGATCGTCGGCACCCGATATTTGTCA
ACTGGGAATCCTCCTGGACCTATTATCGGTCCTTTGAAACTAACAGCAATTACCCCAGAAAGTGTCTGCTAA

>p2_ind1952

ATGCAAATAGACCCCGTTGCATATCATTGTTCCGAGCTGAAGTTCGTGCTTGTACCCGAAGAGTCGGGAAAGTTAT
CCCCGACGGAAATAGGATTCCATTTTCAGATTGGGTGCCGCGTACATGACAGAAGACCCCGCTCTGAGTCAGTGA
TGACGCACCAACGCAGATATCGATGTCTCACGTCTACCTTATTACTTGCCTTGATCGTCGGCACCCGATATTTGTCA
ACTGGGAATCCTCCTGGACCTATTATCGGTCCTTTGAAACTAACAGGAATTACCCCAGAAAGTGTCTGCTAA

>p2_ind1255

ATGCAAATAGACCCCGTTGCATATCATTGTTCCGAGTTGAAGTTCGTGCTTGTACCCGAAGAGTCGGGAAAGTTAT
CCCCGACGGAAATAGGATTCCATTTTCAGATTGGGTGCCGCGTACATGACAGAAGACCCCGCTCTGAGTCAGTGA
TGACGCACCAACGCAGATATCGATGTCTCACGTCTACCTTATTACTTGCCTTGATCGTCGGCACCCGATATTTGTCA
ACTGGGAATCCTCCTGGACCTATTATCGGTCCTTTGAAACTAACAGCAATTACCCCAGAAAGTGTCTGCTAA

>p2_ind4550

ATGCAAATAGACCCCGTTGCATATCATTGTTCCGAGTTGAAGTTCGTGCTTGTACCCGAAGAGTCGGGAAAGTTAT
CCCCGACGGAAATAGGATTCCATTTTCAGATTGGGTGCCGCGTACATGACAGAAGACCCCGCTCTGAGTCAGTGA
TGACGCACCAACGCAGATATCGATGTCTCACGTCTACCTTATTACTTGCCTTGATCGTCGGCACCCGATATTTGTCA
ACTGGGAATCCTCCTGGACCTATTATCGGTCCTTTGAAACTAACAGCAATTACCCCAGAAAGTGTCTGCTAA

>p2_ind3186

ATGCAAATAGACCCCGTTGCATATCATTGTTCCGAGTTGAAGTTCGTGCTTGTACCCGAAGAGTCGGGAAAGTTAT
CCCCGACGGAAATAGGATTCCATTTTCAGATTGGGTGCCGCGTACATGACAGAAGACCCCGCTCTGAGTCAGTGA

TGACGCACCAACGCAGATATCGATGTCTCACGTCTACCTTATTACTTGCCTTGATCGTCGGCACCCGATATTTGTCA
ACTGGGAATCCTCCTGGACCTATTATCGGTCCTTTGAAACTAACAGCAATTACCCCAGAAAGTGTCTGCTAA

>p2_ind3331

ATGCAAATAGACCCCGTTGCATATCATTGTTCCGAGTTGAAGTTCGTGCTTGTACCCGAAGAGTCGGGAAAGTTAT
CCCCGACGGAAATAGGATTCCATTTTCAGATTGGGTGCCGCGTACATGACAGAAGACCCCGCTCTGAGTCAGTGA
TGACGCACCAACGCAGATATCGATGTCTCACGTCTACCTTATTACTTGCCTTGATCGTCGGCACCCGATATTTGTCA
ACTGGGAATCCTCCTGGACCTATTATCGGTCCTTTGAAACTAACAGCAATTACCCCAGAAAGTGTCTGCTAA

>p2_ind3404

ATGCAAATAGACCCCGTTGCATATCATTGATCCGAGTTGAAGTTCGTGCTTGTACCCGAAGAGTCGGGAAAGTTAT
CCCCGACGGAAATAGGATTCCATTTTCAGATTGGGTGCCGCGTACATGACAGAAGACCCCGTTCTGAGTCAGTGAT
GACGCACCAACGCAGATATCGATGTCTCACGTCTACCTTATTACTTGCCTTGATCGTCGGCACCCGATATTTGTCAA
CTGGGAATCCTCCTGGACCTATTATCGGTCCTTTGAAACTAACAGCAATTACCCCAGAAAGTGTCTGCTAA

>p2_ind1085

ATGCAAATAGACCCCGTTGCATATCATTGTTCCGAGTTGAAGTTCGTGCTTGTACCCGAAGAGTCGGGAAAGTTAT
CCCCGACGGAAATAGGATTCCATTTTCAGATTGGGTGCCGCGTACATGACAGAAGACCCCGCTCTGAGTCAGTGA
TGACGCACCAACGCAGATATCGATGTCTCACGTCTACCTTATTACTTGCCTTGATCGTCGGCACCCGATATTTGTCA
ACTGGGAATCCTCCTGGACCTATTATCGGTCCTTTGAAACTAACAGCAATTACCCCAGAAAGTGTCTGCTAA

>p2_ind3087

ATGCAAATAGACCCCGTTGCATATCATTGATCCGAGTTGAAGTTCGTGCTTGTACCCGAAGAGTCGGGAAAGTTAT
CCCCGACGGAAATAGGATTCCATTTTCAGATTGGGTGCCGCGTACATGACAGAAGACCCCGTTCTGAGTCAGTGAT
GACGCACCAACGCAGATATCGATGTCTCACGTCTACCTTATTACTTGCCTTGATCGTCGGCACCCGATATTTGTCAA
CTGGGAATCCTCCTGGACCTATTATCGGTCCTTTGAAACTAACAGCAATTACCCCAGAAAGTGTCTGCTAA

>p2_ind4677

ATGCAAATAGACCCCGTTGCATATCATTGTTCCGAGTTGAAGTTCGTGCTTGTACCCGAAGAGTCGGGAAAGTTAT
CCCCGACGGAAATAGGATTCCATTTTCAGATTGGGTGCCGCGTACATGACAGAAGACCCCGCTCTGAGTCAGTGA
TGACGCACCAACGCAGATATCGATGTCTCACGTCTACCTTATTACTTGCCTTGATCGTCGGCACCCGATATTTGTCA
ACTGGGAATCCTCCTGGACCTATTATCGGTCCTTTGAAACTAACAGCAATTACCCCAGAAAGTGTCTGCTAA

>p2_ind142

ATGCAAATAGACCCCGTTGCATATCATTGTTCCGAGTTGAAGTTCGTGCTTGTACCCGAAGAGTCGGGAAAGTTAT
CCCCGACGGAAATAGGATTCCATTTTCAGATTGGACGCCGCGTACATGACAGAAGACCCCGCTCTGAGTCAGTGA
TGACGCACCAACGCAGATATCGATGTCTCACGTCTACCTTATTACTTGCCTTGATCGTCGGCACCCGATATTTGTCA
ACTGGGAATCCTCCTGGACCTATTATCGGTCCTTTGAAACTAACAGCAATTACCCCAGAAAGTGTCTGCTAA

>p2_ind2998

ATGCAAATAGACCCCGTTGCATATCATTGTTCCGAGTTGAAGTTCGTGCTTGTACCCGAAGAGTCGGGAAAGTTAT
CCCCGACGGAAATAGGATTCCATTTTCAGACTGGGCGCCGCGTACATGACAGAAGACCCCGCTCTGAGTCAGTGA
TGACGCACCAACGCAGACATCGATGTCTCACGTCTACCTTATTACTTGCCTTGATCGTCGGCACCCGATATTTGTCA
ACTGGGAATCCTCCTGGACCTATTATCGGTCCTTTGAAACTAACAGCAATTACCCCAGAAAGTGTCTGCTAA

>p2_ind3809

ATGCAAATAGACCCCGTTGCATATCATTGTTCCGAGTTGAAGTTCGTGCTTGTACCCGAAGAGTCGGGAAAGTTAT
CCCCGACGGAAATAGGATTCCATTTTCAGATTGGGTGCCGCGTACATGACAGAAGACCCCGCTCTGAGTCAGTGA
TGACGCACCAACGCAGATATCGATGTCTCACGTCTACCTTATTACTTGCCTTGATCGTCGGCACCCGATATTTGTCA
ACTGGGAATCCTCCTGGACCTATTATCGGTCCTTTGAAACTAACAGCAATTACCCCAGAAAGTGTCTGCTAA

>p2_ind3577

ATGCAAATAGACCCCGTTGCATATCATTGTTCCGAGTTGAAGTTCGTGCTTGTACCCGAAGAGTCGGGAAAGTTAT
CCCCGACGGAAATAGGATTCCATTTTCAGATTGGGCGCCGCGTACATGACAGAAGACCCCGCTCTGAGTCAGTGA
TGACGCACCAACGCAGATATCGATGTCTCACGTCTACCTTATTACTTGCCTTGATCGTCGGCACCCGATATTTGTCA
ACTGGGAATCCTCCTGGACCTATTATCGGTCCTTTGAAACTAACAGCAATTACCCCAGAAAGTGTCTGCTAA

>p2_ind1

ATGCAAATAGACCCCGTTGCATATCATTGTTCCGAGTTGAAGTTCGTGCTTGTACCCGAAGAGTCGGGAAAGTTAT
CCCCGACGGAAATAGGATTCCATTTTCAGATTGGGTGCCGCGTACATGACAGAAGACCCCGCTCTGAGTCAGTGA
TGACGCACCAACGCAGATATCGATGTCTCACGTCTACCTTATTACTTGCCTTGATCGTCGGCACCCGATATTTGTCA
ACTGGGAATCCTCCTGGACCTATTATCGGTCCTTTGAAACTAACAGCAATTACCCCAGAAAGTGTCTGCTAA

>p2_ind2243

ATGCAAATAGACCCCGTTGCATATCATTGTTCCGAGTTGAAGTTCGTGCTTGTACCCGAAGAGTCGGGAAAGTTAT
CCCCGACGGAAATAGGATTCCATTTTCAGATTGGGTGCCGCGTACATGACAGAAGACCCCGCTCTGAGTCAGTGA
TGACGCACCAACGCAGATATCGATGTCTCACGTCTACCTTATTACTTGCCTTGATCGTCGGCACCCGATATTTGTCA
ACTGGGAATCCTCCTGGACCTATTATCGGTCCTTTGAAACTAAAAGCAATTACCCCAGAAAGTGTCTGCTAA

>p2_ind1963

ATGCAAATAGACCCCGTTGCATATCATTGTTCCGAGTTGAAGTTCGTGCTTGTACCCGAAGAGTCGGGAAAGTTAT
CCCCGACGGAAATAGGATTCCATTTTCAGATTGGGTGCCGCGTACATGACAGAAGACCCCGCTCTGAGTCAGTGA
TGGCGCACCAACGCAAATATCGATGTCTCACGTCTACCTTATTACTTGCCTTGATCGTCGGCACCCGATATTTGTCA
ACTGGGAATCCTCCTGGACCTATTATCGGTCCTTTGAAACTAACAGCAATTACCCCAGAAAGTGTCTGCTAA

>p2_ind1157

ATGCAAATAGACCCCGTTGCATATCATTGTTCCGAGTTGAAGTTCGTGCTTGTATCCGAAGAGTCGGGAAAGTTAT
CCCCGACGGAAATAGGATTCCATTTTCAGATTGGGTGCCGCGTACATGACAGAAGACCCCGCTCTGAGTCAGTGA

TGACGCACCAACGCAGATATCGATGTCTCACGTCTACCTTATTACTTGCCTTGATCGTCGGCACCCGATATTTGTCA
ACTGGGAATCCTCCTGGACCTATTATCGGTCCTTTGAAACTAACAGCAATTACCCCAGAAACTGTCTGCTAA

>p2_ind3178

ATGCAAATAGACCCCGTTGCATATCATTGTTCCGAGTTGAAGTTCGTGCTTGTACCCGAAGAGTCGGGAAAGTTAT
CCCCGACGGAAATAGGATTCCATTTTCAGATTGGGTGCCGCGTACATGACAGAAGACCCCGCTCTGAGTCAGTGA
TGACGCACCAACGCAGATATCGATGTCTCACGTCTACCTTATTACTTGCCTTGATCGTCGGCACCCGATATTTGTCA
ACTGGGAATCCTCCTGGACCTATTATCGGTCCTTTGAAACTAACAGCAATTACCCCAGAAAGTGTCTGCTAA

>p2_ind278

ATGCAAATAGACCCCGTTGCATATCATTGTTCCAAGTTGAAGTTCGTGCTTGTACCCGAAGAGTCGGGAAAGTTAT
CCCCGACGGAAATAGGATTCCATTTTCAGATTGGGTGCCGCGTACATGACAGAAGACCCCGCTCTGAGTCAGTGA
TGACGCACCAACGCAGATATCGATGTCTCACGTCTACCTTATTACTTGCCTTGATCGTCGGCACCCGATATTTGTCA
ACTGGGAATCCTCCTGGACCTATTATCGGTCCTTTGAAACTAACAGCAATTACCCCAGAAAGTGTCTGCTAA

>p2_ind4688

ATGCAAATAGACCCCGTTGCATATCATTGATCCGAGTTGAAGTTCGTGCTTGTACCCGAAGAGTCGGGAAAGTTAT
CCCCGACGGAAATAGGATTCCATTTTCAGATTGGGTGCCGCGTACATGACAGAAGACCCCGCTCTGAGTCAGTGA
TGACGCACCAACGCAGATATCGATGTCTCACGTCTACCTTATTACTTGCCTTGATCGTCGGCACCCGATATTTGTCA
ACTGGGAATCCTCCTGGACCTATTATCGGTCCTTTGAAACTAACAGCAATTACCCCAGAAAGTGTCTGCTAA

>p2_ind653

ATGCAAATAGACCCCGTTGCATATCATTGTTCCGAGTTGAAGTTCGTGCTTGTACCCGAAGAGTCGGGAAAGTTAT
CCCCGACGGAAATAGGATTCCATTTTCAGATTGGGTGCCGCGTACATGACAGAAGACCCCGCTCTGAGTCAGTGA
TGACGCACCAACGCAGATATCGATGTCTCACGTCTACCTTATTACTTGCCTTGATCGTCGGCACCCGATATTTGTCA
ACTGGGAATCCTCCTGGACCTATTATCGGTCCTTTGAAACTAACAGCAATTACCCCAGAAAGTGTCTGCTAA

>p2_ind3030

ATGCAAATAGACCCCGTTGCATATCATTGTTCCGAGTTGAAGTTCGTGCTTGTACCCGAAGAGTCGGGAAAGTTAT
CCCCGACGGAAATAGGATTCCATTTTCAGATTGGGTGCCGCGTACATGACAGAAGACCCCGCTCTGAGTCAGTGA
TGTCGCACCAACGCAGATATCGATGTCTCACGTCTACCTTATTACTTGCCTTGATCGTCGGCACCCGATATTTGTCA
ACTGGGAATCCTCCTGGACCTATTATCGGTCCTTTGAAACTAACAGCAATTACCCCAGAAAGTGTCTGCTAA

>p2_ind4668

ATGCAAATAGACCCCGTTGCATATCATTGTTCCGAGTTGAAGTTCGTGCTTGTACCCGAAGAGTCGGGAAAGTTAT
CCCCGACGGAAATAGGATTCCATTTTCAGATTGGGTGCCGCGTACATGACAGAAGACCCCGCTCTGAGTCAGTGA
TGACGCACCAACGCAGATATCGATGTCTCACGTCTACCTTATTACTTGCCTTGATCGTCGGCACCCGATATTTGTCA
ACTGGGAATCCTCCTGGACCTATTATCGGTCCTTTGAAACTAACAGCAATTACCCCAGAAAGTGTCTGCTAA

>p2_ind2190

ATGCAAATAGACCCCGTTGCATATCATTGTTCCGAGTTGAGGTTCGTGCTTGTACCCGAAGAGTCGGGAAAGTTAT
CCCCGACGGAAATAGGATTCCATTTTCAGATTGGGTGCCGCGTACATGACAGAAGACCCCGCTCTGAGTCAGTTAT
GACGCACCAACGCAGATATCGATGTCTCACGTCTACCTTATTACTTGCCTTGACCGTCGGCACCCGATATTTGTCAA
CTGGGAATCCTCCTAGACCTATTATCGGTCCTTTGAAACTAACAGCAATTACCCCAGAAAGTGTCTGCTAA

>p2_ind220

ATGCAAATAGACCCCGTTGCATATCATTGATCCGAGTTGAAGTTCGTGCTTGTACCCGAAGAGTCGGGAAAGTTAT
CCCCGACGGAAATAGGATTCCATTTTCAGATTGGGTGCCGCGTACATGACAGAAGACCCCGCTCTGAGTCAGTGA
TGACGCACCAACGCAGATATCGATGTCTCACGTCTACCTTATTACTTGCCTTGATCGTCGGCACCCGATATTTGTCA
ACTGGGAATCCTCCTGGACCTATTATCGGTCCTTTGAAACTAACAGCAATTACCCCAGAAAGTGTCTGCTAA

>p2_ind1939

ATGCAAATAGACCCCGTTGCATATCATTGTTCCGAGTTGAAGTTCGTGCTTGTACCCGAAGAGTCGGGAAAGTTAT
CCCCGACGGAAATAGGATTCCATTTTCAGATTGGGTGCCGCGTACATGACAGAAGACCCCGCTCTGAGTCAGTGA
TGACGCACCAACGCAGATATCGATGTCTCACGTCTACCTTATTACTTGCCTTGATCGTCGGCACCCGATATTTGTCA
ACTGGGAATCCTCCTGGACCTATTATCGGTCCTTTGAAACTAACAGCAATTACCCCAGAAAGTGTCTGCTAA

>p2_ind657

ATGCAAATAGACCCCGTTGCATATCATTGTTCCGAGTTGAAGTTCGTGCTTGTACCCGAAGAGTCGGGAAAGTTAT
CCCCGACGGAAATAGGATTCCATTTTCAGATTGGGTGCCGCGTACATGACAGAAGACCCCGCTCTGAGTCAGTGA
TGTCGCACCAACGCAGATATCGATGTCTCACGTCTACCTTATTACTTGCCTTGATCGTCGGCACCCGATATTTGTCA
ACTGGGAATCCTCCTGGACCTATTATCGGTCCTTTGAAACTAACAGCAATTACCCCAGAAAGTGTCTGCTAA

>p2_ind4336

ATGCAAATAGACCCCGTTGCATATCATTGATCCGAGTTGAAGTTCGTGCTTGTACCCGAAGAGTCGGGAAAGTTAT
CCCCGACGGAAATAGGATTCCATTTTCAGATTGGGTGCCGCGTACATGACAGAAGACCCCGTTCTGAGTCAGTGAT
GACGCACCAACGCAGATATCGATGTCTCACGTCTACCTTATTACTTGCCTTGATCGTCGGCACCCGATATTTGTCAA
CTGGGAATCCTCCTGGACCTATTATCGGTCCTTTGAAACTAACAGCAATTACCCCAGAAAGTGTCTGCTAA

>p2_ind1226

ATGCAAATAGACCCCGTTGCATATCATTGATCCGAGTTGAAGTTCGTGCTTGTACCCGAAGAGTCGGGAAAGTTAT
CCCCGACGGAAATAGGATTCCATTTTCAGATTGGGTGCCGCGTACATGACAGAAGACCCCGTTCTGAGTCAGTGAT
GACGCACCAACGCAGATATCGATGTCTCACGTCTACCTTATTACTTGCCTTGATCGTCGGCACCCGATATTTGTCAA
CTGGGAATCCTCCTGGACCTATTATCGGTCCTTTGAAACTAACAGCAATTACCCCAGAAAGTGTCTGCTAA

>p2_ind719

ATGCAAATAGACCCCGTTGCATATCATTGTTCCGAGTTGAAGTTCGTGCTTGTACCCGAAGAGTCGGGAAAGTTAT
CCCCGACGGAAATAGGATTCCATTTTCAGATTGGGTGCCGCGTACATGACAGAAGACCCCGCTCTGAGTCAGTGA

TGACGCACCAACGCAGATATCGATGTCTCACGTCTACCTTATTACTTGCCTTGATCGTCGGCACCCGATATTTGTCA
ACTGGGAATCCTCCTGGACCTATTATCGGTCCTTTGAAACTAACAGCAATTACCCCAGAAAGTGTCTGCTAA

>p2_ind1219

ATGCAAATAGACCCCGTTGCATATCATTGTTCCGAGTTGAGGTTCGTGCTTGTACCCGAAGAGTCGGGAAAGTTAT
CCCCGACGGAAATAGGATTCCATTTTCAGATTGGGTGCCGCGTACATGACAGAAGACCCCGCTCTGAGTCAGTTAT
GACGCACCAACGCAGATATCGATGTCTCACGTCTACCTTATTACTTGCCTTGACCGTCGGCACCCGATATTTGTCAA
CTGGGAATCCTCCTGGACCTATTATCGGTCCTTTGAAACTAACAGCAATTACCCCAGAAAGTGTCTGCTAA

>p2_ind440

ATGCAAATAGACCCCGTTGCATATCATTGTTCCGAGTTGAAGTTCGTGCTTGTACCCGAAGAGTCGGGAAAGTTAT
CCCCGACGGAAATAGGATTCCATTTTCAGATTGGGTGCCGCGTACATGACAGAAGACCCCGCTCTGAGTCAGTGA
TGACGCACCAACGCAGATATCGATGTCTCACGTCTACCTTATTACTTGCCTTGATCGTCGGCACCCGATATTTGTCA
ACTGGGAATCCTCCTGGACCTATTATCGGTCCTTTGAAACTAACGGCAATTACCCCAGAAAGTGTCTGCTAA

>p2_ind194

ATGCAAATAGACCCCGTTGCATATCATGGTTCCGAGTTGAAGTTCGTGCTTGTACCCGAAGAGTCGGGAAAGTTAT
CCCCGACGGAAATAGGATTCCATTTTCAGATTGGGTGCCGCGTACATGACAGAAGACCCCGCTCTGAGTCAGTGA
TGACGCACCAACGCAGATATCGATGTCTCACGTCTACCTTATTACTTGCCTTGATCGTCGGCACCCGATATTTGTCA
ACTGGGAATCCTCCTGGACCTATTATCGGTCCTTTGAAACTAACAGCAATTACCCCAGAAAGTGTCTGCTAA

>p2_ind3699

ATGCAAATAGACCCCGTTGCATATCATTGATCCGAGTTGAAGTTCGTGCTTGTACCCGAAGAGTCGGGAAAGTTAT
CCCCGACGGAAATAGGATTCCATTTTCAGATTGGGTGCCGCGTACATGACAGAAGACCCCGTTCTGAGTCAGTGAT
GACGCACCAACGCAGATATCGATGTCTCACGTCTACCTTATTACTTGCCTTGATCGTCGGCACCCGATATTTGTCAA
CTGGGAATCCTCCTGGACCTATTATCGGTCCTTTGAAACTAACAGCAATTACCCCAGAAAGTGTCTGCTAA

>p2_ind3389

ATGCAAATAGACCCCGTTGCATATCATTGTTCCGAGTTGAAGTTCGTGCTTGTACCCGAAGAGTCGGGAAAGTTAT
CCCCGACGGAAATAGGATTCCATTTTCAGATTGGGTGCCGCGTACATGACAGAAGACCCCGCTCTGAGTCAGTGA
TGACGCACCAACGCAGATATCGATGTCTCACGTCTACCTTATTACTTGCCTTGATCGTCGGCACCCGATATTTGTCA
ACTGGGAATCCTCCTGGACCTATTATCGGTCCTTTGAAACTAACAGCAATTACCCCAGAAAGTGTCTGCTAA

>p2_ind1343

ATGCAAATAGACCCCGTTGCATATCATTGTTCCGAGTTGAAGTTCGTGCTTGTACCCGAAGAGTCGGGAAAGTTAT
CCCCGACGGAAATAGGATTCCATTTTCAGATTGGGCGCCGCGTACATGACAGAAGACCCCGCTCTGAGTCAGTGA
TGACGCACCAACGCAGATATCGATGTCTCACGTCTACCTTATTACTTGCCTTGATCGTCGGCACCCGATATTTGTCA
ACTGGGAATCCTCCTGGACCTATTATCGGTCCTTTGAAACTAACAGCAATTACCCCAGAAAGTGTCTGCTAA

>p2_ind716

ATGCAAATAGACCCCGTTGCATATCATTGTTCCGAGTTGAAGTTCGTGCTTGTACCCGAAGAGTCGGGAAAGTTAT
CCCCGACGGAAATAGGATTCCATTTTCAGATTGGGTGCCGCGTACATGACAGAAGACCCCGCTCTGAGTCAGTGA
TGACGCACCAACGCAGATATCGATGTCTCACGTCTACCTTATTACTTGCCTTGATCGTCGGCACCCGATATTTGTCA
ACTGGGAATCCTCCTGGACCTATTATCGGTCCTTTGAAACTAACAGCAATTACCCCAGAAAGTGTCTGCTAA

>p2_ind2144

ATGCAAATAGACCCCGTTGCATATCATTGTTCCGAGTTGAAGTTCGTGCTTGTACCCGAAGAGTCGGGAAAGTTAT
CCCCGACGGAAATAGGATTCCATTTTCAGATTGGGTGCCGCGTACATGACAGAAGACCCCGCTCTGAGTCAGTGA
TGACGCACCAACGCAGATATCGATGTCTCACGTCTACCTTATTACTTGCCTTGATCGTCGGCACCCGATATTTGTCA
ACTGGGAATCCTCCTGGACCTATTATCGGTCCTTTGAAACTAACAGCAATTACCCCAGAAAGTGTCTGCTAA

>p2_ind72

ATGCAAATAGACCCCGTTGCATATCATTGTTCCGAGTTGAAGTTCGTGCTTGTACCCGCAGAGTCGGGAAAGTTAT
CCCCGACGGAAATAGGATTCCATTTTCAGATTGGGTGCCGCGTACATGACAGAAGACCCCGCTCTGAGTCAGTGA
TGACGCACCAACGCAGATATCGATGTCTCACGTCTACCTTATTACTTGCCTTGATCGTCGGCACCCGATATTTGTCA
ACTGGGAATCCTCCTGGACCTATTATCGGTCCTTTGAAACTAACAGCAATTACCCCAGAAAGTGTCTGCTAA

>p2_ind4773

ATGCAAATAGACCCCGTTGCATATCATTGTTCCGAGTTGAAGTTCGTGCTTGTACCCGAAGAGTCGGGAAAGTTAT
CCCCGACGGAAATAGGATTCCATTTTCAGATTGGGTGCCGCGTACATGACAGAAGACCCCGCTCTGAGTCAGTGA
TGACGCACCAACGCAGACATCGATGTCTCACGTCTACCTTATTACTTGCCTTGATCGTCGGCACCCGATATTTGTCA
ACTGGGAATCCTCCTGGACCTATTATCGGTCCTTTGAAACTAACAGCAATTACCCCAGAAAGTGTCTGCTAA

>p2_ind3676

ATGCAAATAGACCCCGTTGCATATCATTGTTCCGAGTTGAAGTTCGTGCTTGTACCCGAAGAGTCGGGAAAGTTAT
CCCCGACGGAAATAGGATTCCATTTTCAGATTGGGCGCCGCGTACATGACAGAAGACCCCGCTCTGAGTCAGTGA
TGACGCACCAACGCAGATATCGATGTCTCACGTCTACCTTATTACTTGCCTTGATCGTCGGCACCCGATATTTGTCA
ACTGGGAATCCTCCTGGACCTATTATCGGTCCTTTGAAACTAACAGCAATTACCCCAGAAAGTGTCTGCTAA

>p2_ind3552

ATGCAAATAGACCCCGTTGCATATCATTGTTCCGAGTTGAAGTTCGTGCTTGTACCCGAAGAGTCGGGAAAGTTAT
CCCCGACGGAAATAGGATTCCATTTTCAGATTGGGTGCCGCGTACATGACAGAAGACCCCGCTCTGAGTCAGTGA
TGACGCACCAACGCAGATATCGATGTCTCACGTCTACCTTATTACTTGCCTTGATCGTCGGCACCCGATATTTGTCA
ACTGGGAATCCTCCTGGACCTATTATCGGTCCTTTGAAACTAACAGCAATTACTCCAGAAAGTGTCTGCTAA

>p2_ind2319

ATGCAAATAGACCCCGTTGCATATCATTGTTCCGAGTTGAAGTTCGTGCTTGTACCCGAAGAGTCGGGAAAGTTAT
CCCCGACGGAAATAGGATTCCATTTTCAGATTGGGTGCCGCGTACATGACAGAAGACCCCGCTCTGAGTCAGTGA

TGACGCACCAACGCAGATATCGATGTCTCACGTCTACCTTATTACTTGCCTTGATCGTCGGCACCCGATATTTGTCA
ACTGGGAATCCTCCTGGACCTATTATCGGTCCTTTGAAACTAACAGCAATTACACCAGAAAGTGTCTGCTAA

>p2_ind2462

ATGCAAATAGACCCCGTTGCATATCATTGTTCCGAGTTGAAGTTCGTGCTTGTACCCGAAGAGTCGGGAAAGTTAT
CCCCGACGGAAATAGGATTCCATTTTCAGATTGGGTGCCGCGTACATGACAGAAGACCCCGCTCTGAGTCAGTGA
TGACGCACCAACGCAGATATCGATGTCTCACGTCTACCTTATTACTTGCCTTGATCGTCGGCACCCGATATTTGTCA
ACTGGGAATCCTCCTGGACCTATTATCGGTCCTTTGAAACTAACAGCAATTACTCCAGAAAGTGTCTGCTAA

>p2_ind4519

ATGCAAATAGACCCCGTTGCATATCATTGTTCCGAGTTGAAGTTCGTGCTTGTACCCGAAGAGTCGGGAAAGTTAT
CCCCGACGGAAATAGGATTCCATTTTCAGACTGGGCGCCGCGTACATGACAGAAGACCCCGCTCTGAGTCAGTGA
TGACGCACCAACGCAGATATCGATGTCTCACGTCTACCTTATTACTTGCCTTGATCGTCGGCACCCGATATTTGTCA
ACTGGGAATCCTCCTGGACCTATTATCGGTCCTTTGAAACTAACAGCAATTACCCCAGAAAGTGTCTGCTAA

>p2_ind905

ATGCAAATAGACCCCGTTGCATATCATTGTTCCGAGTTGAAGTTCGTGCTTGTACCCGAAGAGTCGGGAAAGTTAT
CCCCGACGGAAATAGGATTCCATTTTCAGATTGGGTGCCGCGTACATGACAGAAGACCCCGCTCTGAGTCAGTGA
TGACGCACCAACGCAGATATCGATGTCTCACGTCTACCTTATTACTTGCCTTGATCGTCGGCACCCGATATTTGTCA
ACTGGGAATCCTCCTGGACCTATTATCGGTCCTTTGAAACTAACAGCAATTACCCCAGAAAGTGTCTGCTAA

>p2_ind4899

ATGCAAATAGACCCCGTTGCATATCATTGTTCCAAGTTGAAGTTCGTGCTTGTACCCGAAGAGTCGGGAAAGTTAT
CCCCGACGGAAATAGGATTCCATTTTCAGATTGGGTGCCGCGTACATGACAGAAGACCCCGCTCTGAGTCAGTGA
TGACGCACCAACGCAGATATCGATGTCTCACGTCTACCTTATTACTTGCCTTGATCGTCGGCACCCGATATTTGTCA
ACTGGGAATCCTCCTGGACCTATTATCGGTCCTTTGAAACTAACAGCAATTACCCCAGAAAGTGTCTGCTAA

>p2_ind426

ATGCAAATAGACCCCGTTGCATATCATTGTTCCGAGTTGAAGTTCGTGCTTGTACCCGAAGAGTCGGGAAAGTTAT
CCCCGACGGAAATAGGATTCCATTTTCAGATTGGGTGCCGCGTACATGACAGAAGACCCCGCTCTGAGTCAGTGA
TGACGCACCAACGCAGATATCGATGTCTCACGTCTACCTTATTACTTGCCTTGATCGTCGGCACCCGATATTTGTCA
ACTGGGAATCCTCCTGGACCTATTATCGGTCCTTTGAAACTAACAGCAATTACCCCAGAAAGTGTCTGCTAA

>p2_ind1203

ATGCAAATAGACCCTGTTGCATATCATTGTTCCGAGTTGAAGTTCGTGCTTGTACCCGAAGAGTCGGGAAAGTTAT
CCCCGACGGAAATAGGATTCCATTTTCAGATTGGGTGCCGCGTACATGACAGAAGACCCCGCTCTGAGTCAGTGA
TGGCGCACCAACGCAAATACAGATGTCTCACGTCTACCTTAATACTTGCCTTGATCGTCGGCACCCGATATTTGTCA
ACTGGGAATCCTCCTGGACCTATTATCGGTCCTTTGAAACTAACAGCAATTACCCCAGAAAGTGTCTGCTAA

>p2_ind4690

ATGCAAATAGACCCCGTTGCATATCATTGTTCCGAGTTGAAGTTCGTGCTTGTACCCGAAGAGTCGGGAAAGTTAT
CCCCGACGGAAATAGGATTCCATTTTCAGATTGGGTGCCGCGTACATGACAGAAGACCCCGCTCTGAGTCAGTGA
TGACGCACCAACGCAGATATCGATGTCTCACGTCTACCTTATTACTTGCCTTGATCGTCGGCACCCGATATTTGTCA
ACTGGGAATCCTCCTGGACCTATTATCGGTCCTTTGAAACTAACAGCAATTACCCCAGAAAGTGTCTGCTAA

>p2_ind338

ATGCAAATAGACCCCGTTGCATATCATTGTTCCGAGTTGAAGTTCGTGCTTGTACCCGAAGAGTCGGGAAAGTTAT
CCCCGACGGAAATAGGATTCCATTTTCAGATTGGGTGCCGCGTACATGACAGAAGACCCCGTTCTGAGTCAGTGAT
GACGCACCAACGCAGATATCGATGTCTCACGTCTACCTTATTACTTGCCTTGATCGTCGGCACCCGATATTTGTCAA
CTGGGAATCCTCCTGGACCTATTATCGGTCCTTTGAAACTAACAGCAATTACCCCAGAAAGTGTCTGCTAA

>p2_ind3421

ATGCAAATAGACCCCGTTGCATATCATTGTTCCGAGTTGAAGTTCGTGCTTGTACCCGAAGAGTCGGGAAAGTTAT
CCCCGACGGAAATAGGATTCCATTTTCAGATTGGGTGCCGCGTACATGACAGAAGACCCCGCTCTGTGTCAGTGAT
GACGCACCTACGCAGATATCGATGTCTCAAGTCTACCTTATTACTTGCCTTGATCGTCGGCACCCGATATTTGTCAA
CTGGGAATCCTCCTGGACCTATTATCGGTCCTTTGAAACTAACAGCAATTACCCCAGAAAGTGTCTGCTAA

>p2_ind863

ATGCAAATAGACCCCGTTGCATATCATTGTTCCGAGTTGAAGTTCGTGCTTGTACCCGAAGAGTCGGGAAAGTTAT
CCCCGACGGAAATAGGATTCCATTTTCAGATTGGGTGCCGCGTACATGACAGAAGACCCCGCTCTGAGTCAGTGA
TGACGCACCAACGCAGATATCGATGTCTCACGTCTACCTTATTACTTGCCTTGATCGTCGGCACCCGATATTTGTCA
ACTGGGAATCCTCCTGGACCTATTATCGGTCCTTTGAAACTAACAGCAATTACCCCAGAAAGTGTCTGCTAA

>p2_ind2168

ATGCAAATAGACCCCGTTGCATATCATTGTTCCGAGTTGAAGTTCGTGCTTGTACCCGAAGAGTCGGGAAAGTTAT
CCCCGACGGAAATAGGATTCCATTTTCAGATTGGGTGCCGCGTACATGACAGAAGACCCCGCTCTGAGTCAGTGA
TGACGCACCAACGCAGATATCGATGTCTCACGTCTACCTTATTACTTGCCTTGATCGTCGGCACCCGATATTTGTCA
ACTGGGAATCCTCCTGGACCTATTATCGGTCCTTTGAAACTAACAGCAATTACTCCAGAAAGTGTCTGCTAA

>p2_ind954

ATGCAAATAGACCCCGTTGCATATCATTGTTCCGAGTTGAAGTTCGTGCTTGTACCCGAAGAGTCGGGAAAGTTAT
CCCCGACGGAAATAGGATTCCATTTTCAGATTGGGTGCCGCGTACATGACAGAAGACCCCGCTCTGAGTCAGTGA
TGTCGCACCAACGCAGATATCGATGTCTCACGTCTACCTTATTACTTGCCTTGATCGTCGGCACCCGATATTTGTCA
ACTGGGAATCCTCCTGGACCTATTATCGGTCCTTTGAAACTAACAGCAATTACCCCAGAAAGTGTCTGCTAA

>p2_ind3011

ATGCAAATAGACCCCGTTGCATATCATTGTTCCGAGTTGAAGTTCGTGCTTGTACCCGAAGAGTCGGGAAAGTTAT
CCCCGACGGAAATAGGATTCCATTTTCAGATTGGGTGCCGCGTACATGACAGAAGACCCCGCTCTGAGTCAGTGA

TGACGCACCAACGCAGATATCGATGTCTCACGTCTACCTTATTACTTGCCTTGATCGTCGGCACCCGATATTTGTCA
ACTGGGAATCCTCCTGGACCTATTATCGGTCCTTTGAAACTAACAGCAATTACCCCAGAAAGTGTCTGCTAA

>p2_ind1694

ATGCAAATAGACCCCGTTGCATATCATTGTTCCGAGTTGAAGTTCGTGCTTGTACCCGAAGAGTCGGGAAAGTTAT
CCCCGACGGAAATAGGATTCCATTTTCAGATTGGGTGCCGCGTACATGACAGAAGACCCCGCTCTGAGTCAGTGA
TGACGCACCAACGCAAATATCGATGTCTCACGTCTACCTTATTACTTGCCTTGATCGTCGGCACCCGATATTTGTCA
ACTGGGAATCCTCCTGGACCTATTATCGGTCCTTTGAAACTAACAGCAATTACCCCAGAAAGTGTCTGCTAA

>p2_ind4770

ATGCAAATAGACCCCGTTGCATATCATTGTTCCGAGTTGAAGTTCGTGCTTGTACCCGAAGAGTCGGGAAAGTTAT
CCCCGACGGAAATAGGATTCCATTTTCAGATTGGGTGCCGCGTACATGACAGAAGACCCCGCTCTGAGTCAGTGA
TGACGCACCAACGCAGATATCGATGTCTCACGTCTACCTTATTACTTGCCTTGATCGTCGGCACCCGATATTTGTCA
ACTGGGAATCCTCCTGGACCTATTATCGGTCCTTTGAAACTAACAGCAATTACCCCAGAAAGTGTCTGCTAA

>p2_ind4068

ATGCAAATAGACCCCGTTGCATATCATTGTTCCGAGTTGAAGTTCGTGCTTGTACCCGAAGAGTCGGGAAAGTTAT
CCCCGACGGAAATAGGATTCCATTTTCAGACTGGGCGCCGCGTACATGACAGAAGACCCCGCTCTGAGTCAGTGA
TGACGCACCAACGCAGATATCGATGTCTCACGTCTACCTTATTACTTGCCTTGATCGTCGGCACCCGATATTTGTCA
ACTGGGAATCCTCCTGGACCTATTATCGGTCCTTTGAAACTAACAGCAATTACCCCAGAAAGTGTCTGCTAA

>p2_ind2613

ATGCAAATAGACCCCGTTGCATATCATTGTTCCGAGTTGAAGTTCGTGCTTGTACCCGAAGAGTCGGGAAAGTTAT
CCCCGACGGAAATAGGATTCCATTTTCAGATTGGGTGCCGCGTACATGACAGAAGACCCCGCTCTGAGTCAGTGA
TGGCGCACCAACGCAAATATCGATGTCTCACGTCTACCTTATTACTTGCCTTGATCGTCGGCACCCGATATTTGTCA
ACTGGGAATCCTCCTGGACCTATTATCGGTCCTTTGAAACTAACAGCAATTACCCCAGAAAGTGTCTGCTAA

>p2_ind1729

ATGCAAATAGACCCCGTTGCATATCATTGTTCCGAGTTGAAGTTCGTGCTTGTACCCGAAGAGTCGGGAAAGTTAT
CCCCGACGGAAATAGGATTCCATTTTCAGATTGGGTGCCGCGTACATGACAGAAGACCCCGCTCTGAGTCAGTGA
TGACGCACCAACGCAGATATCGATGTCTCACGTCTACCTTATTACTTGCCTTGATCGTCGGCACCCGATATTTGTCA
ACTGGGAATCCTCCTGGACCTATTATCGGTCCTTTGAAACTAACAGCAATTACCCCAGAAAGTGTCTGCTAA

>p2_ind4079

ATGCAAATAGACCCCGTTGCATATCATTGTTCCGAGTTGAAGTTCGTGCTTGTACCCGAAGAGTCGGGAAAGTTAT
CCCCGACGGAAATAGGATTCCATTTTCAGATTGGGTGCCGCGTACATGACAGAAGACCCCGCTCTGAGTCAGTGA
TGGCGCACCAACGCAAATATCGATGTCTCACGTCTACCTTATTACTTGCCTTGATCGTCGGCACCCGATATTTGTCA
ACTGGGAATCCTCCTGGACCTATTATCGGTCCTTTGAAACTAACAGCAATTACCCCAGAAAGTGTCTGCTAA

>p2_ind4897

ATGCAAATAGACCCCGTTGCATATCATTGTTCCGAGTTGAAGTTCGTGCTTGTACCCGAAGAGTCGGGAAAGTTAT
CCCCGACGGAAATAGGATTCCATTTTCAGATTGGGTGCCGCGTACATGACAGAAGACCCCGCTCTGAGTCAGTGA
TGTCGCACCAACGCAGATATCGATGTCTCACGTCTACCTTATTACTTGCCTTGATCGTCGGCACCCGATATTTGTCA
ACTGGGAATCCTCCTGGACCTATTATCGGTCCTTTGAAACTAACAGCAATTACCCCAGAAAGTGTCTGCTAA

>p2_ind4438

ATGCAAATAGACCCCGTTGCATATCATTGTTCCGAGTTGAAGTTCGTGCTTGTACCCGAAGAGTCGGGAAAGTTAT
CCCCGACGGAAATAGGATTCCATTTTCAGATTGGGTGCCGCGTACATGACAGAAGACCCCGCTCTGAGTCAGTGA
TGACGCACCAACGCAGATATCGATGTCTCACGTCTACCTTATTACTTGCCTTGATCGTCGGCACCCGATATTTGTCA
ACTGGGAATCCTCCTGGACCTATTATCGGTCCTTTGAAACTAACAGCAATTACCCCAGAAAGTGTCTGCTAA

>p2_ind3769

ATGCAAATAGACCCCGTTGCATATCATTGTTCCGAGTTGAAGTTCGTGCTTGTACCCGAAGAGTCGGGAAAGTTAT
CCCCGACGGAAATAGGATTCCATTTTCAGATTGGGTGCCGCGTACATGACAGAAGACCCCGCTCTGAGTCAGTGA
TGACGCACCAACGCAGATATCGATGTCTCACGTCTACCTTATTACTTGCCTTGATCGTCGGCACCCGATATTTGTCA
ACTGGGAATCCTCCTGGACCTATTATCGGTCCTTTGAAACTAACAGCAATTACCCCAGAAAGTGTCTGCTAA

>p2_ind4487

ATGCAAATAGACCCCGTTGCATATCATTGTTCCGAGTTGAAGTTCGTGCTTGTACCCGAAGAGTCGGGAAAGTTAT
CCCCGACGGAAATAGGATTCCATTTTCAGATTGGGTGCCGCGTACATGACAGAAGACCCCGTTCTGAGTCAGTGAT
GACGCACCAACGCAGATATCGATGTCTCACGTCTACCTTATTACTTGCCTTGATCGTCGGCACCCGATATTTGTCAA
CTGGGAATCCTCCTGGACCTATTATCGGTCCTTTGAAACTAACAGGAATTACCCCAGAAAGTGTCTGCTAA

>p2_ind1062

ATGCAAATAGACCCTGTTGCATATCATTGTTCCGAGTTGAAGTTCGTGCTTGTACCCGAAGAGTCGGGAAAGTTAT
CCCCGACGGAAATAGGATTCCATTTTCAGATTGGGTGCCGCGTACATGACAGAAGACCCCGCTCTGAGTCAGTGA
TGGCGCACCAACGCAAATACAGATGTCTCACGTCTACCTTAATACTTGCCTTGATCGTCGGCACCCGATATTTGTCA
ACTGGGAATCCTCCTGGACCTATTATCGGTCCTTTGAAACTAACAGCAATTACCCCAGAAAGTGTCTGCTAA

>p2_ind2607

ATGCAAATAGACCCCGTTGCATATCATTGTTCCGAGTTGAAGTTCGTGCTTGTACCCGAAGAGTCGGGAAAGTTAT
CCCCGACGGAAATAGGATTCCATTTTCAGATTGGGTGCCGCGTACATGACAGAAGACCCCGCTCTGAGTCAGTGA
TGGCGCACCAACGCAAATATCGATGTCTCACGTCTACCTTATTACTTGCCTTGATCGTCGGCACCCGATATTTGTCA
ACTGGGAATCCTCCTGGACCTATTATCGGTCCTTTGAAACTAACAGCAATTACCCCAGAAAGTGTCTGCTAA

>p2_ind3338

ATGCAAATAGACCCCGTTGCATATCATTGTTCCGAGTTGAAGTTCGTGCTTGTACCCGAAGAGTCGGGAAAGTTAT
CCCCGACGGAAATAGGATTCCATTTTCAGATTGGGTGCCGCGTACATGACAGAAGACCCCGCTCTGAGTCAGTGA

TGACGCACCAACGCAGATATCGATGTCTCACGTCTACCTTATTACTTGCCTTGATCGTCGGCACCCGATATTTGTCA
ACTGGGAATCCTCCTGGACCTATTATCGGTCCTTTGAAACTAACAGCAATTACCCCAGAAAGTGTCTGCTAA

>p2_ind2124

ATGCAAATAGACCCCGTTGCATATCATTGTTCCGAGTTGAAGTTCGTGCTTGTACCCGAAGAGTCGGGAAAGTTAT
CCCCGACGGAAATAGGATTCCATTTTCAGATTGGGTGCCGCGTACATGACAGAAGACCCCGCTCTGAGTCAGTGA
TGACGCACCAACGCAGATATCGATGTCTCACGTCTACCTTATTACTTGCCTTGATCGTCGGCACCCGATATTTGTCA
ACTGGGAATCCTCCTGGACCTATTATCGGTCCTTTGAAACTAACGGCAATTACCCCAGAAAGTGTCTGCTAA

>p2_ind4425

ATGCAAATAGACCCCGTTGCATATCATTGTTCCAAGTTGAAGTTCGTGCTTGTACCCGAAGAGTCGGGAAAGTTAT
CCCCGACGGAAATAGGATTCCATTTTCAGATTGGGTGCCGCGTACATGACAGAAGACCCCGCTCTGAGTCAGTGA
TGACGCACCAACGCAGATATCGATGTCTCACGTCTACCTTATTACTTGCCTTGATCGTCGGCACCCGATATTTGTCA
ACTGGGAATCCTCCTGGACCTATTATCGGTCCTTTGAAACTAACAGCAATTACCCCAGAAAGTGTCTGCTAA

>p2_ind1569

ATGCAAATAGACCCCGTTGCATATCATTGTTCCGAGTTGAAGTTCGTGCTTGTACCCGAAGAGTCGGGAAAGTTAT
CCCCGACGGAAATAGGATTCCATTTTCAGATTGGGTGCCGCGTACATGACAGAAGACCCCGCTCTGAGTCAGTGA
TGACGCACCAACGCAGATATCGATGTCTCACGTCTACCTTATTACTTGCCTTGATCGTCGGCACCCGATATTTGTCA
ACTGGGAATCCTCCTGGACCTATTATCGGTCCTTTGAAACTAACAGCAATTACCCCAGAAAGTGTCTGCTAA

>p2_ind2958

ATGCAAATAGACCCCGTTGCATATCATTGTTCCGAGTTGAAGTTCGTGCTTGTACCCGAAGAGTCGGGAAAGTTAT
CCCCGACGGAAATAGGATTCCATTTTCAGATTGGGTGCCGCGTACATGACAGAAGACCCCGCTCTGAGTCAGTGA
TGACGCACCAACGCAGATATCGATGTCTCACGTCTACCTTATTACTTGCCTTGATCGTCGGCACCCGATATTTGTCA
ACTGGGAATCCTCCTGGACCTATTATCGGTCCTTTGAAACTAACAGCAATTACCCCAGAAAGTGTCTGCTAA

>p2_ind3289

ATGCAAATAGACCCCGTTGCATATCATTGTGCCGAGTTGAAGTTCGTGCTTGTACCCGAAGAGTCGGGAAAGTTAT
CCCCGACGGAAATAGGATTTCATTGTCAGATTGGGTGCCGCGTACATGACAGAAGACCCCGCTCTGAGTCAGTGA
TGGCGCACCAACGCAAATATCGATGTCTCACGTCTACCTTATTACTTGCCTTGATCGTCGGCACCCGATATTTGTCA
ACTGGGAATCCTCCTGGACCTATTATCGGTCCTTTGAAACTAACAGCAATTACCCCAGAAAGTGTCTGCTAA

>p2_ind2518

ATGCAAATAGACCCCGTTGCATATCATTGTTCCGAGTTGAAGTTCGTGCTTGTACCCGAAGAGTCGGGAAAGTTAT
CCCCGACGGAAATAGGATTCCATTTTCAGATTGGGTGCCGCGTACATGACAGAAGACCCCGCTCTGAGTCAGTGA
TGGCGCACCAACGCAAATATCGATGTCTCACGTCTACCTTATTACTTGCCTTGATCGTCGGCACCCGATATTTGTCA
ACTGGGAATCCTCCTGGACCTATTATCGGTCCTTTGAAACTAACAGCAATTACCCCAGAAAGTGTCTGCTAA

>p2_ind195

ATGCAAATAGACCCCGTTGCATATCATTGTTCCGAGTTGAAGTTCGTGCTTGTACCCGAAGAGTCGGGAAAGTTAT
CCCCGACGGAAATAGGATTCCATTTTCAGATTGGGTGCCGCGTACATGACAGAAGACCCCGCTCTGAGTCAGTGA
TGACGCACCAACGCAGATATCGATGTCTCACGTCTACCTTATTACTTGCCTTGATCGTCGGCACGCGATATTTGTCA
ACTGGGAATCCTCCTGGACCTATTATCGGTCCTTTGAAACTAACAGCAATTACCCCAGAAAGTGTCTGCTAA

>p2_ind2269

ATGCAAATAGACCCCGTTGCATATCATTGTTCCGAGTTGAAGTTCGTGCTTGTACCCGAAGAGTCGGGAAAGTTAT
CCCCGACGGAAATAGGATTCCATTTTCAGATTGGGTGCCGCGTACATGACAGAAGACCCCGCTCTGAGTCAGTGA
TGACGCACCAACGCAGATATCGATGTCTCACGTCTACCTTATTACTTGCCTTGATCGTCGGCACCCGATATTTGTCA
ACTGGGAATCCTCCTGGACCTATTATCGGTCCTTTGAAACTAACAGGAATTACCCCAGAAAGTGTCTGCTAA

>p2_ind4339

ATGCAAATAGACCCCGTTGCATATCATTGTTCCGAGTTGAAGTTCGTGCTTGTACCCGAAGAGTCGGGAAAGTTAT
CCCCGACGGAAATAGGATTCCATTTTCAGATTGGGTGCCGCGTACATGACAGAAGACCCCGCTCTGAGTCAGTTAT
GACGCACCAACGCAGATATCGATGTCTCACGTCTACCTTATTACTTGCCTTGATCGTCGGCACCCGATATTTGTCAA
CTGGGAATCCTCCTGGACCTATTATCGGTCCTTTGAAACTAACAGCAATTACCCCATAAAGTGTCTGCTAA

>p2_ind1856

ATGCAAATAGACCCCGTTGCATATCATTGTTCCGAGTTGAAGTTCGTGCTTGTACCCGAAGAGTCGGGAAAGTTAT
CCCCGACGGAAATAGGATTCCATTTTCAGATTGGGTGCCGCGTACATGACAGAAGACCCCGCTCTGAGTCAGTGA
TGACGCACCAACGCAGATATCGATGTCTCACGTCTACCTTATTACTTGCCTTGATCGTCGGCACCCGATATTTGTCA
ACTGGGAATCCTCCTGGACCTATTATCGGTCCTTTGAAACTAACAGCAATTACCCCAGAAAGTGTCTGCTAA

>p2_ind3334

ATGCAAATAGACCCCGTTGCATATCATTGTTCCGAGTTGAAGTTCGTGCTTGTACCCGAAGAGTCGGGAAAGTTAT
CCCCGACGGAAATAGGATTCCATTTTCAGATTGGGCGCCGCGTACATGACAGAAGACCCCGCTCTGAGTCAGTGA
TGACGCACCAACGCAGATATCGATGTCTCACGTCTACCTTATTACTTGCCTTGATCGTCGGCACCCGATATTTGTCA
ACTGGGAATCCTCCTGGACCTATTATCGGTCCTTTGAAACTAACAGCAATTACCCCAGAAAGTGTCTGCTAA

>p2_ind4727

ATGCAAATAGACCCCGTTGCATATCATTGTTCCGAGTTGAAGTTCGTGCTTGTACCCGAAGAGTCGGGAAAGTTAT
CCCCGACGGAAATAGGATTCCATTTTCAGATTGGGTGCCGCGTACATGACAGAAGACCCCGCTCTGAGTCAGTGA
TGACGCACCAACGCAGATATCGATGTCTCACGTCTACCTTATTACTTGCCTTGATCGTCGGCACCCGATATTTGTCA
ACTGGGAATCCTCCTGGACCTATTATCGGTCCTTTGAAACTAACAGCAATTACCCCAGAAAGTGTCTGCTAA

>p2_ind2624

ATGCAAATAGACCCCGTTGCATATCATTGTTCCGAGTTGAAGTTCGTGCTTGTACCCGAAGAGTCGGGAAAGTTAT
CCCCGACGGAAATAGGATTCCATTTTCAGATTGGGTGCCGCGTACATGACAGAAGACCCCGCTCTGAGTCAGTGA

TGACGCACCAACGCAGATATCGATGTCTCACGTCTACCTTATTACTTGCCTTGATCGTCGGCACCCGATATTTGTCA
ACTGGGAATCCTCCTGGACCTATTATCGGTCCTTTGAAACTAACGGCAATTACCCCAGAAAGTGTCTGCTAA

>p2_ind1250

ATGCAAATAGACCCCGTTGCATATCATTGATCCGAGTTGAAGTTCGTGCTTGTACCCGAAGAGTCGGGAAAGTTAT
CCCCGACGGAAATAGGATTCCATTTTCAGATTGGGTGCCGCGTACATGACAGAAGACCCCGTTCTGAGTCAGTGAT
GACGCACCAACGCAGATATCGATGTCTCACGTCTACCTTATTACTTGCCTTGATCGTCGGCACCCGATTTTTGTCAA
CTGGGAATCCTCCTGGACCTATTATCGGTCCTTTGAAACTAACAGCAATTACCCCAGAAAGTGTCTGCTAA

>p2_ind3798

ATGCAAATAGACCCCGTTGCATATCATTGTTCCGAGTTGAAGTTCGTGCTTGTACCCGAAGAGTCGGGAAAGTTAT
CCCCGACGGAAATAGGATTCCATTTTCAGATTGGGTGCCGCGTACATGACAGAAGACCCCGCTCTGAGTCAGTGA
TGACGCACCAACGCAGATATCGATGTCTCACGTCTACCTTATTACTTGGCTTGATCGTCGGCACCCGATATTTGTCA
ACTGGGAATCCTCCTGGACCTATTATCGGTCCTTTGAAACTAACAGCAATTACCCCAGAAAGTGTCTGCTAA

>p2_ind2960

ATGCAAATAGACCCCGTTGCATATCATTGTTCCGAGTTGAAGTTCGTGCTTGTACCCGAAGAGTCGGGAAAGTTAT
CCCCGACGGAAATAGGATTCCATTTTCAGATTGGGTGCCGCGTACATGACAGAAGACCCCGCTCTGAGTCAGTGA
TGACGCACCAACGCAGATATCGATGTCTCACGTCTACCTTATTACTTGCCTTGATCGTCGGCACCCGATATTTGTCA
ACTGGGAATCCTCCTGGACCTATTATCGGTCCTTTGAAACTAACAGCAATTACTCCAGAAAGTGTCTGCTAA

>p2_ind638

ATGCAAATAGACCCCGTTGCATATCATTGTTCCGAGTTGAAGTGCGTGCTTGTACCCGAAGAGTCGGGAAAGTTAT
CCCCGACGGAAATAGGATTCCATTTTCAGATTGGGTGCCGCGTACATGACAGAAGACCCCGCTCTGAGTCAGTGA
TGACGCACCAACGCAAATATCGATGTCTCACGTCTACCTTATTACTTGCCTTGATCGTCGGCACCCGATATTTGTCA
ACTGGGAATCCTCCTGGACCTATTATCGGTCCTTTGAAACTAACAGCAATTACCCCAGAAAGTGTCTGCTAA

>p2_ind2854

ATGCAAATAGACCCCGTTGCATATCATTGTTCCGAGTTGAAGTTCGTGCTTGTACCCGAAGAGTCGGGAAAGTTAT
CCCCGACGGAAATAGGATTCCATTTTCAGATTGGGTGCCGCGTACATGACAGAAGACCCCGCTCTGAGTCAGTGA
TGACGCACCAACGCAGATATCGATGTCTCACGTCTACCTTATTACTTGCCTTGATCGTCGGCACCCGATATTTGTCA
ACTGGGAATCCTCCTGGACCTATTATCGGTCCTTTGAAACTAACAGCAATTACCCCAGAAAGTGTCTGCTAA

>p2_ind245

ATGCAAATAGACCCCGTTGCATATCATTGTTCCGAGTTGAAGTTCGTGCTTGTACCCGAAGAGTCGGGAAAGTTAT
CCCCGACGGAAATAGGATTCCATTTTCAGATTGGGTGCCGCGTACATGACAGAAGACCCCGCTCTGAGTCAGTGA
TGGCGCACCAACGCAGATATCGATGTCTCACGTCTACCTTATTACTTGCCTTGATCGTCGGCACCCGATATTTGTCA
ACTGGGAATCCTCCTGGACCTATTATCGGTCCTTTGAAACTAACAGCAATTACCCCAGAAAGTGTCTGCTAA

>p2_ind4724

ATGCAAATAGACCCCGTTGCATATCATTGTTCCGAGTTGAAGTTCGTGCTTGTACCCGAAGAGTCGGGAAAGTTAT
CCCCGACGGAAATAGGATTCCATTTTCAGATTGGGTGCCGCGTACATGACAGAAGACCCCGCTCTGAGTCAGTGA
TGACGCACCAACGCAGATATCGATGTCTCACGTCTACCTTATTACTTGCCTTGATCGTCGGCACCCGATATTTGTCA
ACTGGGAATCCTCCTGGACCTATTATCGGTCCTTTGAAACTAACAGCAATTACCCCAGAAAGTGTCTGCTAA

>p2_ind4943

ATGCAAATAGACCCCGTTGCATATCATTGTTCCGAGTTGAAGTTCGTGCTTGTACCCGAAGAGTCGGGAAAGTTAT
CCCCGACGGAAATAGGATTCCATTTTCAGATTGGGTGCCGCGTACATGACAGAAGACCCCGCTCTGAGTCAGTGA
TGACGCACCAACGCAGATATCGATGTCTCACGTCTACCTTATTACTTGCCTTGATCGTCGGCACCCGATATTTGTCA
ACTGGGAATCCTCCTGGACCTATTATCGGTCCTTTGAAACTAACAGCAATTACCCCAGAAAGTGTCTGCTAA

>p2_ind1923

ATGCAAATAGACCCTGTTGCATATCATTGTTCCGAGTTGAAGTTCGTGCTTGTACCCGAAGAGTCGGGAAAGTTAT
CCCCGACGGAAATAGGATTCCATTTTCAGATTGGGTGCCGCGTACATGACAGCAGACCCCGCTCTGAGTCAGTGAT
GGCGCACCAACGCAAATATCGATGTCTCACGTCTACCTTATTACTTGCCTTGATCGTCGGCACCCGATATTTGTCAA
CTGGGAATCCTCCTGGACCTATTATCGGTCCTTTGAAACTAACAGCAATTACCCCAGAAAGTGTCTGCTAA

>p2_ind4206

ATGCAAATAGACCCCGTTGCATATCATTGTTCCGAGTTGAAGTTCGTGCTTGTACCCGAAGAGTCGGGAAAGTTAT
CCCCGACGGAAATAGGATTCCATTTTCAGATTGGGTGCCGCGTACATGACAGAAGACCCCGCTCTGAGTCAGTGA
TGACGCACCAACGCAGATATCGATGTCTCACGTCTACCTTATTACTTGCCTTGATCGTCGGCACCCGATATTTGTCA
ACTGGGAATCCTCCTGGACCTATTATCGGTCCTTTGAAACTAACAGCAATTACCCCAGAAAGTGTCTGCTAA

>p2_ind203

ATGCAAATAGACCCCGTTGCATATCATTGTTCCGAGTTGAAGTTCGTGCTTGTACCCGAAGAGTCGGGAAAGTTAT
CCCCGACGGAAATAGGATTCCATTTTCAGATTGGGTGCCGCGTACATGACAGAAGACCCCGCTCTGAGTCAGTGA
TGACGCACCAACGCAGATATCGATGTCTCACGTCTACCTTATTACTTGCCTTGATCGTCGGCACCCGATATTTGTCA
ACTGGGAATCCTCCTGGACCTATTATCGGTCCTTTGAAACTAACAGCAATTACCCCAGAAAGTGTCTGCTAA

>p2_ind3855

ATGCAAATAGACCCCGTTGCATATCATTGTTCCGAGTTGAAGTTCGTGCTTGTACCCGAAGAGTCGGGAAAGTTAT
CCCCGACGGAAATAGGATTCCATTTTCAGATTGGGTGCCGCGTACATGACAGAAGACCCCGCTCTGAGTCAGTGA
TGACGCACCAACGCAAATATCGATGTCTCACGTCTACCTTATTACTTGCCTTGATCGTCGGCACCCGATATTTGTCA
ACTGGGAATCCTCCTGGACCTATTATCGGTCCTTTGAAACTAACAGCAATTACCCCAGAAAGTGTCTGCTAA

>p2_ind4892

ATGCAAATAGACCCCGTTGCATATCATTGTTCCGAGTTGAAGTTCGTGCTTGTACCCGAAGAGTCGGGAAAGTTAT
CCCCGACGGAAATAGGATTCCATTTTCAGACTGGGCGCCGCGTACATGACAGAAGACCCCGCTCTGAGTCAGTGA

TGACGCACCAACGCAGATATCGATGTCTCACGTCTACCTAATTACTTGCCTTGATCGTCGGCACCCGATATTTGTCA
ACTGGGAATCCTCCTGGACCTATTATCGGTCCTTTGAAACTAACAGCAATTACCCCAGAAAGTGTCTGCTAA

>p2_ind436

ATGCAAATAGACCCCGTTGCATATCATTGTTCCGAGTTGAAGTTCGTGCTTGTATCCGAAGAGTCGGGAAAGTTAT
CCCCGACGGAAATAGGATTCCATTTTCAGATTGGGTGCCGCGTACATGACAGAAGACCCCGCTCTGAGTCAGTGA
TGACGCACCAACGCAGATATCGATGTCTCACGTCTACCTTATTACTTGCCTTGATCGTCGGCACCCGATATTTGTCA
ACTGGGAATCCTCCTGGACCTATTATCGGTCCTTTGAAACTAACAGCAATTACCCCAGAAACTGTCTGCTAA

>p2_ind4925

ATGCAAATAGACCCCGTTGCATATCATTGTTCCGAGTTGAAGTTCGTGCTTGTACCCGAAGAGTCGGGAAAGTTAT
CCCCGACGGAAATAGGATTCCATTTTCAGATTGGGTGCCGCGTACATGACAGAAGACCCCGCTCTGAGTCAGTGA
TGGCGCACCAACGCAAATATCGATGTCTCACGTCTACCTTATTACTTGCCTTGATCGTCGGCACCCGATATTTGTCA
ACTGGGAATCCTCCTGGACCTATTATCGGTCCTTTGAAACTAACAGCAATTACCCCAGAAAGTGTCTGCTAA

>p2_ind2775

ATGCAAATAGACCCCGTTGCATATCATTGTTCCGAGTTGAAGTTCGTGCTTGTACCCGAAGAGTCGGGAAAGTTAT
CCCCGACGGAAATAGGATTCCATTTTCAGATTGGGTGCCGCGTACATGACAGAAGACCCCGCTCTGAGTCAGTGA
TGACGCACCTACGCAGATATCGATGTCTCACGTCTACCTTATTACTTGCCTTGATCGTCGGCACCCGATATTTGTCA
ACTGGGAATCCTCCTGGACCTATTATCGGTCCTTTGAAACTAACAGCAATTACCCCAGAAAGTGTCTGCTAA

>p2_ind3069

ATGCAAATAGACCCCGTTGCATATCATTGATCCGAGTTGAAGTTCGTGCTTGTACCCGAAGAGTCGGGAAAGTTAT
CCCCGACGGAAATAGGATTCCATTTTCAGATTGGGTGCCGCGTACATGACAGAAGACCCCGTTCTGAGTCAGTGAT
GACGCACCAACGCAGATATCGATGTCTCACGTCTACCTTATTACTTGCCTTGATCGTCGGCACCCGATATTTGTCAA
CTGGGAATCCTCCTGGACCTATTATCGGTCCTTTGAAACTAACAGCAATTACCCCAGAAAGTGTCTGCTAA

>p2_ind2445

ATGCAAATAGACCCCGTTGCATATCATTGTTCCGAGTTGAAGTTCGTGCTTGTACCCGAAGAGTCGGGAAAGTTAT
CCCCGACGGAAATAGGATTCCATTTTCAGATTGGGTGCCGCGTACATGACAGAAGACCCCGCTCTGAGTCAGTGA
TGACGCACCAACGCAGATATCGATGTCTCACGTCTACCTTATTACTTGCCTTGATCGTCGGCACCCGATATTTGTCA
ACTGGGAATCCTCCTGGACCTATTATCGGTCCTTTGAAACTAACAGCAATTACCCCAGAAAGTGTCTGCTAA

>p2_ind2709

ATGCAAATAGACCCCGTTGCATATCATTGTTCCGAGTTGAAGTTCGTGCTTGTACCCGAAGAGTCGGGAAAGTTAT
CCCCGACGGAAATAGGATTCCATTTTCAGATTGGGTGCCGCGTACATGACAGAAGACCCCGCTCTGAGTCAGTGA
TGACGCACCAACGCAGATATCGATGTCTCACGTCTACCTTATTACTTGCCTTGATCGTCGGCACCCGATATTTGTCA
ACTGGGAATCCTCCTGGACCTATTATCGGTCCTTTGAAACTAACAGCAATTACCCCAGAAAGTGTCTGCTAA

>p2_ind2755

ATGCAAATAGACCCCGTTGCATATCATTGTTCCGAGTTGAAGTTCGTGCTTGTACCCGAAGAGTCGGGAAAGTTAT
CCCCGACGGAAATAGGATTCCATTTTCAGATTGGGTGCCGCGTACATGACAGAAGACCCCGCTCTGAGTCAGTGA
TGACGCACCAACGCAGATATCGATGTCTCACGTCTACCTTATTACTTGCCTTGATCGTCGGCACCCGATATTTGTCA
ACTGGGAATCCTCCTGGACCTATTATCGGTCCTTTGAAACTAACAGCAATTACACCAGAAAGTGTCTGCTAA

>p2_ind952

ATGCAAATAGACCCCGTTGCATATCATTGTTCCGAGTTGAGGTTCGTGCTTGTACCCGAAGAGTCGGGAAAGTTAT
CCCCGACGGAAATAGGATTCCATTTTCAGATTGGGTGCCGCGTACATGACAGAAGACCCCGCTCTGAGTCAGTTAT
GACGCACCAACGCAGATATCGATGTCTCACGTCTACCTTATTACTTGCCTTGACCGTCGGCACCCGATATTTGTCAA
CTGGGAATCCTCCTGGACCTATTATCGGTCCTTTGAAACTAACAGCAATTACCCCAGAAAGTGTCTGCTAA

>p2_ind3054

ATGCAAATAGACCCCGTTGCATATCATTGTTCCGAGTTGAAGTTCGTGCTTGTACCCGAAGAGTCGGGAAAGTTAT
CCCCGACGGAAATAGGATTCCATTTTCAGATTGGGCGCCGCGTACATGACAGAAGACCCCGCTCTGAGTCAGTGA
TGACGCACCAACGCAGATATCGATGTCTCACGTCTACCTTATTACTTGCCTTGATCGTCGGCACCCGATATTTGTCA
ACTGGGAATCCTCCTGGACCTATTATCGGTCCTTTGAAACTAACAGCAATTACCCCAGAAAGTGTCTGCTAA

>p2_ind3790

ATGCAAATAGACCCCGTTGCATATCATTGTTCCGAGTTGAAGTTCGTGCTTGTACCCGAAGAGTCGGGAAAGTTAT
CCCCGACGGAAATAGGATTCCATTTTCAGATTGGGTGCCGCGTACATGACAGAAGACCCCGCTCTGAGTCAGTGA
TGGCGCACCAACGCAAATATCGATGTCTCACGTCTACCTTATTACTTGCCTTGATCGTCGGCACCCGATATTTGTCA
ACTGGGAATCCTCCTGGACCTATTATCGGTCCTTTGAAACTAACAGCAATTACCCCAGAAAGTGTCTGCTAA

>p2_ind1276

ATGCAAATAGACCCCGTTGCATATCATTGTTCCGAGTTGAAGTTCGTGCTTGTACCCGAAGAGTCGGGAAAGTTAT
CCCCGACGGAAATAGGATTCCATTTTCAGATTGGGTGCCGCGTACATGACAGAAGACCCCGCTCTGAGTCAGTGA
TGACGCACCAACGCAGATATCGATGTCTCACGTCTACCTTATTACTTGCCTTGATCGTCGGCACCCGATATTTGTCA
ACTGGGAATCCTCCTGGACCTATTATCGGTCCTTTGAAACTAACAGGAATTACCCCAGAAAGTGTCTGCTAA

>p2_ind1386

ATGCAAATAGACCCCGTTGCATATCATTGTTCCGAGTTGAAGTTCGTGCTTGTACCCGAAGAGTCGGGAAAGTTAT
CCCCGACGGAAATAGGATTCCATTTTCAGATTGGGTGCCGCGTACATGACAGAAGACCCCGCTCTGAGTCAGTGA
TGACGCACCAACGCAGATATCGATGTCTCACGTCTACCTTATTACTTGCCTTGATCGTCGGCACCCGATATTTGTCA
ACTGGGAATCCTCCTGGACCTATTATCGGTCCTTTGAAACTAACAGCAATTACCCCAGAAAGTGTCTGCTAA

>p2_ind1213

ATGCAAATAGACCCCGTTGCATATCATTGTTCCGAGTTGAAGTTCGTGCTTGTACCCGAAGAGTCGGGAAAGTTAT
CCCCGACGGAAATAGGATTACATTTTCAGATTGGGCGCCGCGTACATGACAGAAGACCCCGCTCTGAGTCAGTGA

TGACGCACCAACGCAGATATCGATGTCTCACGTCTACCTTATTACTTGCCTTGATCGTCGGCACCCGATATTTGTCA
ACTGGGAATCCTCCTGGACCTATTATCGGTCCTTTGAAACTAACAGCAATTACCCCAGAAAGTGTCTGCTAA

>p2_ind1489

ATGCAAATAGACCCCGTTGCATATCATTGATCCGAGTTGAAGTTCGTGCTTGTACCCGAAGAGTCGGGAAAGTTAT
CCCCGACGGAAATAGGATTCCATTTTCAGATTGGGTGCCGCGTACATGACAGAAGACCCCGTTCTGAGTCAGTGAT
GACGCACCAACGCAGATATCGATGTCTCACGTCTACCTTATTACTTGCCTTGATCGTCGGCACCCGATATTTGTCAA
CTGGGAATCCTCCTGGACCTATTATCGGTCCTTTGAAACTAACAGCAATTACCCCAGAAAGTGTCTGCTAA

>p2_ind4893

ATGCAAATAGACCCTGTTGCATATCATTGTTCCGAGTTGAAGTTCGTGCTTGTACCCGAAGAGTCGGGAAAGTTAT
CCCCGACGGAAATAGGATTCCATTTTCAGATTGGGTGCCGCGTACATGACAGAAGACCCCGCTCTGAGTCAGTGA
TGGCGCACCAACGCAAATACAGATGTCTCACGTCTACCTTAATACTTGCCTTGATCGTCGGCACCCGATATTTGTCA
ACTGGGAATCCTCCTGGACCTATTATCGGTCCTTTGAAACTAACAGCAATTACCCCAGAAAGTGTCTGCTAA

>p2_ind1728

ATGCAAATAGACCCCGTTGCATATCATTGTTCCGAGTTGAAGTTCGTGCTTGTACCCGAAGAGTCGGGAAAGTTAT
CCCCGACGGAAATAGGATTCCATTTTCAGATTGGGTGCCGCGTACATGACAGAAGACCCCGCTCTGAGTCAGTGA
TGACGCACCAACGCAGATATCGATGTCTCACGTCTACCTTATTACTTGCCTTGATCGTCGGCACCCGATATTTGTCA
ACTGGGAATCCTCCTGGACCTATTATCGGTCCTTTGAAACTAACAGCAATTACCCCAGAAAGTGTCTGCTAA

>p2_ind984

ATGCAAATAGACCCCGTTGCATATCATTGTTCCGAGTTGAAGTTCGTGCTTGTACCCGAAGAGTCGGGAAAGTTAT
CCCCGACGGAAATAGGATTCCATTTTCAGATTGGGTGCCGCGTACATGACAGAAGACCCCGCTCTGAGTCAGTGA
TGACGCACCAACGCAGATATCGATGTCTCACGTCTACCTTATTACTTGCCTTGATCGTCGGCACCCGATATTTGTCA
ACTGGGAATCCTCCTGGACCTATTATCGGTCCTTTGAAACTAACAGCAATTACCCCAGAAAGTGTCTGCTAA

>p2_ind4433

ATGCAAATAGACCCCGTTGCATATCATTGTTCCGAGTTGAAGTTCGTGCTTGTACCCGAAGAGTCGGGAAAGTTAT
CCCCGACGGAAATAGGATTCCATTTTCAGATTGGGTGCCGCGTACATGACAGAAGACCCCGCTCTGAGTCAGTGA
TGACGCACCAACGCAGACATCGATGTCTCACGTCTACCTTATTACTTGCCTTGATCGTCGGCACCCGATATTTGTCA
ACTGGGAATCCTCCTGGACCTATTATCGGTCCTTTGAAACTAACAGCAATTACCCCAGAAAGTGTCTGCTAA

>p2_ind2885

ATGCAAATAGACCCCGTTGCATATCATTGTTCCGAGTTGAAGTGCGTGCTTGTACCCGAAGAGTCGGGAAAGTTAT
CCCCGACGGAAATAGGATTCCATTTTCAGATTGGGTGCCGCGTACATGACAGAAGACCCCGCTCTGAGTCAGTGA
TGGCGCACCAACGCAAATATCGATGTCTCACGTCTACCTTATTACTTGCCTTGATCGTCGGCACCCGATATTTGTCA
ACTGGGAATCCTCCTGGACCTATTATCGGTCCTTTGAAACTAACAGCAATTACCCCAGAAAGTGTCTGCTAA

>p2_ind1809

ATGCAAATAGACCCCGTTGCATATCATTGTTCCGAGTTGAAGTTCGTGCTTGTACCCGAAGAGTCGGGAAAGTTAT
CCCCGACGGAAATAGGATTCCATTTTCAGATTGGGTGCCGCGTACATGACAGAAGACCCCGCTCTGAGTCAGTGA
TGACGCACCAACGCAGATATCGATGTCTCACGTCTACCTTATTACTTGCCTTGATCGTCGGCACCCGATATTTGTCA
ACTGGGAATCCTCCTGGACCTATTATCGGTCCTTTGAAACTAACGGCAATTACCCCAGAAAGTGTCTGCTAA

>p2_ind2813

ATGCAAATAGACCCCGTTGCATATCATTGTTCCGAGTTGAAGTTCGTGCTTGTACCCGAAGAGTCGGGAAAGTTAT
CCCCGACGGAAATAGGATTCCATTTTCAGATTGGGTGCCGCGTACATGACAGAAGACCCCGCTCTGAGTCAGTGA
TGACGCACCAACGCAGATATCGATGTCTCACGTCTACCTTATTACTTGCCTTGATCGTCGGCACCCGATATTTGTCA
ACTGGGAATCCTCCTGGACCTATTATCGGTCCTTTGAAACTAACAGCAATTACCCCAGAAAGTGTCTGCTAA

>p2_ind2623

ATGCAAATAGACCCCGTTGCATATCATTGTTCCGAGTTGAAGTTCGTGCTTGTATCCGAAGAGTCGGGAAAGTTAT
CCCCGACGGAAATAGGATTCCATTTTCAGATTGGGTGCCGCGTACATGACAGAAGACCCCGCTCTGAGTCAGTGA
TGACGCACCAACGCAGATATCGATGTCTCACGTCTACCTTATTACTTGCCTTGATCGTCGGCACCCGATATTTGTCA
ACTGGGAATCCTCCTGGACCTATTATCGGTCCTTTGAAACTAACAGCAATTACCCCAGAAACTGTCTGCTAA

>p2_ind2364

ATGCAAATAGACCCCGTTGCATATCATTGTTCCGAGTTGAAGTTCGTGCTTGTACCCGAAGAGTCGGGAAAGTTAT
CCCCGACGGAAATAGGATTCCATTTTCAGATTGGGTGCCGCGTACATGACAGAAGACCCCGCTCTGAGTCAGTGA
TGACGCACCAACGCAGATATCGATGTCTCACGTCTACCTTATTACTTGCCTTGATCGTCGGCACCCGATATTTGTCA
ACTGGGAATCCTCCTGGACCTATTATCGGTCCTTTGAAACTAACAGCAATTACCCCAGAAAGTGTCTGCTAA

>p2_ind1051

ATGCAAATAGACCCCGTTGCATATCATTGTTCCGAGTTGAAGTTCGTGCTTGTACCCGAAGAGTCGGGAAAGTTAT
CCCCGACGGAAATAGGATTCCATTTTCAGATTGGGCGCCGCGTACATGACAGAAGACCCCGCTCTGAGTCAGTGA
TGACGCACCAACGCAGATATCGATGTCTCACGTCTACCTTATTACTTGCCTTGATCGTCGGCACCCGATATTTGTCA
ACTGGGAATCCTCCTGGACCTATTATCGGTCCTTTGAAACTAACAGCAATTACCCCAGAAAGTGTCTGCTAA

>p2_ind4128

ATGCAAATAGACCCCGTTGCATATCATTGTTCCGAGTTGAAGTTCGTGCTTGTACCCGAAGAGTCGGGAAAGTTAT
CCCCGACGGAAATAGGATTCCATTTTCAGATTGGGTGCCGCGTACATGACAGAAGACCCCGCTCTGAGTCAGTGA
TGACGCACCAACGCAGATATCGATGTCTCACGTCTACCTTATTACTTGCCTTGATCGTCGGCACCCGATATTTGTCA
ACTGGGAATCCTCCTGGACCTATTATCGGTCCTTTGAAACTAACAGCAATTACCCCAGAAAGTGTCTGCTAA

>p2_ind3420

ATGCAAATAGACCCCGTTGCATATCATTGTTCCGAGTTGAAGTTCGTGCTTGTACCCGAAGAGTCGGGAAAGTTAT
CCCCGACGGAAATAGGATTACATTTTCAGATTGGGCGCCGCGTACATGACAGAAGACCCCGCTCTGAGTCAGTGA

TGACGCACCAACGCAGATATCGATGTCTCACGTCTACCTTATTACTTGCCTTGATCGTCGGCACCCGATATTTGTCA
ACTGGGAATCCTCCTGGACCTATTATCGGTCCTTTGAAACTAACAGCAATTACCCCAGAAAGTGTCTGCTAA

>p2_ind1639

ATGCAAATAGACCCCGTTGCATATCATTGTTCCGAGTTGAAGTTCGTGCTTGTACCCGAAGAGTCGGGAAAGTTAT
CCCCGACGGAAATAGGATTCCATTTTCAGATTGGGTGCCGCGTACATGACAGAAGACCCCGCTCTGAGTCAGTGA
TGACGCACCAACGCAGATATCGATGTCTCACGTCTACCTTATTACTTGCCTTGATCGTCGGCACCCGATATTTGTCA
ACTGGGAATCCTCCTGGACCTATTATCGGTCCTTTGAAACTAACAGCAATTACCCCAGAAAGTGTCTGCTAA

>p2_ind1175

ATGCAAATAGACCCCGTTGCATATCATTGTTCCGAGTTGAAGTTCGTGCTTGTACCCGAAGAGTCGGGAAAGTTAT
CCCCGACGGAAATAGGATTCCATTTTCAGATTGGGTGCCGCGTACATGACAGAAGACCCCGCTCTGAGTCAGTGA
TGACGCACCAACGCAGATATCGATGTCTCACGTCTACCTTATTACTTGCCTTGATCGTCGGCACCCGATATTTGTCA
ACTGGGAATCCTCCTGGACCTATTATCGGTCCTTTGAAACTAACAGCAATTACCCCAGAAAGTGTCTGCTAA

>p2_ind4252

ATGCAAATAGACCCCGTTGCATATCATTGTTCCGAGTTGAAGTTCGTGCTTGTACCCGAAGAGTCGGGAAAGTTAT
CCCCGACGGAAATAGGATTCCATTTTCAGATTGGGTGCCGCGTACATGACAGAAGACCCCGCTCTGAGTCAGTGA
TGACGCACCAACGCAGATATCGATGTCTCACGTCTACCTTATTACTTGCCTTGATCGTCGGCACCCGATATTTGTCA
ACTGGGAATCCTCCTGGACCTATTATCGGTCCTTTGAAACTAACAGCAATTACCCCAGAAAGTGTCTGCTAA

>p2_ind1620

ATGCAAATAGACCCCGTTGCATATCATTGTTCCGAGTTGAAGTTCGTGCTTGTACCCGAAGAGTCGGGAAAGTTAT
CCCCGACGGAAATAGGATTCCATTTTCAGATTGGGTGCCGCGTACATGACAGAAGACCCCGTTCTGAGTCAGTGAT
GACGCACCAACGCAGATATCGATGTCTCACGTCTACCTTATTACTTGCCTTGATCGTCGGCACCCGATATTTGTCAA
CTGGGAATCCTCCTGGACCTATTATCGGTCCTTTGAAACTAACAGGAATTACCCCAGAAAGTGTCTGCTAA

>p2_ind4931

ATGCAAATAGACCCCGTTGCATATCATTGTTCCGAGTTGAGGTTCGTGCTTGTACCCGAAGAGTCGGGAAAGTTAT
CCCCGACGGAAATAGGATTCCATTTTCAGATTGGGTGCCGCGTACATGACAGAAGACCCCGCTCTGAGTCAGTTAT
GACGCACCAACGCAGATATCGATGTCTCACGTCTACCTTATTACTTGCCTTGACCGTCGGCACCCGATATTTGTCAA
CTGGGAATCCTCCTGGACCTATTATCGGTCCTTTGAAACTAACAGCAATTACCCCAGAAAGTGTCTGCTAA

>p2_ind1669

ATGCAAATAGACCCCGTTGCATATCATTGTTCCGAGTTGAAGTTCGTGCTTGTACCCGAAGAGTCGGGAAAGTTAT
CCCCGACGGAAATAGGATTCCATTTTCAGATTGGGCGCCGCGTACATGACAGAAGACCCCGCTCTGAGTCTGTGAT
GACGCACCAACGCAGATATCGATGTCTCACGTCTACCTTATTACTTGCCTTGATCGTCGGCACCCGATATTTGTCAA
CTGGGAATCCTCCTGGACCTATTATCGGTCCTTTGAAACTAACAGCAATTACCCCAGAAAGTGTCTGCTAA

>p2_ind4746

ATGCAAATAGACCCCGTTGCATATCATTGATCCGAGTTGAAGTTCGTGCTTGTACCCGAAGAGTCGGGAAAGTTAT
CCCCGACGGAAATAGGATTCCATTTTCAGATTGGGTGCCGCGTACATGACAGAAGACCCCGTTCTGAGTCAGTGAT
GACGCACCAACGCAGATATCGATGTCTCACGTCTACCTTATTACTTGCCTTGATCGTCGGCACCCGATATTTGTCAA
CTGGGAATCCTCCTGGACCTATTATCGGTCCTTTGAAACTAACAGCAATTACCCCAGAAAGTGTCTGCTAA

>p2_ind1007

ATGCAAATAGACCCCGTTGCATATCATTGATCCGAGTTGAAGTTCGTGCTTGTACCCGAAGAGTCGGGAAAGTTAT
CCCCGACGGAAATAGGATTCCATTTTCAGATTGGGTGCCGCGTACATGACAGAAGACCCCGTTCTGAGTCAGTGAT
GACGCACCAACGCAGATATCGATGTCTCACGTCTACCTTATTACTTGCCTTGATCGTCGGCACCCGATATTTGTCAA
CTGGGAATCCTCCTGGACCTATTATCGGTCCTTTGAAACTAACAGCAATTACCCCAGAAAGTGTCTGCTAA

>p2_ind14

ATGCAAATAGACCCCGTTGCATATCATTGTTCCGAGTTGAAGTTCGTGCTTGTACCCGAAGAGTCGGGAAAGTTAT
CCCCGACGGAAATAGGATTCCATTTTCAGACTGGGCGCCGCGTACATGACAGAAGACCCCGCTCTGAGTCAGTGA
TGACGCACCAACGCAGATATCGATGTCTCACGTCTACCTTATTACTTGCCTTGATCGTCGGCACCCGATATTTGTCA
ACTGGGAATCCTCCTGGACCTATTATCGGTCCTTTGAAACTAACAGCAATTACCCCAGAAAGTGTCTGCTAA

>p2_ind603

ATGCAAATAGACCCCGTTGCATATCATTGATCCGAGTTGAAGTTCGTGCTTGTACCCGAAGAGTCGGGAAAGTTAT
CCCCGACGGAAATAGGATTCCATTTTCAGATTGGGTGCCGCGTACATGACAGAAGACCCCGCTCTGAGTCAGTGA
TGACGCACCAACGCAGATATCGATGTCTCACGTCTACCTTATTACTTGCCTTGATCGTCGGCACCCGATATTTGTCA
ACTGGGAATCCTCCTGGACCTATTATCGGTCCTTTGAAACTAACAGCAATTACCCCAGAAAGTGTCTGCTAA

>p2_ind2731

ATGCAAATAGACCCCGTTGCATATCATTGTTCCGAGTTGAAGTTCGTGCTTGTACCCGAAGAGTCGGGAAAGTTAT
CCCCGACGGAAATAGGATTCCATTTTCAGATTGGGTGCCGCGTACATGACAGAAGACCCCGCTCTGAGTCAGTGA
TGACGCACCAACGCAGATATCGATGTCTCACGTCTACCTTATTACTTGCCTTGATCGTCGGCACCCGATATTTGTCA
ACTGGGAATCCTCCTGGACCTATTATCGGTCCTTTGAAACTAACAGCAATTACCCCAGAAAGTGTCTGCTAA

>p2_ind1154

ATGCAAATAGACCCCGTTGCATATCATTGATCCGAGTTGAAGTTCGTGCTTGTACCCGAAGAGTCGGGAAAGTTAT
CCCCGACGGAAATAGGATTCCATTTTCAGATTGGGTGCCGCGTACATGACAGAAGACCCCGCTCTGAGTCAGTGA
TGACGCACCAACGCAGATATCGATGTCTCACGTCTACCTTATTACTTGCCTTGATCGTCGGCACCCGATATTTGTCA
ACTGGGAATCCTCCTGGACCTATTATCGGTCCTTTGAAACTAACAGCAATTACCCCAGAAAGTGTCTGCTAA

>p2_ind200

ATGCAAATAGACCCCGTTGCATATCATTGTTCCGAGTTGAAGTTCGTGCTTGTACCCGAAGAGTCGGGAAAGTTAT
CCCCGACGGAAATAGGATTCCATTTTCAGATTGGGTGCCGCGTACATGACAGAAGACCCCGCTCTGAGTCAGTGA

TGACGCACCAACGCAGATATCGATGTCTCACGTCTACCTTATTACTTGCCTTGATCGTCGGCACCCGATATTTGTCA
ACTGGGAATCCTCCTGGACCTATTATCGGTCCTTTGAAACTAACAGCAATTACCCCAGAAAGTGTCTGCTAA

>p2_ind3351

ATGCAAATAGACCCCGTTGCATATCATTGTTCCGAGTTGAAGTTCGTGCTTGTACCCGAAGAGTCGGGAAAGTTAT
CCCCGACGGAAATAGGATTCCATTTTCAGATTGGGTGCCGCGTACATGACAGAAGACCCCGCTCTGAGTCAGTGA
TGACGCACCAACGCAGATATCGATGTCTCACGTCTACCTTATTACTTGCCTTGATCGTCGGCACCCGATATTTGTTA
ACTGGGAATCCTCCTGGACCTATTATCGGTCCTTTGAAACTAACAGCAATTACCCCATAAAGTGTCTGCTAA

>p2_ind2956

ATGCAAATAGACCCTGTTGCATATCATTGTTCCGAGTTGAAGTTCGTGCTTGTACCCGAAGAGTCGGGAAAGTTAT
CCCCGACGGAAATAGGATTCCATTTTCAGATTGGGTGCCGCGTACATGACAGAAGACCCCGCTCTGAGTCAGTGA
TGACGCACCAACGCAGATATCGATGTCTCACGTCTACCTTATTACTTGCCTTGATCGTCGGCACCCGATATTTGTCA
ACTGGGAATCCTCCTGGACCTATTATCGGTCCTTTGAAACTAACAGCAATTACCCCAGAAAGTGTCTGCTAA

>p2_ind3202

ATGCAAATAGACCCCGTTGCATATCATTGATCCGAGTTGAAGTTCGTGCTTGTACCCGAAGAGTCGGGAAAGTTAT
CCCCGACGGAAATAGGATTCCATTTTCAGATTGGGTGCCGCTTACATGACAGAAGACCCCGCTCTGAGTCAGTGAT
GACGCACCAACGCAGATATCGATGTCTCACGTCTACCTTATTACTTGCCTTGATCGTCGGCACCCGATATTTGTCAA
CTGGGAATCCTCCTGGACCTATTATCGGTCCTATGAAACTAACAGCAATTACCCCAGAAAGTGTCTGCTAA

>p2_ind2835

ATGCAAATAGACCCCGTTGCATATCATTGTTCCGAGTTGAAGTTCGTGCTTGTACCCGAAGAGTCGGGAAAGTTAT
CCCCGACGGAAATAGGATTCCATTTTCAGATTGGGTGCCGCGTACATGACAGAAGACCCCGCTCTGAGTCAGTGA
TGACGCACCAACGCAGATATCGATGTCTCACGTCTACCTTATTACTTGCCTTGATCGTCGGCACCCGATATTTGTCA
ACTGGGAATCCTCCTGGACCTATTATCGGTCCTTTGAAACTAACGGCAATTACCCCAGAAAGTGTCTGCTAA

>p2_ind2612

ATGCAAATAGACCCCGTTGCATATCATTGTTCCGAGTAGAAGTTCGTGCTTGTACCCGAAGAGTCGGGAAAGTTAT
CCCCGACGGAAATAGGATTCCATTTTCAGATTGGGTGCCGCGTACATGACAGAAGACCCCGCTCTGTGTCAGTGAT
GACGCACCTACGCAGATATCGATGTCTCAAGTCTACCTTATTACTTGCCTTGATCGTCGGCACCCGATATTTGTCAA
CTGGGAATCCTCCTGGACCTATTATCGGTCCTTTGAAACTAACAGCAATTACCCCAGAAAGTGTCTGCTAA

>p2_ind391

ATGCAAATAGACCCCGTTGCATATCATTGTTCCGAGTTGAAGTTCGTGCTTGTACCCGAAGAGTCGGGAAAGTTAT
CCCCGACGGAAATAGGATTCCATTTTCAGATTGGGTGCCGCGTACATGACAGAAGACCCCGCTCTGAGTCAGTGA
TGACGCACCAACGCAGATATCGATGTCTCACGTCTACCTTATTACTTGCCTTGATCGTCGGCACCCGATATTTGTCA
ACTGGGAATCCTCCTGGACCTATTATCGGTCCTTTGAAACTAACAGCAATTACCCCAGAAAGTGTCTGCTAA

>p2_ind1974

ATGCAAATAGACCCCGTTGCATATCATTGTTCCGAGTTGAAGTTCGTGCTTGTACCCGAAGAGTCGGGAAAGTTAT
CCCCGACGGAAATAGGATTACATTTTCAGATTGGGCGCCGCGTACATGACAGAAGACCCCGCTCTGAGTCAGTGA
TGACGCACCAACGCAGATATCGATGTCTCACGTCTACCTTATTACTTGCCTTGATCGTCGGCACCCGATATTTGTCA
ACTGGGAATCCTCCTGGACCTATTATCGGTCCTTTGAAACTAACAGCAATTACCCCAGAAAGTGTCTGCTAA

>p2_ind1925

ATGCAAATAGACCCCGTTGCATATCATTGTTCCGAGTTGAAGTTCGTGCTTGTACCCGAAGAGTCGGGAAAGTTAT
CCCCGACGGAAATAGGATTCCATTTTCAGATTGGGTGCCGCGTACATGACAGAAGACCCCGCTCTGAGTCAGTGA
TGACGCACCAACGCAGATATCGATGTCTCACGTCTACCTTATTACTTGCCTTGATCGTCGGCACCCGATATTTGTTA
ACTGGGAATCCTCCTGGACCTATTATCGGTCCTTTGAAACTAACAGCAATTACCCCATAAAGTGTCTGCTAA

>p2_ind1574

ATGCAAATAGACCCCGTTGCATATCATTGTTCCGAGTTGAAGTTCGTGCTTGTACCCGAAGAGTCGGGAAAGTTAT
CCCCGACGGAAATAGGATTCCATTTTCAGATTGGGTGCCGCGTACATGACAGAAGACCCCGCTCTGAGTCAGTGA
TGTCGCACCAACGCAGATATCGATGTCTCACGTCTACCTTATTACTTGCCTTGATCGTCGGCACCCGATATTTGTCA
ACTGGGAATCCTCCTGGACCTATTATCGGTCCTTTGAAACTAACAGCAATTACCCCAGAAAGTGTCTGCTAA

>p2_ind924

ATGCAAATAGACCCCGTTGCATATCATTGTTCCGAGTTGAGGTTCGTGCTTGTACCCGAAGAGTCGGGAAAGTTAT
CCCCGACGGAAATAGGATTCCATTTTCAGATTGGGTGCCGCGTACATGACAGAAGACCCCGCTCTGAGTCAGTTAT
GACGCACCAACGCAGATATCGATGTCTCACGTCTACCTTATTACTTGCCTTGACCGTCGGCACCCGATATTTGTCAA
CTGGGAATCCTCCTAGACCTATTATCGGTCCTTTGAAACTAACAGCAATTACCCCAGAAAGTGTCTGCTAA

>p2_ind1624

ATGCAAATAGACCCCGTTGCATATCATTGTTCCGAGTTGAAGTTCGTGCTTGTACCCGAAGAGTCGGGAAAGTTAT
CCCCGACGGAAATAGGATTCCATTTTCAGATTGGGTGCCGCGTACATGACAGAAGACCCCGCTCTGAGTCAGTGA
TGACGCACCAACGCAGATATCGATGTCTCACGTCTACCTTATTACTTGCCTTGATCGTCGGCACCCGATATTTGTCA
ACTGGGAATCCTCCTGGACCTATTATCGGTCCTTTGAAACTAAAAGCAATTACCCCAGAAAGTGTCTGCTAA

>p2_ind4427

ATGCAAATAGACCCCGTTGCATATCATTGTTCCGAGTTGAAGTTCGTGCTTGTACCCGAAGAGTCGGGAAAGTTAT
CCCCGACGGAAATAGGATTCCATTTTCAGATTGGGTGCCGCGTACATGACAGAAGACCCCGCTCTGAGTCAGTGA
TGACGCACCAACGCAGACATCGATGTCTCACGTCTACCTTATTACTTGCCTTGATCGTCGGCACCCGATATTTGTCA
ACTGGGAATCCTCCTGGACCTATTATCGGTCCTTTGAAACTAACAGCAATTACCCCAGAAAGTGTCTGCTAA

>p2_ind951

ATGCAAATAGACCCCGTTGCATATCATTGTTCCGAGTTGAAGTTCGTGCTTGTACCCGAAGAGTCGGGAAAGTTAT
CCCCGACGGAAATAGGATTCCATTTTCAGATTGGGTGCCGCGTACATGACAGAAGACCCCGCTCTGAGTCAGTGA

TGACGCACCAACGCAGACATCGATGTCTCACGTCTACCTTATTACTTGCCTTGATCGTCGGCACCCGATATTTGTCA
ACTGGGAATCCTCCTGGACCTATTATCGGTCCTTTGAAACTAACAGCAATTACCCCAGAAAGTGTCTGCTAA

>p2_ind60

ATGCAAATAGACCCCGTTGCATATCATTGTTCCGAGTTGAAGTTCGTGCTTGTACCCGAAGAGTCGGGAAAGTTAT
CCCCGACGGAAATAGGATTCCATTTTCAGACTGGGTGCCGCGTACATGACAGAAGACCCCGCTCTGAGTCAGTGA
TGACGCACCAACGTAGATATCGATGTCTCACGTCTACCTTATTACTTGCCTTGATCGTCGGCACCCGATATTTGTCA
ACTGGGAATCCTCCTGGACCTATTATCGGTCCTTTGAAACTAACAGCAATTACCCCAGAAAGTGTCTGCTAA

>p2_ind2233

ATGCAAATAGACCCCGTTGCATATCATTGATCCGAGTTGAAGTTCGTGCTTGTACCCGAAGAGTCGGGAAAGTTAT
CCCCGACGGAAATAGGATTCCATTTTCAGATTGGGTGCCGCGTACATGACAGAAGACCCCGTTCTGAGTCAGTGAT
GACGCACCAACGCAGATATCGATGTCTCACGTCTACCTAATTACTTGCCTTGATCGTCGGCACCCGATATTTGTCAA
CTGGGAATCCTCCTGGACCTATTATCGGTCCTTTGAAACTAACAGCAATTACCCCAGAAAGTGTCTGCTAA

>p2_ind1882

ATGCAAATAGACCCCGTTGCATATCATTGTTCCGAGTTGAAGTTCGTGCTTGTACCCGAAGAGTCGGGAAAGTTAT
CCCCGACGGAAATAGGATTCCATTTTCAGATTGGGTGCCGCGTACATGACAGAAGACCCCGCTCTGAGTCAGTGA
TGACGCACCAACGCAGATATCGATGTCTCACGTCTACCTTATTACTTGCCTTGATCGTCGGCACCCGATATTTGTCA
ACTGGGAATCCTCCTGGACCTATTATCGGTCCTTTGAAACTAACAGCAATTACCCCAGAAAGTGTCTGCTAA

>p2_ind1947

ATGCAAATAGACCCCGTTGCATATCATTGTTCCGAGTTGAAGTTCGTGCTTGTACCCGAAGAGTCGGGAAAGTTAT
CCCCGACGGAAATAGGATTCCATTTTCAGATTGGGTGCCGCGTACATGACAGAAGACCCCGCTCTGAGTCAGTGA
TGACGCACCAACGCAGATATCGATGTCTCACGTCTACCTTATTACTTGCCTTGATCGTCGGCACCCGATATTTGTCA
ACTGGGAATCCTCCTGGACCTATTATCGGTCCTTTGAAACTAACAGGAATTACCCCAGAAAGTGTCTGCTAA

>p2_ind3534

ATGCAAATAGACCCCGTTGCATATCATTGTTCCGAGTTGAGGTTCGTGCTTGTACCCGAAGAGTCGGGAAAGTTAT
CCCCGACGGAAATAGGATTCCATTTTCAGATTGGGTGCCGCGTACATGACAGAAGACCCCGCTCTGAGTCAGTTAT
GACGCACCAACGCAGATATCGATGTCTCACGTCTACCTTATTACTTGCCTTGACCGTCGGCACCCGATATTTGTCAA
CTGGGAATCCTCCTAGACCTATTATCGGTCCTTTGAAACTAACAGCAATTACCCCAGAAAGTGTCTGCTAA

>p2_ind3247

ATGCAAATAGACCCCGTTGCATATCATTGTTCCGAGTTGAAGTTCGTGCTTGTACCCGAAGAGTCGGGAAAGTTAT
CCCCGACGGAAATAGGATTACATTTTCAGATTGGGCGCCGCGTACATGACAGAAGACCCCGCTCTGAGTCAGTGA
TGACGCACCTACGCAGATATCGATGTCTCACGTCTACCTTATTACTTGCCTTGATCGTCGGCACCCGATATTTGTCA
ACTGGGAATCCTCCTGGACCTATTATCGGTCCTTTGAAACTAACAGCAATTACCCCAGAAAGTGTCTGCTAA

>p2_ind460

ATGCAAATAGACCCCGTTGCATATCATTGTTCCGAGTTGAAGTTCGTGCTTGTACCCGAAGAGTCGGGAAAGTTAT
CCCCGACGGAAATAGGATTCCATTTTCAGATTGGGTGCCGCGTACATGACAGAAGACCCCGCTCTGAGTCAGTGA
TGACGCACCAACGCAGATATCGATGTCTCACGTCTACCTTATTACTTGCCTTGATCGTCGGCACCCGATATTTGTCA
ACTGGGAATCCTCCTGGACCTATTATCGGTCCTTTGAAACTAACAGCAATTACTCCAGAAAGTGTCTGCTAA

>p2_ind4039

ATGCAAATAGACCCCGTTGCATATCATTGATCCGAGTTGAAGTTCGTGCTTGTACCCGAAGAGTCGGGAAAGTTAT
CCCCGACGGAAATAGGATTCCATTTTCAGATTGGGTGCCGCGTACATGACAGAAGACCCCGCTCTGAGTCAGTGA
TGACGCACCAACGCAGATATCGATGTCTCACGTCTACCTTATTACTTGCCTTGATCGTCGGCACCCGATATTTGTCA
ACTGGGAATCCTCCTGGACCTATTATCGGTCCTTTGAAACTAACAGCAATTACCCCAGAAAGTGTCTGCTAA

>p2_ind3226

ATGCAAATAGACCCCGTTGCATATCATTGTTCCGAGTTGAAGTTCGTGCTTGTACCCGAAGAGTCGGGAAAGTTAT
CCCCGACGGAAATAGGATTCCATTTTCAGATTGGGTGCCGCGTACATGACAGAAGACCCCGCTCTGAGTCAGTGA
TGACGCACCAACGCAGATATCGATGTCTCACGTCTACCTTATTACTTGCCTTGATCGTCGGCACCCGATATTTGTCA
ACTGGGAATCCTCCTGGACCTATTATCGGTCCTTTGAAACTAACAGCAATTACTCCAGAAAGTGTCTGCTAA

>p2_ind1376

ATGCAAATAGACCCCGTTGCATATCATTGTTCCGAGTTGAAGTTCGTGCTTGTACCCGAAGAGTCGGGAAAGTTAT
CCCCGACGGAAATAGGATTCCATTTTCAGATTGGGTGCCGCGTACATGACAGAAGACCCCGCTCTGAATCAGTGAT
GACGCACCAACGCAGATATCGATGTCTCACGTCTACCTTATTACTTGCCTTGATCGTCGGCACCCGATATTTGTCAA
CTGGGAATCCTCCTGGACCTATTATCGGTCCTTTGAAACTAACAGCAATTACCCCAGAAAGTGTCTGCTAA

>p2_ind3846

ATGCAAATAGACCCCGTTGCATATCATTGTTCCGAGTTGAAGTTCGTGCTTGTACCCGAAGAGTCGGGAAAGTTAT
CCCCGACGGAAATAGGATTCCATTTTCAGATTGGGTGCCGCGTACATGACAGAAGACCCCGCTCTGAGTCAGTGA
TGGCGCACCAACGCAAATATCGATGTCTCACGTCTACCTTATTACTTGCCTTGATCGTCGGCACCCGATATTTGTCA
ACTGGGAATCCTCCTGGACCTATTATCGGTCCTTTGAAACTAACAGCAATTACCCCAGAAAGTGTCTGCTAA

>p2_ind2323

ATGCAAATAGACCCCGTTGCATATCATTGTTCCGAGTTGAAGTTCGTGCTTGTACCCGAAGAGTCGGGAAAGTTAT
CCCCGACGGAAATAGGATTCCATTTTCAGATTGGGTGCCGCGTACATGACAGAAGACCCCGCTCTGAGTCAGTGA
TGACGCACCAACGCAGATATCGATGTCTCACGTCTACCTTATTACTTGCCTTGATCGTCGGCACCCGATATTTGTCA
ACTGGGAATCCTCCTGGACCTATTATCGGTCCTTTGAAACTAACAGCAATTACCCCAGAAAGTGTCTGCTAA

>p2_ind1093

ATGCAAATAGACCCCGTTGCATATCATTGTTCCGAGTTGAAGTTCGTGCTTGTACCCGAAGAGTCGGGAAAGTTAT
CCCCGACGGAAATAGGATTCCATTTTCAGATTGGGTGCCGCGTACATGACAGAAGACCCCGCTCTGAGTCAGTGA

TGACGCACCAACGCAGATATCGATGTCTCACGTCTACCTTATTACTTGCCTTGATCGTCGGCACCCGATATTTGTCA
ACTGGGAATCCTCCTGGACCTATTATCGGTCCTTTGAAACTAACAGCAATTACCCCAGAAAGTGTCTGCTAA

>p2_ind1171

ATGCAAATAGACCCCGTTGCATATCATTGTTCCGAGTTGAAGTTCGTGCTTGTACCCGAAGAGTCGGGAAAGTTAT
CCCCGACGGAAATAGGATTCCATTTTCAGATTGGGTGCCGCGTACATGACAGAAGACCCCGCTCTGAGTCAGTGA
TGGCGCACCAACGCAAATATCGATGTCTCACGTCTACCTTATTACTTGCCTTGATCGTCGGCACCCGATATTTGTCA
ACTGGGAATCCTCCTGGACCTATTATCGGTCCTTTGAAACTAACAGCAATTACCCCAGAAAGTGTCTGCTAA

>p2_ind4265

ATGCAAATAGACCCCGTTGCATATCATTGTTCCGAGTTGAAGTTCGTGCTTGTACCCGAAGAGTCGGGAAAGTTAT
CCCCGACGGAAATAGGATTCCATTTTCAGATTGGGTGCCGCGTACATGACAGAAGACCCCGCTCTGAGTCAGTGA
TGACGCACCAACGCAGATATCGATGTCTCACGTCTACCTTATTACTTGCCTTGATCGTCGGCACCCGATATTTGTCA
ACTGGGAATCCTCCTGGACCTATTATCGGTCCTTTGAAACTAAAAGCAATTACCCCAGAAAGTGTCTGCTAA

>p2_ind4619

ATGCAAATAGACCCCGTTGCATATCATTGTTCCGAGTTGAAGTTCGTGCTTGTACCCGAAGAGTCGGGAAAGTTAT
CCCCGACGGAAATAGGATTCCATTTTCAGATTGGGTGCCGCGTACATGACAGAAGACCCCGCTCTGAGTCAGTGA
TGACGCTCCAACGCAAATACAGATGTCTCACGTCTACCTTAATACTTGCCTTGATCGTCGGCACCCGATATTTGTCA
ACTGGGAATCCTCCTGGACCTATTATCGGTCCTTTGAAACTAACAGCAATTACCCCAGAAAGTGTCTGCTAA

>p2_ind2778

ATGCAAATAGACCCCGTTGCATATCATTGTTCCGAGTTGAAGTTCGTGCTTGTACCCGAAGAGTCGGGAAAGTTAT
CCCCGACGGAAATAGGATTCCATTTTCAGATTGGGTGCCGCGTACATGACAGAAGACCCCGCTCTGAGTCAGTGA
TGTCGCACCAACGCAGATATCGATGTCTCACGTCTACCTTATTACTTGCCTTGATCGTCGGCACCCGATATTTGTCA
ACTGGGAATCCTCCTGGACCTATTATCGGTCCTTTGAAACTAACAGCAATTACCCCAGAAAGTGTCTGCTAA

>p2_ind969

ATGCAAATAGACCCCGTTGCATATCATTGTTCCGAGTTGAAGTTCGTGCTTGTACCCGAAGAGTCGGGAAAGTTAT
CCCCGACGGAAATAGGATTCCATTTTCAGATTGGGTGCCGCGTACATGACAGAAGACCCCGCTCTGAGTCAGTGA
TGACGCACCTACGCAGATATCGATGTCTCAAGTCTACCTTATTACTTGCCTTGATCGTCGGCACCCGATATTTGTCA
ACTGGGAATCCTCCTGGACCTATTATCGGTCCTTTGAAACTAACAGCAATTACCCCAGAAAGTGTCTGCTAA

>p2_ind4885

ATGCAAATAGACCCCGTTGCATATCATTGTTCCGAGTTGAAGTTCGTGCTTGTACCCGAAGAGTCGGGAAAGTTAT
CCCCGACGGAAATAGGATTCCATTTTCAGATTGGGTGCCGCGTACATGACAGAAGACCCCGCTCTGAGTCAGTGA
TGACGCACCAACGCAGATATCGATGTCTCACGTCTACCTTATTACTTGCCTTGATCGTCGGCACCCGATATTTGTCA
ACTGGGAATCCTCCTGGACCTATTATCGGTCCTTTGAAACTAACGGCAATTACCCCAGAAAGTGTCTGCTAA

>p2_ind961

ATGCAAATAGACCCCGTTGCATATCATTGTTCCGAGTTGAAGTTCGTGCTTGTACCCGAAGAGTCGGGAAAGTTAT
CCCCGACGGAAATAGGATTCCATTTTCAGATTGGGTGCCGCGTACATGACAGAAGACCCCGCTCTGAGTCAGTGA
TGACGCACCAACGCAGATATCGATGTCTCACGTCTACCTTATTACTTGCCTTGATCGTCGGCACCCGATATTTGTCA
ACTGGGAATCCTCCTGGACCTATTATCGGTCCTTTGAAACTAACAGCAATTACTCCAGAAAGTGTCTGCTAA

>p2_ind3566

ATGCAAATAGACCCCGTTGCATATCATTGTTCCGAGTTGAAGTTCGTGCTTGTACCCGAAGAGTCGGGAAAGTTAT
CCCCGACGGAAATAGGATTCCATTTTCAGATTGGGTGCCGCGTACATGACAGAAGACCCCGCTCTGAGTCAGTGA
TGACGCACCAACGCAGATATCGATGTCTCACGTCTACCTTATTACTTGCCTTGATCGTCGGCACCCGATATTTGTCA
ACTGGGAATCCTCCTGGACCTATTATCGGTCCTTTGAAACTAACAGCAATTACTCCAGAAAGTGTCTGCTAA

>p2_ind4054

ATGCAAATAGACCCCGTTGCATATCATTGTTCCGAGTTGAAGTTCGTGCTTGTACCCGAAGAGTCGGGAAAGTTAT
CCCCGACGGAAATAGGATTCCATTTTCAGATTGGGTGCCGCGTACATGACAGAAGACCCCGCTCTGAGTCAGTGA
TGACGCACCAACGCAGATATCGATGTCTCACGTCTACCTTATTACTTGCCTTGATCGTCGGCACCCGATATTTGTCA
ACTGGGAATCCTCCTGGACCTATTATCGGTCCTTTGAAACTAACAGCAATTACCCCAGAAAGTGTCTGCTAA

>p2_ind2722

ATGCAAATAGACCCTGTTGCATATCATTGTTCCGAGTTGAAGTTCGTGCTTGTACCCGAAGAGTCGGGAAAGTTAT
CCCCGACGGAAATAGGATTCCATTTTCAGATTGGGTGCCGCGTACATGACAGAAGACCCCGCTCTGAGTCAGTGA
TGGCGCACCAACGCAAATACAGATGTCTCACGTCTACCTTATTACTTGCCTTGATCGTCGGCACCCGATATTTGTCA
ACTGGGAATCCTCCTGGACCTATTATCGGTCCTTTGAAACTAACAGCAATTACCCCAGAAAGTGTCTGCTAA

>p2_ind4005

ATGCAAATAGACCCTGTTGCATATCATTGTTCCGAGTTGAAGTTCGTGCTTGTACCCGAAGAGTCGGGAAAGTTAT
CCCCGACGGAAATAGGATTCCATTTTCAGATTGGGTGCCGCGTACATGACAGCAGACCCCGCTCTGAGTCAGTGAT
GGCGCACCAACGCAAATATCGATGTCTCACGTCTACCTTATTACTTGCCTTGATCGTCGGCACCCGATATTTGTCAA
CTGGGAATCCTCCTGGACCTATTATCGGTCCTTTGAAACTAACAGCAATTACCCCAGAAAGTGTCTGCTAA

>p2_ind280

ATGCAAATAGACCCTGTTGCATATCATTGTTCCGAGTTGAAGTTCGTGCTTGTACCCGAAGAGTCGGGAAAGTTAT
CCCCGACGGAAATAGGATTCCATTTTCAGATTGGGTGCCGCGTACATGACAGAAGACCCCGCTCTGAGTCAGTGA
TGGCGCACCAACGCAAATACAGATGTCTCACGTCTACCTTAATACTTGCCTTGATCGTCGGCACCCGATATTTGTCA
ACTGGGAATCCTCCTGGACCTATTATCGGTCCTTTGAAACTAACAGCAATTACCCCAGAAAGTGTCTGCTAA

>p2_ind4386

ATGCAAATAGACCCCGTTGCATATCATTGTTCCGAGTTGAAGTTCGTGCTTGTACCCGAAGAGTCGGGAAAGTTAT
CCCCGACGGAAATAGGATTCCATTTTCAGACTGGGCGCCGCGTACATGACAGAAGACCCCGCTCTGAGTCAGTGA

TGACGCACCAACGCAGATATCGATGTCTCACGTCTACCTTATTACTTGCCTTGATCGTCGGCACCCGATATTTGTCA
ACTGGGAATCCTCCTGGACCTATTATCGGTCCTTTGAAACTAACAGCAATTACCCCAGAAAGTGTCTGCTAA

>p2_ind352

ATGCAAATAGACCCCGTTGCATATCATTGTTCCGAGTTGAAGTTCGTGCTTGTACCCGAAGAGTCGGGAAAGTTAT
CCCCGACGGAAATAGGATTCCATTTTCAGATTGGGTGCCGCGTACATGACAGAAGACCCCGCTCTGAGTCAGTGA
TGTCGCACCAACGCAGATATCGATGTCTCACGTCTACCTTATTACTTGCCTTGATCGTCGGCACCCGATATTTGTCA
ACTGGGAATCCTCCTGGACCTATTATCGGTCCTTTGAAACTAACAGCAATTACCCCAGAAAGTGTCTGCTAA

>p2_ind134

ATGCAAATAGACCCCGTTGCATATCATTGTTCCGAGTTGAAGTTCGTGCTTGTACCCGAAGAGTCGGGAAAGTTAT
CCCCGACGGAAATAGGATTCCATTTTCAGATTGGGTGCCGCGTACATGACAGAAGACCCCGCTCTGAGTCAGTGA
TGACGCACCAACGCAGATATCGATGTCTCACGTCTACCTTATTACTTGCCTTGATCGTCGGCACCCGATATTTGTCA
ACTGGGAATCCTCCTGGACCTATTATCGGTCCTTTGAAACTAACAGCAATTACCCCAGAAAGTGTCTGCTAA

>p2_ind4387

ATGCAAATAGACCCCGTTGCATATCATTGTTCCGAGTTGAAGTTCGTGCTTGTACCCGAAGAGTCGGGAAAGTTAT
CCCCGACGGAAATAGGATTCCATTTTCAGATTGGGTGCCGCGTACATGACAGAAGACCCCGCTCTGAGTCAGTGA
TGACGCACCAACGCAGATATCGATGTCTCACGTCTACCTTATTACTTGCCTTGATCGTCGGCACCCGATATTTGTCA
ACTGGGAATCCTCCTGGACCTATTATCGGTCCTTTGAAACTAACAGCAATTACCCCAGAAAGTGTCTGCTAA

>p2_ind1868

ATGCAAATAGACCCCGTTGCATATCATTGTTCCGAGTTGAAGTTCGTGCTTGTACCCGAAGAGTCGGGAAAGTTAT
CCCCGACGGAAATAGGATTCCATTTTCAGATTGGGCGCCGCGTACATGACAGAAGACCCCGCTCTGAGTCAGTGA
TGACGCACCAACGCAGATATCGATGTCTCACGTCTACCTTATTACTTGCCTTGATCGTCGGCACCCGATATTTGTCA
ACTGGGAATCCTCCTGGACCTATTATCGGTCCTTTGAAACTAACAGCAATTACCCCAGAAAGTGTCTGCTAA

>p2_ind96

ATGCAAATAGACCCCGTTGCATATCATTGTTCCGAGTTGAAGTTCGTGCTTGTACCCGAAGAGTCGGGAAAGTTAT
CCCCGACGGAAATAGGATTACATTTTCAGATTGGGCGCCGCGTACATGACAGAAGACCCCGCTCTGAGTCAGTGA
TGACGCACCAACGCAGATATCGATGTCTCACGTCTACCTTATTACTTGCCTTGATCGTCGGCACCCGATATTTGTCA
ACTGGGAATCCTCCTGGACCTATTATCGGTCCTTTGAAACTAACAGCAATTACCCCAGAAAGTGTCTGCTAA

>p2_ind2192

ATGCAAATAGACCCCGTTGCATATCATTGTTCCGAGTTGAAGTTCGTGCTTGTACCCGAAGAGTCGGGAAAGTTAT
CCCCGACGGAAATAGGATTCCATTTTCAGATTGGGTGCCGCGTACATGACAGAAGACCCCGCTCTGAGTCAGTGA
TGACGCTCCAACGCAGATATCGATGTCTCACGTCTACCTTATTACTTGCCTTGATCGTCGGCACCCGATATTTGTCA
ACTGGGAATCCTCCTGGACCTATTATCGGTCCTTTGAAACTAACAGCAATTACCCCAGAAAGTGTCTGCTAA

>p2_ind4213

ATGCAAATAGACCCCGTTGCATATCATTGTTCCGAGTAGAAGTTCGTGCTTGTACCCGAAGAGTCGGGAAAGTTAT
CCCCGACGGAAATAGGATTCCATTTTCAGACTGGGCGCCGCGTACATGACAGAAGACCCCGCTCTGAGTCAGTGA
TGACGCACCAACGCAGATATCGATGTCTCACGTCTACCTTATTACTTGCCTTGATCGTCGGCACCCGATATTTGTCA
ACTGGGAATCCTCCTGGACCTATTATCGGTCCTTTGAAACTAACAGCAATTACCCCAGAAAGTGTCTGCTAA

>p2_ind2818

ATGCAAATAGACCCCGTTGCATATCATTGTTCCGAGTTGAAGTTCGTGCTTGTACCCGAAGAGTCGGGAAAGTTAT
CCCCGACGGAAATAGGATTCCATTTTCAGATTGGGTGCCGCGTACATGACAGAAGACCCCGCTCTGAGTCAGTGA
TGTCGCACCAACGCAGATATCGATGTCTCACGTCTACCTTATTACTTGCCTTGATCGTCGGCACCCGATATTTGTCA
ACTGGGAATCCTCCTGGACCTATTATCGGTCCTTTGAAACTAACAGCAATTACCCCAGAAAGTGTCTGCTAA

>p2_ind1122

ATGCAAATAGACCCCGTTGCATATCATTGTGCCGAGTTGAAGTTCGTGCTTGTACCCGAAGAGTCGGGAAAGTTAT
CCCCGACGGAAATAGGATTTCATTGTCAGATTGGGTGCCGCGTACATGACAGAAGACCCCGCTCTGAGTCAGTGA
TGGCGCACCAACGCAAATATCGATGTCTCACGTCTACCTTATTACTTGCCTTGATCGTCGGCACCCGATATTTGTCA
ACTGGGAATCCTCCTGGACCTATTATCGGTCCTTTGAAACTAACAGCAATTACCCCAGAAAGTGTCTGCTAA

>p2_ind4281

ATGCAAATAGACCCCGTTGCATATCATTGTTCCGAGTTGAAGTTCGTGCTTGTACCCGAAGAGTCGGGAAAGTTAT
CCCCGACGGAAATAGGATTCCATTTTCAGATTGGGTGCCGCGTACATGACAGAAGACCCCGCTCTGAGTCAGTGA
TGACGCACCAACGCAGATATCGATGTCTCACGTCTACCTTATTACTTGCCTTGATCGTCGGCACCCGATATTTGTCA
ACTGGGAATCCTCCTGGACCTATTATCGGTCCTTTGAAACTAACAGCAATTACCCCAGAAAGTGTCTGCTAA

>p2_ind3679

ATGCAAATAGACCCCGTTGCATATCATTGTTCCGAGTTGAAGTTCGTGCTTGTACCCGAAGAGTCGGGAAAGTTAT
CCCCGACGGAAATAGGATTCCATTTTCAGATTGGGTGCCGCGTACATGACAGAAGACCCCGCTCTGAGTCAGTGA
TGACGCACCAACGCAGATATCGATGTCTCACGTCTACCTTATTACTTGCCTTGATCGTCGGCACCCGATATTTGTCA
ACTGGGAATCCTCCTGGACCTATTATCGGTCCTTTGAAACTAACAGCAATTACCCCAGAAAGTGTCTGCTAA

>p2_ind431

ATGCAAATAGACCCCGTTGCATATCATTGTTCCGAGTTGAAGTTCGTGCTTGTACCCGAAGAGTCGGGAAAGTTAT
CCCCGACGGAAATAGGATTCCATTTTCAGATTGGGTGCCGCGTACATGACAGAAGACCCCGCTCTGAGTCAGTGA
TGACGCACCAACGCAGATATCGATGTCTCACGTCTACCTTATTACTTGCCTTGATCGTCGGCACCCGATATTTGTCA
ACTGGGAATCCTCCTGGACCTATTATCGGTCCTTTGAAACTAACAGCAATTACTCCAGAAAGTGTCTGCTAA

>p2_ind4731

ATGCAAATAGACCCCGTTGCATATCATTGTTCCGAGTTGAAGTTCGTGCTTGTACCCGAAGAGTCGGGAAAGTTAT
CCCCGACGGAAATAGGATTCCATTTTCAGATTGGGTGCCGCGCACATGACAGAAGACCCCGCTCTGAGTCAGTGA

TGACGCACCTACGCAGATATCGATGTCTCACGTCTACCTTATTACTTGCCTTGATCGTCGGCACCCGATATTTGTCA
ACTGGGAATCCTCCTGGACCTATTATCGGTCCTTTGAAACTAACAGCAATTACCCCAGAAAGTGTCTGCTAA

>p2_ind1628

ATGCAAATAGACCCCGTTGCATATCATTGTTCCGAGTTGAAGTTCGTGCTTGTACCCGAAGAGTCGGGAAAGTTAT
CCCCGACGGAAATAGGATTCCATTTTCAGATTGGGTGCCGCGTACATGACAGAAGACCCCGCTCTGAGTCAGTGA
TGACGCACCAACGCAGATATCGATGTCTCACGTCTACCTTATTACTTGCCTTGATCGTCGGCACCCGATATTTGTCA
ACTGGGAATCCTCCTGGACCTATTATCGGTCCTTTGAAACTAACGGCAATTACCCCAGAAAGTGTCTGCTAA

>p2_ind4494

ATGCAAATAGACCCCGTTGCATATCATTGTTCCAAGTTGAAGTTCGTGCTTGTACCCGAAGAGTCGGGAAAGTTAT
CCCCGACGGAAATAGGATTCCATTTTCAGATTGGGTGCCGCGTACATGACAGAAGACCCCGCTCTGAGTCAGTGA
TGACGCACCAACGCAGATATCGATGTCTCACGTCTACCTTATTACTTGCCTTGATCGTCGGCACCCGATATTTGTCA
ACTGGGAATCCTCCTGGACCTATTATCGGTCCTTTGAAACTAACAGCAATTACCCCAGAAAGTGTCTGCTAA

>p2_ind468

ATGCAAATAGACCCCGTTGCATATCATTGTTCCGAGTTGAAGTTCGTGCTTGTACCCGAAGAGTCGGGAAAGTTAT
CCCCGACGGAAATAGGATTCCATTTTCAGATTGGGTGCCGCGTACATGACAGAAGACCCCGCTCTGAGTCAGTGA
TGACGCACCAACGCAGATATCGATGTCTCACGTCTACCTTATTACTTGCCTTGATCGTCGGCACCCGATATTTGTCA
ACTGGGAATCCTCCTGGACCTATTATCGGTCCTTTGAAACTAACAGCAATTACCCCAGAAAGTGTCTGCTAA

>p2_ind1083

ATGCAAATAGACCCCGTTGCATATCATTGTTCCGAGTTGAAGTTCGTGCTTGTACCCGAAGAGTCGGGAAAGTTAT
CCCCGACGGAAATAGGATTCCATTTTCAGATTGGGTGCCGCGTACATGACAGAAGACCCCGCTCTGAGTCAGTGA
TGACGCACCAACGCAGATATCGATGTCTCACGTCTACCTTATTACTTGCCTTGATCGTCGGCACCCGATATTTGTCA
ACTGGGAATCCTCCTGGACCTATTATCGGTCCTTTGAAACTAACAGCAATTACCCCAGAAAGTGTCTGCTAA

>p2_ind2495

ATGCAAATAGACCCCGTTGCATATCATTGTTCCGAGTTGAAGTTCGTGCTTGTACCCGAAGAGTCGGGAAAGTTAT
CCCCGACGGAAATAGGATTCCATTTTCAGATTGGGTGCCGCGTACATGACAGAAGACCCCGCTCTGAGTCAGTGA
TGACGCACCAACGCAGATATCGATGTCTCACGTCTACCTTATTACTTGCCTTGATCGTCGGCACCCGATATTTGTCA
ACTGGGAATCCTCCTGGACCTATTATCGGTCCTTTGAAACTAACAGCAATTACCCCAGAAAGTGTCTGCTAA

>p2_ind3716

ATGCAAATAGCCCCCGTTGCATATCATTGATCCGAGTTGAAGTTCGTGCTTGTACCCGAAGAGTCGGGAAAGTTAT
CCCCGACGGAAATAGGATTCCATTTTCAGATTGGGTGCCGCGTACATGACAGAAGACCCCGCTCTGAGTCAGTGA
TGACGCACCAACGCAGATATCGATGTCTCACGTCTACCTTATTACTTGCCTTGATCGTCGGCACCCGATATTTGTCA
ACTGGGAATCCTCCTGGACCTATTATCGGTCCTTTGAAACTAACAGCAATTACCCCAGAAAGTGTCTGCTAA

>p2_ind558

ATGCAAATAGACCCCGTTGCATATCATTGATCCGAGTTGAAGTTCGTGCTTGTACCCGAAGAGTCGGGAAAGTTAT
CCCCGACGGAAATAGGATTCCATTTTCAGATTGGGTGCCGCGTACATGACAGAAGACCCCGTTCTGAGTCAGTGAT
GACGCACCAACGCAGATATCGATGTCTCACGTCTACCTTATTACTTGCCTTGATCGTCGGCACCCGATATTTGTCAA
CTGGGAATCCTCCTGGACCTATTATCGGTCCTTTGAAACTAACAGCAATTACCCCAGAAAGTGTCTGCTAA

>p2_ind3836

ATGCAAATAGACCCCGTTGCATATCATTGTTCCGAGTTGAAGTTCGTGCTTGTACCCGAAGAGTCGGGAAAGTTAT
CCCCGACGGAAATAGGATTACATTTTCAGATTGGGCGCCGCGTACATGACAGAAGACCCCGCTCTGAGTCAGTGA
TGACGCACCAACGCAGATATCGATGTCTCACGTCTACCTTATTACTTGCCTTGATCGTCGGCACCCGATATTTGTCA
ACTGGGAATCCTCCTGGACCTATTATCGGTCCTTTGAAACTAACAGCAATTACCCCAGAAAGTGTCTGCTAA

>p2_ind4533

ATGCAAATAGACCCCGTTGCATATCATTGTTCCGAGTTGAAGTTCGTGCTTGTACCCGAAGAGTCGGGAAAGTTAT
CCCCGACGGAAATAGGATTCCATTTTCAGACTGGGCGCCGCGTACATGACAGAAGACCCCGCTCTGAGTCAGTGA
TGACGCACCAACGCAGATATCGATGTCTCACGTCTACCTTATTACTTGCCTTGATCGTCGGCACCCGATATTTGTCA
ACTGGGAATCCTCCTGGACCTATTCTCGGTCCTTTGAAACTAACAGCAATTACCCCAGAAAGTGTCTGCTAA

>p2_ind1707

ATGCAAATAGACCCCGTTGCATATCATTGTTCCGAGTTGAAGTTCGTGCTTGTACCCGAAGAGTCGGGAAAGTTAT
CCCCGACGGAAATAGGATTCCATTTTCAGATTGGGTGCCGCGTACATGACAGAAGACCCCGCTCTGAGTCAGTGA
TGACGCACCAACGCAGATATCGATGTCTCACGTCTACCTTATTACTTGCCTTGATCGTCGGCACCCGATATTTGTCA
ACTGGGAATCCTCCTGGACCTATTATCGGTCCTTTGAAACTAACAGCAATTACCCCAGAAAGTGTCTGCTAA

>p2_ind2268

ATGCAAATAGACCCCGTTGCATATCATTGTTCCGAGTTGAAGTTCGTGCTTGTACCCGAAGAGTCGGGAAAGTTAT
CCCCGACGGAAATAGGATTCCATTTTCAGACTGGGCGCCGCGTACATGACAGAAGACCCCGCTCTGAGTCAGTGA
TGACGCACCAACGCAGACATCGATGTCTCACGTCTACCTTATTACTTGCCTTGATCGTCGGCACCCGATATTTGTCA
ACTGGGAATCCTCCTGGACCTATTATCGGTCCTTTGAAACTAACAGCAATTACCCCAGAAAGTGTCTGCTAA

>p2_ind2277

ATGCAAATAGACCCCGTTGCATATCATTGTTCCAAGTTGAAGTTCGTGCTTGTACCCGAAGAGTCGGGAAAGTTAT
CCCCGACGGAAATAGGATTCCATTTTCAGATTGGGTGCCGCGTACATGACAGAAGACCCCGCTCTGAGTCAGTGA
TGACGCACCAACGCAGATATCGATGTCTCACGTCTACCTTATTACTTGCCTTGATCGTCGGCACCCGATATTTGTCA
ACTGGGAATCCTCCTGGACCTATTATCGGTCCTTTGAAACTAACAGCAATTACCCCAGAAAGTGTCTGCTAA

>p2_ind4485

ATGCAAATAGACCCCGTTGCATATCATTGTTCCGAGTTGAAGTTCGTGCTTGTACCCGAAGAGTCGGGAAAGTTAT
CCCCGACGGAAATAGGATTCCATTTTCAGATTGGGTGCCGCGTACATGACAGAAGACCCCGCTCTGAGTCAGTGA

TGACGCACCAACGCAGATATCGATGTCTCACGTCTACCTTATTACTTGCCTTGATCGTCGGCACCCGATATTTGTCA
ACTGGGAATCCTCCTGGACCTATTATCGGTCCTTTGAAACTAACAGCAATTACCCCAGAAAGTGTCTGCTAA

>p2_ind1795

ATGCAAATAGACCCCGTTGCATATCATTGTTCCGAGTTGAAGTTCGTGCTTGTACCCGAAGAGTCGGGAAAGTTAT
CCCCGACGGAAATAGGATTCCATTTTCAGATTGGGTGCCGCGTACATGACAGAAGACCCCGCTCTGAGTCAGTGA
TGACGCACCAACGCAGATATCGATGTCTCACGTCTACCTTATTACTTGCCTTGATCGTCGGCACCCGATATTTGTCA
ACTGGGAATCCTCCTGGACCTATTATCGGTCCTTTGAAACTAACAGCAATTACCCCAGAAAGTGTCTGCTAA

>p2_ind1398

ATGCAAATAGACCCCGTTGCATATCATTGTTCCGAGTTGAAGTTCGTGCTTGTACCCGAAGAGTCGGGAAAGTTAT
CCCCGACGGAAATAGGATTCCATTTTCAGATTGGGCGCCGCGTACATGACAGAAGACCCCGCTCTGAGTCAGTGA
TGACGCACCAACGCAGATATCGATGTCTCACGTCTACCTTATTACTTGCCTTGATCGTCGGCACCCGATATTTGTCA
ACTGGGAATCCTCCTGGACCTATTATCGGTCCTTTGAAACTAACAGCAATTACCCCTGAAAGTGTCTGCTAA

>p2_ind797

ATGCAAATAGACCCCGTTGCATATCATTGTTCCGAGTAGAAGTTCGTGCTTGTACCCGAAGAGTCGGGAAAGTTAT
CCCCGACGGAAATAGGATTCCATTTTCAGACTGGGCGCCGCGTACATGACAGAAGACCCCGCTCTGAGTCAGTGA
TGACGCACCAACGCAGATATCGATGTCTCACGTCTACCTTATTACTTGCCTTGATCGTCGGCACCCGATATTTGTCA
ACTGGGAATCCTCCTGGACCTATTATCGGTCCTTTGAAACTAACAGCAATTACCCCAGAAAGTGTCTGCTAA

>p2_ind3356

ATGCAAATAGACCCCGTTGCATATCATTGTTCCGAGTTGAAGTTCGTGCTTGTACCCGAAGAGTCGGGAAAGTTAT
CCCCGACGGAAATAGGATTCCATTTTCAGATTGGGTGCCGCGTACATGACAGAAGACCCCGCTCTGAGTCAGTGA
TGACGCACCAACGCAGATATCGATGTCTCACGTCTACCTTATTACTTGCCTTGATCGTCGGCACCCGATATTTGTCA
ACTGGGAATCCTCCTGGACCTATTATCGGTCCTTTGAAACTAACAGGAATTACCCCAGAAAGTGTCTGCTAA

>p2_ind2512

ATGCAAATAGACCCCGTTGCATATCATTGTTCCGAGTTGAAGTTCGTGCTTGTACCCGAAGAGTCGGGAAAGTTAT
CCCCGACGGAAATAGGATTCCATTTTCAGATTGGGTGCCGCGTACATGACAGAAGACCCCGCTCTGAGTCAGTGA
TGACGCACCAACGCAGATATCGATGTCTCACGTCTACCTTATTACTTGCCTTGATCGTCGGCACCCGATATTTGTCA
ACTGGGAATCCTCCTGGACCTATTATCGGTCCTTTGAAACTAACAGCAATTACCCCAGAAAGTGTCTGCTAA

>p2_ind487

ATGCAAATAGACCCCGTTGCATATCATTGTTCCGAGTTGAAGTTCGTGCTTGTACCCGAAGAGTCGGGAAAGTTAT
CCCCGACGGAAATAGGATTCCATTTTCAGACTGGGCGCCGCGTACATGACAGAAGACCCCGCTCTGAGTCAGTGA
TGACGCACCAACGCAGACATCGATGTCTCACGTCTACCTTATTACTTGCCTTGATCGTCGGCACCCGATATTTGTCA
ACTGGGAATCCTCCTGGACCTATTATCGGTCCTTTGAAACTAACAGCAATTACCCCAGAAAGTGTCTGCTAA

>p2_ind1497

ATGCAAATAGACCCCGTTGCATATCATTGTTCCGAGTTGAAGTTCGTGCTTGTACCCGAAGAGTCGGGAAAGTTAT
CCCCGACGGAAATAGGATTCCATTTTCAGATTGGGTGCCGCGTACATGACAGAAGACCCCGCTCTGAGTCAGTGA
TGACGCACCAACGCAGACATCGATGTCTCACGTCTACCTTATTACTTGCCTTGATCGTCGGCACCCGATATTTGTCA
ACTGGGAATCCTCCTGGACCTATTATCGGTCCTTTGAAACTAACAGCAATTACCCCAGAAAGTGTCTGCTAA

>p2_ind4241

ATGCAAATAGACCCCGTTGCATATCATTGTTCCGAGTTGAAGTTCGTGCTTGTACCCGAAGAGTCGGGAAAGTTAT
CCCCGACGGAAATAGGATTCCATTTTCAGATTGGGTGCCGCGTACATGACAGAAGACCCCGCTCTGAGTCAGTGA
TGGCGCACCAACGCAAATATCGATGTCTCACGTCTACCTTATTACTTGCCTTGATCGTCGGCACCCGATATTTGTCA
ACTGGGAATCCTCCTGGACCTATTATCGGTCCTTTGAAACTAACAGCAATTACCCCAGAAAGTGTCTGCTAA

>p2_ind3869

ATGCAAATAGACCCCGTTGCATATCATTGTTCCGAGTTGAAGTTCGTGCTTGTACCCGAAGAGTCGGGAAAGTTAT
CCCCGACGGAAATAGGATTCCATTTTCAGATTGGGCGCCGCGTACATGACAGAAGACCCCGCTCTGAGTCAGTGA
TGACGCACCAACGCAGATATCGATGTCTCACGTCTACCTTATTACTTGCCTTGATCGTCGGCACCCGATATTTGTCA
ACTGGGAATCCTCCTGGACCTATTATCGGTCCTTTGAAACTAACAGCAATTACCCCAGAAAGTGTCTGCTAA

>p2_ind4823

ATGCAAATAGACCCCGTTGCATATCATTGTTCCAAGTTGAAGTTCGTGCTTGTACCCGAAGAGTCGGGAAAGTTAT
CCCCGACGGAAATAGGATTCCATTTTCAGATTGGGTGCCGCGTACATGACAGAAGACCCCGCTCTGAGTCAGTGA
TGACGCACCAACGCAGATATCGATGTCTCACGTCTACCTTATTACTTGCCTTGATCGTCGGCACCCGATTTTTGTCA
ACTGGGAATCCTCCTGGACCTATTATCGGTCCTTTGAAACTAACAGCAATTACCCCAGAAAGTGTCTGCTAA

>p2_ind2741

ATGCAAATAGACCCCGTTGCATATCATTGTTCCGAGTTGAGGTTCGTGCTTGTACCCGAAGAGTCGGGAAAGTTAT
CCCCGACGGAAATAGGATTCCATTTTCAGATTGGGTGCCGCGTACATGACAGAAGACCCCGCTCTGAGTCAGTGA
TGACGCACCAACGCAGATATCGATGTCTCACGTCTACCTTATTACTTGCCTTGATCGTCGGCACCCGATATTTGTCA
ACTGGGAATCCTCCTGGACCTATTATCGGTCCTTTGAAACTAACAGCAATTACGCCAGAAAGTGTCTGCTAA

>p2_ind3626

ATGCAAATAGACCCCGTTGCATATCATTGTTCCGAGTTGAAGTTCGTGCTTGTACCCGAAGAGTCGGGAAAGTTAT
CCCCGACGGAAATAGGATTCCATTTTCAGATTGGGTGCCGCGTACATGACAGAAGACCCCGCTCTGAGTCAGTGA
TGACGCACCAACGCAGATATCGATGTCTCACGTCTACCTTATTACTTGCCTTGATCGTCGGCACCCGATATTTGTCA
ACTGGGAATCCTCCTGGACCTATTATCGGTCCTTTGAAACTAACGGCAATTACCCCAGAAAGTGTCTGCTAA

>p2_ind1866

ATGCAAATAGACCCCGTTGCATATCATTGTTCCGAGCTGAAGTTCGTGCTTGTACCCGAAGAGTCGGGAAAGTTAT
CCCCGACGGAAATAGGATTCCATTTTCAGATTGGGTGCCGCGTACATGACAGAAGACCCCGCTCTGAGTCAGTGA

TGACGCACCAACGCAGATATCGATGTCTCACGTCTACCTTATTACTTGCCTTGATCGTCGGCACCCGATATTTGTCA
ACTGGGAATCCTCCTGGACCTATTATCGGTCCTTTGAAACTAACAGGAATTACCCCAGAAAGTGTCTGCTAA

>p2_ind4456

ATGCAAATAGACCCCGTTGCATATCATTGTTCCGAGTTGAAGTTCGTGCTTGTACCCGAAGAGTCGGGAAAGTTAT
CCCCGACGGAAATAGGATTCCATTTTCAGACTGGGCGCCGCGTACATGACAGAAGACCCCGCTCTGAGTCAGTGA
TGACGCACCAACGCAGATATCGATGTCTCACGTCTACCTTATTACTTGCCTTGATCGTCGGCACCCGATATTTGTCA
ACTGGGAATCCTCCTGGAGCTATTATCGGTCCTTTGAAACTAACAGCAATTACCCCAGAAAGTGTCTGCTAA

>p2_ind1685

ATGCAAATAGACCCCGTTGCATATCATTGTTCCGAGTTGAAGTTCGTGCTTGTACCCGAAGAGTCGGGAAAGTTAT
CCCCGACGGAAATAGGATTCCATTTTCAGATTGGGTGCCGCGTACATGACAGAAGACCCCGCTCTGAGTCAGTGA
TGACGCACCAACGCAGACATCGATGTCTCACGTCTACCTTATTACTTGCCTTGATCGTCGGCACCCGATATTTGTCA
ACTGGGAATCCTCCTGGACCTATTATCGGTCCTTTGAAACTAACAGCAATTACCCCAGAAAGTGTCTGCTAA

>p2_ind4876

ATGCAAATAGACCCCGTTGCATATCATTGTTCCGAGTTGAAGTTCGTGCTTGTACCCGAAGAGTCGGGAAAGTTAT
CCCCGACGGAAATAGGATTCCATTTTCAGACTGGACGCCGCGTACATGACAGAAGACCCCGCTCTGAGTCAGTGA
TGACGCACCAACGCAGATATCGATGTCTCACGTCTACCTTATTACTTGCCTTGATCGTCGGCACCCGATATTTGTCA
ACTGGGAATCCTCCTGGACCTATTATCGGTCCTTTGAAACTAACAGCAATTACCCCAGAAAGTGTCTGCTAA

>p2_ind2198

ATGCAAATAGACCCCGTTGCATATCATTGATCCGAGTTGAAGTTCGTGCTTGTACCCGAAGAGTCGGGAAAGTTAT
CCCCGACGGAAATAGGATTCCATTTTCAGATTGGGTGCCGCGTACATGACAGAAGACCCCGCTCTGAGTCAGTGA
TGACGCACCAACGCAGATATCGATGTCTCACGTCTACCTTATTACTTGCCTTGATCGTCGGCACCCGATATTTGTCA
ACTGGGAATCCTCCTGGACCTATTATCGGTCCTTTGAAACTAACAGCAATTACCCCAGAAAGTGTCTGCTAA

>p2_ind4057

ATGCAAATAGACCCCGTTGCATATCATTGTTCCGAGTTGAAGTTCGTGCTTGTACCCGAAGAGTCGGGAAAGTTAT
CCCCGACGGAAATAGGATTCCATTTTCAGATTGGGTGCCGCGTACATGACAGAAGACCCCGCTCTGAGTCAGTGA
TGTCGCACCAACGCAGATATCGATGTCTCACGTCTACCTTATTACTTGCCTTGATCGTCGGCACCCGATATTTGTCA
ACTGGGAATCCTCCTGGACCTATTATCGGTCCTTTGAAACTAACAGCAATTACCCCAGAAAGTGTCTGCTAA

>p2_ind579

ATGCAAATAGACCCCGTTGCATATCATTGTTCCGAGTTGAAGTTCGTGCTTGTACCCGAAGAGTCGGGAAAGTTAT
CCCCGACGGAAATAGGATTCCATTTTCAGATTGGGCGCCGCGTACATGACAGAAGACCCCGCTCTGAGTCAGTGA
TGACGCACCAACGCAGATATCGATGTCTCACGTCTACCTTATTACTTGCCTTGATCGTCGGCACCCGATATTTGTCA
ACTGGGAATCCTCCTGGACCTATTATCGGTCCTTTGAAACTAACAGCAATTACCCCAGAAAGTGTCTGCTAA

>p2_ind4221

ATGCAAATAGACCCCGTTGCATATCATTGTTCCGAGTTGAAGTTCGTGCTTGTACCCGAAGAGTCGGGAAAGTTAT
CCCCGACGGAAATAGGATTCCATTTTCAGATTGGGTGCCGCGTACATGACAGAAGACCCCGCTCTGAGTCAGTGA
TGACGCCCCAACGCAGATATCGATGTCTCACGTCTACCTTATTAATTGCCTTGATCGTCGGCACCCGATATTTGTCA
ACTGGGAATCCTCCTGGACCTATTATCGGTCCTTTGAAACTAACAGCAATTACCCCAGAAAGTGTCTGCTAA

>p2_ind3042

ATGCAAATAGACCCCGTTGCATATCATTGTTCCGAGTTGAAGTTCGTGCTTGTACCCGAAGAGTCGGGAAAGTTAT
CCCCGACGGAAATAGGATTCCATTTTCAGATTGGGTGCCGCGTACATGACAGAAGACCCCGCTCTGAGTCAGTGA
TGACGCACCAACGCAGATATCGATGTCTCACGTCTACCTTATTACTTGCCTTGATCGTCGGCACCCGATATTTGTCA
ACTGGGAATCCTCCTGGACCTATTATCGGTCCTTTGAAACTAACAGCAATTACCCCATAAAGTGTCTGCTAA

>p2_ind3048

ATGCAAATAGACCCCGTTGCATATCATTGTTCCGAGTTGAAGTTCGTGCTTGTACCCGAAGAGTCGGGAAAGTTAT
CCCCGACGGAAATAGGATTCCATTTTCAGATTGGGTGCCGCGTACATGACAGAAGACCCCGCTCTGAGTCAGTGA
TGACGCACCAACGCAGATATCGATGTCTCACGTCTACCTTATTACTTGCCTTGATCGTCGGCACCCGATATTTGTCA
ACTGGGAATCCTCCTGGACCTATTATCGGTCCTTTGAAACTAACGGCAATTACCCCAGAAAGTGTCTGCTAA

>p2_ind1201

ATGCAAATAGACCCCGTTGCATATCATTGTTCCGAGTTGAAGTTCGTGCTTGTACCCGAAGAGTCGGGAAAGTTAT
CCCCGACGGAAATAGGATTCCATTTTCAGATTGGGTGCCGCGTACATGACAGAAGACCCCGCTCTGAGTCAGTGA
TGACGCACCAACGCAGACATCGATGTCTCACGTCTACCTTATTACTTGCCTTGATCGTCGGCACCCGATATTTGTCA
ACTGGGAATCCTCCTGGACCTATTATCGGTCCTTTGAAACTAACAGCAATTACCCCAGAAAGTGTCTGCTAA

>p2_ind3999

ATGCAAATAGACCCCGTTGCATATCATTGTTCCGAGTTGAAGTTCGTGCTTGTACCCGAAGAGTCGGGAAAGTTAT
CCCCGACGGAAATAGGATTCCATTTTCAGATTGGGTGCCGCGTACATGACAGAAGACCCCGCTCTGAGTCAGTGA
TGACGCACCAACGCAGACATCGATGTCTCACGTCTACCTTATTACTTGCCTTGATCGTCGGCACCCGATATTTGTCA
ACTGGGAATCCTCCTGGACCTATTATCGGTCCTTTGAAACTAACAGCAATTACCCCAGAAAGTGTCTGCTAA

>p2_ind2159

ATGCAAATAGACCCCGTTGCATATCATTGATCCGAGTTGAAGTTCGTGCTTGTACCCGAAGAGTCGGGAAAGTTAT
CCCCGACGGAAATAGGATTCCATTTTCAGATTGGGTGCCGCGTACATGACAGAAGACCCCGTTCTGAGTCAGTGAT
GACGCACCAACGCAGATATCGATGTCTCACGTCTACCTTATTACTTGCCTTGATCGTCGGCACCCGATATTTGTCAA
CTGGGAATCCTCCTGGACCTATTATCGGTCCTTTGAAACTAACAGCAATTACCCCAGAAAGTGTCTGCTAA

>p2_ind4437

ATGCAAATAGACCCCGTTGCATATCATTGTTCCAAGTTGAAGTTCGTGCTTGTACCCGAAGAGTCGGGAAAGTTAT
CCCCGACGGAAATAGGATTCCATTTTCAGATTGGGTGCCGCGTACATGACAGAAGACCCCGCTCTGAGTCAGTGA

TGACGCACCAACGCAGATATCGATGTCTCACGTCTACCTTATTACTTGCCTTGATCGTCGGCACCCGATATTTGTCA
ACTGGGAATCCTCCTGGACCTATTATCGGTCCTTTGAAACTAACAGCAATTACCCCAGAAAGTGTCTGCTAA

>p2_ind890

ATGCAAATAGACCCCGTTGCATATCATTGTTCCGAGTTGAAGTTCGTGCTTGTACCCGAAGAGTCGGGAAAGTTAT
CCCCGACGGAAATAGGATTCCATTTTCAGATTGGGTGCCGCGTACATGACAGAAGACCCCGTTCTGAGTCAGTGAT
GACGCACCAACGCAGATATCGATGTCTCACGTCTACCTTATTACTTGCCTTGATCGTCGGCACCCGATATTTGTCAA
CTGGGAATCCTCCTGGACCTATTATCGGTCCTTTGAAACTAACAGGAATTACCCCAGAAAGTGTCTGCTAA

>p2_ind2573

ATGCAAATAGACCCCGTTGCATATCATTGTTCCGAGTTGAAGTTCGTGCTTGTACCCGAAGAGTCGGGAAAGTTAT
CCCCGACGGAAATAGGATTACATTTTCAGATTGGGCGCCGCGTACATGACAGAAGACCCCGCTCTGAGTCAGTGA
TGACGCACCAACGCAGATATCGATGTCTCACGTCTACCTTATTACTTGCCTTGATCGTCGGCACCCGATATTTGTCA
ACTGGGAATCCTCCTGGACCTATTATCGGTCCTTTGAAACTAACAGCAATTACCCCAGAAAGTGTCTGCTAA

>p2_ind2710

ATGCAAATAGACCCCGTTGCATATCATTGTTCCGAGTTGAAGTTCGTGCTTGTACCCGAAGAGTCGGGAAAGTTAT
CCCCGACGGAAATAGGATTCCATTTTCAGATTGGGTGCCGCGTACATGACAGAAGACCCCGCTCTGAGTCAGTGA
TGACGCACCAACGCAGATATCGATGTCTCACGTCTACCTTATTACTTGCCTTGATCGTCGGCACCCGATATTTGTCA
ACTGGGAATCCTCCTGGACCTATTATCGGTCCTTTGAAACTAACAGCAATTACCCCAGAAAGTGTCTGCTAA

>p2_ind3796

ATGCAAATAGACCCCGTTGCATATCATTGTTCCGAGTTGAAGTTCGTGCTTGTACCCGAAGAGTCGGGAAAGTTAT
CCCCGACGGAAATAGGATTCCATTTTCAGATTGGGTGCCGCGTACATGACAGAAGACCCCGCTCTGAGTCAGTGA
TGACGCACCAACGCAGATATCGATGTCTCACGTCTACCTTATTACTTGCCTTGATCGTCGGCACCCGATATTTGTCA
ACTGGGAATCCTCCTGGACCTATTATCGGTCCTTTGAAACTAACAGCAATTACCCCAGAAAGTGTCTGCTAA

>p2_ind3965

ATGCAAATAGACCCCGTTGCATATCATTGTTCCGAGTTGAAGTTCGTGCTTGTACCCGAAGAGTCGGGAAAGTTAT
CCCCGACGGAAATAGGATTCCATTTTCAGATTGGGTGCCGCGTACATGACAGAAGACCCCGCTCTGAGTCAGTGA
TGACGCACCAACGCAGATATCGATGTCTCACGTCTACCTTATTACTTGCCTTGATCGTCGGCACCCGATATTTGTCA
ACTGGGAATCCTCCTGGACCTATTATCGGTCCTTTGAAACTAACAGCAATTACCCCAGAAAGTGTCTGCTAA

>p2_ind4460

ATGCAAATAGACCCCGTTGCATATCATTGTTCCGAGTTGAAGTTCGTGCTTGTACCCGAAGAGTCGGGAAAGTTAT
CCCCGACGGAAATAGGATTCCATTTTCAGATTGGGTGCCGCGTACATGACAGAAGACCCCGCTCTGAGTCAGTGA
TGACGCACCAACGCAGATATCGATGTCTCACGTCTACCTTATTACTTGCCTTGATCGTCGGCACCCGATATTTGTCA
ACTGGGAATCCTCCTGGACCTATTATCGGTCCTTTGAAACTAACAGCAATTACCCCAGAAAGTGTCTGCTAA

>p2_ind2929

ATGCAAATAGACCCCGTTGCATATCATTGTTCCGAGTTGAAGTTCGTGCTTGTACCCGAAGAGTCGGGAAAGTTAT
CCCCGACGGAAATAGGATTCCATTTTCAGATTGGGTGCCGCGTACATGACAGAAGACCCCGCTCTGAGTCAGTGA
TGACGCACCAACGCAGATATCGATGTCTCACGTCAACCTTATTACTTGCCTTGATCGTCGGCACCCGATATTTGTCA
ACTGGGAATCCTCCTGGACCTATTATCGGTCCTTTGAAACTAACAGCAATTACCCCAGAAAGTGTCTGCTAA

>p2_ind4954

ATGCAAATAGACCCCGTTGCATATCATTGTTCCGAGTTGAAGTTCGTGCTTGTACCCGAAGAGTCGGGAAAGTTAT
CCCCGACGGAAATAGGATTCCATTTTCAGATTGGGTGCCGCGTACATGACAGAAGACCCCGCTCTGAGTCAGTGA
TGTCGCACCAACGCAGATATCGATGTCTCACGTCTACCTTATTACTTGCCTTGATCGTCGGCACCCGATATTTGTCA
ACTGGGAATCCTCCTGGACCTATTATCGGTCCTTTGAAACTAACAGCAATTACCCCAGAAAGTGTCTGCTAA

>p2_ind2026

ATGCAAATAGACCCCGTTGCATATCATTGTTCCGAGTTGAAGTTCGTGCTTGTACCCGAAGAGTCGGGAAAGTTAT
CCCCGACGGAAATAGGATTCCATTTTCAGATTGGGTGCCGCGTACATGACAGAAGACCCCGCTCTGAGTCAGTGA
TGACGCACCAACGCAGATATCGATGTCTCACGTCTACCTTATTACTTGCCTTGATCGTCGGCACCCGATATTTGTCA
ACTGGGAATCCTCCTGGACCTATTATCGGTCCTTTGAAACTAACAGCAATTACCCCAGAAAGTGTCTGCTAA

>p2_ind3415

ATGCAAATAGACCCCGTTGCATATCATTGTTCCGAGTTGAGGTTCGTGCTTGTACCCGAAGAGTCGGGAAAGTTAT
CCCCGACGGAAATAGGATTCCATTTTCAGATTGGGTGCCGCGTACATGACAGAAGACCCCGCTCTGAGTCAGTTAT
GACGCACCAACGCAGATATCGATGTCTCACGTCTACCTTATTACTTGCCTTGACCGTCGGCACCCGATATTTGTCAA
CTGGGAATCCTCCTAGACCTATTATCGGTCCTTTGAAACTAACAGCAATTACCCCAGAAAGTGTCTGCTAA

>p2_ind1298

ATGCAAATAGACCCCGTTGCATATCATTGTTCCGAGTTGAAGTTCGTGCTTGTACCCGAAGAGTCGGGAAAGTTAT
CCCCGACGGAAATAGGATTCCATTTTCAGATTGGGTGCCGCGTACATGACAGAAGACCCCGCTCTGTGTCAGTGAT
GACGCACCTACGCAGATATCGATGTCTCAAGTCTACCTTATTACTTGCCTTGATCGTCGGCACCCGATATTTGTCAA
CTGGGAATCCTCCTGGACCTATTATCGGTCCTTTGAAACTAACAGCAATTACCCCAGAAAGTGTCTGCTAA

>p2_ind1994

ATGCAAATAGACCCCGTTGCATATCATCGTTCCGAGTTGAAGTTCGTGCTTGTACCCGAAGAGTCGGGAAAGTTAT
CCCCGACGGAAATAGGATTCCATTTTCAGATTGGGCGCCGCGTACATGACAGAAGACCCCGCTCTGAGTCAGTGA
TGACGCACCAACGCAGATATCGATGTCTCACGTCTACCTTATTACTTGCCTTGATCGTCGGCACCCGATATTTGTCA
ACTGGGAATCCTCCTGGACCTATTATCGGTCCTTTGAAACTAACAGCAATTACCCCAGAAAGTGTCTGCTAA

>p2_ind1087

ATGCAAATAGACCCCGTTGCATATCATTGTTCCGAGTTGAAGTTCGTGCTTGTACCCGAAGAGTCGGGAAAGTTAT
CCCCGACGGAAATAGGATTCCATTTTCAGATTGGGTGCCGCGTACATGACAGAAGACCCCGCTCTGAGTCAGTGA

TGACGCACCAACGCAGATATCGATGTCTCACGTCTACCTTATTACTTGCCTTGATCGTCGGCACCCGATATTTGTCA
ACTGGGAATCCTCCTGGACCTATTATCGGTCCTTTGAAACTAACAGCAATTACCCCAGAAAGTGTCTGCTAA

>p2_ind3970

ATGCAAATAGACCCCGTTGCATATCATTGTTCCAAGTTGAAGTTCGTGCTTGTACCCGAAGAGTCGGGAAAGTTAT
CCCCGACGGAAATAGGATTCCATTTTCAGATTGGGTGCCGCGTACATGACAGAAGACCCCGCTCTGAGTCAGTGA
TGACGCACCAACGCAGATATCGATGTCTCACGTCTACCTTATTACTTGCCTTGATCGTCGGCACCCGATATTTGTCA
ACTGGGAATCCTCCTGGACCTATTATCGGTCCTTTGAAACTAACAGCAATTACCCCAGAAAGTGTCTGCTAA

>p2_ind1222

ATGCAAATAGACCCCGTTGCATATCATTGTTCCGAGTTGAAGTTCGTGCTTGTACCCGAAGAGTCGGGAAAGTTAT
CCCCGACGGAAATAGGATTCCATTTTCAGATTGGGTGCCGCGTACATGACAGAAGACCCCGCTCTGAGTCAGTGA
TGACGCACCAACGCAGATATCGATGTCTCACGTCTACCTTATTACTTGCCTTGATCGTCGGCACCCGATATTTGTCA
ACTGGGAATCCTCCTGGACCTATTATCGGTCCTTTGAAACTAACAGCAATTAGCCCAGAAAGTGTCTGCTAA

>p2_ind2007

ATGCAAATAGACCCCGTTGCATATCATTGTTCCGAGTAGAAGTTCGTGCTTGTACCCGAAGAGTCGGGAAAGTTAT
CCCCGACGGAAATAGGATTCCATTTTCAGATTGGGTGCCGCGTACATGACAGAAGACCCCGCTCTGAGTCAGTGA
TGACGCACCAACGCAGATATCGATGTCTCACGTCTACCTTATTACTTGCCTTGATCGTCGGCACCCGATATTTGTCA
ACTGGGAATCCTCCTGGACCTATTATCGGTCCTTTGAAACTAACAGCAATTACCCCAGAAAGTGTCTGCTAA

>p2_ind4828

ATGCAAATAGACCCCGTTGCATATCATTGTTCCGAGTTGAAGTTCGTGCTTGTACCCGAAGAGTCGGGAAAGTTAT
CCCCGACGGAAATAGGATTCCATTTTCAGATTGGGTGCCGCGTACATGACAGAAGACCCCGCTCTGAGTCAGTGA
TGACGCACCAACGCAGATATCGATGTCTCACGTCTACCTTATTACTTGCCTTGATCGTCGGCACCCGATATTTGTCA
ACTGGGAATCCTCCTGGACCTATTATCGGTCCTTTGAAACTAACAGCAATTACCCCAGAAAGTGTCTGCTAA

>p2_ind2208

ATGCAAATAGACCCCGTTGCATATCATTGTTCCGAGTTGAAGTTCGTGCTTGTACCCGAAGAGTCGGGAAAGTTAT
CCCCGACGGAAATAGGATTCCATTTTCAGACTGGGCGCCGCGTACATGACAGAAGACCCCGCTCTGAGTCAGTGA
TGACGCACCAACGCAGACATCGATGTCTCACGTCTACCTTATTACTTGCCTTGATCGTCGGCACCCGATATTTGTCA
ACTGGGAATCCTCCTGGACCTATTATCGGTCCTTTGAAACTAACAGCAATTACCCCAGAAAGTGTCTGCTAA

>p2_ind3711

ATGCAAATAGACCCCGTTGCATATCATTGTTCCGAGTTGAAGTTCGTGCTTGTATCCGAAGAGTCGGGAAAGTTAT
CCCCGACGGAAATAGGATTCCATTTTCAGATTGGGTGCCGCGTACATGACAGAAGACCCCGCTCTGAGTCAGTGA
TGACGCACCAACGCAGATATCGATGTCTCACGTCTACCTTATTACTTGCCTTGATCGTCGGCACCCGATATTTGTCA
ACTGGGAATCCTCCTGGACCTATTATCGGTCCTTTGAAACTAACAGCAATTACCCCAGAAACTGTCTGCTAA

>p2_ind1194

ATGCAAATAGACCCCGTTGCATATCATTGTTCCGAGTTGAAGTTCGTGCTTGTACCCGAAGAGTCGGGAAAGTTAT
CCCCGACGGAAATAGGATTCCATTTTCAGATTGGGTGCCGCGTACATGACAGAAGACCCCGCTCTGAGTCAGTGA
TGACGCACCAACGCAGATATCGATGTCTCACGTCTACCTTATTACTTGCCTTGATCGTCGGCACCCGATATTTGTCA
ACTGGGAATCCTCCTGGACCTATTATCGGTCCTTTGAAACTAACAGCAATTACCCCAGAAAGTGTCTGCTAA

>p2_ind4296

ATGCAAATAGACCCCGTTGCATATCATTGTTCCGAGTTGAAGTTCGTGCTTGTACCCGAAGAGTCGGGAAAGTTAT
CCCCGACGGAAATAGGATTCCATTTTCAGATTGGGTGCCGCGTACATGACAGAAGACCCCGCTCTGAGTCAGTGA
TGACGCACCAACGCAGATATCGATGTCTCACGTCTACCTTATTACTTGCCTTGATCGTCGGCACCCGATATTTGTCA
ACTGGGAATCCTCCTGGACCTATTATCGGTCCTTTGAAACTAACAGCAATTACCCCAGAAAGTGTCTGCTAA

>p2_ind1067

ATGCAAATAGACCCCGTTGCATATCATTGTTCCGAGTTGAAGTTCGTGCTTGTACCCGAAGAGTCGGGAAAGTTAT
CCCCGACGGAAATAGGATTCCATTTTCAGATTGGGTGCCGCGTACATGACAGAAGACCCCGCTCTGAGTCAGTGA
TGACGCACCTACGCAGATATCGATGTCTCAAGTCTACCTTATTACTTGCCTTGATCGTCGGCACCCGATATTTGTCA
ACTGGGAATCCTCCTGGACCTATTATCGGTCCTTTGAAACTAACAGCAATTACCCCAGAAAGTGTCTGCTAA

>p2_ind2816

ATGCAAATAGACCCCGTTGCATATCATTGTTCCGAGTTGAAGTTCGTGCTTGTACCCGAAGAGTCGGGAAAGTTAT
CCCCGACGGAAATAGGATTACATTTTCAGATTGGGCGCCGCGTACATGACAGAAGACCCCGCTCTGAGTCAGTGA
TGACGCACCAACGCAGATATCGATGTCTCACGTCTACCTTATTACTTGCCTTGATCGTCGGCACCCGATATTTGTCA
ACTGGGAATCCTCCTGGACCTATTATCGGTCCTTTGAAACTAACAGCAATTACCCCAGAAAGTGTCTGCTAA

>p2_ind4757

ATGCAAATAGACCCCGTTGCATATCATTGTTCCGAGTTGAAGTTCGTGCTTGTACCCGAAGAGTCGGGAAAGTTAT
CCCCGACGGAAATAGGATTCCATTTTCAGATTGGGTGCCGCGTACATGACAGAAGACCCCGCTCTGAGTCAGTGA
TGACGCACCAACGCAGACATCGATGTCTCACGTCTACCTTATTACTTGCCTTGATCGTCGGCACCCGATATTTGTCA
ACTGGGAATCCTCCTGGACCTATTATCGGTCCTTTGAAACTAACAGCAATTACCCCAGAAAGTGTCTGCTAA

>p2_ind1865

ATGCAAATAGACCCCGTTGCATATCATTGTTCCGAGTTGAAGTTCGTGCTTGTACCCGAAGAGTCGGGAAAGTTAT
CCCCGACGGAAATAGGATTCCATTTTCAGATTGGGCGCCGCGTACATGACAGAAGACCCCGCTCTGAGTCAGTGA
TGACGCACCAACGTAGATATCGATGTCTCACGTCTACCTTATTACTTGCCTTGATCGTCGGCACCCGATATTTGTCA
ACTGGGAATCCTCCTGGACCTATTATCGGTCCTTTGAAACTAACAGCAATTACCCCAGAAAGTGTCTGCTAA

>p2_ind1147

ATGCAAATAGACCCCGTTGCATATCATTGTTCCGAGTTGAAGTTCGTGCTTGTACCCGAAGAGTCGGGAAAGTTAT
CCCCGACGGAAATAGGATTCCATTTTCAGATTGGGTGCCGCGTACATGACAGAAGACCCCGCTCTGAGTCAGTGA

TGACGCACCAACGCAGATATCGATGTCTCACGTCAACCTTATTACTTGCCTTGATCGTCGGCACCCGATATTTGTCA
ACTGGGAATCCTCCTGGACCTATTATCGGTCCTTTGAAACTAACAGCAATTACCCCAGAAAGTGTCTGCTAA

>p2_ind4115

ATGCAAATAGACCCCGTTGCATATCATTGATCCGAGTTGAAGTTCGTGCTTGTACCCGAAGAGTCGGGAAAGTTAT
CCCCGACGGAAATAGGATTCCATTTTCAGATTGGGTGCCGCGTACATGACAGAAGACCCCGCTCTGAGTCAGTGA
TGACGCACCAACGCAGATATCGATGTCTCACGTCTACCTTATTACTTGCCTTGATCGTCGGCACCCGATATTTGTCA
ACTGGGAATCCTCCTGGACCTATTATCGGTCCTTTGAAACTAACAGCAATTACCCCAGAAAGTGTCTGCTAA

>p2_ind2393

ATGCAAATAGACCCCGTTGCATATCATTGTTCCGAGTTGAAGTTCGTGCTTGTACCCGAAGAGTCGGGAAAGTTAT
CCCCGACGGAAATAGGATTCCATTTTCAGATTGGGTGCCGCGTACATGACAGAAGACCCCGCTCTGAGTCAGTGA
TGACGCACCAACGCAGATATCGATGTCTCACGTCTACCTTATTACTTGCCTTGATCGTCGGCACCCGATATTTGTCA
ACTGGGAATCCTCCTGGACCTATTATCGGTCCTTTGAAACTAACAGCAATTACCCCAGAAAGTGTCTGCTAA

>p2_ind3168

ATGCAAATAGACCCCGTTGCATATCATTGATCCGAGTTGAAGTTCGTGCTTGTACCCGAAGAGTCGGGAAAGTTAT
CCCCGACGGAAATAGGATTCCATTTTCAGATTGGGTGCCGCGTACATGACAGAAGACCCCGTTCTGAGTCAGTGAT
GACGCACCAACGCAGATATCGATGTCTCACGTCTACCTTATTACTTGCCTTGATCGTCGGCACCCGATATTTGTCAA
CTGGGAATCCTCCTGGACCTATTATCGGTCCTTTGAAACTAACAGCAATTACCCCAGAAAGTGTCTGCTAA

>p2_ind3285

ATGCAAATAGACCCCGTTGCATATCATTGATCCGAGTTGAAGTTCGTGCTTGTACCCGAAGAGTCGGGAAAGTTAT
CCCCGACGGAAATAGGATTCCATTTTCAGATTGGGTGCCGCGTACATGACAGAAGACCCCGCTCTGAGTCAGTGA
TGACGCACCAACGCAGATATCGATGTCTCACGTCTACCTTATTACTTGCCTTGATCGTCGGCACCCGATATTTGTCA
ACTGGGAATCCTCCTGGACCTATTATCGGTCCTTTGAAACTAACAGCAATTACCCCAGAAAGTGTCTGCTAA

>p2_ind3874

ATGCAAATAGACCCCGTTGCATATCATTGTTCCGAGTTGAAGTTCGTGCTTGTACCCGAAGAGTCGGGAAAGTTAT
CCCCGACGGAAATAGGATTCCATTTTCAGATTGGGTGCCGCGTACATGACAGAAGACCCCGCTCTGAGTCAGTGA
TGACGCACCAACGCAGATATCGATGTCTCACGTCTACCTTATTACTTGCCTTGATCGTCGGCACCCGATATTTGTCA
ACTGGGAATCCTCCTGGACCTATTATCGGTCCTTTGAAACTAACAGCAATTACCCCAGAAAGTGTCTGCTAA

>p2_ind1021

ATGCAAATAGACCCCGTTGCATATCATTGTTCCGAGTTGAAGTTCGTGCTTGTACCCGAAGAGTCGGGAAAGTTAT
CCCCGACGGAAATAGGATTCCATTTTCAGATTGGGTGCCGCGTACATGACAGAAGACCCCGCTCTGAGTCAGTGA
TGACGCACCAACGCAGACATCGATGTCTCACGTCTACCTTATTACTTGCCTTGATCGTCGGCACCCGATATTTGTCA
ACTGGGAATCCTCCTGGACCTATTATCGGTCCTTTGAAACTAACAGCAATTACCCCAGAAAGTGTCTGCTAA

>p2_ind3862

ATGCAAATAGACCCTGTTGCATATCATTGTTCCGAGTTGAAGTTCGTGCTTGTACCCGAAGAGTCGGGAAAGTTAT
CCCCGACGGAAATAGGATTCCATTTTCAGATTGGGTGCCGCGTACATGACAGAAGACCCCGCTCTGAGTCAGTGA
TGGCGCACCAACGCAAATACAGATGTCTCACGTCTACCTTAATACTTGCCTTGATCGTCGGCACCCGATATTTGTCA
ACTGGGAATCCTCCTGGACCTATTATCGGTCCTTTGAAACTAACAGCAATTACCCCAGAAAGTGTCTGCTAA

>p2_ind764

ATGCAAATAGACCCCGTTGCATATCATTGTTCCGAGTTGAAGTTCGTGCTTGTACCCGAAGAGTCGGGAAAGTTAT
CCCCGACGGAAATAGGATTCCATTTTCAGATTGGGTGCCGCGTACATGACAGAAGACCCCGCTCTGAGTCAGTGA
TGACGCACCAACGCAGATATCGATGTCTCACGTCTACCTTATTACTTGCCTTGATCGTCGGCACCCGATATTTGTCA
ACTGGGAATCCTCCTGGACCTATTATCGGTCCTTTGAAACTAACAGCAATTACCCCAGAAAGTGTCTGCTAA

>p2_ind4507

ATGCAAATAGACCCCGTTGCATATCATTGATCCGAGTTGAAGTTCGTGCTTGTACCCGAAGAGTCGGGAAAGTTAT
CCCCGACGGAAATAGGATTCCATTTTCAGATTGGGTGCCGCGTACATGACAGAAGACCCCGCTCTGAGTCAGTGA
TGACGCACCAACGCAGATATCGATGTCTCACGTCTACCTTATTACTTGCCTTGATCGTCGGCACCCGATATTTGTCA
ACTGGGAATCCTCCTGGACCTATTATCGGTCCTTTGAAACTAACAGCAATTACCCCAGAAAGTGTCTGCTAA

>p2_ind3938

ATGCAAATAGACCCCGTTGCATATCATTGTTCCGAGTTGAAGTTCGTGCTTGTACCCGAAGAGTCGGGAAAGTTAT
CCCCGACGGAAATAGGATTCCATTTTCAGATTGGGTGCCGCGTACATGACAGAAGACCCCGCTCTGAGTCAGTGA
TGACGCACCAACGCAGATATCGATGTCTCACGTCTACCTTATTACTTGCCTTGATCGTCGGCACCCGATATTTGTTA
ACTGGGAATCCTCCTGGACCTATTATCGGTCCTTTGAAACTAACAGCAATTACCCCATAAAGTGTCTGCTAA

>p2_ind1599

ATGCAAATAGACCCCGTTGCATATCATTGTTCCGAGTTGAAGTTCGTGCTTGTACCCGAAGAGTCGGGAAAGTTAT
CCCCGACGGAAATAGGATTCCATTTTCAGATTGGGTGCCGCGTACATGACAGAAGACCCCGCTCTGAGTCAGTGA
TGACGCACCAACGCAGATATCGATGTCTCACGTCTACCTTATTACTTGCCTTGATCGTCGGCACCCGATATTTGTCA
ACTGGGAATCCTCCTGGACCTATTATCGGTCCTTTGAAACTAACAGCAATTACCCCAGAAAGTGTCTGCTAA

>p2_ind2400

ATGCAAATAGACCCCGTTGCATATCATTGTTCCGAGTTGAAGTTCGTGCTTGTACCCGAAGAGTCGGGAAAGTTAT
CCCCGACGGAAATAGGATTCCATTTTCAGATTGGGTGCCGCGTACATGACAGAAGACCCCGCTCTGAGTCAGTGA
TGACGCTCCAACGCAGATATCGATGTCTCACGTCTACCTTATTACTTGCCTTGATCGTCGGCACCCGATATTTGTCA
ACTGGGAATCCTCCTGGACCTATTATCGGTCCTTTGAAACTAACAGCAATTACCCCAGAAAGTGTCTGCTAA

>p2_ind1421

ATGCAAATAGACCCCGTTGCATATCATTGTTCCGAGTAGAAGTTCGTGCTTGTACCCGAAGAGTCGGGAAAGTTAT
CCCCGACGGAAATAGGATTCCATTTTCAGACTGGGCGCCGCGTACATGACAGAAGACCCCGCTCTGAGTCAGTGA

TGACGCACCAACGCAGATATCGATGTCTCACGTCTACCTTATTACTTGCCTTGATCGTCGGCACCCGATATTTGTCA
ACTGGGAATCCTCCTGGACCTATTATCGGTCCTTTGAAACTAACAGCAATTACCCCAGAAAGTGTCTGCTAA

>p2_ind50

ATGCAAATAGACCCCGTTGCATATCATTGTTCCGAGTTGAAGTTCGTGCTTGTACCCGAAGAGTCGGGAAAGTTAT
CCCCGACGGAAATAGGATTCCATTTTGAGATTGGGTGCCGCGTACATGACAGAAGACCCCGCTCTGAGTCAGTGA
TGGCGCACCAACGCAAATATCGATGTCTCACGTCTACCTTATTACTTGCCTTGATCGTCGGCACCCGATATTTGTCA
ACTGGGAATCCTCCTGGACCTATTATCGGTCCTTTGAAACTAACAGCAATTACGCCAGAAAGTGTCTGCTAA

>p2_ind880

ATGCAAATAGACCCCGTTGCATATCATTGTTCCGAGTTGAAGTTCGTGCTTGTACCCGAAGAGTCGGGAAAGTTAT
CCCCGACGGAAATAGGATTCCATTTTCAGATTGGGTGCCGCGTACATGACAGAAGACCCCGCTCTGAGTCAGTGA
TGACGCACCAACGCAGATATCGATGTCTCACGTCTACCTTATTACTTGCCTTGATCGTCGGCACCCGATATTTGTCA
ACTGGGAATCCTCCTGGACCTATTATCGGTCCTTTGAAACTAAAAGCAATTACCCCAGAAAGTGTCTGCTAA

>p2_ind2201

ATGCAAATAGACCCCGTTGCATATCATTGTTCCGAGTTGAAGTTCGTGCTTGTACCCGAAGAGTCGGGAAAGTTAT
CCCCGACGGAAATAGGATTACATTTTCAGATTGGGCGCCGCGTACATGACAGAAGACCCCGCTCTGAGTCAGTGA
TGACGCACCAACGCAGATATCGATGTCTCACGTCTACCTTATTACTTGCCTTGATCGTCGGCACCCGATATTTGTCA
ACTGGGAATCCTCCTGGACCTATTATCGGTCCTTTGAAACTAACAGCAATTACCCCAGAAAGTGTCTGCTAA

>p2_ind4337

ATGCAAATAGACCCCGTTGCATATCATTGTTCCGAGTTGAAGTTCGTGCTTGTACCCGAAGAGTCGGGAAAGTTAT
CCCCGACGGAAATAGGATTCCATTTTCAGATTGGGTGCCGCGTACATGGCAGAAGACCCCGCTCTGAGTCAGTGA
TGACGCACCAACGCAGATATCGATGTCTCACGTCTACCTTATTACTTGCCTTGATCGTCGGCACCCGATATTTGTCA
ACTGGGAATCCTCCTGGACCTATTATCGGTCCTTTGAAACTAACAGCAATTACCCCAGAAAGTGTCTGCTAA

>p2_ind706

ATGCAAATAGACCCCGTTGCATATCATTGTTCCGAGTTGAAGTTCGTGCTTGTACCCGAAGAGTCGGGAAAGTTAT
CCCCGACGGAAATAGGATTCCATTTTCAGATTGGGTGCCGCGTACATGACAGAAGACCCCGCTCTGAGTCAGTGA
TGACGCACCAACGCAGATATCGATGTCTCACGTCTACCTTATTACTTGCCTTGATCGTCGGCACCCGATATTTGTCA
ACTGGGAATCCTCCTGGACCTATTATCGGTCCTTTGAAACTAACAGCAATTACCCCAGAAAGTGTCTGCTAA

>p2_ind1190

ATGCAAATAGACCCCGTTGCATATCATTGTTCCGAGTTGAAGTTCGTGCTTGTACCCGAAGAGTCGGGAAAGTTAT
CCCCGACGGAAATAGGATTCCATTTTCAGATTGGGTGCCGCGTACATGACAGAAGACCCCGCTCTGAGTCAGTGA
TGACGCACCAACGCAGATATCGATGTCTCACGTCTACCTTATTACTTGCCTTGATCGTCGGCACCCGATATTTGTCA
ACTGGGAATCCTCCTGGACCTATTATCGGTCCTTTGAAACTAACAGCAATTACCCCAGAAAGTGTCTGCTAA

>p2_ind2100

ATGCAAATAGACCCCGTTGCATATCATTGTTCCGAGTTGAAGTTCGTGCTTGTACGCGAAGAGTCGGGAAAGTTAT
CCCCGACGGAAATAGGATTCCATTTTCAGATTGGGTGCCGCGTACATGACAGAAGACCCCGCTCTGAGTCAGTGA
TGGCGCACCAACGCAAATATCGATGTCTCACGTCTACCTTATTACTTGCCTTGATCGTCGGCACCCGATATTTGTCA
ACTGGGAATCCTCCTGGACCTATTATCGGTCCTTTGAAACTAACAGCAATTACCCCAGAAAGTGTCTGCTAA

>p2_ind2212

ATGCAAATAGACCCCGTTGCATATCATTGTTCCGAGTTGAAGTTCGTGCTTGTACCCGAAGAGTCGGGAAAGTTAT
CCCCGACGGAAATAGGATTCCATTTTCAGATTGGGTGCCGCGTACATGACAGAAGACCCCGCTCTGAGTCAGTGA
TGACGCACCAACGCAGATATCGATGTCTCACGTCTACCTTATTACTTGCCTTGATCGTCGGCACCCGATATTTGTCA
ACTGGGAATCCTCCTGGACCTATTATCGGTCCTTTGAAACTAACAGCAATTACCCCAGAAAGTGTCTGCTAA

>p2_ind2193

ATGCAAATAGACCCCGTTGCATATCATTGTTCCGAGTTGAAGTTCGTGCTTGTACCCGAAGAGTCGGGAAAGTTAT
CCCCGACGGAAATAGGATTCCATTTTCAGATTGGGTGCCGCGTACATGACAGAAGACCCCGCTCTGAGTCAGTGA
TGACGCACCAACGCAGATATCGATGTCTCACGTCTACCTTATTACTTGCCTTGATCGTCGGCACCCGATATTTGTCA
ACTGGGAATCCTCCTGGACCTATTATCGGTCCTTTGAAACTAACAGCAATTACCCCAGAAAGTGTCTGCTAA

>p2_ind4368

ATGCAAATAGACCCCGTTGCATATCATTGTTCCGAGTTGAAGTTCGTGCTTGTACCCGAAGAGTCGGGAAAGTTAT
CCCCGACGGAAATAGGATTCCATTTTCAGATTGGGTGCCGCGTACATGACAGAAGACCCCGCTCTGAGTCAGTGA
TGACGCACCAACGCAGATATCGATGTCTCACGTCTACCTTATTACTTGCCTTGATCGTCGGCACCCGATATTTGTCA
ACTGGGAATCCTCCTGGACCTATTATCGGTCCTTTGAAACTAACAGCAATTACCCCAGAAAGTGTCTGCTAA

>p2_ind3589

ATGCAAATAGACCCCGTTGCATATCATTGTTCCGAGTTGAAGTTCGTGCTTGTACCCGAAGAGTCGGGAAAGTTAT
CCCCGACGGAAATAGGATTCCATTTTCAGATTGGGTGCCGCGTCCATGACAGAAGACCCCGCTCTGAGTCAGTGAT
GACGCACCTACGCAGATATCGATGTCTCAAGTCTACCTTATTACTTGCCTTGATCGTCGGCACCCGATATTTGTCAA
CTGGGAATCCTCCTGGACCTATTATCGGTCCTTTGAAACTAACAGCAATTACCCCAGAAAGTGTCTGCTAA

>p2_ind781

ATGCAAATAGACCCCGTTGCATATCATTGTTCCGAGTTGAAGTTCGTGCTTGTACCCGAAGAGTCGGGAAAGTTAT
CCCCGACGGAAATAGGATTCCATTTTCAGATTGGGTGCCGCGTACATGACAGAAGACCCCGCTCTGAGTCAGTGA
TGGCGCACCAACGCAAATATCGATGTCTCACGTCTACCTTATTACTTGCCTTGATCGTCGGCACCCGATATTTGTCA
ACTGGGAATCCTCCTGGACCTATTATCGGTCCTTTGAAACTAACAGCAATTACCCCAGAAAGTGTCTGCTAA

>p2_ind395

ATGCAAATAGACCCCGTTGCATATCATTGTTCCGAGTTGAAGTTCGTGCTTGTACCCGAAGAGTCGGGAAAGTTAT
CCCCGACGGAAATAGGATTCCATTTTCAGATTGGGTGCCGCGCACATGACAGAAGACCCCGCTCTGAGTCAGTGA

TGACGCACCTACGCAGATATCGATGTCTCACGTCTACCTTATTACTTGCCTTGATCGTCGGCACCCGATATTTGTCA
ACTGGGAATCCTCCTGGACCTATTATCGGTCCTTTGAAACTAACAGCAATTACCCCAGAAAGTGTCTGCTAA

>p2_ind2807

ATGCAAATAGACCCCGTTGCATATCATTGTTCCGAGTTGAAGTTCGTGCTTGTACCCGAAGAGTCGGGAAAGTTAT
CCCCGACGGAAATAGGATTCCATTTTCAGATTGGGTGCCGCGTACATGACAGAAGACCCCGCTCTGAGTCAGTGA
TGACGCACCAACGCAGATATCGATGTCTCACGTCTACCTTATTACTTGCCTTGATCGTCGGCACCCGATATTTGTCA
ACTGGGAATCCTCCTGGACCTATTATCGGTCCTTTGAAACTAACAGCAATTACCCCAGAAAGTGTCTGCTAA

>p2_ind2365

ATGCAAATAGACCCCGTTGCATATCATTGTTCCGAGTTGAAGTTCGTGCTTGTACCCGAAGAGTCGGGAAAGTTAT
CCCCGACGGAAATAGGATTCCATTTTCAGATTGGGTGCCGCGTACATGACAGAAGACCCCGCTCTGAGTCAGTGA
TGACGCACCAACGCAGATATCGATGTCTCACGTCTACCTTATTACTTGCCTTGATCGTCGGCACCCGATATTTGTCA
ACTGGGAATCCTCCTGGACCTATTATCGGTCCTTTGAAACTAACAGCAATTACCCCAGAAAGTGTCTGCTAA

>p2_ind1593

ATGCAAATAGACCCCGTTGCATATCATTGTTCCGAGTTGAAGTTCGTGCTTGTACCCGAAGAGTCGGGAAAGTTAT
CCCCGACGGAAATAGGATTCCATTTTCAGATTGGGTGCCGCGCACATGACAGAAGACCCCGCTCTGAGTCAGTGA
TGACGCACCTACGCAGATATCGATGTCTCACGTCTACCTTATTACTTGCCTTGATCGTCGGCACCCGATATTTGTCA
ACTGGGAATCCTCCTGGACCTATTATCGGTCCTTTGAAACTAACAGCAATTACCCCAGAAAGTGTCTGCTAA

>p2_ind3921

ATGCAAATAGACCCCGTTGCATATCATTGATCCGAGTTGAAGTTCGTGCTTGTACCCGAAGAGTCGGGAAAGTTAT
CCCCGACGGAAATAGGATTCCATTTTCAGATTGGGTGCCGCGTACATGACAGAAGACCCCGTTCTGAGTCAGTGAT
GACGCACCAACGCAGATATCGATGTCTCACGTCTACCTTATTACTTGCCTTGATCGTCGGCACCCGATATTTGTCAA
CTGGGAATCCTCCTGGACCTATTATCGGTCCTTTGAAACTAACAGCAATTACCCCAGAAAGTGTCTGCTAA

>p2_ind4410

ATGCAAATAGACCCCGTTGCATATCATTGTTCCGAGTTGAAGTTCGTGCTTGTACCCGAAGAGTCGGGAAAGTTAT
CCCCGACGGAAATAGGATTCCATTTTCAGATTGGGTGCCGCGTACATGACAGAAGACCCCGCTCTGAGTCAGTGA
TGACGCACCAACGCAGATATCGATGTCTCACGTCTACCTTATTACTTGCCTTGATCGTCGGCACCCGATATTTGTCA
ACTGGGAATCCTCCTGGACCTATTATCGGTCCTTTGAAACTAACAGCAATTACCCCAGAAAGTGTCTGCTAA

>p2_ind1670

ATGCAAATAGACCCCGTTGCATATCATTGTTCCGAGTTGAAGTTCGTGCTTGTACCCGAAGAGTCGGGAAAGTTAT
CCCCGACGGAAATAGGATTCCATTTTCAGATTGGGCGCCGCGTACATGACAGAAGACCCCGCTCTGAGTCAGTGA
TGACGCACCAACGCAGATATCGATGTCTCACGTCTACCTTATTACTTGCCTTGATCGTCGGCACCCGATATTTGTCA
ACTGGGAATCCTCCTGGACCTATTATCGGTCCTTTGAAACTAACAGCAATTACCCCAGAAAGTGTCTGCTAA

>p2_ind2515

ATGCAAATAGACCCCGTTGCATATCATTGTTCCGAGTTGAAGTTCGTGCTTGTACCCGAAGAGTCGGGAAAGTTAT
CCCCGACGGAAATAGGATTCCATTTTCAGATTGGGTGCCGCGTACATGACAGAAGACCCCGCTCTGAGTCAGTGA
TGACGCACCAACGCAGATATCGATGTCTCACGTCTACCTTATTACTTGCCTTGATCGTCGGCACCCGATATTTGTCA
ACTGGGAATCCTCCTGGACCTATTATCGGTCCTTTGAAACTAACAGCAATTACCCCAGAAAGTGTCTGCTAA

>p2_ind946

ATGCAAATAGACCCCGTTGCATATCATTGTTCCGAGTTGAAGTTCGTGCTTGTACCCGAAGAGTCGGGAAAGTTAT
CCCCGACGGAAATAGGATTCCATTTTCAGATTGGGTGCCGCGTACATGACAGAAGACCCCGCTCTGAGTCAGTGA
TGACGCACCAACGCAGATATCGATGTCTCACGTCTACCTTATTACTTGCCTTGATCGTCGGCACCCGATATTTGTCA
ACTGGGAATCCTCCTGGACCTATTATCGGTCCTTTGAAACTAACAGCAATTACCCCAGAAAGTGTCTGCTAA

>p2_ind1283

ATGCAAATAGACCCCGTTGCATATCATTGTTCCAAGTTGAAGTTCGTGCTTGTACCCGAAGAGTCGGGAAAGTTAT
CCCCGACGGAAATAGGATTCCATTTTCAGATTGGGTGCCGCGTACATGACAGAAGACCCCGCTCTGAGTCAGTGA
TGACGCACCAACGCAGATATCGATGTCTCACGTCTACCTTATTACTTGCCTTGATCGTCGGCACCCGATATTTGTCA
ACTGGGAATCCTCCTGGACCTATTATCGGTCCTTTGAAACTAACAGCAATTACCCCAGAAAGTGTCTGCTAA

>p2_ind1951

ATGCAAATAGACCCCGTTGCATATCATTGTTCCGAGTTGAAGTTCGTGCTTGTACCCGAAGAGTCGGGAAAGTTAT
CCCCGACGGAAATAGGATTCCATTTTCAGATTGGGTGCCGCGTACATGACAGAAGACCCCGCTCTGAGTCAGTGA
TGACGCACCAACGCAGATATCGATGTCTCACGTCTACCTTATTACTTGCCTTGATCGTCGGCACCCGATATTTGTCA
ACTGGGAATCCTCCTGGACCTATTATCGGTCCTTTGAAACTAACAGCAATTACCCCAGAAAGTGTCTGCTAA

>p2_ind4269

ATGCAAATAGACCCCGTTGCATATCATTGTTCCGAGTTGAAGTTCGTGCTTGTACCCGAAGAGTCGGGAAAGTTAT
CCCCGACGGAAATAGGATTCCATTTTCAGATTGGGTGCCGCGTACATGACAGAAGACCCCGCTCTGAGTCAGTGA
TGACGCACCAACGCAGATATCGATGTCTCACGTCTACCTTATTACTTGCCTTGATCGTCGGCACCCGATATTTGTCA
ACTGGGAATCCTCCTGGACCTATTATCGGTCCTTTGAAACTAACAGCAATTACCCCAGAAAGTGTCTGCTAA

>p2_ind375

ATGCAAATAGACCCCGTTGCATATCATTGTTCCGAGTTGAAGTTCGTGCTTGTACCCGAAGAGTCGGGAAAGTTAT
CCCCGACGGAAATAGGATTCCATTTTCAGATTGGGTGCCGCGTACATGACAGAAGACCCCGCTCTGAGTCAGTGA
TGGCGCACCAACGCAAATATCGATGTCTCACGTCTACCTTATTACTTGCCTTGATCGTCGGCACCCGATATTTGTCA
ACTGGGAATCCTCCTGGACCTATTATCGGTCCTTTGAAACTAACAGCAATTACCCCAGAAAGTGTCTGCTAA

>p2_ind121

ATGCAAATAGACCCCGTTGCATATCATTGTTCCGAGTTGAAGTTCGTGCTTGTACCCGAAGAGTCGGGAAAGTTAT
CCCCGACGGAAATAGGATTCCATTTTCAGATTGGGTGCCGCGTACATGACAGAAGACCCCGCTCTGAGTCAGTGA

TGACGCACCAACGCAGATATCGATGTCTCACGTCTACCTTATTACTTGCCTTGATCGTCGGCACCCGATATTTGTCA
ACTGGGAATCCTCCTGGACCTATTATCGGTCCTTTGAAACTAACAGCAATTACCCCAGAAAGTGTCTGCTAA

>p2_ind4555

ATGCAAATAGACCCCGTTGCATATCATTGTTCCGAGTTGAAGTTCGTGCTTGTACCCGAAGAGTCGGGAAAGTTAT
CCCCGACGGAAATAGGATTCCATTTTCAGATTGGGTGCCGCGTACATGACAGAAGACCCCGCTCTGAGTCAGTGA
TGACGCACCAACGCAGATATCGATGTCTCACGTCTACCTTATTACTTGCCTTGATCGTCGGCACCCGATATTTGTCA
ACTGGGAATCCTCCTGGACCTATTATCGGTCCTTTGAAACTAACAGCAATTACCCCAGAAAGTGTCTGCTAA

>p2_ind276

ATGCAAATAGACCCCGTTGCATATCATTGATCCGAGTTGAAGTTCGTGCTTGTACCCGAAGAGTCGGGAAAGTTAT
CCCCGACGGAAATAGGATTCCATTTTCAGATTGGGTGCCGCGTACATGACAGAAGACCCCGCTCTGAGTCAGTGA
TGACGCACCAACGCAGATATCGATGTCTCACGTCTACCTTATTACTTGCCTTGATCGTCGGCACCCGATATTTGTCA
ACTGGGAATCCTCCTGGACCTATTATCGGTCCTTTGAAACTAACAGCAATTACCCCAGAAAGTGTCTGCTAA

>p2_ind3766

ATGCAAATAGACCCCGTTGCATATCATTGTTCCGAGTTGAAGTTCGTGCTTGTACCCGAAGAGTCGGGAAAGTTAT
CCCCGACGGAAATAGGATTCCATTTTCAGATTGGGTGCCGCGTACATGACAGAAGACCCCGCTCTGAGTCAGTGA
TGACGCACCAACGCAGATATCGATGTCTCACGTCTACCTTATTACTTGCCTTGATCGTCGGCACCCGATATTTGTCA
ACTGGGAATCCTCCTGGACCTATTATCGGTCCTTTGAAACTAACAGCAATTACCCCAGAAAGTGTCTGCTAA

>p2_ind4201

ATGCAAATAGACCCCGTTGCATATCATTGTTCCGAGTTGAAGTTCGTGCTTGTACCCGAAGAGTCGGGAAAGTTAT
CCCCGACGGAAATAGGATTCCATTTTCAGATTGGGTGCCGCGTACATGACAGAAGACCCCGCTCTGAGTCAGTGA
TGACGCACCAACGCAGATATCGATGTCTCACGTCTACCTTATTACTTGCCTTGATCGTCGGCACCCGATATTTGTCA
ACTGGGAATCCTCCTGGACCTATTATCGGTCCTTTGAAACTAACAGCAATTACCCCAGAAAGTGTCTGCTAA

>p2_ind1261

ATGCAAATAGACCCCGTTGCATATCATTGTTCCGAGTTGAAGTTCGTGCTTGTACCCGAAGAGTCGGGAAAGTTAT
CCCCGACGGAAATAGGATTCCATTTTCAGATTGGGTGCCGCGTACATGACAGAAGACCCCGCTCTGAGTCAGTGA
TGACGCACCAACGCAGATATCGATGTCTCACGTCTACCTTATTACTTGCCTTGATCGTCGGCACCCGATATTTGTCA
ACTGGGAATCCTCCTGGACCTATTATCGGTCCTTTGAAACTAACAGCAATTACCCCAGAAAGTGTCTGCTAA

>p2_ind4634

ATGCAAATAGACCCCGTTGCATATCATTGTTCCGAGTTGAAGTTCGTGCTTGTACCCGAAGAGTCGGGAAAGTTAT
CCCCGACGGAAATAGGATTCCATTTTCAGATTGGGTGCCGCGTACATGACAGAAGACCCCGCTCTGAGTCAGTGA
TGACGCACCAACGCAGATATCGATGTCTCACGTCTACCTTATTACTTGCCTTGATCGTCGGCACCCGATATTTGTCA
ACTGGGAATCCTCCTGGACCTATTATCGGTCCTTTGAAACTAACAGCAATTACTCCAGAAAGTGTCTGCTAA

>p2_ind4295

ATGCAAATAGACCCCGTTGCATATCATTGTTCCGAGTTGAAGTTCGTGCTTGTACCCGAAGAGTCGGGAAAGTTAT
CCCCGACGGAAATAGGATTCCATTTTCAGATTGGGTGCCGCGTACATGACAGAAGACCCCGCTCTGAGTCAGTGA
TGACGCACCAACGCAGATATCGATGTCTCACGTCTACCTTATTACTTGCCTTGATCGTCGGCACCCGATATTTGTCA
ACTGGGAATCCTCCTGGACCTATTATCGGTCCTTTGAAACTAACAGCAATTACCCCAGAAAGTGTCTGCTAA

>p2_ind2674

ATGCAAATAGACCCCGTTGCATATCATTGTTCCGAGTTGAAGTTCGTGCTTGTACCCGAAGAGTCGGGAAAGTTAT
CCCCGACGGAAATAGGATTCCATTTTCAGATTGGGTGCCGCGTACATGACAGAAGACCCCGCTCTGAGTCAGTGA
TGACGCACCAACGCAGATATCGATGTCTCACGTCTACCTTATTACTTGCCTTGATCGTCGGCACCCGATATTTGTCA
ACTGGGAATCCTCCTGGACCTATTATCGGTCCTTTGAAACTAACAGCAATTACCCCAGAAAGTGTCTGCTAA

>p2_ind3323

ATGCAAATAGACCCCGTTGCATATCATTGTTCCGAGTTGAAGTTCGTGCTTGTACCCGAAGAGTCGGGAAAGTTAT
CCCCGACGGAAATAGGATTCCATTTTCAGATTGGGTGCCGCGTACATGACAGAAGACCCCGCTCTGAGTCAGTGA
TGACGCACCAACGCAGATATCGATGTCTCACGTCTACCTTATTACTTGCCTTGATCGTCGGCACCCGATATTTGTCA
ACTGGGAATCCTCCTGGACCTATTATCGGTCCTTTGAAACTAACAGCAATTACTCCAGAAAGTGTCTGCTAA

>p2_ind295

ATGCAAATAGACCCCGTTGCATATCATTGTTCCGAGTTGAAGTTCGTGCTTGTACCCGAAGAGTCGGGAAAGTTAT
CCCCGACGGAAATAGGATTCCATTTTCAGATTGGGTGCCGCGTACATGACAGAAGACCCCGCTCTGAGTCAGTGA
TGACGCACCAACGCAGATATCGATGTCTCACGTCTACCTTATTACTTGCCTTGATCGTCGGCACCCGATATTTGTCA
ACTGGGAATCCTCCTGGACCTATTATCGGTCCTTTGAAACTAACAGCAATTACTCCAGAAAGTGTCTGCTAA

>p2_ind972

ATGCAAATAGACCCCGTTGCATATCATTGTTCCGAGTTGAAGTTCGTGCTTGTACCCGAAGAGTCGGGAAAGTTAT
CCCCGACGGAAATAGGATTCCATTTTCAGATTGGGTGCCGCGTACATGACAGAAGACCCCGCTCTGAGTCAGTGA
TGACGCACCAACGCAGATATCGATGTCTCACGTCTACCTTATTACTTGCCTTGATCGTCGGCACCCGATATTTGTCA
ACTGGGAATCCTCCTGGACCTATTATCGGTCCTTTGAAACTAACAGGAATTACCCCAGAAAGTGTCTGCTAA

>p2_ind3219

ATGCAAATAGACCCCGTTGCATATCATTGTTCCGAGTTGAAGTTCGTGCTTGTACCCGAAGAGTCGGGAAAGTTAT
CCCCGACGGAAATAGGATTCCATTTTCAGATTGGGTGCCGCGTACATGACAGAAGACCCCGCTCTGAGTCAGTGA
TGACGCACCAACGCAGATATCGATGTCTCACGTCTACCTTATTACTTGCCTTGATCGTCGGCACCCGATATTTGTCA
ACTGGGAATCCTCCTGGACCTATTATCGGTCCTTTGAAACTAACAGCAATTACCCCAGAAAGTGTCTGCTAA

>p2_ind925

ATGCAAATAGACCCCGTTGCATATCATTGTTCCGAGTTGAAGTTCGTGCTTGTACCCGAAGAGTCGGGAAAGTTAT
CCCCGACGGAAATAGGATTCCATTTTCAGATTGGGTGCCGCGTACATGACAGAAGACCCCGCTCTGAGTCAGTGA

TGACGCACCAACGCAGATATCGATGTCTCACGTCTACCTTATTACTTGCCTTGATCGTCGGCACCCGATATTTGTCA
ACTGGGAATCCTCCTGGACCTATTATCGGTCCTTTGAAACTAACAGCAATTACCCCAGAAAGTGTCTGCTAA

>p2_ind988

ATGCAAATAGACCCCGTTGCATATCATTGTTCCGAGTTGAAGTTCGTGCTTGTACCCGAAGAGTCGGGAAAGTTAT
CCCCGACGGAAATAGGATTCCATTTTCAGATTGGGTGCCGCGTACATGACAGAAGACCCCGCTCTGAGTCAGTGA
TGACGCACCAACGCAGATATCGATGTCTCACGTCTACCTTATTACTTGCCTTGATCGTCGGCACCCGATATTTGTCA
ACTGGGAATCCTCCTGGACCTATTATCGGTCCTTTGAAACTAACAGCAATTACTCCAGAAAGTGTCTGCTAA

>p2_ind3155

ATGCAAATAGACCCCGTTGCATATCATTGTTCCGAGTTGAAGTTCGTGCTTGTACCCGAAGAGTCGGGAAAGTTAT
CCCCGACGGAAATAGGATTCCATTTTCAGATTGGGTGCCGCGTACATGACAGAAGACCCCGCTCTGAGTCAGTGA
TGACGCACCAACGCAGATATCGATGTCTCACGTCTACCTTATTACTTGCCTTGATCGTCGGCACCCGATATTTGTCA
ACTGGGAATCCTCCTGGACCTATTATCGGTCCTTTGAAACTAACAGCAATTACCCCAGAAAGTGTCTGCTAA

>p2_ind2150

ATGCAAATAGACCCCGTTGCATATCATTGTTCCGAGTTGAAGTTCGTGCTTGTACCCGAAGAGTCGGGAAAGTTAT
CCCCGACGGAAATAGGATTCCATTTTCAGATTGGGTGCCGCGTACATGACAGAAGACCCCGCTCTGAGTCAGTGA
TGGCGCACCAACGCAAATATCGATGTCTCACGTCTACCTTATTACTTGCCTTGATCGTCGGCACCCGATATTTGTCA
ACTGGGAATCCTCCTGGACCTATTATCGGTCCTTTGAAACTAACAGCAATTACCCCAGAAAGTGTCTGCTAA

>p2_ind4184

ATGCAAATAGACCCCGTTGCATATCATTGTTCCGAGTTGAAGTTCGTGCTTGTACCCGAAGAGTCGGGAAAGTTAT
CCCCGACGGAAATAGGATTCCATTTTCAGATTGGGTGCCGCGTACATGACAGAAGACCCCGCTCTGAGTCAGTGA
TGACGCACCAACGCAGATATCGATGTCTCACGTCTACCTTATTACTTGCCTTGATCGTCGGCACCCGATATTTGTCA
ACTGGGAATCCTCCTGGACCTATTATCGGTCCTTTGAAACTAACAGCAATTACCCCAGAAAGTGTCTGCTAA

>p2_ind3551

ATGCAAATAGACCCCGTTGCATATCATTGTTCCGAGTTGAAGTTCGTGCTTGTACCCGAAGAGTCGGGAAAGTTAT
CCCCGACGGAAATAGGATTCCATTTTCAGATTGGGTGCCGCGTACATGACAGAAGACCCCGCTCTGAGTCAGTGA
TGACGCACCAACGCAGATATCGATGTCTCACGTCTACCTTATTACTTGCCTTGATCGTCGGCACCCGATATTTGTCA
ACTGGGAATCCTCCTGGACCTATTATCGGTCCTTTGAAACTAACAGCAATTACCCCAGAAAGTGTCTGCTAA

>p2_ind2260

ATGCAAATAGACCCCGTTGCATATCATTGTTCCGAGTTGAAGTTCGTGCTTGTACCCGAAGAGTCGGGAAAGTTAT
CCCCGACGGAAATAGGATTCCATTTTCAGATTGGGTGCCGCGTACATGACAGAAGACCCCGCTCTGAGTCAGTGA
TGACGCACCAACGCAGATATCGATGTCTCACGTCTACCTTATTACTTGCCTTGATCGTCGGCACCCGATATTTGTCA
ACTGGGAATCCTCCTGGACCTATTATCGGTCCTTTGAAACTAACGGCAATTACCCCAGAAAGTGTCTGCTAA

>p2_ind2040

ATGCAAATAGACCCCGTTGCATATCATTGTTCCGAGTTGAAGTTCGTGCTTGTACCCGAAGAGTCGGGAAAGTTAT
CCCCGACGGAAATAGGATTCCATTTTCAGATTGGGTGCCGCGTACATGACAGAAGACCCCGCTCTGAGTCAGTGA
TGACGCTCCAACGCAGATATCGATGTCTCACGTCTACCTTATTACTTGCCTTGATCGTCGGCACCCGATATTTGTCA
ACTGGGAATCCTCCTGGACCTATTATCGGTCCTTTGAAACTAACAGCAATTACCCCAGAAAGTGTCTGCTAA

>p2_ind1718

ATGCAAATAGACCCCGTTGCATATCATTGTTCCGAGTTGAAGTTCGTGCTTGTACCCGAAGAGTCGGGAAAGTTAT
CCCCGACGGAAATAGGATTCCATTTTCAGATTGGGTGCCGCGTACATGACAGAAGACCCCGCTCTGAGTCAGTGA
TGACGCACCAACGCAGATATCGATGTCTCACGTCTACCTTATTACTTGCCTTGATCGTCGGCACCCGATATTTGTCA
ACTGGGAATCCTCCTGGACCTATTATCGGTCCTTTGAAACTAACGGCAATTACCCCAGAAAGTGTCTGCTAA

>p2_ind3525

ATGCAAATAGACCCCGTTGCATATCATTGTTCCGAGTTGAAGTTCGTGCTTGTACCCGAAGAGTCGGGAAAGTTAT
CCCCGACGGAAATAGGATTCCATTTTCAGATTGGGTGCCGCGTACATGACAGAAGACCCCGCTCTGAGTCAGTTAT
GACGCACCAACGCAGATATCGATGTCTCACGTCTACCTTATTACTTGCCTTGATCGTCGGCACCCGATATTTGTCAA
CTGGGAATCCTCCTGGACCTATTATCGGTCCTTTGAAACTAACAGCAATTACCCCAGAAAGTGTCTGCTAA

>p2_ind3838

ATGCAAATAGACCCCGTTGCATATCATTGATCCGAGTTGAAGTTCGTGCTTGTACCCGAAGAGTCGGGAAAGTTAT
CCCCGACGGAAATAGGATTCCATTTTCAGATTGGGTGCCGCGTACATGACAGAAGACCCCGTTCTGAGTCAGTGAT
GACGCACCAACGCAGATATCGATGTCTCACGTCTACCTTATTACTTGCCTTGATCGTCGGCACCCGATATTTGTCAA
CTGGGAATCCTCCTGGACCTATTATCGGTCCTTTGAAACTAACAGCAATTACCCCAGAAAGTGTCTGCTAA

>p2_ind3116

ATGCAAATAGACCCCGTTGCATATCATTGATCCGAGTTGAAGTTCGTGCTTGTACCCGAAGAGTCGGGAAAGTTAT
CCCCGACGGAAATAGGATTCCATTTTCAGATTGGGCGCCGCGTACATGACAGAAGACCCCGCTCTGAGTCAGTGA
TGACGCACCAACGCAGATATCGATGTCTCACGTCTACCTTATTACTTGCCTTGATCGTCGGCACCCGATATTTGTCA
ACTGGGAATCCTCCTGGACCTATTATCGGTCCTTTGAAACTAACAGCAATTACCCCAGAAAGTGTCTGCTAA

>p2_ind619

ATGCAAATAGACCCCGTTGCATATCATTGTTCCGAGTTGAAGTTCGTGCTTGTACCCGAAGAGTCGGGAAAGTTAT
CCCCGACGGAAATAGGATTCCATTTTCAGATTGGGTGCCGCGTACATGACAGAAGACCCCGCTCTGAGTCAGTGA
TGACGCACCAACGCAGATATCGATGTCTCACGTCTACCTTATTACTTGCCTTGATCGTCGGCACCCGATATTTGTCA
ACTGGGAATCCTCCTGGACCTATTATCGGTCCTTTGAAACTAACAGCAATTACCCCAGAAAGTGTCTGCTAA

>p2_ind1354

ATGCAAATAGACCCCGTTGCATATCATTGTTCCGAGTTGAAGTTCGTGCTTGTACCCGAAGAGTCGGGAAAGTTAT
CCCCGACGGAAATAGGATTCCATTTTCAGATTGGGTGCCGCGTACATGACAGAAGACCCCGCTCTGAGTCAGTGA

TGACGCACCAACGCAGATATCGATGTCTCACGTCTACCTTATTACTTGCCTTGATCGTCGGCACCCGATATTTGTCA
ACTGGGAATCCTCCTGGACCTATTATCGGTCCTTTGAAACTAACAGCAATTACCCCAGAAAGTGTCTGCTAA

>p2_ind3492

ATGCAAATAGACCCCGTTGCATATCATTGTTCCGAGTTGAAGTTCGTGCTTGTACCCGAAGAGTCGGGAAAGTTAT
CCCCGACGGAAATAGGATTCCATTTTCAGATTGGGTGCCGCGTACATGACAGAAGACCCCGCTCTGAGTCAGTGA
TGACGCACCAACGCAGATATCGATGTCTCACGTCTACCTTATTACTTGCCTTGATCGTCGGCACCCGATATTTGTCA
ACTGGGAATCCTCCTGGACCTATTATCGGTCCTTTGAAACTAACAGCAATTACCCCAGAAAGTGTCTGCTAA

>p2_ind85

ATGCAAATAGACCCCGTTGCATATCATTGTTCCGAGTTGAAGTGCGTGCTTGTACCCGAAGAGTCGGGAAAGTTAT
CCCCGACGGAAATAGGATTCCATTTTCAGATTGGGTGCCGCGTACATGACAGAAGACCCCGCTCTGAGTCAGTGA
TGGCGCACCAACGCAAATATCGATGTCTCACGTCTACCTTATTACTTGCCTTGATCGTCGGCACCCGATATTTGTCA
ACTGGGAATCCTCCTGGACCTATTATCGGTCCTTTGAAACTAACAGCAATTACCCCAGAAAGTGTCTGCTAA

>p2_ind2867

ATGCAAATAGACCCCGTTGCATATCATTGTTCCGAGTTGAAGTTCGTGCTTGTACCCGAAGAGTCGGGAAAGTTAT
CCCCGACGGAAATAGGATTCCATTTTCAGATTGGGTGCCGCGTACATGACAGAAGACCCCGCTCTGAGTCAGTGA
TGACGCACCAACGCAGATATCGATGTCTCACGTCTACCTTATTACTTGCCTTGATCGTCGGCACCCGATATTTGTCA
ACTGGGAATCCTCCTGGACCTATTATCGGTCCTTTGAAACTAACAGCAATTACACCAGAAAGTGTCTGCTAA

>p2_ind1702

ATGCAAATAGACCCCGTTGCATATCATTGTTCCGAGTTGAAGTTCGTGCTTGTACCCGAAGAGTCGGGAAAGTTAT
CCCCGACGGAAATAGGATTCCATTTTCAGATTGGGTGCCGCGTACATGACAGAAGACCCCGCTCTGAGTCAGTGA
TGACGCACCAACGCAGACATCGATGTCTCACGTCTACCTTATTACTTGCCTTGATCGTCGGCACCCGATATTTGTCA
ACTGGGAATCCTCCTGGACCTATTATCGGTCCTTTGAAACTAACAGCAATTACCCCAGAAAGTGTCTGCTAA

>p2_ind4970

ATGCAAATAGACCCCGTTGCATATCATTGTTCCGAGTTGAAGTTCGTGCTTGTACCCGAAGAGTCGGGAAAGTTAT
CCCCGACGGAAATAGGATTCCATTTTCAGATTGGGTGCCGCGTACATGACAGAAGACCCCGCTCTGAGTCAGTGA
TGACGCACCTACGCAGATATCGATGTCTCAAGTCTACCTTATTACTTGCCTTGATCGTCGGCACCCGATATTTGTCA
ACTGGGAATCCTCCTGGACCTATTATCGGTCCTTTGAAACTAACAGCAATTACCCCAGAAAGTGTCTGCTAA

>p2_ind2850

ATGCAAATAGACCCCGTTGCATATCATTGTTCCGAGCTGAAGTTCGTGCTTGTACCCGAAGAGTCGGGAAAGTTAT
CCCCGACGGAAATAGGATTCCATTTTCAGATTGGGTGCCGCGTACATGACAGAAGACCCCGCTCTGAGTCAGTGA
TGACGCACCAACGCAGATATCGATGTCTCACGTCTACCTTATTACTTGCCTTGATCGTCGGCACCCGATATTTGTCA
ACTGGGAATCCTCCTGGACCTATTATCGGTCCTTTGAAACTAACAGGAATTACCCCAGAAAGTGTCTGCTAA

>p2_ind405

ATGCAAATAGACCCCGTTGCATATCATTGTTCCGAGTTGAAGTTCGTGCTTGTACCCGAAGAGTCGGGAAAGTTAT
CCCCGACGGAAATAGGATTCCATTTTCAGATTGGGTGCCGCGTACATGACAGAAGACCCCGCTCTGAGTCAGTGA
TGACGCACCAACGCAGACATCGATGTCTCACGTCTACCTTATTACTTGCCTTGATCGTCGGCACCCGATATTTGTCA
ACTGGGAATCCTCCTGGACCTATTATCGGTCCTTTGAAACTAACAGCAATTACCCCAGAAAGTGTCTGCTAA

>p2_ind742

ATGCAAATAGACCCCGTTGCATATCATTGTTCCGAGTTGAAGTTCGTGCTTGTACCCGAAGAGTCGGGAAAGTTAT
CCCCGACGGAAATAGGATTCCATTTTCAGATTGGGTGCCGCGTACATGACAGAAGACCCCGCTCTGAGTCAGTGA
TGACGCACCAACGCAGATATCGATGTCTCACGTCTACCTTATTACTTGCCTTGATCGTCGGCACCCGATATTTGTCA
ACTGGGAATCCTCCTGGACCTATTATCGGTCCTTTGAAACTAACAGCAATTACCCCAGAAAGTGTCTGCTAA

>p2_ind3129

ATGCAAATAGACCCCGTTGCATATCATTGTTCCGAGTTGAAGTTCGTGCTTGTATCCGAAGAGTCGGGAAAGTTAT
CCCCGACGGAAATAGGATTCCATTTTCAGATTGGGTGCCGCGTACATGACAGAAGACCCCGCTCTGAGTCAGTGA
TGACGCACCAACGCAGATATCGATGTCTCACGTCTACCTTATTACTTGCCTTGATCGTCGGCACCCGATATTTGTCA
ACTGGGAATCCTCCTGGACCTATTATCGGTCCTTTGAAACTAACAGCAATTACCCCAGAAACTGTCTGCTAA

>p2_ind827

ATGCAAATAGACCCCGTTGCATATCATTGTTCCGAGTTGAAGTTCGTGCTTGTACCCGAAGAGTCGGGAAAGTTAT
CCCCGACGGAAATAGGATTCCATTTTCAGATTGGGTGCCGCGTACATGACAGAAGACCCCGTTCTGAGTCAGTGAT
GACGCACCAACGCAGATATCGATGTCTCACGTCTACCTTATTACTTGCCTTGATCGTCGGCACCCGATATTTGTCAA
CTGGGAATCCTCCTGGACCTATTATCGGTCCTTTGAAACTAACAGCAATTACCCCAGAAAGTGTCTGCTAA

>p2_ind2914

ATGCAAATAGACCCTGTTGCATATCATTGTTCCGAGTTGAAGTTCGTGCTTGTACCCGAAGAGTCGGGAAAGTTAT
CCCCGACGGAAATAGGATTCCATTTTCAGATTGGGTGCCGCGTACATGACAGAAGACCCCGCTCTGAGTCAGTGA
TGGCGCACCAACGCAAATACAGATGTCTCACGTCTACCTTAATACTTGCCTTGATCGTCGGCACCCGATATTTGTCA
ACTGGGAATCCTCCTGGACCTATTATCGGTCCTTTGAAACTAACAGCAATTACCCCAGAAAGTGTCTGCTAA

>p2_ind2559

ATGCAAATAGACCCCGTTGCATATCATTGTTCCGAGTTGAAGTTCGTGCTTGTACCCGAAGAGTCGGGAAAGTTAT
CCCCGACGGAAATAGGATTCCATTTTCAGATTGGGTGCCGCGTACATGACAGAAGACCCCGCTCTGAGTCAGTGA
TGACGCACCAACGCAGATATCGATGTCTCACGTCTACCTTATTACTTGCCTTGATCGTCGGCACCCGATATTTGTCA
ACTGGGGATCCTCCTGGACCTATTATCGGTCCTTTGAAACTAACAGGAATTACCCCAGAAAGTGTCTGCTAA

>p2_ind3230

ATGCAAATAGACCCCGTTGCATATCATTGTTCCAAGTTGAAGTTCGTGCTTGTACCCGAAGAGTCGGGAAAGTTAT
CCCCGACGGAAATAGGATTCCATTTTCAGATTGGGTGCCGCGTACATGACAGAAGACCCCGCTCTGAGTCAGTGA

TGACGCACCAACGCAGATATCGATGTCTCACGTCTACCTTATTACTTGCCTTGATCGTCGGCACCCGATATTTGTCA
ACTGGGAATCCTCCTGGACCTATTATCGGTCCTTTGAAACTAACAGCAATTACCCCAGAAAGTGTCTGCTAA

>p2_ind3396

ATGCAAATAGACCCCGTTGCATATCATTGTTCCGAGTTGAAGTTCGTGCTTGTACCCGAAGAGTCGGGAAAGTTAT
CCCCGACGGAAATAGGATTCCATTTTCAGATTGGGTGCCGCGTACATGACAGAAGACCCCGCTCTGAGTCAGTGA
TGACGCACCAACGCAGATATCGATGTCTCACGTCTACCTTATTACTTGCCTTGATCGTCGGCACCCGATATTTGTCA
ACTGGGAATCCTCCTGGACCTATTATCGGTCCTTTGAAACTAACAGCAATTACCCCAGAAAGTGTCTGCTAA

>p2_ind692

ATGCAAATAGACCCCGTTGCATATCATTGTTCCGAGTTGAAGTTCGTGCTTGTACCCGAAGAGTCGGGAAAGTTAT
CCCCGACGGAAATAGGATTCCATTTTCAGATTGGGTGCCGCGTACATGACAGAAGACCCCGCCCTGAGTCAGTGA
TGACGCACCAACGCAGATATTGATGTCTCACGTCTACCTTATTACTTGCCTTGATCGTCGGCACCCGATATTTGTCA
ACTGGGAATCCTCCTGGACCTATTATCGGTCCTTTGAAACTAACAGCAATTACCCCAGAAAGTGTCTGCTAA

>p2_ind1053

ATGCAAATAGACCCCGTTGCATATCATTGTTCCGAGTTGAAGTTCGTGCTTGTACCCGAAGAGTCGGGAAAGTTAT
CCCCGACGGAAATAGGATTCCATTTTCAGATTGGGTGCCGCGTACATGACAGAAGACCCCGCTCTGAGTCAGTGA
TGACGCACCAACGCAGATATCGATGTCTCACGTCTACCTTATTACTTGCCTTGATCGTCGGCACCCGATATTTGTCA
ACTGGGAATCCTCCTGGACCTATTATCGGTCCTTTGAAACTAACAGCAATTACCCCAGAAAGTGTCTGCTAA

>p2_ind1265

ATGCAAATAGACCCCGTTGCATATCATTGTTCCGAGTTGAAGTTCGTGCTTGTACCCGAAGAGTCGGGAAAGTTAT
CCCCGACGGAAATAGGATTCCATTTTCAGATTGGGTGCCGCGTACATGACAGAAGACCCCGCTCTGAGTCAGTGA
TGACGCACCAACGCAGATATCGATGTCTCACGTCTACCTTATTACTTGCCTTGACCGTCGGCACCCGATATTTGTCA
ACTGGGAATCCTCCTGGACCTATTATCGGTCCTTTGAAACTAACAGCAATTACCCCAGAAAGTGTCTGCTAA

>p2_ind2265

ATGCAAATAGACCCCGTTGCATATCATTGTTCCGAGTTGAAGTTCGTGCTTGTACCCGAAGAGTCGGGAAAGTTAT
CCCCGACGGAAATAGGATTCCATTTTCAGATTGGGTGCCGCGTACATGACAGAAGACCCCGCTCTGAGTCAGTGA
TGACGCACCAACGCAGATATCGATGTCTCACGTCTACCTTATTACTTGCCTTGATCGTCGGCACCCGATATTTGTCA
ACTGGGAATCCTCCTGGACCTATTATCGGTCCTTTGAAACTAACAGCAATTACCCCAGAAAGTGTCTGCTAA

>p2_ind2902

ATGCAAATAGACCCCGTTGCATATCATTGTTCCGAGTTGAAGTTCGTGCTTGTACCCGAAGAGTCGGGAAAGTTAT
CCCCGACGGAAATAGGATTCCATTTTCAGACTGGGCGCCGCGTACATGACAGAAGACCCCGCTCTGAGTCAGTGA
TGACGCACCAACGCAGATATCGATGTCTCACGTCTACCTTATTACTTGCCTTGATCGTCGGCACCCGATATTTGTCA
ACTGGGAATCCTCCTGGACCTATTATCGGTCCTTTGAAACTAACAGCAATTACCCCAGAAAGTGTCTGCTAA

>p2_ind4400

ATGCAAATAGACCCCGTTGCATATCATTGTTCCGAGTTGAAGTTCGTGCTTGTACCCGAAGAGTCGGGAAAGTTAT
CCCCGACGGAAATAGGATTCCATTTTCAGATTGGGTGCCGCGTACATGACAGAAGACCCCGCTCTGAGTCAGTGA
TGACGCACCAACGCAGATATCGATGTCTCACGTCTACCTTATTACTTGCCTTGATCGTCGGCACCCGATATTTGTCA
ACTGGGAATCCTCCTGGACCTATTATCGGTCCTTTGAAACTAACGGCAATTACCCCAGAAAGTGTCTGCTAA

>p2_ind935

ATGCAAATAGACCCCGTTGCATATCATTGTTCCGAGTTGAAGTTCGTGCTTGTACCCGAAGAGTCGGGAAAGTTAT
CCCCGACGGAAATAGGATTACATTTTCAGATTGGGCGCCGCGTACATGACAGAAGACCCCGCTCTGAGTCAGTGA
TGACGCACCAACGCAGATATCGATGTCTCACGTCTACCTTATTACTTGCCTTGATCGTCGGCACCCGATATTTGTCA
ACTGGGAATCCTCCTGGACCTATTATCGGTCCTTTGAAACTAACAGCAATTACCCCAGAAAGTGTCTGCTAA
