# Supplementary material for: GeneScanner: profiling genetic variation across bacterial populations
Source: Microb Genom. 2026 Jun 2;12(6):001714. doi: 10.1099/mgen.0.001714 (PMC13229410; doi:10.1099/mgen.0.001714)
Supplement: Supplementary Material 8. [file mgen-12-01714-s008.pdf]

>ref

TCACTTTCGTTATCCAGATCGGTCATCTGTGGTAAGACTAGAGAAACCGTTCTACTCTATATCCTCATACACAATCG
CCTAGCTGCAGCCCAGGTCTA

>p1_ind1994

TCACTTTCGTTATCCAGATCGGTCATCTGTGGTAAGACTAGAGAAACCGTTCTACTCTATATCCTCATACACAATCG
CCTAGCTGCAGCCCAGGTCTA

>p1_ind2665

TCACTTTCGTTATCCAGATCGGTCATCTGTGGTAAGACTAGAGAAACCGTTCTACTCTATATCCTCATACACAATCG
CCTAGCTGCAGCCCAGGTCTA

>p1_ind3972

TCACTTTCGTTATCCAGATCGGTCATCTGTGGTAAGACTAGAGAAACCGTTCTACTCTATATCCTCATACACAATCG
CCTAGCTGCGGCCCAGGTCTA

>p1_ind931

TCACTTTCGTAATCCAGATCGGTCATCCGCGGTAAGACTAGAGAAACCGTTCTACTCTATATCCTCATACACAATCG
CCTAGCTGCAGCCCAGGTCTA

>p1_ind4211

TCACTTTCGTTATCCAGATCGGTCATCTGCGGTAAGACTAGAGAAACCGTTCTACTCTATATCCTCATACACAATCG
CCTAGCTGCAGCCCAGGTCTA

>p1_ind4169

TCACTTTCGTTATCCAGATCGGTCATGTGTGGTAAGACTAGAGGAACCATTCTACTCTATATCCTCATACACAATCG
CCTAGCTGCAGCCGAGGTCTA

>p1_ind1458

TCACTTTCGTTATCCAGATCGGTCATCTACGGTAAGACTAGAGAAACCGTTCTACTCTATATCCTCATACACAATCG
CCTAGCTGCAGCCCAGGTCTA

>p1_ind1648

TCACTTTCGTTATCCAGATCGGTCATCCGCGGTAAGACTAGAGAAACCGTTCTACTCTATATCCTCACACACAATCG
CCTAGCTGCAGCCCAGGTCTA

>p1_ind1555

TCACTTTCGTTATCCAGATCGGTCATCCGCGGTAAGACTAGAGGAACCATTCTACTCTATATCCTCATACACAATCG
CCTAGCTGCAGCCAAGGTCTA

>p1_ind1751

TCACTTTCGTTATCCAGATCGGTCATCTGTGGTAAGACTAGAGGAACCATTCTACTCTATATCCTCATACACAATCG
CCTAGCTGCAGCCCAGGTCTA

>p1_ind5163

TCACTTTCGTTATCCAGATCGGTCATCCGCGGTAAGACTAGAGAAACCGTTCTACTCTATATCCTCATACACAATCG
CCTAGCTGCAGCCCAGGTCTA

>p1_ind5131

TCACTTTCGTTATCCAGATCGGTCATCTGTGGTAAGACTAGAGGAACCATTCTACTCTATATCCTCATACACAATCG
CCTAGCTGCAGCCCAGGTCTA

>p1_ind787

TCACTTTCGTTATCCAGATCGGTCATCTGTGGTAAGACTAGAGAAACCGTTCTACTCTATATCCTCATACACAATCG
CCTAGCTGCGGCCCAGGTCTA

>p1_ind2611

TCACTTTCGTTATCCAGATCGGTCATCTGCGGTAAGACTAGAGGAACTGTTCTACTCTATATCCTCATACACAATGG
CCTAGCTGCAGCCCAGGTCTA

>p1_ind5087

TAATTTTCGTTATCCAGATCGGTCATCTGTGGTAAGATTAGAGTAACCGTTCTACTCTATATCCTCATACACAATCGC
CTAGCTGCAGCCCAGGTCTA

>p1_ind2287

TCACTTTCGTTATCCAGATCGGTCATCTGTGGTAAGACTAGATAAACCGTTCTACTCTATATCCTCATACACAATCGC
CTAGCTGCAGCCCAGGTCTA

>p1_ind2428

TCACTTTCGTTATCCAGATCGGTCATCCGCGGTAAGACTAGAGAAACCGTTCTACTCTAAATCCTCATACACAATCG
CCTAGCTGCAGCCCAGGTCTA

>p1_ind3761

TCACTTTCGTTATCCAGATCGGTCATCCGCGGTAAGACTAGAGAAACCGTTCTACTCTATATCCTCATACACAATCG
CCTAGCTGCAGCCCAGGTCTA

>p1_ind4030

TCACTTTCGTTATCCAGATCGGTCATCTGTGGTAAGACTAGAGAAACCGTTCTACTCTATATCCTCATACACAATCG
CCTAGCTGCAGCCCAGGTCTA

>p1_ind4959

TCACTTTCGTTATCCAGATCGGTCATCTGTGGTAAGACTAGAGAAACCGTTCTACTCTATATCCTCATACACAATCG
CCTAGCTGCAGCCCAGGTCTA

>p1_ind4815

TCACTTTCGTTATCCAGATCGGTCATCTGTGGTAAGACTAGAGAAACCGTTCTACTCTATATCCTCATACACAATCG
CCTAGCTGCAGCCCAGGTCTA

>p1_ind125

TCACTTTCGTTATCCAGATCGGTAATCTGCGGTAAGACTAGAGAAACCGTTCTACTCTATATCCTCATACACCATCG
CCTAGCTGCAGCCCAGGTCTA

>p1_ind2824

TCACTTTCGTTATCCAGATCGGTCATCTGTGGTAAGCCTAGAGGAACCATTCTACTCTATATCCTCATTCACAATCGC
CTAGCTGCAGCCCAGGTCTA

>p1_ind4843

TCACTTTCGTTATCCAGATCGGTCATCTGTGGTAAGACTTGAGAATCCGTTCTACTCTATATCCTCATACACAATCGC
CTAGCTGCAGCCCAGGTCTA

>p1_ind337

TCACTATCGTTATCCAGATCGGTCATCTGTGGTAAGACTAGTGAAACCGTTCTACTCTATATCCTCATACACAATCG
CCTAGCTGCAGCCCAGGTCTA

>p1_ind5056

TCACTTTCGTTATCCAGATCGGTTATCCGCGGTAAGACTAGAGAAACCGTTCTACTCTAAATCCTCATACACAATCG
CCTAGCTGCAGCCCAGGTCTA

>p1_ind766

TCACTTTCGTTATCCAGATCGGTCATCTGTGGTAAGACTAGAGAAACCGTTCTACTCTATATCCTCATACACAATCG
CCTAGCTGCAGCCCAGGTCTA

>p1_ind1362

TCACTTTCGTTATCCAGATCGGTCATCTGTGGTAAGACTAGAGGAACCATTCTACTCTATATCCTCATACACAATCG
CCTAGCTGCAGCCCAGGTCTA

>p1_ind3345

TCACTTTCGTTATCCAGATCGGTCATCTGTGGTAAGACTAGAGAAACCGTTCTACTCTATATCCTCATACACAATCG
CCTAGCTGCAGCCCAGGTCTA

>p1_ind2058

TCACTTTCGTTATCCAGATCGGTCATCTGTGGTAAGATTAGAGTAACCGTTCTACTCTATATCCTCATACACAATCGC
CTAGCTGCAGCCCAGGTCTA

>p1_ind848

TCACTTTCGTTATCCAGATCGGTCATCTGTGGTAAGACTAGAGAAACCGTTCTACTCTATATCCTCATACACAATCG
CCTAGCTGCAGGCCAGGTCTA

>p1_ind4564

TCACTTTCGTTATCCCGATCGGTCATCTGTGGTAAGACTAGAGAAACCGTTCTACTCTATATCCTCATACACAATCG
CCTAGCTGCAGCCCAGGTCTA

>p1_ind1338

ACACTTTCGTTATCCAGATCGGTCATCCGCGGTAAGCCTAGAGAAACCGTTCTACTCTATATCCTCATACACAATCG
CCTAGCTGCAGCCCAGGTCTA

>p1_ind502

TCACTTTCGTTATCCAGATCGGTCATCCGCGGTAAGACTAGAGAAACCGTTCTACTCTATATCCTCATACACAATCG
CCTAGCTGCAGCCCAGGTCTA

>p1_ind910

TCACTTTCGTTATCCAGATCGGTCATCTACGGTAAGACTAGAGAAACCGTTCTACTCTATATCCTCATACACAATCG
CCTAGCTGCAGCCCAGGTCTA

>p1_ind1847

TCACTTTCGTTATCCAGATCGGTCATCTGCGGTAAGACTAGAGAAACCGTTCTACTCTATATCCTCATACACAATCG
CCTAGCTGCAGCCCAGGTCTA

>p1_ind3020

TCACTTTCGTTATCCAGATCGGTCATCTGTGGTAAGACTAGAGAAACCGTTCTACTCTATATCCTCATACACAATCG
CCTAGCTGCAGCCCAGGTCTA

>p1_ind2195

TCACTTTCGTTATCCAGATCGGTCATCTGTGGTAAGACTAGAGAAACCGTTCTACTCTATATCCTCATACACAATCG
CCTAGCTGCAGGCCAGGTCTA

>p1_ind3201

TCACTTTCGTTATCCAGATCGGTCATCTGTGGCAAGACTAGAGAAACCGTTCTACTCTATATCCTCATACACAACCG
CCTAGCTGCAGCCCAGGTCTA

>p1_ind531

TCATTTTCGTTATCCAGATCGGTCATCTGTGGTAAGACTAGAGGAACCATTCTACTCTATATCCTCATACACAATCG
CCTAGCTGCAGCCCAGGTCTA

>p1_ind2763

TCACTTTCGTTATCCAGATCGGTCATCTGCGGTAAGACTAGAGGAACTGTTCTACTCTATATCCTCATACACAATCG
CCTAGCTGCAGCCCAGGTCTA

>p1_ind4202

TCACTTTCGTTATCCAGATCGGTCATCCGCGGTAAGACTAGAGGAACCGTTCTACTCTATATCCTCATACACAATCG
CCTAGCTGCAGCCCAGGTCTA

>p1_ind1649

TCACTTTCGTTATCCAGATCGGTCATCCACGGTAAGACTAGAGAAACCGTTCTACTCTATATCCTCATACACAATCG
CCTAGCTGCAGCCCAGGTCTA

>p1_ind373

TCACTTTCGTTATCCAGATCGGTCATCTGTGGTAAGACTAGAGAAACCGTTCTACTCTATATCCTCATACACAATCG
CCTAGCTGCAGCCCAGGTCTA

>p1_ind56

TCACTTTCGTTATCCAGATCGGTCATCTGTGGTAAGACTAGAGAAACCGTTCTACTCTATATCCTCATACACAATCG
CCTAGCTGCAGCCCATGTCTA

>p1_ind3562

TCACTTTCGTTATCCAGATCGGTCATCCGCGGTAAGACTAGAGAAACCGTTCTACTCTATATCCTCATACACAATCG
CCTAGCTGCAGCCCAGGTCTA

>p1_ind2213

TCACTTTCGTTATCCAGATCGGTCATCCGCGGTAAGACTAGAGAAACCGTTCTACTCTATATCCTCATACACAATCG
CCTAGCTGCAGCCCAGGTCTA

>p1_ind1459

TCACTTTCGTTATCCAGATCGGTCATCCGCGGTAAGACTAGAGAAACCGTTCTACTCTATATCCTCATACACAATCG
CCTAGCTGCAGCCCAGGTCTA

>p1_ind4700

ACACTTTCGTTATCCAGATCGGTCATCCGCGGTAAGCCTAGAGAAACCGTTCTACTCTATATCCTCATACACAATCG
CCTAGCTGCAGCCCAGGTCTA

>p1_ind1952

TCATTTTCGTTATCCAGATCGGTCATCTGTGGTAAGACTAGAGGAACCGTTCTACTCTATATCCTCATACACAATCG
CCTAGCTGCAGCCCAGGTCTA

>p1_ind3497

TCATTTTCGTTATCCAGATCGGTAATCTGCGGTAAGACTAGAGAAACCGTTCTACTCTATATCCTCATACACAATCG
CCTAGCTGCAGCCCAGGTCTA

>p1_ind4942

TCACTTTCGTTATCCAGATCGGTCATCTGTGGTAAGACTAGAGGAACCATTCTACTCTATATCCTCATACACAATCG
CCTAGCTGCAGCCCAGGTCTA

>p1_ind1503

TCACTTTCGTAATCCAGATCGGTCATCCGCGGTAAGACTAGAGAAACCGTTCTACTCTATATCCTCATACACAATCG
CCTAGCTGCAGCCCAGGTCTA

>p1_ind2292

TCATTTTCGTTATCCAGATCGGTCATCTGTGGTAAGACTAGAGGAACCATCATACTCTATATCCTCATACACAATCG
CCTAGCTGCAGCCCAGGTCTA

>p1_ind2000

TCACTTTCGTTATCCAGATCGGTCATCTGTGGCAAGACTAGAGAAACCGTTCTACTCTATATCCTCATACACAACCG
CCTAGCTGCAGCCCAGGTCTA

>p1_ind888

TCACTTTCGTTATCCAGATCGGTCATCTGTGGTAAGACTTGAGAATCCGTTCTACTCTATATCCTCATACACAATCGC
CTAGCTGCAGCCCAGGTCTA

>p1_ind3832

TCACTTTCGTAATCCAGATCGGTCATCCGCGGTAAGACTAGAGAAACCGTTCTACTCTATATCCTCATACACAATCG
CCTAGCTGCAGCCCAGGTCTA

>p1_ind322

TCACTTTCGTTATCCAGATCGGTCATCCGCGGTAAGACTAGAGAAACCGTTCTACTCTATATCCTCATACACAATCG
CCTCGCTGCAGCCCAGGTCTA

>p1_ind302

ACACTTTCGTTATCCAGATCGGTCATCCGCGGTAAGACTAGAGAAACCGTTCTACTCTATATCCTCATACACAATCG
CCTAGCTGCAGCCCAGGTCTA

>p1_ind1816

TCACTTTCGTTATCCAGATCGGTCATCTGTGGCAAGACTAGAGAAACCGTTCTACTCTATATCCTCATACACAACCG
CCTAGCTGCAGCCCAGGTCTA

>p1_ind854

TCACTTTCGTTATCCAGATCGGTCATCTGTGGTAAGACTAGAGAAACCGTTCTACTCTATATCCTCATACACAATCG
CCTCGCTGCAGCCCAGGTCTA

>p1_ind2063

TCACTTTCGTTATCCAGATCGGTCATCCGCGGTAAGACTAGAGAAATCGTTCTACTCTATATCCTCATACACAATCG
CCTAGCTGCAGCCCAGGTCTA

>p1_ind2666

TCACTTTCGTAATCCAGATCGGTCATCCGCGGTAAGACTAGAGAAACCGTTCTACTCTATATCCTCATACACAATCG
CCTAGCTGCAGCCCAGGTCTA

>p1_ind2976

TCACTTTCGTTATCCAGATCGGTCATCTGCGGTAAGACTAGAGAAACCGTTCTACTCTATATCCTCATACACAATCG
CCTAGCTGCAGCCCAGGTCTA

>p1_ind3280

TCACTATCGTTATCCAGATCGGTCATCTGTGGTAAGATTAGAGAAACCGTTCTACTCTATATCCTCATACACAATCG
CCTAGCTGCAGCCCAGGTCTA

>p1_ind1949

TCACTTTCGTTATCCAGATCGGTCATCCGCGGTAAGACTAGAGCAACCGTTCTACTCTATATCCTCATACACAATCG
CCTAGCTGCAGCCCAGGTCTA

>p1_ind3384

TCACTTTCGTTATCCAGATCGGTCATCTGTGGCAAGACTAGAGAAACCGTTCTACTCTATATCCTCATACACAACCG
CCTAGCTGCAGCCCAGGTCTA

>p1_ind2496

TCACTTTCGTTATCCAGATCGGTCATCCGCGGTAAGACTAGAGAAACCGTTCTACTCTATATCCTCATACACAATCG
CCTAGCTGCAGCCCAGGTCTA

>p1_ind893

TCACTTTCGTTATCCAGATCGGTCATCCGCGGTAAGACTAGAGAAACCGTTCTACTCTATATCCTCATACACAATCG
CCTAGCTGCAGCCCAGGTCTA

>p1_ind982

TCACCTTCGTTATCCAGATCGGTCATCTGTGGTAAGACTAGAGAAACCGTTCTACTCTATATCCTCATACACAATCG
CCTAGCTGCAGCCCAGGTCTA

>p1_ind2960

TCACTTTCGTTATCCAGATCGGTCATCCGCGGTAAGACTAGAGGAACCATTCTACTCTATATCCTCATACACAATCG
CCTAGCTGCAGCCAAGGTCTA

>p1_ind1509

TCACTTTCGTTATCCAGATCGGTCATCCGCGGTAAGACTAGAGGAACCATTCTACTCTATATCCTCATACACAATCG
CCTAGCTGCAGCCAAGGTCTA

>p1_ind172

TCACTTTCGTTATCCAGATCGGTCATCCGCGGTAAGACTAGAGAAACCGTTCTACTCTATATCCTCATACACAATCG
CCTAGCTGCAGCCCAGGTCTA

>p1_ind1514

TCACTTTCGTTATCCAGATCGGTCATCCGCGGTAAGACTAGAGGAACCGTTCTACTCTATATCCTCATACACAATCG
CCTAGCTGCAGCCCAGGTCTA

>p1_ind2459

TCACTTTCGTTATCCAGATCGGTCATCCGCGGTAAGACTAGAGAAACCGTTCTACTCTATATCCTCATACACAATCG
CCTAGCTGCAGCCCAGGTCTA

>p1_ind2202

TCACTTTCGTTATCCAGATCGGTCATCCGCGGTAAGACTAGAGAAACCGTTCTACTCTATATCCTCATACACAATCG
CCTAGCTGCAGCCCAGGTCTA

>p1_ind1017

TCACTTTCGTTATCCAGATCGGTCATCCGCGGTAAGACTAGAGAAACCGTTCTACTCTATATCGTCATACACAATCG
CCTAGCTGCAGCCCAGGTCTA

>p1_ind3155

TCACTTTCGTTATCCAGATCGGTCATCTGTGGCAAGACTAGAGAAACCGTTCTACTCTATATCCTCATACACAACCG
CCTAGCTGCAGCCCAGGTCTA

>p1_ind4464

TCACTTTCGTTATCCAGATCGGTCATCTGTGGTAAGACTAGAGAAACCGTTCTACTCTATATCCTCATACACAATCG
CCTAGCTGCAGCCCAGGTCTA

>p1_ind4761

TCACTTTCGTTATCCAGATCGGTCATCCGCGGTAAGACTAGAGAAACCGTTCTACTCTATATCCTCATACACAATCG
CCTCGCTGCAGCCCAGGTCTA

>p1_ind154

TCACTTTCGTTATCCAGATCGGTCATCTGTGGTAAGACTAGAGGAACCATCATACTCTATAACCTCATACACAATCG
CCTAGCTGCAGCCCAGGTCTA

>p1_ind2619

TCACTTTCGTTATCCAGATCGGTCATCTGTGGCAAGACTAGAGGAACTGTTCTACTCTATATCCTCATACATAATCG
CCTAGCTGCAGCCCAGGTGTA

>p1_ind3250

TCACTTTCGTTATCCAGATCGGTCATGTGTGGTAAGACTAGAGGAACCATTCTACTCTATATCCTCATACACAATCG
CCTAGCTGCAGCCCAGGTCTA

>p1_ind5044

TCACTTTCGTTATCCAGATCGGTCATCTGTGGTAAGACTAGAGAAACCGTTCTACTCTATATCCTCATACACAATCG
CCTAGCTGCAGCCCAGGTCTA

>p1_ind1472

TCACTTTCGTTATCCAGATCGGTCATCTGTGGTAAGACCAGAGAAACCGTTTTACTCTATATCCTCATACACAATCG
CCTAGCTGCAGCCCAGGTCTA

>p1_ind2898

TCACTTTCGTTATCCAGATCGGTCATCCGCGGTAAGACTAGAGAAACCGTTCTACTCTATATCCTCATACACAATCG
CCTAGCTGCAGCCCAGGTCTA

>p1_ind2797

TCACTTTCGTTATCCAGATCGGTCATCTGTGGCAAGACTAGAGAAACCGTTCTACTCTATATCCTCATACACAACCG
CCTAGCTGCAGCCCAGGTCTA

>p1_ind129

TCACTTTCGTTATCCAGATCGGTCATCTGTGGTAAGACTAGAGAAACCGTTCTACTCTATATCCTCATACACAATCG
CCTAGCTGCAGCCCAGGTCTA

>p1_ind1155

TCACTTTCGTTATCCAGATCGGTCATCTGCGGTAAGACTAGAGAAACCGTTCTACTCTATATCCTCATACACAATCG
CCTAGCTGCAGCCCAGGTCTA

>p1_ind2917

TCACTTTCGTAATCCAGATCGGTCATCCGCGGTAAGACTAGAGAATCCGTTCTACTCTATATCCTCATACACAATCG
CCTAGCTGCAGCCCAGGTCTA

>p1_ind3809

TCACTTTCGTTATCCAGATCGGTCATGTGTGGTAAGACTAGAGGAACCATTCTACTCTATATCCTCATACACAATCG
CCTAGCTGCAGCCCAGGTCTA

>p1_ind1521

TCACTTTCGTTATCCAGATCGGTCATCTGCGGTAAGACTAGAGAAACCGTTCTACTCTATATCCTCATACACAATCG
CCTAGCTGCAGCCCAGGTCTA

>p1_ind5021

TCACTTTCGTTATCCAGATCGGTCATCCGCGGTAAGACTAGAGCAACCGTTCTACTCTATATCCTCATACACAATCG
CCTAGCTGCAGCCCAGGTCTA

>p1_ind315

TCACTTTCGTTATCCAGATCGCTCATCTGTGGTAAGACTAGAGAAACCGTTCTACTCTATATCCTCATACACAATCGC
CTAGCTGCAGCCCAGGTCTA

>p1_ind722

TCACTTTCGTTATCCAGATCGGTCATCCGCGGTAAGACTAGAGAAACCGTTCTACTCTATATCCTCATACACAATCG
CCTAGCTGCAGCCCAGGTCTA

>p1_ind2999

TCACTTTCGTTATCCAGATCGGTCATCTGCGGTAAGACTAGAGAAACCGTTCTACTCTATATCCTCATACACAATCG
CCTAGCTGCAGCCCAGGTCTA

>p1_ind2027

TCATTTTCGTTATCCAGATCGGTCATCTGTGGTAAGACTAGAGGAACCGTTCTACTCTATATCCTCATACACAATCG
CCTAGCTGCAGCCCAGGTCTA

>p1_ind5127

TCACTTTCGTTATCCAGATCGGTCATCTGTGGTAAGACTAGAGAAACCGTTCTACTCTATATCCTCATACACAATCG
CCTAGCTGCAGCCCAGGTCTA

>p1_ind4844

TCACTTTCGTTATCCAGATCGGTCATCTGTGGCAAGACTAGAGAAACCGTTCTACTCTATATCCTCATACACAACCG
CCTAGCTGCAGCCCAGGTCTA

>p1_ind2788

TCACTTTCGTTATCCAGATCGGTCATCTGTGGTAAGACTAGAGAAACCGTTCTACTCTATATCCTCATACACAATCG
CCTAGCTGCAGCCCAGGTCTA

>p1_ind2799

TCACTTTCGTTATCCAGATCGGTCATCTGTGGTAAGACTAGAGAAACCGTTCTACTCTATATCCTCATACACAATCG
CCTAGCTGCAGCCTAGGTCTA

>p1_ind2860

TCACTTTCGTTATCCAGATCGGTCATCTGTGGTAAGACTAGAGAAACCGTTCTACTCTATATCCTCATACACAATCG
CCTAGCTGCAGCCCAGGTCTA

>p1_ind1268

TCACTTTCGTTATCCAGATCGGTCATCCGCGGTAAGACTAGAGAAACCGTTCTACTCTATATCCTCATACACAATCG
CCTAGCTGCAGCCCAGGTCTA

>p1_ind2838

TCACTTTCGTTATCCAGATCGGTCATCTGTGGTAAGACTAGAGAAACCGTTCTACTCTATATCCTCATACACAATCG
CCTAGCTGCAGCCCAGGTCTA

>p1_ind3696

TCACTTTCGTAATCCAGATCGGTCATCCGCGGTAAGACTAGAGAAACCGTTCTACTCTATATCCTCATACACAATCG
CCTAGCTGCAGCCCAGGTCTA

>p1_ind1040

TCACTTTCGTAATCCAGATCGGTCATCCGCGGTAAGACTAGAGAAACCGTTCTACTCTATATCCTCATACACAATCG
CCTAGCTGCAGCCCAGGTCTA

>p1_ind3471

TCACTTTCGTTATCCAGATCGGTCATCTGTGGTAAGACTAGAGAAACCGTTCTACTCTATATCCTCATACACAATCG
CCTAGCTGCAGCCCAGGTCTA

>p1_ind1043

TCACTTTCGTTATCCAGATCGGTCATCCGCGGTAAGACTAGAGGAACCATTCTACTCTATATCCTCATACACAATCG
CCTAGCTGCAGCCAAGGTCTA

>p1_ind1453

TCACTTTCGTAATCCAGATCGGTCATCCGCGGTAAGACTAGAGAAACCGTTCTACTCTATATCCTCATACACAATCG
CCTAGCTGCAGCCCAGGTCTA

>p1_ind747

TCATTTTCGTTATCCAGATCGGTCATCTGTGGTAAGCCTAGAGGAACCATTCTACTCTATATCCTCATTCACAATCGC
CTAGCTGCAGCCCAGGTCTA

>p1_ind2098

TCACTTTCGTTATCCAGATCGGTCATCTGTGGTAAGACTAGAGACACCGTTCTACTCTATATACTCATACACAATCG
CCTAGCTGCAGGCCAGGTCTA

>p1_ind2727

TCACTTTCGTTATCCAGATCGGTCATCCGCGGTAAGACTAGAGCAACCGTTCTACTCTATATCCTCATACACAATCG
CCTAGCTGCAGCCCAGGTCTA

>p1_ind561

TCACTTTCGTTATCCAGATCGGTCATCTGTGGTAAGACTAGAGGAACCATTCTACTCTATATCCTCATACACAATCG
CCTAGCTGCAGCCCAGGTCTA

>p1_ind4873

TCACTTTCGTTATCCAGATCGGTCATCCGCGGTAAGACTAGAGAAACCGTTCTACTCTATGTCCTCATACACAATCG
CCTCGCTGCAGCCCAGGTCTA

>p1_ind4838

TCACTTTCGTTATCCAGATCGGTCATCTGTGGTAAGACTAGAGAAACCGTTCTACTCTATATCCTCATACACAATCG
CCTAGCTGCAGCCCAGGTCTA

>p1_ind4605

TCACTTTCGTTATCCAGATCGGTCATCTGTGGTAAGACTAGAGACACCGTTCTACTCTATATCCTCATACACAATCG
CCTAGCTGCAGGCCAGGTCTA

>p1_ind4362

TCATTTTCGTTATCCAGATCGGTCATCTGTGGTAAGACTAGAGGAACCATCATACTCTATAACCTCATACACAATCG
CCTAGCTGCAGCCCAGGTCTA

>p1_ind3044

TCACTTTCGTTATCCAGATCGGTCATCCGCGGTAAGACTAGAGAAACCGTTCTACTCTATATCCTCATACACAATCG
CCTAGCTGCAGCCCAGGTCTA

>p1_ind2164

TCACTTTCGTTATCCAGATCGGTCATCCGCGGTAAGACTAGAGAAACCGTTCTACTCTATATCCTCATACACAATCG
CCTCGATGCAGCCCAGGTCTA

>p1_ind2768

TCACTTTCGTTATCCAGATCGGTCATCTGTGGTAAGACTTGAGAATCCGTTCTACTCTATATCCTCATACACAATCGC
CTAGCTGCAGCCCAGGTCTA

>p1_ind2505

TCACTTTCGTTATCCAGATCGGTCATGTGTGGTAAGACTAGAGAAACCGTTCTACTCTATATCCTCATACACAATCG
CCTAGCTGCAGCCCAGGTCTA

>p1_ind2476

TCACTTTCATTATCCAGATCGGTCATCTGCGGTAAGACTAGAGAAACCGTTCTACTCTATATCCTCATACACAATCG
CCTAGCTGCAGCCCAGGTCTA

>p1_ind4165

TCACTTTCGTTATCCAGATCGGTCATGTGTGGTAAGACTAGAGGAACCATTCTACTCTATATCCTCATACACAATCG
CCTAGCTGCAGCCCAGGTCTA

>p1_ind4334

TCACTTTCGTTATCCAGATCGGTCATCCGCGGTAAGACTAGAGAAACCGTTCTACTCTATATCCTCATACACAATCG
CCTAGCTGCAGCACAGGTCTA

>p1_ind2841

TCACTTTCGTTATCCAGATCGGTCATCCGCGGTAAGACTAGAGAAACCGTTCTACTCTATATCCTCATACACAATCG
CCTAGCTGCAGCCCAGGTCTA

>p1_ind5149

TCACTTTCGTTATCCAGATCGGTCATGTGTGGTAAGACTAGAGGAACCATTCTACTCTATATCCTCATACACAATCG
CCTAGCTGCAGCCCAGGTCTA

>p1_ind4855

TCACTTTCGTTATCCAGATCGGCCATCTGCGGTAAGACTAGAGAAACCGTTCTACTCTATATCCTCATACACAATCG
CCTAGCTGCAGCCCAGGTCTA

>p1_ind2334

TCACTTTCGTTATCCAGATCGGTCATCCGCGGTAAGACTAGAGAAACCGTTCTACTCTATATCCTCATACACAATCG
CCTCGCTGCAGCCCAGGTCTA

>p1_ind2610

TCACTTTCGTTATCCAGATCGGTCATCTGTGGTAAGACTAGAGAAACCGTTCTACTCTATATCCTCATACACAATCG
CCTAGCTGCAGCCCAGGTCTA

>p1_ind4816

TCACTTTCGTTATCCAGATCGGTCATCTGTGGTAAGACTAGAGAAACCGTTCTACTCTATATCCTCATACACAATCG
CCTAGCTGCAGCCCAGGTCTA

>p1_ind2617

TCACTTTCGTTATCCAGATCGGTCATCCGCGGTAAGACTAGAGAAACCGTTCTACTCTATATCCTCATACACAATCG
CCTAGCTGCAGCCCAGGTCTA

>p1_ind1139

TCCCTTTCGTTATCCAGATCGGTCATCTACGGTAAGACTAGAGAAACCGTTCTACTCTATATCCTCATACACAATCG
CCTAGCTGCAGCCCAGGTCTA

>p1_ind2500

TCACTTTCGTTATCCAGATCGGTCATCCGCGGTAAGACTAGAGAAACCGCTCTACTCTATATCCTCATACACAATCG
CCTAGCTGCAGCCCAGGTCTA

>p1_ind3049

TCACTTTCGTTATCCAGATCGGTCATCTGTGGCAAGACTTGAGAATCCGTTCTACTCTATATCCTCATACACAATCGC
CTAGCTGCAGCCCAGGTCTA

>p1_ind371

TCACTTTCGTTATCCAGATCGGTCATCTACGGTAAGACTAGAGAAACCGTTCTACTCTATATCCTCATACACAATCG
CCTAGCTGCAGCCCAGGTCTA

>p1_ind3178

TCACTTTCGTTATCCAGATCGGTCATCTGTGGTAAGACTAGAGGAACCATTCTACTCTATATCCTCATACACAATCG
CCTAGCTGCAGCCCAGGTCTA

>p1_ind2250

TCACTTTCGTTATCCAGATCGGTCATCCGCCGTAAGACTAGAGAAACCGTTCTACTCTATATCCTCATACACAATCG
CCTAGCTGCAGCCCAGGTCTA

>p1_ind3247

TCACTTTCGTTATCCAGATCGGTCATCCGCGGTAAGACTAGAGGAACCGTTCTACTCTATATCCTCATACACAATCG
CCTAGCTGCAGCCCAGGTCTA

>p1_ind3786

TCACTTTCGTTATCCAGATCGGTCATCTGTGGTAAGACTAGAGAAACCGTTCTACTCTATATCCTCATACACAATCG
CCTAGCTGCAGCCCAGGTCTA

>p1_ind2077

TCACTTTCGTTATCCAGATCGGTCATCCGCGGTAAGACTAGAGAAACCGTTCTACTCTATATCCTCATACACAATCG
CCTAGCTGCAGCCCAGGTCTA

>p1_ind2362

TCACTTTCGTTATCCAGATCGGTCATCTGTGGTAAGACTAGAGAAACCGTTCTACTCTATATCCTCATACACAATCG
CCTAGCTGCAGCCTAGGTCTA

>p1_ind628

TCACTTTCGTTATCCAGATCGGTCATCTACGGTAAGACTAGAGAAACCGTTCTACTCTATATCCTCATACACAATCG
CCTAGCTGCAGCCCAGGTCTA

>p1_ind4065

TCATTTTCGTTATCCAGATCGGTAATCTGCGGTAAGACTAGAGAAACCGTTCTACTCTATATCCTCATACACAATCG
CCTAGCTGCAGCCCAGGTCTA

>p1_ind3512

TCACTTTCGTTATCCAGATCGGTCATCCGCGGTAAGACTAGAGAAACCGTTCTACTCTATATCCTCATACACAATCG
CCTCGCTGCAGCCCAGGTCTA

>p1_ind1174

TCACTTTCGTTATCCAGATCGGTCATCTGTGGCAAGACTAGAGAAACCGTTCTACTCTATATCCTCATACACAACCG
CCTAGCTGCAGCCCAGGTCTA

>p1_ind695

TCCCTTTCGTTATCCAGATCGGTCATCTACGGTAAGACTAGAGAAACCGTTCTACTCTATATCCTCATACACAATCG
CCTAGCTGCAGCCCAGGTCTA

>p1_ind736

TCACTTTCGTTATCCAGATCGGTCATCCGCGGTAAGACTAGAGAAACCGTTCTACTCTATATCCTCATACACAATCG
GCTAGCTGCAGCCCAGGTCTA

>p1_ind1701

TCACTTTCGTTATCCAGATCGGTCATCCGCGGTAAGACTAGAGAAACCGTTCTACTCTATATCCTCATACACAATCG
CCTAGCTGCAGCCCAGGTCTA

>p1_ind1610

TCACTTTCGTTATCCAGATCGGTCATCTGTGGTAAGACTAGAGAAACCGTTCTACTCTATATCCTCATACACAATCG
CCTAGCTGCAGCCCAGGTCTA

>p1_ind1629

TCACTTTCGTTATCCAGATCGGTCATCTGTGGTAAGCCTAGAGGAACCATTCTACTCTATATCCTCATTCACAATCGC
CTAGCTGCAGCCCAGGTCTA

>p1_ind2948

TCACTTTCGTTATCCAGATCGGTCATGTGTGGTAAGACTAGAGGAACCATTCTACTCTATATCCTCATACACAATCG
CCTAGCTGCAGCCCAGGTCTA

>p1_ind1404

TCACTTTCGTTATCCAGATCGGTCATCCGCGGTAAGACTAGAGAAACCGTTCTACTCTATATCCTCATACACAATCG
CCTAGCTGCAGCCCAGGTCTA

>p1_ind237

TCACTTTCGTTATCCAGATCGGTCATCTGCGGTAAGACTAGAGAAACCGTTCTACTCTATATCCTCATACACAATCG
CCTAGCTGCAGCCCAGGTCTA

>p1_ind1754

TCACTTTCGTTATCCAGATCGGTCATCTGTGGTAAGACTAGAGAAACCGTTCTACTCTATATCCTCATACACAATCG
CCTAGCTGCAGCCCAGGTCTA

>p1_ind1048

TCACTTTCGTTATCCAGATCGGTCATCTGTGGTAAGACTAGAGAAACCGTTCTACTCTATATCCTCATACACAATCG
CCTAGCTGCAGCCCAGGTCTA

>p1_ind3523

TCACTTTCGTTATCCAGATCGGTCATCTGTGGTAAGACTAGAGAAACCGTTCTACTCTATATCCTCATACACAATCG
CCTAGCTGCAGCCCAGGTCTA

>p1_ind483

TCACTTTCGTTATCCAGATCGGTCATCCGCGGTAAGACTAGAGAAACCGTTCTACTCTATATCCTCATACACAATCG
CCTAGCTGCAGCCCAGGTCTA

>p1_ind1635

TCACTTTCGTTATCCAGATCGGTCATCTGTGGTAAGACTTGAGAATCCGTTCTACTCTATATCCTCATACACAATCGC
CTAGCTGCAGCCCAGGTCTA

>p1_ind2423

TCACTTTAGTTATCCAGATCGGTCATCTGCGGTAAGACTAGAGAAACCGTTCTACTCTATATCCTCATACACAATCG
CCTAGCTGCAGCCCAGGTCTG

>p1_ind1084

TCACTATCGTTATCCAGATCGGTCATCCGCGGTAAGACTAGAGAAACCGTTCTACTCTATATCCTCATACACAATCG
CCTAGCTGCAGCCCAGGTCTA

>p1_ind4624

TCACTTTCGTTATCCAGATCGGTCATCTGTGGTAAGACTAGAGAAACCGTTCTACTCTATATCCTCATACACAATCG
CCTAGCTGCAGCCCAGGTCTA

>p1_ind3979

TCATTTTCGTTATCCAGATCGGTCATCTGTGGTAAGACTAGAGGAACCGTTCGACTCTATATCCTCATACACAATCG
CCTAGCTGCAGCCCAGGTCTA

>p1_ind75

TCACTTTCGTTATCCAGATCGGTCATCCGCGGTAAGACTAGAGCAACCGTTCTACTCTATATCCTCATACACAATCG
CCTAGCTGCAGCCCAGGTCTA

>p1_ind741

TCACTTTCGTTATCCAGATCGGTCATCTGCGGTAAGACTAGAGAAACCGTTCTACTCTATATCCTCATACACAATCG
CCTAGCTGCAGCCCAGGTCTA

>p1_ind4421

TCACTTTCGTTATCCAGATCGGTCATCTGTGGTAAGACTAGAGAAACCGTTCTACTCTATATCCTCATACACAATCG
CCTAGCTGCAGCCCAGGTCTA

>p1_ind1542

TCACTTTCGTTATCCAGATCGGTCATCTGCGGTAAGACTAGAGAAACCGTTCTACTCTATATCCTCATACACAATCG
CCTAGCTGCAGCCCAGGTCTA

>p1_ind3140

TCACTTTCGTTATCCAGATCGGTCATCCGCGGTAAGACTAGAGAAACCGTTCTACTCTATATCCTCATACACAATCG
CCTAGCTGCAGCCCAGGTCTA

>p1_ind4973

TCACTTTCGTTATCCAGATCGGTCATCTGTGGTAAGACTAGAGAAACCGTTCTACTCTATATCCTCATACACAATCG
CCTAGCTGCAGCCCAGGTCTA

>p1_ind917

TCACTTTCGTTATCCAGATCGGTCATCTGTGGTAAGACTAGAGAAACCGTTCTACTCTATATCCTCATACACAATCG
CCTAGCTGCAGCCCAGGTCTA

>p1_ind4923

TCACTTTCGTTATCCAGATCGGTCATCTACGGTAAGACTAGAGAAACCGTTCTACTCTATATCCTCATACACAATCG
CCTAGCTGCAGCCCAGGTCTA

>p1_ind4911

TCACTTTCGTTATCCAGATCGGTCATCCGCGGTAAGACTAGAGAAACCGTTCTACTCTATATCCTCATACACAATCG
CCTAGCTGCAGCCCAGGTCTA

>p1_ind4245

TCACTTTCGTTATCCAGATCGGTCATCTGTGGTAAGACTAGAGACACCGTTCTACTCTATATACTCATACACAATCG
CCTAGCTGCAGGCCAGGTCTA

>p1_ind1261

TCACTTTCGTTATCCAGATCGGTCATCCGCGGTAAGACTAGAGAAACCGTTCTACTCTATATCCTCATACACAATCG
CCTAGCTGCAGCCCAGGTCTA

>p1_ind3481

ACACTTTCGTTATCCAGATCGGTCATCCGCGGTAAGCCTAGAGAAACCGTTCTACTCTATATCCTCATACACAATCG
CCTAGCTGCAGCCCAGGTCTA

>p1_ind2426

TCACTTTCGTTATCCAGATCGGTCATCCGCGGTAAGACTAGAGAAACCGTTCTACTCTATATCCTCATACACAATCG
CCTAGCTGCAGCCCAGGTCTA

>p1_ind2710

TCACTTTCGTTATCCAGATCGGTCATCTGCGGTAAGACTAGAGGAACTGTTCTACTCTATATCCTCATACACAATCG
CCTAGCTGCAGCCCAGGTCTA

>p1_ind923

TCACTTTCGTTATCCAGATCGGTCATCCGCGGTAAGACTAGAGAAACCGTTCTACTCTATATCCTCACACACAATCG
CCTAGCTGCAGCCCAGGTCTA

>p1_ind3720

TCACTTTCGTTATCCAGATCGGTCATCCGCGGTAAGACTAGAGAAACCGTTCTACTCTATATCCTCATACACAATCG
CCTAGCTGCAGCCCAGGTCCA

>p1_ind567

TCACTTTCGTTATCCAGATCGGTCATCTGTGGTAAGACTAGAGAAACCGTTCTACTCTATATCCTCATACACAATCG
CCTAGCTGCAGCCCAGGTCTA

>p1_ind3139

TCACTTTCGTTATCCAGATCGGTCATCTGTGGTAAGACTAGAGAAACCGTTCTACTCTATATCCTCATACACAATCG
CCTAGCTGCAGCCCAGGTCTA

>p1_ind1398

TCACTTTCGTTATCCAGATCGGTCATGTGTGGTAAGACTAGAGAAACCGTTCTACTCTATATCGTCATACACAATCG
CCTAGCTGCAGCCCAGGTCTA

>p1_ind2540

TCACTTTCGTTATCCATATCGGTCATCTGCGGTAAGACTAGAGAAACCGTTCTACTATATATCCTCATACACAATCG
CCTAGCTGCAGCCCAGGTCTA

>p1_ind394

TCACTTTCGTTATCCAGATCGGTCATCTGTGGTAAGACTAGAGAAACCGTTCTACTCTATATCCTCATACACAATCG
CCTAGCTGCAGCCCAGGTCTA

>p1_ind2852

TCACTTTCGTTATCCAGATCGGTTATCCGCGGTAAGACTAGAGAAACCGTTCTACTCTAAATCCTCATACACAATCG
CCTAGCTGCAGCCCAGGTCTA

>p1_ind2400

TCACTTTCGTTATCCAGATCGGTCATCTGTGGTAAGACTAGAGACACCGTTCTACTCTATATCCTCATACACAATCG
CCTAGCTGCAGGCCAGGTCTA

>p1_ind52

ACACTTTCGTTATCCAGATCGGTCATCCGCGGTAAGCCTAGAGAAACCGTTCTACTCTATATCCTCATACACAATCG
CCTAGCTGCAGCCCAGGTCTA

>p1_ind55

TCACTTTCGTTATCCAGATCGGTCATCTGCGGTAAGACTAGAGAAACCGTTCTACTCTATATCCTCATACACAATCG
CCTAGCTGCAGCCCAGGTCTA

>p1_ind1875

TCATTTTCGTTATCCAGATCGGTCATCTGTGGTAAGACTAGAGGAACCGTTCTACTCTATATCCTCATACACAATCG
CCTAGCTGCAGCCCAGGTCTA

>p1_ind3327

TCACTTTCGTTATCCAGATCGGTCATCCGCGGTAAGACTAGAGAAACCGTTCTACTCTAAATCCTCATACACAATCG
CCTAGCTGCAGCCCAGGTCTA

>p1_ind3660

ACACTTTCGTTATCCAGATCGGTCATCCGCGGTAAGACTAGAGAAACCGTTCTACTCTATATCCTCATACACAATCG
CCTAGCTGCAGCCCAGGTCTA

>p1_ind4547

TCACTTTCGTTATCCAGATCGGTCATCTGTGGTAAGACTAGAGAAACCGTTCTACTCTATATCCTCATACACAATCG
CCTAGCTGCAGCCCAGGTCTA

>p1_ind96

TCACTTTCGTTATCCAGATCGGTCATCTGTGGTAAGACTAGAGAAACCGTTCTACTCTATATCCTCATACACAATCG
CCTAGCTGCAGCCCAGGTCTA

>p1_ind952

TCATTTTCGTTATCCAGATCGGTAATCTGCGGTAAGACTAGAGAAACCGTTCTACTCTATATCCTCATACACAATCG
CCTAGCTGCAGCCCAGGTCTA

>p1_ind885

TCACTTTCGTTATCCAGATCGGTCATCTGCGGTAAGACTAGAGAAACCGTTCTACTCTATATCCTCATACACAATCG
CCTAGCTGCAGCCCAGGTCTA

>p1_ind130

TCACTTTCGTTATCCAGATCGGTCATCTGTGGTAAGACTAGAGAAACCGTTCTACTCTATATCCTCATACACAATCG
CCTAGCTGCAGCCCAGGTCTA

>p1_ind4596

TCACTTTCGTTATCCAGATCGGTCATCCGCGGTAAGACTAGAGCAACCGTTCTACTCTATATCCTCATACACAATCG
CCTAGCTGCAGCCCAGGTCTA

>p1_ind3917

TCACTTTCGTTATCCAGATCGGTCATCCGCGGTAAGACTAGAGAAACCGTTCTACTCTATATCCTCATACACAATCG
CCTCGCTGCAGCCCAGGTCTA

>p1_ind4153

TCACTTTCGTTATCCAGATCGGTCATCTGTGGTAAGACCAGAGAAACCGTTCTACTCTATATCCTCATACACAATCG
CCTAGCTGCAGCCCAGGTCTA

>p1_ind2558

TCACTTTCGTTATCCAGATCGGTCATCCGCGGCAAGACTAGAGAAACCGTTCTACTCTATATCCTCATACACAATCG
CCTAGCTGCAGCCCAGGTCTA

>p1_ind1848

TCACTTTCGTTATCCAGATCGGTCATCCGCGGTAAGACTAGAGAAACCGTTCTACTCTATATCCTCATACACAATCG
CCTAGCTGCAGCCCAGGTCTA

>p1_ind2933

TCACTTTCGTTATCCAGATCGGTCATCCGCGGTAAGACTAGAGATACCGTTCTACTCTGTATCCTCATACACAATCG
CCTAGCTGCAGCCCAGGTCTA

>p1_ind3664

TCACTTTCGTTATCCCGATCGGTCATCTGTGGTAAGACTAGAGAAACCGTTCTACTCTATATCCTCATACACAATCG
CCTAGCTGCAGCCCAGGTCTA

>p1_ind2921

TCACTTTCGTTATCCAGATCGGTCATCTACGGTAAGACTAGAGAAACCGTTCTACTCTATATCCTCATACACAATCG
CCTAGCTGCAGCCCAGGTCTA

>p1_ind4981

TCACTTTCGTTATCCAGATCGGTCATCTACGGTAAGACTAGAGAAACCGTTCTACTCTATATCCTCATACACAATCG
CCTAGCTGCAGCCCAGGTCTA

>p1_ind2777

TCACTTTCGTTATCCAGATCGGTCATCCGCGGTAAGACTAGAGCAACCGTTCTACTCTATATCCTCATACACAATCG
CCTAGCTGCAGCCCAGGTCTA

>p1_ind988

TCACTTTCGTTATCCAGATCGGTCATCTGCGGTAAGACTAGAGAAACCGTTCTACTCTATATCCTCATACACAATCG
CCTAGCTGCAGCCCAGGTCTA

>p1_ind1394

TCACTTTCGTTATCCAGATCGGTCATCTGTGGCAAGACTAGAGAAACCGTTCTACTCTATATCCTCATACACAACCG
CCTAGCTGCAGCCCAGGTCTA

>p1_ind709

TCACTTTCATTATCCAGATCGGTCATCTGCGGTAAGACTAGAGAAACCGTTCTACTCTATATCCTCATACACAATCG
CCTAGCTGCAGCCCAGGTCTA

>p1_ind2819

TCACTTTCGTTATCCAGATCGGTCATCTGTGGTAAGACTAGAGAAACCGTTCTACTCTATATCCTCATACACAATCG
CCTAGCTGCAGCCCAGGTCTA

>p1_ind1416

TCCCTTTCGTTATCCAGATCGGTCATCTACGGTAAGACTAGAGAAACCGTTCTACTCTATATCCTCATACACAATCG
CCTAGCTGCAGCCCAGGTCTA

>p1_ind188

TCACTTTCGTTATCCAGATCGGTCATCCGCGGTAAGACTAGAGAAACCGTTCTACTCTATATCCTCATACACAATCG
CCTAGCTGCAGCCCAGGTCTA

>p1_ind294

TCACTTTCGTTATCCAGATCGGTCATCTGTGGTAAGACTAGAGAAACCGTTCTACTCTATATCCTCATTCACAATCGC
CTAGCTGCAGCCCAGGTCTA

>p1_ind4387

TCACTTTCGTTATCCAGATCGGTCATCTGCGGTAAGACTAGAGAAACCGTTCTACTCTATATCCTCATACACAATCG
CCTAGCTGCAGCCCAGGTCTA

>p1_ind967

TCACTTTCGTTATCCAGATCGGTCATCTGTGGTAAGACTAGAGAAACCGTTCTACTCTATATCCTCATACCCAATCG
CCTAGCTGCAGCCCAGGTCTA

>p1_ind1572

TCATTTTCGTTATCCAGATCGGTAATCTGCGGTAAGACTAGAGAAACCGTTCTACTCTATATCCTCATACACAATCG
CCTAGCTGCAGCCCAGGTCTA

>p1_ind1417

TCACTTTCGTTATCCAGATCGGTCATCCGCGGTAAGACTAGAGAAACCGTTCTACTCTATATCCTCATACACAATCG
CCTAGCTGCAGCCCAGGTCTA

>p1_ind3500

TCACTTTCGTTATCCAGATCGGTCATCCGCGGTAAGACTAGAGAAACCGTTCTACTCTATATCCTCATACACAATCG
CCTCGCTGCAGCCCAGGTCTA

>p1_ind456

TCACTTTCGTTATCCAGATCGGTCATCTGTGGTAAGACTAGAGAAACCGTTCTACTCTATATCCTCATACACAATCG
CCTAGCTGCAGCCCAGGTCTA

>p1_ind6

TCACTTTCGTTATCCAGATCGGTCATCTGTGGTAAGACTAGAGAAACCGTTCTACTCTATATCCTCATACACAATCG
CCTAGCTGCAGCCCAGGTCTA

>p1_ind944

TCACTTTCGTTATCCAGATCGGTCATCTGCGGTAAGACTAGAGAAACCGTTCTACTCTATATCCTCATACACAATCG
CCTAGCTGCAGCCCAGGTCTA

>p1_ind1805

TCACTTTCGTTATCCAGATCGGTCATCTGTGGTAAGACTAGAGAAACCGTTCTACTCTATATCCTCATACACAATCG
CCTAGCTGCAGCCTAGGTCTA

>p1_ind2420

TCACTTTCGTTATCCAGATCGGTCATCCGCGGTAAGACTAGAGAAACCGTTCTACTCTATATCCTCATACACAATCG
CCTACCTGCAGCCCAGGTCTA

>p1_ind4728

TCACTTTCGTTATCCAGATCGGTCATCCGCGGTAAGACTAGAGAAACCGTTCTACTCTGTATCCTCATACACAATCG
CCTAGCTGCAGCCCAGGTCTA

>p1_ind368

TCACTATCGTTATCCAGATCGGTCATCCGCGGTAAGACTAGAGAAACCGTTCTACTCTATATCCTCATACACAATCG
CCTAGCTGCAGCCCAGGTCTA

>p1_ind2595

TCACTTTCGTTATCCAGATCGGTCATCTGTGGTAAGACTTGAGAATCCGTTCTACTCTATATCCTCATACACAATCGC
CTAGCTGCAGCCCAGGTCTA

>p1_ind2887

TCACTTTCGTTATCCAGATCGGTCATCTGTGGTAAGACTAGAGAAACCGTTCTACTCTATATCCTCATACACAATCG
CCTAGCTGCAGCCCAGGTCTA

>p1_ind4257

TCACTTTCGCAATCCAGATCGGTCATCTGTGGTAAGACTAAAGAAACCGTTCTACTCTATATCCTCATACACAATCG
CCTAGCTTCAGCCCAGGTCTA

>p1_ind1880

TCACTTTCGTTATCCAGATCGGTCATCCGCGGTAAGACTAGAGAAACCGTTCTACTCTATATCCTCATACACAATCG
CCTAGCTGCAGCCCAGGTCTA

>p1_ind2704

TCACTTTCGTTATCCAGATCGGTCATCTGCGGTAAGACTAGAGAAACCGTTCTACTCTATATCCTCATACACAATCG
CCTAGCTGCAGCCCAGGTCTA

>p1_ind2081

TCACTATCGTTATCCAGATCGGTCATCTGTGGTAAGATTAGAGAAACCGTTCTACTCTATATCCTCATACACAATCG
CCTAGCTGCAGCCCAGGTCTA

>p1_ind842

TCACTTTCGTTATCCAGATCGGTCATCTGTGGTAAGACTAGAGAAACCGTTCTACTCTATATCCTCATACACAATCG
CCTAGCTGCAGCCCAGGTCTA

>p1_ind1646

TCACTATCGTTATCCAGATCGGTCATCCGCGGTAAGACTAGAGAAACCGTTCTACTCTATATCCTCATACACAATCG
CCTAGCTGCAGCCCAGGTCTA

>p1_ind384

TCACTTTCGTTATCCAGATCGGTCATCCGCGGTAAGACTAGAGAAACCGTTCTACTCTATATCCTCATACACAATCG
CCTAGCTGCAGCCCAGGTCTA

>p1_ind1565

TCACTTTCGTTATCCAGATCGGTCATCCGCGGTAAGACTAGAGAAACCGTTCTACTCTATATCCTCATACACAATCG
CCTAGCTGCAGCCCAGGTCTA

>p1_ind4737

TCACTTTCGTTATCCAGATCGGTCATCTGTGGTAAGACTAGAGAAACCGTTCTACTCTATATCCTCATACACAATCG
CCTAGCTGCAGCCCAGGTCTA

>p1_ind3338

TCACTTTCGTTATCCAGATCGGTCATCTGTGGTAAGACTAGAGAAACCGTTCTACTCTATATCCTCATACACAATCG
CCTCGCTGCAGCCCAGGTCTA

>p1_ind1435

TCACTTTCGTTATCCAGATCGGTCATCCGCGGTAAGACTAGAGAAACCGTTCTACTCTATATCCTCATACACAATCG
CCTAGCTGCAGCCCAGGTCTA

>p1_ind4429

TCACTTTCGTTATCCAGATCGGTCATCCGCGGTAAGACTAGAGAAACCGTTCTACTCTATATCCTCATACACAATCG
CCTAGCTGCAGCCCAGGTCTA

>p1_ind1010

TCACTTTCGTTATCCAGATCGGTCATCCGCGGTAAGACTAGAGAAACCGTTCTACTCTATATCCTCATACACAATCG
CCTAGCTGCAGCCCAGGTCTA

>p1_ind2514

ACACTTTCGTTATCCAGATCGGTCATCCGCGGTAAGCCTAGAGAAACCGTTCTACTCTATATCCTCATACACAATCG
CCTAGCTGCAGCCCAGGTCTA

>p1_ind1449

TCATTTTCGTTATCCAGATCGGTAATCTGCGGTAAGACTAGAGAAACCGTTCTACTCTATATCCTCATACACAATCG
CCTAGCTGCAGCCCAGGTCTA

>p1_ind1657

TCACTTTAGTTATCCAGATCGGTCATCTGCGGTAAGACTAGAGAAACCGTTCTACTCTATATCCTCATACACAATCG
CCTAGCTGCAGCCCAGGTCTG

>p1_ind3647

TCATTTTCGTTATCCAGATCGGTCATCTGTGGTAAGACTAGAGGAACCATTCTACTCTATATCCTCATACACAATCG
CCTAGCTGCAGCCCAGGTCTA

>p1_ind23

TCACTTTCGTTATCCAGATCGGTCATCTGTGGTAAGACTAGAGAAACCGTTCTACTCTATATCCTCATACACAATCG
CCTAGCTGCAGCCCAGGTCTA

>p1_ind1588

TCACTTTCGTTATCCAGATCGGTCATCCGCGGTAAGACTAGAGAAACCGTTCTACTCTATATCCTCATACACAATCG
CCTAGCTGCAGCCCAGGTCTA

>p1_ind2743

TCACTTTCGTTATCCAGATCGGTCATCTGTGGTAAGACTAGAGAAACCGTTCTACTCTATATCCTCATACACAATCG
CCTAGCTGCAGCCCAGGTCTA

>p1_ind3491

TCACTTTCGTTATCCAGATCGGTCATCCGCGGTAAGACTAGAGAAACCGTTCTACTCTATATCCTCATACACAATCG
CCTAGCTGCAGCCCAGGTCTA

>p1_ind3037

TCACTTTCGTAATCCAGATCGGTCATCCGCGGTAAGACTAGAGAAACCGTTCTACTCTATATCCTCATACACAATCG
CCTAGCTGCAGCCCAGGTCTA

>p1_ind213

TCACTTTCGTTATCCAGATCGGTCATCTGTGGTAAGACTAGTGAAACCGTTCTACTCTATATCCTCATACACAATCGC
CTAGCTGCAGCCCAGGTCTA

>p1_ind2389

TCACTTTCGTTATCCAGATCGGTCATCTGTGGTAAGACTAGAGAAACCGTTCTACTCTATATCCTCATACACAATCG
CCTAGCTGCAGCCCAGGTCTA

>p1_ind3656

TCACTTTCGTTATCCAGATCGGTCATCTGTGGTAAGACTAGAGAAACCGTTCTACTCTATATCCTCATACACAATCG
CCTAGCTGCAGCCCAGGTCTA

>p1_ind3529

TCACTTTCGTTATCCAGATCGGTCATCTGTGGTAAGACTAGAGAAACCGTTCTACTCTATATCCTCATACACAATCG
CCTAGCTGCAGCCCAGGTCTA

>p1_ind4721

TCACTTTCGTTATCCAGATCGGTCATCTGTGGTAAGACTAGAGAAACCGTTCTACTCTATATCCTCATACACAATCG
CCTAGCTGCAGCCCAGGTCTA

>p1_ind5048

TCACTTTCGTTATCCAGATCGGTCATCCGCGGTAAGACTAGAGAAACCGTTCTACTCTATATCCTCATACACAATCG
CCTAGCTGCAGCCCAGGTCTA

>p1_ind2484

TCACTTTCGTTATCCAGATCGGTCATCTGTGGTAAGACTAGAGAAACCGTTCTACTCTATATCCTCATACACAATCG
CCTAGCTGCAGCCCAGGTCTA

>p1_ind1764

TCACTTTCGTTATCCAGATCAGTCATCTGTGGTAAGACTAGAGAAACCGTTCTACTCTATATCCTCATACACAATCG
CCTAGCTGCAGCCCAGGTCTA

>p1_ind3636

TCACTTTCGTTATCCAGATCGGTCATCTGCGGTAAGACTAGAGGAACTGTTCTACTCTATATCCTCATACACAATCG
CCTAGCTGCAGCCCAGGTCTA

>p1_ind3950

TCACTTTCGTTATCCAGATCGGTCATCTGTGGCAAGACTTGAGAATCCGTTCTACTCTATATCCTCATACACAATCGC
CTAGCTGCAGCCCAGGTCTA

>p1_ind2635

TCACTTTCGTTATCCAGATCGGTCATCCGCGGTAAGACTAGAGAAACCGTTCTACTCTATATCCTCATACACAATCG
CCTAGCTGCAGCCCAGGTCTA

>p1_ind493

TCACTTTCGTTATCCAGATCGGTCATCCGCGGTAAGACTAGAGAAACCGTTCTACTCTATATCCTCATACACAATCG
CCTAGCTGCAGCCCAGGTCTA

>p1_ind3387

TCACTTTCGTTATCCAGATCGGTCATCTGTGGTAAGACTAGAGATACCGTTCTACTCTATATCCTCATACACAATCGC
CTAGCTGCAGCCCAGGTCTA

>p1_ind305

TCACTTTCGTTATCCAGATCGGTCATCCGCGGTAAGACTAGAGAAACCGTTCTACTCTATATCCTCATACACAATCG
CCTAGCTGCAGCCCAGGTCTA

>p1_ind3147

TCACTTTCGTTATCCAGATCGGTCATCTGTGGCAAGACTAGAGGAACTGTTCTACTCTATATCCTCATACATAATCG
CCTAGCTGCAGCCCAGGTCTA

>p1_ind2760

TCACTTTCGTTATCCAGATCGGTCATCCGCGGTAAGACTAGAGAAACCGTTCTACTCTATATCCTCATACACAATCG
CCTAGCTGCAGCCCAGGTCTA

>p1_ind3088

TCACTTTCGTTATCCAGATCGGTCATCCGCGGTAAGACTAGAGAAACCGTTCTACTCTATATCCTCATACACAATCG
CCTAGCTGCAGCCCAGGTCTA

>p1_ind2154

TCACTTTCGTTATCCAGATCGGTCATCTGCGGTAAGACTAGAGAAACCGTTCTACTCTATATCCTCATACACAATCG
CCTAGCTGCAGCCCAGGTCTA

>p1_ind1368

TCACTTTCGTTATCCAGATCGGTCATCCGCGGTAAGACTAGAGAAACCGTTCTACTCTATATCCTCATACACAATCG
CCTAGCTGCAGCCCAGGTCTA

>p1_ind666

TCACTTTCGTTATCCAGATCGGTCATCCGCGGTAAGACTAGAGCAACCGTTCTACTCTATATCCTCATACACAATCG
CCTAGCTGCAGCCCAGGTCTA

>p1_ind2128

TCACTTTCGTTATCCAGATTGGTCATCCGCGGTAAGACTAGAGAAACCGTTCTACTCTATATCCTCATACACAATCG
CCTAGCTGCAGCCCAGGTCTA

>p1_ind2813

TCACTTTCGTTATCCAGATCGGTCATCTGTGGTAAGACTAGAGACACCGTTCTACTCTATATACTCATACACAATCG
CCTAGCTGCCGGCCAGGTCTA

>p1_ind2511

TCACTTTCGTTATCCAGATCGGTCATCCGCGGTAAGACTAGAGAAACCGTTCTACTCTATATCCTCATACACAATCG
CCTAGCTGCAGCCCAGGTCTA

>p1_ind512

TCACTTTCGTTATCCAGATCGGTCATCTACGGTAAGACTAGAGAAACCGTTCTACTCTATATCCTCATACACAATCG
CCTAGCTGCAGCCCAGGTCTA

>p1_ind4471

TCACTTTCGTTATCCAGATCGGTCATCCGCGGTAAGACTAGAGAAACCGTTCTACTCAATATCCTCATACACAATCG
CCTAGCTGCAGCCCAGGTCTA

>p1_ind4138

TCACTTTCGTTATCCAGATCGGTCATCTGTGGTAAGATTAGAGTAACCGTTCTACTCTATATCCTCATACACAATCGC
CTAGCTGCAGCCCAGGTCTA

>p1_ind4715

TCACTTTCGTTATCCAGATCGGTCATCCGCGGTAAGACTAGAGAAACCGTTCTACTCTATATCCTCATACACAATCG
CCTCGCTGCAGCCCAGGTCTA

>p1_ind2385

TCACTTTCGTTATCCAGATCGGTCATCCGCGGTAAGACTAGAGAAACCGTTCTACTCTATATCCTCATACACAATCG
CCTCGCTGCAGCCCAGGTCTA

>p1_ind2512

TCACTTTCGTTATCCAGATCGGTCATCTGTGGTAAGACTAGAGAAACCGTTCTACTCTATATCCTCATACACAATCG
CCTAGCTGCAGCCCAGGTCTA

>p1_ind3588

TCACTTTCGTTATCCATATCGGTCATCTGCGGTAAGACTAGAGAAACCGTTCTACTATATATCCTCATACACAATCG
CCTAGCTGCAGCCCAGGTCTA

>p1_ind4597

TCACTTTCGTTATCCAGATCGGTCATCCGCGGTAAGACTAGAGAAACCGTTCTACTCTATATCCTCATACACAATCG
CCTAGCTGCAGCCCAGGTCTA

>p1_ind4495

TCACTTTCGTTATCCAGATCGGTCATCTGTGGTAAGACTAGAGAAACCGTTCTACTCTATATCCTCATACACAATCG
CCTAGCTGCAGCCCAGGTCTA

>p1_ind3219

TCACTTTCGTTATCCAGATCGGTCATCCGCGGTAAGACTAGAGAAACCGTTCTACTCTATATCCTCATACACAATCG
CCTAGCTGCAGCCCAGGTCTA

>p1_ind2463

TCATTTTCGTTATCCAGATCGGTCATCTGTGGTAAGACTAGAGGAACCGTTCGACTCTATATCCTCATACACAATCG
CCTAGCTGCAGCCCAGGTCTA

>p1_ind2647

TCACTTTCGTTATCCAGATCGGTCATCTGCGGTAAGACTAGAGAAACCGTTCTACTCTATATCCTCATACACAATCG
CCTAGCTGCAGCCCAGGTCTA

>p1_ind2298

TCACTTTCGTAATCCAGATCGGTCATCCGCGGTAAGACTAGAGAATCCGTTCTACTCTATATCCTCATACACAATCG
CCTAGCTGCAGCCCAGGTCTA

>p1_ind3024

TCACTTTCGTAATCCAGATCGGTCTTCCGCGGTAAGACTAGAGAAACCGTTCTACTCTATATCCTCATACACAATCG
CCTAGCTGCAGCCCAGGTCTA

>p1_ind2201

TCACTTTCGTTATCCAGATCGGTCATCTGTGGTAAGACTAGAGAAACCGTTCTACTCTATATCCTCATACACAATCG
CCTAGCTGCAGCCCAGGTCTA

>p1_ind4410

TCACTTTCGTTATCCAGATCGGTCATCCGCGGTAAGACTAGAGAAACCGTTCTACTCTATATCCTCATACACAATCG
CCTAGCTGCAGCCCAGGTCTA

>p1_ind2932

TCATTTTCGTTATCCAGATCGGTAATCTGCGGTAAGACTAGAGAAACCGTTCTACTCTATATCCTCATACACAATCG
CCTAGCTGCAGCCCAGGTCTA

>p1_ind894

TCACTTTCGTTATCCAGATCGGTCATCTGCGGTAAGACTAGAGAAACCGTTCTACTCTATATCCTCATACACAATCG
CCTAGCTGCAGCCCAGGTCTA

>p1_ind1377

TCACTTTCGTTATCCAGATCGGTCATCCGCCGTAAGACTAGAGAAACCGTTCTACTCTATATCCTCATACACAATCG
CCTAGCTGCAGCCCAGGTCTA

>p1_ind2662

TCACTTTCGTTATCCAGATCGGTCATCCGCGGTAAGACTAGAGAAACCGTTCTACTCTATATCCTCATACACAATCG
CCTAGCTGCAGCCCAGGTCTA

>p1_ind1642

TCACTTTCGTTATCCAGATCGGTCATCTGTGGTAAGACTAGAGAAACCGTTCTACTCTATATCCTCATACACAATCG
CCTAGCTGCAGCCTAGGTCTA

>p1_ind3662

TCACTTTCGTTATCCAGATCGGTCATCCGCGGTAAGACTAGAGAAACCGTTCTACTCTATATCCTCATACACAATCG
CCTAGCTGCAGCCCAGGTCTA

>p1_ind204

TCACTTTCGTTATCCAGATCGGTCATCTGCGGTAAGACTAGAGGAACTGTTCTACTCTATATCCTCATACACAATCG
CCTAGCTGCAGCCCAGGTCTA

>p1_ind530

TCACTTTCGTTATCCAGATCGGTCATCCGCGGTAAGACTAGAGAAACCGTTCTACTCAATATCCTCATACACAATCG
CCTAGCTGCAGCCCAGGTCTA

>p1_ind4642

TCACTTTCGTTATCCAGATCGGTCATCCGCGGTAAGACTAGAGAAACCGTTCTACTCTATATCCTCATACACAATCG
CCTAGCTGCAGCCCAGGTCTA

>p1_ind1810

TCACTTTCGTTATCCAGATCGGTCATCCGCGGTAAGACTAGAGAAACCGTTCTACTCTATATCCTCATACACAATCG
CCTAGCTGCAGCCCAGGTCTA

>p1_ind2902

TCACTTTCGTTATCCAGATCGGTCATCCGCGGTAAGACTAGAGCAACCGTTCTACTCTATATCCTCATACACAATCG
CCTAGCTGCAGCCCAGGTCTA

>p1_ind2744

TCACTTTCGTTATCCAGATCGGTCATGTGTGGTAAGACTAGAGGAACCATTCTACTCTATATCCTCATACACAATCG
CCTAGCTGCAGCCCAGGTCTA

>p1_ind1102

TCACTTTCGTTATCCAGATCGGTCATCTACGGTAAGACTAGAGAAACCGTTCTACTCTATATCCTCATACACAATCG
CCTAGCTGCAGCCCAGGTCTA

>p1_ind3376

TCACTTTCGTTATCCAGATCGGTCATCTGTGGTAAGACTAGAGAAACCGTTCTACTCTATATCCTCATACACAATCG
CCTAGCTGCAGCCCAGGTCTA

>p1_ind2598

TCACTTTCGTTATCCAGATCGGTCATGTGTGGTAAGACTAGAGAAACCGTTCTACTCTATATCCTCATACACAATCG
CCTAGCTGCAGCCCAGGTCTA

>p1_ind3843

TCATTTTCGTTATCCAGATCGGTAATCTGCGGTAAGACTAGAGAAACCGTTCTACTCTATATCCTCATACACAATCG
CCTAGCTGCAGCCCAGGTCTA

>p1_ind36

TCACTTTCGTTATCCAGATCGGTCATCCGCGGTAAGACTAGAGAAACCGTTCTACTCTATATCCTCATACACAATCG
CCTAGCTGCAGCCCAGGTCTA

>p1_ind1487

TCACTTTCGTTATCCAGATCGGTCATCTGCGGTAAGACTAGAGAAACCGTTCTACTCTATATCCCCATACACAATCG
CCTAGCTGCAGCCCAGGTCTA

>p1_ind3408

TCACTTTCGTTATCCAGATCGGTCATCTGTGGTAAGACTAGAGAAACCGTTCTACTCTATATCCTCATACACAATCG
CCTAGCTGCAGCCCAGGTCTA

>p1_ind2255

TCACTTTCGTTATCCAGATCGGTCATCTGCGGTAAGACTAGAGAAACCGTTCTACTCTATATCCTCATACACAATCG
CCTAGCTGCAGCCCAGGTCTA

>p1_ind379

TCACTTTCGTTATCCAGATCGGTCATCTGCGGTAAGACTAGAGAAACCGTTCTACTCTATATCCTCATACACAATCG
CCTAGCTGCAGCCCAGGTCTA

>p1_ind1411

TCACTTTCGTTATCCAGATCGGTCATCCGCGGTAAGACTAGAGAAACCGTTCTACTCTAAATCCTCATACACAATCG
CCTAGCTGCAGCCCAGGTCTA

>p1_ind5007

TCATTTTCGTTATCCAGATCGGTCATCTGTGGTAAGACTAGAGGAACCATTCTACTCTATATCCTCATACACAATCG
CCTAGCTGCAGCCCAGGTCTA

>p1_ind2323

TCACTTTCGTTATCCAGATCGGTCATCTACGGTAAGACTAGAGAAACCGTTCTACTCTATATCCTCATACACAATCG
CCTAGCTGCAGCCCAGGTCTA

>p1_ind4189

TCACTTTCGTTATCCAGATCGGTCATCCGCGGTAAGACTAGAGCAACCGTTCTACTCTATATCCTCATACACAATCG
CCTAGCTGCAGCCCAGGTCTA

>p1_ind3335

TCACTTTCGTTATCCAGATCGGTCATCCGCGGTAAGACTAGAGAAACCGTTCTACTCTATATCCTCATACACAATCG
CCTAGCTGCAGCCCAGGTCTA

>p1_ind664

TCACTTTCGTTATCCAGATCGGTCATCTGTGGTAAGACTAGAGAAACCGTTCTACTCTATATCCTCATACACAATCG
CCTAGCTGCAGCCCAGGTCTA

>p1_ind1992

TCACTTTCGTTATCCAGATCGGTCATCCGCGGTAAGACTAGAGCAACCGTTCTACTCTATATCCTCATACACAATCG
CCTAGCTGCAGCCCAGGTCTA

>p1_ind1730

TCACTTTCGTTATCCAGATCGGTCATCCGTGGTAAGACTAGAGAAACCGTTCTACTCTATATCCTCATACACAATCG
CCTAGCTGCAGCCTAGGTCTA

>p1_ind1445

TCACTTTCGTAATCCAGATCGGTCATCCGCGGTAAGACTAGAGAAACCGTTCTACTCTATATCCTCATACACAATCG
CCTAGCTGCAGCCCAGGTCTA

>p1_ind1228

TCACTTTCGTTATCCAGATCGGTCATCCACGGTAAGACTAGAGAAACCGTTCTACTCTATATCCTCATACACAATCG
CCTAGCTGCAGCCCAGGTCTA

>p1_ind174

TCACTTTCGTTATCCAGATCGGTCATCTGTGGTAAGACTAGAGAAACCGTTCTACTCTATATCCTCATACACAATCG
CCTAGCTGCAGCCTAGGTCTA

>p1_ind4228

TCACTTTCGTTATCCAGATCGGTCATCTGCGGTAAGACTAGAGAAACCGTTCTACTCTATATCCTCATACACAATCG
CCTAGCTGCAGCCCAGGTCTA

>p1_ind3697

TCACTTTCGTTATCCAGATCGGTCATCCGCGGTAAGACTAGAGAAACCGTTCTACTCTATATCCTCATACACAATCG
CCTAGCTGCAGCCCAGGTCTA

>p1_ind3428

TCACTTTCGTTATCCAGATCGGTCATCCGCGGTAAGACTAGAGAAACCGTTCTACTCTATATCCTCATACACAATCG
CCGAGCTGCAGCCCAGGTCTA

>p1_ind3298

TCACTTTCGTTATCCAGATCGGTCATCCGCGGTAAGACTAGAGAAACCGTTCTACTCTATATCCTCATACACAATCG
CCTAGCTGCAGCCCAGGTCTA

>p1_ind2214

TCACTTTCGTTATCCAGATCGCTCATCTGTGGTAAGACTAGAGAAACCGTTCTACTCTATATCCTCATACACAATCGC
CTCGCTGCAGCCCAGGTCTA

>p1_ind4921

TCACTTTCGTAATCCAGATCGGTCATCCGCGGTAAGACTAGAGAAACCGTTCTACTCTATATCCTCATACACAATCG
CCTAGCTGCAGCCCAGGTCTA

>p1_ind4218

TCATTTTCGTTATCCAGATCGGTCATCTGTGGTAAGACTAGAGGAACCATCATACTCTATATCCTCATACACAATCG
CCTAGCTGCAGCCCAGGTCTA

>p1_ind3813

TCACTTTCGTTATCCAGATCGGTCATCTGTGGTAAGACTAGAGAAACCGTTCTACTCTATATCCTCATACACAATCG
CCTAGCTGCAGCCTAGGTCTA

>p1_ind947

TCACTTTCGTTATCCAGATCGGTCATCCGCGGTAAGACTAGAGAAACCGTTCTACTCTATATCCTCATACACAATCG
CCTAGCTGCAGCCCAGGTCTA

>p1_ind100

TCACTTTCGTTATCCAGATCGGTCATCTGCGGTAAGACTAGAGAAACCGTTCTACTCTATATCCTCATACACAATCG
CCTAGCTGCAGCCCAGGTCTA

>p1_ind1600

TCACTTTCGTTATCCAGATCGGTCATCTGTGGCAAGACTAGAGATACCGTTCTACTCTATATCCTCATACACAATCG
CCTAGCTGCAGCCCAGGTCTA

>p1_ind3316

TCACTTTCGTTATCCAGATCGGTCATCCGCGGTAAGACTAGAGAAACCGTTCTACTCTATATCCTCATACACAATCG
CCTAGCTGCAGCCCAGGTCTA

>p1_ind4068

TCACTTTCGTTATCCAGATCGGTCATCCGCGGTAAGACTAGAGAAACCGCTCTACTCTATATCCTCATACACAATCG
CCTAGCTGCAGCCCAGGTCTA

>p1_ind2810

ACACTTTCGTTATCCAGATCGGTCATCCGCGGTAAGACTAGAGAAACCGTTCTACTCTATATCCTCATACACAATCG
CCTAGCTGCAGCCCAGGTCTA

>p1_ind1223

TCACTTTCGTTATCCAGATCGGTCATCTGCGGTAAGACTAGAGAAACCGTTCTACTCTATATCCTCATACACAATCG
CCTAGCTGCAGCCCAGGTCTA

>p1_ind3151

TCACTTTCGTTATCCAGATCGGTCATCCGCGGTAAGACTAGAGAAACCGTTCTACTCTATATCGTCATACACAATCG
CCTAGCTGCAGCCCAGGTCTA

>p1_ind2321

TCACTTTCGTTATCCAGATCGGTCATCCGCGGTAAGACTAGAGAAACCGTTCTACTCTATATCCTCATACACAATCG
CCTAGCTGCAGCCCAGGTCTA

>p1_ind3835

TCACTTTCGTTATCCAGATCGGTCATCCGCGGTAAGACTAGAGAAACCGTTCTACTCTATATCCTCATACACAATCG
CCTAGCTGCAGCCCAGGTCTA

>p1_ind281

TCACTTTCGTTATCCAGATCGGTCATCCGCGGTAAGACTAGAGAAACCGTTCTACTCTATATCCTCATACACAATCG
GCTAGCTGCAGCCCAGGTCTA

>p1_ind2147

TCACTTTCGTTATCCAGATCGGTCATCTGTGGTAAGACTAGAGAAACCGTTCTACTCTATATCCTCATACACAATCG
CCTAGCTGCAGCCCAGGTCTA

>p1_ind5034

TCACTTTCGTTATCCAGATCGGTCATCTGCGGTAAGACTAGAGAAACCGTTCTACTCTATATCCTCATACACAATCG
CCTAGCTGCAGCCCAGGTCTA

>p1_ind3445

TCACTTTCGTTATCCAGATCGGTCATCTACGGTAAGACTAGAGAAACCGTTCTACTCTATATCCTCATACACAATCG
CCTAGCTGCAGCCCAGGTCTA

>p1_ind292

TCACTTTCGTAATCCAGATCGGTCATCCGCGGTAAGACTAGAGAAACCGTTCTACTCTATATCCTCATACACAATCG
CCTAGCTGCAGCCCAGGTCTA

>p1_ind182

TCACTTTAGTTATCCAGATCGGTCATCCGCGGTAAGACTAGAGAAACCGTTCTACTCTATATCCTCATACACAATCG
CCTAGCTGCAGCCCAGGTCTA

>p1_ind459

TCACTTTCGTTATCCAGATCGGTCATCCGCGGTAAGACTAGAGAAACCGCTCTACTCTATATCCTCATACACAATCG
CCTAGCTGCAGCCCAGGTCTA

>p1_ind417

TCACTTTCGTTATCCAGATCGGTCATCTGTGGTAAGACTAGAGAAACCGTTCTACTCTATATCCTCATACACAATCG
CCTAGCTGCAGCCCAGGTCTA

>p1_ind2847

TCACTTTCGTTATCCAGATCGGTCATCTGTGGTAAGACTAGAGAAACCGTTCTACTCTATATCCTCATACACAATCG
CCTAGCTGCAGCCCAGGTCTA

>p1_ind1033

TCACTTTCGTTATCCAGATCGGTCATCTGTGGTAAGACTAGAGAAACCGTTCTACTCTATATCCTCATACACAATCG
CCTAGCTGCAGCCCAGGTCTA

>p1_ind3919

TCACTTTCGTTATCCAGATCGGTCATCTGTGGTAAGACTAGAGAAACCGTTCTACTCTATATCCTCATACACAATCG
CCTAGCTGCAGCCCAGGTCTA

>p1_ind1864

TCACTTTCGTTATCCAGATCGGTCATCTGTGGTAAGACTAGAGAAACCGTTCTACTCTATATCCTCATACACAATCG
CCTAGCTGCAGCCCAGGTCTA

>p1_ind1849

TCATTTTCGTTATCCAGATCGGTCATCTGTGGTAAGACTAGAGGAACCATTCTACTCTATATCCTCATACACAATCG
CCTAGCTGCAGCCCAGGTCTA

>p1_ind3718

TCACTTTCGTTATCCCGATCGGTCATCTGTGGTAAGACTAGAGAAACCGTTCTACTCTATATCCTCATACACAATCG
CCTAGCTGCAGCCCAGGTCTA

>p1_ind492

TCACTTTCGTTATCCAGATCGGTCATCTGTGGTAAGACTAGAGAAACCGTTCTACTCTATATCCTCATACACAATCG
CCTAGCTGCAGCCCAGGTCTA

>p1_ind2523

TCACTTTCGTTATCCAGATCGGTCATCTGTGGTAAGACTAGAGAAACCGTTCTACTCTATATCCTCATACACAATCG
CCTAGCTGCAGCCTAGGTCTA

>p1_ind3131

TCACTTTCGTTATCCAGATCGGTCATCTGCGGTAAGACTAGAGAAACCGTTCTACTCTATATCCTCATACACAATCG
CCTAGCTGCAGCCCAGGTCTA

>p1_ind4117

TCACTTTCGTTATCCAGATCGGTCATGTGTGGTAAGACTAGAGAAACCGTTCTACTCTATATCCTCATACACAATCG
CCTAGCTGCAGCCCAGGTCTA

>p1_ind5028

TCACTTTCGTTATCCAGATCGGTCATCTACGGTAAGACTAGAGAAACCGTTCTACTCTATATCCTCATACACAATCG
CCTAGCTGCAGCCCAGGTCTA

>p1_ind223

TCACTTTCGTTATCCAGATCGGTCATCCGCGGTAAGACTAGAGAAACCGTTCTACTCTATATCCTCATACACAATCG
CCTAGCTGCAGCCCAGGTCTA

>p1_ind644

TCACTTTCGTTATCCAGATCGGTCATCCGCGGTAAGACTAGAGGAACCGTTCTACTCTATATCCTCATACACAATCG
CCTAGCTGCAGCCCAGGTCTA

>p1_ind97

TCACTTTCGTTATCCAGATCGGTCATCTGTGGCAAGACTAGAGAAACCGTTCTACTCTATATCCTCATACACAACCG
CCTAGCTGCAGCCCAGGTCTA

>p1_ind3743

TCATTTTCGTTATCCAGATCGGTAATCTGCGGTAAGACTAGAGAAACCGTTCTACTCTATATCCTCATACACAATCG
CCTAGCTGCAGCCCAGGTCTA

>p1_ind3168

TCACTTTCGTAATCCAGATCGGTCATCCGCGGTAAGACTAGAGAAACCGTTCTACTCTATATCCTCATACACAATCG
CCTAGCTGCAGCCCAGGTCTA

>p1_ind1273

TCATTTTCGTTATCCAGATCGGTCATCTGTGGTAAGACTAGAGGAACCGTTCTACTCTATATCCTCATACACAATCG
CCTAGCTGCAGCCCAGGTCTA

>p1_ind5136

TCACTTTCGTTATCCAGATCGGTCATCTACGGTAAGACTAGAGAAACCGTTCTACTCTATATCCTCATACACAATCG
CCTAGCTGCAGCCCAGGTCTA

>p1_ind3214

TCACTTTCGCAATCCAGATCGGTCATCTGTGGTAAGACTAAAGAAACCGTTCTACTCTATATCCTCATACACAATCG
CCTAGCTGCAGCCCAGGTCTA

>p1_ind4837

TCACTTTCGTTATCCAGATCGGTCATCCGCGGTAAGACTAGAGAAACCGTTCTACTCTATATCCTCATACACAATCG
CCTAGCTGCAGCCCAGGTCTA

>p1_ind3302

TCACTTTCGTTATCCAGATCGGTCATCTGTGGCAAGACTAGAGAAACCGTTCTACTCTATATCCTCATACACAATCG
CCTAGCTGCAGCCCAGGTCTA

>p1_ind1167

TCACTTTCGTTATCCAGATCGGTCATCCGCGGTAAGACTAGAGAAACCGTTCTACTCTATATCCTCATACACAATCG
CCTCGCTGCAGCCCAGGTCTA

>p1_ind3558

TCATTTTCGTTATCCAGATCGGTCATCTGTGGTAAGACTAGAGGAACCATTCTACTCTATATCCTCATACACAATCG
CCTAGCTGCAGCCCAGGTCTA

>p1_ind686

TCACTTTCGTTATCCAGATCGGTCATCCGCGGTAAGACTAGAGAAACCGTTCTACTCTATATCCTCATACACAATCG
CCTAGCTGCAGCCCAGGTCTA

>p1_ind3518

TCACTTTCGTTATCCAGATCGGTCATCTGCGGTAAGACTAGAGAAACCGTTCTACTCTATATCCTCATACACAATCG
CCTAGCTGCAGCCCAGGTCTA

>p1_ind401

TCACTTTCGTTATCCAGATCGGTCATCCGCGGTAAGACTAGAGAAACCGTTCTACTCTATATCCTCATACACAATCG
CCTAGCTGCAGCCCAGGTCTA

>p1_ind1278

TCACTTTCGTTATCCAGATCGGTCATCTGTGGTAAGACTAGAGAAACCGTTCTACTCTATATCCTCATACCCAATCG
CCTAGCTGCAGCCCAGGTCTA

>p1_ind3506

TCACTTTCGTTATCCAGATCGGTCATCCGCGGTAAGACTAGAGAAACCGTTCTACTCTATATCCTCATACACAATCG
CCTAGCTGCAGCCCAGGTCTA

>p1_ind4069

TCACTTTCGTTATCCAGATCGGTCATCCGCGGTAAGACTAGAGAAACCGTTCTACTCTATATCCTCATACACAATCG
CCTAGCTGCAGCCCAGGTCTA

>p1_ind4909

TCACTTTCGTTATCCAGATCGGTCATCCGCGGTAAGACTAGAGAAACCGCTCTACTCTATATCCTCATACACAATCG
CCTAGCTGCAGCCCAGGTCTA

>p1_ind3007

TCACTTTCGTTATCCAGATCGGTCATCCGCGGTAAGACTAGAGAAACCGTTCTACTCTATATCCTCATACACAATCG
CCTAGCTGCAGCCCAGGTCTA

>p1_ind1722

TCACTTTCGTTATCCAGATCGGTCATCTGTGGTAAGACTAGAGAAACCGTTCTACTCTATATCCTCATACACAATCG
CCTAGCTGCAGCCCAGGTCTA

>p1_ind4059

TCACTTTCGTTATCCAGATCGGTCATCCGCGGTAAGACTAGAGAAACCGTTCTACTCTATATCCTCATACACAATCG
CCTAGCTGCAGCCCAGGTCTA

>p1_ind4120

TCACTTTCGTTATCCAGATCGGTCATCTGTGGTAAGACTAGAGGAACCATTCTACTCTATATCCTCATACACAATCG
CCTAGCTGCAGCCCAGGTCTA

>p1_ind3565

TCACTTTCGTTATCCAGATCGGTCATCCGCGGTAAGACTAGAGAAACCGTTCTACTCTATATCCTCATACACAATCG
CCTAGCTGCAGCCCAGGTCTA

>p1_ind752

TCACTTTCGTTATCCAGATCGGTCATCTGTGGTAAGACTAGAGACACCGTTCTACTCTATATCCTCATACACAATCG
CCTAGCTGCAGGCCAGGTCTA

>p1_ind3834

TCACTTTCGTTATCCAGATCGGTCATCCGCGGTAAGACTAGAGGAACCGTTCTACTCTATATCCTCATACACAATCG
CCTAGCTGCAGCCCAGGTCTA

>p1_ind4076

TCACTTTCGTTATCCAGATCGGTCATCCGCGGTAAGACTAGAGAAACCGTTCTACTCTATATCCTCATACACAATCG
CCTAGCTGCAGCCCAGGTCTA

>p1_ind3156

TCACTTTCGTTATCCAGATCGGTCATCTGCGGTAAGACTAGAGAAACCGTTCTACTCTATATCCTCATACACAATCG
CCTAGCTGCAGCCCAGGTCTA

>p1_ind332

TCACTTTCGTTATCCAGATCGGTCATCTGTGGCAAGACTAGAGAAACCGTTCTACTCTATATCCTCATACACAACCG
CCTAGCTGCAGCCCAGGTCTA

>p1_ind4305

TCACTTTCGTAATCCAGATCGGTCATCCGCGGTAAGACTAGAGAAACCGTTCTACTCTATATCCTCATACACAATCG
CCTAGCTGCAGCCCAGGTCTA

>p1_ind3925

TCACTTTCGTTATCCAGATCGGTCATCTGTGGTAAGACTAGAGAAACCGTTCTACTCTATATCCTCATACACAATCG
CCTAGCTGCAGCCCAGGTCTA

>p1_ind2571

TCACTTTCGTTATCCAGATCGGTCATCTGTGGTAAGACTAGAGAAACCGTTCTACTCTATATCCTCATACACAATCG
CCTAGCTGCAGTCCAGGTCTA

>p1_ind1513

TCACTTTCGTTATCCAGATCGGTCATCTGTGGTAAGACTAGAGAAACCGTTCTACTCTATATCCTCATACACAATCG
CCTAGCTGCAGCCCAGGTCTA

>p1_ind769

TCACTTTCGTTATCCAGATCGGTCATCCGCGGTAAGACTAGAGAAACCGTTCTACTCTATATCCTCATACACAATCG
CCTAGCTGCAGCCCAGGTCTA

>p1_ind1909

TCACTTTCGTTATCCAGATCGGTCATCCGCGGTAAGACTAGAGAAACCGTTCTACTCTATATCCTCATACACAATCG
CCTAGCTGCAGCCCAGGTCTA

>p1_ind1257

TCACTTTCGTTATCCAGATCGGTCATCCGCGGTAAGACTAGAGAAACCGTTCTACTCTAAATCCTCATACACAATCG
CCTAGCTGCAGCCCAGGTCTA

>p1_ind1972

TCACTTTCGTTATCCAGATCGGTCATCTGTGGTAAGACTAGAGAAACCGTTCTACTCTATATCCTCATACACAATCG
CCTAGCTGCAGTCCAGGTCTA

>p1_ind1298

TCACTTTCGTTATCCAGATCGGTCATCCGCGGTAAGACTAGAGAAACCGTTCTACTCTATATCCTCATACACAATCG
CCTAGCTGCAGCCCAGGTCTA

>p1_ind44

TCACTTTCGTTATCCAGATCGGTCATCTGTGGTAAGACTAGAGGAACTGTTCTACTCTATATCCTCATACACAATCG
CCTAGCTGCAGCCCAGGTCTA

>p1_ind1373

TCACTTTCGTAATCCAGATCGGTCATCCGCGGTAAGACTAGAGAAACCGTTCTACTCTATATCCTCATACACAATCG
CCTAGCTGCAGCCCAGGTCTA

>p1_ind2363

TCACTTTCGTAATCCAGATCGGTCATCCGCGGTAAGACTAGAGAAACCGTTCTACTCTATATCCTCATACACAATCG
CCTAGCTGCAGCCCAGGTCTA

>p1_ind134

TCACTTTCGTTATCCAGATCGGTCATCCGCGGTAAGACTAGAGAAACCGTTCTACTCTAAATCCTCATACACAATCG
CCTAGCTGCAGCCCAGGTCTA

>p1_ind2890

TCACTTTCGTTATCCAGATCGGTCATCTGTGGTAAGACTAGAGAAACCGTTCTACTCTATATCCTCATACACAATCG
CCTAGCTGCAGCCCAGGTCTA

>p1_ind635

TCACTTTCGTTATCCAGATCGGTCATCTGTGGCAAGACTAGAGAAACCGTTCTACTCTATATCCTCATACACAACCG
CCTAGCTGCAGCCCAGGTCTA

>p1_ind2751

TCATTTTCGTTATCCAGATCGGTCATCTGTGGTAAGACTAGAGGAACCATCATACTCTATAACCTCATACACAATCG
CCTAGCTGCAGCCCAGGTCTA

>p1_ind403

TAATTTTCGTTATCCAGATCGGTCATCTGTGGTAAGATTAGAGTAACCGTTCTACTCTATATCCTCATACACAATCGC
CTAGCTGCAGCCCAGGTCTA

>p1_ind3604

TCACTTTCGTTATCCAGATCGGTCATCTGTGGTAAGACTAGAGAAACCGTTCTACTCTATATCCTCATACACAATCG
CCTAGCTGCAGCCCAGGTCTA

>p1_ind4740

TCACTTTCGTTATCCAGATCGGTCATCTGTGGTAAGACTAGAGGAACCATTCTACTCTATATCCTCATACACAATCG
CCTAGCTGCAGCCCAGGTCTA

>p1_ind3401

TCACTTTCGTTATCCAGATCGGTCATCCGCGGTAAGACTAGAGAAACCGTTCTACTCTATATCCTCATACACAATCG
CCTAGCTGCAGCCCAGGTCTA

>p1_ind4082

TCACTTTCGTTATCCAGATCGGTCATCCGCGGTAAGACTAGAGAAACCGTTCTACTCTATATCCTCATACACAATCG
CCTAGCTGCAGCCCAGGTCTA

>p1_ind2304

TCACTTTCGTTATCCAGAACGGTCATCTGTGGTAAGACTAGAGAAACCGTTCTACTCTATATCCTCATACACAATCG
CCTAGCTGCAGCCCAGGTCTA

>p1_ind2904

TCACTTTCGTTATCCAGATCGGTCATCTGTGGTAAGACTAGAGAAACCGTTCTACTCTATATCCTCATACACAATCG
CCTAGCTGCAGCCCAGGTCTA

>p1_ind2877

TCACTTTCGTTATCCAGATCGGTCATCTGCGGTAAGACTAGAGAAACCGTTCTACTCTATATCCTCATACACAATCG
CCTAGCTGCAGCCCAGGTCTA

>p1_ind504

TCATTTTCGTTATCCAGATCGGTAATCTGCGGTAAGACTAGAGAAACCGTTCTACTCTATATCCTCATACACAATCG
CCTAGCTGCAGCCCAGGTCTA

>p1_ind1193

TCACTTTAGTTATCCAGATCGGTCATCTGCGGTAAGACTAGAGAAACCGTTCTACTCTATATCCTCATACACAATCG
CCTAGCTGCAGCCCAGGTCTG

>p1_ind3164

TCACTTTCGTAATCCAGATCGGTCATCCGCGGTAAGACTAGAGAAACCGTTCTACTCTATATCCTCATACACAATCG
CCTAGCTGCAGCCCAGGTCTA

>p1_ind4344

TCACTTTCGTTATCCAGATCGGTCATCCGCGGTAAGACTAGAGCAACCGTTCTACTCTATATCCTCATACACAATCG
CCTAGCTGCAGCCCAGGTCTA

>p1_ind314

TCATTTTCGTTATCCAGATCGGTAATCTGCGGTAAGACTAGAGAAACCGTTCTACTCTATATCCTCATACACAATCG
CCTAGCTGCAGCCCAGGTCTA

>p1_ind1615

TCACTTTCGTTATCCAGATCGGTCATCTGCGGTAAGACTAGAGAAACCGTTCTACTCTATATCCTCATACACAATCG
CCTAGCTGCAGCCCAGGTCTA

>p1_ind2402

ACACTTTCGTTATCCAGATCGGTCATCCGCGGTAAGCCTAGAGAAACCGTTCTACTCTATATCCTCATACACAATCG
CCTAGCTGCAGCCCAGGTCTA

>p1_ind1606

TCACTTTCGTTATCCAGATCGGTCATCCGCGGTAAGACTAGAGAAACCGTTCTACTCTATATCCTCATACACAATCG
CCTCGCTGCAGCCCAGGTCTA

>p1_ind2661

TCACTTTCGTAATCCAGATCGGTCATCCGCGGTAAGACTAGAGAAACCGTTCTACTCTATATCCTCATACACAATCG
CCTAGCTGCAGCCCAGGTCTA

>p1_ind391

TCACTTTCGTTATCCAGATCGGTCATGTGTGGTAAGACTAGAGGAACCATTCTACTCTATATCCTCATACACAATCG
CCTAGCTGCAGCCCAGGTCTA

>p1_ind2279

TCACTTTCGTTATCCAGATCGGTCATCCGCGGTAAGACTAGAGAAACCGTTCTACTCTATATCCTCACACACAATCG
CCTAGCTGCAGCCCAGGTCTA

>p1_ind2102

TCACTTTCGTTATCCAGATCGGTCATCTGCGGTAAGACTAGAGAAACCGTTCTACTCTATATCCTCATACACAATCG
CCTAGCTGCAGCCCAGGTCTA

>p1_ind5080

ACACTTTCGTTATCCAGATCGGTCATCCGCGGTAAGCCTAGAGAAACCGTTCTACTCTATATCCTCATACACAATCG
CCTAGCTGCAGCCCAGGTCTA

>p1_ind244

TCACTTTCGTTATCCAGATCGGTCATCCGCGGTAAGACTAGAGATACCGTTCTACTCTATATCCTCATACACAATCG
CCTAGCTGCAGCCCAGGTCTA

>p1_ind3646

TCACTTTCGTTATCCAGATCGGTCATCTGTGGTAACACTAGAGAAACCGTTCTACTGTATATCCTCATACACAATCG
CCTAGCTGCAGCCCAGGTCTA

>p1_ind4019

TCACTTTCGTAATCCAGATCGGTCATCCGCGGTAAGACTAGAGAAACCGTTCTACTCTATATCCTCATACACAATCG
CCTAGCTGCAGCCCAGGTCTA

>p1_ind665

TCACTTTCGTTATCCAGATCGGTCATCCGCGGTAAGACTAGAGAAACCGTTCTACTCTATATCCTCATACACAATCG
CCTAGCTGCAGCCCAGGTCTA

>p1_ind41

TCACTATCGTTATCCAGATCGGTCATCCGCGGTAAGACTAGAGAAACCGTTCTACTCTATATCCTCATACACAATCG
CCTAGCTGCAGCCCAGGTCTA

>p1_ind3098

TCACTTTCGTTATCCAGATCGGTCATCCGCGGTAAGACTAGAGAAACCGTTCTACTCTATATCCTCATACACAATCG
CCTAGCTGCAGCCCAGGTCTA

>p1_ind3788

TCACTTTCGTTATCCAGATCGGTCATCCGCGGTAAGACTAGAGAAACCGTTCTACTCTATATCCTCATACACAATCG
CCTAGCTGCAGCCCAGGTCTA

>p1_ind4098

ACACTTTCGTTATCCAGATCGGTCATCCGCGGTAAGACTAGAGAAACCGTTCTACTCTATATCCTCATACACAATCG
CCTAGCTGCAGCCCAGGTCTA

>p1_ind4103

TCACTTTCGTTATCCAGATCGGTCATCTGTGGTAAGACTAGAGAAACCGTTCTACTCTATATCCTCATACACAATCG
CCTAGCTGCAGCCCAGGTCTA

>p1_ind3089

TCACTTTCGTTATCCAGATCGGTCATCTGTGGTAAGATTAGAGAAACCGTTCTACTCTATATCCTCATACACAATCG
CCTAGCTGCAGGCCAGGTCTA

>p1_ind3260

TCACTTTCGTTATCCAGATCGGTCATCCGCGGTAAGACTAGAGAAACCGTTCTACTCTATATCCTCATACACAATCG
CCTAGCTGCAGCCCAGGTCTA

>p1_ind69

TCACTTTCGTTATCCAGATCGGTCATCTGTGGTAAGACTAGAGGAACCATTCTACTCTATATCCTCATACACAATCG
CCTAGCTGCAGCCCAGGTCTA

>p1_ind4485

TCACTTTCGTTATCCAGATCGGTCATCTACGGTAAGACTAGAGAAACCGTTCTACTCTATATCCTCATACACAATCG
CCTAGCTGCAGCCCAGGTCTA

>p1_ind79

TCACTTTCGTTATCCAGATCGGTCATCCGCGGTAAGACTAGAGAAACCGTTCTACTCTATATCCTCATACACAATCG
CCTCGCTGCAGCCCAGGTCTA

>p1_ind4116

TCACTTTCGTTATCCAGATCGGTCATCTGTGGCAAGACTAGAGATACCGTTCTACTCTATATCCTCATACACAATCG
CCTAGCTGCAGCCCAGGTCTA

>p1_ind934

TCACTTTCGTTATCCAGATCGGTCATCCGCGGTAAGACTAGAGAAACCGTTCTACTCTATATCCTCATACACAATCG
CCTAGCTGCAGCCCAGGTCTA

>p1_ind2235

TCACTTTCGTAATCCAGATCGGTCATCCGCGGTAAGACTAGAGAAACCGTTCTACTCTATATCCTCATACACAATCG
CCTAGCTGCAGCCCAGGTCTA

>p1_ind2073

TCACTTTCGTTATCCAGATCGGTCATCTGTGGTAAGACTAGAGAAACCGTTCTACTCTATATCCTCATACACAATCG
CCTAGCTGCAGCCCAGGTCTA

>p1_ind994

TCACTTTCGTTATCCAGATCGGTCATCTGTGGTAAGACTAGAGAAACCGTTCTACTCTATATCCTCATACACAATCG
CCTAGCTGCAGCCCAGGTCTA

>p1_ind4346

TCCCTTTCGTTATCCAGATCGGTCATCTACGGTAAGACTAGAGAAACCGTTCTACTCTATATCCTCATACACAATCG
CCTAGCTGCAGCCCAGGTCTA

>p1_ind404

TCACTTTCGTTATCCAGATCGGTCATCCGCGGTAAGACTAGAGAAACCGTTCTACTCTATATCCTCATACACAATCG
CCTAGCTGCAGCCCAGGTCTA

>p1_ind2560

TCACTTTCGTTATCCAGATCGGTCATCTACGGTAAGACTAGAGAAACCGTTCTACTCTATATCCTCATACACAATCG
CCTAGCTGCAGCCCAGGTCTA

>p1_ind4881

TCATTTTCGTTATCCAGATCGGTCATCTGTGGTAAGACTAGAGGAACCATCATACTCTATATCCTCATACACAATCG
CCTAGCTGCAGCCCAGGTCTA

>p1_ind939

TCACTTTCGTTATCCAGATCGGTCATCTGTGGTAACACTAGAGAAACCGTTCTACTGTATATCCTCATACACAATCG
CCTAGCTGCAGCCCAGGTCTA

>p1_ind4509

TCACTTTCGTAATCCAGATCGGTCATCCGCGGTAAGACTAGAGAATCCGTTCTACTCTATATCCTCATACACAATCG
CCTAGCTGCAGCCCAGGTCTA

>p1_ind4604

TCACTTTCGTTATCCAGATCGGTCATCTGTGGTAAGCCTAGAGGAACCATTCTACTCTATATCCTCATTCACAATCGC
CTAGCTGCAGCCCAGGTCTA

>p1_ind4031

TCACTTTCGTTATCCAGATCGGTCATCCGCGGTAAGACTAGAGAAACCGTTCTACTCTATATCCTCATACACAATCG
CCTAGCTGCAGCCCAGGTCTA

>p1_ind4992

TCCCTTTCGTTATCCAGATCGGTCATCTACGGTAAGACTAGAGAAACCGTTCTACTCTATATCCTCATACACAATCG
CCTAGCTGCAGCCCAGGTCTA

>p1_ind1632

TCACTTTCGTTATCCAGATCGGTCATCTGTGGTAAGACTAGAGAAACCGTTCTACTCTATATCCTCATACACAATCG
CCTAGCTGCAGCCCAGGTCTA

>p1_ind3021

TCACTTTCGTTATCCAGATCGGTCATCCGCGGTAAGACTAGAGAAACCGTTCTACTCTATATCCTCATACACAATCG
CCTAGCTGCAGCCCAGGTCTA

>p1_ind3237

TCACTTTCGTTATCCAGATCGGTAATCTGCGGTAAGACTAGAGAAACCGTTCTACTCTATATCCTCATACACAATCG
CCTAGCTGCAGCCCAGGTCTA

>p1_ind3852

TCACTTTCGTTATCCAGATCGGTCATCTGTGGTAAGACTAGAGAAACCGTTCTACTCTATATCCTCATACACAATCG
CCTAGCTGCAGCCCAGGTCTA

>p1_ind4957

TCACTTTCGTTATCCAGATCGGTCATCTGTGGTAAGACTAGAGAAACCGTTCTACTCTATATCCTCGTACACAATCG
CCTAGCTGCAGCCTAGGTCTA

>p1_ind3616

TCACTTTCGTTATCCAGATCGGTCATCTGCGGTAAGACTAGAGAAACCGTTCTACTCTATATCCTCATACACAATCG
CCTAGCTGCAGCCCAGGTCTA

>p1_ind2520

TCACTTTCGTTATCCAGATCGGTCATCTGTGGTAAGACTAGAGGAACCATTCTACTCTATATCCTCATACACATTCGC
CTAGCTGCAGCCCAGGTCTA

>p1_ind5074

TCACTTTCGTTATCCAGATCGGTCATCCGCGGTAAGACTAGAGAAACCGTTCTACTCTATATCCTCATACACAATCG
CCTAGCTGCAGCCCAGGTCTA

>p1_ind660

TCACTTTCGTAATCCAGATCGGTCATCCGCGGTAAGACTAGAGAAACCGTTCTACTCTATATCCTCATACACAATCG
CCTAGCTGCAGCCCAGGGCTA

>p1_ind3769

TCACTTTCGTTATCCAGATCGGTCATCTGTGGCAAGACTAGAGAAACCGTTCTACTCTATATCCTCATACACAATCG
CCTAGCTGCAGGCCAGGTCTA

>p1_ind3571

TCACTTTCGTTATCCAGATCGGTCATCTGTGGCAAGACTAGAGAAACCGTTCTACTCTATATCCTCATACACAACCG
CCTAGCTGCAGCCCAGGTCTA

>p1_ind2837

TCACTTTCGTTATCCAGATCGGTAATCTGCGGTAAGACTAGAGAAACCGTTCTACTCTATATCCTCATACACAATCG
CCTAGCTGCAGCCCAGGTCTA

>p1_ind1653

TCACTTTCGTTATCCAGATCGGTCATCCGCGGTAAGACTAGAGATACCGTTCTACTCTGTATCCTCATACACAATCG
CCTAGCTGCAGCCCAGGTCTA

>p1_ind3674

TCACTTTCGTTATCCAGATCGGTCATCTGCGGTAAGACTAGAGAAACCGTTCTACTCTATATCCTCATACACAATCG
CCTAGCTGCAGCCCAGGTCTA

>p1_ind1846

TCACTTTCGTTATCCAGATCGGTCATCCGCGGTAAGACTAGAGAAACCGTTCTACTCTATATCCTCATACACAATCG
CCTAGCTGCAGCCCAGGTCTA

>p1_ind786

TCACTTTCGTTATCCAGATCGGTCATCTGTGGTAAGACTAGAGAAACCGTTCTACTCTATATCCTCATACACAATCG
CCTCGCTGCAGCCCAGGTCTA

>p1_ind2524

TCACTTTCGTAATCCAGATCGGTCATCCGCGGTAAGACTAGAGAAACCGTTCTACTCTATATCCTCATACACAATCG
CCTAGCTGCAGCCCAGGTCTA

>p1_ind3759

TCACTTTCGTTATCCAGATCGGTCATGTGTGGTAAGACTAGAGGAACCATTCTACTCTATATCCTCATACACAATCG
CCTAGCTGCAGCCCAGGTCTA

>p1_ind4575

TCACTTTCGTTATCCAGATCGGTCATCTGTGGTAAGACTAGAGAAACCGTTCTACTCTATATCCTCATACACAATCG
CCTAGCTGCAGTCCAGGTCTA

>p1_ind4683

TCACTTTCGTTATCCAGATCGGTCATCCGCGGTAAGACTAGAGAAACCGTTCTACTCTATATCCTCATACACAATCG
CCTCGCTGCAGCCCAGGTCTA

>p1_ind4386

TCATTTTCGTTATCCAGATCGGTCATCTGTGGTAAGACTAGAGGAACCATTCTACTCTATATCCTCATACACAATCG
CCTAGCTGCAGCCCAGGTCTA

>p1_ind2690

TCACTTTCGTTATCCAGATCGGTCATCCGCGGTAAGACTAGAGAAACCGTTCTACTCTATATCCTCATACACAATCG
CCTAGCTGCAGCCCAGGTCTA

>p1_ind4712

ACACTTTCGTTATCCAGATCGGTCATCCGCGGTAAGCCTAGAGAAACCGTTCTACTCTATATCCTCATACACAATCG
CCTAGCTGCAGCCCAGGTCTA

>p1_ind4186

TCACTTTCGTTATCCAGATCGGTCATCTGTGGTAAGACTAGAGAAACCGTTCTACTCTATATCCTCATACACAATCG
CCTAGCTGCAGCCCAGGTCTA

>p1_ind4811

TCACTTTCGTTATCCAGATCGCTCATCTGTGGTAAGACTAGAGAAACCGTTCTACTCTATATCCTCATACACAATCGC
CTCGCTGCAGCCCAGGTCTA

>p1_ind3263

ACACTTTCGTTATCCAGATCGGTCATCCGCGGTAAGCCTAGAGAAACCGTTCTACTCTATATCCTCATACACAATCG
CCTAGCTGCAGCCCAGGTCTA

>p1_ind3728

TCACTTTCGTTATCCAGATCGGTCATCTGTGGTAAGACTAGAGAAACCGTTCTACACTATATCCTCATACACAATCG
CCTAGCTGCAGCCCAGGTCTA

>p1_ind229

TCACTTTCGTTATCCAGATCGGTCATCTGTGGTAAGACTAGAGAAACCGTTCTACTCTATATCCTCATACACAATCG
CCTAGCTGCAGCCCAGGTCTA

>p1_ind4926

TCACTTTCGTTATCCAGATCGGTCATCCGCGGTAAGACTAGAGCAACCGTTCTACTCTATATCCTCATACACAATCG
CCTAGCTGCAGCCCAGGTCTA

>p1_ind4537

TCACTTTCGTTATCCAGATCGGTCATCCGCGGTAAGACTAGAGAAACCGTTCTACTCTATATCCTCATACACAATCG
CCTAGCTGCAGCCCAGGTCTA

>p1_ind2586

TCACTTTCGTTATCCAGATCGGTCATCCGCGGTAAGACTAGAGAAACCGTTCTACTCTATATCCTCATACACAATCG
CCTAGCTGCAGCCCAGGTCTA

>p1_ind3624

TCACTTTCGTTATCCAGATCGGTCATCCGCGGTAAGACTAGAGAAACCGTTCTACTCTATATCCTCATACACAATCG
CCTAGCTGCAGCCCAGGTCTA

>p1_ind5066

TCACTTTCGTTATCCAGATCGGTCATCTGTGGTAAGACTAGAGAAACCGTTCTACTCTATATCCTCATACACAATCG
CCTAGCTGCAGCCCAGGTCTA

>p1_ind2548

TCACTTTCGTTATCCAGATCGGTCATCTGCGGTAAGACTAGAGAAACCGTTCTACTCTATATCCTCATACACAATCG
CCTAGCTGCAGCCCAGGTCTA

>p1_ind850

TCACTTTCGTTATCCAGATCGGTCATCCGCGGTAAGACTAGAGAAACCGTTCTACTCTATATCCTCATACACAATCG
CCTCGCTGCAGCCCAGGTCTA

>p1_ind1075

TCACTTTCGTTATCCAGATCGGTCATCCGCGGTAAGACTAGAGGAACCATTCTACTCTATATCCTCATACACAATCG
CCTAGCTGCAGCCAAGGTCTA

>p1_ind4251

TCACTTTCGTTATCCAGATCGGTCATCTGTGGTAAGACTAGAGAAACCGTTCTACTCTATATCCTCATACACAATCG
CCTAGCTGCAGCCCAGGTCTA

>p1_ind2008

TCACTTTCGTTATCCAGATCGGTCATCTGCGGTAAGACTAGAGAAACCGTTCTACTCTATATCCTCATACACAATCG
CCTAGCTGCAGCCCAGGTCTA

>p1_ind498

TCACTTTCGTTATCCAGATCGGTCATCCGCGGTAAGACTAGAGAAACCGTTCTACTCTATATCCTCATACACAATCG
CCTAGCTGCAGCCCAGGTCTA

>p1_ind4086

TCACTTTCGTTATCCAGATCGGTCATCTGTGGTAAGACTAGAGAAACCGTTCTACTCTATATCCTCATACACAATCG
CCTAGCTGCAGCCCAGGTCTA

>p1_ind426

TCACTTTCGTTATCCAGATCGGTCATCTGTGGTAAGACTAGAGAAACCGTTCTACTCTATATCCTCATACACAATCG
CCTAGCTGCAGCCCAGGTCTA

>p1_ind1559

TCACTTTCGTTATCCAGATCGGTCATCTGTGGTAAGACTAGAGAAACCGTTCTACTCTATATCCTCATACCCAATCG
CCTAGCTGCAGCCCAGGTCTA

>p1_ind746

TCACTTTCGTTATCCAGATCGGTCATCTGTGGTAAGACTAGAGAAACCGTTCTACTCTATATCCTCATACACAATCG
CCTAGCTGCAGCCCAGGTCTA

>p1_ind3815

TCACTTTCGTTATCCAGATCGGTCATCCGCGGTAAGACTAGAGAAACCGTTCTACTCTATATCCTCATACACAATCG
CCTAGCTGCAGCCCAGGTCTA

>p1_ind354

TCACTTTCGTTATCCAGATCGGTCATCTGTGGTAAGACTAGAGACACCGTTCTACTCTATATCCTCATACACAATCG
CCTAGCTGCAGGCCAGGTCTA

>p1_ind2547

TCACTTTCGTTATCCAGATCGGTCATCCGCGGTAAGACTAGAGAAACCGCTCTACTCTATATCCTCATACACAATCG
CCTAGCTGCAGCCCAGGTCTA

>p1_ind2569

TCACTTTCGTTATCCAGATCGGTCATCCGCGGTAAGACTAGAGAAACCGTTCTACTCTATATCCTCATACACAATCG
CCTAGCTGCAGCCCAGGTCTA

>p1_ind601

TCACTTTCGTTATCCAGATCGGTCATCCGCGGTAAGACTAGAGAAACCGTTCTACTCTATATCCTCATACACAATCG
GCTAGCTGCAGCCCAGGTCTA

>p1_ind1200

ACACTTTCGTTATCCAGATCGGTCATCCGCGGTAAGACTAGAGAAACCGTTCTACTCTATATCCTCATACACAATCG
CCTAGCTGCAGCCCAGGTCTA

>p1_ind3770

TCACTTTCGTAATCCAGATCGGTCATCCGCGGTAAGACTAGAGAAACCGTTCTACTCTATATCCTCATACACAATCG
CCTAGCTGCAGCCCAGGTCTA

>p2_ind242

TCACTTTCGTTATCCAGATCGGTCATCTGTGGTAAGACTAGAGAAACCGTTCTACTCTATATCCTCATACACAATCG
CCTAGCTGCAGCCCAGGTCTA

>p2_ind2560

TCACTATCGTTATCCAGATCGGTCATCTGTGGTAAGACTAGAGAAACCGTTCTACTCTATATCCTCATACACAATCG
CCTAGCTGCAGCCCAGGTCTA

>p2_ind4457

TCACTTTCGTTATCCAGATCGGTCATCTGTGGTAAGACTTGAGAATCCGTTCTACTCTATATCCTCATACACAATCGC
CTAGCTGCAGCCCAGGTCTA

>p2_ind821

TCACTTTCGTTATCCAGATCGGTCATCTGTGGTAAGACTAGAGAAACCGTTCTACTCTATATCCTCATACACAATCG
CCTAGCTGCAGCCCAGGTCTA

>p2_ind1682

TCACTTTCGTTATCCAGATCGGTCATCTGTGGTAAGACTAGAGAAACCGTTCTACTCTATATCCTCATACACAATCG
CCTAGCTGCAGCCCAGGTCTA

>p2_ind2601

TCACTTTCGTTATCCAGATCGGTAATCTGCGGTAAGACTAGAGAAACCGTTCTACTCTATATCCTCATACACAATCG
CCTAGCTGCAGCCCAGGTCTA

>p2_ind2922

TCACTTTCGTTATCCAGATCGGTCATCCGCGGTAAGACTAGAGAAACCGTTCTACTCTATATCCTCATACACAATCG
CCTAGCTGCAGCCTAGGTCTA

>p2_ind1893

TCACTTTCGTTATCCAGATCGGTCATCTGTGGTAAGACTAGAGGAACCATTCTACTCTATATCCTCATACACAATCG
CCTAGCTGCAGCCCAGGTCTA

>p2_ind1178

TCACTTTCGTTATCCAGATCGGTCATCCGCGGTAAGACTAGAGAAACCGTTCTACTCTATATCCTCATACACAATCG
CCTAGCTGCAGCCAAGGTCTA

>p2_ind4109

TCACTTTCGTTATCCAGATCGGTCATCTGTGGTAAGACTAGAGAAACCGTTCTACTCTATATCCTCATACACAATCG
CCTAGCTGCAGCCCAGGTCTA

>p2_ind1138

TCACTTTCGTTATCCAGATCGGTCATCTGTGGTAAGACTAGAGAAACCGCTCTACTCTATATCCTCATACACAATCG
CCTAGCTGCAGCCCAGGTCTA

>p2_ind3776

TCACTTTCGTTATCCAGATCGGTCATCTGTGGTAAGACTAGAGAAACCGTTCTACTCTATATCCTCATACACAATCG
CCTAGCTGCAGCCCAGGTCTA

>p2_ind1546

TCACTTTCGTTATCCAGATCGGTCATCTGTGGTAAGACTAGAGAAACCGTTCTACTCTATATCCTCATACACAATCG
CCTAGCTGCAGCCCAGGTCTA

>p2_ind721

TCACTTTCGTTATCCAGATCGGTCATCTGTGGTAAGACTAGAGAAACCGTTCTACTCTATATCCTCATACACAATCG
CCTAGCTGCAGCCCAGGTCTA

>p2_ind4499

TCACTTTCGTTATCCAGATCGGTCATCTGTGGTAAGACTAGAGAAACCGTTCTACTCTATATCCTCATACACAATCG
CCTAGCTGCAGCCCAGGTCTA

>p2_ind343

TCACTTTCGTTATCCAGATCGGTCATCTGTGGTAAGACTAGAGATACCGTTCTACTCTATATCCTCATACACAATCGC
CTAGCTGCAGCCCAGGTCTA

>p2_ind2597

TCACTTTCGTTATCCAGATCGGTCATCTGTGGTAAGACTAGAGATACCGTTCTACTCTATATCCTCATACACAATCGC
CTAGCTGCAGCCCAGGTCTA

>p2_ind123

TCACTTTCGTTATCCAGATCGGTCATCTGTGGTAAGACTAGAGGAACCGTTCTACTCTATATCCTCATACACAATCG
CCTAGCTGCAGCCCAGGTCTA

>p2_ind79

TCACTTTCGTTATCCAGATCGGTCATCTGTGGTAAGATTAGAGAAACCGTTCTACTCTATATCCTCATACACAATCG
CCTAGCTGCAGCCCAGGTCTA

>p2_ind183

TCATTTTCGTTATCCAGATCGGTCATCTGTGGTAAGACTAGAGGAACCATCATACTCTATAACCTCATACACAATCG
CCTAGCTGCAGCCCAGGTCTA

>p2_ind834

TCACTTTCGTTATCCAGATCGGTCATCTGTGGTAAGACTAGAGAAACCGTTCTACTCTATATCCTCATACACAATCG
CGTAGCTGCAGCCCAGGTCTA

>p2_ind4244

TCACTTTCGTTATCCAGATCGGTCATCTGTGGTAAGACTAGAGAAACCGTTCTACTCTATATCCTCATACACAATCG
CCTAGCTGCAGGCCAGGTCTA

>p2_ind4582

TCACTTTCGTTATCCAGATCGGTCATCTGCGGTAAGACTAGAGAAACCGTTCTACTCTATATCCTCATACACAATCG
CCTAGCTGCAGCCCAGGTCTA

>p2_ind2689

TCACTATCGTTATCCAGATCGGTCATCTGTGGTAAGATTAGAGAAACCGTTCTACTCTATATCCTCATACACAATCG
CCTAGCTGCAGCCCAGGTCTA

>p2_ind239

TCACTTTCGTTATCCAGATCGGTCATCCGCGGTAAGACTAGAGAAACCGTTCTACTCTATATCCTCATACACAATCG
CCTCGCTGCAGCCCAGGTCTA

>p2_ind822

TCACTTTCGTTATCCAGATCGGTCATCTGTGGTAAGACTAGAGAAACCGTTCTACTCTATATCCTCATACACAATCG
CCTAGCTGCAGCCCAGGTCTA

>p2_ind259

TCATTTTCGTTATCCAGATCGGTCATCTGTGGTAAGACTAGAGGAACCATTCTACTCTATATCCTCATACACAATCG
CCTAGCTGCAGCCCAGGTCTA

>p2_ind3355

TCACTTTCGTTATCCAGATCGGTCATCTGTGGTAAGACTAGAGAAACCGTTCTACTCTATATCCTCATACACAATCG
CCTAGCTGCACCCCAGGTCTA

>p2_ind4621

TCACTTTAGTTATCCAGATCGGTCATCTGCGGTAAGACTAGAGAAACCGTTCTACTCTATATCCTCATACACAATCG
CCTAGCTGCAGCCCAGGTCTG

>p2_ind3898

TCACTTTCGTTATCCAGATCGGTCATCTGTGGTAAGACTAGAGTAACCGTTCTACTCTATATCCTCATACACAATCGC
CTAGCTGCAGCCCAGGTCTA

>p2_ind3124

TCACTTTCGTTATCCAGATCGGTCATCCGCGGTAAGACTAGAGTAACCGTTCTACTCTATATCCTCATACACAATCG
CCTAGCTGCAGCCCAGGTCTA

>p2_ind2897

TCACTTTCGTTATCCAGATCGGTCATCCGCGGTAAGACTAGAGAAACCGTTCTACTCTATATCCTCATACACAATCG
CCTAGCTGCAGCCCAGGTCTA

>p2_ind428

TCACTTTCGTTATCCAGATCGGTCATCTGTGGTAAGACTAGAGAAACCGTTCTACTCTATATCCTCATACACAATCG
CCTAGCTGCAGCCCAGGTCTA

>p2_ind502

TCACTTTCGTTATCCAGATCGGTCAGCTGTGGTAAGACTAGAGAAACCGTTCTACTCTATATCCTCATACACAATCG
CCTAGCTGCAGCCCAGGTCTA

>p2_ind2620

TCACTTTCGTTATCCAGATCGGTCATCTGTGGTAAGACTAGAGAAACCGTTCTACTCTATATCCTCATACACAATCG
CCTAGCTGCAGCCCAGGTCTA

>p2_ind3209

TCACTTTCGTTATCCAGATCGGTAATCTGCGGTAAGACTAGAGGAACCGTTCTACTCTATATCCTCATACACAATCG
CCTAGCTGCAGCCCAGGTCTA

>p2_ind3563

TCACTTTCGTTATCCAGATCGGTCATCCGCGGTAAGACTAGAGAAACCGTTCTACTCTATATCCTCATACACAATCG
CCTAGCTGCAGCCAAGGTCTA

>p2_ind2492

TCACTTTCGTTATCCAGTTCGGTCATCTGTGGTAAGACTAGAGAAACCGTTCTACTCTATATCCTCATACACAATCGC
CTAGCTGCGGCCCAGGTCTA

>p2_ind1667

TCACTTTCGTTATCCAGATCGGTCATCTGTGGTAAGACTAGAGAAACCGTTCTACTCTATATCCTCATACACAATCG
CCTAGCTGCAGCCCAGGTCTA

>p2_ind4307

TCACTTTCGTTATCCAGATCGGTCATCTGTGGTAAGACTAGAGAAACCGTTCTACTCTATATCCTCATACACAATCG
CCTAGCTGCAGCCTAGGTCTA

>p2_ind2107

TCACTATCGTTATCCAGATCGGTCATCTGTGGTAAGACTAGAGAAACCGTTCTACTCTATATCCTCATACACAATCG
CCTAGCTGCAGCCCAGGTCTA

>p2_ind1043

TCACTTTCGTTATCCAGATCGGTCATCTGTGGTAAGACTAGAGAAACCGTTCTACTCTATATCCTCATACACAATCG
CCTAGCTGCAGCCCAGGTCTA

>p2_ind4895

TCACTTTCGTTATCCAGATCGGTCATCTGTGGTAAGACTAGAGATACCGTTCTACTCTATATCCTCATACACAATCGC
CTAGCTGCAGCCCAGGTCTA

>p2_ind2705

TCACTTTCGTTATCCAGATCGGTCATCTGTGGTAAGACTAGAGATACCGTTCTACTCTATATCCTCATACACAATCGC
CTAGCTGCAGCCCAGGTCTA

>p2_ind2132

TCACTTTCGTTATCCAGATCGGTCATCTGTGGTAAGACTAGAGACACCGTTCTACTCTATATCCTCATACACAATCG
CCTAGCTGCAGGCCAGGTCTA

>p2_ind1723

TCACTTTCGTTATCCAGATCGGTCATCCGCGGTAAGACTAGAGAAACCGTTCTACTCTATATCCTCATACACAATCG
CCTAGCTGCAGCCCAGGTCTA

>p2_ind3108

TCACTTTCGTTATCCAGATCGGTCATCTGCGGTAAGACTAGAGAAACTGTTCTACTCTATATCCTCATACACAATCG
CCTAGCTGCAGCCCAGGTCTA

>p2_ind335

TCACTTTCGTTATCCAGATCGGTCATCTGTGGTAAGACTAGAGAAACCGTTCTACTCTATATCCTCATACACAATCG
CCTAGCTGCAGCCTAGGTCTA

>p2_ind848

TCACTTTCGTTATCCAGATCGGTCATCTGTGGTAAGACTAGAGAAACCGTTCTACTCTATATCCTCATACACAATCG
CCTAGCTGCAGCCCAGGTCTA

>p2_ind2380

TCATTTTCGTTATCCAGATCGGTCATCTGTGGTAAGCCTAGAGGAACCATTCTACTCTATATCCTCATACACAATCGC
CTAGCTGCAGCCCAGGTCTA

>p2_ind4367

TCACTTTCGTTATCCAGATCGGTCATCTGTGGTAAGATTAGAGAAACCGTTCTACTCTATATCCTCATACACAATCG
CCTAGCTGCAGGCCAGGTCTA

>p2_ind4798

TCACTTTCGTTATCCAGATCGGTCATCTGTGGTAAGACTAGAGAAACCGTTCTACTCTATATCCTCATACACAATCG
CCTAGCTGCAGCCCAGGTCTA

>p2_ind1066

TCACTTTCGTTATCCAGATCGGTCATCTGTGGTAAGATTAGAGAAACCGTTCTACTCTATATCCTCATACACAATCG
CCTAGCTGCAGGCCAGGTCTA

>p2_ind2899

TCACTATCGTTATCCAGATCGGTCATCTGTGGTAAGATTAGAGAAACCGTTCTACTCTATATCCTCATACACAATCG
CCTAGCTGCAGCCCAGGTCTA

>p2_ind3417

TCACTTTCGTTATCCAGATCGGTCATCCGCGGTAAGACTAGAGAAACCGTTCTACTCTATATCCTCATACACAATCG
CCTAGCTGCAGCCCAGGTCTA

>p2_ind4025

TCACTTTCGTTATCCAGATCGGTCATCTGTGGTAAGACTAGAGAAACCGTTCTACTCTATATCCTCATACACAATCG
CCTAGCTGCAGCCCAGGTCTA

>p2_ind4222

TCACTTTCGTTATCCAGATCGGTCATCTGTGGTAAGACTAGAGAAACCGTTCTACTCTATATCCTCATACACAATCG
CCTAGCTGCAGCCCAGGTCTA

>p2_ind2825

TCACTTTCGTTATCCAGATCGGTCATCTGTGGTAAGACTAGAGAAACCGTTCTACTCTATATCCTCATACACAATCG
CCTAGCTGCAGCCCAGGTCTA

>p2_ind1786

TCACTTTCGTTATCCAGATCGGTCATCTGTGGTAAGACTAGAGAAACCGTTCTACTCTATATCCTCATACACAATCG
CCTAGCTGCAGCCCAGGTCTA

>p2_ind1829

TCATTTTCGTTATCCAGATCGGTCATCTGTGGTAAGACTAGAGAAACCGTTCTACTCTATATCCTCATACACAATCG
CCTAGCTGCAGCCCAGGTCTA

>p2_ind2753

TCACTTTCGTTATCCAGATCGGTCATCTGTGGTAAGACTAGAGAAACCGTTCTACTCTATATCCTCATACACAATCG
CCTAGCTGCAGCCTAGGTCTA

>p2_ind3171

TCACTTTCGTTATCCAGATCGGTCATCTGTGGTAAGACTAGAGAAACCGTTCTACTCAATATCCTCATACACAATCG
CCTAGCTGCAGCCCAGGTCTA

>p2_ind4408

TCACTTTCGTTATCCAGATCGGTCATCTGCGGTAAGACTAGAGAAACCGTTCTACTCTATATCCTCATACACAATCG
CCTAGCTGCAGCCCAGGTCTA

>p2_ind1383

TCACTTTCGTTATCCAGATCGGTCATCTGTGGTAAGCCTAGAGAAACCGTTCTACTCTATATCCTCATACACAATCG
CCTAGCTGCAGCCTAGGTCTA

>p2_ind1512

TCACTTTCGTTATCCAGATCGGTCATCTGTGGTAAGACTAGAGGAACCGTTCTACTCTATATCCTCATACACAATCG
CCTAGCTGCAGCCCATGTCTA

>p2_ind4942

TCACTTTCGTTATCCAGATCGGTCATCCGCGGTAAGACTAGAGAAACCGTTCTACTCTATATCCTCATACACAATCG
CCTAGCTGCAGCCCAGGTCTA

>p2_ind1243

TCACTTTCGTTATCCAGATCGGTCATCTGTGGTAAGACTAGAGAAACCGTTCTACTCTATATCCTCATACACAATCG
CCTAGCTGCAGCCCAGGTCTA

>p2_ind680

TCACTTTCGTTATCCAGATCGGTCATCTGTGGTAAGACTAGAGAAACCGTTCTACTCTATATCCTCATACACAATCG
CCTAGCTGCGGCCCAGGTCTA

>p2_ind677

TCACTTTCGTTATCCAGATCGGTCATCTGTGGTAAGATTAGAGAAACCGTTCTACTCTATATCCTCATACACAATCG
CCTAGCTGCAGCCCAGGTCTA

>p2_ind1967

TCACTTTCGTTATCCAGATCGGTCATCTGTGGTAAGACTAGAGATACCGTTCTACTCTATATCCTCATACACAATCGC
CTAGCTGCAGCCCAGGTCTA

>p2_ind4673

TCACTTTCGTTATCCAGATCGGTCATCTGTGGTAAGACTAGAGGAACCGTTCTACTCTATATCCTCATACACAATCG
CCTAGCTGCAGCCCATGTCTA

>p2_ind770

TCACTTTCGTTATCCAGATCGGTCATCTGTGGTAAGACTAGAGAAACCGTTCTACTCTATATCCTCATACACAATCG
CCTAGCTGCAGCCCAGGTCTA

>p2_ind3509

TCACTTTCGTTATCCAGATCGGTCATCTGTGGTAAGACTTGAGAATCCGTTCTACTCTATATCCTCATACACAATCGC
CTAGCTGCAGCCCAGGTCTA

>p2_ind2965

TCACTTTCGTTATCCAGATCGGTCATCTGTGGTAAGACTAGAGGAACCATTCTACTCTATATCCTCATACACAATCG
CCTAGCTGCAGCCCAGGTCTA

>p2_ind4446

TCACTTTCGTTATCCAGATCGGTCATCTGCGGTAAGACTAGAGAAACCGTTCTACTCTATATCCTCATACACAATCG
CCTAGCTGCAGCCCAGGTCTA

>p2_ind4583

TCACTTTCGTTATCCAGATCGGTCATCTGTGGTAAGACTAGAGAAACCGTTCTACTCTATATCCTCATACACAATCG
CCTAGCTGCAGCCTAGGTCTA

>p2_ind3872

TCACTTTCGTTATCCAGATCGGTCATCCGCGGTAAGACTAGAGAAACCGTTCTACTCTATATCCTCATACACAATCG
CCTAGCTGCAGCCCAGGTCTA

>p2_ind2347

TCATTTTCGTTATCCAGATCGGTCATCTGTGGTAAGACTAAAGAAACCGTTCTACTCTATATCCTCATACACAATCGC
CTAGCTGCAGCCCAGGTCTA

>p2_ind2094

TCACTTTCGTTATCCAGATCGGTCATCCGCGGTAAGACTAGAGAAACCGTTCTACTCTATATCCTCATACACAATCG
GCTAGCTGCAGCCCAGGTCTA

>p2_ind180

TCACTTTCGTTATCCAGATCGGTCATCTGTGGTAAGACTAGAGAAACCGCTCTACTCTATATCCTCATACACAATCG
CCTAGCTGCAGCCCAGGTCTA

>p2_ind1771

TCACTTTCGTTATCCAGATCGGTCATCTGCGGTAAGACTAGAGAAACCGTTCTACTCTATATCCTCATACACAATCG
CCTAGCTGCAGCCCAGGTCTA

>p2_ind3374

TCACTATCGTTATCCAGATCGGTCATCTGTGGTAAGATTAGAGAAACCGTTCTACTCTATATCCTCATACACAATCG
CCTAGCTGCAGCCCAGGTCTA

>p2_ind1428

TCATTTTCGTTATCCAGATCGGTCATCTGTGGTAAGACTAGAGGAACCATCATACTCTATAACCTCATACACAATCG
CCTAGCTGCAGCCCAGGTCTA

>p2_ind1867

TCACTTTCGTTATCCAGATCGGTCATCTGTGGTAAGACTAGAGAAACCGTTCTACTCTATATCCTCATACACAATCG
CCTAGCTGCAGCCCAGGTCTA

>p2_ind2629

TCACTTTCGTTATCCAGATCGGTCATCTGTGGTAAGACTAAAGAAACCGTTCTACTCTATATCCTCATACACAATCG
CCTAGCTGCAGCCCAGGTCTA

>p2_ind3597

TCACTTTCGTTATCCAGATCGGTCATCTGTGGTAAGACTAGAGAAACCGTTCTACTCTATATCCTCATACACAATCG
CCTAGCTGCAGCCCATGTCTA

>p2_ind4760

TCACTTTCGTTATCCAGATCGGTCATCTGTGGTAAGACTAGAGGAACCGTTCTACTCTATATCCTCATACACAATCG
CCTAGCTGCAGCCCATGTCTA

>p2_ind1161

TCACTTTCGTTATCCAGATCGGTCATGTGTGGTAAGACTAGAGGAACCATTCTACTCTATATCCTCATACACAATCG
CCTAGCTGCAGCCCAGGTCTA

>p2_ind3817

TCACTTTCGTTATCCAGATCGGTCATCTGCGGTAAGACTAGAGAAACCGTTCTACTCTATATCCTCATACACAATCG
CCTAGCTGCAGCCCAGGTCTA

>p2_ind3263

TCACTTTCGTTATCCAGATCGGTCATCTGTGGTAAGACTAGAGACACCGTTCTACTCTATATCCTCATACACAATCG
CCTAGCTGCAGCCCAGGTCTA

>p2_ind1690

TCACTTTCGTTATCCAGATCGGTCATCTGTGGTAAGACTAGAGAAACCGTTCTACTCTATATCCTCATACACAATCG
CCTAGCTGCAGCCCAGGTCTA

>p2_ind1703

TCACTTTCGTTATCCAGATCGGTCATCTGTGGTAAGACTAGAGAAACCGCTCTACTCTATATCCTCATACACAATCG
CCTAGCTGCAGCCCAGGTCTA

>p2_ind552

TCACTTTCGTTATCCAGATCGGTCATCTGTGGTAAGACTAGAGAAACCGTTCTACTCTATATCCTCATACACAATCG
CCTAGCTGCGGCCCAGGTCTA

>p2_ind2771

TCACTTTCGTTATCCAGATCGGTCATCTGTGGTAAGACTAGAGAAACCGTTCTACTCTATATCCTCATACACAATCG
CCTAGCTGCAGCCCAGGTCTA

>p2_ind296

TCACTTTCGTTATCCAGATCGGTCATCTGTGGTAAGACTAGAGAAACCGTTCTACTCTATATCCTCATACACAATCG
CCTAGCTGCAGGCCAGGTCTA

>p2_ind1895

TCACTTTCGTTATCCAGATCGGTCATCTGTGGTAAGACTAGAGAAACCGTTCTACTCTATATCCTCATACACAATCG
CCTAGCTGCGGCCCAGGTCTA

>p2_ind3721

TCACTTTCGTTATCCAGATCGGTCATCTGTGGTAAGACTAGAGAAACCGTTCTACTCTATATCCTCATACACAATCG
CCTAGCTGCAGCCCAGGTCTA

>p2_ind3153

TCACTATCGTTATCCAGATCGGTCATCTGTGGTAAGATTAGAGAAACCGTTCTACTCTATATCCTCATACACAATCG
CCTAGCTGCAGCCCAGGTCTA

>p2_ind2464

TCACTTTCGTTATCCAGATCGGTCATCTGTGGTAAGACTAGAGAAACCGCTCTACTCTATATCCTCATACACAATCG
CCTAGCTGCAGCCCAGGTCTA

>p2_ind163

TCACTTTCGTTATCCAGATCGGTCATCCGCGGTAAGACTAGAGAAACCGTTCTACTCTATATCCTCATACACAATCG
CCTAGCTGCAGCCCAGGTCTA

>p2_ind1980

TCACTTTCGTTATCCAGATCGGTCATCTGTGGTAAGACTAGAGAAACCGTTCTACTCTATATCCTCATACACAATCG
CCTAGCTGCAGCCCAGGTCTA

>p2_ind3649

TCACTTTCGTTATCCAGATCGGTAATCTGCGGTAAGACTAGAGAAACCGTTCTACTCTATATCCTCATACACAATCG
CCTAGCTGCAGCCCAGGTCTA

>p2_ind3065

TCACTTTCGTTATCCAGATCGGTCATCTGTGGTAAGACTAGAGAAACCGTTCTACTCTATATCCTCATACACAATCG
CCTAGCTGCAGCCCAGGTCTA

>p2_ind4197

TCACTATCGTTATCCAGATCGGTCATCTGTGGTAAGACTAGAGAAACCGTTCTACTCTATATCCTCATACACAATCG
CCTAGCTGCAGCCCAGGTCTA

>p2_ind3051

TCACTTTCGTTATCCAGATCGGTCATCTGTGGTAAGACTAGAGAATCCGTTCTACTCTATATCCTCATACACAATCGC
CTAGCTGCAGCCCAGGTCTA

>p2_ind2459

TCACTTTCGTTATCCAGATCGGTCATCTGTGGTAAGACTAGAGAAACCGTTCTACTCTATATCCTCATACACAATCG
CCTAGCTGCAGCCCAGGTCTA

>p2_ind4810

TCACTATCGTTATCCAGATCGGTCATCTGTGGTAAGATTAGAGAAACCGTTCTACTCTATATCCTCATACACAATCG
CCTAGCTGCAGCCCAGGTCTA

>p2_ind2262

TCACTTTCGTTATCCAGATCGGTCATCCGTGGTAAGACTAGAGAAACTGTTCTACTCTATATCCTCATACACAATCG
CCTAGCTGCAGCCCAGGTCTA

>p2_ind3996

TCACTTTCGTTATCCAGATCGGTCATCTGTGGTAAGACTAGAGGAACCGTTCTACTCTATATCCTCATACACAATCG
CCTAGCTGCAGCCCATGTCTA

>p2_ind4949

TCACTTTCGTTATCCAGATCGGTCATCTGTGGTAAGACTAGAGAATCCGTTCTAATCTATATCCTCATACACAATCG
CCTAGCTGCAGCCCAGGTCTA

>p2_ind2339

TCACTTTCGTTATCCAGATCGGTCATCTGTGGTAAGACTAGAGGAACCATTCTACTCTATATCCTCATACACAATCG
CCTAGCTGCAGCCCAGGTCTA

>p2_ind3335

TCACTTTCGTTATCCAGATCGGTCATCTGTGGTAAGACTAGAGAAACCGTTCTACTCTATATCGTCATACACAATCG
CCTAGCTGCAGCCCAGGTCTA

>p2_ind3300

TCACTTTCGTAATCCAGATCGGTCATCCGCGGTAAGACTAGAGAAACCGTTCTACTCTATATCCTCATACACAATCG
CCTAGCTGCAGCCCAGGTCTA

>p2_ind2468

TCACTTTCGTTATCCAGATCGGTCATCTGTGGTAAGACTAGAGAAACCGTTCTACTCTATATCCTCATATACAATCG
CCTAGCTGCAGCCCATGTCTA

>p2_ind4474

TCACTTTCGTTATCCAGATCGGTAATCTGCGGTAAGACTAGAGAATCCGTTCTACTCTATATCCTCATACACAATCG
CCTAGCTGCAGCCCAGGTCTA

>p2_ind2801

TCACTTTCGTTATCCAGATCGGTCATCTGTGGTAAGATTAGAGAAACCGTTCTACTCTATATCCTCATACACAATCG
CCTAGCTGCAGCCCAGGTCTA

>p2_ind3346

TCACTTTCGTTATCCAGATCGGTCATCTGCGGTAAGACTAGAGAAACCGTTCTACTCTATATCCTCATACACAATCG
CCTAGCTGCAGCCCAGGTCTA

>p2_ind1938

TCACTTTCGTTATCCAGATCGGTCATCTGTGGTAAGACTAGAGAAACCGTTCTACTCTATATCCTCATACACAATCG
CCTAGCTGCGGCCCAGGTCTA

>p2_ind2603

TCACTTTCGTTATCCAGATCGGTCATCTGTGGTAAGACTAGAGAAACCGTTCTACTCTATATCCTCATACACAATCG
CCTAGCTGCAGCCCAGGTCTA

>p2_ind2686

TCATTTTCGTTATCCAGATCGGTCATCTGTGGTAAGACTAGAGGAACCATCATACTCTATAACCTCATACACAATCG
CCTAGCTGCAGCCCAGGTCTA

>p2_ind548

TCACTTTCGTTATCCAGATCGGTCATCTGTGGTAAGACTAGAGAAACCGTTCTACTCTATATCCTCATACACAATCG
CCTAGCTGAAGCCCAGGTCTA

>p2_ind1919

TCACTTTCGTTATCCAGATCGGTCATCTGTGGTAAGACTAGAGAAACCGTTCTACTCTATATCCTCATACACAATCG
CCTAGCTGCAGCCCAGGTCTA

>p2_ind3947

TCACTTTCGTTATCCAGATCGGTCATCTGTGGTAAGACTAGAGAAACCGTTCTACTCTATATCCTCATACACAATCG
CCTAGCTGCGGCCCAGGTCTA

>p2_ind3316

TCACTTTCGTTATCCAGATCGGTCATCTGTGGTAAGACTAGAGAAACCGCTCTACTCTATATCCTCATACACAATCG
CCTAGCTGCAGCCCAGGTCTA

>p2_ind4552

TCACTTTCGTTATCCAGATCGGTCATCTGTGGTAAGACTAGAGAAACCGTTCTACTCTATATCCTCATACACAATCG
CCTAGCTGCAGCCCAGGTCTA

>p2_ind3982

TCACTTTCGTTATCCAGATCGGTCATCTGTGGTAAGACTTGAGAATCCGTTCTAATCTATATCCTCATACACAATCGC
CTAGCTGCAGCCCAGGTCTA

>p2_ind474

TCACTTTCGTTATCCAGATCGGTCATCTGTGGTAAGACTAGAGAAACCGTTCTACTCTATATCCTCATACACAATCG
CCTAGCTGCAGGCCAGGTCTA

>p2_ind187

TCACTTTCGTTATCCAGATCGGTCATCTGTGGTAAGACTAGAGAAACCGTTCTACTCTATATCCTCATACACAATCG
CCTAGCTGCAGCCCAGGTCTA

>p2_ind3102

TCACTTTCGTTATCCAGATCGGTCATCTGTGGTAAGACTAGAGAAACCGTTCTACTCTATATCCTCATACACAATCG
CCTAGCTGCAGCCCAGGTCTA

>p2_ind3261

TCACTTTCGTTATCCAGATCGGTCATCTGTGGTAAGACTAGAGAAACCGTTCTACTCTATATCCTCATACACAATCG
CCTAGCTGCAGCCCAGGTCTA

>p2_ind3402

TCACTTTCGTTATCCAGATCGGTCATCTGTGGTAAGACTAGAGATACCGTTCTACTCTATATCCTCATACACAATCGC
CTAGCTGCAGCCCAGGTCTA

>p2_ind733

TCACTTTCGTTATCCAGATCGGTCATCTGTGGTAAGACTAGAGAAACCGTTCTACACTATATCCTCATACACAATCG
CCTAGCTGCAGCCCAGGTCTA

>p2_ind373

TCACTTTCGTTATCCAGATCGGTCATCTGTGGTAAGACTAGAGAAACCGTTCTACTCTATATCCTCATACACAATCG
CCTAGCTGCAGCCCAGGTCTA

>p2_ind2585

TCATTTTCGTTATCCAGATCGGTCATCTGTGGTAAGACTAGAGGAACCGTTCTACTCTATATCCTCATACACAATCG
CCTAGCTGCAGCCCAGGTCTA

>p2_ind1015

TCACTTTCGTTATCCAGATCGGTCATCTGTGGTAAGACCAGAGAAACCGTTTTACTCTATATCCTCATACACAATCG
CCTAGCTGCAGCCCAGGTCTA

>p2_ind4325

TCACTTTCGTTATCCAGATCGGTCATCTGTGGTAAGACCAGAGAAACCGTTTTACTCTATATCCTCATACACAATCG
CCTAGCTGCAGCCCAGGTCTA

>p2_ind40

TCACTATCGTTATCCAGATCGGTCATCTGTGGTAAGATTAGAGAAACCGTTCTACTCTATATCCTCATACACAATCG
CCTAGCTGCAGCCCAGGTCTA

>p2_ind4853

TCACTTTCGTTATCCAGATCGGTCATCTGTGGTAAGACCAGAGAAACCGTTTTACTCTATATCCTCATACACAATCG
CCTAGCTGCAGCCCAGGTCTA

>p2_ind1445

TCACTTTCGTTATCCAGATCGGTCATCTGTGGTAAGACTAGAGAAACCGTTCTACTCTATATCCTCATACACAATCG
CCTAGCTGCAGCCCAGGTCTA

>p2_ind3630

TCACTTTCGTTATCCAGATCGGTCATCTGTGGTAAGACTAGAGAAACCGTTCTACTCTATATCCTCATACACAATCG
CCTAGCTGCAGCCTAGGTCTA

>p2_ind15

TCACTTTCGTTATCCAGATCGGTCATCTGTGGTAAGACTAGAGGAACCATTCTACTCTATATCCTCATACACAATCG
CCTAGCTGCAGCCCAGGTCTA

>p2_ind191

TCACTTTCGTTATCCAGATCGGTCATCTGTGGTAAGACTAGAGAAACCGTTCTACTCAATATCCTCATACACAATCG
CCTAGCTGCAGCCCAGGTCTA

>p2_ind1353

TCACTTTCGTTATCCAGATCGGTCATCTGTGGTAAGACTAGAGTAACCGTTCTACTCTATATCCTCATACACAATCGC
CTAGCTGCAGCCCAGGTCTA

>p2_ind1504

TCACTTTCGTTATCCAGATCGGTCATCTGTGGTAAGACTAGAGAAACCGTTCTACTCTATATCCTCATACACAATCG
CCTAGCTGCAGCCCAGGTCTA

>p2_ind2446

TCACTTTCGTTATCCAGATCGGTCATCTGTGGTAAGACTAGAGAAACCGTTCTACTCTATATCCTCATACACAATCG
CCTAGCTGCAGCCCAGGTCTA

>p2_ind1483

TCACTTTCGTTATCCAGATCGGTCATCTGTGGTAAGACTAGAGGAACCATTCTACTCTATATCCTCATACACAATCG
CCTAGCTGCAGCCCAGGTCTA

>p2_ind2326

TCACTTTCGTTATCCAGATCGGTCATCTGTGGTAAGACTAGAGAAACCGTTCTACTCTATATCCTCATACACAATCG
CCTAGCTGCAGCCCAGGTCTA

>p2_ind3911

TCATTTTCGTTATCCAGATCGGTCATCTGTGGTAAGACTAGAGGAACCATCATACTCTATAACCTCATACACAATCG
CCTAGCTGCAGCCCAGGTCTA

>p2_ind783

TCACTTTCGTTATCCAGATCGGTCATCTGTGGTAAGACTAGAGAAACCGTTCTACTCTATATCCTCATACACAATCG
CCTAGCTGCAGCCCAGGTCTA

>p2_ind1364

TCACTTTCGTTATCCAGATCGGTCATCTGTGGTAAGACTAGAGAAACCGTTCTACTCTATATCCTCATACACAATCG
CCTAGCTGCAGCCCAGGTCTA

>p2_ind1086

TCACTTTCGTTATCCAGATCGGTCATCTGTGGTAAGACTAGAGAAACCGCTCTACTCTATATCCTCATACACAATCG
CCTAGCTGCAGCCCAGGTCTA

>p2_ind1028

TCACTTTCGTTATCCAGATCGGTAATCTGCGGTAAGACTAGAGAAACCGTTCTACTCTATATCCTCATACACAATCG
CCTAGCTGAAGCCCAGGTCTA

>p2_ind3609

TCATTTTCGTTATCCAGATCGGTCATCTGTGGTAAGCCTAGAGGAACCATTCTACTCTATATCCTCATACACAATCGC
CTAGCTGCAGCCCAGGTCTA

>p2_ind2717

TCACTTTCGTTATCCAGATCGGTCATCTGTGGTAAGACTAGAGAAACCGTTCTACTCTATATCCTCATACACAATCG
CCTAGCTGCAGCCCAGGTCTA

>p2_ind3751

TCACTTTCGTTATCCAGATCGGTCATCTGTGGTAAGACTAGAGAAACCGTTCTACTCTATATCCTCATACACAATCG
CCTAGCTGCGGCCCAGGTCTA

>p2_ind4072

TCACTTTCGTTATCCAGATCGGTCATCTGTGGTAAGACTAGAGAAACCGTTCTTCTCTATATCCTCATACACAATCGC
CTAGCTGCAGCCCAGGTCTA

>p2_ind2392

TCACTTTCGTTATCCAGATCGGTCATCTGTGGTAAGACTAGAGAATCCGTTCTACTCTATATCCTCATACACAATCGC
CTAGCTGCAGCCCAGGTCTA

>p2_ind3950

TCACTTTCGTTATCCAGATCGGTCATCTGTGGTAAGACTAGAGGAACCGTTCTACTCTATATCCTCATACACAATCG
CCTAGCTGCAGCCCAGGTCTA

>p2_ind2593

TCACTTTCGTTATCCAGATCGGTCATCTGTGGTAAGACTAGAGAAACCGTTCTACTCTATATCCTCATACACAATCG
CCTAGCTGCAGCCCAGGTCTA

>p2_ind339

TCACTTTCGTTATCCAGATCGGTCATCTGTGGTAAGACTAGAGAAACCGTTCTACTCTATATCCTCATACACAATCG
CCTAGCTGCAGCCCAGGTCTA

>p2_ind286

TCACTTTCGTTATCCAGATCGGTCATCTGTGGTAAGACTAGAGAAACCGTTCTACTCTATATCCTCATACACAATCG
CCTAGCTGCAGCCCAGGTCTA

>p2_ind2936

TCACTATCGTTATCCAGATCGGTCATCTGTGGTAAGATTAGAGAAACCGTTCTACTCTATATCCTCATACACAATCG
CCTAGCTGCAGCCCAGGTCTA

>p2_ind3377

TCACTTTCGTTATCCAGATCGGTCATCTGTGGTAAGACTAGAGAAACCGTTCTACTCTATATCCTCATACACAATCG
CCTAGCTGCAGCCCAGGTCTA

>p2_ind1952

TCACTTTCGCTATCCAGATCGGTCATCTGTGGTAAGACTAGAGAAACCGTTCTACTCTATATCCTCATACACAATCG
CCTAGCTGCAGGCCAGGTCTA

>p2_ind1255

TCACTTTCGTTATCCAGATCGGTCATCTGTGGTAAGACTAGAGAAACCGTTCTACTCTATATCCTCATACACAATCG
CCTAGCTGCAGCCCAGGTCTA

>p2_ind4550

TCACTTTCGTTATCCAGATCGGTCATCTGTGGTAAGACTAGAGAAACCGTTCTACTCTATATCCTCATACACAATCG
CCTAGCTGCAGCCCAGGTCTA

>p2_ind3186

TCACTTTCGTTATCCAGATCGGTCATCTGTGGTAAGACTAGAGAAACCGTTCTACTCTATATCCTCATACACAATCG
CCTAGCTGCAGCCCAGGTCTA

>p2_ind3331

TCACTTTCGTTATCCAGATCGGTCATCTGTGGTAAGACTAGAGAAACCGTTCTACTCTATATCCTCATACACAATCG
CCTAGCTGCAGCCCAGGTCTA

>p2_ind3404

TCACTATCGTTATCCAGATCGGTCATCTGTGGTAAGATTAGAGAAACCGTTCTACTCTATATCCTCATACACAATCG
CCTAGCTGCAGCCCAGGTCTA

>p2_ind1085

TCACTTTCGTTATCCAGATCGGTCATCTGTGGTAAGACTAGAGAAACCGTTCTACTCTATATCCTCATACACAATCG
CCTAGCTGCAGCCCAGGTCTA

>p2_ind3087

TCACTATCGTTATCCAGATCGGTCATCTGTGGTAAGATTAGAGAAACCGTTCTACTCTATATCCTCATACACAATCG
CCTAGCTGCAGCCCAGGTCTA

>p2_ind4677

TCACTTTCGTTATCCAGATCGGTCATCTGTGGTAAGACTAGAGAAACCGTTCTACTCTATATCCTCATACACAATCG
CCTAGCTGCAGCCCAGGTCTA

>p2_ind142

TCACTTTCGTTATCCAGATCGGTCATCTACGGTAAGACTAGAGAAACCGTTCTACTCTATATCCTCATACACAATCG
CCTAGCTGCAGCCCAGGTCTA

>p2_ind2998

TCACTTTCGTTATCCAGATCGGTCATCCGCGGTAAGACTAGAGAAACCGCTCTACTCTATATCCTCATACACAATCG
CCTAGCTGCAGCCCAGGTCTA

>p2_ind3809

TCACTTTCGTTATCCAGATCGGTCATCTGTGGTAAGACTAGAGAAACCGTTCTACTCTATATCCTCATACACAATCG
CCTAGCTGCAGCCCAGGTCTA

>p2_ind3577

TCACTTTCGTTATCCAGATCGGTCATCTGCGGTAAGACTAGAGAAACCGTTCTACTCTATATCCTCATACACAATCG
CCTAGCTGCAGCCCAGGTCTA

>p2_ind1

TCACTTTCGTTATCCAGATCGGTCATCTGTGGTAAGACTAGAGAAACCGTTCTACTCTATATCCTCATACACAATCG
CCTAGCTGCAGCCCAGGTCTA

>p2_ind2243

TCACTTTCGTTATCCAGATCGGTCATCTGTGGTAAGACTAGAGAAACCGTTCTACTCTATATCCTCATACACAATCG
CCTAGCTGAAGCCCAGGTCTA

>p2_ind1963

TCACTTTCGTTATCCAGATCGGTCATCTGTGGTAAGACTAGAGGAACCATTCTACTCTATATCCTCATACACAATCG
CCTAGCTGCAGCCCAGGTCTA

>p2_ind1157

TCACTTTCGTTATTCAGATCGGTCATCTGTGGTAAGACTAGAGAAACCGTTCTACTCTATATCCTCATACACAATCG
CCTAGCTGCAGCCCAGCTCTA

>p2_ind3178

TCACTTTCGTTATCCAGATCGGTCATCTGTGGTAAGACTAGAGAAACCGTTCTACTCTATATCCTCATACACAATCG
CCTAGCTGCAGCCCAGGTCTA

>p2_ind278

TCACTTTCATTATCCAGATCGGTCATCTGTGGTAAGACTAGAGAAACCGTTCTACTCTATATCCTCATACACAATCG
CCTAGCTGCAGCCCAGGTCTA

>p2_ind4688

TCACTATCGTTATCCAGATCGGTCATCTGTGGTAAGACTAGAGAAACCGTTCTACTCTATATCCTCATACACAATCG
CCTAGCTGCAGCCCAGGTCTA

>p2_ind653

TCACTTTCGTTATCCAGATCGGTCATCTGTGGTAAGACTAGAGAAACCGTTCTACTCTATATCCTCATACACAATCG
CCTAGCTGCAGCCCAGGTCTA

>p2_ind3030

TCACTTTCGTTATCCAGATCGGTCATCTGTGGTAAGACTAGAGTAACCGTTCTACTCTATATCCTCATACACAATCGC
CTAGCTGCAGCCCAGGTCTA

>p2_ind4668

TCACTTTCGTTATCCAGATCGGTCATCTGTGGTAAGACTAGAGAAACCGTTCTACTCTATATCCTCATACACAATCG
CCTAGCTGCAGCCCAGGTCTA

>p2_ind2190

TCACTTTCGTTGTCCAGATCGGTCATCTGTGGTAAGACTAGATAAACCGTTCTACTCTATATCCCCATACACAATCAC
CTAGCTGCAGCCCAGGTCTA

>p2_ind220

TCACTATCGTTATCCAGATCGGTCATCTGTGGTAAGACTAGAGAAACCGTTCTACTCTATATCCTCATACACAATCG
CCTAGCTGCAGCCCAGGTCTA

>p2_ind1939

TCACTTTCGTTATCCAGATCGGTCATCTGTGGTAAGACTAGAGAAACCGTTCTACTCTATATCCTCATACACAATCG
CCTAGCTGCAGCCCAGGTCTA

>p2_ind657

TCACTTTCGTTATCCAGATCGGTCATCTGTGGTAAGACTAGAGTAACCGTTCTACTCTATATCCTCATACACAATCGC
CTAGCTGCAGCCCAGGTCTA

>p2_ind4336

TCACTATCGTTATCCAGATCGGTCATCTGTGGTAAGATTAGAGAAACCGTTCTACTCTATATCCTCATACACAATCG
CCTAGCTGCAGCCCAGGTCTA

>p2_ind1226

TCACTATCGTTATCCAGATCGGTCATCTGTGGTAAGATTAGAGAAACCGTTCTACTCTATATCCTCATACACAATCG
CCTAGCTGCAGCCCAGGTCTA

>p2_ind719

TCACTTTCGTTATCCAGATCGGTCATCTGTGGTAAGACTAGAGAAACCGTTCTACTCTATATCCTCATACACAATCG
CCTAGCTGCAGCCCAGGTCTA

>p2_ind1219

TCACTTTCGTTGTCCAGATCGGTCATCTGTGGTAAGACTAGATAAACCGTTCTACTCTATATCCCCATACACAATCG
CCTAGCTGCAGCCCAGGTCTA

>p2_ind440

TCACTTTCGTTATCCAGATCGGTCATCTGTGGTAAGACTAGAGAAACCGTTCTACTCTATATCCTCATACACAATCG
CCTAGCTGCGGCCCAGGTCTA

>p2_ind194

TCACGTTCGTTATCCAGATCGGTCATCTGTGGTAAGACTAGAGAAACCGTTCTACTCTATATCCTCATACACAATCG
CCTAGCTGCAGCCCAGGTCTA

>p2_ind3699

TCACTATCGTTATCCAGATCGGTCATCTGTGGTAAGATTAGAGAAACCGTTCTACTCTATATCCTCATACACAATCG
CCTAGCTGCAGCCCAGGTCTA

>p2_ind3389

TCACTTTCGTTATCCAGATCGGTCATCTGTGGTAAGACTAGAGAAACCGTTCTACTCTATATCCTCATACACAATCG
CCTAGCTGCAGCCCAGGTCTA

>p2_ind1343

TCACTTTCGTTATCCAGATCGGTCATCTGCGGTAAGACTAGAGAAACCGTTCTACTCTATATCCTCATACACAATCG
CCTAGCTGCAGCCCAGGTCTA

>p2_ind716

TCACTTTCGTTATCCAGATCGGTCATCTGTGGTAAGACTAGAGAAACCGTTCTACTCTATATCCTCATACACAATCG
CCTAGCTGCAGCCCAGGTCTA

>p2_ind2144

TCACTTTCGTTATCCAGATCGGTCATCTGTGGTAAGACTAGAGAAACCGTTCTACTCTATATCCTCATACACAATCG
CCTAGCTGCAGCCCAGGTCTA

>p2_ind72

TCACTTTCGTTATCCCGATCGGTCATCTGTGGTAAGACTAGAGAAACCGTTCTACTCTATATCCTCATACACAATCG
CCTAGCTGCAGCCCAGGTCTA

>p2_ind4773

TCACTTTCGTTATCCAGATCGGTCATCTGTGGTAAGACTAGAGAAACCGCTCTACTCTATATCCTCATACACAATCG
CCTAGCTGCAGCCCAGGTCTA

>p2_ind3676

TCACTTTCGTTATCCAGATCGGTCATCTGCGGTAAGACTAGAGAAACCGTTCTACTCTATATCCTCATACACAATCG
CCTAGCTGCAGCCCAGGTCTA

>p2_ind3552

TCACTTTCGTTATCCAGATCGGTCATCTGTGGTAAGACTAGAGAAACCGTTCTACTCTATATCCTCATACACAATCG
CCTAGCTGCAGCCTAGGTCTA

>p2_ind2319

TCACTTTCGTTATCCAGATCGGTCATCTGTGGTAAGACTAGAGAAACCGTTCTACTCTATATCCTCATACACAATCG
CCTAGCTGCAGCCAAGGTCTA

>p2_ind2462

TCACTTTCGTTATCCAGATCGGTCATCTGTGGTAAGACTAGAGAAACCGTTCTACTCTATATCCTCATACACAATCG
CCTAGCTGCAGCCTAGGTCTA

>p2_ind4519

TCACTTTCGTTATCCAGATCGGTCATCCGCGGTAAGACTAGAGAAACCGTTCTACTCTATATCCTCATACACAATCG
CCTAGCTGCAGCCCAGGTCTA

>p2_ind905

TCACTTTCGTTATCCAGATCGGTCATCTGTGGTAAGACTAGAGAAACCGTTCTACTCTATATCCTCATACACAATCG
CCTAGCTGCAGCCCAGGTCTA

>p2_ind4899

TCACTTTCATTATCCAGATCGGTCATCTGTGGTAAGACTAGAGAAACCGTTCTACTCTATATCCTCATACACAATCG
CCTAGCTGCAGCCCAGGTCTA

>p2_ind426

TCACTTTCGTTATCCAGATCGGTCATCTGTGGTAAGACTAGAGAAACCGTTCTACTCTATATCCTCATACACAATCG
CCTAGCTGCAGCCCAGGTCTA

>p2_ind1203

TCATTTTCGTTATCCAGATCGGTCATCTGTGGTAAGACTAGAGGAACCATCATACTCTATAACCTCATACACAATCG
CCTAGCTGCAGCCCAGGTCTA

>p2_ind4690

TCACTTTCGTTATCCAGATCGGTCATCTGTGGTAAGACTAGAGAAACCGTTCTACTCTATATCCTCATACACAATCG
CCTAGCTGCAGCCCAGGTCTA

>p2_ind338

TCACTTTCGTTATCCAGATCGGTCATCTGTGGTAAGATTAGAGAAACCGTTCTACTCTATATCCTCATACACAATCG
CCTAGCTGCAGCCCAGGTCTA

>p2_ind3421

TCACTTTCGTTATCCAGATCGGTCATCTGTGGTAAGACTTGAGAATCCGTTCTAATCTATATCCTCATACACAATCGC
CTAGCTGCAGCCCAGGTCTA

>p2_ind863

TCACTTTCGTTATCCAGATCGGTCATCTGTGGTAAGACTAGAGAAACCGTTCTACTCTATATCCTCATACACAATCG
CCTAGCTGCAGCCCAGGTCTA

>p2_ind2168

TCACTTTCGTTATCCAGATCGGTCATCTGTGGTAAGACTAGAGAAACCGTTCTACTCTATATCCTCATACACAATCG
CCTAGCTGCAGCCTAGGTCTA

>p2_ind954

TCACTTTCGTTATCCAGATCGGTCATCTGTGGTAAGACTAGAGTAACCGTTCTACTCTATATCCTCATACACAATCGC
CTAGCTGCAGCCCAGGTCTA

>p2_ind3011

TCACTTTCGTTATCCAGATCGGTCATCTGTGGTAAGACTAGAGAAACCGTTCTACTCTATATCCTCATACACAATCG
CCTAGCTGCAGCCCAGGTCTA

>p2_ind1694

TCACTTTCGTTATCCAGATCGGTCATCTGTGGTAAGACTAGAGAAACCATTCTACTCTATATCCTCATACACAATCG
CCTAGCTGCAGCCCAGGTCTA

>p2_ind4770

TCACTTTCGTTATCCAGATCGGTCATCTGTGGTAAGACTAGAGAAACCGTTCTACTCTATATCCTCATACACAATCG
CCTAGCTGCAGCCCAGGTCTA

>p2_ind4068

TCACTTTCGTTATCCAGATCGGTCATCCGCGGTAAGACTAGAGAAACCGTTCTACTCTATATCCTCATACACAATCG
CCTAGCTGCAGCCCAGGTCTA

>p2_ind2613

TCACTTTCGTTATCCAGATCGGTCATCTGTGGTAAGACTAGAGGAACCATTCTACTCTATATCCTCATACACAATCG
CCTAGCTGCAGCCCAGGTCTA

>p2_ind1729

TCACTTTCGTTATCCAGATCGGTCATCTGTGGTAAGACTAGAGAAACCGTTCTACTCTATATCCTCATACACAATCG
CCTAGCTGCAGCCCAGGTCTA

>p2_ind4079

TCACTTTCGTTATCCAGATCGGTCATCTGTGGTAAGACTAGAGGAACCATTCTACTCTATATCCTCATACACAATCG
CCTAGCTGCAGCCCAGGTCTA

>p2_ind4897

TCACTTTCGTTATCCAGATCGGTCATCTGTGGTAAGACTAGAGTAACCGTTCTACTCTATATCCTCATACACAATCGC
CTAGCTGCAGCCCAGGTCTA

>p2_ind4438

TCACTTTCGTTATCCAGATCGGTCATCTGTGGTAAGACTAGAGAAACCGTTCTACTCTATATCCTCATACACAATCG
CCTAGCTGCAGCCCAGGTCTA

>p2_ind3769

TCACTTTCGTTATCCAGATCGGTCATCTGTGGTAAGACTAGAGAAACCGTTCTACTCTATATCCTCATACACAATCG
CCTAGCTGCAGCCCAGGTCTA

>p2_ind4487

TCACTTTCGTTATCCAGATCGGTCATCTGTGGTAAGATTAGAGAAACCGTTCTACTCTATATCCTCATACACAATCG
CCTAGCTGCAGGCCAGGTCTA

>p2_ind1062

TCATTTTCGTTATCCAGATCGGTCATCTGTGGTAAGACTAGAGGAACCATCATACTCTATAACCTCATACACAATCG
CCTAGCTGCAGCCCAGGTCTA

>p2_ind2607

TCACTTTCGTTATCCAGATCGGTCATCTGTGGTAAGACTAGAGGAACCATTCTACTCTATATCCTCATACACAATCG
CCTAGCTGCAGCCCAGGTCTA

>p2_ind3338

TCACTTTCGTTATCCAGATCGGTCATCTGTGGTAAGACTAGAGAAACCGTTCTACTCTATATCCTCATACACAATCG
CCTAGCTGCAGCCCAGGTCTA

>p2_ind2124

TCACTTTCGTTATCCAGATCGGTCATCTGTGGTAAGACTAGAGAAACCGTTCTACTCTATATCCTCATACACAATCG
CCTAGCTGCGGCCCAGGTCTA

>p2_ind4425

TCACTTTCATTATCCAGATCGGTCATCTGTGGTAAGACTAGAGAAACCGTTCTACTCTATATCCTCATACACAATCG
CCTAGCTGCAGCCCAGGTCTA

>p2_ind1569

TCACTTTCGTTATCCAGATCGGTCATCTGTGGTAAGACTAGAGAAACCGTTCTACTCTATATCCTCATACACAATCG
CCTAGCTGCAGCCCAGGTCTA

>p2_ind2958

TCACTTTCGTTATCCAGATCGGTCATCTGTGGTAAGACTAGAGAAACCGTTCTACTCTATATCCTCATACACAATCG
CCTAGCTGCAGCCCAGGTCTA

>p2_ind3289

TCACTTGCGTTATCCAGATCGGTTAGCTGTGGTAAGACTAGAGGAACCATTCTACTCTATATCCTCATACACAATCG
CCTAGCTGCAGCCCAGGTCTA

>p2_ind2518

TCACTTTCGTTATCCAGATCGGTCATCTGTGGTAAGACTAGAGGAACCATTCTACTCTATATCCTCATACACAATCG
CCTAGCTGCAGCCCAGGTCTA

>p2_ind195

TCACTTTCGTTATCCAGATCGGTCATCTGTGGTAAGACTAGAGAAACCGTTCTACTCTATATCCTGATACACAATCG
CCTAGCTGCAGCCCAGGTCTA

>p2_ind2269

TCACTTTCGTTATCCAGATCGGTCATCTGTGGTAAGACTAGAGAAACCGTTCTACTCTATATCCTCATACACAATCG
CCTAGCTGCAGGCCAGGTCTA

>p2_ind4339

TCACTTTCGTTATCCAGATCGGTCATCTGTGGTAAGACTAGATAAACCGTTCTACTCTATATCCTCATACACAATCGC
CTAGCTGCAGCCCATGTCTA

>p2_ind1856

TCACTTTCGTTATCCAGATCGGTCATCTGTGGTAAGACTAGAGAAACCGTTCTACTCTATATCCTCATACACAATCG
CCTAGCTGCAGCCCAGGTCTA

>p2_ind3334

TCACTTTCGTTATCCAGATCGGTCATCTGCGGTAAGACTAGAGAAACCGTTCTACTCTATATCCTCATACACAATCG
CCTAGCTGCAGCCCAGGTCTA

>p2_ind4727

TCACTTTCGTTATCCAGATCGGTCATCTGTGGTAAGACTAGAGAAACCGTTCTACTCTATATCCTCATACACAATCG
CCTAGCTGCAGCCCAGGTCTA

>p2_ind2624

TCACTTTCGTTATCCAGATCGGTCATCTGTGGTAAGACTAGAGAAACCGTTCTACTCTATATCCTCATACACAATCG
CCTAGCTGCGGCCCAGGTCTA

>p2_ind1250

TCACTATCGTTATCCAGATCGGTCATCTGTGGTAAGATTAGAGAAACCGTTCTACTCTATATCCTCATTCACAATCG
CCTAGCTGCAGCCCAGGTCTA

>p2_ind3798

TCACTTTCGTTATCCAGATCGGTCATCTGTGGTAAGACTAGAGAAACCGTTCTACTCTATATCGTCATACACAATCG
CCTAGCTGCAGCCCAGGTCTA

>p2_ind2960

TCACTTTCGTTATCCAGATCGGTCATCTGTGGTAAGACTAGAGAAACCGTTCTACTCTATATCCTCATACACAATCG
CCTAGCTGCAGCCTAGGTCTA

>p2_ind638

TCACTTTCGTTAGCCAGATCGGTCATCTGTGGTAAGACTAGAGAAACCATTCTACTCTATATCCTCATACACAATCG
CCTAGCTGCAGCCCAGGTCTA

>p2_ind2854

TCACTTTCGTTATCCAGATCGGTCATCTGTGGTAAGACTAGAGAAACCGTTCTACTCTATATCCTCATACACAATCG
CCTAGCTGCAGCCCAGGTCTA

>p2_ind245

TCACTTTCGTTATCCAGATCGGTCATCTGTGGTAAGACTAGAGGAACCGTTCTACTCTATATCCTCATACACAATCG
CCTAGCTGCAGCCCAGGTCTA

>p2_ind4724

TCACTTTCGTTATCCAGATCGGTCATCTGTGGTAAGACTAGAGAAACCGTTCTACTCTATATCCTCATACACAATCG
CCTAGCTGCAGCCCAGGTCTA

>p2_ind4943

TCACTTTCGTTATCCAGATCGGTCATCTGTGGTAAGACTAGAGAAACCGTTCTACTCTATATCCTCATACACAATCG
CCTAGCTGCAGCCCAGGTCTA

>p2_ind1923

TCATTTTCGTTATCCAGATCGGTCATCTGTGGTAAGCCTAGAGGAACCATTCTACTCTATATCCTCATACACAATCGC
CTAGCTGCAGCCCAGGTCTA

>p2_ind4206

TCACTTTCGTTATCCAGATCGGTCATCTGTGGTAAGACTAGAGAAACCGTTCTACTCTATATCCTCATACACAATCG
CCTAGCTGCAGCCCAGGTCTA

>p2_ind203

TCACTTTCGTTATCCAGATCGGTCATCTGTGGTAAGACTAGAGAAACCGTTCTACTCTATATCCTCATACACAATCG
CCTAGCTGCAGCCCAGGTCTA

>p2_ind3855

TCACTTTCGTTATCCAGATCGGTCATCTGTGGTAAGACTAGAGAAACCATTCTACTCTATATCCTCATACACAATCG
CCTAGCTGCAGCCCAGGTCTA

>p2_ind4892

TCACTTTCGTTATCCAGATCGGTCATCCGCGGTAAGACTAGAGAAACCGTTCTACTCTAAATCCTCATACACAATCG
CCTAGCTGCAGCCCAGGTCTA

>p2_ind436

TCACTTTCGTTATTCAGATCGGTCATCTGTGGTAAGACTAGAGAAACCGTTCTACTCTATATCCTCATACACAATCG
CCTAGCTGCAGCCCAGCTCTA

>p2_ind4925

TCACTTTCGTTATCCAGATCGGTCATCTGTGGTAAGACTAGAGGAACCATTCTACTCTATATCCTCATACACAATCG
CCTAGCTGCAGCCCAGGTCTA

>p2_ind2775

TCACTTTCGTTATCCAGATCGGTCATCTGTGGTAAGACTAGAGAATCCGTTCTACTCTATATCCTCATACACAATCGC
CTAGCTGCAGCCCAGGTCTA

>p2_ind3069

TCACTATCGTTATCCAGATCGGTCATCTGTGGTAAGATTAGAGAAACCGTTCTACTCTATATCCTCATACACAATCG
CCTAGCTGCAGCCCAGGTCTA

>p2_ind2445

TCACTTTCGTTATCCAGATCGGTCATCTGTGGTAAGACTAGAGAAACCGTTCTACTCTATATCCTCATACACAATCG
CCTAGCTGCAGCCCAGGTCTA

>p2_ind2709

TCACTTTCGTTATCCAGATCGGTCATCTGTGGTAAGACTAGAGAAACCGTTCTACTCTATATCCTCATACACAATCG
CCTAGCTGCAGCCCAGGTCTA

>p2_ind2755

TCACTTTCGTTATCCAGATCGGTCATCTGTGGTAAGACTAGAGAAACCGTTCTACTCTATATCCTCATACACAATCG
CCTAGCTGCAGCCAAGGTCTA

>p2_ind952

TCACTTTCGTTGTCCAGATCGGTCATCTGTGGTAAGACTAGATAAACCGTTCTACTCTATATCCCCATACACAATCG
CCTAGCTGCAGCCCAGGTCTA

>p2_ind3054

TCACTTTCGTTATCCAGATCGGTCATCTGCGGTAAGACTAGAGAAACCGTTCTACTCTATATCCTCATACACAATCG
CCTAGCTGCAGCCCAGGTCTA

>p2_ind3790

TCACTTTCGTTATCCAGATCGGTCATCTGTGGTAAGACTAGAGGAACCATTCTACTCTATATCCTCATACACAATCG
CCTAGCTGCAGCCCAGGTCTA

>p2_ind1276

TCACTTTCGTTATCCAGATCGGTCATCTGTGGTAAGACTAGAGAAACCGTTCTACTCTATATCCTCATACACAATCG
CCTAGCTGCAGGCCAGGTCTA

>p2_ind1386

TCACTTTCGTTATCCAGATCGGTCATCTGTGGTAAGACTAGAGAAACCGTTCTACTCTATATCCTCATACACAATCG
CCTAGCTGCAGCCCAGGTCTA

>p2_ind1213

TCACTTTCGTTATCCAGATCGGTAATCTGCGGTAAGACTAGAGAAACCGTTCTACTCTATATCCTCATACACAATCG
CCTAGCTGCAGCCCAGGTCTA

>p2_ind1489

TCACTATCGTTATCCAGATCGGTCATCTGTGGTAAGATTAGAGAAACCGTTCTACTCTATATCCTCATACACAATCG
CCTAGCTGCAGCCCAGGTCTA

>p2_ind4893

TCATTTTCGTTATCCAGATCGGTCATCTGTGGTAAGACTAGAGGAACCATCATACTCTATAACCTCATACACAATCG
CCTAGCTGCAGCCCAGGTCTA

>p2_ind1728

TCACTTTCGTTATCCAGATCGGTCATCTGTGGTAAGACTAGAGAAACCGTTCTACTCTATATCCTCATACACAATCG
CCTAGCTGCAGCCCAGGTCTA

>p2_ind984

TCACTTTCGTTATCCAGATCGGTCATCTGTGGTAAGACTAGAGAAACCGTTCTACTCTATATCCTCATACACAATCG
CCTAGCTGCAGCCCAGGTCTA

>p2_ind4433

TCACTTTCGTTATCCAGATCGGTCATCTGTGGTAAGACTAGAGAAACCGCTCTACTCTATATCCTCATACACAATCG
CCTAGCTGCAGCCCAGGTCTA

>p2_ind2885

TCACTTTCGTTAGCCAGATCGGTCATCTGTGGTAAGACTAGAGGAACCATTCTACTCTATATCCTCATACACAATCG
CCTAGCTGCAGCCCAGGTCTA

>p2_ind1809

TCACTTTCGTTATCCAGATCGGTCATCTGTGGTAAGACTAGAGAAACCGTTCTACTCTATATCCTCATACACAATCG
CCTAGCTGCGGCCCAGGTCTA

>p2_ind2813

TCACTTTCGTTATCCAGATCGGTCATCTGTGGTAAGACTAGAGAAACCGTTCTACTCTATATCCTCATACACAATCG
CCTAGCTGCAGCCCAGGTCTA

>p2_ind2623

TCACTTTCGTTATTCAGATCGGTCATCTGTGGTAAGACTAGAGAAACCGTTCTACTCTATATCCTCATACACAATCG
CCTAGCTGCAGCCCAGCTCTA

>p2_ind2364

TCACTTTCGTTATCCAGATCGGTCATCTGTGGTAAGACTAGAGAAACCGTTCTACTCTATATCCTCATACACAATCG
CCTAGCTGCAGCCCAGGTCTA

>p2_ind1051

TCACTTTCGTTATCCAGATCGGTCATCTGCGGTAAGACTAGAGAAACCGTTCTACTCTATATCCTCATACACAATCG
CCTAGCTGCAGCCCAGGTCTA

>p2_ind4128

TCACTTTCGTTATCCAGATCGGTCATCTGTGGTAAGACTAGAGAAACCGTTCTACTCTATATCCTCATACACAATCG
CCTAGCTGCAGCCCAGGTCTA

>p2_ind3420

TCACTTTCGTTATCCAGATCGGTAATCTGCGGTAAGACTAGAGAAACCGTTCTACTCTATATCCTCATACACAATCG
CCTAGCTGCAGCCCAGGTCTA

>p2_ind1639

TCACTTTCGTTATCCAGATCGGTCATCTGTGGTAAGACTAGAGAAACCGTTCTACTCTATATCCTCATACACAATCG
CCTAGCTGCAGCCCAGGTCTA

>p2_ind1175

TCACTTTCGTTATCCAGATCGGTCATCTGTGGTAAGACTAGAGAAACCGTTCTACTCTATATCCTCATACACAATCG
CCTAGCTGCAGCCCAGGTCTA

>p2_ind4252

TCACTTTCGTTATCCAGATCGGTCATCTGTGGTAAGACTAGAGAAACCGTTCTACTCTATATCCTCATACACAATCG
CCTAGCTGCAGCCCAGGTCTA

>p2_ind1620

TCACTTTCGTTATCCAGATCGGTCATCTGTGGTAAGATTAGAGAAACCGTTCTACTCTATATCCTCATACACAATCG
CCTAGCTGCAGGCCAGGTCTA

>p2_ind4931

TCACTTTCGTTGTCCAGATCGGTCATCTGTGGTAAGACTAGATAAACCGTTCTACTCTATATCCCCATACACAATCG
CCTAGCTGCAGCCCAGGTCTA

>p2_ind1669

TCACTTTCGTTATCCAGATCGGTCATCTGCGGTAAGACTAGTGAAACCGTTCTACTCTATATCCTCATACACAATCG
CCTAGCTGCAGCCCAGGTCTA

>p2_ind4746

TCACTATCGTTATCCAGATCGGTCATCTGTGGTAAGATTAGAGAAACCGTTCTACTCTATATCCTCATACACAATCG
CCTAGCTGCAGCCCAGGTCTA

>p2_ind1007

TCACTATCGTTATCCAGATCGGTCATCTGTGGTAAGATTAGAGAAACCGTTCTACTCTATATCCTCATACACAATCG
CCTAGCTGCAGCCCAGGTCTA

>p2_ind14

TCACTTTCGTTATCCAGATCGGTCATCCGCGGTAAGACTAGAGAAACCGTTCTACTCTATATCCTCATACACAATCG
CCTAGCTGCAGCCCAGGTCTA

>p2_ind603

TCACTATCGTTATCCAGATCGGTCATCTGTGGTAAGACTAGAGAAACCGTTCTACTCTATATCCTCATACACAATCG
CCTAGCTGCAGCCCAGGTCTA

>p2_ind2731

TCACTTTCGTTATCCAGATCGGTCATCTGTGGTAAGACTAGAGAAACCGTTCTACTCTATATCCTCATACACAATCG
CCTAGCTGCAGCCCAGGTCTA

>p2_ind1154

TCACTATCGTTATCCAGATCGGTCATCTGTGGTAAGACTAGAGAAACCGTTCTACTCTATATCCTCATACACAATCG
CCTAGCTGCAGCCCAGGTCTA

>p2_ind200

TCACTTTCGTTATCCAGATCGGTCATCTGTGGTAAGACTAGAGAAACCGTTCTACTCTATATCCTCATACACAATCG
CCTAGCTGCAGCCCAGGTCTA

>p2_ind3351

TCACTTTCGTTATCCAGATCGGTCATCTGTGGTAAGACTAGAGAAACCGTTCTACTCTATATCCTCATATACAATCG
CCTAGCTGCAGCCCATGTCTA

>p2_ind2956

TCATTTTCGTTATCCAGATCGGTCATCTGTGGTAAGACTAGAGAAACCGTTCTACTCTATATCCTCATACACAATCG
CCTAGCTGCAGCCCAGGTCTA

>p2_ind3202

TCACTATCGTTATCCAGATCGGTCATCTGTGTTAAGACTAGAGAAACCGTTCTACTCTATATCCTCATACACAATCG
CCTAGCAGCAGCCCAGGTCTA

>p2_ind2835

TCACTTTCGTTATCCAGATCGGTCATCTGTGGTAAGACTAGAGAAACCGTTCTACTCTATATCCTCATACACAATCG
CCTAGCTGCGGCCCAGGTCTA

>p2_ind2612

TCACTTTCGTAATCCAGATCGGTCATCTGTGGTAAGACTTGAGAATCCGTTCTAATCTATATCCTCATACACAATCG
CCTAGCTGCAGCCCAGGTCTA

>p2_ind391

TCACTTTCGTTATCCAGATCGGTCATCTGTGGTAAGACTAGAGAAACCGTTCTACTCTATATCCTCATACACAATCG
CCTAGCTGCAGCCCAGGTCTA

>p2_ind1974

TCACTTTCGTTATCCAGATCGGTAATCTGCGGTAAGACTAGAGAAACCGTTCTACTCTATATCCTCATACACAATCG
CCTAGCTGCAGCCCAGGTCTA

>p2_ind1925

TCACTTTCGTTATCCAGATCGGTCATCTGTGGTAAGACTAGAGAAACCGTTCTACTCTATATCCTCATATACAATCG
CCTAGCTGCAGCCCATGTCTA

>p2_ind1574

TCACTTTCGTTATCCAGATCGGTCATCTGTGGTAAGACTAGAGTAACCGTTCTACTCTATATCCTCATACACAATCGC
CTAGCTGCAGCCCAGGTCTA

>p2_ind924

TCACTTTCGTTGTCCAGATCGGTCATCTGTGGTAAGACTAGATAAACCGTTCTACTCTATATCCCCATACACAATCAC
CTAGCTGCAGCCCAGGTCTA

>p2_ind1624

TCACTTTCGTTATCCAGATCGGTCATCTGTGGTAAGACTAGAGAAACCGTTCTACTCTATATCCTCATACACAATCG
CCTAGCTGAAGCCCAGGTCTA

>p2_ind4427

TCACTTTCGTTATCCAGATCGGTCATCTGTGGTAAGACTAGAGAAACCGCTCTACTCTATATCCTCATACACAATCG
CCTAGCTGCAGCCCAGGTCTA

>p2_ind951

TCACTTTCGTTATCCAGATCGGTCATCTGTGGTAAGACTAGAGAAACCGCTCTACTCTATATCCTCATACACAATCG
CCTAGCTGCAGCCCAGGTCTA

>p2_ind60

TCACTTTCGTTATCCAGATCGGTCATCCGTGGTAAGACTAGAGAAACTGTTCTACTCTATATCCTCATACACAATCG
CCTAGCTGCAGCCCAGGTCTA

>p2_ind2233

TCACTATCGTTATCCAGATCGGTCATCTGTGGTAAGATTAGAGAAACCGTTCTACTCTAAATCCTCATACACAATCG
CCTAGCTGCAGCCCAGGTCTA

>p2_ind1882

TCACTTTCGTTATCCAGATCGGTCATCTGTGGTAAGACTAGAGAAACCGTTCTACTCTATATCCTCATACACAATCG
CCTAGCTGCAGCCCAGGTCTA

>p2_ind1947

TCACTTTCGTTATCCAGATCGGTCATCTGTGGTAAGACTAGAGAAACCGTTCTACTCTATATCCTCATACACAATCG
CCTAGCTGCAGGCCAGGTCTA

>p2_ind3534

TCACTTTCGTTGTCCAGATCGGTCATCTGTGGTAAGACTAGATAAACCGTTCTACTCTATATCCCCATACACAATCAC
CTAGCTGCAGCCCAGGTCTA

>p2_ind3247

TCACTTTCGTTATCCAGATCGGTAATCTGCGGTAAGACTAGAGAATCCGTTCTACTCTATATCCTCATACACAATCG
CCTAGCTGCAGCCCAGGTCTA

>p2_ind460

TCACTTTCGTTATCCAGATCGGTCATCTGTGGTAAGACTAGAGAAACCGTTCTACTCTATATCCTCATACACAATCG
CCTAGCTGCAGCCTAGGTCTA

>p2_ind4039

TCACTATCGTTATCCAGATCGGTCATCTGTGGTAAGACTAGAGAAACCGTTCTACTCTATATCCTCATACACAATCG
CCTAGCTGCAGCCCAGGTCTA

>p2_ind3226

TCACTTTCGTTATCCAGATCGGTCATCTGTGGTAAGACTAGAGAAACCGTTCTACTCTATATCCTCATACACAATCG
CCTAGCTGCAGCCTAGGTCTA

>p2_ind1376

TCACTTTCGTTATCCAGATCGGTCATCTGTGGTAAGACTAAAGAAACCGTTCTACTCTATATCCTCATACACAATCG
CCTAGCTGCAGCCCAGGTCTA

>p2_ind3846

TCACTTTCGTTATCCAGATCGGTCATCTGTGGTAAGACTAGAGGAACCATTCTACTCTATATCCTCATACACAATCG
CCTAGCTGCAGCCCAGGTCTA

>p2_ind2323

TCACTTTCGTTATCCAGATCGGTCATCTGTGGTAAGACTAGAGAAACCGTTCTACTCTATATCCTCATACACAATCG
CCTAGCTGCAGCCCAGGTCTA

>p2_ind1093

TCACTTTCGTTATCCAGATCGGTCATCTGTGGTAAGACTAGAGAAACCGTTCTACTCTATATCCTCATACACAATCG
CCTAGCTGCAGCCCAGGTCTA

>p2_ind1171

TCACTTTCGTTATCCAGATCGGTCATCTGTGGTAAGACTAGAGGAACCATTCTACTCTATATCCTCATACACAATCG
CCTAGCTGCAGCCCAGGTCTA

>p2_ind4265

TCACTTTCGTTATCCAGATCGGTCATCTGTGGTAAGACTAGAGAAACCGTTCTACTCTATATCCTCATACACAATCG
CCTAGCTGAAGCCCAGGTCTA

>p2_ind4619

TCACTTTCGTTATCCAGATCGGTCATCTGTGGTAAGACTAGAGATACCATCATACTCTATAACCTCATACACAATCG
CCTAGCTGCAGCCCAGGTCTA

>p2_ind2778

TCACTTTCGTTATCCAGATCGGTCATCTGTGGTAAGACTAGAGTAACCGTTCTACTCTATATCCTCATACACAATCGC
CTAGCTGCAGCCCAGGTCTA

>p2_ind969

TCACTTTCGTTATCCAGATCGGTCATCTGTGGTAAGACTAGAGAATCCGTTCTAATCTATATCCTCATACACAATCG
CCTAGCTGCAGCCCAGGTCTA

>p2_ind4885

TCACTTTCGTTATCCAGATCGGTCATCTGTGGTAAGACTAGAGAAACCGTTCTACTCTATATCCTCATACACAATCG
CCTAGCTGCGGCCCAGGTCTA

>p2_ind961

TCACTTTCGTTATCCAGATCGGTCATCTGTGGTAAGACTAGAGAAACCGTTCTACTCTATATCCTCATACACAATCG
CCTAGCTGCAGCCTAGGTCTA

>p2_ind3566

TCACTTTCGTTATCCAGATCGGTCATCTGTGGTAAGACTAGAGAAACCGTTCTACTCTATATCCTCATACACAATCG
CCTAGCTGCAGCCTAGGTCTA

>p2_ind4054

TCACTTTCGTTATCCAGATCGGTCATCTGTGGTAAGACTAGAGAAACCGTTCTACTCTATATCCTCATACACAATCG
CCTAGCTGCAGCCCAGGTCTA

>p2_ind2722

TCATTTTCGTTATCCAGATCGGTCATCTGTGGTAAGACTAGAGGAACCATCATACTCTATATCCTCATACACAATCG
CCTAGCTGCAGCCCAGGTCTA

>p2_ind4005

TCATTTTCGTTATCCAGATCGGTCATCTGTGGTAAGCCTAGAGGAACCATTCTACTCTATATCCTCATACACAATCGC
CTAGCTGCAGCCCAGGTCTA

>p2_ind280

TCATTTTCGTTATCCAGATCGGTCATCTGTGGTAAGACTAGAGGAACCATCATACTCTATAACCTCATACACAATCG
CCTAGCTGCAGCCCAGGTCTA

>p2_ind4386

TCACTTTCGTTATCCAGATCGGTCATCCGCGGTAAGACTAGAGAAACCGTTCTACTCTATATCCTCATACACAATCG
CCTAGCTGCAGCCCAGGTCTA

>p2_ind352

TCACTTTCGTTATCCAGATCGGTCATCTGTGGTAAGACTAGAGTAACCGTTCTACTCTATATCCTCATACACAATCGC
CTAGCTGCAGCCCAGGTCTA

>p2_ind134

TCACTTTCGTTATCCAGATCGGTCATCTGTGGTAAGACTAGAGAAACCGTTCTACTCTATATCCTCATACACAATCG
CCTAGCTGCAGCCCAGGTCTA

>p2_ind4387

TCACTTTCGTTATCCAGATCGGTCATCTGTGGTAAGACTAGAGAAACCGTTCTACTCTATATCCTCATACACAATCG
CCTAGCTGCAGCCCAGGTCTA

>p2_ind1868

TCACTTTCGTTATCCAGATCGGTCATCTGCGGTAAGACTAGAGAAACCGTTCTACTCTATATCCTCATACACAATCG
CCTAGCTGCAGCCCAGGTCTA

>p2_ind96

TCACTTTCGTTATCCAGATCGGTAATCTGCGGTAAGACTAGAGAAACCGTTCTACTCTATATCCTCATACACAATCG
CCTAGCTGCAGCCCAGGTCTA

>p2_ind2192

TCACTTTCGTTATCCAGATCGGTCATCTGTGGTAAGACTAGAGATACCGTTCTACTCTATATCCTCATACACAATCGC
CTAGCTGCAGCCCAGGTCTA

>p2_ind4213

TCACTTTCGTAATCCAGATCGGTCATCCGCGGTAAGACTAGAGAAACCGTTCTACTCTATATCCTCATACACAATCG
CCTAGCTGCAGCCCAGGTCTA

>p2_ind2818

TCACTTTCGTTATCCAGATCGGTCATCTGTGGTAAGACTAGAGTAACCGTTCTACTCTATATCCTCATACACAATCGC
CTAGCTGCAGCCCAGGTCTA

>p2_ind1122

TCACTTGCGTTATCCAGATCGGTTAGCTGTGGTAAGACTAGAGGAACCATTCTACTCTATATCCTCATACACAATCG
CCTAGCTGCAGCCCAGGTCTA

>p2_ind4281

TCACTTTCGTTATCCAGATCGGTCATCTGTGGTAAGACTAGAGAAACCGTTCTACTCTATATCCTCATACACAATCG
CCTAGCTGCAGCCCAGGTCTA

>p2_ind3679

TCACTTTCGTTATCCAGATCGGTCATCTGTGGTAAGACTAGAGAAACCGTTCTACTCTATATCCTCATACACAATCG
CCTAGCTGCAGCCCAGGTCTA

>p2_ind431

TCACTTTCGTTATCCAGATCGGTCATCTGTGGTAAGACTAGAGAAACCGTTCTACTCTATATCCTCATACACAATCG
CCTAGCTGCAGCCTAGGTCTA

>p2_ind4731

TCACTTTCGTTATCCAGATCGGTCATCTGTGGCAAGACTAGAGAATCCGTTCTACTCTATATCCTCATACACAATCG
CCTAGCTGCAGCCCAGGTCTA

>p2_ind1628

TCACTTTCGTTATCCAGATCGGTCATCTGTGGTAAGACTAGAGAAACCGTTCTACTCTATATCCTCATACACAATCG
CCTAGCTGCGGCCCAGGTCTA

>p2_ind4494

TCACTTTCATTATCCAGATCGGTCATCTGTGGTAAGACTAGAGAAACCGTTCTACTCTATATCCTCATACACAATCG
CCTAGCTGCAGCCCAGGTCTA

>p2_ind468

TCACTTTCGTTATCCAGATCGGTCATCTGTGGTAAGACTAGAGAAACCGTTCTACTCTATATCCTCATACACAATCG
CCTAGCTGCAGCCCAGGTCTA

>p2_ind1083

TCACTTTCGTTATCCAGATCGGTCATCTGTGGTAAGACTAGAGAAACCGTTCTACTCTATATCCTCATACACAATCG
CCTAGCTGCAGCCCAGGTCTA

>p2_ind2495

TCACTTTCGTTATCCAGATCGGTCATCTGTGGTAAGACTAGAGAAACCGTTCTACTCTATATCCTCATACACAATCG
CCTAGCTGCAGCCCAGGTCTA

>p2_ind3716

TCCCTATCGTTATCCAGATCGGTCATCTGTGGTAAGACTAGAGAAACCGTTCTACTCTATATCCTCATACACAATCG
CCTAGCTGCAGCCCAGGTCTA

>p2_ind558

TCACTATCGTTATCCAGATCGGTCATCTGTGGTAAGATTAGAGAAACCGTTCTACTCTATATCCTCATACACAATCG
CCTAGCTGCAGCCCAGGTCTA

>p2_ind3836

TCACTTTCGTTATCCAGATCGGTAATCTGCGGTAAGACTAGAGAAACCGTTCTACTCTATATCCTCATACACAATCG
CCTAGCTGCAGCCCAGGTCTA

>p2_ind4533

TCACTTTCGTTATCCAGATCGGTCATCCGCGGTAAGACTAGAGAAACCGTTCTACTCTATATCCTCATACACAATCG
CCTCGCTGCAGCCCAGGTCTA

>p2_ind1707

TCACTTTCGTTATCCAGATCGGTCATCTGTGGTAAGACTAGAGAAACCGTTCTACTCTATATCCTCATACACAATCG
CCTAGCTGCAGCCCAGGTCTA

>p2_ind2268

TCACTTTCGTTATCCAGATCGGTCATCCGCGGTAAGACTAGAGAAACCGCTCTACTCTATATCCTCATACACAATCG
CCTAGCTGCAGCCCAGGTCTA

>p2_ind2277

TCACTTTCATTATCCAGATCGGTCATCTGTGGTAAGACTAGAGAAACCGTTCTACTCTATATCCTCATACACAATCG
CCTAGCTGCAGCCCAGGTCTA

>p2_ind4485

TCACTTTCGTTATCCAGATCGGTCATCTGTGGTAAGACTAGAGAAACCGTTCTACTCTATATCCTCATACACAATCG
CCTAGCTGCAGCCCAGGTCTA

>p2_ind1795

TCACTTTCGTTATCCAGATCGGTCATCTGTGGTAAGACTAGAGAAACCGTTCTACTCTATATCCTCATACACAATCG
CCTAGCTGCAGCCCAGGTCTA

>p2_ind1398

TCACTTTCGTTATCCAGATCGGTCATCTGCGGTAAGACTAGAGAAACCGTTCTACTCTATATCCTCATACACAATCG
CCTAGCTGCAGCCCTGGTCTA

>p2_ind797

TCACTTTCGTAATCCAGATCGGTCATCCGCGGTAAGACTAGAGAAACCGTTCTACTCTATATCCTCATACACAATCG
CCTAGCTGCAGCCCAGGTCTA

>p2_ind3356

TCACTTTCGTTATCCAGATCGGTCATCTGTGGTAAGACTAGAGAAACCGTTCTACTCTATATCCTCATACACAATCG
CCTAGCTGCAGGCCAGGTCTA

>p2_ind2512

TCACTTTCGTTATCCAGATCGGTCATCTGTGGTAAGACTAGAGAAACCGTTCTACTCTATATCCTCATACACAATCG
CCTAGCTGCAGCCCAGGTCTA

>p2_ind487

TCACTTTCGTTATCCAGATCGGTCATCCGCGGTAAGACTAGAGAAACCGCTCTACTCTATATCCTCATACACAATCG
CCTAGCTGCAGCCCAGGTCTA

>p2_ind1497

TCACTTTCGTTATCCAGATCGGTCATCTGTGGTAAGACTAGAGAAACCGCTCTACTCTATATCCTCATACACAATCG
CCTAGCTGCAGCCCAGGTCTA

>p2_ind4241

TCACTTTCGTTATCCAGATCGGTCATCTGTGGTAAGACTAGAGGAACCATTCTACTCTATATCCTCATACACAATCG
CCTAGCTGCAGCCCAGGTCTA

>p2_ind3869

TCACTTTCGTTATCCAGATCGGTCATCTGCGGTAAGACTAGAGAAACCGTTCTACTCTATATCCTCATACACAATCG
CCTAGCTGCAGCCCAGGTCTA

>p2_ind4823

TCACTTTCATTATCCAGATCGGTCATCTGTGGTAAGACTAGAGAAACCGTTCTACTCTATATCCTCATTCACAATCGC
CTAGCTGCAGCCCAGGTCTA

>p2_ind2741

TCACTTTCGTTGTCCAGATCGGTCATCTGTGGTAAGACTAGAGAAACCGTTCTACTCTATATCCTCATACACAATCG
CCTAGCTGCAGCCGAGGTCTA

>p2_ind3626

TCACTTTCGTTATCCAGATCGGTCATCTGTGGTAAGACTAGAGAAACCGTTCTACTCTATATCCTCATACACAATCG
CCTAGCTGCGGCCCAGGTCTA

>p2_ind1866

TCACTTTCGCTATCCAGATCGGTCATCTGTGGTAAGACTAGAGAAACCGTTCTACTCTATATCCTCATACACAATCG
CCTAGCTGCAGGCCAGGTCTA

>p2_ind4456

TCACTTTCGTTATCCAGATCGGTCATCCGCGGTAAGACTAGAGAAACCGTTCTACTCTATATCCTCATACACAATCG
GCTAGCTGCAGCCCAGGTCTA

>p2_ind1685

TCACTTTCGTTATCCAGATCGGTCATCTGTGGTAAGACTAGAGAAACCGCTCTACTCTATATCCTCATACACAATCG
CCTAGCTGCAGCCCAGGTCTA

>p2_ind4876

TCACTTTCGTTATCCAGATCGGTCATCCACGGTAAGACTAGAGAAACCGTTCTACTCTATATCCTCATACACAATCG
CCTAGCTGCAGCCCAGGTCTA

>p2_ind2198

TCACTATCGTTATCCAGATCGGTCATCTGTGGTAAGACTAGAGAAACCGTTCTACTCTATATCCTCATACACAATCG
CCTAGCTGCAGCCCAGGTCTA

>p2_ind4057

TCACTTTCGTTATCCAGATCGGTCATCTGTGGTAAGACTAGAGTAACCGTTCTACTCTATATCCTCATACACAATCGC
CTAGCTGCAGCCCAGGTCTA

>p2_ind579

TCACTTTCGTTATCCAGATCGGTCATCTGCGGTAAGACTAGAGAAACCGTTCTACTCTATATCCTCATACACAATCG
CCTAGCTGCAGCCCAGGTCTA

>p2_ind4221

TCACTTTCGTTATCCAGATCGGTCATCTGTGGTAAGACTAGAGACACCGTTCTACTCTATATACTCATACACAATCG
CCTAGCTGCAGCCCAGGTCTA

>p2_ind3042

TCACTTTCGTTATCCAGATCGGTCATCTGTGGTAAGACTAGAGAAACCGTTCTACTCTATATCCTCATACACAATCG
CCTAGCTGCAGCCCATGTCTA

>p2_ind3048

TCACTTTCGTTATCCAGATCGGTCATCTGTGGTAAGACTAGAGAAACCGTTCTACTCTATATCCTCATACACAATCG
CCTAGCTGCGGCCCAGGTCTA

>p2_ind1201

TCACTTTCGTTATCCAGATCGGTCATCTGTGGTAAGACTAGAGAAACCGCTCTACTCTATATCCTCATACACAATCG
CCTAGCTGCAGCCCAGGTCTA

>p2_ind3999

TCACTTTCGTTATCCAGATCGGTCATCTGTGGTAAGACTAGAGAAACCGCTCTACTCTATATCCTCATACACAATCG
CCTAGCTGCAGCCCAGGTCTA

>p2_ind2159

TCACTATCGTTATCCAGATCGGTCATCTGTGGTAAGATTAGAGAAACCGTTCTACTCTATATCCTCATACACAATCG
CCTAGCTGCAGCCCAGGTCTA

>p2_ind4437

TCACTTTCATTATCCAGATCGGTCATCTGTGGTAAGACTAGAGAAACCGTTCTACTCTATATCCTCATACACAATCG
CCTAGCTGCAGCCCAGGTCTA

>p2_ind890

TCACTTTCGTTATCCAGATCGGTCATCTGTGGTAAGATTAGAGAAACCGTTCTACTCTATATCCTCATACACAATCG
CCTAGCTGCAGGCCAGGTCTA

>p2_ind2573

TCACTTTCGTTATCCAGATCGGTAATCTGCGGTAAGACTAGAGAAACCGTTCTACTCTATATCCTCATACACAATCG
CCTAGCTGCAGCCCAGGTCTA

>p2_ind2710

TCACTTTCGTTATCCAGATCGGTCATCTGTGGTAAGACTAGAGAAACCGTTCTACTCTATATCCTCATACACAATCG
CCTAGCTGCAGCCCAGGTCTA

>p2_ind3796

TCACTTTCGTTATCCAGATCGGTCATCTGTGGTAAGACTAGAGAAACCGTTCTACTCTATATCCTCATACACAATCG
CCTAGCTGCAGCCCAGGTCTA

>p2_ind3965

TCACTTTCGTTATCCAGATCGGTCATCTGTGGTAAGACTAGAGAAACCGTTCTACTCTATATCCTCATACACAATCG
CCTAGCTGCAGCCCAGGTCTA

>p2_ind4460

TCACTTTCGTTATCCAGATCGGTCATCTGTGGTAAGACTAGAGAAACCGTTCTACTCTATATCCTCATACACAATCG
CCTAGCTGCAGCCCAGGTCTA

>p2_ind2929

TCACTTTCGTTATCCAGATCGGTCATCTGTGGTAAGACTAGAGAAACCGTTCTACTCAATATCCTCATACACAATCG
CCTAGCTGCAGCCCAGGTCTA

>p2_ind4954

TCACTTTCGTTATCCAGATCGGTCATCTGTGGTAAGACTAGAGTAACCGTTCTACTCTATATCCTCATACACAATCGC
CTAGCTGCAGCCCAGGTCTA

>p2_ind2026

TCACTTTCGTTATCCAGATCGGTCATCTGTGGTAAGACTAGAGAAACCGTTCTACTCTATATCCTCATACACAATCG
CCTAGCTGCAGCCCAGGTCTA

>p2_ind3415

TCACTTTCGTTGTCCAGATCGGTCATCTGTGGTAAGACTAGATAAACCGTTCTACTCTATATCCCCATACACAATCAC
CTAGCTGCAGCCCAGGTCTA

>p2_ind1298

TCACTTTCGTTATCCAGATCGGTCATCTGTGGTAAGACTTGAGAATCCGTTCTAATCTATATCCTCATACACAATCGC
CTAGCTGCAGCCCAGGTCTA

>p2_ind1994

TCACCTTCGTTATCCAGATCGGTCATCTGCGGTAAGACTAGAGAAACCGTTCTACTCTATATCCTCATACACAATCG
CCTAGCTGCAGCCCAGGTCTA

>p2_ind1087

TCACTTTCGTTATCCAGATCGGTCATCTGTGGTAAGACTAGAGAAACCGTTCTACTCTATATCCTCATACACAATCG
CCTAGCTGCAGCCCAGGTCTA

>p2_ind3970

TCACTTTCATTATCCAGATCGGTCATCTGTGGTAAGACTAGAGAAACCGTTCTACTCTATATCCTCATACACAATCG
CCTAGCTGCAGCCCAGGTCTA

>p2_ind1222

TCACTTTCGTTATCCAGATCGGTCATCTGTGGTAAGACTAGAGAAACCGTTCTACTCTATATCCTCATACACAATCG
CCTAGCTGCAGCGCAGGTCTA

>p2_ind2007

TCACTTTCGTAATCCAGATCGGTCATCTGTGGTAAGACTAGAGAAACCGTTCTACTCTATATCCTCATACACAATCG
CCTAGCTGCAGCCCAGGTCTA

>p2_ind4828

TCACTTTCGTTATCCAGATCGGTCATCTGTGGTAAGACTAGAGAAACCGTTCTACTCTATATCCTCATACACAATCG
CCTAGCTGCAGCCCAGGTCTA

>p2_ind2208

TCACTTTCGTTATCCAGATCGGTCATCCGCGGTAAGACTAGAGAAACCGCTCTACTCTATATCCTCATACACAATCG
CCTAGCTGCAGCCCAGGTCTA

>p2_ind3711

TCACTTTCGTTATTCAGATCGGTCATCTGTGGTAAGACTAGAGAAACCGTTCTACTCTATATCCTCATACACAATCG
CCTAGCTGCAGCCCAGCTCTA

>p2_ind1194

TCACTTTCGTTATCCAGATCGGTCATCTGTGGTAAGACTAGAGAAACCGTTCTACTCTATATCCTCATACACAATCG
CCTAGCTGCAGCCCAGGTCTA

>p2_ind4296

TCACTTTCGTTATCCAGATCGGTCATCTGTGGTAAGACTAGAGAAACCGTTCTACTCTATATCCTCATACACAATCG
CCTAGCTGCAGCCCAGGTCTA

>p2_ind1067

TCACTTTCGTTATCCAGATCGGTCATCTGTGGTAAGACTAGAGAATCCGTTCTAATCTATATCCTCATACACAATCG
CCTAGCTGCAGCCCAGGTCTA

>p2_ind2816

TCACTTTCGTTATCCAGATCGGTAATCTGCGGTAAGACTAGAGAAACCGTTCTACTCTATATCCTCATACACAATCG
CCTAGCTGCAGCCCAGGTCTA

>p2_ind4757

TCACTTTCGTTATCCAGATCGGTCATCTGTGGTAAGACTAGAGAAACCGCTCTACTCTATATCCTCATACACAATCG
CCTAGCTGCAGCCCAGGTCTA

>p2_ind1865

TCACTTTCGTTATCCAGATCGGTCATCTGCGGTAAGACTAGAGAAACTGTTCTACTCTATATCCTCATACACAATCG
CCTAGCTGCAGCCCAGGTCTA

>p2_ind1147

TCACTTTCGTTATCCAGATCGGTCATCTGTGGTAAGACTAGAGAAACCGTTCTACTCAATATCCTCATACACAATCG
CCTAGCTGCAGCCCAGGTCTA

>p2_ind4115

TCACTATCGTTATCCAGATCGGTCATCTGTGGTAAGACTAGAGAAACCGTTCTACTCTATATCCTCATACACAATCG
CCTAGCTGCAGCCCAGGTCTA

>p2_ind2393

TCACTTTCGTTATCCAGATCGGTCATCTGTGGTAAGACTAGAGAAACCGTTCTACTCTATATCCTCATACACAATCG
CCTAGCTGCAGCCCAGGTCTA

>p2_ind3168

TCACTATCGTTATCCAGATCGGTCATCTGTGGTAAGATTAGAGAAACCGTTCTACTCTATATCCTCATACACAATCG
CCTAGCTGCAGCCCAGGTCTA

>p2_ind3285

TCACTATCGTTATCCAGATCGGTCATCTGTGGTAAGACTAGAGAAACCGTTCTACTCTATATCCTCATACACAATCG
CCTAGCTGCAGCCCAGGTCTA

>p2_ind3874

TCACTTTCGTTATCCAGATCGGTCATCTGTGGTAAGACTAGAGAAACCGTTCTACTCTATATCCTCATACACAATCG
CCTAGCTGCAGCCCAGGTCTA

>p2_ind1021

TCACTTTCGTTATCCAGATCGGTCATCTGTGGTAAGACTAGAGAAACCGCTCTACTCTATATCCTCATACACAATCG
CCTAGCTGCAGCCCAGGTCTA

>p2_ind3862

TCATTTTCGTTATCCAGATCGGTCATCTGTGGTAAGACTAGAGGAACCATCATACTCTATAACCTCATACACAATCG
CCTAGCTGCAGCCCAGGTCTA

>p2_ind764

TCACTTTCGTTATCCAGATCGGTCATCTGTGGTAAGACTAGAGAAACCGTTCTACTCTATATCCTCATACACAATCG
CCTAGCTGCAGCCCAGGTCTA

>p2_ind4507

TCACTATCGTTATCCAGATCGGTCATCTGTGGTAAGACTAGAGAAACCGTTCTACTCTATATCCTCATACACAATCG
CCTAGCTGCAGCCCAGGTCTA

>p2_ind3938

TCACTTTCGTTATCCAGATCGGTCATCTGTGGTAAGACTAGAGAAACCGTTCTACTCTATATCCTCATATACAATCG
CCTAGCTGCAGCCCATGTCTA

>p2_ind1599

TCACTTTCGTTATCCAGATCGGTCATCTGTGGTAAGACTAGAGAAACCGTTCTACTCTATATCCTCATACACAATCG
CCTAGCTGCAGCCCAGGTCTA

>p2_ind2400

TCACTTTCGTTATCCAGATCGGTCATCTGTGGTAAGACTAGAGATACCGTTCTACTCTATATCCTCATACACAATCGC
CTAGCTGCAGCCCAGGTCTA

>p2_ind1421

TCACTTTCGTAATCCAGATCGGTCATCCGCGGTAAGACTAGAGAAACCGTTCTACTCTATATCCTCATACACAATCG
CCTAGCTGCAGCCCAGGTCTA

>p2_ind50

TCACTTTCGTTATCCAGATCGGTCATGTGTGGTAAGACTAGAGGAACCATTCTACTCTATATCCTCATACACAATCG
CCTAGCTGCAGCCGAGGTCTA

>p2_ind880

TCACTTTCGTTATCCAGATCGGTCATCTGTGGTAAGACTAGAGAAACCGTTCTACTCTATATCCTCATACACAATCG
CCTAGCTGAAGCCCAGGTCTA

>p2_ind2201

TCACTTTCGTTATCCAGATCGGTAATCTGCGGTAAGACTAGAGAAACCGTTCTACTCTATATCCTCATACACAATCG
CCTAGCTGCAGCCCAGGTCTA

>p2_ind4337

TCACTTTCGTTATCCAGATCGGTCATCTGTGGTAGGACTAGAGAAACCGTTCTACTCTATATCCTCATACACAATCG
CCTAGCTGCAGCCCAGGTCTA

>p2_ind706

TCACTTTCGTTATCCAGATCGGTCATCTGTGGTAAGACTAGAGAAACCGTTCTACTCTATATCCTCATACACAATCG
CCTAGCTGCAGCCCAGGTCTA

>p2_ind1190

TCACTTTCGTTATCCAGATCGGTCATCTGTGGTAAGACTAGAGAAACCGTTCTACTCTATATCCTCATACACAATCG
CCTAGCTGCAGCCCAGGTCTA

>p2_ind2100

TCACTTTCGTTATCGAGATCGGTCATCTGTGGTAAGACTAGAGGAACCATTCTACTCTATATCCTCATACACAATCG
CCTAGCTGCAGCCCAGGTCTA

>p2_ind2212

TCACTTTCGTTATCCAGATCGGTCATCTGTGGTAAGACTAGAGAAACCGTTCTACTCTATATCCTCATACACAATCG
CCTAGCTGCAGCCCAGGTCTA

>p2_ind2193

TCACTTTCGTTATCCAGATCGGTCATCTGTGGTAAGACTAGAGAAACCGTTCTACTCTATATCCTCATACACAATCG
CCTAGCTGCAGCCCAGGTCTA

>p2_ind4368

TCACTTTCGTTATCCAGATCGGTCATCTGTGGTAAGACTAGAGAAACCGTTCTACTCTATATCCTCATACACAATCG
CCTAGCTGCAGCCCAGGTCTA

>p2_ind3589

TCACTTTCGTTATCCAGATCGGTCATCTGTGGTCAGACTAGAGAATCCGTTCTAATCTATATCCTCATACACAATCGC
CTAGCTGCAGCCCAGGTCTA

>p2_ind781

TCACTTTCGTTATCCAGATCGGTCATCTGTGGTAAGACTAGAGGAACCATTCTACTCTATATCCTCATACACAATCG
CCTAGCTGCAGCCCAGGTCTA

>p2_ind395

TCACTTTCGTTATCCAGATCGGTCATCTGTGGCAAGACTAGAGAATCCGTTCTACTCTATATCCTCATACACAATCG
CCTAGCTGCAGCCCAGGTCTA

>p2_ind2807

TCACTTTCGTTATCCAGATCGGTCATCTGTGGTAAGACTAGAGAAACCGTTCTACTCTATATCCTCATACACAATCG
CCTAGCTGCAGCCCAGGTCTA

>p2_ind2365

TCACTTTCGTTATCCAGATCGGTCATCTGTGGTAAGACTAGAGAAACCGTTCTACTCTATATCCTCATACACAATCG
CCTAGCTGCAGCCCAGGTCTA

>p2_ind1593

TCACTTTCGTTATCCAGATCGGTCATCTGTGGCAAGACTAGAGAATCCGTTCTACTCTATATCCTCATACACAATCG
CCTAGCTGCAGCCCAGGTCTA

>p2_ind3921

TCACTATCGTTATCCAGATCGGTCATCTGTGGTAAGATTAGAGAAACCGTTCTACTCTATATCCTCATACACAATCG
CCTAGCTGCAGCCCAGGTCTA

>p2_ind4410

TCACTTTCGTTATCCAGATCGGTCATCTGTGGTAAGACTAGAGAAACCGTTCTACTCTATATCCTCATACACAATCG
CCTAGCTGCAGCCCAGGTCTA

>p2_ind1670

TCACTTTCGTTATCCAGATCGGTCATCTGCGGTAAGACTAGAGAAACCGTTCTACTCTATATCCTCATACACAATCG
CCTAGCTGCAGCCCAGGTCTA

>p2_ind2515

TCACTTTCGTTATCCAGATCGGTCATCTGTGGTAAGACTAGAGAAACCGTTCTACTCTATATCCTCATACACAATCG
CCTAGCTGCAGCCCAGGTCTA

>p2_ind946

TCACTTTCGTTATCCAGATCGGTCATCTGTGGTAAGACTAGAGAAACCGTTCTACTCTATATCCTCATACACAATCG
CCTAGCTGCAGCCCAGGTCTA

>p2_ind1283

TCACTTTCATTATCCAGATCGGTCATCTGTGGTAAGACTAGAGAAACCGTTCTACTCTATATCCTCATACACAATCG
CCTAGCTGCAGCCCAGGTCTA

>p2_ind1951

TCACTTTCGTTATCCAGATCGGTCATCTGTGGTAAGACTAGAGAAACCGTTCTACTCTATATCCTCATACACAATCG
CCTAGCTGCAGCCCAGGTCTA

>p2_ind4269

TCACTTTCGTTATCCAGATCGGTCATCTGTGGTAAGACTAGAGAAACCGTTCTACTCTATATCCTCATACACAATCG
CCTAGCTGCAGCCCAGGTCTA

>p2_ind375

TCACTTTCGTTATCCAGATCGGTCATCTGTGGTAAGACTAGAGGAACCATTCTACTCTATATCCTCATACACAATCG
CCTAGCTGCAGCCCAGGTCTA

>p2_ind121

TCACTTTCGTTATCCAGATCGGTCATCTGTGGTAAGACTAGAGAAACCGTTCTACTCTATATCCTCATACACAATCG
CCTAGCTGCAGCCCAGGTCTA

>p2_ind4555

TCACTTTCGTTATCCAGATCGGTCATCTGTGGTAAGACTAGAGAAACCGTTCTACTCTATATCCTCATACACAATCG
CCTAGCTGCAGCCCAGGTCTA

>p2_ind276

TCACTATCGTTATCCAGATCGGTCATCTGTGGTAAGACTAGAGAAACCGTTCTACTCTATATCCTCATACACAATCG
CCTAGCTGCAGCCCAGGTCTA

>p2_ind3766

TCACTTTCGTTATCCAGATCGGTCATCTGTGGTAAGACTAGAGAAACCGTTCTACTCTATATCCTCATACACAATCG
CCTAGCTGCAGCCCAGGTCTA

>p2_ind4201

TCACTTTCGTTATCCAGATCGGTCATCTGTGGTAAGACTAGAGAAACCGTTCTACTCTATATCCTCATACACAATCG
CCTAGCTGCAGCCCAGGTCTA

>p2_ind1261

TCACTTTCGTTATCCAGATCGGTCATCTGTGGTAAGACTAGAGAAACCGTTCTACTCTATATCCTCATACACAATCG
CCTAGCTGCAGCCCAGGTCTA

>p2_ind4634

TCACTTTCGTTATCCAGATCGGTCATCTGTGGTAAGACTAGAGAAACCGTTCTACTCTATATCCTCATACACAATCG
CCTAGCTGCAGCCTAGGTCTA

>p2_ind4295

TCACTTTCGTTATCCAGATCGGTCATCTGTGGTAAGACTAGAGAAACCGTTCTACTCTATATCCTCATACACAATCG
CCTAGCTGCAGCCCAGGTCTA

>p2_ind2674

TCACTTTCGTTATCCAGATCGGTCATCTGTGGTAAGACTAGAGAAACCGTTCTACTCTATATCCTCATACACAATCG
CCTAGCTGCAGCCCAGGTCTA

>p2_ind3323

TCACTTTCGTTATCCAGATCGGTCATCTGTGGTAAGACTAGAGAAACCGTTCTACTCTATATCCTCATACACAATCG
CCTAGCTGCAGCCTAGGTCTA

>p2_ind295

TCACTTTCGTTATCCAGATCGGTCATCTGTGGTAAGACTAGAGAAACCGTTCTACTCTATATCCTCATACACAATCG
CCTAGCTGCAGCCTAGGTCTA

>p2_ind972

TCACTTTCGTTATCCAGATCGGTCATCTGTGGTAAGACTAGAGAAACCGTTCTACTCTATATCCTCATACACAATCG
CCTAGCTGCAGGCCAGGTCTA

>p2_ind3219

TCACTTTCGTTATCCAGATCGGTCATCTGTGGTAAGACTAGAGAAACCGTTCTACTCTATATCCTCATACACAATCG
CCTAGCTGCAGCCCAGGTCTA

>p2_ind925

TCACTTTCGTTATCCAGATCGGTCATCTGTGGTAAGACTAGAGAAACCGTTCTACTCTATATCCTCATACACAATCG
CCTAGCTGCAGCCCAGGTCTA

>p2_ind988

TCACTTTCGTTATCCAGATCGGTCATCTGTGGTAAGACTAGAGAAACCGTTCTACTCTATATCCTCATACACAATCG
CCTAGCTGCAGCCTAGGTCTA

>p2_ind3155

TCACTTTCGTTATCCAGATCGGTCATCTGTGGTAAGACTAGAGAAACCGTTCTACTCTATATCCTCATACACAATCG
CCTAGCTGCAGCCCAGGTCTA

>p2_ind2150

TCACTTTCGTTATCCAGATCGGTCATCTGTGGTAAGACTAGAGGAACCATTCTACTCTATATCCTCATACACAATCG
CCTAGCTGCAGCCCAGGTCTA

>p2_ind4184

TCACTTTCGTTATCCAGATCGGTCATCTGTGGTAAGACTAGAGAAACCGTTCTACTCTATATCCTCATACACAATCG
CCTAGCTGCAGCCCAGGTCTA

>p2_ind3551

TCACTTTCGTTATCCAGATCGGTCATCTGTGGTAAGACTAGAGAAACCGTTCTACTCTATATCCTCATACACAATCG
CCTAGCTGCAGCCCAGGTCTA

>p2_ind2260

TCACTTTCGTTATCCAGATCGGTCATCTGTGGTAAGACTAGAGAAACCGTTCTACTCTATATCCTCATACACAATCG
CCTAGCTGCGGCCCAGGTCTA

>p2_ind2040

TCACTTTCGTTATCCAGATCGGTCATCTGTGGTAAGACTAGAGATACCGTTCTACTCTATATCCTCATACACAATCGC
CTAGCTGCAGCCCAGGTCTA

>p2_ind1718

TCACTTTCGTTATCCAGATCGGTCATCTGTGGTAAGACTAGAGAAACCGTTCTACTCTATATCCTCATACACAATCG
CCTAGCTGCGGCCCAGGTCTA

>p2_ind3525

TCACTTTCGTTATCCAGATCGGTCATCTGTGGTAAGACTAGATAAACCGTTCTACTCTATATCCTCATACACAATCGC
CTAGCTGCAGCCCAGGTCTA

>p2_ind3838

TCACTATCGTTATCCAGATCGGTCATCTGTGGTAAGATTAGAGAAACCGTTCTACTCTATATCCTCATACACAATCG
CCTAGCTGCAGCCCAGGTCTA

>p2_ind3116

TCACTATCGTTATCCAGATCGGTCATCTGCGGTAAGACTAGAGAAACCGTTCTACTCTATATCCTCATACACAATCG
CCTAGCTGCAGCCCAGGTCTA

>p2_ind619

TCACTTTCGTTATCCAGATCGGTCATCTGTGGTAAGACTAGAGAAACCGTTCTACTCTATATCCTCATACACAATCG
CCTAGCTGCAGCCCAGGTCTA

>p2_ind1354

TCACTTTCGTTATCCAGATCGGTCATCTGTGGTAAGACTAGAGAAACCGTTCTACTCTATATCCTCATACACAATCG
CCTAGCTGCAGCCCAGGTCTA

>p2_ind3492

TCACTTTCGTTATCCAGATCGGTCATCTGTGGTAAGACTAGAGAAACCGTTCTACTCTATATCCTCATACACAATCG
CCTAGCTGCAGCCCAGGTCTA

>p2_ind85

TCACTTTCGTTAGCCAGATCGGTCATCTGTGGTAAGACTAGAGGAACCATTCTACTCTATATCCTCATACACAATCG
CCTAGCTGCAGCCCAGGTCTA

>p2_ind2867

TCACTTTCGTTATCCAGATCGGTCATCTGTGGTAAGACTAGAGAAACCGTTCTACTCTATATCCTCATACACAATCG
CCTAGCTGCAGCCAAGGTCTA

>p2_ind1702

TCACTTTCGTTATCCAGATCGGTCATCTGTGGTAAGACTAGAGAAACCGCTCTACTCTATATCCTCATACACAATCG
CCTAGCTGCAGCCCAGGTCTA

>p2_ind4970

TCACTTTCGTTATCCAGATCGGTCATCTGTGGTAAGACTAGAGAATCCGTTCTAATCTATATCCTCATACACAATCG
CCTAGCTGCAGCCCAGGTCTA

>p2_ind2850

TCACTTTCGCTATCCAGATCGGTCATCTGTGGTAAGACTAGAGAAACCGTTCTACTCTATATCCTCATACACAATCG
CCTAGCTGCAGGCCAGGTCTA

>p2_ind405

TCACTTTCGTTATCCAGATCGGTCATCTGTGGTAAGACTAGAGAAACCGCTCTACTCTATATCCTCATACACAATCG
CCTAGCTGCAGCCCAGGTCTA

>p2_ind742

TCACTTTCGTTATCCAGATCGGTCATCTGTGGTAAGACTAGAGAAACCGTTCTACTCTATATCCTCATACACAATCG
CCTAGCTGCAGCCCAGGTCTA

>p2_ind3129

TCACTTTCGTTATTCAGATCGGTCATCTGTGGTAAGACTAGAGAAACCGTTCTACTCTATATCCTCATACACAATCG
CCTAGCTGCAGCCCAGCTCTA

>p2_ind827

TCACTTTCGTTATCCAGATCGGTCATCTGTGGTAAGATTAGAGAAACCGTTCTACTCTATATCCTCATACACAATCG
CCTAGCTGCAGCCCAGGTCTA

>p2_ind2914

TCATTTTCGTTATCCAGATCGGTCATCTGTGGTAAGACTAGAGGAACCATCATACTCTATAACCTCATACACAATCG
CCTAGCTGCAGCCCAGGTCTA

>p2_ind2559

TCACTTTCGTTATCCAGATCGGTCATCTGTGGTAAGACTAGAGAAACCGTTCTACTCTATATCCTCATACACGATCG
CCTAGCTGCAGGCCAGGTCTA

>p2_ind3230

TCACTTTCATTATCCAGATCGGTCATCTGTGGTAAGACTAGAGAAACCGTTCTACTCTATATCCTCATACACAATCG
CCTAGCTGCAGCCCAGGTCTA

>p2_ind3396

TCACTTTCGTTATCCAGATCGGTCATCTGTGGTAAGACTAGAGAAACCGTTCTACTCTATATCCTCATACACAATCG
CCTAGCTGCAGCCCAGGTCTA

>p2_ind692

TCACTTTCGTTATCCAGATCGGTCATCTGTGGTAAGACCAGAGAAACCGTTTTACTCTATATCCTCATACACAATCG
CCTAGCTGCAGCCCAGGTCTA

>p2_ind1053

TCACTTTCGTTATCCAGATCGGTCATCTGTGGTAAGACTAGAGAAACCGTTCTACTCTATATCCTCATACACAATCG
CCTAGCTGCAGCCCAGGTCTA

>p2_ind1265

TCACTTTCGTTATCCAGATCGGTCATCTGTGGTAAGACTAGAGAAACCGTTCTACTCTATATCCCCATACACAATCG
CCTAGCTGCAGCCCAGGTCTA

>p2_ind2265

TCACTTTCGTTATCCAGATCGGTCATCTGTGGTAAGACTAGAGAAACCGTTCTACTCTATATCCTCATACACAATCG
CCTAGCTGCAGCCCAGGTCTA

>p2_ind2902

TCACTTTCGTTATCCAGATCGGTCATCCGCGGTAAGACTAGAGAAACCGTTCTACTCTATATCCTCATACACAATCG
CCTAGCTGCAGCCCAGGTCTA

>p2_ind4400

TCACTTTCGTTATCCAGATCGGTCATCTGTGGTAAGACTAGAGAAACCGTTCTACTCTATATCCTCATACACAATCG
CCTAGCTGCGGCCCAGGTCTA

>p2_ind935

TCACTTTCGTTATCCAGATCGGTAATCTGCGGTAAGACTAGAGAAACCGTTCTACTCTATATCCTCATACACAATCG
CCTAGCTGCAGCCCAGGTCTA
